# Preferential Co-Expression and Colocalization of rDNA-Contacting Genes with LincRNAs Suggest Their Involvement in Shaping Inter-Chromosomal Interactions with Nucleoli

**DOI:** 10.3390/ijms25126333

**Published:** 2024-06-07

**Authors:** Nickolai A. Tchurikov, Ildar R. Alembekov, Elena S. Klushevskaya, Antonina N. Kretova, Viktoriya N. Lukicheva, Vladimir R. Chechetkin, Galina I. Kravatskaya, Yuri V. Kravatsky

**Affiliations:** 1 Department of Epigenetic Mechanisms of Gene Expression Regulation, Engelhardt Institute of Molecular Biology, Russian Academy of Sciences, Moscow 119334, Russiajiri@eimb.ru (Y.V.K.); 2 Center for Precision Genome Editing and Genetic Technologies for Biomedicine, Engelhardt Institute of Molecular Biology, Russian Academy of Sciences, Moscow 119334, Russia

**Keywords:** rDNA clusters, lincRNAs, differentiation, inter-chromosomal contacts, 4C, gene expression, K562, HEK293T, co-expression

## Abstract

Different developmental genes shape frequent dynamic inter-chromosomal contacts with rDNA units in human and *Drosophila* cells. In the course of differentiation, changes in these contacts occur, coupled with changes in the expression of hundreds of rDNA-contacting genes. The data suggest a possible role of nucleoli in the global regulation of gene expression. However, the mechanism behind the specificity of these inter-chromosomal contacts, which are rebuilt in every cell cycle, is not yet known. Here, we describe the strong association of rDNA-contacting genes with numerous long intergenic non-coding RNAs (lincRNAs) in HEK293T cells and in initial and differentiated K562 cells. We observed that up to 600 different lincRNAs were preferentially co-expressed with multiple overlapping sets of rDNA-contacting developmental genes, and there was a strong correlation between the genomic positions of rDNA-contacting genes and lincRNA mappings. These two findings suggest that lincRNAs might guide the corresponding developmental genes toward rDNA clusters. We conclude that the inter-chromosomal interactions of rDNA-contacting genes with nucleoli might be guided by lincRNAs, which might physically link particular genomic regions with rDNA clusters.

## 1. Introduction

Growing evidence suggests that the 3D organization of chromosomes is the basis for different mechanisms of gene expression regulation. Chromosomal 3D structures are associated with both local and distant intra- and inter-chromosomal interactions. Inter-chromosomal interactions are much rarer than intra-chromosomal interactions and are formed by chromosomal regions that possess highly expressed genes [1,2].

Multiple genes controlling development in human and *Drosophila* cells have been reported to form dynamic inter-chromosomal contacts with rDNA clusters [3,4,5]. It was found that changes occurring in these long-range rDNA–genomic interactions are associated with changes in gene expression patterns during tumorigenesis [6]. Recently, it was reported that during differentiation, changes in rDNA–genomic contacts are coupled with changes in the 3D nets of chromosomal structures around developmental genes [6]. These alterations in the inter-chromosomal contacts of rDNA clusters and 3D structures in particular chromosomal regions lead to changes in the expression of genes located in the corresponding chromosomal domains [7]. About half of the rDNA-contacting genes are co-expressed in human cells [7]. Taken together, these data suggest that 3D structures shaped by rDNA clusters are involved in the global regulation of gene expression [7].

The rDNA-contacting regions in the human genome very often correspond to broad (up to 50 kb) regions decorated by H3K27ac marks that correspond to super-enhancers [8,9,10,11]. It has been speculated that nucleoli might form numerous condensates around themselves, possessing different sets of transcription factors, and that many chromatin loops from different chromosomes possessing developmental genes are merged in these condensates. As a result, different sets of developmental genes are co-expressed [7]. However, it remains unknown as to how these inter-chromosomal rDNA contacts are shaped, how the corresponding genomic regions are targeted to nucleoli surrounded by condensates, how these contacts are rebuilt in every cell cycle, and how they are changed in the course of differentiation.

RNA molecules can originate from any genomic region, and there is evidence in favor of so-called pervasive transcription [12]. These RNA copies can potentially recognize any genomic region and provide the precise navigation mechanism for the regulation of gene expression. LincRNAs are non-coding RNAs longer than 200 nucleotide molecules that can be 5′ capped, spliced, and polyadenylated, and they are characterized by higher developmental stage specificity [13,14]. It was suggested that lincRNAs could act *in trans* via base-pairing interactions and have the potential to regulate the expression of target genes at the transcriptional or translational level with remarkable tissue specificity [15,16].

Here, we present evidence suggesting that lincRNAs have a role in guiding different genomic regions to nucleoli. We found that hundreds of lincRNAs are highly associated and co-expressed with multiple sets of rDNA-contacting genes in HEK293T and K562 cells, and their genomic sites exhibit a strong correlation with the genomic positions of mapped rDNA contacts. We reveal that different sets of lincRNAs could be associated with silent and active genes and that differentiating cells are associated with a great amount of new lincRNAs, as compared with the initial proliferating cells. Our study highlights a novel role of lincRNAs as putative navigator molecules guiding the specific set of developmental genes toward nucleoli. These results could open new lines of investigation into lincRNAs’ functions and 3D chromosomal structures.

## 2. Results

### 2.1. Numerous Overlapping Sets of rDNA-Contacting Genes Are Co-Expressed with Hundreds of LincRNAs in HEK293T Cells

To uncover the associations of rDNA-contacting genes with lincRNAs, we searched for lncHUB lncRNA co-expression in Enrichr (https://maayanlab.cloud/Enrichr/, accessed on 5 June 2024). Adjusted *p*-values (*p*_adj_) were calculated by Enrichr. Table 1 shows the top 10 non-coding RNAs that are co-expressed with rDNA-contacting genes in HEK293T (*p*_adj_ up to 2.62 × 10^−7^). The whole list of associated lincRNAs is shown in Table S1. The database indicates that particular lincRNAs overlap with the essential part of co-expressed genes in human cells. The data also indicate that the majority of these co-expressed genes (more than half) correspond to rDNA-contacting genes. In total, in HEK293T cells, we detected 124 lincRNAs that are co-expressed with overlapping sets of rDNA-contacting genes (Table S1). The sets of overlapping genes include a total of 562 genes. Searches of random sets of human genes did not reveal statistically significant associations with lincRNAs. The data shown in Table S1 indicate that a particular gene can be associated with a number of different lincRNAs. For example, the protocadherin gamma gene cluster, which may be involved in the establishment of specific neuronal connections in the brain, is co-expressed with about 180 lincRNAs; at the same time, the *KCNA6* gene, which is involved in the regulation of neurotransmitter release, is co-expressed with only 4 lincRNAs.

### 2.2. Numerous Overlapping Sets of rDNA-Contacting Genes Are Co-Expressed with about 200 LincRNAs in K562 Cells

The data prompted us to determine whether rDNA-contacting genes in another cell type would also be associated with lincRNAs. It was previously shown that 40% of rDNA-contacting genes are common between HEK293T and K562 cells [7]. These cell types are of different origins: HEK293 cells originate from human embryonic kidney neurons, while K562 cells, which are more differentiated, are obtained from bone marrow and are of the erythroleukemia type. Both cell types have about half of the common rDNA-contacting genes, and we expected that we would also detect a strong association of these genes in K562 cells. This supposition was confirmed, and Table 2 shows that the top 10 lincRNAs are highly associated with rDNA-contacting genes in K562 cells (*p*_adj_ up to 7.26 × 10^−10^). The whole list of associated lincRNAs in K562 cells is shown in Table S2. We detected 198 different lincRNAs that are co-expressed with overlapping sets of rDNA-contacting genes. In total, 1276 rDNA-contacting genes are present in these overlapping sets.

**Figure 1:**
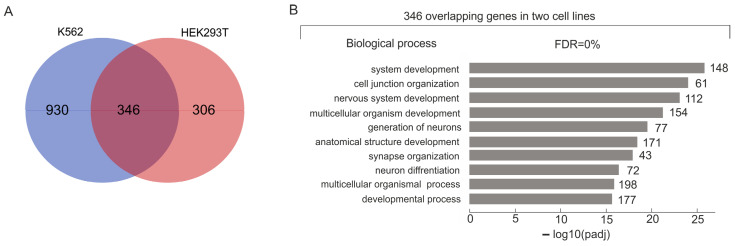
Intersection between the sets of rDNA-contacting genes associated with different lincRNAs in K562 and HEK293T cells. (**A**) Venn diagram showing the intersection between the sets of rDNA-contacting genes associated with different lincRNAs in K562 and HEK293T cells (the list of corresponding genes is shown in Table S3). (**B**) The top 10 detected terms of biological processes associated with overlapping rDNA-contacting genes associated with lincRNAs in both cell lines. Values to the right of the bars show the number of rDNA-contacting genes associated with each term. A full list of corresponding genes and terms is shown in Table S4. The search was performed using g:Profiler (https://biit.cs.ut.ee/gprofiler/gost, accessed 5 June 2024).

Figure 1A shows the intersection between the sets of rDNA-contacting genes associated with different lincRNAs in K562 and HEK293T cells. We detected that 346 overlapping rDNA-contacting genes are highly associated with development in both cell lines (Figure 1B).

### 2.3. Induced Differentiation of K562 Cells Leads to Changes in Both Sets of rDNA-Contacting Genes and Co-Expressed LincRNAs

Next, we attempted to determine how the hemin-induced erythroid differentiation of K562 cells would affect the lincRNA species associated with rDNA-contacting genes. Hemin-induced K562 cell differentiation is known to lead to changes in the inter-chromosomal contacts of rDNA clusters [7]. Here, we observed strong associations of lincRNAs with particular rDNA-contacting genes, which we expected, as well as alterations in the co-expressing lincRNA species upon induced differentiation. The data shown in Table 3 confirm our supposition. The table presents the top 10 lincRNAs highly associated with rDNA-contacting genes in differentiating K562 cells (*p*_adj_ up to 3.09 × 10^−8^). The whole list of associated lincRNAs in these cells is shown in Table S5. We detected 312 different lincRNAs that are co-expressed with overlapping sets of rDNA-contacting genes. In total, 1060 rDNA-contacting genes are present in these overlapping sets.

**Figure 2:**
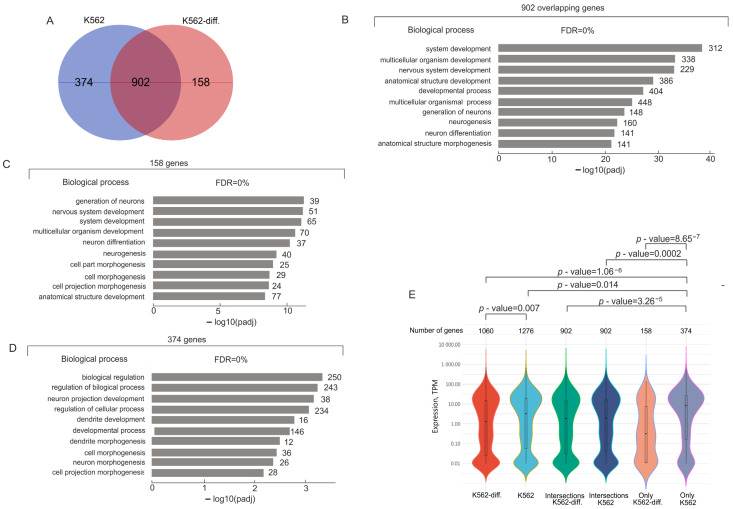
Analysis of rDNA-contacting genes associated with different lincRNAs in K562 cells before and after induced differentiation. (**A**) Venn diagram showing the intersection between sets of rDNA-contacting genes associated with different lincRNAs in K562 before and after induced differentiation (K562 and K562-diff., respectively). The list of corresponding genes is shown in Table S6. (**B**) Top 10 detected terms of biological processes associated with 902 rDNA-contacting genes commonly associated with lincRNAs in initial and differentiated K562 cells. Values to the right of bars show the number of rDNA-contacting genes associated with each term. The full list of corresponding genes and terms is shown in Table S7. The search was performed using g:Profiler (https://biit.cs.ut.ee/gprofiler/gost, accessed 5 June 2024). (**C**) Top 10 detected terms of biological processes associated with 158 rDNA-contacting genes associated with lincRNAs characteristic of only differentiated K562 cells. Values to the right of bars show the number of rDNA-contacting genes associated with each term. The full list of corresponding genes and terms is shown in Table S8. The search was performed using g:Profiler (https://biit.cs.ut.ee/gprofiler/gost, accessed 5 June 2024). (**D**) Top 10 detected terms of biological processes associated with 374 rDNA-contacting genes associated with lincRNAs and characteristic only of initial K562 cells. Values to the right of bars show the number of rDNA-contacting genes associated with each term. The full list of corresponding genes and terms is shown in Table S9. The search was performed using g:Profiler (https://biit.cs.ut.ee/gprofiler/gost, accessed 5 June 2024). (**E**) Violin plots show the distribution of expression of rDNA-contacting genes that are co-expressed with lincRNAs in initial K562 cells or in differentiated K562 cells (K562-diff.). The numbers of corresponding rDNA-contacting genes are shown at the top. *p*-values were assessed as described in Methods.

Mostly, the genes associated with lincRNAs are common in the initial K562 cells and in the differentiating cells (902 genes; Figure 2A). Large groups of these genes are extremely strongly associated with development, up to 2.8 × 10^−36^ (Figure 2B, Table S7). These common genes exhibited about the same expression levels in K562 cells in both states (Figure 2E).

There are also genes that were co-expressed with particular lincRNAs, either in initial K562 cells or in differentiating cells (374 and 158 genes, respectively). The first group of 374 genes is associated with biological regulation and morphogenesis. Mostly, these genes are actively expressed (Figure 2E). Interestingly, the 158 genes that are characteristic of differentiating cells are involved mainly in the same biological processes as the main group of 902 genes (Figure 2B,C). Although the groups of 902 and 158 genes are mainly associated with the same biological processes (Figure 2B,D), part of the latter group was repressed upon hemin-induced differentiation (Figure 2E).

Interestingly, the 374 mostly actively expressed genes in the initial cells and the 158 genes including repressed genes have only one common lincRNA (Figure S1). This result strongly suggests that specific groups of developmental genes that are either activated or repressed may be associated with different sets of lincRNAs.

### 2.4. Expression of LincRNAs Co-Expressing with rDNA-Contacting Genes Detected in HEK293T and K562 Cells

**Figure 3:**
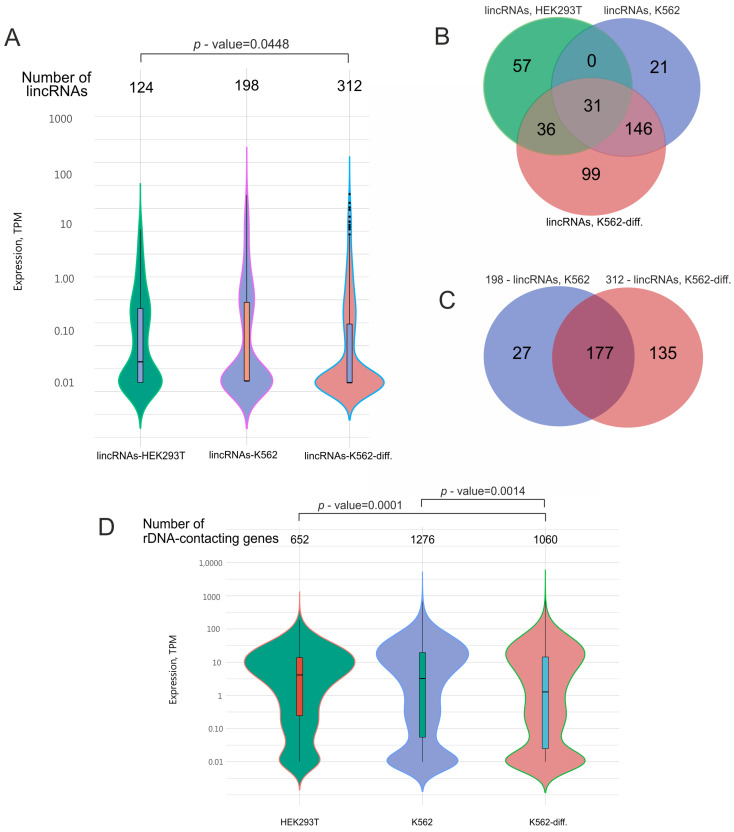
Analysis of lincRNAs that are co-expressed in HEK293T cells and K562 cells before and after induced differentiation (K562-diff.). (**A**) Expression levels of the lincRNAs. The width in each violin curve corresponds to the number of corresponding genes in a region. (**B**) Overlapping lincRNA species that are co-expressed with rDNA-contacting genes in HEK293T cells (124 lincRNA species, corresponding to 652 rDNA-contacting genes), initial K562 cells (198 lincRNA species, corresponding to 1276 rDNA-contacting genes), and differentiating K562 cells (312 lincRNA species, corresponding to 1060 rDNA-contacting genes). The list of corresponding genes is shown in Table S10. (**C**) Overlapping lincRNA species that are co-expressed with rDNA-contacting genes in initial K562 cells (198 lincRNA species, corresponding to 1276 rDNA-contacting genes) and differentiating K562 cells (312 lincRNA species, corresponding to 1060 rDNA-contacting genes). (**D**) Expression levels of rDNA-contacting genes that are co-expressed with the corresponding lincRNAs indicated in (**A**). *p*-values were assessed as described in Methods.

In our study, we detected 634 lincRNAs (Figure 3A, Table S10) that were co-expressed with rDNA-contacting genes in HEK293T cells and in initial or differentiated K562 cells. Our data indicate that about half of the genes detected in HEK293T cells were also present in K562 cells (Figure 1B). Hence, we expected that these cells would also share many lincRNA species.

Figure 3B shows that HEK293T cells had 31 common lincRNAs with initial K562 cells and 67 common lincRNAs with differentiated K562 cells. As expected, K562 cells, in their initial or differentiated state, shared 177 lincRNA species, comprising about 90% of the lincRNAs detected in initial cells and about 57% of those observed in differentiated cells (Figure 3C).

The violin plots in Figure 3 show that these lincRNAs had extremely low expression compared with the expression levels of the corresponding rDNA-contacting genes. For example, the median expression of lincRNAs was more than 10 times lower than that of rDNA-contacting genes. This may reflect the different roles of non-coding and coding RNA and suggests that regulatory RNA is not required in large amounts.

### 2.5. Co-Localization of rDNA-Contacting Genes and Co-Expressing LincRNAs

The initial aim of this study was to determine whether the reproducible inter-chromosomal contacts of particular sets of developmental genes with nucleoli in every cell cycle could be RNA-mediated. One conceivable way for such an RNA guiding mechanism to function involves the close chromosomal neighborhood of genes for both rDNA-contacting genes and co-expressing lincRNAs. In this case, lincRNAs could directly (by way of complementary short regions—seeds—with RNAs located around nucleoli) or indirectly (via a phase separation mechanism) shape contacts with nucleoli and tether the corresponding developmental genes to rDNA clusters. Therefore, we searched for supposed correlations between residing sites of rDNA-contacting genes and corresponding lincRNAs. Recently, a tool for the genome-wide study of colocalization between genomic stretches was described: the Genome Track Colocalization Analyzer (GTCA) [17].

We assessed the colocalization of lincRNAs from the LNCIpedia v.5.2 high-confidence database (see Methods) and the co-expressing rDNA-contacting genes detected in HEK293T cells, initial K562 cells, and differentiated K562 cells. Our analysis revealed statistically significant colocalization of lincRNAs with co-expressing rDNA-contacting genes for all three cell lines (*IO* < −0.5, *p* < 3.5 × 10^-5^; Table 4). This colocalization is represented by the lncRNAs’ centers residing within co-expressing genes at a distance of approximately one quarter of the gene length from the gene boundary (*IO* = −1 if the centers of colocalizing stretches coincide, *IO* = 0 if the centers of the first set of stretches are at the boundaries of the second set, and *IO* = 1 if the centers of stretches are located at the maximum possible distance from each other; see Methods). The analysis was performed using the GTCA [17], as described in Methods.

This colocalization means that lincRNAs are encoded within the introns of the co-expressing rDNA-contacting genes or opposite to a gene DNA strand. We observed that about 82% of the co-expressing rDNA-contacting genes were involved in such relations with the corresponding lincRNA genes. Furthermore, using bedtools (see Methods), we identified a substantial direct overlap between the co-expressing genes and lincRNAs from the LNCIpedia database: 87.8% for HEK293T cells, 85.0% for initial K562 cells, and 86.5% for differentiated K562 cells. These results suggest that lincRNA genes and rDNA-contacting genes are physically linked and that this coupling may reflect their functional connection. This organization supports our view that lincRNAs have a targeting role in inter-chromosomal interactions of developmental genes with nucleoli.

## 3. Discussion

### 3.1. Co-Expression of rDNA-Contacting Genes and Numerous LincRNAs Suggests Their Functional Interaction

We accidentally revealed the statistically significant (*p*-value up to 10^−7^) co-expression of the sets of rDNA-contacting genes from HEK293T and K562 cells with multiple lincRNAs during our analysis of the expression of rDNA-contacting genes (Tables S1–S3). Our interest in this finding increased when we observed that the random groups of human genes of the same size did not exhibit such a correlation. The mere occurrence of active coding and non-coding genes at the same time and in the same cells (co-expression) strongly suggests the possibility of RNA-mediated interactions between them. This co-expression was very intriguing to us, as we recently searched for possible mechanisms behind specific inter-chromosomal interactions of developmental genes with nucleoli.

There are overlapping groups of rDNA-contacting genes corresponding to different lincRNAs. Each gene has more than one corresponding lincRNA. Nevertheless, 158 repressed genes and 374 activated genes (Figure 2) had only one common lincRNA (Figure S1). This means that different lincRNAs could be associated with silent and active genes. Moreover, differentiated K562 cells are associated with a large number of new lincRNAs, as compared with the initial cells (Figure 3). Taken together, these data strongly suggest that there is a functional link between rDNA-contacting developmental genes and this class of non-coding RNAs.

### 3.2. Putative LincRNA-Mediated Mechanisms Guiding Particular rDNA-Contacting Genes toward Nucleoli

Different classes of non-coding RNAs serving as navigator molecules have been studied for decades. Small siRNAs, piRNAs, and miRNAs are guides that attack cognate RNAs in complexes with proteins (RISC) [18,19]. Enhancer RNAs (eRNAs) are longer molecules, up to 1000 nt long, that often act locally in the proximity of corresponding genes, may even originate from foreign DNA located close to enhancer sequences, and work efficiently with target promoters [20]. Unlike eRNAs, lincRNAs are transcribed from special non-coding genes, which are often located in intergenic regions.

Our findings of both preferred co-expression and colocalization of rDNA-contacting genes and lincRNA genes in human cells suggest that lincRNAs might serve as guide molecules to drive genomic regions possessing developmental genes toward nucleoli. We suppose that the short complementary sequences in lincRNAs, resembling seeds in miRNAs, could be complementary to short stretches in nascent rRNA molecules and, in this way, could recognize nucleoli. A preliminary search revealed complementary sequences between different lincRNAs associated with rDNA-contacting genes and different sense and antisense rRNAs, including pRNA, PAPAS, ETS, and others (a detailed analysis will be published separately). Both the co-expression and colocalization of rDNA-contacting genes and particular lincRNAs suggest their physical interaction, while the presence of short sequences that are complementary to rDNA or its transcripts in these lincRNAs additionally suggests the guidance of corresponding genomic regions toward nucleoli. The important property of these sets of genes of shaping frequent contacts with nucleoli is also strong evidence in favor of such guidance.

It was proposed that nucleoli might form liquid–liquid phase separation condensates containing activators or repressors of transcription, which could be involved in the regulation of developmental genes [9,21]. It is known that different chromosomal regions in contact with nucleoli are decorated by broad H3K27ac marks [4,9]. These marks correspond to liquid–liquid phase separation condensates [10,11]. Our finding that different lincRNA species are connected with either silent or active genes (Figure 2A and Figure S1) might indicate that different sets of lincRNAs could guide developmental genes toward different condensates containing activators or repressors of transcription. We suppose that nucleoli shape numerous condensates around them. Thereafter, the navigation of particular chromosomal regions by specific lincRNAs toward nucleoli and, hence, to these condensates provides the mechanism for the concerted co-expression of numerous developmental genes [7,22].

Currently, we are addressing several questions raised in this study by analyzing individual rDNA-contacting genes and corresponding lincRNAs, including detailed research on whether sequences in lincRNAs complementary to rDNA transcripts are capable of navigating genetic reporter constructs toward nucleoli (these data will be published separately).

## 4. Materials and Methods

### 4.1. Cell Culture Growth and Induction of Differentiation with Hemin

Human leukemia K562 cells were obtained from the American Type Culture Collection (ATCC, Manassas, VA, USA). The cells were grown in RPMI 1640 media (PanEco, Moscow, Russia) supplemented with heat-inactivated fetal calf serum (HyClone, Logan, UT, USA), 2 mM glutamine, 250 u/mL penicillin, and 250 μg/mL streptomycin (PanEco, Russia) at a temperature of 37 °C in a humidified atmosphere containing 5% CO_2_. Hemin (50 μM, neoFroxx, Einhausen, Germany) was added to the medium to induce erythroid differentiation, as previously described [23], and cells were incubated further for 108 h. At this concentration, the drug did not affect cell proliferation.

HEK293T cells were grown in DMEM with 10% FBS in a humidified incubator at 37 C and 5% CO_2_ to 80% confluency.

### 4.2. The 4C-rDNA Procedure

The DNA samples used for circular chromosome conformation capture (4C) experiments were isolated according to a previously described procedure [4]. The cells were then fixed in 1.5% formaldehyde, and the nuclei were isolated. Then, digestion with a 6-cutter *EcoR*I enzyme and the ligation of extensively diluted DNA to favor intramolecular ligations were performed. To shorten the ligated DNA fragments, digestion with a 4-cutter *Fae*I endonuclease was performed, followed by the ligation of diluted DNA samples to favor circularization and minimize dimerization. The primers 5′ TCTTTGAAAAAAATCCCAGAAGTGGT 3′ and 5′ AAGTCCAGAAATCAACTCGCCAGT 3′ for 4C-rDNA were selected inside the intergenic spacer (IGS) in rDNA genes, as previously described [9]. The final DNA samples were used for the preparation of DNA libraries, which were subjected to deep sequencing: for the K562 cell line, using Illumina HiSeq 2500 Rapid v2 (Illumina, San Diego, CA, USA) with 150 nt long paired-end reads, and for the HEK293T cell line, using Illumina HiSeq 1500 (Illumina, San Diego, CA, USA) with 250 nt long reads. The 4C-rDNA raw data corresponding to two pairs of biological replicates, initial and differentiated K562 cells, were deposited under accession number GSE232392. The 4C-rDNA raw data corresponding to HEK293T cell replicates were deposited under accession number GSE121413.

### 4.3. Mapping and Processing of 4C-rDNA 4C Data

The raw reads and processed mappings of K562 cells were uploaded to the GEO database under accession no. GSE232392. We obtained the following read numbers: K562 white/untreated cells (17,831,199 for replicate 1 and 18,964,663 for replicate 2) and K562-diff./hemin-treated cells (11,110,278 for replicate 1 and 11,603,232 for replicate 2). The raw reads and processed mappings of HEK293T cells were uploaded to the GEO database under accession no. GSE121413. We obtained 11,944,107 reads for replicate 1 and 11,642,775 for replicate 2.

K562 4C-rDNA-contacting region-specific adapters were removed separately from direct (R1) and reverse (R2) reads using cutadapt 3.5 [24], employing a multi-step procedure as previously described [7]. Only reads trimmed from 4C-specific adapters were retained for subsequent analysis, and their association with 4C was ensured. Direct and reverse reads were then re-paired using the BBTools 38.62 repair.sh script [25] with the “repair” option. Only paired-end reads were preserved for additional analysis: K562/untreated (16,848,786 for replicate 1 and 17,951,920 for replicate 2) and K562-diff./hemin-treated (10,259,396 for replicate 1 and 10,762,480 for replicate 2); all singleton reads were omitted.

For HEK293T, 4C-rDNA-contacting associated region-specific adapters were removed using cutadapt 3.5 [24], employing a two-step procedure as previously described [4]. Only reads trimmed from 4C-specific adapters were retained for further analysis: 11,722,390 for replicate 1 and 11,432,611 for replicate 2.

The alignment of all filtered reads to the genome was performed uniformly for all datasets using the bwa 0.7.17-r1188 MEM method [26]; samtools 1.14 [27] was utilized to exclude unaligned reads from the resulting BAM files (-F4 option) and subsequently re-sort them by their coordinates (samtools sort). Final mappings with genome coordinates, read counts, coverage, and sequences per mapping were obtained using ad hoc in-house Bash and Perl scripts. Replicate quality control was conducted using deepTools2 [28], as previously described [4,7]. The Pearson and Spearman correlation coefficients between all replicates were calculated, resulting in r = 0.99 for both the untreated and hemin-treated 4C-rDNA K562 datasets. The Spearman correlation coefficient values were *ρ* = 0.74 for the untreated and *ρ* = 0.83 for the hemin-treated 4C-rDNA K562 datasets. For the HEK293 datasets, the Pearson and Spearman correlation coefficients between the replicates were r = 0.99 and *ρ* = 0.92, respectively. The calculated correlation coefficients indicate that the replicates obtained during the 4C-rDNA experiments for all three datasets (untreated K562, hemin-treated K562, and HEK293T) were in good mutual agreement. Therefore, we can conclude that the results for 4C-rDNA-associated reads for all three cell lines are reliable.

An intersection of 4C-rDNA-mapped replicates was generated using an ad hoc in-house Bash script employing the intersect and merge methods from bedtools v.2.29.1 [29], as well as the partition, bedmap, and map-id-uniq methods from bedops v.2.4.40 [30]. All reads that were entirely mapped within low-complexity DFAM database regions [31] were excluded from further processing. These specific intersections were identified using the bedtools [29] intersect method with option -f 1.0.

The final output for each cell line comprised a dataset obtained by calculating the intersections between replicates, then excluding reads that were entirely mapped within DFAM regions.

The scripts and data files that were used to perform the described procedures are deposited in a public GitHub repository (https://github.com/lokapal/K562.hemin/tree/main/4C, accessed 15 March 2024).

### 4.4. Quantification of 4C-rDNA-Associated Genes

As described in Section 4.2, we used the 6-cutter *EcoR*I enzyme in the 4C-rDNA experiments. This means that 4C-rDNA contacts were cut at a resolution of ±2.5 kb; therefore, each dataset was projected onto hg19/hg38 gene coordinates, with each 4C contact size/length extended to 5 kb using in-house Bash and Perl scripts employing bedtools [9]. The genomic coordinates were obtained in the form of GTF annotations from Ensembl [32]. Each 4C-rDNA-associated gene was characterized by the corresponding count of associated 4C contact reads, allowing for a ranking of the resulting gene set based on these counts. We specifically identified the most significant 4C-rDNA-associated genes, considering counts of corresponding reads exceeding 100. Consequently, we obtained final lists of 3699 and 2566 4C-rDNA-contacting genes for untreated and hemin-treated K562 cells, respectively [6], and 4920 4C-rDNA-contacting genes for HEK293T cells [4].

### 4.5. RNA-Seq Analysis

All three cell lines (untreated K562, hemin-treated K562, and HEK293T) were processed uniformly to obtain consistent and comparable RNA-Seq datasets. The total RNA was extracted from cells that were lysed with Trizol using a PureLink RNA Micro Kit (Invitrogen, Waltham, MA, USA) in accordance with the manufacturer’s instructions. The RNA quality was checked using a Bioanalyzer and an RNA 6000 Nano Kit (Agilent, Santa Clara, CA, USA). Poly(A)+ RNA was purified using a Dynabeads^®^ mRNA Purification Kit (Ambion, CT, USA). The Illumina library was prepared from poly(A)+ RNA with a NEBNext^®^ Ultra™II RNA Library Prep Kit for Illumina^®^ (NEB, Ipswich, MA, USA), according to the manual. Sequencing was performed with a 50 bp read length. At least 10 million reads were generated for each sample.

Both the raw RNA-Seq reads and the processed gene expression values for untreated and hemin-treated K562 cells were deposited in the GEO repository under accession no. GSE232390. We obtained the following read numbers: K562/untreated cells (10,899,858 for replicate 1 and 10,110,489 for replicate 2) and K562-diff./hemin-treated cells (10,376,685 for replicate 1 and 11,214,611 for replicate 2). The RNA-Seq reads and the processed gene expression values for HEK293T cells were deposited in the GEO repository under accession no. GSE130262. The number of reads for HEK293T cells was 29,342,326 for replicate 1 and 31,029,489 for replicate 2.

The processing of RNA-Seq expression data for all three cell lines was performed uniformly as follows: Trimmomatic 0.39 [33] was applied to remove low-quality reads (Q < 22), excessively short reads (length < 20 bp), and the remaining Illumina TruSeq 3′ SE adapters with the following options: LEADING:18, TRAILING:18, SLIDINGWINDOW:4:22, MINLEN:20, and ILLUMINACLIP:TruSeq3-SE:2:30:10. The trimmed reads were accurately quantified using RSEM 1.3.1 [34] for *H. sapiens* Ensembl v.106 genome annotation with the following options for both replicates separately: --fragment-length-mean 255 --star --calc-ci --ci-memory 30720. The obtained expression values (TPM per gene) were averaged between replicates using an in-house R script.

The scripts and data files used to perform the described procedures were deposited in a public GitHub repository (https://github.com/lokapal/K562.hemin/tree/main/RNASeq, accessed 15 March 2024).

### 4.6. Assessment of Colocalization of rDNA-Contacting LincRNA Co-Expressing Genes and LincRNAs

The colocalization of 4C-rDNA-associated lincRNA co-expressing genes and lincRNAs was assessed using a recently developed tool for the genome-wide study of colocalization between genomic stretches: the Genome Track Colocalization Analyzer (GTCA) [17]. First, the nearest neighbors were determined from the positions of the stretch centers. Then, the colocalization of neighboring stretches of two datasets (A and B in the notation below) was quantitatively characterized using the index of overlapping (*IO*), defined as

(1) $$IO_{k}=\frac{L_{A_{k}B_{k}}-(a_{k}+b_{k})/2}{L_{A_{k}B_{k}}+(a_{k}+b_{k})/2}$$

where *k* refers to the *k*-th pair of nearest neighbors, $L_{A_{k}B_{k}}$ is the distance between the centers of neighboring stretches, and *a_k_* and *b_k_* denote the lengths of these stretches. *IO* varies within the interval (−1, +1), where the extreme boundaries correspond to complete overlapping of stretches with coincident centers (−1) or complete non-overlapping of remote stretches (+1). The mean index of overlapping for *K*-many pairs in total is

(2) $$\bar{IO}\;=\frac{1}{K}\sum_{k=1}^{K}IO_{k}$$

and can be obtained by averaging over a particular genomic configuration, then compared against the counterpart index obtained by averaging over the simultaneous permutation of stretches A and B:

(3) $$IO_{kk^{\prime }}^{\left(2\right)}=\frac{1}{2}\;\left(\frac{L_{A_{k}B_{k}}-(a_{k^{\prime }}+b_{k^{\prime }})/2}{L_{A_{k}B_{k}}+(a_{k^{\prime }}+b_{k^{\prime }})/2}+\frac{L_{A_{k^{\prime }}B_{k^{\prime }}}-(a_{k}+b_{k})/2}{L_{A_{k^{\prime }}B_{k^{\prime }}}+(a_{k}+b_{k})/2}\right)\;;\;k\neq k^{\prime }$$

We applied the complete permutation test to avoid uncertainty in sampling. The significance of deviation between the corresponding mean values is

(4) $$\Delta I=\frac{1}{K}\sum_{k=1}^{K}IO_{k}-\frac{2}{K(K-1)}\sum_{k=1}^{K-1}\sum_{k^{\prime }=k+1}^{K}IO_{kk’}^{\left(2\right)}\equiv \bar{IO}\;-\;<IO{>}_{p}$$

and can be assessed using the following normalization, since the resulting statistics proved to be close to Gaussian [17]:

(5) $$\zeta =\Delta I/\sigma_{eff}(\Delta I)$$

where

(6) $$\sigma_{eff}^{2}(\Delta I)\approx \sigma^{2}(I)/K+2\sigma^{2}(I_{kk^{\prime }})/K-2Cov$$

(7) $$Cov=\frac{2}{K^{2}(K-1)}\sum_{k=1}^{K-1}\sum_{k^{\prime }=k+1}^{K}\left[\left(I_{k}-\bar{I}\;\right)\left(I_{kk^{\prime }}-<I{>}_{p}\right)+\left(I_{k^{\prime }}-\bar{I}\right)\left(I_{kk^{\prime }}-<I{>}_{p}\right)\right]$$

Statistical simulations [17] revealed that ζ can be transformed to a standard Gaussian statistic, with a mean value equal to 0 and a standard deviation equal to 1, via the relevant parametrization; thus, the resulting significance of *IO* can be conveniently estimated using widely applied thresholds (|z|≥1.96, p≤0.05, |z|≥2.58, p≤0.01, etc.).

The described method was implemented in GTCA, a free multi-platform command-line utility available for download at https://ancorr.eimb.ru, accessed 5 June 2024. The colocalization assessment was performed using GTCA with the LNCIpedia v.5.2 [35] high-confidence database and the parameters -pl -ps 100,000 (limiting the correlation pair size to a maximum size of 100 kb).

### 4.7. Analysis of Gene Distribution and Expression Levels

Given that gene expression datasets do not conform to a normal distribution [36], a non-parametric statistical test should be used to compare the distributions of two expression datasets. We previously validated the suitability of the non-parametric, independent, two-group Mann–Whitney U-test for this purpose [17]. Consequently, we applied the Mann–Whitney U-test to assess whether different gene expression subsets could originate from the same distribution. The test revealed that some expression datasets did not originate from the same distribution and were statistically independent. Multiple tests were computed with Holm’s (FWER) correction [37] for multiple comparisons using an R script employing the standard built-in pairwise.wilcox.test() function.

All violin plots were generated using ad hoc in-house R scripts and the following libraries: readr, dplyr [38], powerjoin, ggplot2 [39], hrbrthemes, scales, ggsci, plyranges [40], tidyr, and stringr.

## Figures and Tables

**Table 1:** Overlapping groups of rDNA-contacting genes in HEK293T cells are co-expressed with different long non-coding RNAs. The top 10 lincRNAs are shown. The complete list of 124 lincRNAs is shown in Table S1.

Term	Overlap	Adjusted *p*-Value	Genes
FRMPD3-AS1	55/100	2.623275 × 10^−7^	*PCDHGB7 PCDHGB6 PCDHGB4 CTNND2 PCDHGB3 PCDHGB2 GRIK4 FMN2 GRIK2 TRIM9 OPHN1 LRRTM3 DENND5A EPHB1 PCDHGA8 PCDHGA7 GRID2 PCDHGA6 PCDHGA5 DSCAM PCDHGA4 PCDHGA3 PCDHGA2 TCF12 PCDHGA1 GPR75 PCDHGA9 CDH10 PCDHGB1 ASTN1 ADCYAP1R1 LUZP2 NRXN1 ADCY2 KCNA6 NDRG2 MAP2 LRIG1 NCAM1 KCNN3 LRRC4C GRIA3 GPR158 GRIA4 CLASP2 NTRK2 CADM2 NTRK3 LSAMP QKI GNAO1 PCDHGA10 PCDHGA11 PCDHGA12 APC*
CHL1-AS1	54/100	4.914291 × 10^−7^	*SEMA5A PCDHGB6 PCDHGB4 PCDHGB3 DOCK7 PCDHGB2 SLC35F1 FMN1 EDNRB UBL3 CHL1 HMCN1 ANKS1A DIP2C STK32A SOX5 PCDHGA8 PCDHGA7 PCDHGA6 PCDHGA5 TMEM178B PCDHGA4 PCDHGA3 PCDHGA2 KAZN MITF ANK2 ACTR8 PCDHGA9 NRG3 TBC1D5 PCDHGB1 CREB5 MTPN NLGN1 AKAP6 NKAIN3 ZMAT3 ZC3H13 FARP2 ARNT2 MYEF2 SORT1 S100B CORO2B IGSF11 PCDHGA11 NELL1 PCDHGA12 FMNL2 VPS41 NAPEPLD CNIH3 SLC24A5*
LINC00945	52/100	3.065009 × 10^−6^	*PCDHGB7 PCDHGB6 PCDHGB4 CTNND2 PCDHGB3 PCDHGB2 GRIK4 SLC35F1 DOCK10 TRIM9 LRRTM3 BAALC DENND5A SOX8 PCDHGA8 PCDHGA7 PCDHGA6 GRID2 PCDHGA5 DSCAM PCDHGA4 PCDHGA3 PCDHGA2 PCDHGA1 TCF12 PCDHGA9 BCAN PCDHGB1 CRB1 AKAP6 MAP2 TSPAN7 FYN NCAM1 DISC1 GRIA3 GRIA4 CLASP2 ARNT2 WSCD1 ST8SIA1 LSAMP LHFPL3 CORO2B QKI PCDHGA10 IGSF11 PCDHGA11 CCDC88A PCDHGA12 NOVA1 APBA2*
SLC8A1-AS1	52/100	3.065009 × 10^−6^	*GPR21 STON1 ZBTB20 FRY SLC8A1 MYLK SYNE1 KIAA1109 PGM5 WDR7 SBF2 TEAD1 CC2D2A PRKG1 TNS1 MEF2A EPM2A NCOA1 ZNF483 TRPC4 PDE4D STON1-GTF2A1L EML1 KATNAL1 SETBP1 KCNMA1 WDFY3 PPP1R12B ZNF510 CDKL1 SYNPO2 PRUNE2 PRICKLE2 CACNA1C LPP ATXN1 PLN DPP8 FAM172A CALD1 MYH11 FILIP1L KIDINS220 NDE1 FOXN3 MYO9A FER NBEA CCSER2 MSRB3 MRVI1 FERMT2*
LINC00928	51/100	6.804969 × 10^−6^	*PCDHGB7 PCDHGB6 PCDHGB4 CTNND2 PCDHGB3 PCDHGB2 GRIK4 TRIM9 OPHN1 LRRTM3 SOX8 DENND5A EPHB1 SCN1A PCDHGA8 PCDHGA7 GRID2 PCDHGA6 PCDHGA5 DSCAM PCDHGA4 KCND2 PCDHGA3 PCDHGA2 TCF12 PCDHGA1 PCDHGA9 BCAN FCHSD2 CDH10 PCDHGB1 DSCAML1 CRB1 LUZP2 PCDH15 MAP2 NCAM1 KCNN3 GRIA3 GRIA4 CLASP2 CADM2 LSAMP LHFPL3 QKI PCDHGA10 ATAT1 PCDHGA11 PCDHGA12 NOVA1 APBA2*
LINC01572	51/100	6.804969 × 10^−6^	*PCDHGB7 PCDHGB6 PCDHGB4 PCDHGB3 PCDHGB2 GRIK4 GRIK2 TRIM9 LRRTM3 TNR BAALC DENND5A SOX8 PCDHGA8 PCDHGA7 PCDHGA6 GRID2 PCDHGA5 DSCAM PCDHGA4 PCDHGA3 PCDHGA2 TCF12 PCDHGA1 GPR75 SEZ6L PCDHGA9 FCHSD2 CDH10 PCDHGB1 DSCAML1 ASTN1 SHC3 NRXN1 DRP2 MAP2 NCAM1 LRRC4C CACNG2 GRIA3 GRIA4 CLASP2 CADM2 LHFPL3 QKI PCDHGA10 ATAT1 CCDC88A PCDHGA11 PCDHGA12 APC*
ARHGEF7-IT1	50/100	1.453048 × 10^−5^	*DOCK4 MAML2 DENND5B FAM13C ITSN1 ZBTB20 KIAA1109 OPHN1 SACS DENND5A SBF2 SCAPER TRIM23 NCOA1 KLF12 ZNF483 FBXW11 TCF12 DNM3 ARMCX4 MMP16 SETBP1 PEAK1 DOK6 RAPGEF2 WDFY3 PIK3C3 SIK2 ZNF510 CALCRL CTTNBP2 RNF180 CRMP1 SNTG1 MAP2 NCAM1 MPDZ CLASP2 KIDINS220 MYO9A QKI CCDC144NL NBEA APC DCHS2 TTC3 ST7 FAT3 TCF4 CNTN3*
PRICKLE2-AS1	50/100	1.453048 × 10^-5^	*SYNM TGFB1I1 ITSN1 STON1 MPRIP DIP2C SBF2 TEAD1 PRKG1 TNS1 RBFOX2 AFAP1 ABCC9 STON1-GTF2A1L EML1 KATNAL1 SETBP1 PALLD PEAK1 KCNMA1 WDFY3 TTLL11 PPP1R12B PDZRN3 VCL RABGAP1 SYNPO2 LAMA4 PRUNE2 PRICKLE2 CACNA1C LPP TOR1AIP1 ATXN1 DPP8 CALD1 MYH11 FLNC MPDZ STARD13 NEGR1 KIDINS220 NDE1 FOXN3 FER APC CCSER2 MSRB3 MRVI1 FERMT2*
OPCML-IT1	50/100	1.453048 × 10^−5^	*PCDHGB7 PCDHGB6 MEGF11 PCDHGB4 PCDHGB3 PCDHGB2 GRIK3 GRIK4 SLC35F1 GRIK2 LRRTM3 TMEM108 TNR SOX8 EPHB1 PCDHGA8 PCDHGA7 GRID2 PCDHGA6 PCDHGA5 DSCAM PCDHGA4 PCDHGA3 PCDHGA2 TCF12 PCDHGA1 PCDHGA9 BCAN PCDHGB1 DSCAML1 CRB1 PCDH15 CRMP1 MAP2 FYN NCAM1 GRIA3 GRIA4 OPCML CA10 CADM2 TMEM132B LSAMP LHFPL3 PCDHGA10 ATAT1 PCDHGA11 PCDHGA12 NOVA1 APBA2*
LINC02283	49/100	3.357257 × 10^−5^	*PCDHGB7 PCDHGB6 PCDHGB4 MEGF11 PCDHGB3 PCDHGB2 GRIK4 GRIK2 TRIM9 LRRTM3 TNR SOX8 DENND5A EPHB1 PCDHGA8 PCDHGA7 PCDHGA6 GRID2 PCDHGA5 DSCAM PCDHGA4 PCDHGA3 PCDHGA2 TCF12 PCDHGA1 PCDHGA9 BCAN CDH10 PCDHGB1 DSCAML1 CRB1 PCDH15 CRMP1 MAP2 NCAM1 FYN GRIA3 GRIA4 CLASP2 BTBD17 CADM2 LSAMP LHFPL3 QKI PCDHGA10 ATAT1 PCDHGA11 PCDHGA12 NOVA1*

**Table 2:** Overlapping groups of rDNA-contacting genes in K562 cells are co-expressed with different long non-coding RNAs. The top 10 lincRNAs are shown. The complete list of 198 lincRNAs is shown in Table S2.

Term	Overlap	Adjusted *p*-Value	Genes
PRICKE2-AS3	51/100	7.261056 × 10^−10^	*CHD9 ZBTB20 SLC9C1 SYNE1 AKAP11 ZNF407 TEAD1 PRKG1 RALGAPA1 VPS13C ATRX RC3H1 VPS13B ASH1L STON1-GTF2A1L EML1 SETBP1 PEAK1 KCNMA1 WDFY3 BIRC6 MACF1 KMT2C PRICKLE2 RGPD5 RGPD8 UBR1 CACNA1C LPP PCNX1 ATXN1 HECTD2 CALD1 EVI5 SVIL MBD5 MON2 ERCC6L2 LNPEP PLEKHA3 HOOK3 PHC3 MYO9A HIPK3 FER AGO3 NFIA SLMAP CCSER2 BRWD1 KIAA0825*
LINC02827	49/100	7.359774 × 10^−9^	*KDM5A PATJ CHD6 TRPS1 POTED SAMD12 PRMT8 CERS6 RALGAPA1 ZNF160 MRTFB TBC1D9 VPS13B ASH1L EDAR KIAA1217 SPOPL LMX1B ZNF236 SHANK2 GREB1L RGPD6 RGPD5 RGPD8 FAM214A TMEM241 MIPOL1 CRACR2A ADAMTS18 NSD1 KHDC4 SLC25A21 APBB2 MARCHF6 ANKRD26 AUTS2 ANKRD30B ANKRD30A LRBA DNAH14 DEFB108B ESR1 GON4L TTC6 KCNS3 SP3 TASOR2 NEK10 KIAA0825*
GSN-AS1	46/100	2.499656 × 10^−7^	*MACF1 ANKRD36 KMT2C TULP4 FMN1 ASAP1 LPP DOCK10 STK10 SRGAP2C AKAP13 FYCO1 ZFYVE26 DSTYK ATXN1 SGCD POTEJ ZNF407 ABL2 HMCN1 MAP4 ERC1 SRGAP2 EVI5 TEAD1 SRGAP2B KIRREL1 MBD5 ERCC6L2 DENND2B SAMD4A MYO5A VPS13B MITF MYO9B ASH1L LHFPL2 TANC1 FER NIN PEAK1 SLMAP CDC42EP3 BIRC6 VCL TNRC6B*
LRRC7-AS1	46/100	2.499656 × 10^−7^	*ADCYAP1R1 GABRB1 RTN1 CTNND2 NRXN1 KLHL32 SLC1A2 OTUD7A ADAM22 FMN2 HTR2A NDRG2 KIAA0513 GRM5 BRINP1 FUT9 MAP2 NCS1 CHN1 DLGAP1 NCAM1 ERC2 WASF3 OPCML ARNT2 DTNA CADM2 NTRK3 TMOD2 LSAMP ANK2 SLC39A12 GRIN2B SYN2 SNAP91 GABRG1 CNKSR2 TTLL7 DLG2 SYNJ1 APC ADGRB3 LRRC7 PPP2R2B ASTN1 RAPGEF4*
TUB-AS1	45/100	7.540575 × 10^−7^	*NFAT5 TBC1D19 ACSS3 CUL5 PPM1L PRUNE2 ZBTB20 ACSM2A SLC5A12 PEPD TRHDE TSPAN33 ACSM2B HS6ST3 CDH7 SNX29 AP5M1 KIAA1328 PLCZ1 KIF21A THSD7A SLC16A9 SLC17A1 RNF152 TINAG CUBN KL STPG2 PDE4D SLC2A13 WDR72 UNC5D ARHGAP24 MOB1B MSRA PLCXD3 CLCN5 SYT10 RRAGD LRRC9 WDFY3 CNTN3 DGKI CNTNAP5 CPEB4*
LINC02720	44/100	2.277643 × 10^−6^	*UHRF1BP1L CUL5 PPM1L ZBTB20 ITPR2 LDLRAD4 FAM214A TMEM241 HS6ST3 CDH7 KIAA1328 PLCZ1 ERBB4 TRPS1 FAM241A APBB2 THSD7A RNF152 RALGPS2 VAV3 UNC13C MON2 STPG2 LRBA TBC1D9 GFRA1 SCAMP1 PRLR ESR1 PBX1 MOB1B INPP4B RABEP1 SYT10 CCNG2 LRRC9 NEK10 SPOPL ZNF678 LMX1B BRWD1 KIAA0825 DGKI CNTNAP5*
ARHGEF7-IT1	43/100	5.289800 × 10^−6^	*DOCK4 MAML2 PPM1L CTTNBP2 ADAM22 ZBTB20 AKAP11 SNTG1 MAP2 SACS NCAM1 SCAPER MPDZ TRIM23 ZNF462 KLF12 MBD5 TMOD2 TCF12 KIAA0232 IL17RD MYO9A PJA2 PYGO1 DNM3 ZEB1 NBEA MMP16 SETBP1 APC ADGRB3 PEAK1 NFIB TTC3 RAPGEF2 WDFY3 FAT3 TCF4 CNTN3 PIK3C3 FAT4 ARHGEF7 PAFAH1B1*
MRPS30-DT	43/100	5.289800 × 10^−6^	*PTPRT CLSTN2 KCNE4 LDLRAD3 AFF3 FAM214A TMEM241 RERG SLC7A2 TMEM25 ERBB4 TRPS1 FAM241A SLC39A6 POTED APBB2 RALGPS2 VAV3 KDM4B CERS6 SIAH2 LRBA MRTFB CYBRD1 TBC1D9 ELP2 GFRA1 PRLR ESR1 PBX1 MED13L RABEP1 TSPAN13 NAT1 TTC6 KIF16B BCL2 NEK10 SPOPL LMX1B BMPR1B FSIP1 FGF10*
PGR-AS1	43/100	5.289800 × 10^−6^	*CLSTN2 ZFAND4 SLC40A1 GREB1L PIK3R3 LDLRAD4 EFCAB6 AFF3 FAM214A GALNT10 TMEM241 RERG TMEM25 ADAMTS19 ERBB4 TRPS1 NRIP1 FAM241A SPIN1 ST8SIA6 APBB2 RALGPS2 VAV3 ANKRD26 KDM4B ANKRD30B EYA2 SIAH2 LRBA GREB1 MRTFB TBC1D9 GFRA1 PRLR ESR1 PBX1 DCDC1 INPP4B RABEP1 NAT1 NEK10 SPOPL LMX1B*
LINC00472	42/100	1.328224 × 10^−6^	*CYFIP2 NFAT5 PATJ PRKAA2 CUL5 STXBP4 KMT2C RGPD6 ZBTB20 RGPD5 RGPD8 EFCAB6 SYNE2 SYNE1 KIAA1328 ERBB4 ZNF407 THSD7A RNF152 ZNF124 ANKRD26 MBD5 MON2 ARHGEF12 STPG2 ERCC6L2 LRBA DNAH14 VPS13B LNPEP ASH1L PPP2R3A PHC3 MYO9A MOB1B ZNF717 SETBP1 WDFY3 UTRN BRWD1 KIAA0825 DOCK1*

**Table 3:** Overlapping groups of rDNA-contacting genes in differentiating K562 cells are co-expressed with different long non-coding RNAs. The top 10 lincRNAs are shown. The complete list of 312 lincRNAs is shown in Table S5.

Term	Overlap	Adjusted *p*-Value	Genes
RICKLE2-AS3	40/100	3.089393 × 10^−8^	*MACF1 CHD9 KMT2C PRICKLE2 ZBTB20 RGPD5 RGPD8 UBR1 CACNA1C SLC9C1 LPP PCNX1 ATXN1 AKAP11 ZNF407 EVI5 TEAD1 PRKG1 SVIL MBD5 MON2 VPS13C RC3H1 VPS13B LNPEP PLEKHA3 STON1-GTF2A1L EML1 PHC3 MYO9A HIPK3 FER NFIA PEAK1 SLMAP KCNMA1 CCSER2 WDFY3 BIRC6 KIAA0825*
LINC02827	39/100	4.695381 × 10^−8^	*PATJ CHD6 GREB1L RGPD6 RGPD5 RGPD8 FAM214A TMEM241 MIPOL1 PCNX2 POTED KHDC4 SLC25A21 APBB2 SAMD12 MARCHF6 ANKRD26 PRMT8 CERS6 AUTS2 ANKRD30B ANKRD30A ZNF160 LRBA DNAH14 MRTFB TBC1D9 VPS13B ESR1 EDAR KIAA1217 TTC6 KCNS3 SP3 TASOR2 NEK10 ZNF236 KIAA0825 SHANK2*
LRRC7-AS1	39/100	4.695382 × 10^−8^	*GABRB1 RTN1 CTNND2 NRXN1 KLHL32 SLC1A2 OTUD7A FMN2 KIAA0513 GRM5 BRINP1 FUT9 DLGAP1 NCAM1 ERC2 SH3GL2 WASF3 OPCML ARNT2 DTNA CADM2 NTRK3 TMOD2 LSAMP SLC39A12 GRIN2B SYN2 SNAP91 GABRG1 CNKSR2 TTLL7 DLG2 SYNJ1 APC ADGRB3 LRRC7 PPP2R2B ASTN1 RAPGEF4*
LINC00472	37/100	3.694256 × 10^−7^	*CYFIP2 NFAT5 PATJ PRKAA2 CUL5 STXBP4 KMT2C RGPD6 ZBTB20 RGPD5 RGPD8 EFCAB6 SYNE2 KIAA1328 ERBB4 ZNF407 SLC16A7 THSD7A RNF152 ANKRD26 MBD5 MON2 ARHGEF12 STPG2 LRBA DNAH14 VPS13B LNPEP PPP2R3A PHC3 MYO9A MOB1B ZNF717 WDFY3 UTRN KIAA0825 DOCK1*
LINC01651	37/100	3.694256 × 10^−7^	*CRB1 GALNT13 PID1 PHLPP1 CTTNBP2 MEGF11 NRXN1 PCDH15 GRIK4 GRIK2 TRIM9 RASSF2 CDH20 DNER TNR NCAM1 KIF21B CSMD3 SOX6 LRRC4C CACNG2 GRIA4 RFTN2 GRID2 DSCAM SPHKAP CADM2 TCF12 LSAMP LHFPL3 SEZ6L FCHSD2 ADGRB3 SMOC1 DSCAML1 UNC79 ASTN1*
VWC2L-IT1	37/100	3.694256 × 10^−7^	*DGKG GRIA1 GABRB1 GALNT13 SHC3 RTN1 KCNC1 MEGF11 NRXN1 KLHL32 SLC35F1 GRIK2 GRM5 RASSF2 SNTG1 TNR LRRC4C CACNG2 SH3GL2 GPR158 GRIA4 OPCML CADM2 TMEM132B TMOD2 FAM219A LHFPL3 SEZ6L SNAP91 EPN2 DNM3 SYNJ1 APC ADGRB3 PPP2R2B UNC79 ASTN1*
GSN-AS1	37/100	3.694256 × 10^−7^	*MACF1 ANKRD36 KMT2C FMN1 ASAP1 LPP DOCK10 SRGAP2C AKAP13 FYCO1 ATXN1 SGCD ZNF407 ABL2 HMCN1 ERC1 SRGAP2 EVI5 TEAD1 SRGAP2B KIRREL1 MBD5 RBFOX2 DENND2B SAMD4A MYO5A VPS13B MITF LHFPL2 TANC1 FER PEAK1 SLMAP CDC42EP3 BIRC6 VCL TNRC6B*
SRGAP3-AS1	36/100	1.040485 × 10^−7^	*GALNT13 RTN1 CTNND2 NRXN1 KLHL32 GRIK4 NALCN NPAS3 NKAIN3 CDH20 LRIG1 TNR DLGAP1 NCAM1 SRGAP3 LRRC4C WASF3 GRIA4 RFTN2 ARNT2 GRID2 DSCAM KCND2 CADM2 TMEM132B NTRK3 TMOD2 TCF12 LSAMP MAPK8IP1 DNM3 APC ADGRB3 TBC1D5 PPP2R2B ASTN1*
PRICKLE2-AS1	36/100	1.040485 × 10^−6^	*RABGAP1 PRICKLE2 CACNA1C LPP FYCO1 ATXN1 AKAP11 MPRIP ABL1 DIP2C MPDZ TEAD1 PRKG1 SVIL STARD13 RBFOX2 NEGR1 AFAP1 SHISAL1 ABCC9 FOXN3 STON1-GTF2A1L EML1 PJA2 FER ARHGAP31 APC PEAK1 SLMAP KCNMA1 CCSER2 WDFY3 PPP1R12B PDZRN3 VCL DDR2*
RERG-AS1	36/100	1.040485 × 10^−6^	*PATJ TBC1D19 ACSS3 PRKAA2 CUL5 ANKRD36 ZBTB20 KIAA1328 ZNF407 POTED SLC16A7 THSD7A RNF152 ANKRD26 MON2 MBD5 STPG2 ANKRD30B ANKRD30A DNAH14 VPS13B KLHL3 PHC3 MYO9A LRP1B MOB1B INPP4B TBC1D1 ZNF717 RCAN2 ERP27 WDFY3 KIAA0825 DGKI ANKRD36B CPEB4*

**Table 4:** Colocalization of rDNA-contacting genes and co-expressing lincRNAs from the LNCIpedia high-confidence database, assessed using the Genome Track Colocalization Analyzer (Section 4.6).

rDNA-Contacting Genes From	*IO*	*z*(*IO*)	Colocalization	*p*(*IO*)	Pairs	Number of rDNA-Contacting Genes	Number of LincRNA Genes
HEK293T	−0.5458	−5.1513	+	<1 × 10^-6^	541	656	126,454
K562	−0.5077	−4.9922	+	0.000001	1091	1276	126,454
K562-hemin	−0.5449	−4.1379	+	0.000035	880	1060	126,454

*IO*, index of overlapping. *IO* < 0 indicates that lincRNAs’ stretch centers are located inside the genes. *IO* varies from −1 (complete overlapping of stretches with coinciding centers) to +1 (complete non-overlapping of remote stretches). Correlation pair sizes were limited to 100 kb.

## Data Availability

4C-rDNA experimental datasets are available in GEO database for K562 cells and HEK293T cells under Accession Numbers GSE232392 and GSE121413, respectively. RNA-Seq experimental datasets are available in GEO database for K562 cells and HEK293T cells under Accession Numbers GSE232390 and GSE130262, respectively.

## Supplementary Materials

The supporting information can be downloaded at: https://www.mdpi.com/article/10.3390/ijms25126333/s1.
