# Supplementary material for: Preferential Co-Expression and Colocalization of rDNA-Contacting Genes with LincRNAs Suggest Their Involvement in Shaping Inter-Chromosomal Interactions with Nucleoli
Source: Int J Mol Sci. 2024 Jun 7;25(12):6333. doi: 10.3390/ijms25126333 (PMC11204237; doi:10.3390/ijms25126333)
Supplement: Supplementary file 1 [file ijms-25-06333-s001.zip › ijms-2972485-supplementary/Supplemental Information-10-5--2024.pdf]

Supplemental Information for:

Preferential co-expression and colocalization of rDNA-contacting genes with lincRNAs suggest their involvement in shaping inter-chromosomal interactions with nucleoli

Nickolai A. Tchurikov,[1*] Ildar R. Alembekov[1], Elena S. Klushevskaya[1], Antonina N. Kretova[1], Viktoriya N. Lukicheva[1], Vladimir R. Chechetkin[1], Galina I. Kravatskaya[1], Yuri V. Kravatsky[1,2]

[1] Department of Epigenetic Mechanisms of Gene Expression Regulation, Engelhardt Institute of Molecular Biology Russian Academy of Sciences, Moscow, 119334, Russia
[2] Center for Precision Genome Editing and Genetic Technologies for Biomedicine, Engelhardt Institute of Molecular Biology Russian Academy of Sciences, Moscow, 119334, Russia

*Corresponding author.

This PDF file includes:

Tables S1–S10

Figure S1

Table S1. Overlapping groups of rDNA-contacting gene in HEK293T cells are co-expressed with 124 different long non-coding RNAs.

Term	Overlap	Adjusted P-value	Genes
FRMPD3-AS1	55/100	2.6232750318341253E-7	*PCDHGB7;PCDHGB6;PCDHGB4;CTNND2;PCDHGB3;PCDHGB2;GRIK4;FMN2;GRIK2;TRIM9;OPHN1;LRRTM3;DENND5A;EPHB1;PCDHGA8;PCDHGA7;GRID2;PCDHGA6;PCDHGA5;DSCAM;PCDHGA4;PCDHGA3;PCDHGA2;TCF12;PCDHGA1;GPR75;PCDHGA9;CDH10;PCDHGB1;ASTN1;ADCYAP1R1;LUZP2;NRXN1;ADCY2;KCNA6;NDRG2;MAP2;LRIG1;NCAM1;KCNN3;LRRC4C;GRIA3;GPR158;GRIA4;CLASP2;NTRK2;CADM2;NTRK3;LSAMP;QKI;GNAO1;PCDHGA10;PCDHGA11;PCDHGA12;APC*
CHL1-AS1	54/100	4.914290492803087E-7	*SEMA5A;PCDHGB6;PCDHGB4;PCDHGB3;DOCK7;PCDHGB2;SLC35F1;FMN1;EDNRB;UBL3;CHL1;HMCN1;ANKS1A;DIP2C;STK32A;SOX5;PCDHGA8;PCDHGA7;PCDHGA6;PCDHGA5;TMEM178B;PCDHGA4;PCDHGA3;PCDHGA2;KAZN;MITF;ANK2;ACTR8;PCDHGA9;NRG3;TBC1D5;PCDHGB1;CREB5;MTPN;NLGN1;AKAP6;NKAIN3;ZMAT3;ZC3H13;FARP2;ARNT2;MYEF2;SORT1;S100B;CORO2B;IGSF11;PCDHGA11;NELL1;PCDHGA12;FMNL2;VPS41;NAPEPLD;*

			CNIH3;SLC24A5
LINC00945	52/100	3.0650094424632597E-6	*PCDHGB7;PCDHGB6;PCDHGB4;CTNND2;PCDHGB3;PCDHGB2;GRIK4;SLC35F1;DOCK10;TRIM9;LRRTM3;BAALC;DENND5A;SOX8;PCDHGA8;PCDHGA7;PCDHGA6;GRID2;PCDHGA5;DSCAM;PCDHGA4;PCDHGA3;PCDHGA2;PCDHGA1;TCF12;PCDHGA9;BCAN;PCDHGB1;CRB1;AKAP6;MAP2;TSPAN7;FYN;NCAM1;DISC1;GRIA3;GRIA4;CLASP2;ARNT2;WSCD1;ST8SIA1;LSAMP;LHFPL3;CORO2B;QKI;PCDHGA10;IGSF11;PCDHGA11;CCDC88A;PCDHGA12;NOVA1;APBA2*
SLC8A1-AS1	52/100	3.0650094424632597E-6	*GPR21;STON1;ZBTB20;FRY;SLC8A1;MYLK;SYNE1;KIAA1109;PGM5;WDR7;SBF2;TEAD1;CC2D2A;PRKG1;TNS1;MEF2A;EPM2A;NCOA1;ZNF483;TRPC4;PDE4D;STON1-GTF2A1L;EML1;KATNAL1;SETBP1;KCNMA1;WDFY3;PPP1R12B;ZNF510;CDKL1;SYNPO2;PRUNE2;PRICKLE2;CACNA1C;LPP;ATXN1;PLN;DPP8;FAM172A;CALD1;MYH11;FILIP1L;KIDINS220;NDE1;FOXN3;MYO9A;FER;NBEA;CCSER2;MSRB3;MRVI1;FERMT2*
LINC00928	51/100	6.8049684634282214E-6	*PCDHGB7;PCDHGB6;PCDHGB4;CTNND2;PCDHGB3;PCDHGB2;GRIK4;TRIM9;OPHN1;LRRTM3;SOX8;DENND5A;EPHB1;SCN1A;PCDHGA8;PCDHGA7;GRID2;PCDHGA6;PCDHGA5;DSCAM;PCDHGA4;KCND2;PCDHGA3;PCDHGA2;TCF12;PCDHGA1;PCDHGA9;BCAN;FCHSD2;CDH10;PCDHGB1;DSCAML1;CRB1;LUZP2;PCDH15;MAP2;NCAM1;KCNN3;GRIA3;GRIA4;CLASP2;CADM2;LSAMP;LHFPL3;QKI;PCDHGA10;ATAT1;PCDHGA11;PCDHGA12;NOVA1;APBA2*
LINC01572	51/100	6.8049684634282214E-6	*PCDHGB7;PCDHGB6;PCDHGB4;PCDHGB3;PCDHGB2;GRIK4;GRIK2;TRIM9;LRRTM3;TNR;BAALC;DENND5A;SOX8;PCDHGA8;PCDHGA7;PCDHGA6;GRID2;PCDHGA5;DSCAM;PCDHGA4;PCDHGA3;PCDHGA2;TCF12;PCDHGA1;GPR75;SEZ6L;PCDHGA9;FCHSD2;CDH10;PCDHGB1;DSCAML1;ASTN1;SHC3;NRXN1;DRP2;MAP2;NCAM1;LRRC4C;CACNG2;GRIA3;GRIA4;CLASP2;CADM2;LHFPL3;QKI;PCDHGA10;ATAT1;CCDC88A;PCDHGA11;PCDHGA12;APC*
ARHGEF7-IT1	50/100	1.4530475903009115E-5	*DOCK4;MAML2;DENND5B;FAM13C;ITSN1;ZBTB20;KIAA1109;OPHN1;SACS;DENND5A;SBF2;SCAPER;TRIM23;NCOA1;KLF12;ZNF483;FBXW11;TCF12;DNM3;ARMCX4;MMP16;SETBP1;PEAK1;DOK6;RAPGEF2;WDFY3;PIK3C3;SIK2;ZNF510;CALCRL;CTTNBP2;RNF180;CRMP1;SNTG1;MAP2;NCAM1;MPDZ;CLASP2;KIDINS220;MYO9A;QKI;CCDC144NL;NBEA;APC;DCHS2;TTC3;ST7;FAT3;TCF4;CNTN3*
PRICKLE2-AS1	50/100	1.4530475903009115E-5	*SYNM;TGFB1I1;ITSN1;STON1;MPRIP;DIP2C;SBF2;TEAD1;PRKG1;TNS1;RBFOX2;AFAP1;ABCC9;STON1-GTF2A1L;EML1;KATNAL1;SETBP1;PALLD;PEAK1;KCNMA1;WDFY3;TTLL11;PPP1R12B;PDZRN3;VCL;RABGAP1;SYNPO2;LAMA4;PRUNE2;PRICKLE2;CACNA1C;LPP;TOR1AIP1;ATXN1;DPP8;CALD1;MYH11;FLNC;MPDZ;STAR*

			D13;NEGR1;KIDINS220;NDE1;FOXN3;FER;APC;CCSER2;MSRB3;MRVI1;FERMT2
OPCML-IT1	50/100	1.4530475903009115E-5	*PCDHGB7;PCDHGB6;MEGF11;PCDHGB4;PCDHGB3;PCDHGB2;GRIK3;GRIK4;SLC35F1;GRIK2;LRRTM3;TMEM108;TNR;SOX8;EPHB1;PCDHGA8;PCDHGA7;GRID2;PCDHGA6;PCDHGA5;DSCAM;PCDHGA4;PCDHGA3;PCDHGA2;TCF12;PCDHGA1;PCDHGA9;BCAN;PCDHGB1;DSCAML1;CRB1;PCDH15;CRMP1;MAP2;FYN;NCAM1;GRIA3;GRIA4;OPCML;CA10;CADM2;TMEM132B;LSAMP;LHFPL3;PCDHGA10;ATAT1;PCDHGA11;PCDHGA12;NOVA1;APBA2*
LINC02283	49/100	3.357256471404773E-5	*PCDHGB7;PCDHGB6;PCDHGB4;MEGF11;PCDHGB3;PCDHGB2;GRIK4;GRIK2;TRIM9;LRRTM3;TNR;SOX8;DENND5A;EPHB1;PCDHGA8;PCDHGA7;PCDHGA6;GRID2;PCDHGA5;DSCAM;PCDHGA4;PCDHGA3;PCDHGA2;TCF12;PCDHGA1;PCDHGA9;BCAN;CDH10;PCDHGB1;DSCAML1;CRB1;PCDH15;CRMP1;MAP2;NCAM1;FYN;GRIA3;GRIA4;CLASP2;BTBD17;CADM2;LSAMP;LHFPL3;QKI;PCDHGA10;ATAT1;PCDHGA11;PCDHGA12;NOVA1*
LINC02060	49/100	3.357256471404773E-5	*PCDHGB7;PCDHGB6;PCDHGB4;CTNND2;PCDHGB3;PCDHGB2;GRIK4;FMN2;IQCJ-SCHIP1;TRIM9;DENND5A;PCDHGA8;PCDHGA7;PCDHGA6;PCDHGA5;DSCAM;PCDHGA4;PCDHGA3;PCDHGA2;PCDHGA1;PCDHGA9;BCAN;RFX4;CDH10;PCDHGB1;ADCYAP1R1;LUZP2;PCDH15;SLC1A3;ADCY2;NDRG2;SNN;MAP2;NCAM1;KCNN3;GRIA3;JAM2;CLASP2;BBS2;DTNA;CADM2;LSAMP;QKI;PCDHGA10;PCDHGA11;PCDHGA12;APC;NOVA1;APBA2*
LINC02318	49/100	3.357256471404773E-5	*PCDHGB7;PCDHGB6;PCDHGB4;CTNND2;PCDHGB3;PCDHGB2;GRIK4;GRIK2;FMN2;TRIM9;DPYSL2;PDE4B;BAALC;DENND5A;EPHB1;SH3GL2;WLS;PCDHGA8;PCDHGA7;PCDHGA6;PCDHGA5;PCDHGA4;PCDHGA3;PCDHGA2;PCDHGA1;PCDHGA9;BCAN;FRMD5;RFX4;PCDHGB1;GRIA1;CRB1;CRMP1;LRIG1;NCAM1;FYN;LRRC4C;GRIA3;GPR158;JAM2;CLASP2;CADPS;QKI;PCDHGA10;ATAT1;PCDHGA11;PCDHGA12;NOVA1;APBA2*
LINC01602	48/100	5.969071848879689E-5	*CRB1;PCDHGB7;DGKB;FAM13C;PCDHGB4;PCDHGB2;GRIK4;GRIK2;DRP2;TRIM9;OPHN1;FAM184B;MAP2;FAM110B;PHACTR3;TNR;SOX8;FYN;CSMD3;SOX6;LRRC4C;CACNG2;EPHB1;GRIA3;GPR158;SCN1A;GRIA4;PCDHGA6;CA10;PCDHGA5;DSCAM;PCDHGA4;PCDHGA3;TMEM132B;PCDHGA2;TCF12;PCDHGA1;LHFPL3;MYO16;PCDHGA10;PCDHGA11;PCDHGA12;FCHSD2;PCDHGB1;TOX;MDGA2;FGF12;DSCAML1*
LINC00689	48/100	5.969071848879689E-5	*CRB1;DGKB;FAM13C;CTTNBP2;MEGF11;LUZP2;PCDH15;GRIK4;GRIK2;TMEM100;DRP2;TRIM9;OPHN1;MAP2;FAM110B;PHACTR3;LRIG1;TNR;GSG1L;SOX8;DENND5A;NCAM1;FYN;CSMD3;SOX6;RHBDL3;EPHB1;GRIA3;SCN1A;GRIA4;CLASP2;GRID2;DSCAM;CADM2;TCF12;LSAMP;LHFPL3;QKI;MYO16;PCDHGA1*

			0;ATAT1;BCAN;PCDHGA12;FCHSD2;NOVA1;CDH10;FGF12;DSCAML1
LINC00391	48/100	5.969071848879689E-5	*PCDHGB7;PCDHGB6;PCDHGB4;CTNND2;PCDHGB3;NRXN1;PCDHGB2;SLC1A3;GRIK4;ADCY2;FMN2;NDRG2;TRIM9;SNN;LRRTM3;MAP2;BAALC;DENND5A;NCAM1;GRIA3;JAM2;GRIA4;CLASP2;PCDHGA8;PCDHGA7;PCDHGA6;DTNA;PCDHGA5;DSCAM;PCDHGA4;CADM2;PCDHGA3;PCDHGA2;NTRK3;PCDHGA1;LSAMP;GPR75;QKI;PCDHGA9;PCDHGA10;PCDHGA11;PCDHGA12;APC;RFX4;CDH10;PCDHGB1;APBA2;ASTN1*
LINC02226	48/100	5.969071848879689E-5	*PCDHGB7;NLGN1;KHDRBS3;PCDHGB6;PCDHGB4;CTNND2;PCDHGB3;PCDHGB2;GRIK4;SLC35F1;AKAP6;FMN2;TRIM9;EDNRB;UBL3;TSPAN7;BAALC;DENND5A;FYN;NCAM1;SOX6;CLASP2;PCDHGA8;PCDHGA7;ARNT2;PCDHGA6;PCDHGA5;MYEF2;PCDHGA4;ST8SIA1;PCDHGA3;PCDHGA2;MAGI2;PCDHGA1;LSAMP;CORO2B;QKI;PCDHGA9;IGSF11;PCDHGA10;BCAN;PCDHGA11;PCDHGA12;PTPRA;PCDHGB1;MDGA2;APBA2;ASTN1*
LINC01415	48/100	5.969071848879689E-5	*PCDHGB7;SHC3;PCDHGB6;PCDHGB4;PCDHGB3;NRXN1;PCDHGB2;GRIK4;CRMP1;GRIK2;TRIM9;LRRTM3;SNTG1;MAP2;BAALC;TNR;DENND5A;NCAM1;LRRC4C;CACNG2;GRIA3;GRIA4;CLASP2;PCDHGA8;PCDHGA7;PCDHGA6;GRID2;PCDHGA5;DSCAM;PCDHGA4;CADM2;PCDHGA3;PCDHGA2;PCDHGA1;TCF12;LSAMP;LHFPL3;SEZ6L;QKI;PCDHGA9;PCDHGA10;PCDHGA11;PCDHGA12;APC;CDH10;PCDHGB1;TCF4;ASTN1*
NRXN2-AS1	48/100	5.969071848879689E-5	*PCDHGB7;PCDHGB6;PCDHGB4;CTNND2;PCDHGB3;NRXN1;PCDHGB2;GRIK4;CRMP1;FMN2;GRIK2;TRIM9;LRRTM3;MAP2;BAALC;DENND5A;NCAM1;FYN;LRRC4C;GRIA3;GRIA4;CLASP2;PCDHGA8;PCDHGA7;PCDHGA6;GRID2;PCDHGA5;DSCAM;PCDHGA4;CADM2;PCDHGA3;PCDHGA2;NTRK3;PCDHGA1;LSAMP;LHFPL3;QKI;PCDHGA9;PCDHGA10;GNAO1;ATAT1;BCAN;PCDHGA11;PCDHGA12;CDH10;PCDHGB1;APBA2;ASTN1*
NKAIN3-IT1	48/100	5.969071848879689E-5	*ADCYAP1R1;PCDHGB7;PCDHGB4;LUZP2;CTNND2;PCDHGB3;NRXN1;SLC1A3;GRIK4;ADCY2;FMN2;NDRG2;TRIM9;SNN;NKAIN3;OPHN1;DENND5A;NCAM1;KCNN3;GRIA3;JAM2;GRIA4;CLASP2;PCDHGA8;PCDHGA7;NTRK2;PCDHGA6;DTNA;PCDHGA5;KCND2;PCDHGA4;CADM2;PCDHGA3;PCDHGA2;PCDHGA1;LSAMP;GPR75;QKI;GNAO1;PCDHGA11;APC;DAAM2;RFX4;CDH10;PCDHGB1;SLC25A53;APBA2;ASTN1*
JAKMIP2-AS1	48/100	5.969071848879689E-5	*PCDHGB7;PCDHGB6;PCDHGB4;CTNND2;PCDHGB3;NRXN1;PCDHGB2;GRIK4;CRMP1;FMN2;TRIM9;DPP6;LRRTM3;MAP2;TSPAN7;BAALC;DENND5A;NCAM1;LRRC4C;GRIA3;GRIA4;CLASP2;PCDHGA8;PCDHGA7;PCDHGA6;GRID2;DTNA;PCDHGA5;DSCAM;PCDHGA4;CADM2;PCDHGA3;PCDHGA2;PCDHGA1;LSAMP;GPR75;*

			QKI;PCDHGA9;GNAO1;PCDHGA10;PCDHGA11;PCDHGA12;APC;RFX4;CDH10;PCDHGB1;APBA2;ASTN1
LINC01690	47/100	1.2153385460900866E-4	*CRB1;ADCYAP1R1;PCDHGB7;PCDHGB6;PCDHGB4;LUZP2;CTNND2;PCDHGB3;PCDHGB2;PCDH15;GRIK4;NDRG2;TRIM9;OPHN1;LRRTM3;MAP2;DENND5A;SOX8;NCAM1;FYN;KCNN3;GRIA3;GRIA4;CLASP2;PCDHGA8;PCDHGA7;PCDHGA6;GRID2;PCDHGA5;DSCAM;PCDHGA4;CADM2;PCDHGA3;PCDHGA2;PCDHGA1;TCF12;LSAMP;QKI;PCDHGA9;PCDHGA10;BCAN;PCDHGA11;PCDHGA12;FCHSD2;CDH10;PCDHGB1;DSCAML1*
LINC00461	47/100	1.2153385460900866E-4	*CRB1;ADCYAP1R1;PCDHGB7;PCDHGB6;PCDHGB4;CTNND2;PCDHGB3;NRXN1;PCDHGB2;SLC1A3;GRIK4;FMN2;TRIM9;SNN;LRRTM3;MAP2;BAALC;DENND5A;NCAM1;GRIA3;GRIA4;CLASP2;PCDHGA8;PCDHGA7;PCDHGA6;GRID2;PCDHGA5;DSCAM;PCDHGA4;CADM2;PCDHGA3;PCDHGA2;PCDHGA1;LSAMP;QKI;PCDHGA9;PCDHGA10;ATAT1;BCAN;PCDHGA11;PCDHGA12;APC;RFX4;CDH10;PCDHGB1;APBA2;ASTN1*
LINC02144	47/100	1.2153385460900866E-4	*PCDHGB7;RTN1;PCDHGB6;PCDHGB4;CTNND2;PCDHGB3;NRXN1;PCDHGB2;SLC1A3;CRMP1;SLC35F1;FMN2;TRIM9;SNN;LRRTM3;MAP2;SNTG1;TSPAN7;BAALC;NCAM1;GRIA3;CLASP2;PCDHGA8;PCDHGA7;GRID2;PCDHGA6;DTNA;PCDHGA5;PCDHGA4;CADM2;PCDHGA3;PCDHGA2;NTRK3;PCDHGA1;LSAMP;QKI;PCDHGA9;PCDHGA10;ATAT1;PCDHGA11;PCDHGA12;APC;NOVA1;CDH10;PCDHGB1;APBA2;ASTN1*
CTNNA2-AS1	47/100	1.2153385460900866E-4	*CRB1;PCDHGB7;PCDHGB6;PCDHGB4;CTNND2;PCDHGB3;NRXN1;PCDHGB2;SLC1A3;GRIK4;FMN2;NPAS3;TRIM9;DPP6;SNN;LRRTM3;MAP2;TSPAN7;BAALC;DENND5A;NCAM1;CTNNA2;GRIA3;JAM2;CLASP2;PCDHGA8;PCDHGA7;PCDHGA6;DTNA;PCDHGA5;PCDHGA4;CADM2;PCDHGA3;PCDHGA2;PCDHGA1;LSAMP;QKI;PCDHGA9;PCDHGA10;PCDHGA11;PCDHGA12;APC;RFX4;CDH10;PCDHGB1;APBA2;ASTN1*
TRAF3IP2-AS1	47/100	1.2153385460900866E-4	*PCDHGB7;PCDHGB6;PCDHGB4;CTNND2;PCDHGB3;PCDHGB2;GRIK4;CRMP1;TRIM9;LRRTM3;MAP2;BAALC;DENND5A;FYN;NCAM1;GRIA3;GRIA4;CLASP2;PCDHGA8;PCDHGA7;PCDHGA6;PCDHGA5;DSCAM;PCDHGA4;CADM2;PCDHGA3;PCDHGA2;PCDHGA1;TCF12;LSAMP;GPR75;ZDHHC17;QKI;PCDHGA9;PCDHGA10;ATAT1;BCAN;CCDC88A;PCDHGA11;PCDHGA12;FCHSD2;APC;CDH10;PCDHGB1;APBA2;ASB3;ASTN1*
SLC6A1-AS1	47/100	1.2153385460900866E-4	*CRB1;PCDHGB7;PCDHGB6;PCDHGB4;MEGF11;PCDHGB3;NRXN1;PCDHGB2;GRIK4;ABAT;GRIK2;NDRG2;TRIM9;LRRTM3;MAP2;TNR;DENND5A;SOX8;NCAM1;GRIA3;GRIA4;CLASP2;PCDHGA8;PCDHGA7;PCDHGA6;PCDHGA5;DSCAM;PCDHGA4;CADM2;PCDHGA3;PCDHGA2;NTRK3;PCDHGA1;TCF12;LSAMP;GPR75;LHFP*

			L3;QKI;PCDHGA9;PCDHGA10;PCDHGA11;PCDHGA12;APC;CDH10;PCDHGB1;DSCAML1;ASTN1
LRP4-AS1	47/100	1.2153385460900866E-4	*PCDHGB7;SHC3;DOCK3;PCDHGB6;MEGF11;PCDHGB4;PCDHGB3;NRXN1;PCDHGB2;GRIK4;LRP4;GRIK2;TRIM9;LRRTM3;MAP2;TNR;SOX8;FYN;NCAM1;LRRC4C;CACNG2;GRIA3;GRIA4;PCDHGA8;PCDHGA7;PCDHGA6;PCDHGA5;DSCAM;PCDHGA4;CADM2;PCDHGA3;TMEM132B;PCDHGA2;NTRK3;PCDHGA1;TCF12;GPR75;LHFPL3;PCDHGA9;PCDHGA10;BCAN;PCDHGA11;PCDHGA12;CDH10;PCDHGB1;DSCAML1;ASTN1*
ADGRL3-AS1	47/100	1.2153385460900866E-4	*CRB1;PCDHGB7;PCDHGB6;PCDHGB4;CTNND2;PCDHGB3;PCDHGB2;GRIK4;CRMP1;TRIM9;LRRTM3;MAP2;TSPAN7;BAALC;DENND5A;SOX8;NCAM1;GRIA3;GRIA4;CLASP2;PCDHGA8;PCDHGA7;PCDHGA6;GRID2;PCDHGA5;DSCAM;PCDHGA4;CADM2;PCDHGA3;PCDHGA2;NTRK3;PCDHGA1;LSAMP;QKI;PCDHGA9;PCDHGA10;GNAO1;DNM3;ATAT1;BCAN;PCDHGA11;PCDHGA12;NOVA1;CDH10;PCDHGB1;APBA2;ASTN1*
LINC02774	46/100	3.110362831089401E-4	*PCDHGB7;SHC3;RTN1;PCDHGB6;PCDHGB4;MEGF11;PCDHGB3;NRXN1;PCDHGB2;GRIK4;CRMP1;SLC35F1;GRIK2;DRP2;TRIM9;LRRTM3;MAP2;SNTG1;TNR;SOX8;NCAM1;GRIA3;GRIA4;OPCML;CLASP2;PCDHGA8;PCDHGA7;PCDHGA6;PCDHGA5;DSCAM;PCDHGA4;PCDHGA3;TMEM132B;PCDHGA2;PCDHGA1;LHFPL3;PCDHGA9;PCDHGA10;ATAT1;BCAN;PCDHGA11;PCDHGA12;CDH10;PCDHGB1;DSCAML1;APBA2*
DSCAM-IT1	46/100	3.110362831089401E-4	*GRIA2;CRB1;PCDHGB7;PCDHGB6;PCDHGB4;LUZP2;PCDHGB3;PCDHGB2;PCDH15;GRIK4;GRIK2;MAP2;SOX8;NCAM1;FYN;SOX6;CSMD2;EPHB1;GRIA3;SCN1A;GRIA4;CLASP2;PCDHGA8;PCDHGA7;GRID2;PCDHGA6;PCDHGA5;DSCAM;PCDHGA4;CADM2;PCDHGA3;PCDHGA2;TCF12;PCDHGA1;LSAMP;LHFPL3;PCDHGA9;PCDHGA10;ATAT1;BCAN;PCDHGA11;PCDHGA12;NOVA1;PCDHGB1;DSCAML1;APBA2*
LINC00237	45/100	5.860319946433883E-4	*CRB1;PCDHGB7;PCDHGB6;PCDHGB4;MEGF11;PCDHGB3;PCDHGB2;GRIK4;CRMP1;GRIK2;TRIM9;LRRTM3;MAP2;SNTG1;TNR;BAALC;SOX8;NCAM1;GRIA3;GRIA4;CLASP2;PCDHGA8;PCDHGA7;PCDHGA6;GRID2;PCDHGA5;DSCAM;PCDHGA4;CADM2;PCDHGA3;PCDHGA2;PCDHGA1;TCF12;LHFPL3;PCDHGA9;PCDHGA10;ATAT1;BCAN;PCDHGA11;PCDHGA12;NOVA1;CDH10;PCDHGB1;DSCAML1;APBA2*
LINC01074	45/100	5.860319946433883E-4	*CRB1;ADCYAP1R1;PCDHGB7;PCDHGB6;PCDHGB4;CTNND2;PCDHGB3;PCDHGB2;SLC1A3;GRIK4;ADCY2;FMN2;KCNA6;NDRG2;PHACTR3;TNR;DENND5A;NCAM1;CTNNA2;JAM2;CLASP2;PCDHGA8;PCDHGA7;NTRK2;PCDHGA6;DTNA;PCDHGA5;DSCAM;PCDHGA4;PCDHGA3;PCDHGA2;PCDHGA1;LSAMP;GPR75;QKI;GABRG1;PCDHGA9;PCDHGA10;BCAN;PCDHGA11;PC*

			DHGA12;RFX4;PCDHGB1;SLC25A53;ASTN1
LINC01546	45/100	5.860319946433883E-4	*PCDHGB7;PCDHGB6;MEGF11;PCDHGB4;PCDHGB3;PCDHGB2;GRIK4;SLC35F1;GRIK2;TRIM9;LRRTM3;SNTG1;MAP2;TNR;BAALC;SOX8;NCAM1;LRRC4C;CACNG2;GRIA3;GRIA4;PCDHGA8;PCDHGA7;PCDHGA6;GRID2;PCDHGA5;DSCAM;WSCD1;PCDHGA4;PCDHGA3;TMEM132B;PCDHGA2;PCDHGA1;LHFPL3;SEZ6L;PCDHGA9;PCDHGA10;ATAT1;BCAN;PCDHGA11;PCDHGA12;CDH10;PCDHGB1;DSCAML1;ASTN1*
LINC01102	45/100	5.860319946433883E-4	*PCDHGB7;PCDHGB6;PCDHGB4;PCDHGB3;PCDHGB2;GRIK4;CRMP1;GRIK2;DRP2;TRIM9;DPP6;LRRTM3;MAP2;SNTG1;TSPAN7;SOX8;NCAM1;LRRC4C;CACNG2;GRIA3;GRIA4;CLASP2;PCDHGA8;PCDHGA7;PCDHGA6;PCDHGA5;DSCAM;PCDHGA4;CADM2;PCDHGA3;PCDHGA2;PCDHGA1;LHFPL3;SEZ6L;PCDHGA9;PCDHGA10;GNAO1;ATAT1;PCDHGA11;PCDHGA12;CDH10;PCDHGB1;DSCAML1;UNC79;ASTN1*
LINC02588	45/100	5.860319946433883E-4	*CRB1;PCDHGB7;PCDHGB6;PCDHGB4;CTNND2;PCDHGB3;PCDHGB2;PCDH15;GRIK4;CRMP1;SLC35F1;IQCJ-SCHIP1;TRIM9;LRRTM3;SNTG1;MAP2;BAALC;SOX8;NCAM1;GRIA3;PCDHGA8;PCDHGA7;GRID2;PCDHGA6;PCDHGA5;DSCAM;PCDHGA4;KCND2;PCDHGA3;PCDHGA2;PCDHGA1;TCF12;LHFPL3;QKI;PCDHGA9;PCDHGA10;ATAT1;BCAN;PCDHGA11;PCDHGA12;NOVA1;CDH10;PCDHGB1;MKRN3;APBA2*
ANKRD62P1-PARP4P3	45/100	5.860319946433883E-4	*GRIA2;CRB1;ADCYAP1R1;LUZP2;CTNND2;PCDH15;GRIK3;GRIK4;NDRG2;ARHGAP35;OPHN1;TRPS1;FAM110B;LRIG1;SOX8;DENND5A;DLGAP1;NCAM1;KCNN3;CSMD2;GRIA3;SCN1A;GRIA4;CLASP2;NTRK2;DSCAM;KCND2;CADM2;TCF12;OSGIN2;IQCJ;QKI;DNM3;BCAN;RAB30;FRMD5;FCHSD2;NFIA;DAAM2;NOVA1;TTC3;MKRN3;ZMYND11;DSCAML1;ARHGEF6*
NCAM1-AS1	45/100	5.860319946433883E-4	*PCDHGB7;PCDHGB6;PCDHGB4;CTNND2;PCDHGB3;NRXN1;PCDHGB2;GRIK4;CRMP1;FMN2;GRIK2;SCAMP5;TRIM9;DPP6;LRRTM3;MAP2;NCAM1;LRRC4C;GRIA3;GPR158;GRIA4;CLASP2;PCDHGA8;PCDHGA7;PCDHGA6;GRID2;PCDHGA5;DSCAM;PCDHGA4;CADM2;PCDHGA3;PCDHGA2;NTRK3;PCDHGA1;LSAMP;PCDHGA9;GNAO1;PCDHGA10;DNM3;PCDHGA11;PCDHGA12;CDH10;PCDHGB1;UNC79;ASTN1*
GSN-AS1	45/100	5.860319946433883E-4	*MACF1;ANKRD36;KMT2C;GPR21;LAMA4;DOCK7;TULP4;FMN1;ASAP1;GPATCH2L;SPATA5;LPP;DOCK10;AKAP13;ATXN1;SGCD;CHST11;ZNF407;TIMP2;POTEE;ABL2;EPG5;POTEF;HMCN1;ERC1;EVI5;TEAD1;SRGAP2B;FARP2;MAP3K3;RBFOX2;SORT1;ANKRD44;SAMD4A;MITF;GANC;TANC1;FER;KATNAL1;PEAK1;CDC42EP3;PMP22;MSRB3;VCL;TNRC6B*
CPB2-AS1	45/100	5.860319946433883E-4	*CRB1;PCDHGB7;PCDHGB6;PCDHGB4;PCDHGB3;NRXN1;PCDHGB2;GRIK4;ABAT;GRIK2;TRIM9;LRRTM3;MAP2;TSPAN7;TNR;BAALC;DENND5A;SOX8;NCAM1;GRIA3;GRIA4;CLASP2*

			;PCDHGA8;PCDHGA7;ARNT2;PCDHGA6;PCDHGA5;DSCAM;PCDHGA4;CADM2;PCDHGA3;PCDHGA2;PCDHGA1;LSAMP;LHFPL3;PCDHGA9;PCDHGA10;GNAO1;IGSF11;ATAT1;PCDHGA11;PCDHGA12;CDH10;PCDHGB1;ASTN1
NMBR-AS1	45/100	5.860319946433883E-4	*FTO;TPH2;ATP8A2;MAST4;KCNE4;GRIK1;LRP2;FRY;ABCC11;RERG;LRRC53;PHF21B;DACH1;GRM4;ERBB4;KIAA1324;SUSD4;MALRD1;PRKACB;EDIL3;UNC13A;PCDH9;NEGR1;SIAH3;TPRG1;SEMA6D;MAGI2;TNNI3K;MANEAL;ESR1;GDAP1;GABRG2;DCDC1;DCLK1;MED13L;CACNB2;XKR7;SLC5A8;PEX5L;FGF14;SLCO3A1;KCNS3;PI15;CCDC170;FGF12*
CXXC4-AS1	45/100	5.860319946433883E-4	*GRIA2;DGKB;CTNND2;NRXN1;PCDH15;GRIK4;CRMP1;ABAT;GRIK2;SCAMP5;DPP6;OPHN1;LRRTM3;MAP2;TNR;DENND5A;NCAM1;NCAM2;LRRC4C;CACNG2;GRIA3;GPR158;TRIM23;GRIA4;CLASP2;GRID2;DSCAM;KCND2;CADM2;KIDINS220;TCF12;LSAMP;LHFPL3;SEZ6L;QKI;GNAO1;DNM3;BCAN;APC;NOVA1;CDH10;CNTN1;DSCAML1;UNC79;ASTN1*
NNT-AS1	45/100	5.860319946433883E-4	*PCDHGB7;PCDHGB6;FAM13C;PCDHGB4;PCDHGB3;PCDHGB2;GRIK4;FMN2;TRIM9;LRRTM3;MAP2;TSPAN7;BAALC;DENND5A;NCAM1;SOX6;GRIA3;TRIM23;GRIA4;CLASP2;BBS2;PCDHGA8;PCDHGA7;ARNT2;PCDHGA6;PCDHGA5;ZHX3;PCDHGA4;CADM2;PCDHGA3;PCDHGA2;MAGI2;PCDHGA1;LSAMP;FBXL17;PRKCA;QKI;PCDHGA9;PCDHGA10;PCDHGA11;PCDHGA12;APC;PCDHGB1;MAPRE2;ASTN1*
LINC01351	44/100	0.0012010527864875081	*PCDHGB7;PCDHGB6;PCDHGB4;CTNND2;PCDHGB3;NRXN1;PCDHGB2;GRIK4;SLC35F1;FMN2;TRIM9;LRRTM3;MAP2;BAALC;DENND5A;NCAM1;GRIA3;GRIA4;CLASP2;PCDHGA8;PCDHGA7;PCDHGA6;GRID2;PCDHGA5;DSCAM;PCDHGA4;CADM2;PCDHGA3;PCDHGA2;NTRK3;PCDHGA1;LSAMP;LHFPL3;QKI;PCDHGA9;PCDHGA10;ATAT1;BCAN;PCDHGA11;PCDHGA12;CDH10;PCDHGB1;APBA2;ASTN1*
LINC02234	44/100	0.0012010527864875081	*PCDHGB7;KHDRBS3;PCDHGB6;PCDHGB4;PCDHGB3;PCDHGB2;SLC35F1;AKAP6;GRIK2;TRIM9;DPP6;LRRTM3;SNTG1;MAP2;TSPAN7;BAALC;DENND5A;FYN;NCAM1;LRRC4C;GRIA3;JAM2;CLASP2;PCDHGA8;PCDHGA7;PCDHGA6;PCDHGA5;PCDHGA4;PCDHGA3;PCDHGA2;PCDHGA1;LSAMP;LHFPL3;RGMB;CORO2B;QKI;PCDHGA9;PCDHGA10;PCDHGA11;PCDHGA12;NOVA1;CDH10;PCDHGB1;ASTN1*
LINC01963	44/100	0.0012010527864875081	*PCDHGB7;PCDHGB6;PCDHGB4;CTNND2;PCDHGB3;NRXN1;PCDHGB2;GRIK4;CRMP1;FMN2;TRIM9;DPP6;LRRTM3;MAP2;DPYSL2;TSPAN7;NCAM1;FYN;GRIA3;GRIA4;CLASP2;PCDHGA8;PCDHGA7;PCDHGA6;PCDHGA5;PCDHGA4;CADM2;PCDHGA3;PCDHGA2;NTRK3;PCDHGA1;LSAMP;QKI;PCDHGA9;PCDHGA10;GNAO1;DNM3;PCDHGA11;PCDHGA12;APC;CDH10;PCDHGB1;APBA2;ASTN1*
LINC00906	44/100	0.0012010527864875081	*CRB1;ADCYAP1R1;PCDHGB7;PCDHGB4;LUZP2;CTNND2;PCDHGB3;PCDHGB2;PCDH15;GRI*

			K4;ADCY2;FMN2;NDRG2;ADCY8;SNN;OPHN1;DENND5A;NCAM1;KCNN3;SOX6;EPHB1;GRIA3;JAM2;GRIA4;PCDHGA8;PCDHGA7;PCDHGA6;PCDHGA5;BTBD17;PCDHGA4;CADM2;PCDHGA3;PCDHGA1;LSAMP;PDE4DIP;QKI;PCDHGA10;BCAN;PCDHGA11;FRMD5;PCDHGA12;DAAM2;PCDHGB1;SLC25A53
EDNRB-AS1	44/100	0.0012010527864875081	*MIPEP;PCDHGB7;CHRM1;PCDHGB6;CAB39L;PCDHGB4;LUZP2;CTNND2;PCDHGB3;PCDHGB2;ADCY2;AKAP6;VPS26B;FMN2;DACH2;SLC4A4;EDNRB;RIC8B;OPHN1;BAALC;TRIM23;CLASP2;BBS2;PCDHGA8;PCDHGA7;ARNT2;PCDHGA6;PCDHGA5;PCDHGA4;PCDHGA3;PCDHGA2;PCDHGA1;LSAMP;QKI;PCDHGA9;IGSF11;PCDHGA10;PCDHGA11;PCDHGA12;DPY19L2;DAAM2;CDH10;PCDHGB1;CPE*
ASIC4-AS1	44/100	0.0012010527864875081	*PCDHGB7;PCDHGB6;MEGF11;PCDHGB4;PCDHGB3;PCDHGB2;GRIK4;GRIK2;DRP2;TRIM9;LRRTM3;MAP2;SNTG1;FAM110B;TNR;SOX8;NCAM1;CACNG2;GRIA3;GRIA4;CLASP2;PCDHGA8;PCDHGA7;PCDHGA6;PCDHGA5;DSCAM;PCDHGA4;CADM2;PCDHGA3;PCDHGA2;PCDHGA1;TCF12;LHFPL3;SEZ6L;PCDHGA9;PCDHGA10;ATAT1;BCAN;PCDHGA11;PCDHGA12;CDH10;PCDHGB1;DSCAML1;UNC79*
SOX21-AS1	44/100	0.0012010527864875081	*CRB1;PCDHGB7;PCDHGB6;PCDHGB4;LUZP2;CTNND2;PCDHGB3;PCDHGB2;GRIK4;FMN2;NDRG2;TRIM9;SNN;LRRTM3;MAP2;BAALC;SOX8;NCAM1;GRIA3;GRIA4;PCDHGA8;PCDHGA7;NTRK2;PCDHGA6;PCDHGA5;DSCAM;PCDHGA4;CADM2;PCDHGA3;PCDHGA2;PCDHGA1;TCF12;LSAMP;QKI;PCDHGA9;PCDHGA10;BCAN;PCDHGA11;PCDHGA12;RFX4;CDH10;PCDHGB1;APBA2;ASTN1*
SEPTIN7-AS1	44/100	0.0012010527864875081	*ADCYAP1R1;PCDHGB7;PCDHGB6;PCDHGB4;CTNND2;PCDHGB3;PCDHGB2;GRIK4;ADCY2;FMN2;NDRG2;TRIM9;SNN;BAALC;DENND5A;NCAM1;KCNN3;JAM2;CLASP2;BBS2;PCDHGA8;PCDHGA7;PCDHGA6;DTNA;ZHX3;PCDHGA5;PCDHGA4;PCDHGA3;PCDHGA2;MAGI2;PCDHGA1;GPR75;ZDHHC17;QKI;PCDHGA9;PCDHGA10;PCDHGA11;PCDHGA12;APC;TBC1D5;RFX4;PCDHGB1;SLC25A53;ARHGEF6*
GPR158-AS1	44/100	0.0012010527864875081	*PCDHGB7;PCDHGB6;PCDHGB4;PCDHGB3;PCDHGB2;GRIK4;GRIK2;FMN2;SCAMP5;JPH4;TRIM9;DPP6;LRRTM3;MAP2;TNR;NCAM1;LRRC4C;CACNG2;GRIA3;GPR158;GRIA4;CLASP2;PCDHGA8;PCDHGA7;PCDHGA6;UNC13A;PCDHGA5;PCDHGA4;CADM2;PCDHGA3;PCDHGA2;PCDHGA1;LSAMP;PCDHGA9;GNAO1;PCDHGA10;PCDHGA11;FAM155A;PCDHGA12;CDH10;REEP2;PCDHGB1;UNC79;ASTN1*
MIR9-3HG	44/100	0.0012010527864875081	*CRB1;PCDHGB7;PCDHGB6;PCDHGB4;CTNND2;PCDHGB3;PCDHGB2;PCDH15;GRIK4;GRIK2;NDRG2;TRIM9;OPHN1;LRRTM3;MAP2;SOX8;DENND5A;NCAM1;EPHB1;GRIA3;GRIA4;CLASP2;PCDHGA8;PCDHGA7;PCDHGA6;PCDHGA5;DSCAM;PCDHGA4;CADM2;PCDHGA3;PCDHGA2;TCF12;PCDHGA1;QKI;PCDHGA10;ATAT1*

			;BCAN;PCDHGA11;PCDHGA12;FCHSD2;CDH10;PCDHGB1;DSCAML1;APBA2
LINC02293	43/100	0.002722181580630758	*PCDHGB7;PCDHGB6;PCDHGB4;CTNND2;PCDHGB3;PCDHGB2;PCDH15;GRIK4;CRMP1;TRIM9;MAP2;SOX8;NCAM1;FYN;CSMD2;GRIA3;SCN1A;GRIA4;CLASP2;PCDHGA8;PCDHGA7;PCDHGA6;GRID2;PCDHGA5;DSCAM;KCND2;PCDHGA4;PCDHGA3;PCDHGA2;PCDHGA1;TCF12;QKI;PCDHGA9;PCDHGA10;ATAT1;BCAN;PCDHGA11;PCDHGA12;NOVA1;PCDHGB1;MKRN3;DSCAML1;APBA2*
GRID1-AS1	43/100	0.002722181580630758	*FAM49A;PCDHGB7;DOCK3;PCDHGB6;ATP8A1;PCDHGB4;PCDHGB3;GRIK4;SCAMP5;DPP6;MAP2;NCAM1;FYN;LRRC4C;SCAPER;TRIM23;CLASP2;ZNF540;PCDHGA8;TUB;PCDHGA7;PCDHGA6;PCDHGA5;PCDHGA4;CADM2;GRID1;PCDHGA3;KIDINS220;PCDHGA2;PCDHGA1;PCDHGA9;GNAO1;PCDHGA10;DNM3;MGAT4C;ARMCX4;PCDHGA11;PCDHGA12;PCDHGB1;DSCAML1;UNC79;ASTN1;CDS2*
CCND2-AS1	43/100	0.002722181580630758	*PCDHGB7;PCDHGB6;PCDHGB4;CTNND2;PCDHGB3;PCDHGB2;GRIK4;CRMP1;FMN2;KCNA6;TRIM9;DPP6;LRRTM3;MAP2;SOX8;NCAM1;FYN;LRRC4C;GRIA3;GRIA4;CLASP2;BBS2;PCDHGA8;PCDHGA7;PCDHGA6;PCDHGA5;DSCAM;PCDHGA4;CADM2;PCDHGA3;GRID1;PCDHGA2;NTRK3;PCDHGA1;PCDHGA9;PCDHGA10;GNAO1;PCDHGA11;PCDHGA12;IL1RAPL1;PCDHGB1;DSCAML1;ASTN1*
FOXG1-AS1	43/100	0.002722181580630758	*PCDHGB7;PCDHGB6;PCDHGB4;CTNND2;PCDHGB3;NRXN1;PCDHGB2;SLC1A3;GRIK4;SLC35F1;FMN2;IQCJ-SCHIP1;TRIM9;LRRTM3;SNTG1;MAP2;BAALC;DENND5A;GRIA3;PCDHGA8;PCDHGA7;PCDHGA6;DTNA;PCDHGA5;WSCD1;PCDHGA4;PCDHGA3;PCDHGA2;NTRK3;PCDHGA1;LHFPL3;QKI;PCDHGA9;PCDHGA10;ATAT1;PCDHGA11;PCDHGA12;NOVA1;RFX4;CDH10;PCDHGB1;APBA2;ASTN1*
LINC00652	42/100	0.004902856023861726	*CRB1;PCDHGB7;PCDHGB6;PCDHGB4;PCDHGB3;PCDHGB2;GRIK4;CRMP1;GRIK2;TRIM9;LRRTM3;MAP2;TSPAN7;BAALC;SOX8;NCAM1;EPHB1;GRIA3;GRIA4;PCDHGA8;PCDHGA7;PCDHGA6;GRID2;PCDHGA5;BTBD17;PCDHGA4;PCDHGA3;PCDHGA2;PCDHGA1;TCF12;LSAMP;QKI;PCDHGA9;PCDHGA10;ATAT1;BCAN;PCDHGA11;PCDHGA12;CDH10;PCDHGB1;APBA2;ASTN1*
LINC00994	42/100	0.004902856023861726	*ADCYAP1R1;PCDHGB7;PCDHGB6;PCDHGB4;LUZP2;CTNND2;PCDHGB3;NRXN1;PCDHGB2;GRIK4;ADCY2;FMN2;NDRG2;LRRTM3;DENND5A;SOX8;NCAM1;KCNN3;RHBDL3;GRIA4;CLASP2;PCDHGA8;PCDHGA7;PCDHGA6;PCDHGA5;DSCAM;PCDHGA4;CADM2;PCDHGA3;PCDHGA2;PCDHGA1;LSAMP;GPR75;QKI;PCDHGA9;PCDHGA10;GNAO1;PCDHGA11;PCDHGA12;DAAM2;CDH10;PCDHGB1*
LINC02731	42/100	0.004902856023861726	*PCDHGB7;PCDHGB6;PCDHGB4;PCDHGB3;PCDHGB2;GRIK4;CRMP1;GRIK2;DPP6;LRRTM3;*

			MAP2;SOX8;NCAM1;LRRC4C;CACNG2;GRIA3;GRIA4;CLASP2;PCDHGA8;PCDHGA7;PCDHGA6;PCDHGA5;DSCAM;PCDHGA4;CADM2;PCDHGA3;PCDHGA2;PCDHGA1;LSAMP;SEZ6L;PCDHGA9;PCDHGA10;GNAO1;ATAT1;PCDHGA11;FAM155A;PCDHGA12;REEP2;PCDHGB1;DSCAML1;UNC79;ASTN1
LINC01114	42/100	0.004902856023861726	*CRB1;PCDHGB7;PCDHGB6;PCDHGB4;CTNND2;PCDHGB3;PCDHGB2;SLC1A3;GRIK4;SLC35F1;FMN2;FGF1;IQCJ-SCHIP1;TRIM9;SNN;DPYSL2;BAALC;DPF3;DENND5A;CTNNA2;JAM2;PCDHGA8;PCDHGA7;PCDHGA6;DTNA;PCDHGA5;WSCD1;PCDHGA4;PCDHGA3;PCDHGA2;PCDHGA1;DENND2A;QKI;PCDHGA9;PCDHGA10;PCDHGA11;PCDHGA12;APC;RFX4;PCDHGB1;APBA2;ASTN1*
LINC01141	42/100	0.004902856023861726	*PCDHGB7;PCDHGB6;PCDHGB4;CTNND2;PCDHGB3;PCDHGB2;SLC1A3;GRIK4;ADCY2;FMN2;FGF1;NDRG2;IQCJ-SCHIP1;TRIM9;SNN;DPYSL2;BAALC;DENND5A;NCAM1;KCNN3;GRIA3;JAM2;PCDHGA8;PCDHGA7;PCDHGA6;DTNA;PCDHGA5;PCDHGA4;PCDHGA3;PCDHGA2;NTRK3;PCDHGA1;LSAMP;QKI;PCDHGA9;PCDHGA10;PCDHGA11;PCDHGA12;APC;RFX4;PCDHGB1;APBA2*
LINC02488	42/100	0.004902856023861726	*CRB1;PCDHGB4;CTNND2;PCDHGB3;PCDHGB2;PCDH15;GRIK3;CRMP1;IQCJ-SCHIP1;TRIM9;MAP2;SNTG1;SOX8;NCAM1;FYN;CSMD2;EPHB1;GRIA3;SCN1A;CLASP2;PCDHGA8;PCDHGA7;GRID2;PCDHGA6;PCDHGA5;DSCAM;WSCD1;KCND2;PCDHGA4;PCDHGA3;PCDHGA2;TCF12;PCDHGA1;LHFPL3;QKI;ATAT1;BCAN;NOVA1;CDH10;PCDHGB1;MKRN3;APBA2*
LINC01152	42/100	0.004902856023861726	*PCDHGB7;NLGN1;PCDHGB6;MAML2;PCDHGB4;CTNND2;PCDHGB3;PCDHGB2;DOCK7;GRIK4;TRIM9;MAP2;BAALC;DENND5A;NCAM1;ZNF501;JAM2;GRIA4;CLASP2;PCDHGA8;PCDHGA7;PCDHGA6;PCDHGA5;PCDHGA4;ST8SIA1;PCDHGA3;PCDHGA2;NTRK3;PCDHGA1;TCF12;QKI;PCDHGA9;PCDHGA10;ATAT1;BCAN;PCDHGA11;PCDHGA12;APC;PCDHGB1;WDPCP;ZNF730;ASTN1*
LINC02598	42/100	0.004902856023861726	*CRB1;LUZP2;CTNND2;PCDH15;GRIK4;CRMP1;ADCY2;GRIK2;NDRG2;IQCJ-SCHIP1;TRIM9;OPHN1;LRRTM3;MAP2;SNTG1;BAALC;SOX8;NCAM1;KCNN3;CSMD2;EPHB1;GRIA3;GRIA4;MARK1;CLASP2;NTRK2;GRID2;DSCAM;KCND2;TCF12;LSAMP;LHFPL3;QKI;ATAT1;BCAN;VANGL2;FCHSD2;NOVA1;CDH10;MKRN3;DSCAML1;APBA2*
LINC02552	42/100	0.004902856023861726	*CRB1;TRIM51;ITIH6;WDR63;XYLT1;FMN1;AKAP6;RLBP1;EDNRB;SGCD;UBL3;GNG2;CHST11;SCFD2;CHL1;LRRTM4;CHCHD6;HMCN1;STK32A;ANKS1A;ZC3H13;DISC1;SOX5;PKNOX2;FARP2;MCOLN3;MYEF2;EYA1;PCDH7;PAX3;MITF;NSG1;S100B;CORO2B;IGSF11;P2RX7;NELL1;NRG3;RHOJ;MDGA2;CNIH3;SLC24A5*

LINC02282	42/100	0.004902856023861726	*PCDHGB7;SHC3;PCDHGB6;PCDHGB4;PCDHGB3;NRXN1;PCDHGB2;SLC1A3;SLC35F1;GRIK2;TRIM9;LRRTM3;SNTG1;MAP2;BAALC;DENND5A;GRIA3;PCDHGA8;PCDHGA7;PCDHGA6;GRID2;PCDHGA5;DSCAM;WSCD1;PCDHGA4;PCDHGA3;TMEM132B;PCDHGA2;PCDHGA1;LSAMP;LHFPL3;SEZ6L;QKI;PCDHGA9;PCDHGA10;ATAT1;PCDHGA11;PCDHGA12;CDH10;PCDHGB1;APBA2;ASTN1*
LINC00609	42/100	0.004902856023861726	*CRB1;ADCYAP1R1;PCDHGB7;PCDHGB6;PCDHGB4;LUZP2;CTNND2;PCDHGB3;PCDH15;GRIK4;NDRG2;TRIM9;OPHN1;SOX8;DENND5A;NCAM1;FYN;KCNN3;GRIA3;GRIA4;CLASP2;PCDHGA8;PCDHGA7;PCDHGA6;PCDHGA5;DSCAM;PCDHGA4;CADM2;PCDHGA3;PCDHGA2;TCF12;PCDHGA1;QKI;PCDHGA10;ATAT1;BCAN;PCDHGA11;PCDHGA12;NOVA1;PCDHGB1;DSCAML1;APBA2*
LINC02339	42/100	0.004902856023861726	*PCDHGB7;SHC3;PCDHGB6;MEGF11;PCDHGB4;NRXN1;SLC35F1;GRIK2;DRP2;LRRTM3;SNTG1;MAP2;TNR;BAALC;SOX8;LRRC4C;CACNG2;GRIA3;GRIA4;PCDHGA8;GRID2;PCDHGA6;CA10;PCDHGA5;DSCAM;PCDHGA4;CADM2;PCDHGA3;TMEM132B;PCDHGA2;PCDHGA1;LHFPL3;SEZ6L;PCDHGA9;PCDHGA10;ATAT1;BCAN;PCDHGA12;CDH10;PCDHGB1;DSCAML1;UNC79*
ARHGEF7-AS1	42/100	0.004902856023861726	*MAML2;DENND5B;FAM13C;RNF180;NRXN1;ITSN1;CRMP1;FRY;NOL4;SCAMP5;CTIF;DPP6;OPHN1;MAP2;DENND5A;WDR7;NCAM1;DIP2C;SBF2;MPDZ;TRIM23;CLASP2;NCOA1;ZHX3;FBXW11;CADM2;KIDINS220;TCF12;EBF1;QKI;DNM3;SGSM1;ARMCX4;NBEA;SETBP1;APC;WDFY3;TCF4;SIK2;TLN2;ZNF510;CDS2*
NR2F1-AS1	42/100	0.004902856023861726	*PCDHGB7;PCDHGB6;PCDHGB4;CTNND2;PCDHGB3;NRXN1;PCDHGB2;SLC1A3;GRIK4;ADCY2;FMN2;NDRG2;SNN;DPYSL2;BAALC;DENND5A;FYN;NCAM1;GRIA3;JAM2;BBS2;PCDHGA8;PCDHGA7;PCDHGA6;DTNA;PCDHGA5;PCDHGA4;PCDHGA3;PCDHGA2;NTRK3;PCDHGA1;LSAMP;QKI;PCDHGA9;PCDHGA10;PCDHGA11;PCDHGA12;APC;RFX4;CDH10;PCDHGB1;APBA2*
LRRC7-AS1	42/100	0.004902856023861726	*ADCYAP1R1;GABRB1;RTN1;CTNND2;NRXN1;SLC1A2;OTUD7A;FMN2;NDRG2;SNN;GRM5;BRINP1;MAP2;CHN1;CREG2;DENND5A;DLGAP1;NCAM1;ERC2;SH3GL2;OPCML;ARNT2;DTNA;CADM2;NTRK3;LSAMP;GPR75;ANK2;SLC39A12;GRIN2B;GABRG1;GNAO1;CNKSR2;DLG2;APC;LRRC7;DNAJC6;DOK6;CDH10;RGS7BP;ASTN1;RAPGEF4*
ARHGEF26-AS1	42/100	0.004902856023861726	*ADCYAP1R1;PCDHGB7;PCDHGB6;PCDHGB4;LUZP2;CTNND2;PCDHGB3;NRXN1;PCDHGB2;SLC1A3;GRIK4;ADCY2;FMN2;NDRG2;SNN;BAALC;NCAM1;KCNN3;JAM2;PCDHGA8;PCDHGA7;NTRK2;PCDHGA6;DTNA;PCDHGA5;PCDHGA4;CADM2;PCDHGA3;PCDHGA2;PCDHGA1;LSAMP;GPR75;QKI;PCDHGA9;PCDHGA10;PCDHG*

			A11;PCDHGA12;RFX4;CDH10;PCDHGB1;CPE;ASTN1
BDNF-AS	42/100	0.004902856023861726	*PCDHGB7;PCDHGB6;FAM13C;PCDHGB4;CTNND2;PCDHGB3;PCDHGB2;GRIK4;ABAT;FMN2;NDRG2;SNN;TSPAN7;BAALC;DENND5A;NCAM1;GRIA3;JAM2;GRIA4;CLASP2;BBS2;PCDHGA8;PCDHGA7;PCDHGA6;DTNA;PCDHGA5;PCDHGA4;CADM2;PCDHGA3;PCDHGA2;MAGI2;PCDHGA1;LSAMP;QKI;PCDHGA9;PCDHGA10;GNAO1;PCDHGA11;PCDHGA12;CDH10;PCDHGB1;ASTN1*
LINC01561	41/100	0.009184611282291914	*PCDHGB7;PCDHGB6;PCDHGB4;LUZP2;CTNND2;PCDHGB3;PCDHGB2;PCDH15;GRIK4;ADCY2;FMN2;NDRG2;SNN;BAALC;DENND5A;SOX8;NCAM1;KCNN3;GRIA3;GRIA4;PCDHGA8;PCDHGA7;PCDHGA6;PCDHGA5;DSCAM;PCDHGA4;CADM2;PCDHGA3;PCDHGA2;NTRK3;PCDHGA1;LSAMP;QKI;PCDHGA9;PCDHGA10;GNAO1;PCDHGA11;PCDHGA12;PCDHGB1;APBA2;ASTN1*
LINC02549	41/100	0.009184611282291914	*PCDHGB7;PCDHGB6;PCDHGB4;CTNND2;RNF180;PCDHGB3;PCDHGB2;SLC1A3;FMN2;FGF1;IQCJ-SCHIP1;TRIM9;DPF3;BAALC;FYN;RGS6;JAM2;PCDHGA8;PCDHGA7;PCDHGA6;DTNA;PCDHGA5;WSCD1;PCDHGA4;PCDHGA3;PCDHGA2;MAGI2;DENND2A;PCDHGA1;CORO2B;QKI;PCDHGA9;PCDHGA10;PCDHGA11;PCDHGA12;IGDCC4;APC;PTPRA;RFX4;PCDHGB1;ASTN1*
LINC01066	41/100	0.009184611282291914	*PCDHGB6;PCDHGB4;CTNND2;PCDHGB3;PCDHGB2;SLC1A2;SLC1A3;FMN2;FGF1;SNN;MAP2;DPYSL2;BAALC;NCAM1;CTNNA2;JAM2;OR10K2;PCDHGA8;PCDHGA7;ARNT2;PCDHGA6;DTNA;PCDHGA5;PCDHGA4;PCDHGA3;PCDHGA2;MAGI2;DENND2A;PCDHGA1;LSAMP;SLC39A12;CORO2B;QKI;PCDHGA9;PCDHGA11;PCDHGA12;APC;RFX4;REEP2;APBA2;ASTN1*
LINC00437	41/100	0.009184611282291914	*PCDHGB7;PCDHGB6;PCDHGB4;CTNND2;RNF180;PCDHGB3;PCDHGB2;SLC1A2;SLC1A3;FMN2;FGF1;SPATA6;SNN;BAALC;CTNNA2;RGS6;JAM2;MAPK4;PCDHGA8;PCDHGA7;ARNT2;PCDHGA6;DTNA;PCDHGA5;PCDHGA4;PCDHGA3;PCDHGA2;MAGI2;DENND2A;ANK2;CORO2B;QKI;PCDHGA9;PCDHGA10;PCDHGA11;PCDHGA12;APC;PTPRA;RFX4;PCDHGB1;ASTN1*
LINC01741	41/100	0.009184611282291914	*CRB1;SHC3;DGKB;FAM13C;MEGF11;GRIK4;SLC35F1;GRIK2;DRP2;TRIM9;LRRTM3;FAM184B;MAP2;FAM110B;TNR;SOX8;CSMD3;SOX6;LRRC4C;CACNG2;EPHB1;DLGAP2;GRIA3;GPR158;TECTA;GRIA4;CA10;PCDHGA5;DSCAM;TMEM132B;TCF12;LHFPL3;MYO16;PCDHGA10;BCAN;PCDHGA12;FCHSD2;PCDHGB1;TOX;FGF12;DSCAML1*
LINC01563	41/100	0.009184611282291914	*CRB1;PCDHGB7;PCDHGB6;PCDHGB4;CTNND2;PCDHGB3;PCDHGB2;GRIK4;CRMP1;SLC35F1;GRIK2;FMN2;TRIM9;BAALC;DENND5A;NCAM1;FYN;GRIA4;CLASP2;PCDHGA8;PCDHGA7;PCDHGA6;PCDHGA5;PCDHGA4;PCDHGA3;PCDHGA2;PCDHGA1;LSAMP;CORO2B;QKI;PCD*

			HGA9;PCDHGA10;IGSF11;ATAT1;BCAN;PCDHGA11;PCDHGA12;RFX4;PCDHGB1;APBA2;ASTN1
LINC02008	41/100	0.009184611282291914	*ADCYAP1R1;NRXN1;GRIK4;CRMP1;GRIK2;FMN2;KCNA6;NPAS3;DRP2;TRIM9;CECR2;OPHN1;LRRTM3;MAP2;SNTG1;TNR;BAALC;SOX8;DENND5A;ZPLD1;NCAM1;CSMD3;CACNG2;GRIA3;GPR158;GRIA4;CLASP2;GRID2;DSCAM;CADM2;TCF12;PCDHGA1;GABRA3;LHFPL3;QKI;MYO16;PCDHGA10;BCAN;CDH10;DSCAML1;ASTN1*
LINC02720	41/100	0.009184611282291914	*PANK3;ZNF493;NVL;ZBTB20;ITPR2;LDLRAD4;FAM214A;CCDC144A;KIAA0040;TMEM241;ZBTB41;HS6ST3;KIAA1109;KIAA1328;ERBB4;TRPS1;SLC16A7;APBB2;THSD7A;ZNF720;FNIP1;RNF152;YAF2;RALGPS2;VAV3;UNC13C;MON2;LRBA;TBC1D9;PRLR;ESR1;PBX1;AR;ZNF91;RAB30;CCNG2;SIDT1;ZNF678;KIAA0825;DGKI;CNTNAP5*
ZNF32-AS3	41/100	0.009184611282291914	*GRIA2;SPIN3;NRXN1;GRIK4;DRP2;OPHN1;LRRTM3;MAP2;FAM110B;LRIG1;TNR;DENND5A;DLGAP1;NCAM1;SRGAP3;ZNF501;NCAM2;LRRC4C;EPHB1;GRIA3;TRIM23;GRIA4;CLASP2;GRID2;DSCAM;KCND2;CADM2;NTRK3;ZFP2;TCF12;LHFPL3;DNM3;ATAT1;FCHSD2;APC;CDH10;TTC3;ZMYND11;DSCAML1;ZNF510;ASTN1*
GDNF-AS1	41/100	0.009184611282291914	*PCDHGB7;KHDRBS3;PCDHGB6;PCDHGB4;PCDHGB3;PCDHGB2;PCDH15;AKAP6;GRIK2;LRRTM3;MAP2;SOX8;NCAM1;SOX6;GPR158;GRIA4;CLASP2;PCDHGA8;PCDHGA7;PCDHGA6;PCDHGA5;DSCAM;PCDHGA4;PCDHGA3;PCDHGA2;PCDHGA1;LSAMP;PCDHGA9;PCDHGA10;GNAO1;ATAT1;BCAN;PCDHGA11;P2RX6;PCDHGA12;MMP16;PCDHGB1;MDGA2;DSCAML1;UNC79;ASTN1*
SEMA6A-AS2	41/100	0.009184611282291914	*NLGN1;DENND5B;PCDHGB4;PCDHGB3;DOCK7;FMN1;EDNRB;NKAIN3;UBL3;MXI1;DENND5A;PHACTR1;DIP2C;SOX6;CC2D2A;SRGAP2B;PKNOX2;CLASP2;PCDHGA8;PCDHGA7;ARNT2;MYEF2;PCDHGA4;FBXW11;PCDHGA3;MICAL3;PAX3;CABLES1;MITF;ANK2;MFSD12;QKI;IGSF11;P2RX7;PCDHGA12;NRG3;APC;DAAM2;MDGA2;MAPRE2;SLC24A5*
EDIL3-DT	41/100	0.009184611282291914	*NRP1;GPR21;TGFB1I1;LAMA4;PRUNE2;PRICKLE2;MYLK;LOXL2;MPRIP;CALD1;RAB23;DNAJB4;ABI3BP;DIP2C;SNX7;FILIP1L;EDIL3;GPC6;PRKG1;MEF2A;ARHGEF10;RBFOX2;PDE4D;AFAP1;EML1;BICC1;PTPRD;VCAN;KATNAL1;CDH11;ITGA8;CASQ2;CDH13;MSRB3;ROR1;MRVI1;ESYT2;RAPGEF5;PPP1R12B;VCL;FERMT2*
PPP1R12A-AS1	41/100	0.009184611282291914	*SYNM;RABGAP1;SYNPO2;PRUNE2;STON1;PRICKLE2;ARID4A;CACNA1C;LPP;TOR1AIP1;MYLK;ZCWPW2;PLN;DPP8;CALD1;RAB23;MYH11;PGM5;FPGT-TNNI3K;SBF2;SCAPER;TEAD1;TNS1;EPM2A;NDE1;FBXL17;FOXN3;STON1-GTF2A1L;EML1;CLIP1;SETBP1;JMY;KCNMA*

			1;CCSER2;MSRB3;MRVI1;PPP1R12B;ZMYND11;ZNF510;VCL;FERMT2
NKX2-2-AS1	41/100	0.009184611282291914	*CRB1;PCDHGB7;PCDHGB6;PCDHGB4;PCDHGB3;PCDHGB2;CACNA1A;GRIK4;CRMP1;SLC35F1;NOL4;TRIM9;LRRTM3;SNTG1;BAALC;SOX8;NCAM1;GRIA3;PCDHGA8;PCDHGA7;PCDHGA6;PCDHGA5;DSCAM;WSCD1;PCDHGA4;PCDHGA3;TMEM132B;PCDHGA2;PCDHGA1;GAD2;LHFPL3;PCDHGA9;PCDHGA10;ATAT1;BCAN;PCDHGA11;PCDHGA12;NOVA1;PCDHGB1;CPE;APBA2*
MAPT-AS1	41/100	0.009184611282291914	*CRB1;RTN1;PCDHGB4;CTNND2;NRXN1;GRIK4;CRMP1;ADCY2;FMN2;GRIK2;TRIM9;OPHN1;LRRTM3;MAP2;DENND5A;SOX8;NCAM1;LRRC4C;GRIA3;GRIA4;CLASP2;PCDHGA8;GRID2;PCDHGA5;DSCAM;PCDHGA4;CADM2;PCDHGA3;PCDHGA2;TCF12;PCDHGA1;LSAMP;QKI;GNAO1;PCDHGA10;BCAN;NOVA1;CDH10;PCDHGB1;DSCAML1;ASTN1*
LINC02798	40/100	0.016561025969389458	*PCDHGB7;PCDHGB6;PCDHGB4;CTNND2;PCDHGB3;PCDHGB2;SLC1A3;FMN2;FGF1;CNN3;IQCJ-SCHIP1;TRIM9;SNN;DPYSL2;ADORA3;BAALC;DENND5A;FYN;SRGAP2B;JAM2;PCDHGA8;PCDHGA7;PCDHGA6;DTNA;PCDHGA5;PCDHGA4;PCDHGA3;PCDHGA2;DENND2A;PCDHGA1;CORO2B;QKI;PCDHGA9;PCDHGA10;PCDHGA11;PCDHGA12;NOVA1;RFX4;PCDHGB1;APBA2*
LINC01516	40/100	0.016561025969389458	*PCDHGB7;PCDHGB6;PCDHGB4;CTNND2;PCDHGB3;PCDHGB2;GRIK4;CRMP1;FMN2;TRIM9;DPP6;TSPAN7;BAALC;NCAM1;GRIA3;GPR158;JAM2;GRIA4;CLASP2;PCDHGA8;PCDHGA7;PCDHGA6;PCDHGA5;PCDHGA4;CADM2;PCDHGA3;PCDHGA2;PCDHGA1;LSAMP;PCDHGA9;PCDHGA10;GNAO1;PCDHGA11;PCDHGA12;RFX4;CDH10;REEP2;PCDHGB1;APBA2;ASTN1*
LINC01103	40/100	0.016561025969389458	*PCDHGB7;PCDHGB6;PCDHGB4;PCDHGB3;PCDHGB2;CRMP1;GRIK2;JPH4;TRIM9;DPP6;MAP2;LRRTM4;TSPAN7;NCAM1;LRRC4C;CACNG2;GPR158;GRIA4;CLASP2;PCDHGA8;PCDHGA7;PCDHGA6;PCDHGA5;PCDHGA4;PCDHGA3;PCDHGA2;PCDHGA1;LSAMP;SEZ6L;PCDHGA9;PCDHGA10;GNAO1;IGSF11;PCDHGA11;FAM155A;PCDHGA12;REEP2;PCDHGB1;UNC79;ASTN1*
LINC00298	40/100	0.016561025969389458	*ADCYAP1R1;PCDHGB7;PCDHGB6;PCDHGB4;CTNND2;PCDHGB3;PCDHGB2;SLC1A3;GRIK4;ADCY2;FMN2;NDRG2;TRIM9;SNN;BAALC;DENND5A;NCAM1;KCNN3;JAM2;BBS2;PCDHGA8;PCDHGA7;DTNA;PCDHGA5;PCDHGA4;PCDHGA3;PCDHGA2;PCDHGA1;GPR75;PRKCA;QKI;PCDHGA10;PCDHGA11;APC;DAAM2;RFX4;CDH10;PCDHGB1;SLC25A53;ASTN1*
LINC02058	40/100	0.016561025969389458	*GRIA1;ADCYAP1R1;PCDHGB7;PCDHGB6;PCDHGB4;LUZP2;CTNND2;NRXN1;PCDHGB2;GRIK4;ADCY2;FMN2;NDRG2;SNN;LRRTM3;BAALC;DENND5A;NCAM1;KCNN3;GRIA3;GRIA4;CLASP2;PCDHGA8;NTRK2;GRID2;DTNA;DSCAM;PCDHGA4;CADM2;PCDHGA3;PCDHGA2;PCD*

			HGA1;LSAMP;GPR75;QKI;PCDHGA10;APC;DAAM2;CDH10;PCDHGB1
LINC01340	40/100	0.016561025969389458	*OR11L1;MTPN;PCDHGB7;PCDHGB6;PCDHGB4;RNF180;PCDHGB3;PCDHGB2;SLC35F1;RYR3;CNN3;SPRED3;TRIM9;PDE4B;BAALC;DPF3;TRIM23;PCDHGA8;PCDHGA7;PCDHGA6;PCDHGA5;WSCD1;PCDHGA4;FBXW11;PCDHGA3;PCDHGA2;MAGI2;DENND2A;PCDHGA1;S100B;CORO2B;PCDHGA9;PCDHGA10;IGSF11;PCDHGA11;PCDHGA12;PTPRA;VPS41;PCDHGB1;ASTN1*
LINC02110	40/100	0.016561025969389458	*PCDHGB7;PCDHGB6;PCDHGB4;PCDHGB3;PCDHGB2;GRIK4;CRMP1;GRIK2;LRRTM3;MAP2;SOX8;NCAM1;SOX6;GRIA3;GPR158;GRIA4;CLASP2;PCDHGA8;PCDHGA7;PCDHGA6;PCDHGA5;DSCAM;PCDHGA4;CADM2;PCDHGA3;PCDHGA2;PCDHGA1;LSAMP;PCDHGA9;GNAO1;PCDHGA10;ATAT1;BCAN;PCDHGA11;PCDHGA12;REEP2;PCDHGB1;DSCAML1;UNC79;ASTN1*
LINC02440	40/100	0.016561025969389458	*PCDHGB7;SHC3;PCDHGB6;PCDHGB4;PCDHGB3;PCDHGB2;GRIK4;CRMP1;GRIK2;DRP2;LRRTM3;SNTG1;MAP2;TNR;SOX8;LRRC4C;CACNG2;GRIA3;GRIA4;PCDHGA8;PCDHGA7;PCDHGA6;PCDHGA5;DSCAM;BTBD17;PCDHGA4;PCDHGA3;PCDHGA2;PCDHGA1;LHFPL3;SEZ6L;PCDHGA9;PCDHGA10;ATAT1;BCAN;PCDHGA11;PCDHGA12;PCDHGB1;DSCAML1;UNC79*
MRVI1-AS1	40/100	0.016561025969389458	*RYR2;SYNM;TGFB1I1;SYNPO2;PRUNE2;STON1;PRICKLE2;CACNA1C;LPP;SLC8A1;MYLK;INPP5A;PLN;CALD1;RAB23;MYH11;PGM5;FLNC;FILIP1L;CC2D2A;CAMK2G;PRKG1;TNS1;GTF2A1L;PPP1R14A;TRPC4;NDE1;STON1-GTF2A1L;EML1;ARHGAP10;PALLD;KCNMA1;ITGA8;CASQ2;MSRB3;MRVI1;PPP1R12B;PDZRN3;VCL;FERMT2*
TMEM72-AS1	40/100	0.016561025969389458	*PCDHGB7;RTN1;PCDHGB6;PCDHGB4;CTNND2;PCDHGB3;NRXN1;PCDHGB2;GRIK4;ADCY2;NDRG2;FAM171A1;LRRTM3;MAP2;DENND5A;NCAM1;KCNN3;SH3GL2;GRIA3;GRIA4;CLASP2;BBS2;PCDHGA8;PCDHGA7;ARNT2;PCDHGA6;ZHX3;PCDHGA5;PCDHGA4;CADM2;PCDHGA3;PCDHGA2;NTRK3;PCDHGA1;ANK2;QKI;DNM3;PCDHGB1;DSCAML1;APBA2*
PRICKLE2-AS3	40/100	0.016561025969389458	*MACF1;KMT2C;SYNPO2;STON1;PRICKLE2;ZBTB20;CACNA1C;ARHGAP5;SLC9C1;LPP;SYNE1;KIAA1109;ATXN1;DPP8;CALD1;ZNF407;EVI5;TEAD1;PRKG1;SHPRH;ZNF483;MON2;RALGAPA1;VPS13C;ATRX;STON1-GTF2A1L;EML1;MYO9A;FER;KATNAL1;AGO3;SETBP1;NFIA;PEAK1;KCNMA1;NF1;CCSER2;WDFY3;KIAA0825;FERMT2*
NDP-AS1	40/100	0.016561025969389458	*GRIA1;ADCYAP1R1;PCDHGB7;PCDHGB6;PCDHGB4;CTNND2;PCDHGB3;PCDHGB2;SLC1A3;GRIK4;ADCY2;FMN2;FGF1;NDRG2;ADCY8;NPAS3;IQCJ-SCHIP1;TRIM9;SNN;BAALC;DENND5A;CTNNA2;JAM2;PCDHGA8;PCDHGA7;PCDHGA6;DTNA;PCDHGA5;PCDHGA4;PCDHGA3;PCDHGA2;P*

			CDHGA1;QKI;DCLK1;PCDHGA9;PCDHGA11;PCDHGA12;RFX4;PCDHGB1;APBA2
KCNMA1-AS1	40/100	0.016561025969389458	*SYNM;TGFB1I1;SYNPO2;PRUNE2;STON1;PRICKLE2;NRXN3;CACNA1C;LPP;SLC8A1;MYLK;INPP5A;PLN;CALD1;RAB23;MYH11;PGM5;DMD;FLNC;FILIP1L;PRKG1;TNS1;GTF2A1L;RBFOX2;NDE1;ITGA1;ABCC9;FOXN3;STON1-GTF2A1L;EML1;METTL24;SETBP1;PALLD;KCNMA1;MSRB3;MRVI1;PPP1R12B;PDZRN3;VCL;FERMT2*
NFIA-AS2	40/100	0.016561025969389458	*ADCYAP1R1;PCDHGB6;PCDHGB4;CTNND2;PCDHGB3;PCDHGB2;SLC1A3;ADCY2;FMN2;FGF1;NDRG2;ADCY8;CNN3;IQCJ-SCHIP1;TRIM9;SNN;DPYSL2;ADORA3;BAALC;FYN;KCNN3;JAM2;PCDHGA8;PCDHGA7;PCDHGA6;DTNA;PCDHGA5;PCDHGA4;PCDHGA3;PCDHGA2;DENND2A;QKI;PCDHGA10;PCDHGA11;FRMD5;PCDHGA12;RFX4;PCDHGB1;SLC25A53;ASB3*
MYLK-AS1	40/100	0.016561025969389458	*SYNM;TGFB1I1;SYNPO2;PRUNE2;STON1;CACNA1C;LPP;SLC8A1;MYLK;RAP1A;INPP5A;PLN;CALD1;RAB23;MYH11;PGM5;DMD;FLNC;FILIP1L;PRKG1;TNS1;GTF2A1L;PPP1R14A;TRPC4;NDE1;FBXO32;STON1-GTF2A1L;EML1;ARHGAP10;PALLD;KCNMA1;CDC42EP3;ITGA8;CASQ2;MSRB3;MRVI1;PPP1R12B;PDZRN3;VCL;FERMT2*
ADAMTS9-AS2	40/100	0.016561025969389458	*NRP1;ENPEP;FLT1;PRKAA2;STXBP4;PRUNE2;ZBTB20;PTPRM;RORA;LDB2;SLC8A1;SYNE1;TTC28;PTPRG;PREX2;PKHD1;KIAA1109;ARHGAP42;KDR;ACAD11;CC2D2A;ZNF366;TNS1;MEF2A;CEP112;ZNF483;INSR;ITGA1;SLC16A12;MYO9A;SHROOM4;SNRK;EMCN;SETBP1;WDFY3;ALPK2;UTRN;FBXL5;RBMS2;RBMS3*
LINGO1-AS1	40/100	0.016561025969389458	*PCDHGB7;PCDHGB6;PCDHGB4;CTNND2;PCDHGB3;PCDHGB2;GRIK4;CRMP1;GRIK2;TRIM9;MAP2;TNR;BAALC;SOX8;NCAM1;GRIA3;GRIA4;CLASP2;PCDHGA8;PCDHGA7;PCDHGA6;PCDHGA5;DSCAM;PCDHGA4;PCDHGA3;TMEM132B;PCDHGA2;PCDHGA1;LHFPL3;PCDHGA9;PCDHGA10;ATAT1;BCAN;PCDHGA11;PCDHGA12;NOVA1;PCDHGB1;LRRC3B;DSCAML1;APBA2*
PGR-AS1	40/100	0.016561025969389458	*SPEF2;CLSTN2;ZFAND4;SLC40A1;GREB1L;PIK3R3;LDLRAD4;EFCAB6;CELSR1;AFF3;FAM214A;KIAA0040;TMEM241;RERG;ZBTB41;ADAMTS19;ERBB4;TRPS1;KIAA1324;NRIP1;ISOC1;APBB2;RALGPS2;VAV3;KDM4B;ANKRD30B;LRBA;GREB1;TBC1D9;ELP3;PRLR;ESR1;PBX1;DCDC1;TTC39A;AR;RAB30;ZNF516;CCDC170;ZNF799*
LINC02520	39/100	0.0295713170732 5519	*CRB1;DENND5B;MEGF11;LAMA1;GRIK4;ABAT;GRIK2;DRP2;TRIM9;RELN;OPHN1;LRRTM3;MAP2;ADAMTS18;FAM110B;GPC3;TNR;SOX8;NCAM1;CSMD3;NCAM2;RHBDL3;CACNG2;EPHB1;GRIA3;GPR158;GRIA4;CLASP2;DSCAM;CADM2;TCF12;LHFPL3;SEZ6L;HS3ST4;*

			MYO16;BCAN;FCHSD2;DSCAML1;UNC79
LINC00606	39/100	0.0295713170732 5519	*PCDHGB7;PCDHGB6;PCDHGB4;CTNND2;PCDHGB3;PCDHGB2;SLC1A2;SLC1A3;SLC35F1;FMN2;FGF1;TRIM9;SNN;LRRTM3;BAALC;JAM2;PCDHGA8;PCDHGA7;PCDHGA6;DTNA;PCDHGA5;WSCD1;PCDHGA4;PCDHGA3;PCDHGA2;NTRK3;PCDHGA1;DENND2A;QKI;PCDHGA9;PCDHGA10;BCAN;PCDHGA11;PCDHGA12;APC;RFX4;PCDHGB1;APBA2;ASTN1*
LINC02051	39/100	0.0295713170732 5519	*SLC24A3;RABGAP1;GPR21;TGFB1I1;CRMP1;SLC35F1;FHL5;GRIK2;SEL1L2;TRIM9;PLN;LRRTM3;RAB23;TMEM108;TNR;BAALC;DENND5A;MYH11;NCAM1;EPHB1;GRIA3;PLXNA4;SH3BGR;GRIA4;SPECC1;DSCAM;PCDHGA4;TRPC4;TCF12;PCDHGA1;LHFPL3;PCDHGA9;PCDHGA10;LRRC7;CASQ2;ITGA8;PCDHGB1;MRVI1;ASTN1*
LINC00511	39/100	0.0295713170732 5519	*CRB1;PCDHGB7;PCDHGB6;PCDHGB4;PCDHGB3;PCDHGB2;TRIM9;TMEM189;BAALC;POLR2F;SOX8;BID;PCDHGA8;FARP2;PCDHGA7;PCDHGA6;PCDHGA5;DSCAM;PCDHGA4;ST8SIA1;PCDHGA3;PCDHGA2;PCDHGA1;LSAMP;LHFPL3;S100B;CORO2B;PCDHGA9;IGSF11;PCDHGA10;ATAT1;BCAN;PCDHGA11;FRMD5;PCDHGA12;TMEM117;PCDHGB1;MKRN3;APBA2*
LINC01349	39/100	0.0295713170732 5519	*SEMA5B;ENPEP;ADCYAP1R1;MTMR10;NLGN1;CTNND2;SLC22A2;RNF180;FGF1;CNN3;IQCJ-SCHIP1;SNN;SNX29;ADORA3;DPYSL2;DPF3;DENND5A;KCNN3;ACAD11;SLC16A4;JAM2;SLC17A3;DTNA;ZHX3;SLC16A12;BBOX1;ANK2;QKI;ARHGAP24;KBTBD11;MSRA;DAB2;DAAM2;RFX4;CRYL1;SLC25A53;FBXL5;RGL1;MAPRE2*
LINC01640	39/100	0.0295713170732 5519	*SYNM;KCNE2;C2CD3;TGFB1I1;SYNPO2;STON1;PRICKLE2;NRXN3;CACNA1C;LPP;MED15;INPP5A;KIAA1328;CALD1;MS4A15;PGM5;FILIP1L;PRKG1;RALGPS1;MAGI1;GTF2A1L;ZNF483;RBFOX2;NDE1;FOXN3;STON1-GTF2A1L;EML1;PARD3B;TGFBR3;SETBP1;ASXL3;NFIC;KCNMA1;PI4KA;CCSER2;TTLL11;MRVI1;FERMT2;BMPR1A*
LINC00396	39/100	0.0295713170732 5519	*PCDHGB7;PCDHGB6;PCDHGB4;CTNND2;PCDHGB3;PCDHGB2;SLC1A3;FMN2;FGF1;IQCJ-SCHIP1;TRIM9;SNN;DPYSL2;PDE4B;BAALC;DPF3;JAM2;MAPK4;PCDHGA8;PCDHGA7;ARNT2;PCDHGA6;DTNA;PCDHGA5;PCDHGA4;PCDHGA3;PCDHGA2;DENND2A;PCDHGA1;LSAMP;CORO2B;QKI;PCDHGA9;PCDHGA10;PCDHGA11;PCDHGA12;RFX4;PCDHGB1;APBA2*
LINC01397	39/100	0.0295713170732 5519	*PCDHGB7;PCDHGB6;PCDHGB4;CTNND2;PCDHGB3;PCDHGB2;OR9Q1;ADCY2;FMN2;CNN3;IQCJ-SCHIP1;TRIM9;SNN;DPYSL2;BAALC;GSG1L;DENND5A;NCAM1;PLXNA4;JAM2;PCDHGA8;PCDHGA7;PCDHGA6;DTNA;PCDHGA5;PCDHGA4;PCDHGA3;PCDHGA2;NTRK3;PCDHGA1;QKI;PCDHGA9;PCDHGA10;PCDHGA11;PCDHGA12;RFX4;PCDHGB1;CYLC2;APBA2*

LINC02134	39/100	0.02957131707325519	*PCDHGB7;PCDHGB6;PCDHGB4;PCDHGB3;PCDHGB2;GRIK4;CRMP1;SLC35F1;GRIK2;TRIM9;LRRTM3;SNTG1;MAP2;BAALC;SOX8;GRIA3;PCDHGA8;PCDHGA7;PCDHGA6;PCDHGA5;DSCAM;WSCD1;BTBD17;PCDHGA4;PCDHGA3;PCDHGA2;PCDHGA1;LHFPL3;PCDHGA9;PCDHGA10;ATAT1;BCAN;PCDHGA11;PCDHGA12;NOVA1;CDH10;PCDHGB1;APBA2;ASTN1*
LINC01727	39/100	0.02957131707325519	*PCDHGB7;PCDHGB6;PCDHGB4;PCDHGB3;PCDHGB2;SLC1A2;SLC1A3;ABAT;SLC35F1;FGF1;TRIM9;SNN;GRM5;BAALC;DPF3;RGS6;MAPK4;JAM2;PCDHGA8;LGI1;PCDHGA7;PCDHGA6;DTNA;PCDHGA5;WSCD1;PCDHGA4;PCDHGA3;PCDHGA2;DENND2A;PCDHGA1;ACYP2;SLC39A12;CORO2B;PCDHGA9;PCDHGA11;PCDHGA12;PCDHGB1;ACSBG1;ASTN1*
LINC01651	39/100	0.02957131707325519	*GRIA2;CRB1;CTTNBP2;MEGF11;NRXN1;PCDH15;GRIK4;GRIK2;TRIM9;OPHN1;LRRTM3;MAP2;TNR;SOX8;DENND5A;NCAM1;CSMD3;SOX6;LRRC4C;CACNG2;EPHB1;GRIA3;GRIA4;CLASP2;GRID2;DSCAM;CADM2;PCDHGA3;TCF12;PCDHGA1;LSAMP;LHFPL3;SEZ6L;BCAN;FCHSD2;CDH10;DSCAML1;UNC79;ASTN1*
LMCD1-AS1	39/100	0.02957131707325519	*ADCYAP1R1;PCDHGB7;PCDHGB4;CTNND2;PCDHGB3;NRXN1;SLC1A3;GRIK4;ADCY2;FMN2;NDRG2;IQCJ-SCHIP1;SNN;LRRTM3;BAALC;DENND5A;NCAM1;KCNN3;CLASP2;PCDHGA8;PCDHGA7;ARNT2;NTRK2;DTNA;PCDHGA4;PCDH9;PCDHGA3;PCDHGA2;MAGI2;PCDHGA1;LSAMP;QKI;PCDHGA10;PCDHGA12;APC;DAAM2;RFX4;PCDHGB1;ASB3*
INKA2-AS1	39/100	0.02957131707325519	*PCDHGB7;KHDRBS3;PCDHGB6;PCDHGB4;PCDHGB3;PCDHGB2;GRIK4;CRMP1;GRIK2;TRIM9;DPP6;MAP2;DENND5A;NCAM1;FYN;GRIA3;GRIA4;PCDHGA8;PCDHGA7;PCDHGA6;PCDHGA5;PCDHGA4;PCDHGA3;PCDHGA2;PCDHGA1;LSAMP;PCDHGA9;PCDHGA10;GNAO1;ATAT1;BCAN;PCDHGA11;PCDHGA12;NOVA1;REEP2;PCDHGB1;APBA2;ASTN1;RNF165*
GNG12-AS1	39/100	0.02957131707325519	*SYNM;ABCB5;DOCK7;STON1;SPATA6;CNN3;EDNRB;SGCD;UBL3;TIMP2;HMCN1;DIP2C;EVI5;CC2D2A;WLS;SOX5;PKNOX2;ST3GAL3;EPM2A;GTF2A1L;PHC2;MYEF2;SORT1;STX8;PCDH7;DENND2A;SAMD4A;PAX3;CABLES1;MITF;GNG12;STON1-GTF2A1L;IGSF11;NFIA;RHOJ;PRKD1;PDZRN3;SLC24A5;FERMT2*
MKLN1-AS	39/100	0.02957131707325519	*MTMR10;NLGN1;PLEKHB2;FAM13C;ZBTB20;SLC4A4;CDC14B;ARHGAP12;BCL2L13;IMMP2L;UBL3;OPHN1;SOX6;SBF2;CC2D2A;METTL9;PAQR8;BBS2;ACBD5;ZHX3;MYEF2;FBXW11;SLC2A13;PRKCA;ARHGAP24;KBTBD11;PARD3B;IGSF11;IFT88;TBC1D5;SPATA13;VPS41;NAPEPLD;EXOC4;CRYL1;WDFY3;PRKD1;CDKL1;CDS2*
SOX2-OT	39/100	0.02957131707325519	*PCDHGB7;PCDHGB6;PCDHGB4;CTNND2;PCDHGB3;PCDHGB2;GRIK4;FMN2;NDRG2;IQCJ-SCHIP1;TRIM9;SNN;DPYSL2;BAALC;DENND*

			5A;NCAM1;GRIA3;GRIA4;CLASP2;PCDHGA8;PCDHGA7;PCDHGA6;DTNA;PCDHGA5;DSCAM;PCDHGA4;CADM2;PCDHGA3;PCDHGA2;PCDHGA1;LSAMP;QKI;PCDHGA9;PCDHGA10;PCDHGA11;PCDHGA12;RFX4;PCDHGB1;APBA2
RIC3-DT	39/100	0.02957131707325519	*SPON1;PCDHGB7;PCDHGB6;MAML2;PCDHGB3;CHD7;PCDHGB2;CRMP1;TRIM9;MAP2;DPF3;SOX8;NCAM1;SRGAP3;CSMD2;GRIA3;ANKRD6;PCDHGA8;PCDHGA7;PCDHGA6;PCDHGA5;DSCAM;MUC16;PCDHGA4;PCDHGA3;PCDHGA2;PCDHGA1;QKI;PCDHGA9;PCDHGA10;ATAT1;BCAN;PCDHGA11;FCHSD2;DOK5;NOVA1;PCDHGB1;APBA2;ST6GALNAC5*
SEC23A-AS1	39/100	0.02957131707325519	*SYNM;FAM114A1;TSHZ3;TGFB1I1;SYNPO2;PRUNE2;CACNA1C;MYLK;PANX1;PLN;CALD1;RAB23;POTEE;POTEF;MYH11;FLNC;MGAT2;FILIP1L;TEAD1;PRKG1;RBFOX2;NDE1;AFAP1;FBXL18;ABCC9;FOXN3;GNG12;EML1;STON1-GTF2A1L;IGF2R;PALLD;KCNMA1;MYH9;MSRB3;TTLL11;MRVI1;PPP1R12B;VCL;FERMT2*

Table S2. Overlapping groups of rDNA-contacting gene in initial K562 cells are co-expressed with 198 different long non-coding RNAs.

Term	Overlap	Adjusted P-value	Genes
PRICKLE2-AS3	51/100	7.26105952383235E-10	*CHD9;ZBTB20;SLC9C1;SYNE1;AKAP11;ZNF407;TEAD1;PRKG1;RALGAPA1;VPS13C;ATRX;RC3H1;VPS13B;ASH1L;STON1-GTF2A1L;EML1;SETBP1;PEAK1;KCNMA1;WDFY3;BIRC6;MACF1;KMT2C;PRICKLE2;RGPD5;RGPD8;UBR1;CACNA1C;LPP;PCNX1;ATXN1;HECTD2;CALD1;EVI5;SVIL;MBD5;MON2;ERCC6L2;LNPEP;PLEKHA3;HOOK3;PHC3;MYO9A;HIPK3;FER;AGO3;NFIA;SLMAP;CCSER2;BRWD1;KIAA0825*
LINC02827	49/100	7.3597740204547755E-9	*KDM5A;PATJ;CHD6;TRPS1;POTED;SAMD12;PRMT8;CERS6;RALGAPA1;ZNF160;MRTFB;TBC1D9;VPS13B;ASH1L;EDAR;KIAA1217;SPOPL;LMX1B;ZNF236;SHANK2;GREB1L;RGPD6;RGPD5;RGPD8;FAM214A;TMEM241;MIPOL1;CRACR2A;ADAMTS18;NSD1;KHDC4;SLC25A21;APBB2;MARCHF6;ANKRD26;AUTS2;ANKRD30B;ANKRD30A;LRBA;DNAH14;DEFB108B;ESR1;GON4L;TTC6;KCNS3;SP3;TASOR2;NEK10;KIAA0825*
GSN-AS1	46/100	2.4996563735110316E-7	*MACF1;ANKRD36;KMT2C;TULP4;FMN1;ASAP1;LPP;DOCK10;STK10;SRGAP2C;AKAP13;FYCO1;ZFYVE26;DSTYK;ATXN1;SGCD;POTEJ;ZNF407;ABL2;HMCN1;MAP4;ERC1;SRGAP2;EVI5;TEAD1;SRGAP2B;KIRREL1;MBD5;ERCC6L2;DENND2B;SAMD4A;MYO5A;VPS13B;MITF;MYO9B;ASH1L;LHFPL2;TANC1;FER;NIN;PEAK1;SLMAP;CDC42EP3;BIRC6;VCL;TNRC6B*
LRRC7-AS1	46/100	2.4996563735110316E-7	*ADCYAP1R1;GABRB1;RTN1;CTNND2;NRXN1;KLHL32;SLC1A2;OTUD7A;ADAM22;FMN2;HTR2A;NDRG2;KIAA0513;GRM5;BRINP1;FUT9;MAP2;NCS1;CHN1;DLGAP1;NCAM1;ERC2;WASF3;OPCML;ARNT2;DTNA;CADM2;NTRK3;TMOD2;LSAMP;ANK2;SLC39A12;GRIN2B;SYN2;SNAP91;GABRG1;CNKSR2;TTLL7;DLG2;SYNJ1;APC;ADGRB3;LRRC7;PPP2R2B;ASTN1;RAPGEF4*

TUB-AS1	45/100	7.540575140311747E-7	*NFAT5;TBC1D19;ACSS3;CUL5;PPM1L;PRUNE2;ZBTB20;ACSM2A;SLC5A12;PEPD;TRHDE;TSPAN33;ACSM2B;HS6ST3;CDH7;SNX29;AP5M1;KIAA1328;PLCZ1;KIF21A;THSD7A;SLC16A9;SLC17A1;RNF152;TINAG;CUBN;KL;STPG2;PDE4D;SLC2A13;WDR72;UNC5D;ARHGAP24;MOB1B;MSRA;PLCXD3;CLCN5;SYT10;RRAGD;LRRC9;WDFY3;CNTN3;DGKI;CNTNAP5;CPEB4*
LINC02720	44/100	2.2776424959244053E-6	*UHRF1BP1L;CUL5;PPM1L;ZBTB20;ITPR2;LDLRAD4;FAM214A;TMEM241;HS6ST3;CDH7;KIAA1328;PLCZ1;ERBB4;TRPS1;FAM241A;APBB2;THSD7A;RNF152;RALGPS2;VAV3;UNC13C;MON2;STPG2;LRBA;TBC1D9;GFRA1;SCAMP1;PRLR;ESR1;PBX1;MOB1B;INPP4B;RABEP1;SYT10;CCNG2;LRRC9;NEK10;SPOPL;ZNF678;LMX1B;BRWD1;KIAA0825;DGKI;CNTNAP5*
ARHGEF7-IT1	43/100	5.289799755006147E-6	*DOCK4;MAML2;PPM1L;CTTNBP2;ADAM22;ZBTB20;AKAP11;SNTG1;MAP2;SACS;NCAM1;SCAPER;MPDZ;TRIM23;ZNF462;KLF12;MBD5;TMOD2;TCF12;KIAA0232;IL17RD;MYO9A;PJA2;PYGO1;DNM3;ZEB1;NBEA;MMP16;SETBP1;APC;ADGRB3;PEAK1;NFIB;TTC3;RAPGEF2;WDFY3;FAT3;TCF4;CNTN3;PIK3C3;FAT4;ARHGEF7;PAFAH1B1*
MRPS30-DT	43/100	5.289799755006147E-6	*PTPRT;CLSTN2;KCNE4;LDLRAD3;AFF3;FAM214A;TMEM241;RERG;SLC7A2;TMEM25;ERBB4;TRPS1;FAM241A;SLC39A6;POTED;APBB2;RALGPS2;VAV3;KDM4B;CERS6;SIAH2;LRBA;MRTFB;CYBRD1;TBC1D9;ELP2;GFRA1;PRLR;ESR1;PBX1;MED13L;RABEP1;TSPAN13;NAT1;TTC6;KIF16B;BCL2;NEK10;SPOPL;LMX1B;BMPR1B;FSIP1;FGF10*
PGR-AS1	43/100	5.289799755006147E-6	*CLSTN2;ZFAND4;SLC40A1;GREB1L;PIK3R3;LDLRAD4;EFCAB6;AFF3;FAM214A;GALNT10;TMEM241;RERG;TMEM25;ADAMTS19;ERBB4;TRPS1;NRIP1;FAM241A;SPIN1;ST8SIA6;APBB2;RALGPS2;VAV3;ANKRD26;KDM4B;ANKRD30B;EYA2;SIAH2;LRBA;GREB1;MRTFB;TBC1D9;GFRA1;PRLR;ESR1;PBX1;DCDC1;INPP4B;RABEP1;NAT1;NEK10;SPOPL;LMX1B*
LINC00472	42/100	1.3282243356481516E-5	*CYFIP2;NFAT5;PATJ;PRKAA2;CUL5;STXBP4;KMT2C;RGPD6;ZBTB20;RGPD5;RGPD8;EFCAB6;SYNE2;SYNE1;KIAA1328;ERBB4;ZNF407;THSD7A;RNF152;ZNF124;ANKRD26;MBD5;MON2;ARHGEF12;STPG2;ERCC6L2;LRBA;DNAH14;VPS13B;LNPEP;ASH1L;PPP2R3A;PHC3;MYO9A;MOB1B;ZNF717;SETBP1;WDFY3;UTRN;BRWD1;KIAA0825;DOCK1*
LINC01651	42/100	1.3282243356481516E-5	*CRB1;GALNT13;PID1;PLPPR1;PHLPP1;CTTNBP2;MEGF11;NRXN1;PCDH15;ADAM22;GRIK4;GRIK2;TRIM9;MAP2;CDH20;DNER;GDAP1L1;TNR;NCAM1;KIF21B;CSMD3;SOX6;LRRC4C;CACNG2;EPHB1;GRIA4;RFTN2;GRID2;DSCAM;SPHKAP;CADM2;TCF12;LSAMP;LHFPL3;SEZ6L;MYT1;FCHSD2;ADGRB3;SMOC1;SCG3;UNC79;ASTN1*
SYNE1-AS1	42/100	1.3282243356481516E-5	*TBC1D19;DOCK4;PRKAA2;STXBP4;CHD9;GLIS3;PRICKLE2;ZBTB20;SYNE2;SYNE1;PTPRG;TTC28;ARHGAP42;SNX29;BBS9;DIP2C;TEAD1;ZNF124;TRIM23;MBD5;ARHGEF12;ERCC6L2;SLC2A13;PDE4D;PPP2R3A;CDC42BPA;MYO9A;ARHGAP24;PJA2;HIPK3;FER;ARHGAP31;SNRK;LATS2;COL4A3;CCSER2;RAPGEF2;WDFY3;FAT4;UTRN;PTPN4;DOCK1*
LINC01087	41/100	3.204513456367577E-5	*PATJ;ANKRD36;TULP4;LDLRAD4;FAM214A;MIPOL1;HECTD2;POTEJ;TRPS1;PSD3;NRIP1;POTED;POTEG;ANKRD26;MON2;ADGRV1;ANKRD30B;ANKRD30A;LRBA*

			;DNAH14;OR1L6;MRTFB;TBC1D9;VPS13B;PLXDC2;PRLR;LRP1B;PBX1;VMP1;INPP4B;TTC6;CCNG2;AKAP9;BCL2;NEK10;SPOPL;ZNF678;BRWD1;FSIP1;KIAA0825;ANKRD36B
LINC01906	41/100	3.204513456367577E-5	*PATJ;CLSTN2;ANKRD36;KMT2C;GREB1L;LDLRAD4;SLC9C1;POTEJ;TRPS1;ZNF407;POTED;APBB2;ANKRD26;MON2;MBD5;ANKRD30B;ANKRD30A;LRBA;DNAH14;MUC19;OR1L6;VPS13B;ASH1L;DEFB108B;ESR1;PHC3;LRP1B;PBX1;INPP4B;ZNF717;TTC6;AKAP9;TASOR2;SPOPL;ZNF678;BIRC6;BRWD1;ZNF236;KIAA0825;ANKRD36B;SHANK2*
PRICKLE2-AS1	41/100	3.204513456367577E-5	*RABGAP1;PRUNE2;PRICKLE2;CACNA1C;LPP;FYCO1;ATXN1;AKAP11;MPRIP;CALD1;ABL1;MAP4;DIP2C;MPDZ;TEAD1;PRKG1;SVIL;STARD13;NEGR1;AFAP1;SHISAL1;ABCC9;FOXN3;STON1-GTF2A1L;EML1;PJA2;FER;ARHGAP31;ZEB1;SETBP1;APC;PEAK1;SLMAP;KCNMA1;CCSER2;WDFY3;TTLL11;PPP1R12B;PDZRN3;VCL;PAFAH1B1*
SLC14A2-AS1	41/100	3.204513456367577E-5	*SLC44A5;NFAT5;TBC1D19;ACSS3;CUL5;PPM1L;ZDHHC21;NYAP2;PPP1R9A;CDH7;HS6ST3;MTMR7;MIPOL1;KIAA1328;PLCZ1;TOGARAM1;THSD7A;RNF152;SLC15A5;ZNF124;UNC13C;MBD5;STPG2;FANCM;RALGAPA1;PDE4D;DNAH14;POU6F2;EBF2;HOOK3;FOXP2;HIPK3;MOB1B;ZNF717;TRAPPC6B;SYT10;LRRC9;ZNF678;ATP6V0D2;DGKI;CNTNAP5*
LINC00689	40/100	6.0942217326211904E-5	*CRB1;GALNT13;PID1;DGKB;CTTNBP2;MEGF11;LUZP2;PCDH15;ADAM22;GRIK4;GRIK2;TRIM9;MAP2;CDH20;DNER;GDAP1L1;PHACTR3;LRIG1;TNR;GSG1L;NCAM1;FYN;KIF21B;CSMD3;SOX6;EPHB1;GRIA4;RFTN2;GRID2;DSCAM;CADM2;TCF12;LSAMP;LHFPL3;ATAT1;FCHSD2;SMOC1;UST;SCG3;FGF12*
LINC00498	40/100	6.0942217326211904E-5	*CLSTN2;ANKRD36;KMT2C;TULP4;GREB1L;ITPR2;LDLRAD4;ERBB4;POTEJ;TRPS1;ZNF407;NSD1;ST8SIA6;CEP192;APBB2;ANKS1B;ANKRD26;ANKRD30B;ANKRD30A;LRBA;DNAH14;VPS13B;TBC1D9;ASH1L;DEFB108B;ESR1;PBX1;INPP4B;GON4L;RABEP1;TTC6;TASOR2;SPOPL;ZNF678;BIRC6;BRWD1;ZNF236;KIAA0825;ANKRD36B;SHANK2*
CYP1B1-AS1	40/100	6.0942217326211904E-5	*NOTCH2;CCDC186;SGMS1;SEL1L;RGPD8;LPP;RGPD4;FYCO1;EFR3A;SCAF8;ATXN1;CALD1;HLCS;ZNF407;HIVEP1;PHACTR2;PDLIM5;ABCC4;SVIL;ZFHX3;IL1R1;BICRAL;DENND4C;LNPEP;ASH1L;PLXDC2;STON1-GTF2A1L;HIPK3;PARD3B;KIAA1217;CRISPLD2;ZNF615;ZNF613;ROR1;FAT4;UTRN;VCL;JCAD;FKBP5;SNTB2*
MYCBP2-AS1	40/100	6.0942217326211904E-5	*ZNF573;ANKRD17;MACF1;SETD2;INO80D;ROCK1;CHD9;ZNF292;KMT2C;LTN1;BAZ2B;ZC3HAV1;PRDM10;PCNX1;NIPBL;AKAP11;BTAF1;HERC1;BPTF;USP24;SPEN;ERCC6L2;VPS13C;MGA;TRAPPC10;ATRX;YLPM1;RC3H1;VPS13B;ASH1L;GAPVD1;ARID1B;RC3H2;ZZEF1;NIN;NCOR1;AGO3;RFX7;BIRC6;TNRC6B*
NMBR-AS1	40/100	6.0942217326211904E-5	*FTO;TPH2;ATP8A2;MAST4;KCNE4;GRIK1;LRP2;FRY;CELSR2;PRSS23;RERG;SLC7A2;FAM107B;PHF21B;TMEM25;ADAM29;DACH1;ERBB4;SUSD4;MALRD1;PRKACB;EDIL3;PCDH9;NEGR1;SIAH3;SEMA6D;MAGI2;CYBRD1;ESR1;GABRG2;DCDC1;DCLK1;MED13L;CACNB2;SLCO3A1;KCNS3;CDHR3;LMX1B;FGF12;FGF10*
PRICKLE	40/100	6.0942217326	*NFAT5;INO80D;ANKRD36;CHD9;KMT2C;RGPD6;PRIC*

2-AS2		211904E-5	*KLE2;ZBTB20;RGPD5;ZNF518A;RGPD8;BAZ2B;LPP;SYNE1;ATXN1;HECTD2;ZNF407;ZNF124;ANKRD36C;MBD5;MON2;RALGAPA1;ANKRD30A;ERCC6L2;VPS13C;OR1L6;RC3H1;VPS13B;ASH1L;PHC3;VMP1;FER;AGO3;WDFY3;BIRC6;UTRN;BRWD1;KIAA0825;ANKRD36B;TNRC6B*
CXXC4-AS1	40/100	6.0942217326211904E-5	*GALNT13;DGKB;CTNND2;NRXN1;PCDH15;ADAM22;GRIK4;GRIK2;NALCN;DPP6;MAP2;CDH20;DNER;TNR;NCAM1;NCAM2;LRRC4C;CACNG2;GPR158;TRIM23;GRIA4;RFTN2;GRID2;DSCAM;KCND2;CADM2;TMOD2;TCF12;LSAMP;LHFPL3;SEZ6L;MYT1;DNM3;ZEB1;APC;ADGRB3;CNTN1;SCG3;UNC79;ASTN1*
FAM198B-AS1	40/100	6.0942217326211904E-5	*UHRF1BP1L;SEL1L;LDLRAD4;AFF3;FAM214A;RERG;PRRC1;TRPS1;FAM241A;TRAPPC11;TOGARAM1;ST8SIA6;APBB2;FLNB;ARFGEF3;RALGPS2;TTC37;KDM4B;CERS6;LRBA;MRTFB;TBC1D9;GFRA1;ASH1L;PRLR;ESR1;PBX1;CDYL2;MED13L;INPP4B;RABEP1;TTC6;KIF16B;CCNG2;NEK10;SPOPL;LMX1B;DHX29;BMPR1B;FSIP1*
RERG-AS1	40/100	6.0942217326211904E-5	*PATJ;TBC1D19;ACSS3;PRKAA2;CUL5;ANKRD36;ZBTB20;EPB41L4A;KIAA1328;PLCZ1;ZNF407;POTED;THSD7A;RNF152;ANKRD26;MON2;MBD5;STPG2;ANKRD30B;ANKRD30A;DNAH14;OR1L6;VPS13B;DEFB108B;PHC3;MYO9A;LRP1B;MOB1B;INPP4B;TBC1D1;ZNF717;RCAN2;SYT10;ERP27;WDFY3;ATP6V0D2;KIAA0825;DGKI;ANKRD36B;CPEB4*
SFTPD-AS1	40/100	6.0942217326211904E-5	*NFAT5;ANKRD36;CHD9;RGPD6;ZBTB20;RGPD5;RORA;ZDHHC21;BAZ2B;PPP1R9A;SNX30;SLC9C1;SYNE1;PTAR1;MACROD2;SCAI;ANKRD36C;DNAH11;MON2;MBD5;WSB1;RALGAPA1;VPS13C;MTUS1;PLEKHA3;HOOK3;BTBD9;PTPN13;CDC42BPA;PHC3;KCNQ3;GSAP;ZNF780B;FAT4;BRWD1;RAPGEF5;PTPN4;KIAA0825;ANKRD36B;RBMS3*
LINC02552	39/100	1.5171570268143067E-4	*SHC4;CRB1;PHLPP1;XYLT1;FMN1;PTPRJ;AKAP6;FCRLA;STK10;SDCBP;SGCD;UBL3;GNG2;SCFD2;LRRTM4;CHCHD6;GNG7;ZNF106;PRAME;HMCN1;STK32A;DISC1;SOX5;PKNOX2;MYEF2;EYA1;MYO10;PCDH7;MOK;MYO5A;MITF;NSG1;S100B;CORO2B;IGSF11;NELL1;NRG3;MDGA2;CNIH3*
SRGAP3-AS1	39/100	1.5171570268143067E-4	*ADCYAP1R1;GALNT13;RTN1;CTNND2;NRXN1;KLHL32;ADAM22;GRIK4;NALCN;NPAS3;NKAIN3;MAP2;CDH20;LRIG1;TNR;DLGAP1;NCAM1;SRGAP3;LRRC4C;WASF3;GRIA4;RFTN2;ARNT2;GRID2;DSCAM;KCND2;CADM2;NTRK3;TMOD2;TCF12;LSAMP;MAPK8IP1;DNM3;APC;ADGRB3;PTPRA;TBC1D5;PPP2R2B;ASTN1*
CHL1-AS1	39/100	1.5171570268143067E-4	*SEMA5A;SHC4;MTPN;NLGN1;PHLPP1;MEGF10;PTPRJ;SLC35F1;FMN1;AKAP6;SDCBP;DSTYK;NKAIN3;UBL3;GNG7;ANKFY1;ITGB8;ZNF106;HMCN1;DIP2C;STK32A;SOX5;ARNT2;MYEF2;TMEM178B;MYO10;KAZN;MYO5A;MITF;ANK2;S100B;CORO2B;IGSF11;NELL1;NRG3;TBC1D5;VPS41;CNIH3;CREB5*
ADAMTS9-AS2	39/100	1.5171570268143067E-4	*NRP1;ENPEP;TBC1D19;FLT1;PRKAA2;STXBP4;ARHGEF28;CEP120;PRUNE2;ZBTB20;RORA;LDB2;SLC8A1;SYNE1;TTC28;PTPRG;ARHGAP42;EPB41L4A;FCHO2;CEP112;MBD5;ARHGEF12;INSR;ITGA1;CRIM1;LNPEP;ANO6;MYO9A;PJA2;HIPK3;ZNF33B;SNRK;SETBP1;WDFY3;ALPK2;FAT4;ZNF611;UTRN;RBMS3*
WDFY3-AS1	39/100	1.5171570268143067E-4	*ZNF573;ANKRD17;DDX6;UHRF1BP1L;CHD9;KMT2C;LTN1;ZBTB20;RGPD8;UBR1;RGPD4;PCNX1;ATXN1;AK*

			AP11;HERC1;ZNF407;HECTD4;TRAPPC11;TOGARAM1;MBD5;MON2;ARHGEF12;RALGAPA1;ERCC6L2;LRBA;DENND4C;VPS13D;ATRX;VPS13B;ASH1L;PHC3;MYO9A;RC3H2;HIPK3;FER;WDFY3;BIRC6;ZNF236;KIAA0825
LINC02520	38/100	3.527775382613342E-4	*DGKG;CRB1;GALNT13;PID1;PLPPR1;MEGF11;LAMA1;ADAM22;GRIK4;GRIK2;TRIM9;RELN;MAP2;ADAMTS18;DNER;GDAP1L1;TNR;NCAM1;KIF21B;CSMD3;NCAM2;CACNG2;EPHB1;GPR158;WASF3;GRIA4;DSCAM;CADM2;TCF12;LHFPL3;SEZ6L;MYT1;HS3ST4;FCHSD2;ZEB1;SMOC1;SCG3;UNC79*
PPP3CB-AS1	38/100	3.527775382613342E-4	*STX12;GALNT13;DOCK3;RABGAP1;PHLPP1;ATP8A1;KLHL32;GRIK4;BAZ2B;PPP1R9A;HIRA;CDH20;HECTD4;PHACTR3;LRIG1;ANKFY1;EPC2;SRGAP3;SCAPER;RALGPS1;WASF3;ATP9A;RFTN2;NTRK2;SLC15A2;NEK4;VPS13D;ARAP2;FAM189A2;YLPM1;ATAT1;TOM1L2;TAOK3;TBC1D5;RUFY2;UST;ZMYND11;ASB3*
PACRG-AS2	38/100	3.527775382613342E-4	*NFAT5;ACSS3;CUL5;PDE1C;PPM1L;ZBTB20;TRHDE;CDH7;HS6ST3;AP5M1;KIAA1328;PLCZ1;TRIM2;ZNF208;KIF21A;THSD7A;STXBP6;RNF152;UNC13C;FYB2;STPG2;ABCA5;PDE4D;SLC2A13;ADAM32;SCAMP1;MOB1B;INPP4B;TBC1D1;LRFN5;TMEM116;SYT10;LRRC9;ERP27;ATP6V0D2;DGKI;CNTNAP5;ZNF431*
SLC8A1-AS1	38/100	3.527775382613342E-4	*MARCHF1;CHD9;PRUNE2;RNF38;PRICKLE2;ZBTB20;CACNA1C;FRY;LPP;SLC8A1;SYNE1;ATXN1;AKAP11;CALD1;TSPAN2;PGM5;TEAD1;PRKG1;MBD5;ERCC6L2;PDE4D;TPM1;LNPEP;FOXN3;STON1-GTF2A1L;EML1;MYO9A;PJA2;HIPK3;FER;ZEB1;NBEA;SETBP1;SLMAP;KCNMA1;CCSER2;WDFY3;PPP1R12B*
MYO16-AS2	38/100	3.527775382613342E-4	*NFAT5;ACSS3;MARCHF1;CUL5;PPM1L;ZBTB20;CDH7;HS6ST3;FGD4;AP5M1;KIAA1328;PLCZ1;TRIM2;ZNF208;KIF21A;DPH6;THSD7A;RNF152;UNC13C;FYB2;STPG2;ABCA5;ERCC6L2;PDE4D;SLC2A13;ADAM32;SCAMP1;MOB1B;INPP4B;LRFN5;SYT10;LRRC9;ERP27;ATP6V0D2;DGKI;CNTNAP5;ZNF431;CPEB4*
TMEM26-AS1	38/100	3.527775382613342E-4	*PTPRT;CLSTN2;ZFAND4;LDLRAD3;AFF3;FAM214A;RERG;TMEM25;HHAT;TRPS1;FAM241A;POTED;PIEZO2;SLC39A6;ST8SIA6;FLNB;KDM4B;LRBA;GREB1;MRTFB;CYBRD1;TBC1D9;ELP2;GFRA1;PRLR;ESR1;DCDC1;CDYL2;INPP4B;RABEP1;TTC6;KIF16B;CCNG2;BCL2;NEK10;SPOPL;LMX1B;BMPR1B*
NAV2-AS2	38/100	3.527775382613342E-4	*OCA2;DOCK9;FMN1;ATP10A;AMBRA1;ZFYVE1;TRPM1;FYCO1;ZFYVE26;DSTYK;UBL3;SGCD;MPRIP;KIF13A;ABL1;ANKFY1;NPHP4;ZNF106;ERC1;STK32A;DIP2C;TEAD1;ARHGEF11;MYEF2;MYO10;DENND2B;SAMD4A;MYO5A;CABLES1;MITF;NAV2;GPR137B;ITPKB;TANC1;FER;RAB38;ST6GALNAC3;ITGA9*
LINC00862	37/100	7.054326517377347E-4	*LPGAT1;WDR26;CLSTN2;ANKRD36;KMT2C;GREB1L;LDLRAD4;MIPOL1;ODR4;KIAA1328;ZNF407;EYS;RALGPS2;ANKRD36C;ANKRD26;ANKRD30B;ANKRD30A;SLX4IP;DNAH14;RC3H1;VPS13B;ASH1L;DEFB108B;ESR1;PBX1;INPP4B;GON4L;TTC6;TASOR2;RAB3GAP2;ZNF678;BIRC6;BRWD1;ZNF236;KIAA0825;ANKRD36B;SHANK2*
LINC02571	37/100	7.054326517377347E-4	*PATJ;PDXDC1;GREB1L;AFF3;DUX4;FAM214A;TMEM241;MIPOL1;TMEM25;ERBB4;TRPS1;POTED;KIAA0319L;ARFGEF3;POTEC;CERS6;ANKRD30A;LRBA;MRTFB;TBC1D9;GFRA1;ASH1L;TC2N;PRLR;ESR1;PBX1;CD*

			YL2;RABEP1;TSPAN13;TTC6;DOP1B;CCNG2;MYO5C;NEK10;SPOPL;LMX1B;FSIP1
LINC01538	37/100	7.054326517377347E-4	NFAT5;ACSS3;MARCHF1;CUL5;PDE1C;PPM1L;ZBTB20;MSI2;CDH7;HS6ST3;AP5M1;KIAA1328;PLCZ1;TRIM2;ZNF208;KIF21A;THSD7A;RNF152;UNC13C;STPG2;ABCA5;ERCC6L2;PDE4D;SLC2A13;ADAM32;SCAMP1;MOB1B;INPP4B;LRFN5;GNAQ;SYT10;LRRC9;ERP27;ATP6V0D2;DGKI;CNTNAP5;ZNF431
LINC01801	37/100	7.054326517377347E-4	ZNF573;ANKRD17;MACF1;ZNF292;IREB2;CHD6;BAZ2A;AFF3;SCAF8;HERC1;PPIP5K2;TTC21B;NSD1;MYB;TOGARAM1;EPC2;ST8SIA6;BPTF;USP24;CREBBP;CNOT6L;ITGA4;BICRAL;LRBA;DENND4C;TBC1D9;VPS13B;ASH1L;MYO9A;FLI1;MED13L;ZNF33B;ZNF718;ELF2;BCL2;SPOPL;ZNF611
SHANK2-AS3	37/100	7.054326517377347E-4	KMT2E;INO80D;ANKRD36;KMT2C;GREB1L;ZBTB20;LDLRAD4;BAZ2B;KIAA1328;ZNF407;NSD1;HIVEP1;ANKRD26;MBD5;ANKRD30B;ANKRD30A;LRBA;MGA;DNAH14;OR1L6;RC3H1;VPS13B;ASH1L;DEFB108B;PHC3;ZNF717;TTC6;TASOR2;ZNF678;BIRC6;UTRN;BRWD1;ZNF236;KIAA0825;ANKRD36B;SHANK2;TNRC6B
ZNF32-AS3	37/100	7.054326517377347E-4	GALNT13;NRXN1;ADAM22;GRIK4;MAP2;CDH20;LRIG1;TNR;DLGAP1;NCAM1;SRGAP3;NCAM2;LRRC4C;EPHB1;WASF3;TRIM23;GRIA4;RFTN2;GRID2;DSCAM;SPHKAP;KCND2;CADM2;NTRK3;TMOD2;TCF12;LHFPL3;DNM3;ATAT1;ZEB1;FCHSD2;APC;ADGRB3;RUFY2;TTC3;ZMYND11;ASTN1
INHBA-AS1	37/100	7.054326517377347E-4	NLGN1;RTN1;KCNC1;MYT1L;MEGF11;NTM;NRXN1;DIRAS2;KLHL32;OTUD7A;ADAM22;GRIK2;HTR2A;SLC6A1;GRM5;BRINP1;TNR;CACNG2;ANKS1B;TRIM23;RGS7;GRIA4;OPCML;DSCAM;TMOD2;SYT16;FAM219A;SEZ6L;GRIN2B;SNAP91;DNM3;SYNJ1;APC;ADGRB3;MAPRE2;UNC79;RAPGEF4
VWC2L-IT1	37/100	7.054326517377347E-4	DGKG;GRIA1;GABRB1;GALNT13;RTN1;KCNC1;MEGF11;NRXN1;KLHL32;ADAM22;SLC35F1;GRIK2;SLC6A1;GRM5;MAP2;SNTG1;GDAP1L1;TNR;LRRC4C;CACNG2;GPR158;GRIA4;OPCML;CADM2;TMOD2;FAM219A;LHFPL3;SEZ6L;SNAP91;EPN2;DNM3;SYNJ1;APC;ADGRB3;PPP2R2B;UNC79;ASTN1
UST-AS1	37/100	7.054326517377347E-4	ZNF573;MACF1;NFAT5;PPM1L;CHD9;KMT2C;WDR41;RGPD6;ZBTB20;RGPD5;RGPD8;BAZ2B;SLC9C1;PLCZ1;ZNF407;SCAPER;EVI5;ZNF462;MBD5;ST6GAL2;ERCC6L2;VPS13D;OR1L6;HOOK3;PHC3;PYGO1;MOB1B;FER;PDP2;UST;LPCAT2;CCSER2;ZNF780B;PTPN4;DGKI;ZNF431;TNRC6B
OBI1-AS1	37/100	7.054326517377347E-4	ADCYAP1R1;GALNT13;PHLPP1;HEPACAM;LUZP2;CTNND2;NRXN1;KLHL32;PCDH15;ADAM22;GRIK4;FMN2;NDRG2;NKAIN3;AKAP11;CDH20;FUT9;ANKFY1;NCAM1;KIF21B;KCNN3;WASF3;RFTN2;NTRK2;ARNT2;KCND2;CADM2;TMOD2;TCF12;LSAMP;CCDC88A;FCHSD2;APC;ADGRB3;PPP2R2B;ARHGEF7;ASB3
PTPRG-AS1	37/100	7.054326517377347E-4	IPO11;DOCK4;CUL5;STXBP4;ZBTB20;RGPD8;UBR1;PTPRG;ARHGAP42;SPRED1;ATXN1;AKAP11;ZNF449;BBS9;TOGARAM1;EPC2;EVI5;TEAD1;TRIM23;TTC37;MBD5;ARHGEF12;ERCC6L2;ERBIN;SMAD5;MYO9A;PJA2;MOSMO;FER;SNRK;TBC1D5;PEAK1;HECW2;RAPGEF2;WDFY3;FAT4;CFAP97
FGF10-AS1	37/100	7.054326517377347E-4	PTPRT;CLSTN2;MAST4;KCNE4;GRIK1;LRP2;AFF3;PRSS23;FAM214A;RERG;GLI3;SLC7A2;TMEM25;DACH1;ERBB4;TRPS1;FAM241A;PIEZO2;SLC39A6;ENPP1

			;ST8SIA6;MALRD1;PRKACB;EDIL3;AMFR;CYBRD1;TBC1D9;ELP2;ESR1;DCDC1;MED13L;CACNB2;RABEP1;KIF16B;LMX1B;BMPR1B;FGF10
LRP1-AS	37/100	7.054326517377347E-4	NOTCH2;UHRF1BP1L;DOCK4;TRIO;CHD9;ROCK2;KMT2C;RGPD8;ANTXR1;AKAP13;PCNX1;ATXN1;AKAP11;HERC1;ZNF407;HIVEP1;EVI5;TEAD1;CREBBP;ERCC6L2;DENND4C;EBF1;AFAP1;VPS13B;FOXN3;ASH1L;PLXDC2;ZZEF1;FER;VCAN;PEAK1;CDH11;WDFY3;BIRC6;FAT4;ZNF236;FBN1
FRMD6-AS1	37/100	7.054326517377347E-4	CHD6;TULP4;GREB1L;LDLRAD3;DUX4;GLI3;MIPOL1;RPS6KA5;HECTD2;TRPS1;ZNF407;POTED;ABL2;TOGARAM1;POTEC;SUPT16H;ANKRD26;FANCM;ANKRD30A;DNAH14;MRTFB;EXOC6B;TBC1D9;ASH1L;MCC;PRLR;ESR1;DCDC1;KTN1;FRMD6;GON4L;RABEP1;TTC6;NEK10;BRWD1;ZNF236;HDGFL3
LINC00504	36/100	0.0015793057335155385	PTPRT;IGSF3;TULP4;LDLRAD3;FMN1;AFF3;FAM214A;TMEM241;GLI3;FCRLA;HHAT;TRPS1;FAM241A;SLC39A6;ABL2;ST8SIA6;IARS2;RALGPS2;VAV3;CERS6;MRTFB;TBC1D9;ELP2;MITF;GFRA1;PRLR;ESR1;GPR137B;INPP4B;RABEP1;KIF16B;SPOPL;ENTHD1;LMX1B;BMPR1B;FSIP1
ANKRD62P1-PARP4P3	36/100	0.0015793057335155385	CRB1;ADCYAP1R1;GALNT13;LUZP2;CTNND2;PCDH15;GRIK3;GRIK4;NDRG2;CDH20;TRPS1;LRIG1;MARCHF8;DLGAP1;NCAM1;KCNN3;CSMD2;GRIA4;RFTN2;NTRK2;HFM1;DSCAM;SPHKAP;KCND2;CADM2;TCF12;DNM3;TNRC6C;FRMD5;FCHSD2;NFIA;SMOC1;ADGRB1;TTC3;PWWP3A;ZMYND11
PKP4-AS1	36/100	0.0015793057335155385	ANKRD17;NFAT5;PATJ;INO80D;STXBP4;ZNF292;KMT2C;TULP4;RGPD6;RGPD5;RGPD8;BAZ2B;SYNE2;RGPD4;SCAF8;ZNF407;NSD1;SAMD12;ZNF124;BPTF;MBD5;ARHGEF12;LRBA;VPS13B;ASH1L;PHC3;MYO9A;AKAP9;WDPCP;WDFY3;STRN;BIRC6;ZNF611;ZNF236;DOCK1;CSNK1G1
FRMPD3-AS1	36/100	0.0015793057335155385	ADCYAP1R1;GALNT13;LUZP2;CTNND2;NRXN1;ADAM22;GRIK4;FMN2;GRIK2;NDRG2;TRIM9;CDH20;MAP2;DNER;LRIG1;NCAM1;KCNN3;LRRC4C;EPHB1;WASF3;GPR158;GRIA4;RFTN2;NTRK2;GRID2;DSCAM;CADM2;NTRK3;TMOD2;TCF12;LSAMP;ZEB1;APC;ADGRB3;SCG3;ASTN1
SLC6A1-AS1	36/100	0.0015793057335155385	CRB1;PID1;GALNT13;PLPPR1;PHLPP1;MEGF11;NRXN1;ADAM22;GRIK4;GRIK2;NDRG2;SLC6A1;TRIM9;MAP2;CDH20;DNER;TNR;NCAM1;WASF3;GRIA4;RFTN2;DSCAM;CADM2;NTRK3;TMOD2;TCF12;LSAMP;LHFPL3;ATP2B2;KLF15;SLC25A18;ZEB1;APC;ADGRB3;SMOC1;ASTN1
SHANK2-AS2	36/100	0.0015793057335155385	PATJ;INO80D;ANKRD36;KMT2C;GREB1L;ZBTB20;LDLRAD4;BAZ2B;DUX4;KIAA1328;ZNF407;NSD1;POTEC;ANKRD26;MBD5;ANKRD30B;ANKRD30A;LRBA;DNAH14;OR1L6;RC3H1;VPS13B;ASH1L;DEFB108B;PHC3;ZNF717;TTC6;TASOR2;ZNF678;BIRC6;BRWD1;ZNF236;KIAA0825;ANKRD36B;SHANK2;TNRC6B
OIP5-AS1	36/100	0.0015793057335155385	ZNF573;INO80D;CHD9;ZNF292;CELF2;IREB2;UBE3A;UBR1;PTAR1;PCNX1;BTAF1;HERC1;TRPM7;SCAI;S100PBP;BPTF;USP8;AQR;PRKCB;ERCC6L2;VPS13C;MGA;ATRX;RC3H1;INO80;FAM126B;MYO9A;RC3H2;PIAS1;CCDC88A;AGO3;KANSL1;RFX7;GNAQ;ZNF236;TNRC6B
CLSTN2-AS1	36/100	0.0015793057335155385	KMT2E;CLSTN2;ANKRD36;KMT2C;ITPR2;LDLRAD4;BAZ2B;KIAA1328;POTEJ;ZNF407;NSD1;CEP192;ANK

			S1B;ANKRD26;ANKRD30B;ANKRD30A;LRBA;MGA;DNAH14;VPS13B;ASH1L;DEFB108B;PHC3;PBX1;INPP4B;TTC6;TASOR2;ZNF678;BIRC6;UTRN;BRWD1;ZNF236;KIAA0825;ANKRD36B;SHANK2;TNRC6B
PPP1R12A-AS1	36/100	0.0015793057335155385	*RABGAP1;ROCK1;PPM1L;PRUNE2;RNF38;PRICKLE2;CACNA1C;UBR1;LPP;FYCO1;CALD1;HECTD4;SPOP;TSPAN2;PGM5;SCAPER;TEAD1;SVIL;MYOCD;ERCC6L2;FBXL17;FOXN3;STON1-GTF2A1L;EML1;PJA2;TOM1L2;CLIP1;SMTN;SETBP1;SLMAP;KCNMA1;CCSER2;PPP1R12B;ZMYND11;ASB2;VCL*
LINC00305	35/100	0.0029969704005812603	*ZNF397;ATP8A2;KDM1B;SYCP1;PIGN;DNAH5;PLD5;HTR2C;FHIT;ABCC12;ADAMTSL1;TTR;ZSCAN30;ERBB4;KIF13A;ZNF648;SLC39A6;RPRD1A;ZNF385D;PDE6A;TRPM3;OR4C46;NTNG1;WWOX;KCNH5;SLC13A4;GALNT1;ABCA4;ELP2;TRAPPC8;RAB27B;GABRG3;LMX1A;NAT1;GALNTL6*
LINC00839	35/100	0.0029969704005812603	*GALNT14;ENPEP;NLGN1;ANKRD33B;MAML2;PLPPR5;SLC35F1;SRGAP2C;MXI1;ENPP3;SOX6;DIP2C;SRGAP2;GPR156;SRGAP2B;ANKRD6;IKBIP;LINGO1;KIRREL1;EGLN3;VCAM1;KSR1;CD70;HPCAL1;KAZN;FRMD4A;HSPA12A;SORCS3;IGSF11;MYO3A;SPIRE1;ALPK2;RGL1;COPS8;CHST3*
LINC01344	35/100	0.0029969704005812603	*PATJ;GREB1L;DUX4;FAM214A;MIPOL1;CYP4Z1;HHAT;ODR4;ERBB4;TRPS1;ZNF648;PSD3;POTED;ST8SIA6;POTEC;RALGPS2;ANKRD26;ANKRD30A;DNAH14;COG2;MRTFB;TBC1D9;ASH1L;PRLR;ESR1;DCDC1;LRP1B;RABEP1;NAT1;MYO3B;TTC6;NEK10;SPOPL;ATF6;KIAA0825*
LINC01761	35/100	0.0029969704005812603	*KDM5A;CLSTN2;ANKRD36;KMT2C;ITPR2;LDLRAD4;KIAA1328;POTEJ;ZNF407;NSD1;HIVEP1;CEP192;ANKS1B;ANKRD26;ANKRD30B;ANKRD30A;MGA;DNAH14;VPS13B;ASH1L;DEFB108B;ESR1;PBX1;INPP4B;TTC6;TASOR2;ZNF678;BIRC6;UTRN;BRWD1;ZNF236;KIAA0825;ANKRD36B;SHANK2;TNRC6B*
LINC01741	35/100	0.0029969704005812603	*DGKG;CRB1;GALNT13;PID1;PLPPR1;DGKB;MEGF11;GRIK4;SLC35F1;GRIK2;TRIM9;MAP2;RASGEF1C;GDAP1L1;DNER;TNR;KIF21B;CSMD3;SOX6;LRRC4C;CACNG2;EPHB1;DLGAP2;GPR158;GRIA4;CA10;DSCAM;TCF12;LHFPL3;EPN2;FCHSD2;SMOC1;UST;TOX;FGF12*
LINC02115	35/100	0.0029969704005812603	*FTO;ACSS3;KCNE4;RAP1GDS1;GRIK1;LRP2;AFF3;FAM107B;CDH7;PAK1;DACH1;ZSWIM6;MALRD1;PRKACB;STK32B;EDIL3;PACRG;SIAH3;AMFR;CYBRD1;SCAMP1;ESR1;CDYL2;MED13L;MOB1B;CACNB2;TBC1D1;BCL2;CDHR3;BMP2K;ERP27;BMPR1B;ATP6V0D2;FGF12;FGF10*
LINC00499	35/100	0.0029969704005812603	*GRIA1;ADCYAP1R1;RTN1;HEPACAM;CTNND2;NRXN1;KLHL32;SLC1A2;AKAP6;FMN2;NDRG2;CDH20;FUT9;CHN1;DLGAP1;NCAM1;KCNN3;ERC2;WASF3;RFTN2;ARNT2;NTRK2;TMOD2;LSAMP;ANK2;SLC39A12;CORO2B;GABRG1;ETNPPL;TAFA5;NRG3;APC;PPP2R2B;APBA2;RAPGEF4*
GPC6-AS2	35/100	0.0029969704005812603	*ZNF891;NFAT5;INO80D;CUL5;ANKRD36;CHD9;ZNF292;ZBTB20;LPP;ACTR3C;PLCZ1;RNF152;GPC6;ZNF124;ANKRD36C;MBD5;STPG2;VPS13C;PDE4D;FNDC3B;RC3H1;LNPEP;PLEKHA3;PHC3;MOB1B;VMP1;ZNF717;AGO3;SYT10;WDPCP;ZNF780B;BRWD1;KIAA0825;DGKI;ANKRD36B*

AGBL4-IT1	35/100	0.0029969704005812603	*ZNF891;INO80D;ANKRD36;STXBP4;KMT2C;DNAH6;RGPD6;ZNF518A;RGPD5;EFCAB6;SLC9C1;PTAR1;ZNF407;ANKRD36C;ANKRD26;DNAH11;MON2;MBD5;ANKRD30A;VPS13C;DNAH14;RANBP17;OR1L6;RFX3;RC3H1;VPS13B;PLEKHA3;PHC3;DCDC1;ZNF717;ZNF678;WDPCP;BRWD1;KIAA0825;ANKRD36B*
KCNMA1-AS3	35/100	0.0029969704005812603	*INO80D;ANKRD36;KMT2C;GREB1L;ZBTB20;BAZ2B;EFCAB6;DUX4;ATXN1;KIAA1328;ZNF407;POTED;POTEC;ANKRD26;MBD5;MON2;RALGAPA1;ANKRD30A;LRBA;DNAH14;OR1L6;RC3H1;VPS13B;ASH1L;PHC3;DCDC1;ZNF717;TTC6;NEK10;BIRC6;BRWD1;ZNF236;KIAA0825;ANKRD36B;TNRC6B*
ARHGEF7-AS1	35/100	0.0029969704005812603	*MAML2;PPM1L;ATL1;NRXN1;ADAM22;FRY;NOL4;CTIF;DPP6;MAP2;NCAM1;DIP2C;MPDZ;WASF3;TRIM23;ZHX3;CADM2;TMOD2;TCF12;EBF1;KIAA0232;PJA2;DNM3;SGSM1;ZEB1;NBEA;SETBP1;APC;ADGRB3;WDFY3;TCF4;TLN2;ARHGEF7;CDS2;PAFAH1B1*
GLCCI1-DT	35/100	0.0029969704005812603	*STX12;DOCK3;ATP8A1;PPM1L;RNF38;PRTG;HIRA;DPP6;MAP2;EPC2;NCAM1;FYN;SCAPER;TRIM23;NDFIP1;GRID1;ERCC6L2;TCF12;VPS13D;FBXL17;PJA2;ARHGAP31;TG;ZEB1;FCHSD2;PDP2;NBEA;SYNJ1;TBC1D5;UST;ELMO1;CCSER2;RAPGEF2;ZMYND11;PAFAH1B1*
ETV5-AS1	35/100	0.0029969704005812603	*NFAT5;INO80D;ANKRD36;CHD9;KMT2C;FMN1;PTPRJ;ASAP1;RGPD8;LYST;SDCBP;SPRED1;ZNF407;ZNF449;ABL2;ZNF106;HMCN1;DISC1;EVI5;ANKRD36C;MBD5;MON2;MYEF2;ERCC6L2;VPS13C;SETDB2;MYO5A;VPS13B;MITF;FER;WDPCP;BIRC6;ZNF780B;ZNF236;ANKRD36B*
PEX5L-AS2	35/100	0.0029969704005812603	*MARCHF1;ANKRD36;CCDC126;GREB1L;EFCAB6;LRP2;NLK;DUX4;RIMS1;KIAA1328;HERC1;ERBB4;ZNF407;PSD3;POTED;HYDIN;TOGARAM1;ZNF385D;POTEC;ANKS1B;AUTS2;ANKRD30A;DNAH14;SORCS1;ASH1L;DCDC1;ARHGAP32;RABEP1;MYO3B;TTC6;NOS1AP;NEK10;ZNF236;KIAA0825;ANKRD36B*
EDIL3-DT	35/100	0.0029969704005812603	*NRP1;PRUNE2;PRICKLE2;LOXL2;MPRIP;CALD1;CHSY3;DIP2C;EDIL3;GPC6;ARSB;PRKG1;IKBIP;MYOCD;PDE4D;TPM1;AFAP1;EML1;PTPRD;TTLL7;VCAN;COL4A2;SLMAP;EOGT;CDH11;ITGA8;ULK2;CDH13;ROR1;ESYT2;FAT4;RAPGEF5;PPP1R12B;ASB2;VCL*
MAGI2-AS1	35/100	0.0029969704005812603	*ZNF891;NFAT5;INO80D;ANKRD36;CHD9;KMT2C;RGPD6;ZBTB20;ZDHHC21;SLC9C1;LPP;PTAR1;ATXN1;ZNF124;ANKRD36C;MBD5;MON2;RALGAPA1;ERCC6L2;VPS13C;OR1L6;RC3H1;VPS13B;LNPEP;PLEKHA3;HOOK3;PHC3;VMP1;FER;ZNF717;AGO3;ZNF780B;BRWD1;KIAA0825;ANKRD36B*
RERG-IT1	35/100	0.0029969704005812603	*STAU2;AFF3;FAM214A;RERG;HECTD2;TRPS1;RB1CC1;FAM241A;POTED;APBB2;NBN;PCMTD1;VAV3;ARFGEF1;LYPLA1;CERS6;ANKRD30A;LRBA;MRTFB;TBC1D9;ELP2;GFRA1;PRLR;ESR1;PBX1;INPP4B;RABEP1;TSPAN13;NDUFAF6;TTC6;BCL2;NEK10;SPOPL;LMX1B;FSIP1*
RORA-AS2	35/100	0.0029969704005812603	*PATJ;ANKRD36;KMT2C;GREB1L;RGPD6;ZBTB20;RORA;DUX4;KIAA1328;ZNF407;POTED;POTEG;ANKRD26;MBD5;MON2;ANKRD30A;ERCC6L2;DNAH14;OR1L6;RC3H1;VPS13B;ASH1L;PHC3;LRP1B;ZNF717;SETBP1;TTC6;NFIA;NEK10;BIRC6;BRWD1;ZNF236;KIAA0825;ANKRD36B;TNRC6B*
FBXW7-	35/100	0.0029969704	*MYT1L;FRMPD4;DIRAS2;HTR2A;FBXO41;KIAA0513;*

AS1		005812603	*AKAP11;HERC1;SV2B;CHN1;TRAPPC11;ERC2;SCAPER;CACNG3;RBFOX1;MEF2C;SYT1;SGTB;PRKCB;ERCC6L2;SLC4A10;GRIN2B;SYN2;C1QL3;GABRG2;SNAP91;PJA2;CNKSR2;SYNJ1;APC;SCN8A;RAPGEF2;SCN2A;PAFAH1B1;RAPGEF4*
TSC22D1-AS1	35/100	0.0029969704005812603	*RERE;HDAC4;CRACD;ADAM22;AGAP1;NALCN;FBXO41;UNC80;HERC2;AKAP11;MAP2;HECTD4;LRIG1;DIP2A;DLGAP1;NPIPA1;NCAM1;SRGAP3;RALGPS1;ATP9A;MBD5;PTPRN2;CADM2;CADPS;LSAMP;PRKCA;SNAP91;DNM3;SYNJ1;TAOK3;ADGRB3;TBC1D5;MADD;CNTN3;ASTN1*
LINC02085	34/100	0.005752228436968856	*WDR26;SLC1A1;SLC40A1;BZW1;FAM214A;RERG;MIPOL1;CYP4Z1;HHAT;FUT8;PRRC1;ZNHIT6;FAM241A;SLC39A6;IARS2;PRKACB;RALGPS2;CERS6;TBC1D9;ELP2;TC2N;ESR1;PBX1;SRP9;FAM135B;RABEP1;NAT1;SPOPL;RAB3GAP2;INTS7;LMX1B;BMPR1B;FSIP1;SEC24D*
LINC01567	34/100	0.005752228436968856	*NFAT5;MARCHF1;CUL5;PPM1L;ZNF292;RGPD6;ZBTB20;RGPD5;CDH7;HS6ST3;AP5M1;KIAA1328;PLCZ1;TRIM2;THSD7A;RNF152;ZNF124;UNC13C;ANKRD36C;MBD5;STPG2;ZNF382;RALGAPA1;PDE4D;ADAM32;PLEKHA3;MOB1B;ZNF717;SYT10;LRRC9;ZNF780B;DGKI;CNTNAP5;ZNF431*
LINC00457	34/100	0.005752228436968856	*RYR2;CCDC186;KMT2C;RGPD6;ZBTB20;NEDD4L;LCLAT1;ACACA;POTEM;POTEH;SNX25;POTEG;VSTM4;ZFHX3;ST6GAL2;SLC15A2;LIMCH1;RALGAPA1;RALGAPA2;MAGI3;LRBA;PBX3;ARHGAP28;KCNAB1;ASH1L;PHC3;FRMD3;NBEA;TTC6;NFIA;PRR16;UTRN;DGKI;XKR6*
LINC00710	34/100	0.005752228436968856	*MACF1;NFAT5;INO80D;ANKRD36;CHD9;KMT2C;ZBTB20;RORA;ZDHHC21;BAZ2B;SLC9C1;LPP;SYNE1;ATXN1;MACROD2;EVI5;ANKRD36C;DNAH11;MBD5;MON2;ERCC6L2;VPS13C;ATRX;OR1L6;RC3H1;VPS13B;PLEKHA3;PHC3;AGO3;FAT4;BRWD1;KIAA0825;ANKRD36B;TNRC6B*
LINC00362	34/100	0.005752228436968856	*PATJ;KCNE4;PRDM15;TULP4;LDLRAD3;LDLRAD4;FAM214A;TRPS1;GAST;APBB2;PRKACB;CPA6;SGCG;RALGPS2;LRBA;VPS13B;LNPEP;GFRA1;ESR1;PBX1;MED13L;VMP1;SPATA17;RABEP1;TTC6;BCL2;BMP2K;SPOPL;ZNF678;BMPR1B;BRWD1;FSIP1;FGF12;FBXL7*
LINC01677	34/100	0.005752228436968856	*DGKG;CRB1;GALNT13;RTN1;HEPACAM;MEGF11;CTNND2;KLHL32;ADAM22;GRIK4;GRIK2;NDRG2;TRIM9;CDH20;MAP2;GDAP1L1;DNER;PHACTR3;TNR;NCAM1;EPHB1;GRIA4;RFTN2;NTRK2;GRID2;DSCAM;CADM2;LSAMP;ATAT1;SLC25A18;ADGRB3;SMOC1;SCG3;APBA2*
LINC01069	34/100	0.005752228436968856	*SHC4;NLGN1;PHLPP1;ABCB5;AKAP6;SDCBP;DSTYK;UBL3;SGCD;CHCHD6;MXI1;RGS20;RTTN;PHACTR1;PRAME;ZNF106;HMCN1;STK32A;SOX6;PKNOX2;SOX5;BCAS3;MYEF2;MYO10;MICAL3;MYO5A;CABLES1;MITF;NSG1;CORO2B;IGSF11;NRG3;SPIRE1;MDGA2*
LINC01238	34/100	0.005752228436968856	*PTPRT;USP14;GREB1L;DUX4;FAM214A;TMEM25;CYP4Z1;TRPS1;PSD3;FAM241A;POTED;SLC39A6;ST8SIA6;ZNF385D;POTEC;RALGPS2;KDM4B;AUTS2;ANKRD30A;MRTFB;COG2;TBC1D9;ELP2;GFRA1;ASH1L;ESR1;DCDC1;RABEP1;NAT1;TTC6;NEK10;SPOPL;LMX1B;BMPR1B*
LINC02224	34/100	0.005752228436968856	*PTPRT;USP14;CLSTN2;KCNE4;LRP2;AFF3;BZW1;TMEM241;RERG;TMEM25;DACH1;TRPS1;FAM241A;SLC3*

			9A6;MALRD1;PRKACB;RALGPS2;CERS6;CYBRD1;TBC1D9;ELP2;GFRA1;ESR1;PBX1;MED13L;RABEP1;TSPAN13;NAT1;KIF16B;BCL2;LMX1B;BMPR1B;FSIP1;FGF10
LINC02598	34/100	0.005752228436968856	*CRB1;GALNT13;PHLPP1;PLPPR1;LUZP2;CTNND2;PCDH15;ADAM22;GRIK4;GRIK2;NDRG2;IQCJ-SCHIP1;TRIM9;MAP2;SNTG1;CDH20;NCAM1;KIF21B;KCNN3;CSMD2;EPHB1;GRIA4;RFTN2;NTRK2;GRID2;DSCAM;KCND2;TCF12;LSAMP;LHFPL3;ATAT1;FCHSD2;ZEB1;APBA2*
LINC00517	34/100	0.005752228436968856	*PATJ;ANKRD36;KMT2C;GREB1L;ZBTB20;LPP;POTEM;ATXN1;KIAA1328;POTEH;POTEJ;ZNF407;POTED;POTEG;ANKRD26;MBD5;ANKRD30B;ANKRD30A;ERCC6L2;DNAH14;OR1L6;VPS13B;ASH1L;DEFB108B;PHC3;ZNF717;TTC6;NFIA;UTRN;BRWD1;ZNF236;KIAA0825;ANKRD36B;TNRC6B*
RAMP2-AS1	34/100	0.005752228436968856	*PTPRT;GALNT13;CLSTN2;ADAM22;GRIK4;LDLRAD3;LDLRAD4;GLI3;SCAF8;CDH20;TRPS1;LRIG1;APBB2;MPDZ;WASF3;TRIM23;RFTN2;ARNT2;ANKRD26;MAGI2;EBF1;GFRA1;ESR1;ZDHHC17;PBX1;TNRC6C;ARHGAP31;RABEP1;ZEB1;PTPRA;TTC3;PWWP3A;ULK2;FBXL7*
GCC2-AS1	34/100	0.005752228436968856	*RBM47;PRKAA1;CCDC186;RGPD5;RGPD8;PRDM10;TM9SF3;DCAF1;DTWD2;MFSD9;UFL1;AUH;MAP7;RHPN2;SEC23B;PLS1;RANBP2;SLC37A1;BICRAL;RALGAPA2;DENND4C;SHROOM3;ELOVL7;CD2AP;ZNF33B;TM9SF2;RSPH3;NCOR1;DOP1B;MYO5B;SHLD2;MYO5C;CMPK1;ANAPC1*
TPRG1-AS2	34/100	0.005752228436968856	*PTPRT;MAST4;KCNE4;CCDC126;GREB1L;LDLRAD3;FAM214A;TMEM241;TMEM25;TRPS1;FAM241A;POTED;SLC39A6;ST8SIA6;RALGPS2;VAV3;KDM4B;SIAH2;COG2;CYBRD1;TBC1D9;ELP2;GFRA1;PRLR;ESR1;DCDC1;RABEP1;NAT1;KIF16B;NEK10;SPOPL;LMX1B;BMPR1B;FSIP1*
LRRC8C-DT	34/100	0.005752228436968856	*ZNF573;MACF1;PHLPP1;CHD9;CELF2;LYST;DOCK10;SRGAP2C;HERC2;HERC1;MAN2A2;SACS;ANKFY1;DIP2B;LRRC8B;DISC1;SRGAP2B;ANKRD28;USP24;SGTB;PRKCB;ERCC6L2;YLPM1;MYO5A;IGSF11;CCDC88A;NIN;SYNJ1;AGO3;APC;TAOK3;RFX7;WDPCP;TNRC6B*
SMAD9-IT1	34/100	0.005752228436968856	*KMT2E;RABGAP1;ATP8A1;MAML2;ADAM22;ZBTB20;SYNE1;POTEM;AKAP11;POTEH;HECTD4;SCAPER;MPDZ;ZNF462;RIC3;MBD5;TMEM178B;ZBTB16;VPS13D;YLPM1;ZDHHC17;MYO9A;PYGO1;TJP1;ZEB1;SETBP1;NFIA;PEAK1;RUFY2;CCSER2;RAPGEF2;WDFY3;UTRN;ZMYND11*
MKLN1-AS	34/100	0.005752228436968856	*MTMR10;NLGN1;PLEKHB2;ZBTB20;PTPRJ;SLC4A4;CDC14B;ARHGAP12;BCL2L13;IMMP2L;RANBP3L;UBL3;TYW1;SOX6;BBS2;ZHX3;MYEF2;PNPLA8;SLC2A13;PRKCA;ARHGAP24;GPR137B;AIF1L;PARD3B;IGSF11;HADHB;CLCN5;TBC1D5;VPS41;EXOC4;SPIRE1;WDFY3;PRKD1;CDS2*
WWTR1-IT1	34/100	0.005752228436968856	*KMT2E;INO80D;ANKRD36;KMT2C;TSHZ2;RGPD6;ZBTB20;RGPD5;RGPD8;LPP;ATXN1;KIAA1328;ZNF407;ZNF124;ANKRD36C;ZNF462;ZFHX3;MBD5;MGA;OR1L6;RC3H1;VPS13B;ASH1L;PHC3;VMP1;ZNF717;AGO3;WDPCP;BIRC6;FAT4;ZNF236;KIAA0825;ANKRD36B;TNRC6B*
PKIA-	34/100	0.0057522284	*ZNF891;NFAT5;ACSS3;CUL5;PPM1L;RGPD6;ZBTB20*

AS1		36968856	*;RGPD5;ZDHHC21;CDH7;HS6ST3;AP5M1;KIAA1328;PLCZ1;THSD7A;RNF152;UNC13C;MBD5;STPG2;ABCA5;RALGAPA1;ERCC6L2;PDE4D;PHC3;MOB1B;INPP4B;ZNF717;SYT10;LRRC9;BRWD1;ATP6V0D2;DGKI;CNTNAP5;ZNF431*
NAV2-AS3	34/100	0.005752228436968856	*NFAT5;RGPD6;ZBTB20;RGPD5;ZDHHC21;SLC9C1;SYNE1;ACTR3C;AP5M1;KIAA1328;TRIM2;KIF21A;THSD7A;SHOC1;SCAPER;ANKRD36C;MBD5;MON2;ARHGEF12;RALGAPA1;ERCC6L2;VPS13C;FBXL17;UBE2QL1;PLEKHA3;MOB1B;TRAPPC6B;ZNF678;ZNF780B;BRWD1;PTPN4;ATP6V0D2;KIAA0825;CPEB4*
DLG2-AS2	34/100	0.005752228436968856	*RYR2;ZNF891;KMT2C;OR4K2;RGPD6;ZBTB20;RGPD5;ZDHHC21;RGPD8;LPP;RGPD4;POTEM;ATXN1;POTEH;TMEM225;POTEG;ANKRD36C;ZFHX3;MBD5;RALGAPA1;ERCC6L2;PLEKHA3;ASH1L;PHC3;PYGO1;GOLGA6C;DLG2;ZNF717;NFIA;PRR16;ZNF780B;UTRN;BRWD1;PTPN4*
LINC01602	33/100	0.010877980802436248	*CRB1;GALNT13;PID1;DGKB;GRIK4;GRIK2;UNC80;TRIM9;MAP2;DNER;GDAP1L1;PHACTR3;TNR;FYN;KIF21B;CSMD3;SOX6;LRRC4C;CACNG2;EPHB1;GPR158;GRIA4;CA10;DSCAM;TCF12;LHFPL3;FCHSD2;SMOC1;UST;SCG3;TOX;MDGA2;FGF12*
LINC02679	33/100	0.010877980802436248	*SLC44A5;ZNF891;KHDRBS2;ATP8A1;PELI2;ZDHHC21;NYAP2;PPP1R9A;MTMR7;MIPOL1;KIAA1328;ZSCAN30;TOGARAM1;SLC15A5;ZNF124;MBD5;WSB1;FANCM;RALGAPA1;LIMCH1;VPS13C;DNAH14;POU6F2;HOOK3;ASH1L;DDHD1;FOXP2;HIPK3;ZNF717;ZNF678;BRWD1;ZNF236;FRA10AC1*
LINC00446	33/100	0.010877980802436248	*SHC4;PHLPP1;ABCB5;FMN1;AKAP6;TRPM1;SYNPR;SDCBP;UBL3;SGCD;SCFD2;CHCHD6;RGS20;RTTN;PHACTR1;PRAME;ZNF106;STK32A;SNX8;PKNOX2;SOX5;MYEF2;MYO10;MOK;MYO5A;CABLES1;MITF;NSG1;GPR137B;IGSF11;NRG3;RAB38;RGS12*
LINC01739	33/100	0.010877980802436248	*SHC4;IGSF3;ABCB5;FMN1;PTPRJ;LYST;SDCBP;DSTYK;SGCD;UBL3;SCFD2;CHCHD6;PACSIN2;ABL2;RGS20;PHACTR1;PRAME;ZNF106;HMCN1;STK32A;ARHGEF11;MYEF2;MYO10;MOK;MYO5A;CABLES1;MITF;NSG1;PARVB;S100B;IGSF11;FAM167B;ENTHD1*
LINC01640	33/100	0.010877980802436248	*KMT2E;PPM1L;PRICKLE2;NRXN3;CACNA1C;LPP;MED15;FYCO1;INPP5A;KIAA1328;PLCZ1;CALD1;ABL1;RBPMS2;PGM5;LARGE1;PRKG1;RALGPS1;MAGI1;ZFHX3;CBLIF;FOXN3;STON1-GTF2A1L;EML1;PARD3B;SMTN;SETBP1;ASXL3;SLMAP;KCNMA1;ZNF74;CCSER2;TTLL11*
LINC02676	33/100	0.010877980802436248	*ZNF891;PATJ;ANKRD36;TULP4;GXYLT2;FAM214A;MIPOL1;HECTD2;POTEJ;TRPS1;PSD3;POTED;POTEG;MBD5;MON2;ADGRV1;ANKRD30A;CFAP70;LRBA;DNAH14;OR1L6;PLXDC2;LRP1B;VMP1;ZNF717;TTC6;AKAP9;NEK10;SPOPL;ZNF678;BRWD1;KIAA0825;ANKRD36B*
LINC02151	33/100	0.010877980802436248	*GALNT13;PLPPR1;MEGF11;ADAM22;GRIK4;GRIK2;UNC80;DPP6;SNTG1;MAP2;RASGEF1C;GDAP1L1;DNER;TNR;NCAM1;LRRC4C;CACNG2;GPR158;GRIA4;LINGO1;CA10;DSCAM;LHFPL3;SEZ6L;MYT1;EPN2;SLC8A3;ATAT1;NSG2;ADGRB3;SMOC1;UNC79;ASTN1*
LINC02556	33/100	0.010877980802436248	*DGKG;GALNT13;MTMR3;PHLPP1;MEGF11;CELF2;TUT4;ADAM22;GRIK4;GRIK2;CABIN1;CDH20;MAP2;TNR;NCAM1;SRGAP3;SCAPER;GRIA4;RFTN2;SPEN;USP49;DSCAM;ZBTB16;TCF12;YLPM1;BCR;CCDC88A;FCH*

			SD2;AGO3;RFX7;SMOC1;ELMO1;FAM193A
LINC02199	33/100	0.010877980802436248	*SHC4;PHLPP1;FMN1;SDCBP;DSTYK;UBL3;SCFD2;CHCHD6;GNG7;ABL2;PHACTR1;PRAME;ZNF106;HMCN1;SRGAP2;DISC1;SOX5;PKNOX2;ARNT2;MYEF2;MYO10;ST8SIA1;MICAL3;MOK;MYO5A;MITF;NSG1;S100B;CORO2B;GPR137B;IGSF11;ITPKB;MDGA2*
LINC01490	33/100	0.010877980802436248	*NECAB1;PPM1L;PTPRQ;ITPR2;AFF3;HS6ST3;CDH7;PLCZ1;GSE1;THSD7A;KIAA0319L;RNF152;SLC15A5;VAV3;UNC13C;KDM4B;STPG2;CERS6;AUTS2;ATRNL1;AGL;VPS13B;SCAMP1;DCLK1;MOB1B;INPP4B;TSPAN13;SYT10;LRRC9;BMPR1B;FSIP1;DGKI;MBTPS2*
LINC01255	33/100	0.010877980802436248	*KMT2E;CLSTN2;ANKRD36;KMT2C;ITPR2;LDLRAD4;KIAA1328;POTEJ;ZNF407;NSD1;CEP192;ANKS1B;ANKRD26;ANKRD30B;ANKRD30A;LRBA;DNAH14;VPS13B;ASH1L;DEFB108B;ESR1;PBX1;INPP4B;TTC6;TASOR2;BIRC6;UTRN;BRWD1;ZNF236;KIAA0825;ANKRD36B;SHANK2;TNRC6B*
LINC02334	33/100	0.010877980802436248	*ZNF891;NFAT5;INO80D;CUL5;ANKRD36;CHD9;ZNF292;RGPD6;ZBTB20;RGPD5;PTAR1;ACTR3C;PLCZ1;RNF152;ZNF124;ANKRD36C;MBD5;MON2;STPG2;ERCC6L2;VPS13C;PDE4D;RC3H1;LNPEP;PLEKHA3;PHC3;MOB1B;ZNF717;AGO3;SYT10;DGKI;ANKRD36B;TNRC6B*
RBMS3-AS2	33/100	0.010877980802436248	*ZNF891;NFAT5;INO80D;ANKRD36;CHD9;KMT2C;RGPD6;ZBTB20;ZDHHC21;SLC9C1;LPP;ATXN1;ZNF407;ANKRD36C;DNAH11;MBD5;MON2;VPS13C;OR1L6;RC3H1;VPS13B;PLEKHA3;PHC3;VMP1;ZNF717;AGO3;WDPCP;ZNF780B;BIRC6;FAT4;BRWD1;KIAA0825;ANKRD36B*
MAPRE3-AS1	33/100	0.010877980802436248	*NFAT5;INO80D;MARCHF1;CUL5;PPM1L;ANKRD36;KMT2C;RGPD6;ZBTB20;RGPD5;SLC9C1;DUX4;ACTR3C;KIAA1328;PLCZ1;THSD7A;RNF152;MBD5;STPG2;ERCC6L2;OR1L6;RC3H1;VPS13B;ASH1L;PHC3;MOB1B;ZNF717;SYT10;BIRC6;ZNF236;DGKI;ANKRD36B;TNRC6B*
ZMYM4-AS1	33/100	0.010877980802436248	*ZNF573;ANKRD17;INO80D;CHD9;ZNF292;KMT2C;IREB2;LTN1;RGPD8;UBR1;RGPD4;ZMYM1;NIPBL;ZMYM4;NSD1;TRAPPC11;CEP192;ZNF124;BPTF;USP24;FANCM;ERCC6L2;VPS13C;MGA;ATRX;RC3H1;VPS13B;ASH1L;RC3H2;AGO3;RFX7;BIRC6;CSNK1G1*
MFF-DT	33/100	0.010877980802436248	*MTMR10;NLGN1;PPM1L;ADAM22;ZBTB20;MSANTD4;PPP1R9A;UBL3;THSD7A;SOX6;TRIM23;BBS2;RFTN2;ARNT2;MBD5;NDFIP1;ZHX3;PNPLA8;SLC2A13;MAGI2;FBXL17;ANK2;ZDHHC17;PJA2;MOB1B;PDP2;TBC1D5;RRAGD;VPS41;SPIRE1;WDFY3;COPS8;CPEB4*
DENND6A-AS1	33/100	0.010877980802436248	*ZNF573;ANKRD17;MACF1;SETD2;ROCK1;CHD9;IREB2;LTN1;PRDM10;DCAF1;PCNX1;TRAPPC11;BPTF;USP24;USP8;MORC3;ERCC6L2;DENND4C;ATRX;RC3H1;VPS13B;ASH1L;GAPVD1;HIPK1;ARID1B;RC3H2;MED13L;NIN;ELF2;RFX7;BIRC6;ZNF236;CSNK1G1*
NR2F2-AS1	33/100	0.010877980802436248	*NRP1;NFAT5;GALNT14;ENPEP;FLT1;PRKAA2;STXBP4;ARHGEF28;ZBTB20;LDB2;SLC6A3;CNDP2;TTC28;ARHGAP42;EPB41L4A;SNX29;GRB10;ARSB;MBD5;VCAM1;INSR;ITGA1;CRIM1;PPP2R3A;MYO9A;ARHGAP24;SNRK;CLCN5;WDFY3;ALPK2;FAT4;ZNF611;TRABD2B*
MAPK10-AS1	33/100	0.010877980802436248	*NFAT5;KMT2C;RGPD6;ZBTB20;RGPD5;ZNF66;RGPD8;BAZ2B;LIMD1;LPP;SYNE1;POTEM;POTEH;ZNF407;POTEG;ANKRD36C;ZNF462;ZFHX3;MBD5;ERCC6L2;M*

			GA;OR1L6;VPS13B;PHC3;SMAD5;IQCM;ZNF717;WDFY3;CCSER1;BIRC6;UTRN;ANKRD36B;TNRC6B
NAV2-AS1	33/100	0.010877980802436248	*DOCK3;DOCK9;ZFYVE1;HIRA;UBL3;SNX25;BBS9;STK32A;VSTM4;TEAD1;RXRG;PKNOX2;CTSB;EPHA4;TRPC5;MYEF2;CADM1;MYO10;DENND2B;SAMD4A;FAM189A2;CABLES1;MITF;NAV2;FRMD3;ITPKB;TANC1;TG;TCERG1L;IFT81;SLC27A6;ST6GALNAC3;ITGA9*
NAV2-AS5	33/100	0.010877980802436248	*MACF1;NFAT5;INO80D;ANKRD36;CHD9;KMT2C;ZBTB20;RORA;ZDHHC21;BAZ2B;SLC9C1;LPP;RPS6KA3;ACTR3C;ATXN1;EVI5;ANKRD36C;DNAH11;MBD5;MON2;ERCC6L2;VPS13C;OR1L6;RC3H1;PLEKHA3;PHC3;VMP1;FER;AGO3;BRWD1;KIAA0825;ANKRD36B;TNRC6B*
TSPAN9-IT1	33/100	0.010877980802436248	*ZNF891;NFAT5;INO80D;CUL5;ANKRD36;KMT2C;RGPD6;ZBTB20;ACTR3C;KIAA1328;PLCZ1;ZNF407;RNF152;MBD5;MON2;STPG2;RALGAPA1;ERCC6L2;PDE4D;OR1L6;RC3H1;VPS13B;LNPEP;PHC3;MYO9A;MOB1B;ZNF717;SYT10;BRWD1;KIAA0825;DGKI;ANKRD36B;TNRC6B*
TPM1-AS	33/100	0.010877980802436248	*PATJ;INO80D;ANKRD36;KMT2C;GREB1L;RGPD6;ZBTB20;RGPD5;RGPD8;DUX4;LPP;ATXN1;KIAA1328;ZNF407;POTED;POTEC;MBD5;ANKRD30A;DNAH14;OR1L6;RC3H1;VPS13B;ASH1L;PHC3;DCDC1;ZNF717;TTC6;NEK10;BIRC6;ZNF236;KIAA0825;ANKRD36B;TNRC6B*
LINC01208	32/100	0.019239054039447853	*PTPRT;KCNE4;SLC1A1;SLC40A1;ICA1;GREB1L;PIK3R3;LDLRAD3;FAM214A;TMEM241;ABCC12;TMEM25;ADAM29;TRPS1;PRKACB;KDM4B;SIAH2;TBC1D9;TC2N;ESR1;NAALADL2;SRP9;AIMP1;RABEP1;TSPAN13;NAT1;TTC6;KIF16B;SPOPL;LMX1B;ADSS2;FSIP1*
LINC01674	32/100	0.019239054039447853	*CCDC126;AFF3;FAM214A;TMEM241;TMEM25;TRPS1;FAM241A;POTED;SLC39A6;POTEB;POTEC;RALGPS2;CERS6;POTEB2;POTEB3;COG2;MRTFB;TBC1D9;ELP2;GFRA1;TC2N;PRLR;ESR1;RABEP1;TSPAN13;NAT1;TTC6;NEK10;SPOPL;LMX1B;DHX29;BMPR1B*
LINC01209	32/100	0.019239054039447853	*KMT2E;ZNF891;ANKRD36;KMT2C;ZBTB20;ZDHHC21;LPP;ACTR3C;ATXN1;ZNF124;ANKRD36C;MBD5;RALGAPA1;DNAH14;ATRX;RC3H1;VPS13B;PLEKHA3;HOOK3;ASH1L;PHC3;PHF20L1;VMP1;NUP210L;AGO3;AGO2;ZNF678;WDPCP;CCSER1;BIRC6;KIAA0825;ANKRD36B*
LINC00387	32/100	0.019239054039447853	*PTPRT;TMEM63C;GREB1L;LDLRAD4;FAM214A;TMEM25;HHAT;TRPS1;PSD3;FAM241A;POTED;SLC39A6;ST8SIA6;APBB2;PCMTD1;VAV3;KDM4B;CERS6;NEBL;SIAH2;MRTFB;TBC1D9;ELP2;GFRA1;PRLR;ESR1;PBX1;NELL2;RABEP1;NAT1;SPOPL;LMX1B*
LINC02006	32/100	0.019239054039447853	*NFAT5;ACSS3;CUL5;PPM1L;ABCA13;CDH7;HS6ST3;AP5M1;KIAA1328;PLCZ1;TRIM2;ZNF208;KIF21A;THSD7A;SAMD12;RNF152;UNC13C;STPG2;ABCA5;PDE4D;SCAMP1;MOB1B;MAB21L3;INPP4B;TMEM116;SYT10;LRRC9;ERP27;ATP6V0D2;DGKI;CNTNAP5;ZNF431*
LINC02017	32/100	0.019239054039447853	*ADCYAP1R1;GALNT13;DIPK1A;RTN1;HEPACAM;LUZP2;CTNND2;KLHL32;PCDH15;GRIK4;FMN2;NDRG2;SLC25A48;CDH20;FUT9;NCAM1;KCNN3;CSMD2;WASF3;RFTN2;NTRK2;SLC15A2;KCND2;CADM2;TMOD2;PDE4DIP;ETNPPL;FRMD5;TAFA5;SMOC1;APBA2;ASB3*
LINC01088	32/100	0.019239054039447853	*ADCYAP1R1;GALNT13;HPSE2;HEPACAM;LUZP2;CTNND2;KLHL32;GRIK4;FMN2;NDRG2;CDH20;FUT9;NCAM*

			1;KCNN3;WASF3;JAM2;RFTN2;SLC14A1;NTRK2;SLC15A2;DTNA;ZHX3;LSAMP;PDE4DIP;GABRG1;ETNPPL;FRMD5;SLC25A18;PPP2R2B;CNTN1;CPE;ASB3
LINC01016	32/100	0.019239054039447853	*CLIC6;PARN;AFF3;FAM214A;TMEM241;RERG;GLI3;TMEM25;TRPS1;FAM241A;NRIP1;ST8SIA6;FLNB;RALGPS2;VAV3;KDM4B;MAGI3;SIAH2;GREB1;MRTFB;TBC1D9;GFRA1;PRLR;ESR1;INPP4B;RABEP1;TSPAN13;NEK10;SPOPL;LMX1B;BMPR1B;FSIP1*
LINC00564	32/100	0.019239054039447853	*KCNE4;PTPRQ;ITPR2;SIPA1L2;AFF3;PRSS23;TMEM25;ALCAM;ZNRF3;ERBB4;TRPS1;ANKRD31;ENPP1;LARGE1;SLC37A2;ATRNL1;SIAH2;ELP2;PXDNL;ERLIN2;PRLR;GABRG2;PARP8;KITLG;SPATA17;CNKSR3;BMPR1B;VSTM2A;FSIP1;PDZRN3;FBXL7;FGF10*
LINC00836	32/100	0.019239054039447853	*ADCYAP1R1;GALNT13;PHLPP1;HEPACAM;LUZP2;CTNND2;KLHL32;PCDH15;GRIK4;FMN2;NDRG2;SLC25A48;CDH20;FUT9;NCAM1;KCNN3;WASF3;JAM2;GRIA4;RFTN2;NTRK2;DTNA;CADM2;TMOD2;LSAMP;PDE4DIP;ETNPPL;FRMD5;PPP2R2B;SMOC1;SCG3;ASB3*
WDFY3-AS2	32/100	0.019239054039447853	*ADCYAP1R1;MTMR10;NLGN1;CTNND2;KLHL32;ADAM22;GRIK4;FMN2;NDRG2;IQCJ-SCHIP1;CDH20;NCAM1;KCNN3;WASF3;JAM2;TRIM23;GRIA4;BBS2;RFTN2;NTRK2;DTNA;ZHX3;CADM2;TMOD2;MAGI2;ANK2;APC;ADGRB3;PPP2R2B;SPIRE1;CPE;WDFY3*
RORB-AS1	32/100	0.019239054039447853	*SLC23A2;ABCD2;RTN1;KCNC1;MYT1L;FRMPD4;ATL1;SLC1A2;RORB;KIAA0513;SV2B;NCS1;CHN1;SPOCK1;CTNNA2;PPFIA2;RGS7;RBFOX1;PDE2A;SLC4A10;SYT16;ATP2B2;SYN2;SNAP91;CNKSR2;EML6;TAFA4;DLG2;SCN8A;PRKD1;SCN2A;ASTN1*
DOCK4-AS1	32/100	0.019239054039447853	*ZNF891;NFAT5;INO80D;ANKRD36;KMT2C;RGPD6;ZBTB20;RGPD5;ZDHHC21;RGPD8;SLC9C1;ATXN1;ZNF407;ZNF124;ANKRD36C;MBD5;MON2;VPS13C;DNAH14;OR1L6;RC3H1;VPS13B;PLEKHA3;PHC3;VMP1;ZNF717;AGO3;WDPCP;BIRC6;BRWD1;KIAA0825;ANKRD36B*
ENTPD1-AS1	32/100	0.019239054039447853	*ITSN2;MACF1;NFAT5;INO80D;DOCK9;CHD9;KMT2C;BAZ2B;AKAP13;PCNX1;HERC1;VSTM4;SCAPER;ST6GAL2;ERCC6L2;VPS13C;ATRX;RC3H1;HOOK3;PHC3;ARID1B;PIAS1;FRMD3;MLLT10;TG;TCERG1L;AGO3;LPCAT2;BIRC6;ZNF431;ITGA9;TNRC6B*
NCKAP5-IT1	32/100	0.019239054039447853	*PATJ;ANKRD36;KMT2C;TULP4;SLC9C1;ATXN1;ZNF407;PSD3;POTED;ZNF124;ANKRD26;MBD5;MON2;ANKRD30A;DNAH14;OR1L6;RC3H1;VPS13B;ASH1L;PLXDC2;LRP1B;VMP1;TTC6;AKAP9;NEK10;SPOPL;ZNF678;WDPCP;BIRC6;BRWD1;KIAA0825;ANKRD36B*
NCKAP5-AS1	32/100	0.019239054039447853	*PATJ;INO80D;CATSPER2;ANKRD36;KMT2C;GREB1L;ZBTB20;DUX4;FBXL20;ATXN1;KIAA1328;ZNF407;POTEC;ANKRD26;MBD5;RALGAPA1;ANKRD30A;DNAH14;OR1L6;RC3H1;VPS13B;ASH1L;PHC3;ZNF717;TTC6;ZNF678;BIRC6;CDK12;BRWD1;ZNF236;KIAA0825;ANKRD36B*
PCDH9-AS4	32/100	0.019239054039447853	*DOCK4;INO80D;ANKRD36;KMT2C;RGPD6;ZBTB20;RGPD5;ZNF518A;ZDHHC21;RGPD8;BAZ2B;SYNE1;ATXN1;ZNF124;ANKRD36C;MBD5;PCDH9;RALGAPA1;ANKRD30A;RC3H1;VPS13B;PLEKHA3;LRP1B;DNM3;VMP1;AGO3;AGO2;WDPCP;ZNF678;BIRC6;DOCK1;ANKRD36B*
PABPC5-AS1	32/100	0.019239054039447853	*SLC23A2;ATL1;ADAM22;NALCN;UNC80;CTIF;DPP6;DSTYK;GNG2;ZNF704;AKT3;NCAM1;DIP2C;LRRC4C;*

			MPDZ;GPR158;TRIM23;GARNL3;JAZF1;ZNF287;ANKRD26;MYEF2;ANKRD30B;LSAMP;PJA2;PYGO1;DNM3;NBEA;MMP16;ADGRB3;TTC3;CDS2
RBMS3-AS1	32/100	0.019239054039447853	*NFAT5;INO80D;ANKRD36;CHD9;KMT2C;ZBTB20;ZDHHC21;SLC9C1;PTAR1;ATXN1;ZNF407;ANKRD36C;DNAH11;MBD5;MON2;ERCC6L2;VPS13C;OR1L6;RC3H1;VPS13B;PLEKHA3;PHC3;VMP1;FER;AGO3;BIRC6;ZNF780B;FAT4;BRWD1;KIAA0825;ANKRD36B;RBMS3*
GPC5-IT1	32/100	0.019239054039447853	*ZNF518A;RORB;MSI2;BAZ2B;ANKRD20A1;CDH9;KALRN;SYNE1;ADAM28;CAPN5;NRIP1;GPC5;HSF5;CTSE;RGS6;SCAI;ZNF124;BPTF;MEF2C;POU1F1;KLF12;MBD5;XRCC4;ITGA4;RC3H1;SLC6A11;FAM126B;FER1L6;GNAQ;CLDN18;MDGA2;TNRC6B*
CREB3L2-AS1	32/100	0.019239054039447853	*ZNF891;NFAT5;INO80D;ANKRD36;KMT2C;ZBTB20;ZDHHC21;SLC9C1;LPP;ATXN1;ZNF124;ANKRD36C;MBD5;MON2;RALGAPA1;VPS13C;DNAH14;OR1L6;RC3H1;VPS13B;PLEKHA3;PHC3;VMP1;ZNF717;AGO3;ZNF678;WDPCP;BIRC6;BRWD1;KIAA0825;ANKRD36B;TNRC6B*
CNTN4-AS1	32/100	0.019239054039447853	*APP;STX12;ATP8A1;CPQ;DOCK9;COL14A1;SDC2;PRICKLE2;LAMC1;PTPRG;ABL1;BBS9;EPC2;SLIT3;PTCHD4;VSTM4;STARD13;ARHGEF12;CNTN6;LAMB1;PJA2;PARD3B;ADCY9;TBC1D5;SLC27A6;CNTN4;UTRN;ZMYND11;DOCK1;JCAD;RBMS3;ITGA9*
C3orf67-AS1	32/100	0.019239054039447853	*MTPN;UHRF1BP1L;DOCK4;KCNC1;MYT1L;RAP1GDS1;SLC9C1;EHBP1;ATXN1;AKAP11;ANKRD31;TRIM23;TANGO6;ANKRD26;MBD5;PTCD2;SYT1;SGTB;ATRNL1;SLC2A13;CADPS;KIAA1549L;ATP2B2;GRIN2B;SYN2;GABRG2;MOSMO;FER;SYNJ1;PPP2R2C;SCN8A;MBTPS2*
ZFAT-AS1	32/100	0.019239054039447853	*ZNF573;ANKRD17;ASAP1;ZFYVE26;KIAA1328;ZNF407;NSD1;TOGARAM1;NBN;ATP9B;USP24;CUBN;MBD5;FANCM;YTHDF3;ERCC6L2;MGA;TRAPPC10;VPS13B;DNAJC13;ASH1L;MYO9A;HIPK3;PHF20L1;AGO2;GEMIN5;RAB3GAP2;WDPCP;BIRC6;ZNF611;ZNF236;CSNK1G1*
MAST4-IT1	32/100	0.019239054039447853	*ZNF891;INO80D;ANKRD36;KMT2C;ZBTB20;ZDHHC21;SLC9C1;FAM214A;ATXN1;DNAH11;MBD5;MON2;RALGAPA1;ANKRD30A;VPS13C;DNAH14;MUC19;OR1L6;RC3H1;VPS13B;LNPEP;PHC3;LRP1B;VMP1;ZNF717;AGO3;TTC6;ZNF678;ZNF780B;BRWD1;KIAA0825;ANKRD36B*
EGFLAM-AS3	32/100	0.019239054039447853	*ZNF891;NFAT5;INO80D;ANKRD36;KMT2C;RGPD6;TULP4;RGPD5;SLC9C1;LPP;ACTR3C;ATXN1;ZNF407;ZNF124;ANKRD36C;ANKRD26;MBD5;DNAH14;OR1L6;RC3H1;VPS13B;PLEKHA3;ZNF717;AGO3;APLF;WDPCP;CCSER1;BIRC6;BRWD1;KIAA0825;ANKRD36B;TNRC6B*
SEMA6A-AS2	32/100	0.019239054039447853	*NLGN1;PHLPP1;CRACD;FMN1;DSTYK;NKAIN3;UBL3;MXI1;ANKFY1;MAPK1;PHACTR1;MAP4;DIP2C;SOX6;SRGAP2B;PKNOX2;ARNT2;MYEF2;MYO10;MICAL3;MYO5A;CABLES1;MITF;ANK2;GPR137B;IGSF11;ITPKB;NRG3;APC;SPIRE1;MDGA2;MAPRE2*
C1QTNF7-AS1	32/100	0.019239054039447853	*ABCB5;FMN1;PTPRJ;ZFYVE1;TRPM1;BCL2L13;SDCBP;ZFYVE26;DSTYK;SGCD;UBL3;MXI1;LONP2;ABL2;RGS20;RTTN;ZNF106;DIP2C;MYEF2;MYO10;SETDB2;MYO5A;CABLES1;MITF;PARVB;NSG1;GNG12;GPR137B;INPP4B;BCL2;SPIRE1;RAB38*
SLC25A4	32/100	0.0192390540	*PATJ;INO80D;CATSPER2;ANKRD36;KMT2C;ZBTB20;*

8-AS1		39447853	*ZDHHC21;SLC9C1;FBXL20;ATXN1;ZNF407;MED1;ANKRD26;MBD5;MON2;DNAH14;MRTFB;RC3H1;VPS13B;LNPEP;ASH1L;DEFB108B;PHC3;VMP1;ZNF717;AGO3;ZNF678;BIRC6;CDK12;BRWD1;KIAA0825;ANKRD36B*
TSPAN18-AS1	32/100	0.019239054039447853	*ZNF891;ENPEP;INO80D;DYSF;RORA;CACNA1C;SLC9C1;LPP;TTC28;PTAR1;ZNF449;PHACTR2;ALKAL2;PRKG1;PDK1;MBNL1;MBD5;KSR1;ZNF160;INSR;ITGA1;ATP11C;RC3H1;TET1;EML1;PHC3;MYO9A;AGO2;FAT4;ZNF611;PPP1R12B;TNRC6B*
PAPPA-AS1	32/100	0.019239054039447853	*NFAT5;TSPAN33;FSTL1;CMIP;SYNE2;HRH1;ADAMTS2;SNX29;IMPA2;SLC17A1;GPC6;KCNJ1;KL;PAQR5;PDE4D;SLC2A13;SLC12A1;CRIM1;KCNJ15;LAMB1;PPP2R3A;GNG12;ARHGAP24;AIF1L;MYO1E;CLCN5;EVC;LATS2;PAPPA;FAT1;FBN1;SNTB2*
LDLRAD4-AS1	32/100	0.019239054039447853	*INO80D;ANKRD36;KMT2C;ZBTB20;LDLRAD4;BAZ2B;ATXN1;KIAA1328;ZNF407;BPTF;ANKRD26;MBD5;MON2;ANKRD30A;MGA;DNAH14;OR1L6;RC3H1;VPS13B;ASH1L;DEFB108B;PHC3;ZNF717;AGO3;ZNF678;WDPCP;BIRC6;BRWD1;ZNF236;KIAA0825;ANKRD36B;TNRC6B*
LINC00654	31/100	0.03230757286402911	*ZNF573;DIDO1;NFAT5;INO80D;CHD9;ROCK2;KMT2C;ZNF518A;BAZ2B;AKAP11;EPC2;ATP9A;BPTF;MBD5;PTCD2;ERCC6L2;ZBTB38;VPS13C;DENND4C;ATRX;SUSD1;FNDC3A;PLCB4;ZNF717;AGO3;TBC1D5;VPS41;BIRC6;ZNF780B;AVL9;FRYL*
LINC00630	31/100	0.03230757286402911	*ZNF573;INO80D;ANKRD36;ZNF292;KMT2C;ZNF518A;RGPD8;BAZ2B;NIPBL;ZMYM1;CEP192;ZNF124;ANKRD26;MBD5;FANCM;ERCC6L2;VPS13C;MGA;ATRX;RC3H1;VPS13B;ASH1L;PHC3;AGO3;WDPCP;ZNF678;BIRC6;BRWD1;ZNF236;ANKRD36B;TNRC6B*
LINC00863	31/100	0.03230757286402911	*ZNF573;HDAC4;KMT2C;SOGA1;HERC2;HERC1;HECTD4;MARCHF8;ANKFY1;DIP2A;SCAPER;BPTF;CREBBP;MBD5;USP49;FBXW8;ZNF382;ERCC6L2;YLPM1;ASH1L;ARID1B;GATAD2B;TNRC6C;RFX7;TBC1D5;RUFY2;MADD;TTC3;FAM193A;ZNF236;TNRC6B*
LINC02794	31/100	0.03230757286402911	*NRP1;GALNT14;ENPEP;FLT1;ANKRD33B;PRKAA2;ARHGEF28;USH1C;ACSM2A;LRP2;SLC6A3;CNDP2;EPB41L4A;SNX29;IMPA2;ITGB8;ENPP3;SLC17A1;TINAG;CUBN;EGLN3;VCAM1;INSR;CRIM1;PPP2R3A;MYO9A;CLCN5;ALPK2;ZNF611;TRABD2B;CREB5*
LINC01293	31/100	0.03230757286402911	*SHC4;IGSF3;ABCB5;FMN1;LYST;STK10;SDCBP;DSTYK;SGCD;UBL3;CHCHD6;ABL2;RGS20;PHACTR1;PRAME;ZNF106;HMCN1;SRGAP2;ARHGEF11;BCAS3;MYEF2;MYO10;SETDB2;MOK;MYO5A;CABLES1;MITF;NSG1;GPR137B;IGSF11;FAM167B*
LINC00484	31/100	0.03230757286402911	*ZNF573;NFAT5;INO80D;ANKRD36;KMT2C;RGPD6;ZBTB20;RGPD8;BAZ2B;PCNX1;KIAA1328;HERC1;ZNF407;ATP9B;ZFHX3;MBD5;ERCC6L2;MGA;OR1L6;RC3H1;LNPEP;ASH1L;PHC3;ZNF717;AGO3;WDPCP;BIRC6;ZNF780B;UTRN;ANKRD36B;TNRC6B*
LINC01945	31/100	0.03230757286402911	*ITIH5;MEGF10;SH3KBP1;SEMA3A;AKAP6;LAMC1;PCMTD2;SDCBP;GNG2;GNG7;PRAME;HMCN1;SRGAP2;SOX5;MAP4K4;KIRREL1;PHC2;EYA1;MYO10;PCDH7;HMGA2;SORCS1;CORO2B;AIMP1;IGSF11;MOSMO;MMP16;CNTN4;B4GALT6;FREM1;CNIH3*
LINC02030	31/100	0.03230757286402911	*ADCYAP1R1;SLC24A3;BNC2;LUZP2;CTNND2;PCDH15;KLHL13;CACNA1C;NDRG2;TSPAN11;PHF21B;RIMS2;ADAMTS19;CDH20;LRIG1;NCAM1;KCNN3;RFTN2;MY*

			OCD;KCND2;LRRC49;SHISAL1;UNC5D;TAFA5;GHRH;ADGRB1;PRR16;PPP1R12B;PLCB1;PDZRN3;APBA2
LINC02024	31/100	0.03230757286402911	*RYR2;KMT2C;ZBTB20;POTEM;KIAA1328;POTEH;PLCZ1;HECTD4;POTEG;POTEB;ZNF462;ZFHX3;EPHA6;POTEB2;POTEB3;ERCC6L2;ZBTB16;PDE4D;MGA;OR1L6;VPS13B;PHC3;SETBP1;TTC6;NFIA;LRRC9;FAT3;UTRN;DGKI;ANKRD36B;TNRC6B*
LINC02157	31/100	0.03230757286402911	*NRP1;GALNT14;ENPEP;FLT1;PRKAA2;ARHGEF28;RORA;SLC6A3;SYNE2;CNDP2;TTC28;ARHGAP42;EPB41L4A;SNX29;ITGB8;ARSB;VCAM1;INSR;ITGA1;PPP2R3A;MYO9A;SNRK;CLCN5;EVC;GSAP;WDFY3;ALPK2;FAT4;ZNF611;TRABD2B;CREB5*
LINC02516	31/100	0.03230757286402911	*ZNF891;PATJ;INO80D;ANKRD36;GREB1L;ZDHHC21;BAZ2B;SLC9C1;ZNF407;POTED;ZNF124;ANKRD36C;ANKRD26;MBD5;MON2;ANKRD30A;LRBA;VPS13C;DNAH14;OR1L6;RC3H1;VPS13B;ASH1L;PHC3;VMP1;ZNF717;TTC6;ZNF678;BRWD1;KIAA0825;ANKRD36B*
LINC01751	31/100	0.03230757286402911	*NRP1;GALNT14;ENPEP;FLT1;ANKRD33B;PRKAA2;ARHGEF28;LRP2;SLC6A3;CNDP2;ARHGAP42;EPB41L4A;TTC21B;ST8SIA4;ENPP3;PDK1;CUBN;EGLN3;VCAM1;KSR1;INSR;ANXA4;COL23A1;FOXJ3;MYO9A;OR2T2;PLIN2;TLL1;ALPK2;ZNF611;SMPDL3A*
LINC01924	31/100	0.03230757286402911	*NFAT5;ACSS3;CUL5;PPM1L;SLC40A1;CDH7;HS6ST3;SPATA48;AP5M1;KIAA1328;PLCZ1;TRIM2;ZNF208;THSD7A;RNF152;UNC13C;FYB2;STPG2;PDE4D;SCAMP1;MOB1B;INPP4B;TBC1D1;TMEM116;SYT10;LRRC9;ERP27;ATP6V0D2;DGKI;CNTNAP5;ZNF431*
LINC02035	31/100	0.03230757286402911	*MAPKBP1;MACF1;MTMR3;USP31;CHD9;KMT2C;BAZ2B;PCNX1;NIPBL;KPNA1;SPEN;MBNL1;DST;ERCC6L2;EXOC6B;RC3H1;DNAJC13;LNPEP;ASH1L;GAPVD1;PHC3;RC3H2;HIPK3;PIAS1;AGO3;KANSL1;RFX7;SLC49A4;ZNF236;CSNK1G1;TNRC6B*
EGFR-AS1	31/100	0.03230757286402911	*DOCK4;NLGN1;FLT1;TSHZ2;MEOX2;EGFR;CHCHD2;SNTG1;SACS;DPF3;MAPK1;ENPP3;ADAMTS9;ZNF462;EGLN3;WSCD1;KSR1;NEK6;FOXJ3;NETO2;KLHL4;IL17RD;CCDC88A;MMP16;FAT1;FAT3;TCF4;PLIN2;FAT4;ADGRL2;CREB5*
LAMC1-AS1	31/100	0.03230757286402911	*KMT2E;NFAT5;INO80D;TUT4;RGPD6;RGPD5;ZNF518A;RGPD8;LAMC1;BTAF1;MSANTD2;KIAA0753;ZNF449;KHDC4;HIVEP2;TEAD1;ZNF124;ZFHX3;MBD5;ZNF160;DNAH14;FNDC3B;RC3H1;HOOK3;PHC3;VMP1;AGO3;AGO2;PKN2;ZNF236;SNTB2*
TGFA-IT1	31/100	0.03230757286402911	*INO80D;ANKRD36;CHD9;ZNF292;TGFA;RORA;SYNE2;SLC6A3;PTAR1;ACTR3C;ATXN1;CCDC141;PDK1;PRELID2;DNAH11;MBD5;MON2;VPS13C;RC3H1;LNPEP;FAM126B;MYO9A;OR2T2;AGO3;WDFY3;BIRC6;ALPK2;FAT4;ZNF611;KIAA0825;ANKRD36B*
EIPR1-IT1	31/100	0.03230757286402911	*KHDRBS2;CPQ;SDC2;RAP1GAP;LRRC2;PRTG;HIRA;DEPTOR;APMAP;ZMAT4;RGS8;GOLGA8S;ST6GAL2;WSCD2;EIPR1;ZNF804B;KCNAB1;SORBS2;IPCEF1;BMP7;POR;TG;UST;MPPED2;LPCAT2;ELMO1;TRIM58;PKHD1L1;ZMYND11;PTPN4;MATN2*
LAMP5-AS1	31/100	0.03230757286402911	*DOCK3;TENM3;TSHZ2;CELF4;CACNA1E;FBXO41;CLTCL1;CHN1;SRGAP3;ERC2;ADAMTS9;ZNF521;RGS7;GOLGA8S;EPHA4;RBFOX1;PRMT8;GOLGA8T;CADM1;GRID1;GOLGA8J;IL17RD;IL17RA;CNKSR2;MMP16;AMPH;FAT3;CNTN3;TCF4;ANKRD18A;PDZRN4*
SHANK2-AS1	31/100	0.03230757286402911	*PATJ;INO80D;ANKRD36;KMT2C;RGPD6;ZBTB20;BAZ2B;SLC9C1;ATXN1;KIAA1328;ZNF407;ZNF124;ANK*

			RD26;MBD5;ANKRD30B;ANKRD30A;DNAH14;OR1L6;RC3H1;VPS13B;ASH1L;DEFB108B;PHC3;ZNF717;ZNF678;BIRC6;BRWD1;ZNF236;KIAA0825;ANKRD36B;TNRC6B
CNTN4-AS2	31/100	0.03230757286402911	*ZNF891;TSHZ2;RGPD6;ZBTB20;RGPD5;RGPD8;AKAP13;SCAF8;KIAA1328;HECTD2;ZNF407;MPDZ;ADAMTS9;ZNF462;MBD5;ANKRD30A;VPS13B;IL17RD;LRP1B;ZNF717;MMP16;SETBP1;NFIA;TTC3;FAT3;CNTN3;CNTN4;FAT4;UTRN;ZNF236;RBMS3*
TTC3-AS1	31/100	0.03230757286402911	*DDX6;UHRF1BP1L;SETD2;TNKS;LTN1;CHD6;RGPD8;AKAP11;HECTD4;TOGARAM1;EPC2;SCAPER;TRIM23;TTC37;NEK4;ERCC6L2;ATRX;TCF12;YLPM1;ASH1L;PJA2;SYNJ1;APC;TBC1D5;RUFY2;TTC3;CCSER2;ZNF678;BRWD1;ZMYND11;ZNF112*
GPC5-AS2	31/100	0.03230757286402911	*NFAT5;ANKRD36;ZNF292;KMT2C;RGPD6;ZNF518A;RGPD5;RGPD8;ZNF66;RORB;BAZ2B;ANKRD20A1;CDH9;SYNE1;NRIP1;HSF5;SCAI;ZNF124;BPTF;MEF2C;POU1F1;MBD5;ITGA4;RC3H1;KITLG;AGO3;NCOA7;UTRN;MDGA2;DOCK1;TNRC6B*
SLC16A12-AS1	31/100	0.03230757286402911	*CYFIP2;PRKN;GALNT14;ENPEP;PRKAA2;ARHGEF28;ACSM2A;SLC5A12;GLYAT;LRP2;ACSM2B;CNDP2;PBLD;SNX29;IMPA2;SLC16A9;SLC17A1;TINAG;CUBN;KL;MGAM;PAQR5;PLCL1;CRIM1;KCNJ15;MYO9A;ARHGAP24;AIF1L;MSRA;CLCN5;GIPC2*
ID2-AS1	31/100	0.03230757286402911	*ADCYAP1R1;GALNT13;HEPACAM;LUZP2;CTNND2;NRXN1;KLHL32;ADAM22;GRIK4;FMN2;NDRG2;IQCJ-SCHIP1;CDH20;FUT9;NCAM1;KCNN3;MAP4;WASF3;BBS2;RFTN2;NTRK2;DTNA;ZHX3;CADM2;TMOD2;PRKCA;GABRG1;ETNPPL;PTPRD;APC;PPP2R2B*
MSC-AS1	31/100	0.03230757286402911	*NRP1;GALNT14;ENPEP;MTMR2;BNC2;DYSF;ASAP1;ETS1;GLIS1;SDCBP;ARHGAP42;SNX29;CHSY3;DIP2C;GPC6;ARSB;IKBIP;KIRREL1;VCAM1;PLEKHA2;MITF;ANO6;LHFPL2;GNG12;SNAI2;SRFBP1;PLIN2;TLL1;ALPK2;RGL1;LRP12*
VLDLR-AS1	31/100	0.03230757286402911	*FHOD3;PRKN;TBC1D19;ACSS3;ARHGEF28;ZFYVE1;BCL2L13;FGF9;OPA3;KIF21A;THSD7A;ATP6V1E1;DIP2C;TEAD1;PACRG;TANGO2;TTC7B;MITF;ITFG1;MOB1B;AFG3L2;TBC1D1;RCAN2;RRAGD;ATP6V1B2;SPIRE1;ALPK3;ATP6V0D2;CNTNAP5;CPEB4;CDS2*
STARD13-AS	31/100	0.03230757286402911	*ZNF891;NFAT5;INO80D;ANKRD36;KMT2C;RGPD6;ZBTB20;SLC9C1;ACTR3C;ATXN1;ZNF407;ZNF124;MBD5;MON2;VPS13C;DNAH14;OR1L6;RC3H1;VPS13B;PLEKHA3;ASH1L;PHC3;VMP1;ZNF717;AGO3;ZNF678;BIRC6;BRWD1;KIAA0825;ANKRD36B;TNRC6B*
EDRF1-AS1	31/100	0.03230757286402911	*ANKRD17;SETD2;INO80D;KMT2C;TUT4;ZNF518A;RGPD8;PRDM10;RGPD4;BTAF1;HECTD4;NSD1;DIP2A;CEP192;ZNF169;BPTF;USP24;MGA;RC3H1;VPS13B;ASH1L;ARID1B;SFPQ;ELF2;AGO3;KANSL1;RFX7;BIRC6;ZNF236;ANKRD36B;TNRC6B*
BRWD1-AS1	31/100	0.03230757286402911	*ZNF397;ZNF891;CATSPER2;ANKRD36;OR4K2;GREB1L;OR9Q1;KIAA1328;TMEM225;ZNF606;ZMYM4;TRPS1;BPTF;ANKRD26;SLX4IP;MGA;DNAH14;VPS13B;ASH1L;PBX1;GATAD2B;GOLGA6C;GOLGA6B;ZNF717;GOLGA8F;GOLGA6D;TTC3;ZNF678;BRWD1;ZNF236;ANKRD36B*
ARHGAP31-AS1	31/100	0.03230757286402911	*NLGN1;MAML2;PPM1L;CTTNBP2;RNF38;ADAM22;ZBTB20;IQCJ-SCHIP1;EPC2;SOX6;EPHB1;WASF3;BBS2;RFTN2;ZNF462;ARNT2;HFM1;ZHX3;TMEM178B;TCF12;ZFP90;*

			PYGO1;PTPRD;ATAT1;CCDC88A;ARHGAP31;TBC1D5;RRAGD;SPIRE1;ASB3;CREB5
IQCJ-SCHIP1-AS1	31/100	0.03230757286402911	*KMT2E;NFAT5;INO80D;ANKRD36;KMT2C;RGPD6;ZBTB20;RGPD5;RGPD8;BAZ2B;LPP;SYNE1;POTEM;POTEH;POTEG;ZNF124;ZFHX3;MBD5;MGA;OR1L6;RC3H1;VPS13B;ASH1L;PHC3;AGO3;SETBP1;NFIA;BIRC6;UTRN;ANKRD36B;TNRC6B*
ZBTB20-AS4	31/100	0.03230757286402911	*KMT2E;NFAT5;GALNT13;INO80D;CHD9;KMT2C;ADAM22;ZBTB20;BAZ2B;AKAP11;SHOC1;SCAPER;RFTN2;MBD5;RALGAPA1;ERCC6L2;TCF12;YLPM1;VPS13B;ASH1L;PHC3;GATAD2B;ZEB1;FCHSD2;APC;RFX7;RUFY2;TTC3;CCSER2;WDFY3;TNRC6B*
FAM13A-AS1	31/100	0.03230757286402911	*NRP1;GALNT14;ENPEP;FLT1;PRKAA2;ARHGEF28;LDB2;SLC6A3;CNDP2;EPS8;ARHGAP42;EPB41L4A;FCHO2;ENPP3;EGLN3;ARHGEF12;KSR1;INSR;COL23A1;CRIM1;ANO6;MYO9A;ARHGAP24;ZNF33B;SNRK;NUMB;RAPGEF2;PLIN2;ALPK2;ZNF611;SMPDL3A*
NNT-AS1	31/100	0.03230757286402911	*GALNT13;PHLPP1;ADAM22;GRIK4;FMN2;TRIM9;MAP2;DNER;NCAM1;SOX6;TRIM23;GRIA4;BBS2;RFTN2;ARNT2;ZHX3;CADM2;TTC33;PNPLA8;TMOD2;MAGI2;LSAMP;FBXL17;PRKCA;APC;ADGRB3;PPP2R2B;RRAGD;SPIRE1;MAPRE2;ASTN1*
SLIT2-IT1	31/100	0.03230757286402911	*NFAT5;CUL5;PPM1L;RGPD6;ZBTB20;RGPD5;RGPD8;KIAA1328;PLCZ1;THSD7A;RNF152;ZNF124;UNC13C;MBD5;STPG2;ERCC6L2;PDE4D;VPS13C;VPS13B;ADAM32;PLEKHA3;PHC3;MOB1B;INPP4B;SYT10;LRRC9;WDPCP;BRWD1;DGKI;CNTNAP5;ZNF431*
SEMA6A-AS1	31/100	0.03230757286402911	*OCA2;NLGN1;PHLPP1;CRACD;TRPM1;ZNF608;ZNF280B;MXI1;ANKFY1;PHACTR1;SOX6;DIP2C;SRGAP2;JARID2;DISC1;SRGAP2B;PKNOX2;MYEF2;MYO10;MICAL3;MYO5A;TET1;CABLES1;MITF;GPR137B;IGSF11;ITPKB;NRG3;PWWP3A;UBAP1L;MDGA2*
SH3TC2-DT	31/100	0.03230757286402911	*SHC4;FMN1;LYST;PPM1F;COL19A1;STK10;SDCBP;DSTYK;GNPTAB;SCFD2;GNG7;MGAT5;ABL2;PHACTR1;ZNF106;HMCN1;SRGAP2;ANKRD28;COL22A1;MOK;MYO5A;NFATC2;MITF;LHFPL2;S100B;IGSF11;MOSMO;NIN;MELTF;B4GALT6;CNIH3*
KCNIP1-AS1	31/100	0.03230757286402911	*CRACD;MEGF11;TMPRSS3;AGAP1;ILDR2;SLC35F1;FAM171A1;RIC8B;SV2C;MAN2A2;SNTG1;CAPN5;DNER;TSPAN3;CTSE;FAM81A;PAK5;KLHL29;NTRK3;RANBP17;TCF12;CACNA2D3;PRKCA;ARHGAP26;SEZ6L;CDC42BPA;MAPK8IP1;ATAT1;LRFN2;CLDN10;FAT3*
ANO3-AS1	31/100	0.03230757286402911	*ACSS3;CUL5;PPM1L;SLC5A12;GLYAT;PEPD;HS6ST3;CDH7;PBLD;AP5M1;PLCZ1;THSD7A;SLC17A1;RNF152;TINAG;UNC13C;CUBN;STPG2;ABCA5;PDE4D;SLC2A13;WDR72;MOB1B;INPP4B;MSRA;PLCXD3;SYT10;LRRC9;DGKI;CNTNAP5;CPEB4*
NKAIN3-IT1	31/100	0.03230757286402911	*ADCYAP1R1;PHLPP1;HEPACAM;LUZP2;CTNND2;NRXN1;KLHL32;GRIK4;FMN2;NDRG2;TRIM9;NKAIN3;CDH20;FUT9;NCAM1;KCNN3;WASF3;JAM2;GRIA4;RFTN2;NTRK2;DTNA;KCND2;CADM2;TMOD2;LSAMP;APC;ADGRB3;PPP2R2B;APBA2;ASTN1*

Table S3. The overlap between the rDNA-contacting genes co-expressing with lincRNAs obtained from untreated K562 cells (1276 genes) and for HEK293T cells (652 genes). Related to the Venn diagram presented in Figure 1A.

Names	total	elements
HEK293T - K562	346	*SAMD4A ERBB4 KCNMA1 PKNOX2 ZHX3 APBB2 LPP ATRX ADAMTS18 MACF1 FAM214A CTNND2 MITF RTN1 TRIM23 WSCD1 MED13L NRP1 TEAD1 NFIA FRY CDH13 MDGA2 ABL2 DIP2C UTRN PHACTR3 MAML2 SLC8A1 DACH1 EML1 SLC2A13 ANKRD6 RERG SIAH3 SNTG1 CTIF TBC1D5 GREB1 FRMD5 LDLRAD4 STARD13 PCDH7 MMP16 SLC40A1 LRRC4C CADM2 MALRD1 SLCO3A1 PDZRN3 PIK3C3 MYEF2 DCLK1 SLC9C1 CDC42EP3 HMCN1 ZFAND4 GRIK2 IGSF11 RGL1 NKAIN3 NRIP1 NCAM1 ITGA1 CLSTN2 TCF12 CCNG2 KDM4B GNG12 TANC1 CORO2B TTLL11 EVI5 ITGA8 VPS41 GRIK4 ZBTB20 ZNF407 ASB3 TCF4 GABRG1 PBX1 FAM171A1 PHACTR1 PIK3R3 SLC39A12 DISC1 FMN1 RALGPS1 ARHGAP42 PEAK1 EYA1 PHF21B SLC35F1 ADAMTS19 GPR158 GABRB1 NDRG2 TMEM241 APBA2 TTC3 ASAP1 SGSM1 ST8SIA1 UNC79 FYN ANK2 SLC1A2 CADPS CSMD3 DLGAP1 LRBA GRIN2B PHC2 STK32A GRIA1 OTUD7A NEGR1 RABGAP1 FGF12 DGKB CACNG2 EBF1 TULP4 S100B TOX PCDH15 ESR1 ARHGAP12 KLF12 KCNN3 ABCB5 LUZP2 MEGF11 PDE4D MTMR10 LRIG1 ERC2 PRKACB GNG2 PRUNE2 TRIM9 FCHSD2 LAMA1 CDH11 SETBP1 CDS2 DOCK4 MAST4 ITPR2 BRINP1 NRXN1 NTRK3 BCL2L13 WDPCP MAGI1 RAPGEF4 CNKSR2 SNX29 ADCYAP1R1 EPHB1 PRICKLE2 DPP6 LSAMP CTTNBP2 GRID1 GREB1L CREB5 TNRC6B ZDHHC17 ABCC9 GSG1L DCDC1 CNTN3 CACNA1C PRKAA2 CCSER2 TMEM178B DNM3 BBS2 ASXL3 DPF3 FLT1 SCAPER EDIL3 EXOC4 DLG2 NOL4 PPP1R12B SACS SCFD2 RORA PTPRD CDC14B LRRTM4 TLN2 MYO9A FOXN3 NTRK2 LOXL2 WDFY3 GRIK1 SOX5 PRKG1 DSCAM DGKI FTO UBL3 THSD7A NBEA MXI1 AFAP1 NCAM2 TTC28 MAGI2 NELL1 AKAP6 ANKRD30B CSMD2 SRGAP2B IQCJ-SCHIP1 RNF152 CNTN1 DOCK10 OPCML GRM5 ENPEP PLEKHB2 CA10 ANKRD36 LDB2 PRKD1 NRG3 ATP8A1 NLGN1 PTPRG OR9Q1 JAM2 MICAL3 GRID2 LRRC7 LRP2 SEMA6D SYNE1 RALGPS2 FER SNRK MON2 RYR2 FBXL17 MAP2 SUSD4 PRLR PTPRA FAT3 GRIK3 CACNB2 STXBP4 MED15 MTPN EFCAB6 ESYT2 MAPRE2 SOX6 SGCD PDE4DIP MSRA RIC8B PCDH9 MPDZ VCL SEZ6L VCAN ATAT1 CNTNAP5 RBMS3 RAPGEF2 ZMYND11 RAPGEF5 INSR RGS6 SRGAP3 AKAP13 IMMP2L CHCHD6 ATXN1 GABRG2 KIAA1328 UNC13C NRXN3 HS6ST3 PGM5 RALGAPA1 ASTN1 DLGAP2 NSG1 CLIP1 CPE KCND2 SEMA5A CABLES1 ATP8A2 CALD1 SLC24A3 LHFPL3 MPRIP ARHGAP24 GRIA4 CNIH3 DOCK3 CTNNA2 TPH2 APC GPC6 PARD3B CHN1 RELN VPS13C TRPS1 HS3ST4 CEP112 TBC1D9 ROR1 STON1-GTF2A1L SLC4A4 KAZN TNR DTNA CRB1 VAV3 NPAS3 PRKCA KMT2C INPP5A ZNF678 KCNE4 KCNS3 FMN2 ERC1 XYLT1 KIAA0825 AGO3 ALPK2 CCDC88A ARNT2 AFF3*
K562	930	*FSTL1 TBC1D19 HPSE2 SLMAP FAM219A MAP4 TSPAN11 PDE6A KLHL13 HIVEP1 OR1L6 ANKRD36B NBN PRTG RNF38 FYB2 CUBN SEC24D SLC17A1 MPPED2 KMT2E PAK1 EPB41L4A PCMTD2 PELI2 DNAH14 FRMD3 SLC6A11 SCAF8 COL22A1 ATP9B MYO9B TRIO ZNF385D TC2N SETD2 KIRREL1 PWWP3A ITIH5 PACSIN2 SEC23B ZEB1 SLC15A5 FAT4 PARN AMBRA1 CHD9 SYNE2 EPN2 NETO2 PDE1C SLC15A2 DOP1B ZNF208 TTC37 NUP210L TSPAN3 IGSF3 ALPK3 FBXW8 MYO3B EVC ZFYVE1 ZNF718 RC3H1 RPS6KA5 POTED LHFPL2 EPS8 DIP2A TANGO2 SFPQ MAB21L3 TAOK3 HERC2 ZSCAN30 LRFN5 ZNF66 OR4C46 HOOK3 GPR137B PRKCB SLC23A2 ARHGEF7 ROCK2 GPC5 ACACA ZNF704 ARHGEF12 SAMD12 LYPLA1 MAN2A2 CMIP LNPEP PRR16 C1QL3 CDK12 POU1F1 HTR2C ZNF397 TSPAN13 KCNC1 DUX4 GALNT13 DYSF CCDC141 STAU2 NEK4 CACNA1E YTHDF3 SPATA17 SYT10 ZBTB38 PAFAH1B1 TMEM63C FYCO1 LPGAT1 CNTN4 USP14 EXOC6B HECTD2 ST8SIA4 GON4L AFG3L2 CNKSR3 ZNF648 SEMA3A IQCM USP49 TRIM2 PLIN2 SLC8A3 DNAH6 RFX3 MGAT5 RBFOX1 ARFGEF3 FAM167B BCL2 KL PHC3 MYO5B ALCAM USP24 CCSER1 ERLIN2 PPP1R9A ATP11C LIMD1 RAP1GAP ADAM32 DNAH11 SPOPL ADAMTS9 CDC42BPA ANKRD28 MAGI3 SLC13A4 NIPBL CEP120 POTEG ZZEF1*

		CHD6 FRA10AC1 FGD4 FAM135B FSIP1 SNX25 APMAP CCDC186 GSAP SHOC1 MBD5 MYO5C KIF16B SDC2 INO80D KDM5A ZNF382 GOLGA6D NDUFAF6 SLC25A21 MARCHF6 SIPA1L2 ITSN2 RCAN2 ADGRL2 RGPD5 ODR4 GDAP1L1 HIRA UFL1 PAPPA EPHA4 NEBL DIDO1 SYCP1 COL4A3 MYO10 ADAMTS2 PJA2 PRKAA1 RASGEF1C TAFA4 NAALADL2 TSHZ2 FRYL TOM1L2 PBLD IREB2 KIAA1549L ABCC4 FAM126B MYT1 ASH1L ATP10A HECTD4 ELOVL7 BICRAL TTC21B SORBS2 NEK6 MEGF10 ABCD2 OPA3 MOB1B COG2 ZC3HAV1 ARHGAP28 TMEM25 RAB38 CLTCL1 SV2B HECW2 RIC3 ANKRD20A1 ZNF891 VMP1 SLC25A18 KSR1 RALGAPA2 RORB PIEZO2 VSTM4 KIAA1217 RAB27B ZNF236 CTSB MORC3 SVIL ANKS1B NTM TTLL7 LYST FRMPD4 SLC16A9 COL23A1 LRRC2 NSD1 NEDD4L EDAR PLEKHA3 ATP2B2 ANKRD26 PLS1 FRMD6 RGS20 HDGFL3 CNDP2 SH3KBP1 AGO2 NALCN ABCC12 CREBBP DSTYK ZBTB16 PRMT8 LPCAT2 XRCC4 BZW1 FBXL20 FAM193A KIF21A B4GALT6 SLIT3 RGS7 MAP7 FHIT POTEJ SGTB ANTXR1 ZNF518A SHISAL1 SRGAP2 DCAF1 NSG2 CD2AP LRP1B ARSB RGS8 KALRN TRABD2B ZNF124 KIAA0319L BMP7 TAFA5 SPRED1 AP5M1 MYO5A SPEN PLD5 NAV2 KIAA0753 ANKRD17 TSPAN33 PPP2R2C GARNL3 KANSL1 BTBD9 SPATA48 NPIPA1 TPM1 COL19A1 LINGO1 DPH6 CRIM1 TNKS SMPDL3A LRFN2 OR2T2 NOTCH2 FLNB IMPA2 SCAI ZFP90 MADD CLDN10 LTN1 GABRG3 KIAA0232 FKBP5 TINAG NFAT5 SRGAP2C DIRAS2 AQR FLI1 TMOD2 PLCXD3 SLC44A5 FAM107B ANAPC1 ZSWIM6 KCNAB1 KLHL4 CDHR3 TJP1 ARHGAP26 ITGB8 PTPRT TRHDE PTPN4 RIMS1 GXYLT2 HIPK3 WSB1 TMEM225 POR SGMS1 PARVB JCAD AIF1L GLI3 GOLGA8S ZNF780B RBPMS2 GAST LRRC9 WDR26 ATP6V1E1 FRMD4A SLC37A1 PNPLA8 SUPT16H DTWD2 LMX1A JAZF1 SGCG FBN1 WWOX NELL2 RSPH3 WASF3 LONP2 ARID1B MTUS1 HYDIN TRAPPC10 KHDRBS2 TSPAN2 CPQ PRRC1 VPS13D AUH ZNF292 MOSMO SPIN1 EHBP1 SNTB2 KPNA1 FUT8 NEK10 ZNF74 ANKRD31 PIAS1 CDH20 LAMC1 ST6GALNAC3 ZNF611 ZNF521 SRP9 PBX3 PTAR1 PCNX1 MYOCD GALNT10 TRIM58 PRELID2 HMGA2 HHAT KLHL32 GOLGA8J ZMYM1 FHOD3 UBE2QL1 UBAP1L PARP8 PLPPR5 AVL9 ZNF287 FER1L6 RERE CACNA2D3 SEL1L ZNF608 ZNF33B SLC4A10 PHLPP1 SYN2 ATL1 PTCD2 MYO1E SLC7A2 PKN2 ITGA4 CPA6 HFM1 HLCS ACSS3 ROCK1 STK10 HERC1 CFAP70 DOCK1 MGAM GLIS1 GAPVD1 ARHGAP31 UBR1 RXRG ETNPPL ARHGEF11 AMPH INTS7 RGPD4 SOGA1 UHRF1BP1L SORCS1 COL4A2 PACRG SYT1 GRB10 ARHGAP32 SLC27A6 PRKN MUC19 FOXP2 TRPM7 SIAH2 ADSS2 RB1CC1 IKBIP NPHP4 LATS2 DOCK9 FOXJ3 PDE2A CBLIF IL17RD ATP6V1B2 GIPC2 KCNJ1 CDYL2 TTR BBS9 PTPRN2 SLC5A12 NCS1 RANBP3L TG HDAC4 ADGRB3 ADAM29 INPP4B DGKG ZNF717 TRPC5 MYO3A KIAA0513 ENTHD1 AMFR USP31 PLCB1 PLCB4 HIVEP2 MAPK1 LAMB1 SDCBP ATP9A LCLAT1 BPTF TASOR2 CUL5 BIRC6 PLXDC2 SLC6A1 CDH9 EPC2 NFIB DIP2B TUT4 NLK SPOP EGFR GOLGA6B PPFIA2 POTEC ADAM28 STXBP6 PTPRQ CYP4Z1 MGA CLCN5 BCR SLC49A4 LARGE1 ANKRD36C TRAPPC8 MBNL1 RTTN FGF9 ACSM2A STX12 TRAPPC11 NIN FANCM ARHGEF28 PLCL1 SLC1A1 MELTF HPCAL1 FUT9 RAB3GAP2 GALNTL6 MYT1L TMPRSS3 MOK PXDNL ANKFY1 TBC1D1 SLC37A2 SORCS3 LIMCH1 PDP2 SLC12A1 LRRC49 CMPK1 SCN2A ERP27 BAZ2B EML6 POTEB3 ZNRF3 POU6F2 CRACD GNAQ ZNF449 AGL BAZ2A TENM3 ITPKB MEOX2 MED1 MTMR7 GNPTAB EPHA6 DDX6 CTSE TRPM1 CACNG3 IPCEF1 GLIS3 FNDC3A SPIRE1 PHACTR2 RPRD1A COPS8 AKAP9 ARAP2 AIMP1 NECAB1 COL14A1 WDR72 PLCZ1 SNX30 CPEB4 RBM47 PID1 ABL1 NCOR1 JARID2 PIGN PRDM10 PAQR5 EBF2 SHROOM3 FBXO41 MSI2 ELMO1 FAM241A RANBP17 ERBIN PRSS23 ETS1 PCMTD1 PPM1L CABIN1 ZNF573 MBTPS2 KHDC4 SMOC1 PAK5 PDZRN4 SETDB2 HRH1 RABEP1 ST6GAL2 TMEM116 TOGARAM1 SCAMP1 ELF2 IL17RA PTCHD4 LDLRAD3 SLX4IP PKHD1L1 ALKAL2 PLPPR1 CEP192 MCC GLYAT TGFA GALNT14 ZFYVE26 ZNF160 ZNF280B GOLGA8F DHX29 HADHB NFATC2 DST NUMB STPG2 TNRC6C CELF2 CD70 TM9SF2 PDXDC1 DEFB108B NTNG1 WSCD2 ZNF606 VSTM2A GNG7 MTMR2 ZNF804B CAPN5 MLLT10 KCNH5 FBXL7 LRP12 LRRC8B FGF10 CSNK1G1 GOLGA6C ZNF169 KIF13A FAM189A2 NOS1AP CADM1 KLHL29 ABCA4 POTEM RC3H2 SMTN TCERG1L PLEKHA2 SCG3 SYNPR YLPM1 TRPM3 ATF6 EYS ZMYM4 SP3 ITFG1 IPO11 STK32B DIPK1A ZNF106 DEPTOR ZDHHC21 XKR6 POTEB2

		VCAM1 TM9SF3 POTEH DDHD1 RGS12 ICA1 SRFBP1 MATN2 CDH7 IFT81 SHANK2 PDK1 UNC80 CRACR2A WDR41 ILDR2 ZNF615 ATP6V0D2 MIPOL1 PPIP5K2 SYNJ1 PHF20L1 KLF15 TTC6 PPP2R2B DENND4C UNC5D KDM1B ZNF431 ARFGEF1 FAT1 PATJ ITGA9 ENPP1 ULK2 IARS2 S100PBP TANGO6 CELSR2 ZNF462 RIMS2 CFAP97 PTPRJ TET1 CATSPER2 RHPN2 ANKRD30A PPM1F KCNQ3 ANKRD33B SCN8A ZFHX3 DENND2B SNX8 EGLN3 CHSY3 LMX1B RRAGD FNDC3B GFRA1 SHLD2 ATRNL1 SPHKAP GPR156 ZMAT4 BMPR1B MARCHF8 RGPD8 SHC4 MAPKBP1 FCRLA ELP2 ZNF613 UST TLL1 PRDM15 GOLGA8T UBE3A KITLG PYGO1 SV2C TYW1 HSF5 AGAP1 FREM1 ADAM22 GSE1 MAP4K4 EFR3A CERS6 ZNF112 HTR2A KIF21B PRAME NCOA7 ANKRD18A APLF GALNT1 DNER ABCA5 MARCHF1 RFX7 ADGRV1 MACROD2 GATAD2B AKAP11 INO80 BNC2 ANXA4 SLC14A1 MFSD9 TTC33 RANBP2 EOGT KCNJ15 NAT1 POTEB CYBRD1 AUTS2 STRN CRISPLD2 PPP2R3A EIPR1 FAM81A MTMR3 BMP2K SNAI2 CHCHD2 CYFIP2 ADAMTSL1 CCDC126 CLDN18 SYT16 NDFIP1 ADCY9 SLC25A48 MSANTD4 PTPN13 SNAP91 OCA2 CELF4 AKT3 ST8SIA6 GEMIN5 IL1R1 BCAS3 MSANTD2 FCHO2 RFTN2 ANO6 KTN1 ERCC6L2 RUFY2 HEPACAM OR4K2 USP8 USH1C MRTFB DNAH5 SUSD1 HIPK1 CNTN6 ENPP3 CLIC6 ACSM2B HSPA12A TTC7B RGPD6 PEPD APP CHST3 PDLIM5 RPS6KA3 BTAF1 BRWD1 SMAD5 SLC6A3 DNAJC13 NYAP2 PSD3 VPS13B ABCA13 ASB2 EYA2 MAPK8IP1 ADGRB1 CNOT6L MEF2C GHRH TRAPPC6B RAP1GDS1 ZNHIT6 SPOCK1 ACTR3C MYB SLC39A6
HEK293T	306	*ANKS1A RYR3 BBOX1 ZPLD1 PANX1 BID ZNF799 DCHS2 RGMB PPP1R14A SIDT1 RLBP1 TECTA RAP1A KCNA6 MSRB3 DPYSL2 MAP3K3 SYNPO2 CYLC2 SLC22A2 ST6GALNAC5 BCAN ACTR8 ZNF510 STX8 ACYP2 BMPR1A PALLD DSCAML1 PLN CECR2 MRVI1 SLC16A7 C2CD3 KATNAL1 SLC16A12 MUC16 CREG2 BTBD17 MARK1 PI4KA OPHN1 ACBD5 SORT1 FNIP1 PCDHGA7 SLC24A5 CDH10 KIAA1324 MYH9 RNF180 TMEM117 CHST11 MEF2A SLC17A3 TSPAN7 PAQR8 GPATCH2L PCDHGA1 POTEE QKI FLNC LRRTM3 FGF1 CHRM1 CNN3 PKHD1 RBMS2 ABCC11 MYO16 ZNF366 ZNF540 STON1 LRRC53 GANC ACAD11 SCN1A GNAO1 SNN CCDC170 PCDHGB4 TOR1AIP1 ITSN1 CHL1 VANGL2 P2RX6 SPON1 RAB23 PREX2 ZNF516 PI15 OR11L1 GDAP1 DNAJB4 TMEM189 RHBDL3 PCDHGA10 XKR7 EDNRB SPATA6 MYH11 NF1 FAM49A KBTBD11 SHPRH PCDHGA5 FPGT-TNNI3K GAD2 ABI3BP CCDC144A TUB SCAMP5 PCDHGB2 SEL1L2 PCDHGA9 JPH4 NAPEPLD DENND2A SPECC1 SIK2 FAM155A DPY19L2 DENND5B TNS1 TRPC4 MS4A15 FBXL5 OR10K2 DOK6 DRP2 MANEAL ADCY8 FHL5 ZBTB41 PCDHGA12 NFIC ELP3 TRIM51 GRIA3 ARHGAP10 SHC3 IL1RAPL1 PCDHGA4 PANK3 OSGIN2 CLASP2 CAMK2G FGF14 ZMAT3 SNX7 SPRED3 TMEM100 RNF165 DAAM2 PLXNA4 KIAA1109 KIAA0040 MIPEP NDE1 DNAJC6 FERMT2 DACH2 VPS26B ZC3H13 EMCN KDR PCDHGA2 FAM13C DENND5A SEMA5B CC2D2A DAB2 TPRG1 GPR21 PCDHGB7 FAM184B ZNF493 METTL9 YAF2 KIDINS220 MGAT2 CHD7 SBF2 POTEF GTF2A1L FARP2 MYLK ITIH6 ACSBG1 JMY POLR2F TTC39A CACNA1A TSHZ3 MCOLN3 IGDCC4 WDR7 MGAT4C ZNF501 EPG5 LGI1 KHDRBS3 SOX8 CALCRL ABAT ADORA3 ANKRD44 SLC16A4 CELSR1 TNNI3K UNC13A FAM172A ZNF483 PCDHGA8 NCOA1 EPM2A BICC1 IFT88 ARHGAP5 ZFP2 CCDC144NL ZNF720 METTL24 RHOJ RBFOX2 FBXW11 CRMP1 PDE4B MFSD12 ZNF730 ARID4A FILIP1L TMEM108 DPP8 RAB30 MKRN3 IQCJ ISOC1 SPATA5 NVL SLC1A3 SHROOM4 PCDHGA11 ARHGEF6 TGFBR3 SPIN3 ST3GAL3 PTPRM KCNE2 CAB39L GPC3 PCDHGB6 DOK5 DMD ADCY2 CRYL1 NOVA1 FAM114A1 PAX3 GRM4 LRP4 SH3BGR PCDHGB3 TIMP2 SPATA13 ARHGAP35 IGF2R TMEM132B TGFB1I1 RFX4 SH3GL2 FBXL18 LAMA4 CASQ2 ZNF91 BAALC PMP22 PEX5L P2RX7 DOCK7 ST7 FMNL2 ARMCX4 RGS7BP FAM110B FBXO32 PCDHGA6 SYNM AR MAPK4 ARHGEF10 GABRA3 ZCWPW2 PCDHGB1 LRRC3B GPR75 REEP2 CDKL1 PCDHGA3 SLC5A8 GRIA2 SLC25A53 WDR63 WLS SPEF2*

Table S4. GO associations with biological processes associated with 346 overlapping rDNA-contacting genes that are associated with lincRNAs in both K562 and HEK293T cell lines. The search was performed using g:Profiler (https://biit.cs.ut.ee/gprofiler/gost). Related to Figure 1B.

GO.ID	Description	padj	Genes
		**BP**	
GO:0048731	system development	9.510179154170174e-27	*ERBB4,APBB2,ATRX,ADAMTS18,MACF1,CTNND2,MITF,RTN1,NRP1,NFIA,FRY,CDH13,MDGA2,ABL2,SLC8A1,EML1,STARD13,MMP16,SLC40A1,LRRC4C,MYEF2,DCLK1,NRIP1,NCAM1,ITGA1,CLSTN2,TCF12,KDM4B,ITGA8,TCF4,PBX1,PHACTR1,PIK3R3,SLC39A12,DISC1,FMN1,EYA1,GPR158,GABRB1,NDRG2,APBA2,ASAP1,FYN,ANK2,SLC1A2,CSMD3,GRIN2B,GRIA1,NEGR1,FGF12,S100B,TOX,PCDH15,ESR1,ABCB5,MEGF11,LRIG1,PRKACB,LAMA1,CDH11,BRINP1,NRXN1,NTRK3,WDPCP,EPHB1,LSAMP,GREB1L,ZDHHC17,ABCC9,CACNA1C,DNM3,BBS2,DPF3,FLT1,SCAPER,RORA,PTPRD,MYO9A,FOXN3,NTRK2,LOXL2,GRIK1,SOX5,PRKG1,DSCAM,THSD7A,NCAM2,MAGI2,NELL1,AKAP6,SRGAP2B,CNTN1,DOCK10,OPCML,GRM5,ENPEP,CA10,LDB2,PRKD1,NRG3,NLGN1,PTPRG,JAM2,GRID2,LRP2,SEMA6D,SYNE1,FER,RYR2,FBXL17,MAP2,PRLR,FAT3,MTPN,SOX6,SGCD,PCDH9,VCL,SEZ6L,VCAN,ATAT1,RAPGEF2,RAPGEF5,INSR,AKAP13,IMMP2L,ATXN1,GABRG2,NRXN3,ASTN1,CPE,SEMA5A,CABLES1,ATP8A2,CALD1,ARHGAP24,CTNNA2,APC,CHN1,RELN,TRPS1,ROR1,TNR,CRB1,VAV3,PRKCA,XYLT1,ALPK2,CCDC88A,ARNT2*
GO:0034330	cell junction organization	2.023791975805433e-24	*ERBB4,APBB2,MACF1,CTNND2,NRP1,NFIA,CDH13,LRRC4C,PDZRN3,CLSTN2,TANC1,CORO2B,DISC1,FMN1,PEAK1,GPR158,ASAP1,FYN,ANK2,GRIN2B,GRIA1,NEGR1,DGKB,CACNG2,ERC2,CDH11,NRXN1,WDPCP,CNKSR2,EPHB1,CTTNBP2,GRID1,DNM3,PTPRD,TLN2,MYO9A,NTRK2,DSCAM,NBEA,DOCK10,GRM5,NRG3,NLGN1,GRID2,FER,PTPRA,CACNB2,MAPRE2,MPDZ,VCL,SEZ6L,RAPGEF2,INSR,GABRG2,UNC13C,NRXN3,CTNNA2,APC,RELN,TNR,PRKCA,ERC1*
GO:0007399	nervous system development	4.512403013877728e-24	*ERBB4,APBB2,ATRX,MACF1,CTNND2,RTN1,NRP1,NFIA,FRY,MDGA2,ABL2,EML1,LRRC4C,MYEF2,DCLK1,NCAM1,ITGA1,CLSTN2,TCF12,KDM4B,ITGA8,TCF4,PBX1,PHACTR1,SLC39A12,DISC1,EYA1,GPR158,GABRB1,NDRG2,APBA2,ASAP1,FYN,ANK2,SLC1A2,CSMD3,GRIN2B,GRIA1,NEGR1,FGF12,S100B,TOX,PCDH15,LRIG1,PRKACB,LAMA1,CDH11,BRINP1,NRXN1,NTRK3,WDPCP,EPHB1,LSAMP,ZDHHC17,DNM3,BBS2,DPF3,RORA,PTPRD,MYO9A,NTRK2,GRIK1,SOX5,PRKG1,DSCAM,NCAM2,MAGI*

			2,NELL1,SRGAP2B,CNTN1,DOCK10,OPCML,GRM5,CA10,LDB2,PRKD1,NRG3,NLGN1,PTPRG,JAM2,GRID2,LRP2,SEMA6D,SYNE1,FBXL17,MAP2,FAT3,MTPN,SOX6,PCDH9,VCL,SEZ6L,VCAN,ATAT1,RAPGEF2,RAPGEF5,IMMP2L,ATXN1,GABRG2,NRXN3,ASTN1,SEMA5A,CABLES1,ATP8A2,CTNNA2,APC,CHN1,RELN,ROR1,TNR,CRB1,CCDC88A,ARNT2
GO:0007275	multicellular organism development	1.7159463118780894e-22	ERBB4,APBB2,ATRX,ADAMTS18,MACF1,CTNND2,MITF,RTN1,NRP1,TEAD1,NFIA,FRY,CDH13,MDGA2,ABL2,SLC8A1,DACH1,EML1,SIAH3,STARD13,MMP16,SLC40A1,LRRC4C,MYEF2,DCLK1,NRIP1,NCAM1,ITGA1,CLSTN2,TCF12,KDM4B,ITGA8,TCF4,PBX1,PHACTR1,PIK3R3,SLC39A12,DISC1,FMN1,EYA1,GPR158,GABRB1,NDRG2,APBA2,ASAP1,FYN,ANK2,SLC1A2,CSMD3,GRIN2B,GRIA1,NEGR1,FGF12,S100B,TOX,PCDH15,ESR1,ABCB5,MEGF11,LRIG1,PRKACB,LAMA1,CDH11,BRINP1,NRXN1,NTRK3,WDPCP,ADCYAP1R1,EPHB1,LSAMP,GREB1L,ZDHHC17,ABCC9,CACNA1C,DNM3,BBS2,DPF3,FLT1,SCAPER,EXOC4,RORA,PTPRD,MYO9A,FOXN3,NTRK2,LOXL2,GRIK1,SOX5,PRKG1,DSCAM,THSD7A,NCAM2,MAGI2,NELL1,AKAP6,SRGAP2B,CNTN1,DOCK10,OPCML,GRM5,ENPEP,CA10,LDB2,PRKD1,NRG3,NLGN1,PTPRG,JAM2,GRID2,LRP2,SEMA6D,SYNE1,FER,RYR2,FBXL17,MAP2,PRLR,FAT3,MTPN,SOX6,SGCD,PCDH9,VCL,SEZ6L,VCAN,ATAT1,RAPGEF2,RAPGEF5,INSR,AKAP13,IMMP2L,ATXN1,GABRG2,NRXN3,ASTN1,CPE,SEMA5A,CABLES1,ATP8A2,CALD1,ARHGAP24,CTNNA2,APC,CHN1,RELN,TRPS1,ROR1,TNR,CRB1,VAV3,PRKCA,XYLT1,ALPK2,CCDC88A,ARNT2,AFF3
GO:0048699	generation of neurons	1.178452905507181e-18	ERBB4,APBB2,MACF1,CTNND2,RTN1,NRP1,NFIA,FRY,MDGA2,ABL2,EML1,LRRC4C,MYEF2,DCLK1,NCAM1,ITGA1,TCF12,TCF4,PBX1,PHACTR1,SLC39A12,DISC1,EYA1,GABRB1,ASAP1,FYN,CSMD3,NEGR1,S100B,TOX,PCDH15,LAMA1,CDH11,BRINP1,NRXN1,NTRK3,WDPCP,EPHB1,ZDHHC17,DNM3,RORA,PTPRD,MYO9A,NTRK2,SOX5,PRKG1,DSCAM,NCAM2,MAGI2,CNTN1,DOCK10,OPCML,PRKD1,NRG3,NLGN1,PTPRG,GRID2,LRP2,SEMA6D,SYNE1,MAP2,FAT3,MTPN,VCL,VCAN,ATAT1,RAPGEF2,NRXN3,ASTN1,SEMA5A,ATP8A2,CTNNA2,CHN1,RELN,ROR1,TNR,CRB1,CCDC88A
GO:0050808	synapse organization	3.270158284748875e-18	ERBB4,APBB2,CTNND2,NRP1,NFIA,LRRC4C,PDZRN3,CLSTN2,TANC1,DISC1,GPR158,ASAP1,FYN,GRIN2B,GRIA1,NEGR1,DGKB,CACNG2,ERC2,NRXN1,CNKSR2,EPHB1,CTTNBP2,GRID1,DNM3,PTPRD,NTRK2,DSCAM,NBEA,DOCK10,GRM5,NRG3,NLGN1,GRID2,CACNB2,SEZ6L,INSR,GABRG2,U

			NC13C,NRXN3,CTNNA2,RELN,TNR,ERC1
GO:0048856	anatomical structure development	9.2831091162 92847e-18	*ERBB4,APBB2,ATRX,ADAMTS18,MACF1,CTNND2,MITF,RTN1,NRP1,TEAD1,NFIA,FRY,CDH13,MDGA2,ABL2,UTRN,SLC8A1,DACH1,EML1,SIAH3,LDLRAD4,STARD13,MMP16,SLC40A1,LRRC4C,MYEF2,DCLK1,CDC42EP3,NRIP1,NCAM1,ITGA1,CLSTN2,TCF12,KDM4B,TANC1,ITGA8,TCF4,PBX1,FAM171A1,PHACTR1,PIK3R3,SLC39A12,DISC1,FMN1,EYA1,GPR158,GABRB1,NDRG2,APBA2,ASAP1,FYN,ANK2,SLC1A2,CSMD3,GRIN2B,GRIA1,NEGR1,FGF12,S100B,TOX,PCDH15,ESR1,ARHGAP12,ABCB5,MEGF11,PDE4D,LRIG1,PRKACB,LAMA1,CDH11,BRINP1,NRXN1,NTRK3,WDPCP,ADCYAP1R1,EPHB1,LSAMP,GREB1L,ZDHHC17,ABCC9,CACNA1C,DNM3,BBS2,ASXL3,DPF3,FLT1,SCAPER,EXOC4,RORA,PTPRD,MYO9A,FOXN3,NTRK2,LOXL2,GRIK1,SOX5,PRKG1,DSCAM,FTO,THSD7A,NCAM2,MAGI2,NELL1,AKAP6,SRGAP2B,CNTN1,DOCK10,OPCML,GRM5,ENPEP,CA10,LDB2,PRKD1,NRG3,NLGN1,PTPRG,JAM2,GRID2,LRP2,SEMA6D,SYNE1,FER,SNRK,RYR2,FBXL17,MAP2,PRLR,FAT3,MTPN,SOX6,SGCD,PCDH9,VCL,SEZ6L,VCAN,ATAT1,RAPGEF2,RAPGEF5,INSR,AKAP13,IMMP2L,ATXN1,GABRG2,NRXN3,PGM5,ASTN1,CPE,SEMA5A,CABLES1,ATP8A2,CALD1,SLC24A3,ARHGAP24,CTNNA2,APC,CHN1,RELN,TRPS1,ROR1,KAZN,TNR,CRB1,VAV3,PRKCA,FMN2,XYLT1,ALPK2,CCDC88A,ARNT2,AFF3*
GO:0030182	neuron differentiation	6.0011229392 0762e-17	*ERBB4,APBB2,MACF1,CTNND2,RTN1,NRP1,NFIA,FRY,MDGA2,ABL2,LRRC4C,MYEF2,DCLK1,NCAM1,ITGA1,TCF12,TCF4,PBX1,PHACTR1,SLC39A12,DISC1,EYA1,GABRB1,ASAP1,FYN,CSMD3,NEGR1,S100B,TOX,PCDH15,LAMA1,CDH11,BRINP1,NRXN1,NTRK3,WDPCP,EPHB1,ZDHHC17,DNM3,RORA,PTPRD,MYO9A,NTRK2,PRKG1,DSCAM,NCAM2,MAGI2,CNTN1,DOCK10,OPCML,PRKD1,NLGN1,PTPRG,GRID2,LRP2,SEMA6D,SYNE1,MAP2,FAT3,MTPN,VCL,ATAT1,RAPGEF2,NRXN3,SEMA5A,ATP8A2,CTNNA2,CHN1,RELN,ROR1,TNR,CRB1,CCDC88A*
GO:0022008	neurogenesis	3.6199246028 61529e-16	*ERBB4,APBB2,MACF1,CTNND2,RTN1,NRP1,NFIA,FRY,MDGA2,ABL2,EML1,LRRC4C,MYEF2,DCLK1,NCAM1,ITGA1,TCF12,TCF4,PBX1,PHACTR1,SLC39A12,DISC1,EYA1,GABRB1,ASAP1,FYN,CSMD3,NEGR1,S100B,TOX,PCDH15,LAMA1,CDH11,BRINP1,NRXN1,NTRK3,WDPCP,EPHB1,ZDHHC17,DNM3,RORA,PTPRD,MYO9A,NTRK2,SOX5,PRKG1,DSCAM,NCAM2,MAGI2,CNTN1,DOCK10,OPCML,GRM5,PRKD1,NRG3,NLGN1,PTPRG,GRID2,LRP2,SEMA6D,SYNE1,MAP2,FAT3,MTPN,SOX6,VCL,VCAN,ATAT1,R*

			APGEF2,NRXN3,ASTN1,SEMA5A,ATP8A2,CTNNA2,CHN1,RELN,ROR1,TNR,CRB1,CCDC88A
GO:0032502	developmental process	4.670331502233188e-16	*ERBB4,ZHX3,APBB2,ATRX,ADAMTS18,MACF1,CTNND2,MITF,RTN1,NRP1,TEAD1,NFIA,FRY,CDH13,MDGA2,ABL2,UTRN,SLC8A1,DACH1,EML1,SIAH3,LDLRAD4,STARD13,MMP16,SLC40A1,LRRC4C,MYEF2,DCLK1,SLC9C1,CDC42EP3,NRIP1,NCAM1,ITGA1,CLSTN2,TCF12,KDM4B,TANC1,ITGA8,TCF4,PBX1,FAM171A1,PHACTR1,PIK3R3,SLC39A12,DISC1,FMN1,EYA1,GPR158,GABRB1,NDRG2,APBA2,ASAP1,FYN,ANK2,SLC1A2,CSMD3,GRIN2B,PHC2,GRIA1,NEGR1,FGF12,S100B,TOX,PCDH15,ESR1,ARHGAP12,ABCB5,MEGF11,PDE4D,LRIG1,PRKACB,LAMA1,CDH11,BRINP1,NRXN1,NTRK3,WDPCP,ADCYAP1R1,EPHB1,LSAMP,GREB1L,ZDHHC17,ABCC9,CACNA1C,DNM3,BBS2,ASXL3,DPF3,FLT1,SCAPER,EXOC4,RORA,PTPRD,MYO9A,FOXN3,NTRK2,LOXL2,GRIK1,SOX5,PRKG1,DSCAM,FTO,THSD7A,NCAM2,MAGI2,NELL1,AKAP6,SRGAP2B,CNTN1,DOCK10,OPCML,GRM5,ENPEP,PLEKHB2,CA10,LDB2,PRKD1,NRG3,NLGN1,PTPRG,JAM2,GRID2,LRP2,SEMA6D,SYNE1,FER,SNRK,RYR2,FBXL17,MAP2,PRLR,FAT3,MED15,MTPN,SOX6,SGCD,PCDH9,VCL,SEZ6L,VCAN,ATAT1,RAPGEF2,RAPGEF5,INSR,AKAP13,IMMP2L,ATXN1,GABRG2,UNC13C,NRXN3,PGM5,ASTN1,CPE,SEMA5A,CABLES1,ATP8A2,CALD1,SLC24A3,ARHGAP24,CTNNA2,APC,CHN1,RELN,TRPS1,ROR1,KAZN,TNR,CRB1,VAV3,PRKCA,FMN2,XYLT1,ALPK2,CCDC88A,ARNT2,AFF3*
GO:0007268	chemical synaptic transmission	5.438063231062327e-16	*LRRC4C,GRIK2,IGSF11,CLSTN2,GRIK4,GABRG1,DISC1,GPR158,APBA2,FYN,SLC1A2,CADPS,DLGAP1,GRIN2B,GRIA1,FGF12,DGKB,CACNG2,S100B,ERC2,FCHSD2,CDH11,NRXN1,EPHB1,GRID1,EXOC4,DLG2,PTPRD,NTRK2,GRIK1,DGKI,GRM5,NRG3,NLGN1,GRID2,SYNE1,PTPRA,GRIK3,CACNB2,RAPGEF2,GABRG2,UNC13C,NRXN3,DLGAP2,NSG1,KCND2,GRIA4,RELN,TNR,DTNA,ERC1*
GO:0098916	anterograde trans-synaptic signaling	5.438063231062327e-16	*LRRC4C,GRIK2,IGSF11,CLSTN2,GRIK4,GABRG1,DISC1,GPR158,APBA2,FYN,SLC1A2,CADPS,DLGAP1,GRIN2B,GRIA1,FGF12,DGKB,CACNG2,S100B,ERC2,FCHSD2,CDH11,NRXN1,EPHB1,GRID1,EXOC4,DLG2,PTPRD,NTRK2,GRIK1,DGKI,GRM5,NRG3,NLGN1,GRID2,SYNE1,PTPRA,GRIK3,CACNB2,RAPGEF2,GABRG2,UNC13C,NRXN3,DLGAP2,NSG1,KCND2,GRIA4,RELN,TNR,DTNA,ERC1*
GO:0099536	synaptic signaling	5.488015893095248e-16	*UTRN,LRRC4C,GRIK2,IGSF11,CLSTN2,GRIK4,GABRG1,DISC1,GPR158,APBA2,FYN,SLC1A2,CADPS,DLGAP1,GRIN2B,GRIA*

			1,FGF12,DGKB,CACNG2,S100B,ERC2,FCHSD2,CDH11,NRXN1,EPHB1,GRID1,EXOC4,DLG2,PTPRD,NTRK2,GRIK1,DGKI,GRM5,NRG3,NLGN1,GRID2,SYNE1,PTPRA,GRIK3,CACNB2,RAPGEF2,GABRG2,UNC13C,NRXN3,DLGAP2,NSG1,KCND2,GRIA4,RELN,TNR,DTNA,ERC1
GO:0099537	trans-synaptic signaling	7.653461834547519e-16	*LRRC4C,GRIK2,IGSF11,CLSTN2,GRIK4,GABRG1,DISC1,GPR158,APBA2,FYN,SLC1A2,CADPS,DLGAP1,GRIN2B,GRIA1,FGF12,DGKB,CACNG2,S100B,ERC2,FCHSD2,CDH11,NRXN1,EPHB1,GRID1,EXOC4,DLG2,PTPRD,NTRK2,GRIK1,DGKI,GRM5,NRG3,NLGN1,GRID2,SYNE1,PTPRA,GRIK3,CACNB2,RAPGEF2,GABRG2,UNC13C,NRXN3,DLGAP2,NSG1,KCND2,GRIA4,RELN,TNR,DTNA,ERC1*
GO:0032501	multicellular organismal process	1.823482863059553e-15	*ERBB4,KCNMA1,ZHX3,APBB2,ATRX,ADAMTS18,MACF1,CTNND2,MITF,RTN1,NRP1,TEAD1,NFIA,FRY,CDH13,MDGA2,ABL2,UTRN,SLC8A1,DACH1,EML1,SLC2A13,SIAH3,LDLRAD4,STARD13,MMP16,SLC40A1,LRRC4C,SLCO3A1,MYEF2,DCLK1,SLC9C1,HMCN1,GRIK2,IGSF11,NRIP1,NCAM1,ITGA1,CLSTN2,TCF12,KDM4B,TANC1,CORO2B,ITGA8,ZBTB20,ASB3,TCF4,PBX1,PHACTR1,PIK3R3,SLC39A12,DISC1,FMN1,ARHGAP42,EYA1,GPR158,GABRB1,NDRG2,APBA2,ASAP1,FYN,ANK2,SLC1A2,CSMD3,GRIN2B,PHC2,GRIA1,NEGR1,FGF12,DGKB,CACNG2,S100B,TOX,PCDH15,ESR1,ABCB5,MEGF11,PDE4D,LRIG1,PRKACB,LAMA1,CDH11,DOCK4,BRINP1,NRXN1,NTRK3,WDPCP,ADCYAP1R1,EPHB1,LSAMP,GRID1,GREB1L,ZDHHC17,ABCC9,CACNA1C,PRKAA2,DNM3,BBS2,DPF3,FLT1,SCAPER,EXOC4,PPP1R12B,RORA,PTPRD,MYO9A,FOXN3,NTRK2,LOXL2,GRIK1,SOX5,PRKG1,DSCAM,DGKI,FTO,THSD7A,NBEA,NCAM2,MAGI2,NELL1,AKAP6,SRGAP2B,CNTN1,DOCK10,OPCML,GRM5,ENPEP,CA10,LDB2,PRKD1,NRG3,ATP8A1,NLGN1,PTPRG,OR9Q1,JAM2,GRID2,LRP2,SEMA6D,SYNE1,FER,RYR2,FBXL17,MAP2,PRLR,FAT3,CACNB2,MED15,MTPN,MAPRE2,SOX6,SGCD,PCDH9,VCL,SEZ6L,VCAN,ATAT1,RAPGEF2,RAPGEF5,INSR,AKAP13,IMMP2L,ATXN1,GABRG2,NRXN3,ASTN1,CPE,KCND2,SEMA5A,CABLES1,ATP8A2,CALD1,SLC24A3,LHFPL3,ARHGAP24,CTNNA2,APC,CHN1,RELN,TRPS1,ROR1,SLC4A4,KAZN,TNR,DTNA,CRB1,VAV3,PRKCA,KCNE4,FMN2,XYLT1,ALPK2,CCDC88A,ARNT2,AFF3*
GO:0048666	neuron development	2.4320485113264353e-15	*APBB2,MACF1,CTNND2,NRP1,FRY,ABL2,LRRC4C,DCLK1,NCAM1,ITGA1,PBX1,PHACTR1,SLC39A12,DISC1,GABRB1,ASAP1,FYN,CSMD3,NEGR1,S100B,TOX,PCDH15,LAMA1,CDH11,BRINP1,NRXN1,NTRK3,WDPCP,EPHB1,ZDHHC17,DNM3,PTPRD,MYO9*

			A,NTRK2,PRKG1,DSCAM,NCAM2,MAGI2,CNTN1,DOCK10,OPCML,PRKD1,NLGN1,PTPRG,GRID2,LRP2,SEMA6D,SYNE1,MAP2,FAT3,VCL,ATAT1,RAPGEF2,NRXN3,SEMA5A,ATP8A2,CTNNA2,CHN1,RELN,ROR1,TNR,CRB1,CCDC88A
GO:0007267	cell-cell signaling	1.9640264386329733e-14	*MACF1,CTNND2,MITF,NRP1,UTRN,ANKRD6,LRRC4C,GRIK2,IGSF11,CLSTN2,GRIK4,GABRG1,DISC1,GPR158,GABRB1,NDRG2,APBA2,FYN,ANK2,SLC1A2,CADPS,DLGAP1,GRIN2B,GRIA1,FGF12,DGKB,CACNG2,S100B,ERC2,FCHSD2,CDH11,NRXN1,RAPGEF4,EPHB1,PRICKLE2,GRID1,CACNA1C,PRKAA2,EXOC4,DLG2,PTPRD,NTRK2,GRIK1,DGKI,MAGI2,GRM5,ENPEP,PRKD1,NRG3,NLGN1,GRID2,SYNE1,RYR2,PTPRA,GRIK3,CACNB2,STXBP4,RBMS3,RAPGEF2,GABRG2,UNC13C,NRXN3,DLGAP2,NSG1,CPE,KCND2,SEMA5A,GRIA4,APC,RELN,ROR1,TNR,DTNA,CRB1,ERC1,ALPK2*
GO:0009653	anatomical structure morphogenesis	2.6764543230726155e-14	*ERBB4,APBB2,ATRX,MACF1,CTNND2,NRP1,NFIA,FRY,CDH13,STARD13,MMP16,SLC40A1,LRRC4C,DCLK1,CDC42EP3,NCAM1,ITGA1,TANC1,ITGA8,PBX1,FAM171A1,PHACTR1,PIK3R3,SLC39A12,DISC1,FMN1,EYA1,FYN,ANK2,S100B,PCDH15,ESR1,ARHGAP12,MEGF11,LRIG1,PRKACB,LAMA1,CDH11,NRXN1,WDPCP,EPHB1,GREB1L,ZDHHC17,ABCC9,CACNA1C,DNM3,BBS2,ASXL3,FLT1,EXOC4,RORA,PTPRD,MYO9A,FOXN3,NTRK2,LOXL2,SOX5,PRKG1,DSCAM,THSD7A,MAGI2,CNTN1,DOCK10,ENPEP,PRKD1,NRG3,NLGN1,JAM2,GRID2,LRP2,SEMA6D,SYNE1,RYR2,MAP2,FAT3,MTPN,SOX6,SGCD,VCL,RAPGEF2,INSR,AKAP13,NRXN3,PGM5,CPE,SEMA5A,ATP8A2,CALD1,ARHGAP24,CTNNA2,APC,CHN1,RELN,TNR,CRB1,VAV3,PRKCA,ALPK2,AFF3*
GO:0031175	neuron projection development	1.0235204144000854e-13	*APBB2,MACF1,CTNND2,NRP1,FRY,ABL2,LRRC4C,DCLK1,NCAM1,ITGA1,PHACTR1,SLC39A12,DISC1,ASAP1,FYN,CSMD3,NEGR1,S100B,TOX,PCDH15,LAMA1,CDH11,NRXN1,NTRK3,EPHB1,ZDHHC17,DNM3,PTPRD,MYO9A,NTRK2,PRKG1,DSCAM,NCAM2,MAGI2,CNTN1,DOCK10,PRKD1,NLGN1,PTPRG,GRID2,LRP2,SEMA6D,SYNE1,MAP2,FAT3,VCL,RAPGEF2,NRXN3,SEMA5A,ATP8A2,CTNNA2,CHN1,RELN,ROR1,TNR,CCDC88A*
GO:0065008	regulation of biological quality	1.5712523839621706e-13	*SAMD4A,ERBB4,KCNMA1,ADAMTS18,MACF1,NRP1,ABL2,SLC8A1,SIAH3,SLC9C1,CDC42EP3,GRIK2,IGSF11,ITGA1,CLSTN2,TANC1,CORO2B,GRIK4,GABRG1,FAM171A1,DISC1,FMN1,ARHGAP42,GPR158,GABRB1,ASAP1,FYN,ANK2,GRIN2B,GRIA1,NEGR1,FGF12,DGKB,CACNG2,S100B,ESR1,ABCB5,PDE4D,ERC2,PRKACB,FCHSD2,DOCK4,NRXN1,WDPCP,RAPGEF4,ADCYAP1R1,EPHB1,CTTNBP2,GRID1,TNRC6B,ABCC*

			9,GSG1L,CACNA1C,DNM3,BBS2,PTPRD,NTRK2,GRIK1,PRKG1,DSCAM,DGKI,FTO,NBEA,AKAP6,GRM5,ENPEP,PRKD1,ATP8A1,NLGN1,GRID2,SEMA6D,SYNE1,FER,RYR2,MAP2,PRLR,GRIK3,CACNB2,STXBP4,MTPN,VCL,RAPGEF2,GABRG2,UNC13C,NRXN3,NSG1,CPE,KCND2,SEMA5A,ATP8A2,GRIA4,CTNNA2,GPC6,RELN,SLC4A4,TNR,VAV3,PRKCA,KCNE4,ERC1,AGO3
GO:0048513	animal organ development	1.8911502004397216e-13	*ERBB4,ATRX,ADAMTS18,MITF,RTN1,NRP1,TEAD1,NFIA,UTRN,SLC8A1,EML1,LDLRAD4,MMP16,SLC40A1,DCLK1,NRIP1,NCAM1,TCF12,KDM4B,ITGA8,PBX1,PHACTR1,DISC1,FMN1,EYA1,GPR158,NDRG2,FYN,ANK2,SLC1A2,GRIN2B,GRIA1,FGF12,TOX,PCDH15,ESR1,ABCB5,MEGF11,LRIG1,LAMA1,CDH11,BRINP1,NRXN1,NTRK3,WDPCP,EPHB1,GREB1L,ABCC9,CACNA1C,BBS2,ASXL3,DPF3,FLT1,SCAPER,EXOC4,RORA,FOXN3,NTRK2,LOXL2,SOX5,PRKG1,DSCAM,FTO,MAGI2,NELL1,AKAP6,CNTN1,ENPEP,CA10,LDB2,NRG3,GRID2,LRP2,SEMA6D,SYNE1,FER,RYR2,FBXL17,PRLR,FAT3,MTPN,SOX6,SGCD,PCDH9,ATAT1,RAPGEF2,INSR,AKAP13,IMMP2L,ATXN1,CPE,SEMA5A,ATP8A2,SLC24A3,CTNNA2,APC,RELN,TRPS1,ROR1,KAZN,TNR,CRB1,XYLT1,ALPK2,ARNT2*
GO:0050804	modulation of chemical synaptic transmission	4.0539475756367744e-13	*LRRC4C,GRIK2,IGSF11,CLSTN2,GRIK4,DISC1,GPR158,APBA2,FYN,GRIN2B,GRIA1,DGKB,CACNG2,S100B,ERC2,CDH11,NRXN1,EPHB1,GRID1,PTPRD,NTRK2,GRIK1,DGKI,GRM5,NRG3,NLGN1,GRID2,SYNE1,PTPRA,GRIK3,RAPGEF2,UNC13C,NRXN3,NSG1,GRIA4,RELN,TNR,ERC1*
GO:0099177	regulation of trans-synaptic signaling	4.335883391750187e-13	*LRRC4C,GRIK2,IGSF11,CLSTN2,GRIK4,DISC1,GPR158,APBA2,FYN,GRIN2B,GRIA1,DGKB,CACNG2,S100B,ERC2,CDH11,NRXN1,EPHB1,GRID1,PTPRD,NTRK2,GRIK1,DGKI,GRM5,NRG3,NLGN1,GRID2,SYNE1,PTPRA,GRIK3,RAPGEF2,UNC13C,NRXN3,NSG1,GRIA4,RELN,TNR,ERC1*
GO:0050789	regulation of biological process	4.3426714956934525e-13	*SAMD4A,ERBB4,KCNMA1,PKNOX2,ZHX3,APBB2,ATRX,ADAMTS18,MACF1,CTNND2,MITF,RTN1,TRIM23,MED13L,NRP1,TEAD1,NFIA,FRY,CDH13,ABL2,UTRN,MAML2,SLC8A1,DACH1,SLC2A13,ANKRD6,RERG,SIAH3,CTIF,TBC1D5,FRMD5,LDLRAD4,STARD13,SLC40A1,LRRC4C,MALRD1,SLCO3A1,PIK3C3,DCLK1,CDC42EP3,GRIK2,IGSF11,RGL1,NKAIN3,NRIP1,NCAM1,ITGA1,CLSTN2,TCF12,CCNG2,KDM4B,GNG12,TANC1,CORO2B,ITGA8,VPS41,GRIK4,ZBTB20,ZNF407,ASB3,TCF4,GABRG1,PBX1,FAM171A1,PHACTR1,PIK3R3,SLC39A12,DISC1,FMN1,RALGPS1,ARHGAP42,PEAK1,EYA1,GPR158,GABRB1,NDRG2,APBA2,ASAP1,ST8SIA1,FYN,ANK2,SLC1A2,CADPS,CSMD3,DLGAP1,GRIN2B,PHC2,STK32*

			A,GRIA1,NEGR1,FGF12,DGKB,CACNG2,EBF1,S100B,TOX,ESR1,ARHGAP12,KLF12,PDE4D,ERC2,PRKACB,GNG2,FCHSD2,LAMA1,CDH11,SETBP1,DOCK4,MAST4,ITPR2,BRINP1,NRXN1,NTRK3,BCL2L13,WDPCP,MAGI1,RAPGEF4,CNKSR2,ADCYAP1R1,EPHB1,PRICKLE2,DPP6,CTTNBP2,GRID1,CREB5,TNRC6B,ZDHHC17,ABCC9,GSG1L,DCDC1,CACNA1C,PRKAA2,DNM3,BBS2,ASXL3,DPF3,FLT1,EDIL3,EXOC4,DLG2,PPP1R12B,SACS,RORA,PTPRD,CDC14B,MYO9A,FOXN3,NTRK2,LOXL2,GRIK1,SOX5,PRKG1,DSCAM,DGKI,FTO,NBEA,MXI1,TTC28,MAGI2,NELL1,AKAP6,IQCJ-SCHIP1,RNF152,CNTN1,DOCK10,GRM5,ENPEP,PLEKHB2,LDB2,PRKD1,NRG3,ATP8A1,NLGN1,PTPRG,OR9Q1,JAM2,GRID2,LRP2,SEMA6D,SYNE1,RALGPS2,FER,SNRK,RYR2,FBXL17,MAP2,SUSD4,PRLR,PTPRA,FAT3,GRIK3,CACNB2,STXBP4,MED15,MTPN,MAPRE2,SOX6,SGCD,PDE4DIP,RIC8B,MPDZ,VCL,SEZ6L,VCAN,ATAT1,RBMS3,RAPGEF2,ZMYND11,RAPGEF5,INSR,RGS6,SRGAP3,AKAP13,ATXN1,GABRG2,UNC13C,NRXN3,RALGAPA1,DLGAP2,NSG1,CLIP1,CPE,KCND2,SEMA5A,CABLES1,ATP8A2,SLC24A3,ARHGAP24,GRIA4,CNIH3,DOCK3,CTNNA2,APC,GPC6,CHN1,RELN,VPS13C,TRPS1,ROR1,SLC4A4,TNR,DTNA,CRB1,VAV3,NPAS3,PRKCA,KMT2C,INPP5A,ZNF678,KCNE4,FMN2,ERC1,AGO3,ALPK2,CCDC88A,ARNT2,AFF3
GO:0120036	plasma membrane bounded cell projection organization	1.938829867636505e-12	*APBB2,MACF1,CTNND2,NRP1,FRY,CDH13,ABL2,LRRC4C,DCLK1,CDC42EP3,NCAM1,ITGA1,TANC1,PHACTR1,SLC39A12,DISC1,ASAP1,FYN,CSMD3,GRIN2B,NEGR1,S100B,TOX,PCDH15,LAMA1,CDH11,NRXN1,NTRK3,WDPCP,EPHB1,ZDHHC17,DNM3,BBS2,PTPRD,CDC14B,MYO9A,NTRK2,PRKG1,DSCAM,NCAM2,MAGI2,CNTN1,DOCK10,PRKD1,NLGN1,PTPRG,GRID2,LRP2,SEMA6D,SYNE1,FER,MAP2,FAT3,VCL,ATAT1,RAPGEF2,INSR,NRXN3,SEMA5A,ATP8A2,ARHGAP24,CTNNA2,APC,CHN1,RELN,ROR1,TNR,VAV3,CCDC88A*
GO:0030030	cell projection organization	2.170354676121407e-12	*APBB2,MACF1,CTNND2,NRP1,FRY,CDH13,ABL2,LRRC4C,DCLK1,CDC42EP3,NCAM1,ITGA1,TANC1,ITGA8,PHACTR1,SLC39A12,DISC1,ASAP1,FYN,CSMD3,GRIN2B,NEGR1,S100B,TOX,PCDH15,LAMA1,CDH11,NRXN1,NTRK3,WDPCP,EPHB1,ZDHHC17,DNM3,BBS2,PTPRD,CDC14B,MYO9A,NTRK2,PRKG1,DSCAM,NCAM2,MAGI2,CNTN1,DOCK10,PRKD1,NLGN1,PTPRG,GRID2,LRP2,SEMA6D,SYNE1,FER,MAP2,FAT3,VCL,ATAT1,RAPGEF2,INSR,NRXN3,SEMA5A,ATP8A2,ARHGAP24,CTNNA2,APC,CHN1,RELN,ROR1,TNR,VAV3,CCDC88A*
GO:0007155	cell	3.7359122155	*LPP,ADAMTS18,MACF1,CTNND2,NRP1,CD*

	adhesion	50562e-12	*H13,ABL2,UTRN,FRMD5,PCDH7,LRRC4C,CADM2,HMCN1,IGSF11,NCAM1,ITGA1,CLSTN2,CORO2B,ITGA8,DISC1,FMN1,PEAK1,FYN,NEGR1,S100B,PCDH15,MEGF11,LAMA1,CDH11,NRXN1,WDPCP,MAGI1,EPHB1,LSAMP,CNTN3,EDIL3,DLG2,PTPRD,TLN2,PRKG1,DSCAM,NCAM2,CNTN1,OPCML,NLGN1,JAM2,GRID2,FER,PRLR,PTPRA,FAT3,PCDH9,VCL,VCAN,CNTNAP5,NRXN3,PGM5,ASTN1,SEMA5A,CTNNA2,APC,PARD3B,RELN,TNR,CRB1,VAV3,PRKCA*
GO:0065007	biological regulation	3.9387956301 39636e-12	*SAMD4A,ERBB4,KCNMA1,PKNOX2,ZHX3,APBB2,ATRX,ADAMTS18,MACF1,CTNND2,MITF,RTN1,TRIM23,MED13L,NRP1,TEAD1,NFIA,FRY,CDH13,ABL2,UTRN,MAML2,SLC8A1,DACH1,SLC2A13,ANKRD6,RERG,SIAH3,CTIF,TBC1D5,FRMD5,LDLRAD4,STARD13,SLC40A1,LRRC4C,MALRD1,SLCO3A1,PIK3C3,DCLK1,SLC9C1,CDC42EP3,GRIK2,IGSF11,RGL1,NKAIN3,NRIP1,NCAM1,ITGA1,CLSTN2,TCF12,CCNG2,KDM4B,GNG12,TANC1,CORO2B,EVI5,ITGA8,VPS41,GRIK4,ZBTB20,ZNF407,ASB3,TCF4,GABRG1,PBX1,FAM171A1,PHACTR1,PIK3R3,SLC39A12,DISC1,FMN1,RALGPS1,ARHGAP42,PEAK1,EYA1,GPR158,GABRB1,NDRG2,APBA2,ASAP1,ST8SIA1,FYN,ANK2,SLC1A2,CADPS,CSMD3,DLGAP1,GRIN2B,PHC2,STK32A,GRIA1,NEGR1,FGF12,DGKB,CACNG2,EBF1,S100B,TOX,ESR1,ARHGAP12,KLF12,ABCB5,PDE4D,ERC2,PRKACB,GNG2,FCHSD2,LAMA1,CDH11,SETBP1,DOCK4,MAST4,ITPR2,BRINP1,NRXN1,NTRK3,BCL2L13,WDPCP,MAGI1,RAPGEF4,CNKSR2,ADCYAP1R1,EPHB1,PRICKLE2,DPP6,CTTNBP2,GRID1,CREB5,TNRC6B,ZDHHC17,ABCC9,GSG1L,DCDC1,CACNA1C,PRKAA2,DNM3,BBS2,ASXL3,DPF3,FLT1,EDIL3,EXOC4,DLG2,PPP1R12B,SACS,RORA,PTPRD,CDC14B,MYO9A,FOXN3,NTRK2,LOXL2,GRIK1,SOX5,PRKG1,DSCAM,DGKI,FTO,NBEA,MXI1,TTC28,MAGI2,NELL1,AKAP6,IQCJ-SCHIP1,RNF152,CNTN1,DOCK10,GRM5,ENPEP,PLEKHB2,LDB2,PRKD1,NRG3,ATP8A1,NLGN1,PTPRG,OR9Q1,JAM2,GRID2,LRP2,SEMA6D,SYNE1,RALGPS2,FER,SNRK,RYR2,FBXL17,MAP2,SUSD4,PRLR,PTPRA,FAT3,GRIK3,CACNB2,STXBP4,MED15,MTPN,MAPRE2,SOX6,SGCD,PDE4DIP,RIC8B,MPDZ,VCL,SEZ6L,VCAN,ATAT1,RBMS3,RAPGEF2,ZMYND11,RAPGEF5,INSR,RGS6,SRGAP3,AKAP13,ATXN1,GABRG2,UNC13C,NRXN3,RALGAPA1,DLGAP2,NSG1,CLIP1,CPE,KCND2,SEMA5A,CABLES1,ATP8A2,SLC24A3,ARHGAP24,GRIA4,CNIH3,DOCK3,CTNNA2,APC,GPC6,CHN1,RELN,VPS13C,TRPS1,ROR1,SLC4A4,TNR,DTNA,CRB1,VAV3,NPAS3,PRKCA,KMT2C,INPP5A*

			,*ZNF678,KCNE4,FMN2,ERC1,AGO3,ALPK2,CCDC88A,ARNT2,AFF3*
GO:0007154	cell communication	4.28247976429831e-12	*ERBB4,APBB2,ATRX,ADAMTS18,MACF1,CTNND2,MITF,NRP1,TEAD1,NFIA,CDH13,ABL2,UTRN,MAML2,SLC8A1,ANKRD6,RERG,SNTG1,LDLRAD4,STARD13,LRRC4C,PIK3C3,DCLK1,CDC42EP3,GRIK2,IGSF11,RGL1,NCAM1,ITGA1,CLSTN2,GNG12,ITGA8,VPS41,GRIK4,ASB3,GABRG1,PIK3R3,SLC39A12,DISC1,RALGPS1,ARHGAP42,EYA1,GPR158,GABRB1,NDRG2,APBA2,FYN,ANK2,SLC1A2,CADPS,DLGAP1,GRIN2B,STK32A,GRIA1,FGF12,DGKB,CACNG2,S100B,ESR1,ARHGAP12,PDE4D,ERC2,PRKACB,GNG2,FCHSD2,LAMA1,CDH11,DOCK4,MAST4,ITPR2,NRXN1,NTRK3,WDPCP,MAGI1,RAPGEF4,CNKSR2,ADCYAP1R1,EPHB1,PRICKLE2,GRID1,ZDHHC17,DCDC1,CACNA1C,PRKAA2,BBS2,FLT1,EXOC4,DLG2,PPP1R12B,RORA,PTPRD,CDC14B,MYO9A,FOXN3,NTRK2,GRIK1,PRKG1,DSCAM,DGKI,MAGI2,AKAP6,IQCJ-SCHIP1,RNF152,CNTN1,DOCK10,GRM5,ENPEP,PRKD1,NRG3,NLGN1,PTPRG,OR9Q1,GRID2,LRP2,SEMA6D,SYNE1,RALGPS2,FER,SNRK,RYR2,FBXL17,PRLR,PTPRA,GRIK3,CACNB2,STXBP4,MAPRE2,SGCD,RIC8B,SEZ6L,ATAT1,RBMS3,RAPGEF2,ZMYND11,RAPGEF5,INSR,RGS6,SRGAP3,AKAP13,GABRG2,UNC13C,NRXN3,RALGAPA1,DLGAP2,NSG1,CPE,KCND2,SEMA5A,ARHGAP24,GRIA4,CNIH3,DOCK3,APC,GPC6,CHN1,RELN,ROR1,TNR,DTNA,CRB1,VAV3,PRKCA,INPP5A,FMN2,ERC1,AGO3,ALPK2,CCDC88A*
GO:0023052	signaling	6.480835719666655e-12	*ERBB4,APBB2,ATRX,ADAMTS18,MACF1,CTNND2,MITF,NRP1,TEAD1,NFIA,CDH13,ABL2,UTRN,MAML2,SLC8A1,ANKRD6,RERG,LDLRAD4,STARD13,LRRC4C,PIK3C3,DCLK1,CDC42EP3,GRIK2,IGSF11,RGL1,NCAM1,ITGA1,CLSTN2,GNG12,ITGA8,GRIK4,ASB3,GABRG1,PIK3R3,SLC39A12,DISC1,RALGPS1,ARHGAP42,EYA1,GPR158,GABRB1,NDRG2,APBA2,FYN,ANK2,SLC1A2,CADPS,DLGAP1,GRIN2B,STK32A,GRIA1,FGF12,DGKB,CACNG2,S100B,ESR1,ARHGAP12,PDE4D,ERC2,PRKACB,GNG2,FCHSD2,LAMA1,CDH11,DOCK4,MAST4,ITPR2,NRXN1,NTRK3,WDPCP,MAGI1,RAPGEF4,CNKSR2,ADCYAP1R1,EPHB1,PRICKLE2,GRID1,ZDHHC17,DCDC1,CACNA1C,PRKAA2,BBS2,FLT1,EXOC4,DLG2,PPP1R12B,RORA,PTPRD,CDC14B,MYO9A,FOXN3,NTRK2,GRIK1,PRKG1,DSCAM,DGKI,MAGI2,AKAP6,IQCJ-SCHIP1,RNF152,CNTN1,DOCK10,GRM5,ENPEP,PRKD1,NRG3,NLGN1,PTPRG,OR9Q1,GRID2,LRP2,SEMA6D,SYNE1,RALGPS2,FER,SNRK,RYR2,FBXL17,PRLR,PTPRA,G*

			RIK3,CACNB2,STXBP4,MAPRE2,SGCD,RIC8B,SEZ6L,ATAT1,RBMS3,RAPGEF2,ZMYND11,RAPGEF5,INSR,RGS6,SRGAP3,AKAP13,GABRG2,UNC13C,NRXN3,RALGAPA1,DLGAP2,NSG1,CPE,KCND2,SEMA5A,ARHGAP24,GRIA4,CNIH3,DOCK3,APC,GPC6,CHN1,RELN,ROR1,TNR,DTNA,CRB1,VAV3,PRKCA,INPP5A,FMN2,ERC1,AGO3,ALPK2,CCDC88A
GO:0010975	regulation of neuron projection development	1.0570619688874916e-11	*MACF1,NRP1,ABL2,LRRC4C,SLC39A12,DISC1,FYN,CSMD3,NEGR1,TOX,NRXN1,NTRK3,DNM3,PTPRD,NTRK2,DSCAM,MAGI2,CNTN1,PRKD1,NLGN1,PTPRG,GRID2,SEMA6D,SYNE1,MAP2,FAT3,RAPGEF2,SEMA5A,ATP8A2,CTNNA2,CHN1,RELN,ROR1,TNR,CCDC88A*
GO:0042391	regulation of membrane potential	1.2783855167420576e-11	*KCNMA1,SLC8A1,GRIK2,IGSF11,GRIK4,GABRG1,GABRB1,ANK2,GRIN2B,GRIA1,FGF12,CACNG2,ABCB5,NRXN1,GRID1,ABCC9,CACNA1C,NTRK2,GRIK1,DGKI,AKAP6,GRM5,NLGN1,GRID2,SYNE1,RYR2,GRIK3,CACNB2,GABRG2,KCND2,GRIA4,RELN,SLC4A4,KCNE4*
GO:0035556	intracellular signal transduction	1.3615603270895394e-11	*ERBB4,APBB2,ATRX,NRP1,TEAD1,CDH13,ABL2,SLC8A1,ANKRD6,RERG,LDLRAD4,STARD13,PIK3C3,DCLK1,CDC42EP3,GRIK2,RGL1,ITGA1,ASB3,PIK3R3,DISC1,RALGPS1,ARHGAP42,NDRG2,FYN,ANK2,GRIN2B,STK32A,FGF12,DGKB,S100B,ESR1,ARHGAP12,PDE4D,PRKACB,DOCK4,MAST4,NRXN1,NTRK3,RAPGEF4,CNKSR2,ADCYAP1R1,EPHB1,ZDHHC17,DCDC1,CACNA1C,PRKAA2,FLT1,RORA,CDC14B,MYO9A,FOXN3,NTRK2,PRKG1,DGKI,MAGI2,AKAP6,IQCJ-SCHIP1,RNF152,DOCK10,GRM5,PRKD1,NRG3,NLGN1,LRP2,RALGPS2,FER,SNRK,RYR2,MAPRE2,SGCD,SEZ6L,RAPGEF2,ZMYND11,RAPGEF5,INSR,RGS6,SRGAP3,AKAP13,RALGAPA1,SEMA5A,ARHGAP24,DOCK3,APC,CHN1,RELN,ROR1,VAV3,PRKCA,FMN2,ERC1,AGO3,CCDC88A*
GO:0031344	regulation of cell projection organization	1.5839465409103994e-11	*MACF1,NRP1,ABL2,LRRC4C,CDC42EP3,SLC39A12,DISC1,FYN,CSMD3,GRIN2B,NEGR1,TOX,NRXN1,NTRK3,WDPCP,DNM3,PTPRD,MYO9A,NTRK2,DSCAM,MAGI2,CNTN1,PRKD1,NLGN1,PTPRG,GRID2,SEMA6D,SYNE1,FER,MAP2,FAT3,RAPGEF2,SEMA5A,ATP8A2,ARHGAP24,CTNNA2,APC,CHN1,RELN,ROR1,TNR,CCDC88A*
GO:0120035	regulation of plasma membrane bounded cell projection organization	3.5777925462243896e-11	*MACF1,NRP1,ABL2,LRRC4C,CDC42EP3,SLC39A12,DISC1,FYN,CSMD3,GRIN2B,NEGR1,TOX,NRXN1,NTRK3,WDPCP,DNM3,PTPRD,NTRK2,DSCAM,MAGI2,CNTN1,PRKD1,NLGN1,PTPRG,GRID2,SEMA6D,SYNE1,FER,MAP2,FAT3,RAPGEF2,SEMA5A,ATP8A2,ARHGAP24,CTNNA2,APC,CHN1,RELN,ROR1,TNR,CCDC88A*
GO:0034329	cell	3.7075619838	*ERBB4,MACF1,CTNND2,NRP1,CDH13,CLS*

	junction assembly	87765e-11	*TN2,CORO2B,FMN1,PEAK1,ANK2,GRIA1,NEGR1,CDH11,NRXN1,WDPCP,EPHB1,DNM3,PTPRD,TLN2,MYO9A,NTRK2,DSCAM,NRG3,NLGN1,GRID2,FER,PTPRA,MPDZ,VCL,RAPGEF2,GABRG2,NRXN3,APC,PRKCA*
GO:0010646	regulation of cell communication	1.3224056399207177e-10	*ERBB4,MACF1,CTNND2,NRP1,CDH13,ABL2,SLC8A1,ANKRD6,LDLRAD4,STARD13,LRRC4C,GRIK2,IGSF11,NCAM1,ITGA1,CLSTN2,ITGA8,GRIK4,DISC1,RALGPS1,ARHGAP42,EYA1,GPR158,NDRG2,APBA2,FYN,ANK2,GRIN2B,GRIA1,FGF12,DGKB,CACNG2,S100B,ESR1,ARHGAP12,PDE4D,ERC2,PRKACB,LAMA1,CDH11,NRXN1,NTRK3,RAPGEF4,CNKSR2,ADCYAP1R1,EPHB1,GRID1,ZDHHC17,PRKAA2,FLT1,RORA,PTPRD,MYO9A,NTRK2,GRIK1,DGKI,MAGI2,AKAP6,IQCJ-SCHIP1,RNF152,GRM5,PRKD1,NRG3,NLGN1,GRID2,LRP2,SYNE1,FER,RYR2,FBXL17,PRLR,PTPRA,GRIK3,STXBP4,MAPRE2,RIC8B,SEZ6L,ATAT1,RBMS3,RAPGEF2,ZMYND11,INSR,RGS6,SRGAP3,AKAP13,UNC13C,NRXN3,RALGAPA1,NSG1,SEMA5A,ARHGAP24,GRIA4,CNIH3,DOCK3,APC,GPC6,CHN1,RELN,ROR1,TNR,VAV3,PRKCA,INPP5A,ERC1,AGO3,ALPK2,CCDC88A*
GO:0035249	synaptic transmission, glutamatergic	1.9022068427490215e-10	*GRIK2,GRIK4,DISC1,GRIN2B,GRIA1,CACNG2,NRXN1,GRID1,GRIK1,DGKI,GRM5,NLGN1,GRID2,GRIK3,UNC13C,GRIA4,RELN,TNR*
GO:0048667	cell morphogenesis involved in neuron differentiation	2.431164329503362e-10	*APBB2,MACF1,CTNND2,NRP1,LRRC4C,DCLK1,NCAM1,PHACTR1,DISC1,FYN,S100B,PCDH15,CDH11,NRXN1,WDPCP,EPHB1,ZDHHC17,DNM3,PTPRD,NTRK2,PRKG1,DSCAM,CNTN1,DOCK10,NLGN1,SEMA6D,SYNE1,MAP2,FAT3,VCL,RAPGEF2,NRXN3,SEMA5A,ATP8A2,CTNNA2,CHN1,RELN,TNR*
GO:0060078	regulation of postsynaptic membrane potential	2.8054833154741963e-10	*GRIK2,IGSF11,GRIK4,GABRG1,GABRB1,GRIN2B,GRIA1,NRXN1,GRID1,GRIK1,DGKI,GRM5,NLGN1,GRID2,SYNE1,GRIK3,GABRG2,KCND2,GRIA4,RELN*
GO:0007420	brain development	4.4364062595224383e-10	*ERBB4,ATRX,RTN1,NRP1,EML1,DCLK1,KDM4B,PBX1,PHACTR1,DISC1,GPR158,NDRG2,FYN,SLC1A2,GRIN2B,GRIA1,TOX,BRINP1,NRXN1,EPHB1,BBS2,RORA,NTRK2,PRKG1,CNTN1,CA10,NRG3,GRID2,LRP2,SEMA6D,MTPN,SOX6,PCDH9,ATAT1,RAPGEF2,IMMP2L,ATXN1,SEMA5A,CTNNA2,RELN,TNR,ARNT2*
GO:0032989	cellular anatomical entity morphogenesis	8.901224388020229e-10	*APBB2,MACF1,CTNND2,NRP1,LRRC4C,DCLK1,NCAM1,ITGA1,PHACTR1,SLC39A12,DISC1,FYN,ANK2,S100B,CDH11,NRXN1,EPHB1,ZDHHC17,DNM3,PTPRD,MYO9A,NTRK2,PRKG1,DSCAM,CNTN1,DOCK10,PRKD1,NLGN1,LRP2,SEMA6D,SYNE1,MAP2,VCL,RAPGEF2,AKAP13,NRXN3,PGM5,SEMA5*

			A,ATP8A2,CTNNA2,CHN1,RELN,TNR
GO:0048812	neuron projection morphogenesis	9.281934919124884e-10	*APBB2,MACF1,CTNND2,NRP1,LRRC4C,DCLK1,NCAM1,ITGA1,PHACTR1,SLC39A12,DISC1,FYN,S100B,CDH11,NRXN1,EPHB1,ZDHHC17,DNM3,PTPRD,MYO9A,NTRK2,PRKG1,DSCAM,CNTN1,DOCK10,NLGN1,LRP2,SEMA6D,SYNE1,MAP2,VCL,RAPGEF2,NRXN3,SEMA5A,ATP8A2,CTNNA2,CHN1,RELN,TNR*
GO:0007417	central nervous system development	1.0590359417733588e-9	*ERBB4,ATRX,RTN1,NRP1,MDGA2,EML1,DCLK1,KDM4B,PBX1,PHACTR1,DISC1,GPR158,GABRB1,NDRG2,FYN,SLC1A2,GRIN2B,GRIA1,S100B,TOX,CDH11,BRINP1,NRXN1,EPHB1,BBS2,RORA,NTRK2,GRIK1,PRKG1,CNTN1,CA10,NRG3,GRID2,LRP2,SEMA6D,MAP2,MTPN,SOX6,PCDH9,VCAN,ATAT1,RAPGEF2,IMMP2L,ATXN1,SEMA5A,CTNNA2,RELN,ROR1,TNR,ARNT2*
GO:0050794	regulation of cellular process	1.6951900595657904e-9	*SAMD4A,ERBB4,KCNMA1,PKNOX2,ZHX3,APBB2,ATRX,ADAMTS18,MACF1,CTNND2,MITF,TRIM23,MED13L,NRP1,TEAD1,NFIA,CDH13,ABL2,UTRN,MAML2,SLC8A1,DACH1,ANKRD6,RERG,SIAH3,CTIF,TBC1D5,FRMD5,LDLRAD4,STARD13,SLC40A1,LRRC4C,MALRD1,SLCO3A1,PIK3C3,DCLK1,CDC42EP3,GRIK2,IGSF11,RGL1,NRIP1,NCAM1,ITGA1,CLSTN2,TCF12,CCNG2,KDM4B,GNG12,TANC1,CORO2B,ITGA8,VPS41,GRIK4,ZBTB20,ZNF407,ASB3,TCF4,PBX1,PHACTR1,PIK3R3,SLC39A12,DISC1,FMN1,RALGPS1,ARHGAP42,PEAK1,EYA1,GPR158,GABRB1,NDRG2,APBA2,ASAP1,ST8SIA1,FYN,ANK2,SLC1A2,CADPS,CSMD3,GRIN2B,PHC2,STK32A,GRIA1,NEGR1,FGF12,DGKB,CACNG2,EBF1,S100B,TOX,ESR1,ARHGAP12,KLF12,PDE4D,ERC2,PRKACB,GNG2,FCHSD2,LAMA1,CDH11,SETBP1,DOCK4,MAST4,ITPR2,BRINP1,NRXN1,NTRK3,BCL2L13,WDPCP,MAGI1,RAPGEF4,CNKSR2,ADCYAP1R1,EPHB1,PRICKLE2,DPP6,CTTNBP2,GRID1,CREB5,TNRC6B,ZDHHC17,GSG1L,DCDC1,CACNA1C,PRKAA2,DNM3,BBS2,ASXL3,DPF3,FLT1,EDIL3,EXOC4,PPP1R12B,SACS,RORA,PTPRD,CDC14B,MYO9A,FOXN3,NTRK2,LOXL2,GRIK1,SOX5,PRKG1,DSCAM,DGKI,FTO,NBEA,MXI1,TTC28,MAGI2,NELL1,AKAP6,IQCJ-SCHIP1,RNF152,CNTN1,DOCK10,GRM5,PLEKHB2,LDB2,PRKD1,NRG3,ATP8A1,NLGN1,PTPRG,OR9Q1,JAM2,GRID2,LRP2,SEMA6D,SYNE1,RALGPS2,FER,SNRK,RYR2,FBXL17,MAP2,PRLR,PTPRA,FAT3,GRIK3,CACNB2,STXBP4,MED15,MTPN,MAPRE2,SOX6,SGCD,PDE4DIP,RIC8B,MPDZ,VCL,SEZ6L,VCAN,ATAT1,RBMS3,RAPGEF2,ZMYND11,RAPGEF5,INSR,RGS6,SRGAP3,AKAP13,ATXN1,UNC13C,NRXN3,RALGAPA1,NSG1,CLIP1,CPE,SEMA5A,CABLES1,ATP*

			8A2,SLC24A3,ARHGAP24,GRIA4,CNIH3,DOCK3,CTNNA2,APC,GPC6,CHN1,RELN,VPS13C,TRPS1,ROR1,SLC4A4,TNR,DTNA,VAV3,NPAS3,PRKCA,KMT2C,INPP5A,ZNF678,FMN2,ERC1,AGO3,ALPK2,CCDC88A,ARNT2,AFF3
GO:0120039	plasma membrane bounded cell projection morphogenesis	1.9703533990876688e-9	*APBB2,MACF1,CTNND2,NRP1,LRRC4C,DCLK1,NCAM1,ITGA1,PHACTR1,SLC39A12,DISC1,FYN,S100B,CDH11,NRXN1,EPHB1,ZDHHC17,DNM3,PTPRD,MYO9A,NTRK2,PRKG1,DSCAM,CNTN1,DOCK10,NLGN1,LRP2,SEMA6D,SYNE1,MAP2,VCL,RAPGEF2,NRXN3,SEMA5A,ATP8A2,CTNNA2,CHN1,RELN,TNR*
GO:0048858	cell projection morphogenesis	2.4806987769238918e-9	*APBB2,MACF1,CTNND2,NRP1,LRRC4C,DCLK1,NCAM1,ITGA1,PHACTR1,SLC39A12,DISC1,FYN,S100B,CDH11,NRXN1,EPHB1,ZDHHC17,DNM3,PTPRD,MYO9A,NTRK2,PRKG1,DSCAM,CNTN1,DOCK10,NLGN1,LRP2,SEMA6D,SYNE1,MAP2,VCL,RAPGEF2,NRXN3,SEMA5A,ATP8A2,CTNNA2,CHN1,RELN,TNR*
GO:0000902	cell morphogenesis	2.5073006938521696e-9	*APBB2,MACF1,CTNND2,NRP1,NFIA,FRY,CDH13,LRRC4C,DCLK1,CDC42EP3,NCAM1,ITGA1,FAM171A1,PHACTR1,SLC39A12,DISC1,FYN,S100B,PCDH15,CDH11,NRXN1,WDPCP,EPHB1,ZDHHC17,DNM3,PTPRD,MYO9A,NTRK2,PRKG1,DSCAM,CNTN1,DOCK10,NLGN1,LRP2,SEMA6D,SYNE1,MAP2,FAT3,VCL,RAPGEF2,NRXN3,SEMA5A,ATP8A2,CTNNA2,CHN1,RELN,TNR,CRB1*
GO:0060322	head development	4.024831048960218e-9	*ERBB4,ATRX,RTN1,NRP1,EML1,DCLK1,KDM4B,PBX1,PHACTR1,DISC1,GPR158,NDRG2,FYN,SLC1A2,GRIN2B,GRIA1,TOX,BRINP1,NRXN1,EPHB1,BBS2,RORA,NTRK2,PRKG1,CNTN1,CA10,NRG3,GRID2,LRP2,SEMA6D,MTPN,SOX6,PCDH9,ATAT1,RAPGEF2,IMMP2L,ATXN1,SEMA5A,CTNNA2,RELN,TNR,ARNT2*
GO:0023051	regulation of signaling	4.990709674957525e-9	*ERBB4,MACF1,CTNND2,NRP1,CDH13,ABL2,ANKRD6,LDLRAD4,STARD13,LRRC4C,GRIK2,IGSF11,NCAM1,ITGA1,CLSTN2,ITGA8,GRIK4,DISC1,RALGPS1,ARHGAP42,EYA1,GPR158,NDRG2,APBA2,FYN,GRIN2B,GRIA1,DGKB,CACNG2,S100B,ESR1,ARHGAP12,PDE4D,ERC2,PRKACB,LAMA1,CDH11,NRXN1,NTRK3,RAPGEF4,CNKSR2,ADCYAP1R1,EPHB1,GRID1,ZDHHC17,PRKAA2,FLT1,RORA,PTPRD,MYO9A,NTRK2,GRIK1,DGKI,MAGI2,AKAP6,IQCJ-SCHIP1,RNF152,GRM5,PRKD1,NRG3,NLGN1,GRID2,LRP2,SYNE1,FER,FBXL17,PRLR,PTPRA,GRIK3,STXBP4,MAPRE2,RIC8B,SEZ6L,ATAT1,RBMS3,RAPGEF2,ZMYND11,INSR,RGS6,SRGAP3,AKAP13,UNC13C,NRXN3,RALGAPA1,NSG1,SEMA5A,ARHGAP24,GRIA4,CNIH3,DOCK3,APC,GPC6,CHN1,RELN,ROR1,TNR,VAV3,PRKCA,INPP5A,ERC1,AGO3,ALPK2,CCDC88A*

GO:0003008	system process	6.919975438253169e-9	*KCNMA1,APBB2,UTRN,SLC8A1,SLC2A13,SLCO3A1,HMCN1,GRIK2,IGSF11,ITGA1,CLSTN2,TANC1,CORO2B,ITGA8,ASB3,DISC1,ARHGAP42,EYA1,GPR158,FYN,ANK2,SLC1A2,GRIN2B,GRIA1,FGF12,CACNG2,S100B,PCDH15,PDE4D,LRIG1,PRKACB,DOCK4,BRINP1,NRXN1,EPHB1,ABCC9,CACNA1C,BBS2,PPP1R12B,MYO9A,NTRK2,PRKG1,DGKI,FTO,AKAP6,GRM5,ENPEP,PRKD1,ATP8A1,NLGN1,OR9Q1,JAM2,GRID2,LRP2,SYNE1,RYR2,CACNB2,MTPN,SGCD,INSR,AKAP13,IMMP2L,ATXN1,NRXN3,KCND2,ATP8A2,CALD1,SLC24A3,LHFPL3,CTNNA2,RELN,ROR1,SLC4A4,TNR,DTNA,CRB1,PRKCA,KCNE4*
GO:0051128	regulation of cellular component organization	1.3162343004460935e-8	*ATRX,MACF1,NRP1,TEAD1,CDH13,ABL2,RERG,TBC1D5,LRRC4C,CDC42EP3,CLSTN2,TANC1,CORO2B,VPS41,SLC39A12,DISC1,FMN1,PEAK1,GPR158,ASAP1,FYN,CSMD3,GRIN2B,NEGR1,DGKB,TOX,ERC2,FCHSD2,LAMA1,NRXN1,NTRK3,WDPCP,EPHB1,CTTNBP2,GRID1,GSG1L,PRKAA2,DNM3,DPF3,SACS,PTPRD,MYO9A,NTRK2,DSCAM,MAGI2,AKAP6,IQCJ-SCHIP1,CNTN1,PRKD1,NRG3,ATP8A1,NLGN1,PTPRG,GRID2,SEMA6D,SYNE1,FER,MAP2,PTPRA,FAT3,MTPN,MAPRE2,PDE4DIP,MPDZ,VCL,ATAT1,RAPGEF2,INSR,AKAP13,CLIP1,SEMA5A,ATP8A2,ARHGAP24,CTNNA2,APC,CHN1,RELN,VPS13C,ROR1,TNR,CCDC88A*
GO:0141124	intracellular signaling cassette	2.0866936236319476e-8	*ERBB4,NRP1,CDH13,ABL2,SLC8A1,ANKRD6,RERG,LDLRAD4,STARD13,CDC42EP3,GRIK2,RGL1,ITGA1,PIK3R3,RALGPS1,ARHGAP42,NDRG2,FYN,ANK2,GRIN2B,FGF12,S100B,ESR1,ARHGAP12,PDE4D,DOCK4,NRXN1,NTRK3,RAPGEF4,ADCYAP1R1,EPHB1,ZDHHC17,CACNA1C,FLT1,RORA,MYO9A,NTRK2,PRKG1,DGKI,MAGI2,AKAP6,DOCK10,GRM5,PRKD1,LRP2,RALGPS2,FER,RYR2,MAPRE2,SGCD,RAPGEF2,ZMYND11,RAPGEF5,INSR,SRGAP3,AKAP13,RALGAPA1,SEMA5A,ARHGAP24,DOCK3,CHN1,RELN,ROR1,VAV3,PRKCA,ERC1,AGO3,CCDC88A*
GO:0030154	cell differentiation	2.6956311658411362e-8	*ERBB4,ZHX3,APBB2,ATRX,MACF1,CTNND2,MITF,RTN1,NRP1,NFIA,FRY,MDGA2,ABL2,SLC8A1,EML1,LDLRAD4,LRRC4C,MYEF2,DCLK1,SLC9C1,NCAM1,ITGA1,TCF12,TANC1,ITGA8,TCF4,PBX1,PHACTR1,PIK3R3,SLC39A12,DISC1,EYA1,GABRB1,NDRG2,ASAP1,FYN,ANK2,CSMD3,NEGR1,S100B,TOX,PCDH15,ESR1,ABCB5,PDE4D,LAMA1,CDH11,BRINP1,NRXN1,NTRK3,WDPCP,ADCYAP1R1,EPHB1,ZDHHC17,DNM3,BBS2,DPF3,FLT1,RORA,PTPRD,MYO9A,NTRK2,LOXL2,SOX5,PRKG1,DSCAM,FTO,THSD7A,NCAM2,MAGI2,NELL1,AKAP6,CNTN1,DOCK10,OPCML,GRM5,PLEKHB2,PRK*

			D1,NRG3,NLGN1,PTPRG,JAM2,GRID2,LRP2,SEMA6D,SYNE1,FER,SNRK,FBXL17,MAP2,PRLR,FAT3,MTPN,SOX6,SGCD,VCL,VCAN,ATAT1,RAPGEF2,AKAP13,NRXN3,PGM5,ASTN1,SEMA5A,ATP8A2,ARHGAP24,CTNNA2,APC,CHN1,RELN,TRPS1,ROR1,KAZN,TNR,CRB1,PRKCA,FMN2,ALPK2,CCDC88A
GO:0048869	cellular developmental process	2.737995198807853e-8	*ERBB4,ZHX3,APBB2,ATRX,MACF1,CTNND2,MITF,RTN1,NRP1,NFIA,FRY,MDGA2,ABL2,SLC8A1,EML1,LDLRAD4,LRRC4C,MYEF2,DCLK1,SLC9C1,NCAM1,ITGA1,TCF12,TANC1,ITGA8,TCF4,PBX1,PHACTR1,PIK3R3,SLC39A12,DISC1,EYA1,GABRB1,NDRG2,ASAP1,FYN,ANK2,CSMD3,NEGR1,S100B,TOX,PCDH15,ESR1,ABCB5,PDE4D,LAMA1,CDH11,BRINP1,NRXN1,NTRK3,WDPCP,ADCYAP1R1,EPHB1,ZDHHC17,DNM3,BBS2,DPF3,FLT1,RORA,PTPRD,MYO9A,NTRK2,LOXL2,SOX5,PRKG1,DSCAM,FTO,THSD7A,NCAM2,MAGI2,NELL1,AKAP6,CNTN1,DOCK10,OPCML,GRM5,PLEKHB2,PRKD1,NRG3,NLGN1,PTPRG,JAM2,GRID2,LRP2,SEMA6D,SYNE1,FER,SNRK,FBXL17,MAP2,PRLR,FAT3,MTPN,SOX6,SGCD,VCL,VCAN,ATAT1,RAPGEF2,AKAP13,NRXN3,PGM5,ASTN1,SEMA5A,ATP8A2,ARHGAP24,CTNNA2,APC,CHN1,RELN,TRPS1,ROR1,KAZN,TNR,CRB1,PRKCA,FMN2,ALPK2,CCDC88A*
GO:0099173	postsynapse organization	6.74412148981806e-8	*CTNND2,NRP1,TANC1,DISC1,ASAP1,FYN,GRIN2B,DGKB,NRXN1,CNKSR2,EPHB1,GRID1,DNM3,PTPRD,DOCK10,NLGN1,GRID2,INSR,NRXN3,RELN*
GO:0035235	ionotropic glutamate receptor signaling pathway	9.606799111371116e-8	*GRIK2,GRIK4,GRIN2B,GRIA1,GRID1,GRIK1,GRID2,GRIK3,GRIA4*
GO:0016477	cell migration	9.80951375583242e-8	*ERBB4,APBB2,MACF1,MITF,NRP1,CDH13,ABL2,DACH1,FRMD5,LDLRAD4,STARD13,DCLK1,ITGA1,PHACTR1,PIK3R3,DISC1,PEAK1,FYN,LAMA1,CDH11,DOCK4,NTRK3,WDPCP,EPHB1,FLT1,NTRK2,LOXL2,PRKG1,MAGI2,DOCK10,ENPEP,LDB2,PRKD1,NRG3,ATP8A1,PTPRG,JAM2,SEMA6D,FER,FAT3,MAPRE2,VCL,VCAN,RAPGEF2,INSR,SRGAP3,ASTN1,SEMA5A,ARHGAP24,CTNNA2,APC,GPC6,RELN,TNR,VAV3,PRKCA,FMN2,CCDC88A*
GO:0007165	signal transduction	2.146327827615588e-7	*ERBB4,APBB2,ATRX,ADAMTS18,MACF1,CTNND2,MITF,NRP1,TEAD1,NFIA,CDH13,ABL2,MAML2,SLC8A1,ANKRD6,RERG,LDLRAD4,STARD13,PIK3C3,DCLK1,CDC42EP3,GRIK2,IGSF11,RGL1,NCAM1,ITGA1,GNG12,ITGA8,GRIK4,ASB3,PIK3R3,SLC39A12,DISC1,RALGPS1,ARHGAP42,EYA1,GPR158,GABRB1,NDRG2,FYN,ANK2,GRIN2B,STK32A,GRIA1,FGF12,DGKB,CACNG2*

			,S100B,ESR1,ARHGAP12,PDE4D,PRKACB,GNG2,LAMA1,DOCK4,MAST4,ITPR2,NRXN1,NTRK3,WDPCP,MAGI1,RAPGEF4,CNKSR2,ADCYAP1R1,EPHB1,PRICKLE2,GRID1,ZDHHC17,DCDC1,CACNA1C,PRKAA2,BBS2,FLT1,PPP1R12B,RORA,PTPRD,CDC14B,MYO9A,FOXN3,NTRK2,GRIK1,PRKG1,DSCAM,DGKI,MAGI2,AKAP6,IQCJ-SCHIP1,RNF152,CNTN1,DOCK10,GRM5,PRKD1,NRG3,NLGN1,PTPRG,OR9Q1,GRID2,LRP2,SEMA6D,SYNE1,RALGPS2,FER,SNRK,RYR2,FBXL17,PRLR,PTPRA,GRIK3,STXBP4,MAPRE2,SGCD,RIC8B,SEZ6L,ATAT1,RBMS3,RAPGEF2,ZMYND11,RAPGEF5,INSR,RGS6,SRGAP3,AKAP13,NRXN3,RALGAPA1,NSG1,CPE,SEMA5A,ARHGAP24,GRIA4,CNIH3,DOCK3,APC,GPC6,CHN1,RELN,ROR1,DTNA,VAV3,PRKCA,INPP5A,FMN2,ERC1,AGO3,ALPK2,CCDC88A
GO:0016358	dendrite development	2.76988259803178e-7	*CTNND2,NRP1,DCLK1,PHACTR1,DISC1,ASAP1,FYN,CSMD3,EPHB1,DNM3,PTPRD,PRKG1,DSCAM,DOCK10,NLGN1,SYNE1,MAP2,FAT3,RAPGEF2,CTNNA2,RELN*
GO:0048468	cell development	2.8344370789338016e-7	*ERBB4,APBB2,ATRX,MACF1,CTNND2,MITF,NRP1,FRY,ABL2,SLC8A1,EML1,LRRC4C,DCLK1,NCAM1,ITGA1,PBX1,PHACTR1,PIK3R3,SLC39A12,DISC1,GABRB1,ASAP1,FYN,ANK2,CSMD3,NEGR1,S100B,TOX,PCDH15,ESR1,PDE4D,LAMA1,CDH11,BRINP1,NRXN1,NTRK3,WDPCP,EPHB1,ZDHHC17,DNM3,BBS2,RORA,PTPRD,MYO9A,NTRK2,PRKG1,DSCAM,NCAM2,MAGI2,AKAP6,CNTN1,DOCK10,OPCML,GRM5,PRKD1,NLGN1,PTPRG,JAM2,GRID2,LRP2,SEMA6D,SYNE1,FER,SNRK,MAP2,FAT3,SGCD,VCL,VCAN,ATAT1,RAPGEF2,AKAP13,NRXN3,PGM5,SEMA5A,ATP8A2,CTNNA2,CHN1,RELN,ROR1,TNR,CRB1,PRKCA,FMN2,ALPK2,CCDC88A*
GO:0007610	behavior	4.2428867032037527e-7	*ABL2,DACH1,GRIK2,CLSTN2,TANC1,ITGA8,APBA2,FYN,SLC1A2,GRIN2B,GRIA1,NEGR1,FGF12,S100B,PCDH15,BRINP1,NRXN1,GRID1,BBS2,NTRK2,DSCAM,DGKI,CNTN1,GRM5,ATP8A1,NLGN1,INSR,ATXN1,GABRG2,NRXN3,ASTN1,KCND2,ATP8A2,RELN,TNR*
GO:0007215	glutamate receptor signaling pathway	4.36136997194045e-7	*GRIK2,GRIK4,FYN,GRIN2B,GRIA1,GRID1,GRIK1,GRM5,GRID2,GRIK3,GRIA4*
GO:0031346	positive regulation of cell projection organization	6.673601216077046e-7	*MACF1,NRP1,ABL2,CDC42EP3,DISC1,FYN,NEGR1,TOX,NRXN1,NTRK3,DNM3,PTPRD,NTRK2,DSCAM,MAGI2,CNTN1,PRKD1,NLGN1,RAPGEF2,SEMA5A,ATP8A2,APC,RELN,ROR1,CCDC88A*
GO:0048813	dendrite morphogenesis	0.000001303769847761657	*CTNND2,NRP1,DCLK1,PHACTR1,FYN,EPHB1,DNM3,PTPRD,DSCAM,DOCK10,NLGN1,SYNE1,MAP2,RAPGEF2,CTNNA2,RELN*

GO:0003012	muscle system process	0.0000013672212273203492	*KCNMA1,APBB2,UTRN,SLC8A1,ASB3,ARHGAP42,ANK2,FGF12,PDE4D,DOCK4,ABCC9,CACNA1C,BBS2,PPP1R12B,PRKG1,AKAP6,PRKD1,RYR2,CACNB2,MTPN,SGCD,KCND2,ATP8A2,CALD1,DTNA,PRKCA,KCNE4*
GO:0006936	muscle contraction	0.0000014667196381866337	*KCNMA1,APBB2,UTRN,SLC8A1,ASB3,ARHGAP42,ANK2,FGF12,PDE4D,DOCK4,ABCC9,CACNA1C,BBS2,PPP1R12B,PRKG1,PRKD1,RYR2,CACNB2,SGCD,KCND2,ATP8A2,CALD1,DTNA,KCNE4*
GO:0007409	axonogenesis	0.0000018504248561084967	*APBB2,MACF1,NRP1,LRRC4C,DCLK1,NCAM1,DISC1,FYN,S100B,CDH11,NRXN1,EPHB1,ZDHHC17,PTPRD,NTRK2,PRKG1,DSCAM,CNTN1,SEMA6D,MAP2,VCL,NRXN3,SEMA5A,ATP8A2,CTNNA2,CHN1,RELN,TNR*
GO:0048870	cell motility	0.0000021502027936913314	*ERBB4,APBB2,MACF1,MITF,NRP1,CDH13,ABL2,DACH1,FRMD5,LDLRAD4,STARD13,DCLK1,SLC9C1,ITGA1,PHACTR1,PIK3R3,DISC1,PEAK1,FYN,LAMA1,CDH11,DOCK4,NTRK3,WDPCP,EPHB1,BBS2,FLT1,NTRK2,LOXL2,PRKG1,MAGI2,DOCK10,ENPEP,LDB2,PRKD1,NRG3,ATP8A1,PTPRG,JAM2,SEMA6D,FER,FAT3,MAPRE2,VCL,VCAN,RAPGEF2,INSR,SRGAP3,ASTN1,SEMA5A,ARHGAP24,CTNNA2,APC,GPC6,RELN,TNR,VAV3,PRKCA,FMN2,CCDC88A*
GO:0050807	regulation of synapse organization	0.0000024472136043285076	*CLSTN2,TANC1,DISC1,GPR158,ASAP1,FYN,GRIN2B,NEGR1,DGKB,NRXN1,EPHB1,CTTNBP2,GRID1,DNM3,PTPRD,NTRK2,NLGN1,GRID2,CTNNA2,RELN*
GO:1990806	ligand-gated ion channel signaling pathway	0.000002510263881523522	*GRIK2,GRIK4,GRIN2B,GRIA1,GRID1,GRIK1,GRID2,GRIK3,GRIA4*
GO:0007264	small GTPase-mediated signal transduction	0.000002708563556413108	*NRP1,CDH13,ABL2,RERG,STARD13,CDC42EP3,RGL1,RALGPS1,ARHGAP42,ARHGAP12,DOCK4,RAPGEF4,ADCYAP1R1,MYO9A,DGKI,DOCK10,RALGPS2,MAPRE2,RAPGEF2,RAPGEF5,SRGAP3,AKAP13,RALGAPA1,ARHGAP24,DOCK3,CHN1,RELN,VAV3,CCDC88A*
GO:0072359	circulatory system development	0.0000029293718601619894	*ERBB4,NRP1,CDH13,SLC8A1,STARD13,PIK3R3,SLC39A12,EYA1,ANK2,FGF12,LAMA1,CDH11,NRXN1,NTRK3,WDPCP,EPHB1,GREB1L,ABCC9,CACNA1C,FLT1,RORA,NTRK2,LOXL2,PRKG1,THSD7A,AKAP6,ENPEP,PRKD1,LRP2,SYNE1,RYR2,SOX6,SGCD,RAPGEF2,INSR,AKAP13,IMMP2L,NRXN3,CPE,SEMA5A,CALD1,ARHGAP24,APC,VAV3,PRKCA,ALPK2*
GO:0044057	regulation of system process	0.000003138367636966731	*SLC8A1,GRIK2,IGSF11,CORO2B,ASB3,ARHGAP42,ANK2,GRIN2B,FGF12,PDE4D,DOCK4,NRXN1,ABCC9,CACNA1C,PPP1R12B,PRKG1,FTO,AKAP6,PRKD1,NLGN1,JAM2,SYNE1,RYR2,CACNB2,MTPN,KCND2,RELN,TNR,PRKCA,KCNE4*
GO:0050803	regulation of synapse	0.000004022466057793396	*CLSTN2,TANC1,DISC1,GPR158,ASAP1,FYN,GRIN2B,NEGR1,DGKB,NRXN1,EPHB1,CTTNBP2,GRID1,DNM3,PTPRD,NTRK2,NL*

	structure or activity		GN1,GRID2,CTNNA2,RELN
GO:0051966	regulation of synaptic transmission, glutamatergic	0.000006018709200952947	GRIK2,DISC1,GRIN2B,CACNG2,NRXN1,GRIK1,DGKI,GRM5,NLGN1,GRIK3,RELN,TNR
GO:0003013	circulatory system process	0.000006200808511794539	KCNMA1,SLC8A1,SLC2A13,SLCO3A1,ITGA1,CORO2B,ASB3,ARHGAP42,FYN,ANK2,SLC1A2,FGF12,PDE4D,DOCK4,ABCC9,CACNA1C,BBS2,PRKG1,ENPEP,ATP8A1,LRP2,RYR2,CACNB2,SGCD,INSR,AKAP13,IMMP2L,KCND2,SLC24A3,SLC4A4,KCNE4
GO:0010976	positive regulation of neuron projection development	0.000006328159637756768	ABL2,DISC1,FYN,NEGR1,TOX,NRXN1,NTRK3,NTRK2,MAGI2,CNTN1,PRKD1,NLGN1,RAPGEF2,ATP8A2,RELN,ROR1
GO:0061564	axon development	0.000006820912787406052	APBB2,MACF1,NRP1,LRRC4C,DCLK1,NCAM1,DISC1,FYN,S100B,CDH11,NRXN1,EPHB1,ZDHHC17,PTPRD,NTRK2,PRKG1,DSCAM,NCAM2,CNTN1,SEMA6D,MAP2,VCL,NRXN3,SEMA5A,ATP8A2,CTNNA2,CHN1,RELN,TNR
GO:0051716	cellular response to stimulus	0.000008051544783713974	ERBB4,APBB2,ATRX,ADAMTS18,MACF1,CTNND2,MITF,NRP1,TEAD1,NFIA,CDH13,ABL2,MAML2,SLC8A1,ANKRD6,RERG,LDLRAD4,STARD13,PIK3C3,DCLK1,CDC42EP3,GRIK2,IGSF11,RGL1,NRIP1,NCAM1,ITGA1,GNG12,CORO2B,ITGA8,VPS41,GRIK4,ZBTB20,ASB3,PIK3R3,SLC39A12,DISC1,RALGPS1,ARHGAP42,EYA1,GPR158,GABRB1,NDRG2,ST8SIA1,FYN,ANK2,SLC1A2,GRIN2B,STK32A,GRIA1,FGF12,DGKB,CACNG2,S100B,ESR1,ARHGAP12,PDE4D,PRKACB,GNG2,LAMA1,DOCK4,MAST4,ITPR2,BRINP1,NRXN1,NTRK3,WDPCP,MAGI1,RAPGEF4,CNKSR2,ADCYAP1R1,EPHB1,PRICKLE2,GRID1,ZDHHC17,DCDC1,CACNA1C,PRKAA2,BBS2,DPF3,FLT1,DLG2,PPP1R12B,RORA,PTPRD,CDC14B,MYO9A,FOXN3,NTRK2,GRIK1,PRKG1,DSCAM,DGKI,FTO,MAGI2,AKAP6,IQCJ-SCHIP1,RNF152,CNTN1,DOCK10,GRM5,PRKD1,NRG3,NLGN1,PTPRG,OR9Q1,GRID2,LRP2,SEMA6D,SYNE1,RALGPS2,FER,SNRK,RYR2,FBXL17,PRLR,PTPRA,GRIK3,STXBP4,MTPN,MAPRE2,SGCD,MSRA,RIC8B,SEZ6L,ATAT1,RBMS3,RAPGEF2,ZMYND11,RAPGEF5,INSR,RGS6,SRGAP3,AKAP13,IMMP2L,CHCHD6,GABRG2,NRXN3,RALGAPA1,NSG1,CPE,KCND2,SEMA5A,ARHGAP24,GRIA4,CNIH3,DOCK3,APC,GPC6,CHN1,RELN,VPS13C,ROR1,TNR,DTNA,CRB1,VAV3,PRKCA,INPP5A,FMN2,ERC1,AGO3,ALPK2,CCDC88A
GO:0030334	regulation	0.0000095824	ERBB4,MACF1,MITF,NRP1,CDH13,ABL2,

	of cell migration	37430533792	*DACH1,FRMD5,LDLRAD4,STARD13,PHACTR1,PIK3R3,LAMA1,CDH11,DOCK4,NTRK3,WDPCP,FLT1,PRKG1,MAGI2,DOCK10,LDB2,PRKD1,NRG3,ATP8A1,PTPRG,JAM2,SEMA6D,FER,MAPRE2,VCL,RAPGEF2,INSR,SRGAP3,SEMA5A,CTNNA2,APC,RELN,TNR,PRKCA*
GO:0034220	monoatomic ion transmembrane transport	0.000009763717432480526	*KCNMA1,UTRN,SLC8A1,SLC40A1,SLC9C1,GRIK2,GRIK4,GABRG1,SLC39A12,GABRB1,FYN,ANK2,SLC1A2,GRIN2B,GRIA1,FGF12,CACNG2,KCNN3,PDE4D,ITPR2,ADCYAP1R1,DPP6,GRID1,ABCC9,CACNA1C,GRIK1,AKAP6,GRM5,PRKD1,ATP8A1,GRID2,RYR2,GRIK3,CACNB2,GABRG2,KCND2,SLC24A3,GRIA4,RELN,SLC4A4,KCNE4,KCNS3*
GO:0097120	receptor localization to synapse	0.000010085384546317328	*ERBB4,CACNG2,NRXN1,DLG2,NBEA,NLGN1,NRXN3,NSG1,GPC6,RELN,CEP112*
GO:0098609	cell-cell adhesion	0.000013707939986257894	*LPP,ADAMTS18,CTNND2,CDH13,ABL2,PCDH7,LRRC4C,CADM2,HMCN1,IGSF11,ITGA1,CLSTN2,ITGA8,FYN,NEGR1,PCDH15,MEGF11,CDH11,NRXN1,MAGI1,DLG2,PTPRD,TLN2,PRKG1,DSCAM,NCAM2,CNTN1,NLGN1,JAM2,GRID2,FER,FAT3,PCDH9,VCL,NRXN3,ASTN1,CTNNA2,TNR,CRB1,PRKCA*
GO:0048522	positive regulation of cellular process	0.000013997624392621482	*SAMD4A,ERBB4,KCNMA1,ZHX3,APBB2,ATRX,MACF1,MITF,TRIM23,NRP1,TEAD1,NFIA,CDH13,ABL2,UTRN,MAML2,ANKRD6,CTIF,TBC1D5,FRMD5,SLC40A1,SLCO3A1,CDC42EP3,GRIK2,IGSF11,NRIP1,ITGA1,CLSTN2,TCF12,CORO2B,ITGA8,ZBTB20,ZNF407,TCF4,PBX1,PIK3R3,DISC1,FMN1,EYA1,GPR158,ASAP1,ST8SIA1,FYN,ANK2,SLC1A2,CADPS,GRIN2B,GRIA1,NEGR1,CACNG2,S100B,TOX,ESR1,KLF12,PDE4D,ERC2,FCHSD2,LAMA1,DOCK4,BRINP1,NRXN1,NTRK3,MAGI1,RAPGEF4,ADCYAP1R1,EPHB1,CREB5,TNRC6B,ZDHHC17,PRKAA2,DNM3,ASXL3,DPF3,FLT1,EDIL3,RORA,PTPRD,CDC14B,NTRK2,LOXL2,SOX5,DSCAM,DGKI,FTO,MAGI2,NELL1,AKAP6,IQCJ-SCHIP1,RNF152,CNTN1,GRM5,LDB2,PRKD1,ATP8A1,NLGN1,JAM2,GRID2,LRP2,SEMA6D,SYNE1,FER,RYR2,MAP2,PRLR,CACNB2,STXBP4,MED15,MTPN,MAPRE2,SOX6,PDE4DIP,VCAN,ATAT1,RBMS3,RAPGEF2,INSR,AKAP13,NSG1,CLIP1,SEMA5A,ATP8A2,SLC24A3,APC,RELN,ROR1,SLC4A4,TNR,VAV3,NPAS3,PRKCA,KMT2C,FMN2,AGO3,CCDC88A,ARNT2*
GO:0099175	regulation of postsynapse organization	0.000015214731121117651	*TANC1,DISC1,ASAP1,FYN,GRIN2B,DGKB,NRXN1,GRID1,DNM3,PTPRD,NLGN1,GRID2,RELN*
GO:2000145	regulation	0.0000186412	*ERBB4,MACF1,MITF,NRP1,CDH13,ABL2,DACH1,FRMD5,LDLRAD4,STARD13,PHACT*

	of cell motility	95712171672	*R1,PIK3R3,LAMA1,CDH11,DOCK4,NTRK3,WDPCP,BBS2,FLT1,PRKG1,MAGI2,DOCK10,LDB2,PRKD1,NRG3,ATP8A1,PTPRG,JAM2,SEMA6D,FER,MAPRE2,VCL,RAPGEF2,INSR,SRGAP3,SEMA5A,CTNNA2,APC,RELN,TNR,PRKCA*
GO:0040012	regulation of locomotion	0.0000197601 82977511903	*ERBB4,MACF1,MITF,NRP1,CDH13,ABL2,DACH1,FRMD5,LDLRAD4,STARD13,PHACTR1,PIK3R3,LAMA1,CDH11,DOCK4,NTRK3,WDPCP,BBS2,FLT1,PRKG1,DSCAM,MAGI2,DOCK10,LDB2,PRKD1,NRG3,ATP8A1,PTPRG,JAM2,SEMA6D,FER,MAPRE2,VCL,RAPGEF2,INSR,SRGAP3,SEMA5A,CTNNA2,APC,RELN,TNR,PRKCA*
GO:0030029	actin filament-based process	0.0000201674 0879651108	*NRP1,ABL2,PHACTR3,FRMD5,STARD13,CDC42EP3,HMCN1,CORO2B,FAM171A1,PHACTR1,FMN1,ANK2,FGF12,PCDH15,ARHGAP12,PDE4D,FCHSD2,ABCC9,CACNA1C,PRKG1,THSD7A,PRKD1,MICAL3,FER,RYR2,CACNB2,MTPN,SGCD,AKAP13,PGM5,SEMA5A,CALD1,CTNNA2,KCNE4,FMN2,CCDC88A*
GO:0007010	cytoskeleton organization	0.0000257883 71758174694	*ATRX,MACF1,NRP1,ABL2,PHACTR3,EML1,FRMD5,STARD13,DCLK1,CDC42EP3,HMCN1,CORO2B,TTLL11,FAM171A1,PHACTR1,SLC39A12,DISC1,FMN1,ANK2,PCDH15,ARHGAP12,FCHSD2,MAST4,WDPCP,PRKAA2,CCSER2,BBS2,CDC14B,TLN2,PRKG1,THSD7A,IQCJ-SCHIP1,PRKD1,NLGN1,MICAL3,FER,MAP2,MTPN,MAPRE2,PDE4DIP,MPDZ,ATAT1,AKAP13,PGM5,CLIP1,SEMA5A,ATP8A2,CALD1,CTNNA2,APC,PARD3B,FMN2,CCDC88A*
GO:0048518	positive regulation of biological process	0.0000298424 701611029	*SAMD4A,ERBB4,KCNMA1,ZHX3,APBB2,ATRX,MACF1,MITF,TRIM23,NRP1,TEAD1,NFIA,CDH13,ABL2,UTRN,MAML2,SLC8A1,SLC2A13,ANKRD6,CTIF,TBC1D5,FRMD5,SLC40A1,SLCO3A1,CDC42EP3,GRIK2,IGSF11,NRIP1,ITGA1,CLSTN2,TCF12,CORO2B,ITGA8,ZBTB20,ZNF407,TCF4,PBX1,PIK3R3,SLC39A12,DISC1,FMN1,EYA1,GPR158,ASAP1,ST8SIA1,FYN,ANK2,SLC1A2,CADPS,GRIN2B,GRIA1,NEGR1,FGF12,CACNG2,S100B,TOX,ESR1,KLF12,PDE4D,ERC2,FCHSD2,LAMA1,DOCK4,BRINP1,NRXN1,NTRK3,BCL2L13,MAGI1,RAPGEF4,ADCYAP1R1,EPHB1,CREB5,TNRC6B,ZDHHC17,PRKAA2,DNM3,BBS2,ASXL3,DPF3,FLT1,EDIL3,RORA,PTPRD,CDC14B,NTRK2,LOXL2,SOX5,PRKG1,DSCAM,DGKI,FTO,MAGI2,NELL1,AKAP6,IQCJ-SCHIP1,RNF152,CNTN1,GRM5,LDB2,PRKD1,ATP8A1,NLGN1,JAM2,GRID2,LRP2,SEMA6D,SYNE1,FER,RYR2,MAP2,SUSD4,PRLR,CACNB2,STXBP4,MED15,MTPN,MAPRE2,SOX6,PDE4DIP,VCAN,ATAT1,RBMS3,RAPGEF2,INSR,AKAP13,NSG1,CLIP1,SEMA5A,ATP8A2,SLC24A3,APC,RELN,ROR1*

			,*SLC4A4,TNR,VAV3,NPAS3,PRKCA,KMT2C,FMN2,AGO3,CCDC88A,ARNT2*
GO:0051179	localization	0.00004087072680075381	*ERBB4,KCNMA1,ATRX,MACF1,TRIM23,NRP1,CDH13,ABL2,UTRN,SLC8A1,SLC2A13,SIAH3,TBC1D5,SLC40A1,SLCO3A1,PIK3C3,DCLK1,SLC9C1,GRIK2,IGSF11,NKAIN3,NRIP1,CORO2B,EVI5,ITGA8,VPS41,GRIK4,ASB3,GABRG1,SLC39A12,DISC1,SLC35F1,GPR158,GABRB1,TMEM241,APBA2,ASAP1,FYN,ANK2,SLC1A2,CADPS,LRBA,GRIN2B,GRIA1,FGF12,CACNG2,ESR1,ARHGAP12,KCNN3,ABCB5,MEGF11,PDE4D,ERC2,FCHSD2,CDS2,ITPR2,NRXN1,WDPCP,RAPGEF4,ADCYAP1R1,DPP6,GRID1,ZDHHC17,ABCC9,GSG1L,CACNA1C,PRKAA2,DNM3,BBS2,EXOC4,DLG2,SCFD2,NTRK2,GRIK1,PRKG1,DGKI,FTO,NBEA,MAGI2,AKAP6,CNTN1,GRM5,PRKD1,ATP8A1,NLGN1,MICAL3,GRID2,LRP2,SYNE1,FER,MON2,RYR2,MAP2,PRLR,GRIK3,CACNB2,STXBP4,ESYT2,MAPRE2,SGCD,VCL,RAPGEF2,INSR,IMMP2L,ATXN1,GABRG2,UNC13C,NRXN3,NSG1,CPE,KCND2,ATP8A2,SLC24A3,GRIA4,CNIH3,APC,GPC6,PARD3B,RELN,VPS13C,CEP112,STON1-GTF2A1L,SLC4A4,CRB1,VAV3,KCNE4,KCNS3,FMN2,ERC1,CCDC88A*
GO:0055085	transmembrane transport	0.000041802145507388965	*KCNMA1,UTRN,SLC8A1,SLC2A13,SLC40A1,SLCO3A1,SLC9C1,GRIK2,GRIK4,GABRG1,SLC39A12,SLC35F1,GABRB1,TMEM241,FYN,ANK2,SLC1A2,GRIN2B,GRIA1,FGF12,CACNG2,KCNN3,ABCB5,PDE4D,ITPR2,NRXN1,ADCYAP1R1,DPP6,GRID1,ABCC9,CACNA1C,GRIK1,AKAP6,GRM5,PRKD1,ATP8A1,NLGN1,GRID2,LRP2,RYR2,GRIK3,CACNB2,STXBP4,INSR,GABRG2,KCND2,SLC24A3,GRIA4,CNIH3,RELN,SLC4A4,KCNE4,KCNS3*
GO:0021953	central nervous system neuron differentiation	0.00004494284597105402	*NRP1,MDGA2,DCLK1,DISC1,GABRB1,TOX,CDH11,BRINP1,NRXN1,EPHB1,RORA,NTRK2,GRID2,MAP2,MTPN,RAPGEF2*
GO:0006811	monoatomic ion transport	0.00007560626451255241	*KCNMA1,UTRN,SLC8A1,SLC40A1,SLCO3A1,SLC9C1,GRIK2,NKAIN3,GRIK4,GABRG1,SLC39A12,GABRB1,FYN,ANK2,SLC1A2,GRIN2B,GRIA1,FGF12,CACNG2,KCNN3,PDE4D,ITPR2,ADCYAP1R1,DPP6,GRID1,ABCC9,CACNA1C,GRIK1,AKAP6,CNTN1,GRM5,PRKD1,ATP8A1,GRID2,LRP2,RYR2,GRIK3,CACNB2,GABRG2,KCND2,SLC24A3,GRIA4,RELN,SLC4A4,KCNE4,KCNS3*
GO:0040011	locomotion	0.00012311622950567547	*ERBB4,MACF1,MITF,NRP1,CDH13,ABL2,DACH1,FRMD5,LDLRAD4,STARD13,ITGA1,PHACTR1,PIK3R3,LAMA1,CDH11,DOCK4,NTRK3,WDPCP,EPHB1,BBS2,FLT1,PRKG1,DSCAM,MAGI2,DOCK10,LDB2,PRKD1,NRG3,ATP8A1,PTPRG,JAM2,SEMA6D,FER,*

			MAPRE2,VCL,RAPGEF2,INSR,SRGAP3,SEMA5A,CTNNA2,APC,RELN,TNR,VAV3,PRKCA
GO:0031503	protein-containing complex localization	0.00012837881272203362	*ERBB4,CACNG2,NRXN1,WDPCP,GSG1L,DNM3,DLG2,NBEA,NLGN1,SYNE1,SGCD,NRXN3,NSG1,GPC6,RELN,CEP112*
GO:0051130	positive regulation of cellular component organization	0.0001519867036360592	*ATRX,MACF1,NRP1,ABL2,TBC1D5,CDC42EP3,CLSTN2,DISC1,FMN1,ASAP1,FYN,NEGR1,TOX,ERC2,FCHSD2,NRXN1,NTRK3,EPHB1,DNM3,PTPRD,NTRK2,DSCAM,MAGI2,CNTN1,PRKD1,ATP8A1,NLGN1,GRID2,SYNE1,FER,MAPRE2,PDE4DIP,ATAT1,RAPGEF2,INSR,CLIP1,SEMA5A,ATP8A2,APC,RELN,ROR1,CCDC88A*
GO:0007166	cell surface receptor signaling pathway	0.00015312780095795821	*ERBB4,ADAMTS18,MACF1,CTNND2,MITF,NRP1,NFIA,CDH13,MAML2,ANKRD6,LDLRAD4,GRIK2,IGSF11,NCAM1,ITGA1,ITGA8,GRIK4,PIK3R3,DISC1,EYA1,NDRG2,FYN,GRIN2B,GRIA1,PDE4D,PRKACB,LAMA1,NRXN1,NTRK3,WDPCP,MAGI1,ADCYAP1R1,EPHB1,PRICKLE2,GRID1,ZDHHC17,PRKAA2,BBS2,FLT1,RORA,PTPRD,NTRK2,GRIK1,DSCAM,DGKI,MAGI2,CNTN1,GRM5,PRKD1,NRG3,NLGN1,PTPRG,GRID2,LRP2,SEMA6D,SYNE1,FER,FBXL17,PRLR,PTPRA,GRIK3,STXBP4,RBMS3,RAPGEF2,ZMYND11,INSR,CPE,SEMA5A,GRIA4,DOCK3,APC,CHN1,RELN,ROR1,VAV3,PRKCA,ALPK2,CCDC88A*
GO:0007158	neuron cell-cell adhesion	0.0001881272185176868	*NRXN1,NCAM2,NLGN1,NRXN3,ASTN1,TNR*
GO:0051239	regulation of multicellular organismal process	0.00033849199579465215	*ERBB4,ADAMTS18,MACF1,MITF,NRP1,ABL2,SLC8A1,LDLRAD4,STARD13,GRIK2,IGSF11,CLSTN2,CORO2B,ZBTB20,ASB3,PBX1,SLC39A12,DISC1,ARHGAP42,NDRG2,FYN,ANK2,GRIN2B,FGF12,TOX,ESR1,PDE4D,LAMA1,DOCK4,BRINP1,NRXN1,WDPCP,EPHB1,ABCC9,CACNA1C,BBS2,FLT1,PPP1R12B,RORA,PTPRD,NTRK2,LOXL2,SOX5,PRKG1,DSCAM,FTO,NELL1,AKAP6,GRM5,PRKD1,NLGN1,PTPRG,JAM2,GRID2,LRP2,SEMA6D,SYNE1,FER,RYR2,MAP2,PRLR,CACNB2,MTPN,MAPRE2,SOX6,VCL,VCAN,RAPGEF2,INSR,KCND2,SEMA5A,ATP8A2,APC,RELN,TRPS1,TNR,VAV3,PRKCA,KCNE4,ALPK2*
GO:0009966	regulation of signal transduction	0.00035546511166465936	*ERBB4,MACF1,CTNND2,NRP1,CDH13,ABL2,ANKRD6,LDLRAD4,STARD13,GRIK2,IGSF11,NCAM1,ITGA1,ITGA8,DISC1,RALGPS1,ARHGAP42,EYA1,GPR158,NDRG2,FYN,GRIN2B,CACNG2,S100B,ESR1,ARHGAP12,PDE4D,PRKACB,LAMA1,NRXN1,NTRK3,CNKSR2,ADCYAP1R1,EPHB1,ZDHHC17,PRKAA2,FLT1,RORA,PTPRD,MYO9A,NTRK2,DGKI,MAGI2,AKAP6,IQCJ-SCHIP1,RNF152,GRM5,PRKD1,NLGN1,LRP2,SYNE1,FER,FBXL17,PRLR,MAPRE2,R*

			IC8B,SEZ6L,ATAT1,RBMS3,RAPGEF2,ZMYND11,INSR,RGS6,SRGAP3,AKAP13,RALGAPA1,SEMA5A,ARHGAP24,CNIH3,DOCK3,APC,GPC6,CHN1,RELN,ROR1,VAV3,PRKCA,INPP5A,AGO3,ALPK2,CCDC88A
GO:0050793	regulation of developmental process	0.00037080875698658467	*ERBB4,ZHX3,MACF1,MITF,NRP1,SLC8A1,LDLRAD4,LRRC4C,CDC42EP3,CLSTN2,TCF12,TCF4,PBX1,FAM171A1,SLC39A12,DISC1,EYA1,ASAP1,FYN,CSMD3,S100B,TOX,ESR1,LAMA1,BRINP1,NRXN1,WDPCP,EPHB1,DNM3,BBS2,DPF3,FLT1,RORA,PTPRD,MYO9A,NTRK2,LOXL2,SOX5,DSCAM,FTO,NELL1,AKAP6,GRM5,PLEKHB2,PRKD1,NLGN1,JAM2,GRID2,LRP2,SEMA6D,SYNE1,MAP2,PRLR,FAT3,MTPN,SOX6,VCL,VCAN,ATAT1,RAPGEF2,INSR,SEMA5A,ATP8A2,APC,CHN1,RELN,TRPS1,TNR,PRKCA,ALPK2*
GO:1901888	regulation of cell junction assembly	0.0003820641910571218	*MACF1,NRP1,CLSTN2,FMN1,PEAK1,NEGR1,NRXN1,WDPCP,EPHB1,PTPRD,NTRK2,NLGN1,GRID2,PTPRA,VCL,RAPGEF2*
GO:0050896	response to stimulus	0.0004045873736272355	*ERBB4,KCNMA1,APBB2,ATRX,ADAMTS18,MACF1,CTNND2,MITF,TRIM23,NRP1,TEAD1,NFIA,CDH13,ABL2,MAML2,SLC8A1,DACH1,ANKRD6,RERG,TBC1D5,LDLRAD4,STARD13,PIK3C3,DCLK1,CDC42EP3,HMCN1,GRIK2,IGSF11,RGL1,NRIP1,NCAM1,ITGA1,TCF12,GNG12,TANC1,CORO2B,ITGA8,VPS41,GRIK4,ZBTB20,ASB3,PIK3R3,SLC39A12,DISC1,RALGPS1,ARHGAP42,EYA1,GPR158,GABRB1,NDRG2,ST8SIA1,FYN,ANK2,SLC1A2,GRIN2B,STK32A,GRIA1,FGF12,DGKB,CACNG2,S100B,PCDH15,ESR1,ARHGAP12,PDE4D,PRKACB,GNG2,LAMA1,DOCK4,MAST4,ITPR2,BRINP1,NRXN1,NTRK3,WDPCP,MAGI1,RAPGEF4,CNKSR2,ADCYAP1R1,EPHB1,PRICKLE2,GRID1,ZDHHC17,ABCC9,DCDC1,CACNA1C,PRKAA2,BBS2,DPF3,FLT1,DLG2,PPP1R12B,RORA,PTPRD,CDC14B,MYO9A,FOXN3,NTRK2,LOXL2,GRIK1,SOX5,PRKG1,DSCAM,DGKI,FTO,MAGI2,AKAP6,IQCJ-SCHIP1,RNF152,CNTN1,DOCK10,GRM5,PRKD1,NRG3,NLGN1,PTPRG,OR9Q1,GRID2,LRP2,SEMA6D,SYNE1,RALGPS2,FER,SNRK,RYR2,FBXL17,SUSD4,PRLR,PTPRA,GRIK3,STXBP4,MTPN,MAPRE2,SOX6,SGCD,MSRA,RIC8B,VCL,SEZ6L,ATAT1,RBMS3,RAPGEF2,ZMYND11,RAPGEF5,INSR,RGS6,SRGAP3,AKAP13,IMMP2L,CHCHD6,GABRG2,NRXN3,RALGAPA1,NSG1,CPE,KCND2,SEMA5A,ATP8A2,ARHGAP24,GRIA4,CNIH3,DOCK3,CTNNA2,APC,GPC6,CHN1,RELN,VPS13C,ROR1,TNR,DTNA,CRB1,VAV3,PRKCA,KMT2C,INPP5A,FMN2,ERC1,AGO3,ALPK2,CCDC88A,ARNT2,AFF3*
GO:0048167	regulation of synaptic	0.0004937956424297483	*GRIK2,IGSF11,GRIN2B,GRIA1,S100B,ERC2,NTRK2,DGKI,GRM5,GRID2,RAPGEF2*

	plasticity		*,UNC13C,NSG1,RELN,TNR,ERC1*
GO:0099505	regulation of presynaptic membrane potential	0.0006043648236529639	*GRIK2,GRIK4,GABRB1,GRIN2B,GRIA1,GRIK3,GRIA4*
GO:1902531	regulation of intracellular signal transduction	0.0006825947601046064	*ERBB4,NRP1,CDH13,ABL2,ANKRD6,LDLRAD4,STARD13,GRIK2,ITGA1,RALGPS1,ARHGAP42,NDRG2,FYN,S100B,ESR1,ARHGAP12,PDE4D,PRKACB,NRXN1,NTRK3,ADCYAP1R1,EPHB1,ZDHHC17,PRKAA2,FLT1,RORA,MYO9A,NTRK2,DGKI,MAGI2,AKAP6,IQCJ-SCHIP1,RNF152,GRM5,PRKD1,NLGN1,FER,MAPRE2,SEZ6L,RAPGEF2,ZMYND11,INSR,SRGAP3,AKAP13,RALGAPA1,SEMA5A,ARHGAP24,DOCK3,CHN1,RELN,ROR1,VAV3,PRKCA,AGO3,CCDC88A*
GO:0006810	transport	0.0006880726390757017	*ERBB4,KCNMA1,MACF1,TRIM23,NRP1,CDH13,ABL2,UTRN,SLC8A1,SLC2A13,TBC1D5,SLC40A1,SLCO3A1,PIK3C3,DCLK1,SLC9C1,GRIK2,NKAIN3,EVI5,VPS41,GRIK4,GABRG1,SLC39A12,SLC35F1,GPR158,GABRB1,TMEM241,APBA2,FYN,ANK2,SLC1A2,CADPS,GRIN2B,GRIA1,FGF12,CACNG2,ARHGAP12,KCNN3,ABCB5,MEGF11,PDE4D,ERC2,FCHSD2,ITPR2,NRXN1,WDPCP,RAPGEF4,ADCYAP1R1,DPP6,GRID1,ZDHHC17,ABCC9,GSG1L,CACNA1C,DNM3,BBS2,EXOC4,DLG2,SCFD2,NTRK2,GRIK1,PRKG1,DGKI,MAGI2,AKAP6,CNTN1,GRM5,PRKD1,ATP8A1,NLGN1,MICAL3,GRID2,LRP2,SYNE1,FER,MON2,RYR2,MAP2,PRLR,GRIK3,CACNB2,STXBP4,ESYT2,INSR,ATXN1,GABRG2,UNC13C,NRXN3,NSG1,CPE,KCND2,ATP8A2,SLC24A3,GRIA4,CNIH3,RELN,VPS13C,STON1-GTF2A1L,SLC4A4,VAV3,KCNE4,KCNS3,FMN2,ERC1,CCDC88A*
GO:0007611	learning or memory	0.0007358661855945334	*CLSTN2,TANC1,ITGA8,FYN,GRIN2B,GRIA1,S100B,BRINP1,NRXN1,NTRK2,DGKI,GRM5,ATP8A1,INSR,ATXN1,NRXN3,RELN,TNR*
GO:0048646	anatomical structure formation involved in morphogenesis	0.0007369885898676773	*NRP1,CDH13,STARD13,SLC40A1,TANC1,ITGA8,PIK3R3,SLC39A12,FMN1,EYA1,ANK2,MEGF11,PRKACB,CDH11,NRXN1,EPHB1,FLT1,EXOC4,RORA,LOXL2,DSCAM,THSD7A,CNTN1,ENPEP,PRKD1,NRG3,JAM2,GRID2,LRP2,FAT3,MTPN,AKAP13,NRXN3,PGM5,SEMA5A,ATP8A2,CALD1,ARHGAP24,RELN,CRB1,VAV3,PRKCA*
GO:0001508	action potential	0.0007566977376085893	*SLC8A1,GRIK2,ANK2,GRIA1,FGF12,ABCC9,CACNA1C,NTRK2,AKAP6,RYR2,CACNB2,KCND2,KCNE4*
GO:0097061	dendritic spine organization	0.0008540730839360207	*CTNND2,TANC1,FYN,GRIN2B,EPHB1,DNM3,DOCK10,NLGN1,INSR,RELN*
GO:0035418	protein	0.0009612908	*ERBB4,CACNG2,NRXN1,DLG2,NBEA,NLGN*

	localization to synapse	227087199	*1,NRXN3,NSG1,GPC6,RELN*
GO:0034762	regulation of transmembrane transport	0.0009714698492955683	*UTRN,SLC8A1,FYN,ANK2,SLC1A2,GRIN2B,FGF12,CACNG2,PDE4D,NRXN1,ADCYAP1R1,DPP6,CACNA1C,AKAP6,GRM5,PRKD1,NLGN1,RYR2,CACNB2,STXBP4,INSR,CNIH3,RELN*
GO:0048589	developmental growth	0.0010860396345236383	*ERBB4,ATRX,MACF1,NRP1,DCLK1,SLC39A12,DISC1,FMN1,APBA2,SLC1A2,S100B,PCDH15,ESR1,BBS2,SCAPER,PRKG1,DSCAM,FTO,MAGI2,AKAP6,SEMA6D,MAP2,PRLR,MTPN,VCL,INSR,SEMA5A,ATP8A2,TNR*
GO:0007416	synapse assembly	0.0011407536381933764	*ERBB4,CLSTN2,GRIA1,NEGR1,NRXN1,EPHB1,DNM3,PTPRD,NTRK2,DSCAM,NRG3,NLGN1,GRID2,GABRG2,NRXN3*
GO:0050890	cognition	0.001358115702537152	*CLSTN2,TANC1,ITGA8,GPR158,FYN,GRIN2B,GRIA1,S100B,BRINP1,NRXN1,NTRK2,DGKI,GRM5,ATP8A1,INSR,ATXN1,NRXN3,RELN,TNR*
GO:0021954	central nervous system neuron development	0.0018880768495466223	*NRP1,DCLK1,DISC1,GABRB1,CDH11,BRINP1,EPHB1,NTRK2,MAP2,RAPGEF2*
GO:0007612	learning	0.0018998547462411323	*CLSTN2,TANC1,FYN,NRXN1,NTRK2,DGKI,GRM5,ATP8A1,INSR,ATXN1,NRXN3,RELN,TNR*
GO:0006941	striated muscle contraction	0.0019037465599399633	*SLC8A1,ASB3,ANK2,FGF12,PDE4D,ABCC9,CACNA1C,PRKD1,RYR2,CACNB2,SGCD,ATP8A2,DTNA,KCNE4*
GO:0001764	neuron migration	0.0019037465599399633	*APBB2,NRP1,DCLK1,PHACTR1,DISC1,FYN,NTRK2,PRKG1,NRG3,FAT3,RAPGEF2,ASTN1,CTNNA2,RELN*
GO:0044087	regulation of cellular component biogenesis	0.0020263694262775403	*MACF1,NRP1,CDC42EP3,CLSTN2,CORO2B,VPS41,SLC39A12,FMN1,PEAK1,NEGR1,FCHSD2,NRXN1,WDPCP,EPHB1,PRKAA2,DNM3,SACS,PTPRD,NTRK2,LDB2,PRKD1,NLGN1,GRID2,FER,MAP2,PTPRA,MTPN,PDE4DIP,VCL,ATAT1,RAPGEF2,CLIP1,ARHGAP24,APC,PRKCA,CCDC88A*
GO:1902414	protein localization to cell junction	0.002410537829448266	*ERBB4,CACNG2,NRXN1,DLG2,NBEA,NLGN1,VCL,NRXN3,NSG1,GPC6,RELN*
GO:0051056	regulation of small GTPase mediated signal transduction	0.0028124402301346603	*NRP1,ABL2,STARD13,RALGPS1,ARHGAP42,ARHGAP12,ADCYAP1R1,MYO9A,DGKI,MAPRE2,SRGAP3,AKAP13,RALGAPA1,ARHGAP24,DOCK3,CHN1,RELN,VAV3*
GO:0106027	neuron projection organization	0.0028736246809353536	*CTNND2,TANC1,FYN,GRIN2B,EPHB1,DNM3,DOCK10,NLGN1,INSR,RELN*
GO:0086003	cardiac muscle cell contraction	0.003280936643087311	*ANK2,FGF12,PDE4D,ABCC9,CACNA1C,RYR2,CACNB2,SGCD,KCNE4*

GO:0060560	developmental growth involved in morphogenesis	0.0033289893908000612	*MACF1,NRP1,DCLK1,SLC39A12,DISC1,FMN1,S100B,ESR1,PRKG1,DSCAM,MAGI2,SEMA6D,MAP2,VCL,SEMA5A,TNR*
GO:0099072	regulation of postsynaptic membrane neurotransmitter receptor levels	0.003516311828269161	*ERBB4,CACNG2,GSG1L,DNM3,NBEA,SYNE1,STXBP4,NRXN3,NSG1,GPC6*
GO:0048583	regulation of response to stimulus	0.003527200144333425	*ERBB4,ADAMTS18,MACF1,CTNND2,NRP1,CDH13,ABL2,ANKRD6,LDLRAD4,STARD13,GRIK2,IGSF11,NCAM1,ITGA1,CORO2B,ITGA8,DISC1,RALGPS1,ARHGAP42,EYA1,GPR158,NDRG2,FYN,GRIN2B,CACNG2,S100B,ESR1,ARHGAP12,PDE4D,PRKACB,LAMA1,NRXN1,NTRK3,CNKSR2,ADCYAP1R1,EPHB1,ZDHHC17,PRKAA2,BBS2,DPF3,FLT1,RORA,PTPRD,MYO9A,NTRK2,PRKG1,DSCAM,DGKI,MAGI2,AKAP6,IQCJ-SCHIP1,RNF152,GRM5,PRKD1,NLGN1,GRID2,LRP2,SEMA6D,SYNE1,FER,FBXL17,SUSD4,PRLR,MTPN,MAPRE2,RIC8B,SEZ6L,ATAT1,RBMS3,RAPGEF2,ZMYND11,INSR,RGS6,SRGAP3,AKAP13,RALGAPA1,SEMA5A,ARHGAP24,CNIH3,DOCK3,CTNNA2,APC,GPC6,CHN1,RELN,VPS13C,ROR1,TNR,VAV3,PRKCA,INPP5A,FMN2,AGO3,ALPK2,CCDC88A*
GO:0031345	negative regulation of cell projection organization	0.0036073457029294656	*NRP1,FYN,GRIN2B,NRXN1,DNM3,NLGN1,PTPRG,SEMA6D,MAP2,FAT3,RAPGEF2,SEMA5A,ARHGAP24,TNR*
GO:0098742	cell-cell adhesion via plasma-membrane adhesion molecules	0.003615021922246196	*CDH13,PCDH7,LRRC4C,CADM2,HMCN1,IGSF11,CLSTN2,PCDH15,CDH11,NRXN1,PTPRD,DSCAM,NLGN1,GRID2,FAT3,PCDH9,CRB1*
GO:0030900	forebrain development	0.004526707579466748	*ERBB4,ATRX,NRP1,DCLK1,PHACTR1,DISC1,FYN,SLC1A2,GRIA1,TOX,BBS2,NTRK2,PRKG1,NRG3,LRP2,PCDH9,ATAT1,RAPGEF2,SEMA5A,RELN,TNR*
GO:0044093	positive regulation of molecular function	0.00506694400342444	*NRP1,ABL2,SLCO3A1,ITGA1,EVI5,ARHGAP42,ASAP1,FYN,ANK2,GRIN2B,ESR1,NRXN1,NTRK3,BCL2L13,CACNA1C,FLT1,CDC14B,MYO9A,MAGI2,AKAP6,DOCK10,GRM5,PRKD1,FER,RYR2,PRLR,CACNB2,MTPN,MAPRE2,RAPGEF2,INSR,RGS6,RALGAPA1,CHN1,RELN,ROR1,ERC1,CCDC88A*
GO:0051234	establishment of localization	0.005600358769908067	*ERBB4,KCNMA1,MACF1,TRIM23,NRP1,CDH13,ABL2,UTRN,SLC8A1,SLC2A13,SIAH3,TBC1D5,SLC40A1,SLCO3A1,PIK3C3,DCLK1,SLC9C1,GRIK2,NKAIN3,CORO2B,E*

			VI5,ITGA8,VPS41,GRIK4,GABRG1,SLC39A12,SLC35F1,GPR158,GABRB1,TMEM241,APBA2,FYN,ANK2,SLC1A2,CADPS,GRIN2B,GRIA1,FGF12,CACNG2,ARHGAP12,KCNN3,ABCB5,MEGF11,PDE4D,ERC2,FCHSD2,ITPR2,NRXN1,WDPCP,RAPGEF4,ADCYAP1R1,DPP6,GRID1,ZDHHC17,ABCC9,GSG1L,CACNA1C,DNM3,BBS2,EXOC4,DLG2,SCFD2,NTRK2,GRIK1,PRKG1,DGKI,MAGI2,AKAP6,CNTN1,GRM5,PRKD1,ATP8A1,NLGN1,MICAL3,GRID2,LRP2,SYNE1,FER,MON2,RYR2,MAP2,PRLR,GRIK3,CACNB2,STXBP4,ESYT2,INSR,IMMP2L,ATXN1,GABRG2,UNC13C,NRXN3,NSG1,CPE,KCND2,ATP8A2,SLC24A3,GRIA4,CNIH3,APC,PARD3B,RELN,VPS13C,STON1-GTF2A1L,SLC4A4,VAV3,KCNE4,KCNS3,FMN2,ERC1,CCDC88A
GO:0051960	regulation of nervous system development	0.006653007303845113	*MACF1,NRP1,CLSTN2,DISC1,BRINP1,NRXN1,EPHB1,PTPRD,NTRK2,DSCAM,GRM5,NLGN1,JAM2,GRID2,LRP2,SEMA6D,MAP2,VCAN,RAPGEF2,SEMA5A,RELN,TNR*
GO:0050806	positive regulation of synaptic transmission	0.007362975679677428	*GRIK2,IGSF11,CLSTN2,GPR158,GRIN2B,GRIA1,CACNG2,NRXN1,NTRK2,NLGN1,NSG1,RELN,TNR*
GO:0007169	transmembrane receptor protein tyrosine kinase signaling pathway	0.0074221595558066475	*ERBB4,NRP1,CDH13,ITGA1,PIK3R3,FYN,NRXN1,NTRK3,EPHB1,ZDHHC17,FLT1,NTRK2,PRKD1,NRG3,PTPRG,FER,PRLR,PTPRA,STXBP4,RAPGEF2,INSR,DOCK3,APC,CHN1,ROR1,CCDC88A*
GO:0007274	neuromuscular synaptic transmission	0.007579551808772913	*ERC2,FCHSD2,NRXN1,NLGN1,DTNA,ERC1*
GO:0009888	tissue development	0.00794862696151652	*ERBB4,ATRX,NRP1,NFIA,SLC8A1,LDLRAD4,STARD13,SLC40A1,NCAM1,ITGA8,PBX1,SLC39A12,FMN1,EYA1,TOX,PCDH15,ESR1,ARHGAP12,PDE4D,PRKACB,LAMA1,WDPCP,EPHB1,GREB1L,BBS2,EXOC4,MYO9A,LOXL2,SOX5,PRKG1,FTO,MAGI2,NELL1,AKAP6,LDB2,LRP2,SEMA6D,SYNE1,FER,RYR2,FBXL17,PRLR,MTPN,SOX6,SGCD,VCL,RAPGEF2,INSR,AKAP13,PGM5,SEMA5A,SLC24A3,ARHGAP24,APC,TRPS1,KAZN,ALPK2*
GO:0009887	animal organ morphogenesis	0.008713234479217957	*ERBB4,NRP1,MMP16,SLC40A1,ITGA8,PBX1,FMN1,EYA1,PCDH15,ESR1,MEGF11,LRIG1,LAMA1,WDPCP,EPHB1,GREB1L,ABCC9,BBS2,ASXL3,EXOC4,FOXN3,NTRK2,SOX5,DSCAM,NRG3,LRP2,RYR2,FAT3,SOX6,INSR,CPE,ATP8A2,CTNNA2,APC,CRB1,ALPK2*
GO:0051897	positive regulation of phosphatidyl	0.008872956385558692	*ERBB4,NRXN1,NTRK3,FLT1,NTRK2,PRKD1,FER,INSR,SEMA5A,RELN,ROR1,VAV3,CCDC88A*

	inositol 3-kinase/protein kinase B signal transduction		
GO:0150115	cell-substrate junction organization	0.008971895932374161	*MACF1,NRP1,CORO2B,FMN1,PEAK1,CDH11,WDPCP,PTPRA,MAPRE2,VCL*
GO:0010880	regulation of release of sequestered calcium ion into cytosol by sarcoplasmic reticulum	0.00943435376868892	*SLC8A1,ANK2,PDE4D,CACNA1C,AKAP6,RYR2*
GO:0043087	regulation of GTPase activity	0.01031417412266019	*EVI5,ARHGAP42,ASAP1,NTRK3,MYO9A,NTRK2,PRKG1,DOCK10,MAPRE2,RAPGEF2,RGS6,RALGAPA1,CHN1,VAV3*
GO:0003018	vascular process in circulatory system	0.010466746927811797	*KCNMA1,SLC8A1,SLC2A13,SLCO3A1,ITGA1,ARHGAP42,SLC1A2,DOCK4,ABCC9,BBS2,PRKG1,ATP8A1,LRP2,INSR,SLC24A3,SLC4A4*
GO:0051493	regulation of cytoskeleton organization	0.011073624751509614	*NRP1,ABL2,CDC42EP3,CORO2B,SLC39A12,FMN1,FCHSD2,PRKAA2,IQCJ-SCHIP1,PRKD1,FER,MAP2,MTPN,MAPRE2,PDE4DIP,MPDZ,ATAT1,AKAP13,CLIP1,SEMA5A,CTNNA2,APC,CCDC88A*
GO:0016043	cellular component organization	0.011499764449925105	*ERBB4,KCNMA1,APBB2,ATRX,ADAMTS18,MACF1,CTNND2,MITF,RTN1,NRP1,TEAD1,NFIA,FRY,CDH13,ABL2,PHACTR3,EML1,RERG,TBC1D5,FRMD5,STARD13,MMP16,LRRC4C,PDZRN3,PIK3C3,DCLK1,CDC42EP3,HMCN1,NCAM1,ITGA1,CLSTN2,KDM4B,TANC1,CORO2B,TTLL11,ITGA8,VPS41,TCF4,FAM171A1,PHACTR1,SLC39A12,DISC1,FMN1,PEAK1,EYA1,ADAMTS19,GPR158,ASAP1,FYN,ANK2,SLC1A2,CSMD3,LRBA,GRIN2B,GRIA1,NEGR1,DGKB,CACNG2,S100B,TOX,PCDH15,ESR1,ARHGAP12,ERC2,PRKACB,FCHSD2,LAMA1,CDH11,SETBP1,CDS2,MAST4,NRXN1,NTRK3,WDPCP,MAGI1,CNKSR2,EPHB1,CTTNBP2,GRID1,ZDHHC17,GSG1L,PRKAA2,CCSER2,DNM3,BBS2,DPF3,EXOC4,SACS,PTPRD,CDC14B,TLN2,MYO9A,NTRK2,LOXL2,PRKG1,DSCAM,THSD7A,NBEA,NCAM2,MAGI2,AKAP6,IQCJ-SCHIP1,CNTN1,DOCK10,GRM5,PRKD1,NRG3,ATP8A1,NLGN1,PTPRG,MICAL3,GRID2,LRP2,SEMA6D,SYNE1,FER,MAP2,PRLR,PTPRA,FAT3,CACNB2,MED15,MTPN,ESYT2,MAPRE2,PDE4DIP,MPDZ,VCL,SEZ6L,ATAT1,RAPGEF2,ZMYND11,INSR,AKAP13,IMMP2L,CHCHD6,GABRG2,UNC13C,NRXN3,PGM5,NSG1,CLIP1,KCND2,SEMA5A,AT*

			P8A2,CALD1,ARHGAP24,CTNNA2,APC,PARD3B,CHN1,RELN,VPS13C,ROR1,TNR,CRB1,VAV3,PRKCA,KMT2C,KCNS3,FMN2,ERC1,AGO3,CCDC88A
GO:0048041	focal adhesion assembly	0.01333537231689404	*MACF1,NRP1,CORO2B,FMN1,PEAK1,CDH11,WDPCP,PTPRA,VCL*
GO:0065009	regulation of molecular function	0.013426825268222727	*NRP1,ABL2,UTRN,SLC8A1,SLCO3A1,ITGA1,EVI5,PBX1,DISC1,ARHGAP42,ASAP1,FYN,ANK2,GRIN2B,FGF12,CACNG2,ESR1,NRXN1,NTRK3,BCL2L13,CACNA1C,FLT1,CDC14B,MYO9A,NTRK2,PRKG1,MAGI2,AKAP6,DOCK10,GRM5,LDB2,PRKD1,NLGN1,FER,RYR2,MAP2,PRLR,CACNB2,MTPN,MAPRE2,RAPGEF2,INSR,RGS6,RALGAPA1,CNIH3,APC,CHN1,RELN,ROR1,VAV3,ERC1,CCDC88A*
GO:0051345	positive regulation of hydrolase activity	0.014317926371556896	*ABL2,ITGA1,EVI5,ARHGAP42,ASAP1,FYN,GRIN2B,ESR1,NTRK3,BCL2L13,FLT1,MYO9A,MAGI2,DOCK10,MAPRE2,RAPGEF2,RGS6,RALGAPA1,CHN1*
GO:0150104	transport across blood-brain barrier	0.016173925275929583	*SLC2A13,SLCO3A1,SLC1A2,ABCC9,ATP8A1,LRP2,INSR,SLC24A3,SLC4A4*
GO:0010232	vascular transport	0.016173925275929583	*SLC2A13,SLCO3A1,SLC1A2,ABCC9,ATP8A1,LRP2,INSR,SLC24A3,SLC4A4*
GO:0007411	axon guidance	0.01628389387651497	*APBB2,NRP1,NCAM1,FYN,NRXN1,EPHB1,PTPRD,DSCAM,CNTN1,SEMA6D,NRXN3,SEMA5A,CHN1,RELN,TNR*
GO:0097485	neuron projection guidance	0.01628389387651497	*APBB2,NRP1,NCAM1,FYN,NRXN1,EPHB1,PTPRD,DSCAM,CNTN1,SEMA6D,NRXN3,SEMA5A,CHN1,RELN,TNR*
GO:0060048	cardiac muscle contraction	0.016463822312346867	*SLC8A1,ASB3,ANK2,FGF12,PDE4D,ABCC9,CACNA1C,RYR2,CACNB2,SGCD,KCNE4*
GO:0140058	neuron projection arborization	0.01731507163321655	*MACF1,NRP1,PHACTR1,MYO9A,NLGN1,LRP2*
GO:0050877	nervous system process	0.01878243709886259	*HMCN1,GRIK2,IGSF11,CLSTN2,TANC1,ITGA8,DISC1,EYA1,GPR158,FYN,GRIN2B,GRIA1,FGF12,CACNG2,S100B,PCDH15,LRIG1,BRINP1,NRXN1,EPHB1,BBS2,MYO9A,NTRK2,DGKI,GRM5,PRKD1,ATP8A1,NLGN1,OR9Q1,JAM2,GRID2,LRP2,SYNE1,CACNB2,INSR,ATXN1,NRXN3,KCND2,ATP8A2,LHFPL3,CTNNA2,RELN,ROR1,TNR,CRB1*
GO:0098815	modulation of excitatory postsynaptic potential	0.019874124642329512	*GRIK2,IGSF11,GRIN2B,NRXN1,NLGN1,SYNE1,RELN*
GO:0150116	regulation of cell-substrate junction	0.02002616868705161	*MACF1,NRP1,FMN1,PEAK1,WDPCP,PTPRA,MAPRE2,VCL*

	organization		
GO:2000311	regulation of AMPA receptor activity	0.022758674803196273	*CACNG2,NRXN1,NLGN1,CNIH3,RELN*
GO:0060074	synapse maturation	0.025044071369971114	*NFIA,DISC1,NRXN1,NLGN1,SEZ6L,RELN*
GO:0014808	release of sequestered calcium ion into cytosol by sarcoplasmic reticulum	0.025044071369971114	*SLC8A1,ANK2,PDE4D,CACNA1C,AKAP6,RYR2*
GO:0090257	regulation of muscle system process	0.02514043389763358	*SLC8A1,ASB3,ARHGAP42,ANK2,PDE4D,DOCK4,CACNA1C,PPP1R12B,PRKG1,AKAP6,PRKD1,RYR2,MTPN,PRKCA*
GO:0031644	regulation of nervous system process	0.02603842373837887	*GRIK2,IGSF11,GRIN2B,FGF12,NRXN1,NLGN1,JAM2,SYNE1,RELN,TNR*
GO:0043547	positive regulation of GTPase activity	0.026672051893072746	*EVI5,ARHGAP42,ASAP1,NTRK3,MYO9A,DOCK10,MAPRE2,RAPGEF2,RGS6,RALGAPA1,CHN1*
GO:0051962	positive regulation of nervous system development	0.02675778273801192	*MACF1,NRP1,CLSTN2,DISC1,NRXN1,EPHB1,PTPRD,NTRK2,DSCAM,GRM5,NLGN1,GRID2,LRP2,VCAN,SEMA5A,RELN*
GO:0035295	tube development	0.027726570032468895	*ATRX,NRP1,NFIA,CDH13,STARD13,PBX1,PIK3R3,SLC39A12,FMN1,EYA1,ESR1,PRKACB,LAMA1,NRXN1,WDPCP,EPHB1,GREB1L,FLT1,SCAPER,RORA,NTRK2,LOXL2,THSD7A,ENPEP,PRKD1,LRP2,FER,RYR2,SGCD,RAPGEF2,NRXN3,SEMA5A,CALD1,ARHGAP24,VAV3,PRKCA*
GO:0035239	tube morphogenesis	0.027798720095875765	*NRP1,CDH13,STARD13,PBX1,PIK3R3,SLC39A12,FMN1,EYA1,ESR1,PRKACB,LAMA1,NRXN1,EPHB1,GREB1L,FLT1,RORA,NTRK2,LOXL2,THSD7A,ENPEP,PRKD1,LRP2,RYR2,SGCD,RAPGEF2,NRXN3,SEMA5A,CALD1,ARHGAP24,VAV3,PRKCA*
GO:0043491	phosphatidylinositol 3-kinase/protein kinase B signal transduction	0.027917173064315185	*ERBB4,PIK3R3,NRXN1,NTRK3,FLT1,NTRK2,MAGI2,PRKD1,LRP2,FER,INSR,SEMA5A,RELN,ROR1,VAV3,CCDC88A*
GO:0048514	blood vessel morphogenesis	0.02883107461447525	*NRP1,CDH13,STARD13,PIK3R3,SLC39A12,EYA1,LAMA1,NRXN1,EPHB1,FLT1,RORA,NTRK2,LOXL2,THSD7A,ENPEP,PRKD1,LRP2,SGCD,RAPGEF2,NRXN3,SEMA5A,CALD1,ARHGAP24,VAV3,PRKCA*
GO:1903514	release of sequestered	0.02983883711684524	*SLC8A1,ANK2,PDE4D,CACNA1C,AKAP6,RYR2*

	calcium ion into cytosol by endoplasmic reticulum		
GO:0048588	developmental cell growth	0.033729666018701164	*MACF1,NRP1,DCLK1,SLC39A12,DISC1,S100B,PRKG1,DSCAM,AKAP6,SEMA6D,MAP2,VCL,SEMA5A,TNR*
GO:0001654	eye development	0.0340567615 5144981	*ADAMTS18,MITF,NRP1,NFIA,DCLK1,PBX1,ABCB5,MEGF11,LAMA1,WDPCP,EPHB1,CACNA1C,FLT1,SCAPER,NTRK2,DSCAM,FAT3,ATP8A2,CRB1*
GO:0043954	cellular component maintenance	0.03408246390317018	*TANC1,FYN,GRIN2B,ERC2,NRXN1,NLGN1,INSR,ERC1*
GO:0051968	positive regulation of synaptic transmission, glutamatergic	0.03534853967540784	*GRIN2B,CACNG2,NRXN1,NLGN1,RELN,TNR*
GO:0007044	cell-substrate junction assembly	0.03624861121395607	*MACF1,NRP1,CORO2B,FMN1,PEAK1,CDH11,WDPCP,PTPRA,VCL*
GO:0007160	cell-matrix adhesion	0.03711933903626974	*MACF1,NRP1,CDH13,UTRN,ITGA1,CORO2B,ITGA8,DISC1,FMN1,PEAK1,CDH11,WDPCP,PTPRA,VCL*
GO:0071840	cellular component organization or biogenesis	0.037305516218590844	*ERBB4,KCNMA1,APBB2,ATRX,ADAMTS18,MACF1,CTNND2,MITF,RTN1,NRP1,TEAD1,NFIA,FRY,CDH13,ABL2,PHACTR3,EML1,RERG,TBC1D5,FRMD5,STARD13,MMP16,LRRC4C,PDZRN3,PIK3C3,DCLK1,CDC42EP3,HMCN1,NCAM1,ITGA1,CLSTN2,KDM4B,TANC1,CORO2B,TTLL11,ITGA8,VPS41,TCF4,FAM171A1,PHACTR1,SLC39A12,DISC1,FMN1,PEAK1,EYA1,ADAMTS19,GPR158,ASAP1,FYN,ANK2,SLC1A2,CSMD3,LRBA,GRIN2B,GRIA1,NEGR1,DGKB,CACNG2,S100B,TOX,PCDH15,ESR1,ARHGAP12,ERC2,PRKACB,FCHSD2,LAMA1,CDH11,SETBP1,CDS2,MAST4,NRXN1,NTRK3,WDPCP,MAGI1,CNKSR2,EPHB1,CTTNBP2,GRID1,ZDHHC17,GSG1L,PRKAA2,CCSER2,DNM3,BBS2,DPF3,EXOC4,SACS,PTPRD,CDC14B,TLN2,MYO9A,NTRK2,LOXL2,PRKG1,DSCAM,THSD7A,NBEA,NCAM2,MAGI2,AKAP6,IQCJ-SCHIP1,CNTN1,DOCK10,GRM5,LDB2,PRKD1,NRG3,ATP8A1,NLGN1,PTPRG,MICAL3,GRID2,LRP2,SEMA6D,SYNE1,FER,MAP2,PRLR,PTPRA,FAT3,CACNB2,MED15,MTPN,ESYT2,MAPRE2,PDE4DIP,MPDZ,VCL,SEZ6L,ATAT1,RAPGEF2,ZMYND11,INSR,AKAP13,IMMP2L,CHCHD6,GABRG2,UNC13C,NRXN3,PGM5,NSG1,CLIP1,KCND2,SEMA5A,ATP8A2,CALD1,ARHGAP24,CTNNA2,A*

			PC,PARD3B,CHN1,RELN,VPS13C,ROR1,TNR,CRB1,VAV3,PRKCA,KMT2C,KCNS3,FMN2,ERC1,AGO3,CCDC88A
GO:0001952	regulation of cell-matrix adhesion	0.03775879487905421	*MACF1,NRP1,CDH13,UTRN,DISC1,FMN1,PEAK1,WDPCP,PTPRA,VCL*
GO:0030036	actin cytoskeleton organization	0.038013423150302275	*NRP1,ABL2,PHACTR3,FRMD5,STARD13,CDC42EP3,HMCN1,CORO2B,FAM171A1,PHACTR1,FMN1,PCDH15,ARHGAP12,FCHSD2,PRKG1,THSD7A,PRKD1,MICAL3,FER,MTPN,AKAP13,PGM5,SEMA5A,CALD1,CTNNA2,FMN2,CCDC88A*
GO:0032879	regulation of localization	0.03824841447646923	*ERBB4,NRP1,CDH13,ABL2,UTRN,SLC8A1,SIAH3,TBC1D5,DCLK1,NKAIN3,CORO2B,GPR158,FYN,ANK2,SLC1A2,CADPS,GRIN2B,FGF12,CACNG2,PDE4D,ERC2,ITPR2,NRXN1,WDPCP,RAPGEF4,ADCYAP1R1,DPP6,GSG1L,CACNA1C,PRKAA2,DNM3,PRKG1,FTO,NBEA,MAGI2,AKAP6,CNTN1,GRM5,PRKD1,ATP8A1,NLGN1,SYNE1,FER,RYR2,MAP2,CACNB2,STXBP4,VCL,INSR,ATP8A2,CNIH3,APC,GPC6,RELN,CCDC88A*
GO:0150063	visual system development	0.03906868461987302	*ADAMTS18,MITF,NRP1,NFIA,DCLK1,PBX1,ABCB5,MEGF11,LAMA1,WDPCP,EPHB1,CACNA1C,FLT1,SCAPER,NTRK2,DSCAM,FAT3,ATP8A2,CRB1*
GO:0090066	regulation of anatomical structure size	0.039995341080731855	*KCNMA1,MACF1,NRP1,SLC8A1,CDC42EP3,ITGA1,DISC1,FMN1,ARHGAP42,FCHSD2,DOCK4,BBS2,PRKG1,DSCAM,PRKD1,SEMA6D,FER,MAP2,MTPN,SEMA5A,TNR,VAV3*
GO:0040007	growth	0.046326760152248035	*ERBB4,ATRX,MACF1,NRP1,TEAD1,RERG,DCLK1,SLC39A12,DISC1,FMN1,APBA2,SLC1A2,S100B,PCDH15,ESR1,BBS2,SCAPER,PRKG1,DSCAM,FTO,MAGI2,AKAP6,NRG3,SEMA6D,MAP2,PRLR,MTPN,VCL,INSR,SEMA5A,ATP8A2,TNR*
GO:0048675	axon extension	0.046770671195971766	*MACF1,NRP1,DCLK1,DISC1,DSCAM,SEMA6D,MAP2,VCL,SEMA5A,TNR*
GO:0048880	sensory system development	0.04783034705883865	*ADAMTS18,MITF,NRP1,NFIA,DCLK1,PBX1,ABCB5,MEGF11,LAMA1,WDPCP,EPHB1,CACNA1C,FLT1,SCAPER,NTRK2,DSCAM,FAT3,ATP8A2,CRB1*
GO:1990138	neuron projection extension	0.04926261245446397	*MACF1,NRP1,DCLK1,SLC39A12,DISC1,S100B,DSCAM,SEMA6D,MAP2,VCL,SEMA5A,TNR*

Table S5. Overlapping groups of rDNA-contacting gene in differentiating K562 cells are co-expressed with 312 different long non-coding RNAs.

Term	Overlap	Adjusted P-value	Genes
PRICKLE2-AS3	40/100	3.0893930894723625E-8	*MACF1;CHD9;KMT2C;PRICKLE2;ZBTB20;RGPD5;RGPD8;UBR1;CACNA1C;SLC9C1;LPP;PCNX1;ATXN1;AKAP11;ZNF407;EVI5;TEAD1;PRKG1;SVIL;*

			MBD5;MON2;VPS13C;RC3H1;VPS13B;LNPEP;PLEKHA3;STON1-GTF2A1L;EML1;PHC3;MYO9A;HIPK3;FER;NFIA;PEAK1;SLMAP;KCNMA1;CCSER2;WDFY3;BIRC6;KIAA0825
LINC02827	39/100	4.6953817628259136E-8	*PATJ;CHD6;GREB1L;RGPD6;RGPD5;RGPD8;FAM214A;TMEM241;MIPOL1;PCNX2;POTED;KHDC4;SLC25A21;APBB2;SAMD12;MARCHF6;ANKRD26;PRMT8;CERS6;AUTS2;ANKRD30B;ANKRD30A;ZNF160;LRBA;DNAH14;MRTFB;TBC1D9;VPS13B;ESR1;EDAR;KIAA1217;TTC6;KCNS3;SP3;TASOR2;NEK10;ZNF236;KIAA0825;SHANK2*
LRRC7-AS1	39/100	4.6953817628259136E-8	*GABRB1;RTN1;CTNND2;NRXN1;KLHL32;SLC1A2;OTUD7A;FMN2;KIAA0513;GRM5;BRINP1;FUT9;DLGAP1;NCAM1;ERC2;SH3GL2;WASF3;OPCML;ARNT2;DTNA;CADM2;NTRK3;TMOD2;LSAMP;SLC39A12;GRIN2B;SYN2;SNAP91;GABRG1;CNKSR2;TTLL7;DLG2;SYNJ1;APC;ADGRB3;LRRC7;PPP2R2B;ASTN1;RAPGEF4*
LINC00472	37/100	3.694256115825165E-7	*CYFIP2;NFAT5;PATJ;PRKAA2;CUL5;STXBP4;KMT2C;RGPD6;ZBTB20;RGPD5;RGPD8;EFCAB6;SYNE2;KIAA1328;ERBB4;ZNF407;SLC16A7;THSD7A;RNF152;ANKRD26;MBD5;MON2;ARHGEF12;STPG2;LRBA;DNAH14;VPS13B;LNPEP;PPP2R3A;PHC3;MYO9A;MOB1B;ZNF717;WDFY3;UTRN;KIAA0825;DOCK1*
LINC01651	37/100	3.694256115825165E-7	*CRB1;GALNT13;PID1;PHLPP1;CTTNBP2;MEGF11;NRXN1;PCDH15;GRIK4;GRIK2;TRIM9;RASSF2;CDH20;DNER;TNR;NCAM1;KIF21B;CSMD3;SOX6;LRRC4C;CACNG2;GRIA4;RFTN2;GRID2;DSCAM;SPHKAP;CADM2;TCF12;LSAMP;LHFPL3;SEZ6L;FCHSD2;ADGRB3;SMOC1;DSCAML1;UNC79;ASTN1*
VWC2L-IT1	37/100	3.694256115825165E-7	*DGKG;GRIA1;GABRB1;GALNT13;SHC3;RTN1;KCNC1;MEGF11;NRXN1;KLHL32;SLC35F1;GRIK2;GRM5;RASSF2;SNTG1;TNR;LRRC4C;CACNG2;SH3GL2;GPR158;GRIA4;OPCML;CADM2;TMEM132B;TMOD2;FAM219A;LHFPL3;SEZ6L;SNAP91;EPN2;DNM3;SYNJ1;APC;ADGRB3;PPP2R2B;UNC79;ASTN1*
GSN-AS1	37/100	3.694256115825165E-7	*MACF1;ANKRD36;KMT2C;FMN1;ASAP1;LPP;DOCK10;SRGAP2C;AKAP13;FYCO1;ATXN1;SGCD;ZNF407;ABL2;HMCN1;ERC1;SRGAP2;EVI5;TEAD1;SRGAP2B;KIRREL1;MBD5;RBFOX2;DENND2B;SAMD4A;MYO5A;VPS13B;MITF;LHFPL2;TANC1;FER;PEAK1;SLMAP;CDC42EP3;BIRC6;VCL;TNRC6B*
SRGAP3-AS1	36/100	1.0404853611043894E-6	*GALNT13;RTN1;CTNND2;NRXN1;KLHL32;GRIK4;NALCN;NPAS3;NKAIN3;CDH20;LRIG1;TNR;DLGAP1;NCAM1;SRGAP3;LRRC4C;WASF3;GRIA4;RFTN2;ARNT2;GRID2;DSCAM;KCND2;CADM2;TMEM132B;NTRK3;TMOD2;TCF12;LSAMP;MAPK8IP1;DNM3;APC;ADGRB3;TBC1D5;PPP2R2B;ASTN1*
PRICKLE2-AS1	36/100	1.0404853611043894E-6	*RABGAP1;PRICKLE2;CACNA1C;LPP;FYCO1;ATXN1;AKAP11;MPRIP;ABL1;DIP2C;MPDZ;TEAD1;PRKG1;SVIL;STARD13;RBFOX2;NEGR1;AFAP1;SHISAL1;ABCC9;FOXN3;STON1-GTF2A1L;EML1;PJA2;FER;ARHGAP31;APC;PEAK1;SLMAP;KCNMA1;CCSER2;WDFY3;PPP1R12B;PDZRN3;VCL;DDR2*
RERG-AS1	36/100	1.0404853611043894E-6	*PATJ;TBC1D19;ACSS3;PRKAA2;CUL5;ANKRD36;ZBTB20;KIAA1328;ZNF407;POTED;SLC16A7;TH*

			SD7A;RNF152;ANKRD26;MON2;MBD5;STPG2;ANKRD30B;ANKRD30A;DNAH14;VPS13B;KLHL3;PHC3;MYO9A;LRP1B;MOB1B;INPP4B;TBC1D1;ZNF717;RCAN2;ERP27;WDFY3;KIAA0825;DGKI;ANKRD36B;CPEB4
LINC00689	35/100	2.2291696420807726E-6	*CRB1;GALNT13;PID1;DGKB;CTTNBP2;MEGF11;LUZP2;PCDH15;GRIK4;GRIK2;TRIM9;BRINP2;CDH20;DNER;PHACTR3;LRIG1;TNR;GSG1L;NCAM1;FYN;KIF21B;CSMD3;SOX6;GRIA4;RFTN2;GRID2;DSCAM;CADM2;TCF12;LSAMP;LHFPL3;FCHSD2;SMOC1;FGF12;DSCAML1*
LINC02720	35/100	2.2291696420807726E-6	*CUL5;PPM1L;ZBTB20;ITPR2;LDLRAD4;FAM214A;TMEM241;HS6ST3;CDH7;KIAA1328;ERBB4;FAM241A;SLC16A7;APBB2;THSD7A;RNF152;VAV3;UNC13C;MON2;STPG2;LRBA;TBC1D9;GFRA1;SCAMP1;PRLR;ESR1;PBX1;MOB1B;INPP4B;CCNG2;NEK10;ZNF678;KIAA0825;DGKI;CNTNAP5*
INHBA-AS1	35/100	2.2291696420807726E-6	*NLGN1;RTN1;SHC3;KCNC1;MYT1L;MEGF11;NTM;NRXN1;KLHL32;OTUD7A;GRIK2;GRM5;RASSF2;BRINP1;TNR;CACNG2;ANKS1B;RGS7;GRIA4;OPCML;DSCAM;TMOD2;SYT16;FAM219A;SEZ6L;GRIN2B;SNAP91;DNM3;SYNJ1;APC;ADGRB3;DSCAML1;MAPRE2;UNC79;RAPGEF4*
PACRG-AS2	35/100	2.2291696420807726E-6	*NFAT5;ACSS3;CUL5;PDE1C;PPM1L;ZBTB20;TRHDE;CDH7;HS6ST3;AP5M1;KIAA1328;TRIM2;ZNF208;SLC16A7;KIF21A;THSD7A;STXBP6;RNF152;UNC13C;FYB2;STPG2;ABCA5;PDE4D;SLC2A13;ADAM32;KLHL3;SCAMP1;MOB1B;INPP4B;TBC1D1;LRFN5;TMEM116;ERP27;DGKI;CNTNAP5*
OBI1-AS1	35/100	2.2291696420807726E-6	*GALNT13;PHLPP1;HEPACAM;LUZP2;CTNND2;NRXN1;KLHL32;PCDH15;GRIK4;ADCY2;FMN2;NKAIN3;AKAP11;CDH20;FUT9;ANKFY1;NCAM1;KIF21B;SH3GL2;WASF3;RFTN2;NTRK2;ARNT2;KCND2;CADM2;TMOD2;TCF12;LSAMP;CCDC88A;FCHSD2;APC;ADGRB3;PPP2R2B;ARHGEF7;ASB3*
SYNE1-AS1	35/100	2.2291696420807726E-6	*TBC1D19;DOCK4;PRKAA2;STXBP4;CHD9;GLIS3;PRICKLE2;ZBTB20;SYNE2;PTPRG;TTC28;ARHGAP42;SNX29;BBS9;DIP2C;TEAD1;MBD5;ARHGEF12;SLC2A13;PDE4D;PPP2R3A;CDC42BPA;MYO9A;ARHGAP24;PJA2;HIPK3;FER;ARHGAP31;LATS2;CCSER2;RAPGEF2;WDFY3;UTRN;PTPN4;DOCK1*
CXXC4-AS1	35/100	2.2291696420807726E-6	*GALNT13;DGKB;CTNND2;NRXN1;PCDH15;GRIK4;GRIK2;NALCN;DPP6;CDH20;DNER;TNR;NCAM1;NCAM2;LRRC4C;CACNG2;GPR158;GRIA4;RFTN2;GRID2;DSCAM;KCND2;CADM2;TMOD2;TCF12;LSAMP;LHFPL3;SEZ6L;DNM3;APC;ADGRB3;CNTN1;DSCAML1;UNC79;ASTN1*
TUB-AS1	35/100	2.2291696420807726E-6	*NFAT5;TBC1D19;ACSS3;CUL5;PPM1L;ZBTB20;TRHDE;TSPAN33;HS6ST3;CDH7;SNX29;AP5M1;KIAA1328;SLC16A7;KIF21A;THSD7A;SLC16A9;SLC17A1;RNF152;CUBN;STPG2;PDE4D;SLC2A13;WDR72;UNC5D;ARHGAP24;MOB1B;MSRA;PLCXD3;CLCN5;WDFY3;CNTN3;DGKI;CNTNAP5;CPEB4*
LINC02552	34/100	7.059176065441783E-6	*CRB1;PHLPP1;XYLT1;FMN1;AKAP6;FCRLA;SDCBP;SGCD;UBL3;GNG2;SCFD2;LRRTM4;CHCHD6;GNG7;ZNF106;PRAME;HMCN1;STK32A;DISC1;SOX5;MYEF2;EYA1;MYO10;PCDH7;MOK;MYO5A;MITF;NSG1;CORO2B;IGSF11;NELL1;NRG3;MDGA2;CNIH3*

ARHGEF7-IT1	34/100	7.0591760654<br>41783E-6	*DOCK4;MAML2;PPM1L;CTTNBP2;ZBTB20;AKAP11;SNTG1;SACS;NCAM1;SCAPER;MPDZ;ZNF462;MBD5;TMOD2;TCF12;KIAA0232;MYO9A;PJA2;PYGO1;DNM3;NBEA;MMP16;APC;ADGRB3;PEAK1;NFIB;TTC3;RAPGEF2;WDFY3;FAT3;TCF4;CNTN3;PIK3C3;ARHGEF7*
NMBR-AS1	34/100	7.0591760654<br>41783E-6	*ATP8A2;MAST4;GRIK1;LRP2;FRY;PRSS23;RERG;SLC7A2;FAM107B;PHF21B;ADAM29;DACH1;ERBB4;SUSD4;MALRD1;PRKACB;EDIL3;PCDH9;NEGR1;SIAH3;SEMA6D;MAGI2;CYBRD1;ESR1;GABRG2;DCDC1;DCLK1;MED13L;CACNB2;FGF14;SLCO3A1;KCNS3;FGF12;FGF10*
LINC01906	33/100	2.2802050469<br>406323E-5	*PATJ;CLSTN2;ANKRD36;KMT2C;GREB1L;LDLRAD4;SLC9C1;ZNF407;POTED;APBB2;ANKRD26;MON2;MBD5;ANKRD30B;ANKRD30A;LRBA;DNAH14;MUC19;VPS13B;ESR1;PHC3;LRP1B;PBX1;INPP4B;ZNF717;TTC6;TASOR2;ZNF678;BIRC6;ZNF236;KIAA0825;ANKRD36B;SHANK2*
MYO16-AS2	33/100	2.2802050469<br>406323E-5	*NFAT5;ACSS3;MARCHF1;CUL5;PPM1L;ZBTB20;CDH7;HS6ST3;FGD4;AP5M1;KIAA1328;TRIM2;ZNF208;SLC16A7;KIF21A;DPH6;THSD7A;RNF152;UNC13C;FYB2;STPG2;ABCA5;PDE4D;SLC2A13;ADAM32;SCAMP1;MOB1B;INPP4B;LRFN5;ERP27;DGKI;CNTNAP5;CPEB4*
LINC01741	32/100	5.7281556269<br>667816E-5	*DGKG;CRB1;GALNT13;PID1;SHC3;DGKB;MEGF11;GRIK4;SLC35F1;GRIK2;TRIM9;DNER;TNR;KIF21B;CSMD3;SOX6;LRRC4C;CACNG2;DLGAP2;GPR158;GRIA4;CA10;DSCAM;TMEM132B;TCF12;LHFPL3;EPN2;FCHSD2;SMOC1;TOX;FGF12;DSCAML1*
LINC00499	32/100	5.7281556269<br>667816E-5	*GRIA1;RTN1;HEPACAM;CTNND2;NRXN1;KLHL32;SLC1A2;ADCY2;AKAP6;FMN2;CDH20;FUT9;DLGAP1;NCAM1;ERC2;SH3GL2;WASF3;RFTN2;ARNT2;NTRK2;TMOD2;LSAMP;SLC39A12;CORO2B;GABRG1;ETNPPL;TAFA5;NRG3;APC;PPP2R2B;APBA2;RAPGEF4*
SLC8A1-AS1	32/100	5.7281556269<br>667816E-5	*MARCHF1;CHD9;RNF38;PRICKLE2;ZBTB20;CACNA1C;FRY;LPP;SLC8A1;ATXN1;AKAP11;TSPAN2;PGM5;TEAD1;PRKG1;MBD5;PDE4D;TPM1;LNPEP;FOXN3;STON1-GTF2A1L;EML1;MYO9A;PJA2;HIPK3;FER;NBEA;SLMAP;KCNMA1;CCSER2;WDFY3;PPP1R12B*
ETV5-AS1	32/100	5.7281556269<br>667816E-5	*NFAT5;ANKRD36;CHD9;KMT2C;FMN1;ASAP1;RGPD8;LYST;MYSM1;SDCBP;SPRED1;ZNF407;ZNF449;ABL2;ZNF106;HMCN1;DISC1;EVI5;ANKRD36C;MBD5;MON2;MYEF2;VPS13C;SETDB2;MYO5A;VPS13B;MITF;FER;WDPCP;BIRC6;ZNF236;ANKRD36B*
CHL1-AS1	32/100	5.7281556269<br>667816E-5	*SEMA5A;MTPN;NLGN1;PHLPP1;SLC35F1;FMN1;AKAP6;SDCBP;NKAIN3;UBL3;GNG7;ANKFY1;ZNF106;HMCN1;DIP2C;STK32A;SOX5;ARNT2;MYEF2;TMEM178B;MYO10;KAZN;MYO5A;MITF;CORO2B;IGSF11;NELL1;NRG3;TBC1D5;VPS41;CNIH3;CREB5*
PEX5L-AS2	32/100	5.7281556269<br>667816E-5	*MARCHF1;ANKRD36;CCDC126;GREB1L;EFCAB6;LRP2;NLK;DUX4;RIMS1;KIAA1328;HERC1;ERBB4;ZNF407;PSD3;POTED;HYDIN;ZNF385D;POTEC;ANKS1B;AUTS2;ANKRD30A;DNAH14;SORCS1;DCDC1;ARHGAP32;MYO3B;TTC6;NOS1AP;NEK10;ZNF236;KIAA0825;ANKRD36B*

MRPS30-DT	32/100	5.7281556269667816E-5	*PTPRT;CLSTN2;LDLRAD3;AFF3;FAM214A;TMEM241;RERG;SLC7A2;ERBB4;FAM241A;POTED;APBB2;VAV3;KDM4B;CERS6;SIAH2;LRBA;MRTFB;CYBRD1;TBC1D9;GFRA1;PRLR;ESR1;PBX1;MED13L;NAT1;TTC6;KIF16B;NEK10;BMPR1B;FSIP1;FGF10*
SLC14A2-AS1	32/100	5.7281556269667816E-5	*SLC44A5;NFAT5;TBC1D19;ACSS3;CUL5;PPM1L;ZDHHC21;NYAP2;CDH7;HS6ST3;MTMR7;MIPOL1;KIAA1328;SLC16A7;THSD7A;RNF152;SLC15A5;UNC13C;MBD5;STPG2;FANCM;PDE4D;DNAH14;POU6F2;EBF2;FOXP2;HIPK3;MOB1B;ZNF717;ZNF678;DGKI;CNTNAP5*
LINC01087	31/100	1.474656769250351E-4	*PATJ;ANKRD36;CBWD3;LDLRAD4;FAM214A;MIPOL1;PSD3;POTED;POTEG;ANKRD26;MON2;ADGRV1;ANKRD30B;ANKRD30A;LRBA;DNAH14;MRTFB;TBC1D9;VPS13B;PLXDC2;PRLR;LRP1B;PBX1;INPP4B;TTC6;CCNG2;NEK10;ZNF678;FSIP1;KIAA0825;ANKRD36B*
LINC01069	31/100	1.474656769250351E-4	*NLGN1;PHLPP1;ABCB5;AKAP6;SDCBP;UBL3;SGCD;CHCHD6;MXI1;RGS20;RTTN;PHACTR1;PRAME;ZNF106;HMCN1;STK32A;SOX6;SOX5;BCAS3;MYEF2;MYO10;MICAL3;MYO5A;CABLES1;MITF;NSG1;CORO2B;IGSF11;NRG3;SPIRE1;MDGA2*
LINC01538	31/100	1.474656769250351E-4	*NFAT5;ACSS3;MARCHF1;CUL5;PDE1C;PPM1L;ZBTB20;CDH7;HS6ST3;AP5M1;KIAA1328;TRIM2;ZNF208;SLC16A7;KIF21A;THSD7A;RNF152;UNC13C;STPG2;ABCA5;PDE4D;SLC2A13;ADAM32;SCAMP1;MOB1B;INPP4B;LRFN5;GNAQ;ERP27;DGKI;CNTNAP5*
ZNF32-AS3	31/100	1.474656769250351E-4	*GALNT13;NRXN1;GRIK4;CDH20;LRIG1;TNR;DLGAP1;NCAM1;SRGAP3;NCAM2;LRRC4C;WASF3;GRIA4;RFTN2;GRID2;DSCAM;SPHKAP;KCND2;CADM2;NTRK3;TMOD2;TCF12;LHFPL3;DNM3;FCHSD2;APC;ADGRB3;TTC3;ZMYND11;DSCAML1;ASTN1*
SLC6A1-AS1	31/100	1.474656769250351E-4	*CRB1;PID1;GALNT13;PHLPP1;MEGF11;NRXN1;GRIK4;GRIK2;TRIM9;RASSF2;CDH20;DNER;TNR;NCAM1;WASF3;GRIA4;RFTN2;DSCAM;CADM2;NTRK3;TMOD2;TCF12;LSAMP;LHFPL3;ATP2B2;KLF15;APC;ADGRB3;SMOC1;DSCAML1;ASTN1*
NAV2-AS2	31/100	1.474656769250351E-4	*OCA2;DOCK9;FMN1;ATP10A;AMBRA1;FYCO1;UBL3;SGCD;MPRIP;KIF13A;ABL1;ANKFY1;ZNF106;ERC1;STK32A;DIP2C;TEAD1;ARHGEF11;MYEF2;MYO10;DENND2B;SAMD4A;MYO5A;CABLES1;MITF;NAV2;ITPKB;TANC1;FER;ST6GALNAC3;ITGA9*
TPM1-AS	31/100	1.474656769250351E-4	*PATJ;ANKRD36;KMT2C;GREB1L;RGPD6;ZBTB20;RGPD5;RGPD8;DUX4;LPP;MYSM1;ATXN1;KIAA1328;ZNF407;POTED;POTEC;MBD5;ANKRD30A;DNAH14;RC3H1;VPS13B;PHC3;DCDC1;ZNF717;TTC6;NEK10;BIRC6;ZNF236;KIAA0825;ANKRD36B;TNRC6B*
PGR-AS1	31/100	1.474656769250351E-4	*CLSTN2;SLC40A1;GREB1L;LDLRAD4;EFCAB6;AFF3;FAM214A;GALNT10;TMEM241;RERG;ERBB4;FAM241A;ST8SIA6;APBB2;VAV3;ANKRD26;KDM4B;ANKRD30B;EYA2;SIAH2;LRBA;MRTFB;TBC1D9;GFRA1;PRLR;ESR1;PBX1;DCDC1;INPP4B;NAT1;NEK10*
LINC00862	30/100	3.297501509956885E-4	*LPGAT1;WDR26;CLSTN2;ANKRD36;KMT2C;GREB1L;LDLRAD4;MIPOL1;ODR4;KIAA1328;ZNF407;EYS;ANKRD36C;ANKRD26;ANKRD30B;ANKRD30A;D*

			NAH14;RC3H1;VPS13B;ESR1;PBX1;INPP4B;TTC6;TASOR2;ZNF678;BIRC6;ZNF236;KIAA0825;ANKRD36B;SHANK2
LINC02520	30/100	3.297501509956885E-4	*DGKG;CRB1;GALNT13;PID1;MEGF11;LAMA1;GRIK4;GRIK2;TRIM9;RELN;DNER;TNR;NCAM1;KIF21B;CSMD3;NCAM2;CACNG2;GPR158;WASF3;GRIA4;DSCAM;CADM2;TCF12;LHFPL3;SEZ6L;HS3ST4;FCHSD2;SMOC1;DSCAML1;UNC79*
LINC01602	30/100	3.297501509956885E-4	*CRB1;GALNT13;PID1;DGKB;GRIK4;GRIK2;UNC80;TRIM9;DNER;PHACTR3;TNR;FYN;KIF21B;CSMD3;SOX6;LRRC4C;CACNG2;GPR158;GRIA4;CA10;DSCAM;TMEM132B;TCF12;LHFPL3;FCHSD2;SMOC1;TOX;MDGA2;FGF12;DSCAML1*
LINC02115	30/100	3.297501509956885E-4	*ACSS3;RAP1GDS1;GRIK1;LRP2;AFF3;FAM107B;CDH7;PAK1;DACH1;ZSWIM6;MALRD1;PRKACB;STK32B;EDIL3;PACRG;SIAH3;CYBRD1;KLHL3;SCAMP1;ESR1;CDYL2;MED13L;MOB1B;CACNB2;TBC1D1;BMP2K;ERP27;BMPR1B;FGF12;FGF10*
ANKRD62P1-PARP4P3	30/100	3.297501509956885E-4	*CRB1;GALNT13;LUZP2;CTNND2;PCDH15;GRIK3;GRIK4;CDH20;LRIG1;DLGAP1;NCAM1;CSMD2;GRIA4;RFTN2;NTRK2;HFM1;DSCAM;SPHKAP;KCND2;CADM2;TCF12;DNM3;FRMD5;FCHSD2;NFIA;SMOC1;ADGRB1;TTC3;ZMYND11;DSCAML1*
AGBL4-IT1	30/100	3.297501509956885E-4	*ANKRD36;STXBP4;KMT2C;DNAH6;RGPD6;RGPD5;EFCAB6;SLC9C1;PTAR1;ZNF407;ANKRD36C;ANKRD26;DNAH11;MON2;MBD5;ANKRD30A;VPS13C;DNAH14;RANBP17;RFX3;RC3H1;VPS13B;PLEKHA3;PHC3;DCDC1;ZNF717;ZNF678;WDPCP;KIAA0825;ANKRD36B*
KCNMA1-AS3	30/100	3.297501509956885E-4	*ANKRD36;KMT2C;GREB1L;ZBTB20;EFCAB6;DUX4;MYSM1;ATXN1;KIAA1328;ZNF407;POTED;POTEC;ANKRD26;MBD5;MON2;ANKRD30A;LRBA;DNAH14;RC3H1;VPS13B;PHC3;DCDC1;ZNF717;TTC6;NEK10;BIRC6;ZNF236;KIAA0825;ANKRD36B;TNRC6B*
LRRC8C-DT	30/100	3.297501509956885E-4	*ZNF573;MACF1;PHLPP1;CHD9;LYST;DOCK10;SRGAP2C;HERC2;HERC1;MAN2A2;SACS;ANKFY1;DIP2B;LRRC8B;DISC1;SRGAP2B;ANKRD28;USP24;SGTB;PRKCB;YLPM1;MYO5A;IGSF11;CCDC88A;SYNJ1;APC;TAOK3;RFX7;WDPCP;TNRC6B*
CYP1B1-AS1	30/100	3.297501509956885E-4	*NOTCH2;CCDC186;SGMS1;SEL1L;RGPD8;LPP;RGPD4;FYCO1;EFR3A;SCAF8;ATXN1;ZNF407;PHACTR2;PDLIM5;SVIL;ZFHX3;IL1R1;BICRAL;DENND4C;LNPEP;PLXDC2;STON1-GTF2A1L;HIPK3;PARD3B;KIAA1217;ROR1;UTRN;VCL;JCAD;FKBP5*
PTPRG-AS1	30/100	3.297501509956885E-4	*IPO11;DOCK4;CUL5;STXBP4;ZBTB20;RGPD8;UBR1;PTPRG;ARHGAP42;SPRED1;ATXN1;AKAP11;ZNF449;BBS9;EVI5;TEAD1;TTC37;MBD5;ARHGEF12;ERBIN;SMAD5;MYO9A;PJA2;MOSMO;FER;TBC1D5;PEAK1;HECW2;RAPGEF2;WDFY3*
C3orf67-AS1	30/100	3.297501509956885E-4	*MTPN;DOCK4;KCNC1;MYT1L;RAP1GDS1;SLC9C1;EHBP1;ATXN1;AKAP11;ANKRD31;TANGO6;ANKRD26;MBD5;SYT1;SGTB;ATRNL1;SLC2A13;CADPS;KIAA1549L;ATP2B2;GRIN2B;SYN2;GABRG2;MOSMO;AGBL4;FER;SYNJ1;PPP2R2C;SCN8A;MBTPS2*
FGF10-AS1	30/100	3.297501509956885E-4	*PTPRT;CLSTN2;MAST4;GRIK1;LRP2;AFF3;PRSS23;FAM214A;RERG;GLI3;SLC7A2;DACH1;ERBB4;FAM241A;PIEZO2;ENPP1;ST8SIA6;MALRD1;PR*

			KACB;EDIL3;CYBRD1;TBC1D9;ESR1;DCDC1;MED13L;CACNB2;FGF14;KIF16B;BMPR1B;FGF10
LRP1-AS	30/100	3.297501509956885E-4	NOTCH2;DOCK4;TRIO;CHD9;KMT2C;RGPD8;ANTXR1;AKAP13;PCNX1;ATXN1;AKAP11;HERC1;ZNF407;EVI5;TEAD1;RNF111;CREBBP;DENND4C;EBF1;AFAP1;VPS13B;FOXN3;PLXDC2;FER;PEAK1;WDFY3;BIRC6;ZNF236;FBN1;DDR2
RORA-AS2	30/100	3.297501509956885E-4	PATJ;ANKRD36;KMT2C;GREB1L;RGPD6;ZBTB20;RORA;DUX4;KIAA1328;ZNF407;POTED;POTEG;ANKRD26;MBD5;MON2;ANKRD30A;DNAH14;RC3H1;VPS13B;PHC3;LRP1B;ZNF717;TTC6;NFIA;NEK10;BIRC6;ZNF236;KIAA0825;ANKRD36B;TNRC6B
SFTPD-AS1	30/100	3.297501509956885E-4	NFAT5;ANKRD36;CHD9;RGPD6;ZBTB20;RGPD5;RORA;ZDHHC21;SNX30;SLC9C1;PTAR1;MACROD2;ANKRD36C;DNAH11;MON2;MBD5;WSB1;VPS13C;MTUS1;PLEKHA3;BTBD9;PTPN13;CDC42BPA;PHC3;KCNQ3;GSAP;RAPGEF5;PTPN4;KIAA0825;ANKRD36B
LINC00839	29/100	7.117636586447822E-4	GALNT14;ENPEP;NLGN1;ANKRD33B;MAML2;SLC35F1;SRGAP2C;MXI1;ENPP3;SOX6;DIP2C;SRGAP2;GPR156;SRGAP2B;ANKRD6;KIRREL1;EGLN3;VCAM1;KSR1;KAZN;FRMD4A;SORCS3;IGSF11;MYO3A;SPIRE1;ALPK2;RGL1;DSCAML1;CHST3
LINC00943	29/100	7.117636586447822E-4	RTN1;KCNC1;MYT1L;NRXN1;SLC1A2;GRM3;KIAA0513;GRM5;DLGAP1;ERC2;SH3GL2;ANKS1B;OPCML;RBFOX1;TMEM132B;TMOD2;SLC4A10;SLC39A12;GRIN2B;SYN2;SNAP91;DNM3;SYNJ1;APC;ADGRB3;PPP2R2B;AMPH;SCN2A;RAPGEF4
LINC02556	29/100	7.117636586447822E-4	DGKG;GALNT13;MTMR3;PHLPP1;MEGF11;TUT4;GRIK4;GRIK2;CABIN1;CDH20;TNR;NCAM1;SRGAP3;SCAPER;GRIA4;RFTN2;SPEN;DSCAM;ZBTB16;TCF12;YLPM1;BCR;CCDC88A;FCHSD2;RFX7;SMOC1;ELMO1;FAM193A;DSCAML1
LINC00498	29/100	7.117636586447822E-4	CLSTN2;ANKRD36;KMT2C;GREB1L;ITPR2;LDLRAD4;ERBB4;ZNF407;ST8SIA6;APBB2;ANKS1B;ANKRD26;ANKRD30B;ANKRD30A;LRBA;DNAH14;VPS13B;TBC1D9;ESR1;PBX1;INPP4B;TTC6;TASOR2;ZNF678;BIRC6;ZNF236;KIAA0825;ANKRD36B;SHANK2
LINC00500	29/100	7.117636586447822E-4	SLC24A4;RTN1;PHLPP1;HEPACAM;CTNND2;NRXN1;KLHL32;SLC1A2;AKAP6;ADCY2;FMN2;GRM3;CDH20;GNG7;SH3GL2;WASF3;RFTN2;ARNT2;DTNA;TMOD2;LSAMP;SLC39A12;CORO2B;ETNPPL;IGSF11;TAFA5;NRG3;PPP2R2B;APBA2
LINC01490	29/100	7.117636586447822E-4	NECAB1;PPM1L;PTPRQ;ITPR2;AFF3;HS6ST3;CDH7;SLC16A7;GSE1;THSD7A;RNF152;SLC15A5;VAV3;UNC13C;KDM4B;STPG2;CERS6;AUTS2;ATRNL1;AGL;VPS13B;SCAMP1;DCLK1;MOB1B;INPP4B;BMPR1B;FSIP1;DGKI;MBTPS2
LINC01572	29/100	7.117636586447822E-4	GALNT13;SHC3;PHLPP1;NRXN1;GRIK4;GRIK2;TRIM9;RASSF2;DNER;TNR;NCAM1;KIF21B;LRRC4C;CACNG2;GRIA4;RFTN2;GRID2;DSCAM;CADM2;TMOD2;TCF12;LHFPL3;SEZ6L;CCDC88A;FCHSD2;APC;ADGRB3;DSCAML1;ASTN1
PPP3CB-AS1	29/100	7.117636586447822E-4	STX12;GALNT13;DOCK3;RABGAP1;PHLPP1;ATP8A1;KLHL32;GRIK4;CDH20;HECTD4;PHACTR3;LRIG1;ANKFY1;SRGAP3;SCAPER;RALGPS1;WASF3;ATP9A;RFTN2;NTRK2;NEK4;ARAP2;FAM189A2;YLPM1;TOM1L2;TAOK3;TBC1D5;ZMYND11;ASB3

WDR11-AS1	29/100	7.117636586447822E-4	*GALNT13;PHLPP1;HEPACAM;LUZP2;CTNND2;KLHL32;PCDH15;GRIK4;ADCY2;FMN2;IQCJ-SCHIP1;CDH20;NCAM1;SH3GL2;WASF3;JAM2;GRIA4;RFTN2;NTRK2;DTNA;DSCAM;KCND2;CADM2;TMOD2;LSAMP;FRMD5;PPP2R2B;ASB3;APBA2*
FRMPD3-AS1	29/100	7.117636586447822E-4	*GALNT13;LUZP2;CTNND2;NRXN1;GRIK4;ADCY2;FMN2;GRIK2;TRIM9;CDH20;DNER;LRIG1;NCAM1;LRRC4C;WASF3;GPR158;GRIA4;RFTN2;NTRK2;GRID2;DSCAM;CADM2;NTRK3;TMOD2;TCF12;LSAMP;APC;ADGRB3;ASTN1*
SHANK2-AS2	29/100	7.117636586447822E-4	*PATJ;ANKRD36;KMT2C;GREB1L;ZBTB20;LDLRAD4;DUX4;KIAA1328;ZNF407;POTEC;ANKRD26;MBD5;ANKRD30B;ANKRD30A;LRBA;DNAH14;RC3H1;VPS13B;PHC3;ZNF717;TTC6;TASOR2;ZNF678;BIRC6;ZNF236;KIAA0825;ANKRD36B;SHANK2;TNRC6B*
ADAMTS9-AS2	29/100	7.117636586447822E-4	*NRP1;ENPEP;TBC1D19;PRKAA2;STXBP4;ARHGEF28;CEP120;ZBTB20;PTPRM;RORA;LDB2;SLC8A1;TTC28;PTPRG;PKHD1;ARHGAP42;FCHO2;CEP112;MBD5;ARHGEF12;INSR;CRIM1;LNPEP;MYO9A;PJA2;HIPK3;WDFY3;ALPK2;UTRN*
LRP4-AS1	29/100	7.117636586447822E-4	*GALNT13;SHC3;DOCK3;MEGF11;NRXN1;GRIK4;GRIK2;TRIM9;RASSF2;CDH20;TNR;FYN;NCAM1;LRRC4C;CACNG2;WASF3;GRIA4;RFTN2;DSCAM;CADM2;TMEM132B;NTRK3;TMOD2;TCF12;LHFPL3;EPN2;ADGRB3;DSCAML1;ASTN1*
FAM198B-AS1	29/100	7.117636586447822E-4	*SEL1L;LDLRAD4;AFF3;FAM214A;RERG;PRRC1;FAM241A;ST8SIA6;APBB2;FLNB;TTC37;KDM4B;CERS6;LRBA;MRTFB;TBC1D9;GFRA1;PRLR;ESR1;PBX1;CDYL2;MED13L;INPP4B;TTC6;KIF16B;CCNG2;NEK10;BMPR1B;FSIP1*
MAPK10-AS1	29/100	7.117636586447822E-4	*NFAT5;KMT2C;RGPD6;ZBTB20;RGPD5;ZNF66;RGPD8;LIMD1;LPP;POTEM;POTEH;ZNF407;POTEG;ANKRD36C;ZNF462;ZFHX3;MBD5;MGA;VPS13B;PHC3;SMAD5;IQCM;ZNF717;WDFY3;CCSER1;BIRC6;UTRN;ANKRD36B;TNRC6B*
WDFY3-AS1	29/100	7.117636586447822E-4	*ZNF573;CHD9;KMT2C;ZBTB20;RGPD8;UBR1;RGPD4;PCNX1;ATXN1;AKAP11;HERC1;ZNF407;HECTD4;RNF111;MBD5;MON2;ARHGEF12;LRBA;DENND4C;VPS13B;PHC3;MYO9A;RC3H2;HIPK3;FER;WDFY3;BIRC6;ZNF236;KIAA0825*
PPP1R12A-AS1	29/100	7.117636586447822E-4	*RABGAP1;ROCK1;PPM1L;RNF38;PRICKLE2;CACNA1C;UBR1;LPP;FYCO1;HECTD4;SPOP;TSPAN2;PGM5;SCAPER;TEAD1;SVIL;FBXL17;FOXN3;STON1-GTF2A1L;EML1;PJA2;TOM1L2;SLMAP;KCNMA1;CCSER2;PPP1R12B;ZMYND11;ASB2;VCL*
THSD4-AS1	29/100	7.117636586447822E-4	*PATJ;MARCHF1;ANKRD36;GREB1L;EFCAB6;DUX4;MYSM1;KIAA1328;HERC1;ERBB4;ZNF407;PSD3;POTED;POTEC;ANKRD26;ANKRD30A;LRBA;DNAH14;VPS13B;DCDC1;ARHGAP32;ZNF717;MYO3B;TTC6;NOS1AP;NEK10;ZNF236;KIAA0825;ANKRD36B*
NKAIN3-IT1	29/100	7.117636586447822E-4	*PHLPP1;HEPACAM;LUZP2;CTNND2;NRXN1;KLHL32;GRIK4;ADCY2;FMN2;TRIM9;NKAIN3;CDH20;FUT9;NCAM1;WASF3;JAM2;GRIA4;RFTN2;NTRK2;DTNA;KCND2;CADM2;TMOD2;LSAMP;APC;ADGRB3;PPP2R2B;APBA2;ASTN1*
TSC22D1-	29/100	7.1176365864	*RERE;HDAC4;NALCN;UNC80;HERC2;AKAP11;HEC*

AS1		47822E-4	*TD4;LRIG1;DLGAP1;NPIPA1;NCAM1;SRGAP3;RALGPS1;ATP9A;MBD5;PTPRN2;CADM2;CADPS;LSAMP;PRKCA;SNAP91;DNM3;SYNJ1;TAOK3;ADGRB3;TBC1D5;MADD;CNTN3;ASTN1*
LINC01546	28/100	0.0015656727001641304	*DGKG;GALNT13;MEGF11;GRIK4;SLC35F1;GRIK2;TRIM9;BRINP2;RASSF2;SNTG1;CDH20;DNER;TNR;NCAM1;LRRC4C;CACNG2;GRIA4;RFTN2;GRID2;DSCAM;TMEM132B;LHFPL3;SEZ6L;EPN2;ADGRB3;SMOC1;DSCAML1;ASTN1*
LINC01344	28/100	0.0015656727001641304	*PATJ;GREB1L;DUX4;FAM214A;MIPOL1;HHAT;ODR4;ERBB4;PSD3;POTED;ST8SIA6;POTEC;ANKRD26;ANKRD30A;DNAH14;COG2;MRTFB;TBC1D9;PRLR;ESR1;DCDC1;LRP1B;NAT1;MYO3B;TTC6;NEK10;ATF6;KIAA0825*
LINC00446	28/100	0.0015656727001641304	*PHLPP1;ABCB5;FMN1;AKAP6;SYNPR;SDCBP;UBL3;SGCD;SCFD2;CHCHD6;RGS20;RTTN;PHACTR1;PRAME;ZNF106;STK32A;SNX8;SOX5;MYEF2;MYO10;MOK;MYO5A;CABLES1;MITF;NSG1;IGSF11;NRG3;RGS12*
LINC01761	28/100	0.0015656727001641304	*CLSTN2;ANKRD36;KMT2C;ITPR2;LDLRAD4;PCNX2;KIAA1328;ZNF407;ANKS1B;ANKRD26;ANKRD30B;ANKRD30A;MGA;DNAH14;VPS13B;ESR1;PBX1;INPP4B;TTC6;TASOR2;ZNF678;BIRC6;UTRN;ZNF236;KIAA0825;ANKRD36B;SHANK2;TNRC6B*
LINC02008	28/100	0.0015656727001641304	*GALNT13;NRXN1;GRIK4;GRIK2;FMN2;NPAS3;TRIM9;CECR2;RASSF2;SNTG1;CDH20;DNER;TNR;NCAM1;CSMD3;CACNG2;GPR158;GRIA4;RFTN2;GRID2;DSCAM;CADM2;TCF12;LHFPL3;ADGRB3;SMOC1;DSCAML1;ASTN1*
LINC02017	28/100	0.0015656727001641304	*GALNT13;RTN1;HEPACAM;LUZP2;CTNND2;KLHL32;PCDH15;GRIK4;ADCY2;FMN2;CDH20;FUT9;NCAM1;CSMD2;SH3GL2;WASF3;RFTN2;NTRK2;KCND2;CADM2;TMOD2;PDE4DIP;ETNPPL;FRMD5;TAFA5;SMOC1;APBA2;ASB3*
LINC02199	28/100	0.0015656727001641304	*PHLPP1;FMN1;SDCBP;UBL3;SCFD2;CHCHD6;GNG7;ABL2;PHACTR1;PRAME;ZNF106;HMCN1;SRGAP2;DISC1;SOX5;ARNT2;MYEF2;MYO10;ST8SIA1;MICAL3;MOK;MYO5A;MITF;NSG1;CORO2B;IGSF11;ITPKB;MDGA2*
LINC02598	28/100	0.0015656727001641304	*CRB1;GALNT13;PHLPP1;LUZP2;CTNND2;PCDH15;GRIK4;ADCY2;GRIK2;IQCJ-SCHIP1;TRIM9;SNTG1;CDH20;NCAM1;KIF21B;CSMD2;GRIA4;RFTN2;NTRK2;GRID2;DSCAM;KCND2;TCF12;LSAMP;LHFPL3;FCHSD2;DSCAML1;APBA2*
LINC02571	28/100	0.0015656727001641304	*PATJ;PDXDC1;GREB1L;AFF3;DUX4;FAM214A;TMEM241;MIPOL1;ERBB4;POTED;POTEC;CERS6;ANKRD30A;LRBA;MRTFB;TBC1D9;GFRA1;TC2N;PRLR;ESR1;PBX1;CDYL2;TTC6;DOP1B;CCNG2;MYO5C;NEK10;FSIP1*
LINC00517	28/100	0.0015656727001641304	*PATJ;ANKRD36;KMT2C;GREB1L;ZBTB20;LPP;POTEM;ATXN1;KIAA1328;POTEH;ZNF407;POTED;POTEG;ANKRD26;MBD5;ANKRD30B;ANKRD30A;DNAH14;VPS13B;PHC3;ZNF717;TTC6;NFIA;UTRN;ZNF236;KIAA0825;ANKRD36B;TNRC6B*
LINC01415	28/100	0.0015656727001641304	*GALNT13;SHC3;NRXN1;GRIK4;GRIK2;TRIM9;RASSF2;SNTG1;DNER;TNR;NCAM1;LRRC4C;CACNG2;GRIA4;RFTN2;GRID2;DSCAM;CADM2;TMOD2;TCF12;LSAMP;FAM219A;LHFPL3;SEZ6L;APC;ADGR*

			B3;TCF4;ASTN1
LINC00836	28/100	0.0015656727001641304	*GALNT13;PHLPP1;HEPACAM;LUZP2;CTNND2;KLHL32;PCDH15;GRIK4;ADCY2;FMN2;CDH20;FUT9;NCAM1;WASF3;JAM2;GRIA4;RFTN2;NTRK2;DTNA;CADM2;TMOD2;LSAMP;PDE4DIP;ETNPPL;FRMD5;PPP2R2B;SMOC1;ASB3*
SHANK2-AS3	28/100	0.0015656727001641304	*ANKRD36;KMT2C;GREB1L;ZBTB20;LDLRAD4;KIAA1328;ZNF407;ANKRD26;MBD5;ANKRD30B;ANKRD30A;LRBA;MGA;DNAH14;RC3H1;VPS13B;PHC3;ZNF717;TTC6;TASOR2;ZNF678;BIRC6;UTRN;ZNF236;KIAA0825;ANKRD36B;SHANK2;TNRC6B*
PABPC5-AS1	28/100	0.0015656727001641304	*SLC23A2;ATL1;NALCN;UNC80;CTIF;DPP6;GNG2;ZNF704;AKT3;NCAM1;DIP2C;LRRC4C;MPDZ;GPR158;GARNL3;JAZF1;ZNF287;ANKRD26;MYEF2;ANKRD30B;LSAMP;PJA2;PYGO1;DNM3;NBEA;MMP16;ADGRB3;TTC3*
MYCBP2-AS1	28/100	0.0015656727001641304	*ZNF573;MACF1;SETD2;ROCK1;CHD9;KMT2C;PRDM10;PCNX1;NIPBL;AKAP11;BTAF1;HERC1;RNF111;BPTF;USP24;SPEN;VPS13C;MGA;TRAPPC10;YLPM1;RC3H1;VPS13B;ARID1B;RC3H2;NCOR1;RFX7;BIRC6;TNRC6B*
TMEM26-AS1	28/100	0.0015656727001641304	*PTPRT;CLSTN2;LDLRAD3;AFF3;FAM214A;RERG;HHAT;FAM241A;POTED;PIEZO2;ST8SIA6;FLNB;KDM4B;LRBA;MRTFB;CYBRD1;TBC1D9;GFRA1;PRLR;ESR1;DCDC1;CDYL2;INPP4B;TTC6;KIF16B;CCNG2;NEK10;BMPR1B*
KCND3-AS1	28/100	0.0015656727001641304	*PATJ;ANKRD36;KMT2C;GREB1L;ZBTB20;EFCAB6;DUX4;POTEM;KIAA1328;POTEH;ZNF407;POTED;POTEG;POTEC;MBD5;ANKRD30A;DNAH14;VPS13B;PHC3;DCDC1;ZNF717;TTC6;NEK10;UTRN;ZNF236;KIAA0825;ANKRD36B;TNRC6B*
CNTN4-AS1	28/100	0.0015656727001641304	*APP;STX12;ATP8A1;CPQ;DOCK9;COL14A1;SDC2;PRICKLE2;PTPRM;PTPRG;ABL1;BBS9;SLIT3;STARD13;RBFOX2;ARHGEF12;CNTN6;LAMB1;PJA2;PARD3B;TBC1D5;SLC27A6;CNTN4;UTRN;ZMYND11;DOCK1;JCAD;ITGA9*
PRICKLE2-AS2	28/100	0.0015656727001641304	*NFAT5;ANKRD36;CHD9;KMT2C;RGPD6;PRICKLE2;ZBTB20;RGPD5;RGPD8;LPP;MYSM1;ATXN1;ZNF407;ANKRD36C;MBD5;MON2;ANKRD30A;VPS13C;RC3H1;VPS13B;PHC3;FER;WDFY3;BIRC6;UTRN;KIAA0825;ANKRD36B;TNRC6B*
NR2F2-AS1	28/100	0.0015656727001641304	*NRP1;NFAT5;GALNT14;ENPEP;PRKAA2;STXBP4;ARHGEF28;ZBTB20;LDB2;SLC6A3;CNDP2;TTC28;PKHD1;ARHGAP42;SNX29;GRB10;ARSB;MBD5;VCAM1;INSR;CRIM1;PPP2R3A;MYO9A;ARHGAP24;CLCN5;WDFY3;ALPK2;TRABD2B*
CLSTN2-AS1	28/100	0.0015656727001641304	*CLSTN2;ANKRD36;KMT2C;ITPR2;LDLRAD4;KIAA1328;ZNF407;ANKS1B;ANKRD26;ANKRD30B;ANKRD30A;LRBA;MGA;DNAH14;VPS13B;PHC3;PBX1;INPP4B;TTC6;TASOR2;ZNF678;BIRC6;UTRN;ZNF236;KIAA0825;ANKRD36B;SHANK2;TNRC6B*
LINC00305	27/100	0.003311681967244395	*ZNF397;ATP8A2;KDM1B;SYCP1;PIGN;DNAH5;PLD5;HTR2C;FHIT;ADAMTSL1;TTR;ZSCAN30;ERBB4;KIF13A;RPRD1A;ZNF385D;PDE6A;TRPM3;OR4C46;NTNG1;WWOX;KCNH5;TRAPPC8;GABRG3;NAT1;TAFA1;GALNTL6*
LINC01567	27/100	0.003311681967244395	*NFAT5;MARCHF1;CUL5;PPM1L;RGPD6;ZBTB20;RGPD5;CDH7;HS6ST3;AP5M1;KIAA1328;TRIM2;SLC16A7;THSD7A;RNF152;UNC13C;ANKRD36C;MB*

			D5;STPG2;PDE4D;ADAM32;PLEKHA3;PIAS2;MOB1B;ZNF717;DGKI;CNTNAP5
LINC00945	27/100	0.003311681967244395	*CRB1;PHLPP1;CTNND2;ILDR2;GRIK4;SLC35F1;AKAP6;DOCK10;TRIM9;RASSF2;FYN;NCAM1;DISC1;GRIA4;RFTN2;ARNT2;GRID2;DSCAM;ST8SIA1;TMOD2;TCF12;LSAMP;LHFPL3;CORO2B;IGSF11;CCDC88A;APBA2*
LINC01677	27/100	0.003311681967244395	*DGKG;CRB1;GALNT13;RTN1;HEPACAM;MEGF11;CTNND2;KLHL32;GRIK4;GRIK2;TRIM9;CDH20;DNER;PHACTR3;TNR;NCAM1;GRIA4;RFTN2;NTRK2;GRID2;DSCAM;CADM2;LSAMP;ADGRB3;SMOC1;DSCAML1;APBA2*
LINC01945	27/100	0.003311681967244395	*SH3KBP1;SEMA3A;AKAP6;PCMTD2;SDCBP;GNG2;GNG7;PRAME;HMCN1;SRGAP2;SOX5;MAP4K4;KIRREL1;EYA1;MYO10;PCDH7;HMGA2;SORCS1;CORO2B;AIMP1;IGSF11;MOSMO;MMP16;CNTN4;B4GALT6;FREM1;CNIH3*
LINC01351	27/100	0.003311681967244395	*HEPACAM;CTNND2;NRXN1;GRIK4;SLC35F1;FMN2;TRIM9;RASSF2;CDH20;DNER;NCAM1;BRINP3;WASF3;GRIA4;RFTN2;GRID2;DSCAM;CADM2;NTRK3;TMOD2;LSAMP;LHFPL3;EPN2;ADGRB3;PPP2R2B;APBA2;ASTN1*
LINC02006	27/100	0.003311681967244395	*NFAT5;ACSS3;CUL5;PPM1L;ABCA13;CDH7;HS6ST3;AP5M1;KIAA1328;TRIM2;ZNF208;SLC16A7;KIF21A;THSD7A;SAMD12;RNF152;UNC13C;STPG2;ABCA5;PDE4D;SCAMP1;MOB1B;INPP4B;TMEM116;ERP27;DGKI;CNTNAP5*
LINC02058	27/100	0.003311681967244395	*GRIA1;GALNT13;PHLPP1;LUZP2;CTNND2;NRXN1;KLHL32;GRIK4;ADCY2;FMN2;RASSF2;CDH20;FUT9;NCAM1;KIF21B;WASF3;GRIA4;RFTN2;NTRK2;GRID2;DTNA;DSCAM;CADM2;TMOD2;LSAMP;APC;ADGRB3*
LINC02510	27/100	0.003311681967244395	*COL14A1;LPP;NTF3;HMCN2;TSPAN2;EDIL3;ZNF367;PLXNA4;SPECC1;ANKRD30A;NUBPL;PDE4D;TPM1;TOP3A;FBXO32;LRP1B;TTLL7;FAM135B;COL4A2;LRRC7;SLMAP;EOGT;ITGA8;ULK2;PPP1R12B;ASB2;VCL*
LINC00641	27/100	0.003311681967244395	*RTN1;DGKB;CTNND2;NRXN1;OTUD7A;FMN2;NALCN;DPP6;FUT9;NCAM1;LRRC4C;SH3GL2;WASF3;CADM2;TMOD2;LSAMP;ZDHHC17;SNAP91;DNM3;CNKSR2;CCDC88A;SYNJ1;APC;ADGRB3;PPP2R2B;CNTN1;ASTN1*
LINC00564	27/100	0.003311681967244395	*PTPRQ;ITPR2;SIPA1L2;AFF3;PRSS23;ALCAM;ZNRF3;ERBB4;ANKRD31;ENPP1;LARGE1;SLC37A2;ATRNL1;SIAH2;PXDNL;ERLIN2;PRLR;GABRG2;PARP8;KITLG;CNKSR3;BMPR1B;VSTM2A;FSIP1;PDZRN3;FBXL7;FGF10*
LINC01924	27/100	0.003311681967244395	*NFAT5;ACSS3;CUL5;PPM1L;SLC40A1;CDH7;HS6ST3;SPATA48;AP5M1;KIAA1328;TRIM2;ZNF208;SLC16A7;THSD7A;RNF152;UNC13C;FYB2;STPG2;PDE4D;SCAMP1;MOB1B;INPP4B;TBC1D1;TMEM116;ERP27;DGKI;CNTNAP5*
LINC01255	27/100	0.003311681967244395	*CLSTN2;ANKRD36;KMT2C;ITPR2;LDLRAD4;PCNX2;KIAA1328;ZNF407;ANKS1B;ANKRD26;ANKRD30B;ANKRD30A;LRBA;DNAH14;VPS13B;ESR1;PBX1;INPP4B;TTC6;TASOR2;BIRC6;UTRN;ZNF236;KIAA0825;ANKRD36B;SHANK2;TNRC6B*
WDFY3-AS2	27/100	0.003311681967244395	*NLGN1;CTNND2;KLHL32;GRIK4;ADCY2;FMN2;IQCJ-*

			SCHIP1;CDH20;NCAM1;SH3GL2;WASF3;JAM2;GRIA4;BBS2;RFTN2;NTRK2;DTNA;CADM2;TMOD2;MAGI2;APC;ADGRB3;PPP2R2B;SPIRE1;CPE;WDFY3;DSCAML1
MAPRE3-AS1	27/100	0.003311681967244395	*NFAT5;MARCHF1;CUL5;PPM1L;ANKRD36;KMT2C;RGPD6;ZBTB20;RGPD5;SLC9C1;DUX4;KIAA1328;SLC16A7;THSD7A;RNF152;MBD5;STPG2;RC3H1;VPS13B;PHC3;MOB1B;ZNF717;BIRC6;ZNF236;DGKI;ANKRD36B;TNRC6B*
RORB-AS1	27/100	0.003311681967244395	*SLC23A2;ABCD2;RTN1;KCNC1;MYT1L;FRMPD4;ATL1;SLC1A2;RORB;KIAA0513;SV2B;SPOCK1;CTNNA2;PPFIA2;RGS7;RBFOX1;SLC4A10;SYT16;ATP2B2;SYN2;SNAP91;CNKSR2;TAFA4;DLG2;SCN8A;SCN2A;ASTN1*
ARHGEF7-AS1	27/100	0.003311681967244395	*MAML2;PPM1L;ATL1;NRXN1;FRY;NOL4;CTIF;DPP6;NCAM1;DIP2C;MPDZ;WASF3;CADM2;TMOD2;TCF12;EBF1;KIAA0232;PJA2;DNM3;SGSM1;NBEA;APC;ADGRB3;WDFY3;TCF4;TLN2;ARHGEF7*
NCAM1-AS1	27/100	0.003311681967244395	*GALNT13;CTNND2;NRXN1;GRIK4;FMN2;GRIK2;NALCN;TRIM9;DPP6;DNER;NCAM1;LRRC4C;GPR158;GRIA4;RFTN2;GRID2;DSCAM;CADM2;NTRK3;TMOD2;LSAMP;MAPK8IP1;DNM3;ADGRB3;PPP2R2B;UNC79;ASTN1*
UST-AS1	27/100	0.003311681967244395	*ZNF573;MACF1;NFAT5;PPM1L;CHD9;KMT2C;WDR41;RGPD6;ZBTB20;RGPD5;RGPD8;SLC9C1;ZNF407;SLC16A7;SCAPER;EVI5;ZNF462;MBD5;PHC3;PYGO1;MOB1B;FER;PDP2;CCSER2;PTPN4;DGKI;TNRC6B*
KAZN-AS1	27/100	0.003311681967244395	*PATJ;ANKRD36;KMT2C;GREB1L;EFCAB6;DUX4;MYSM1;ERBB4;PSD3;POTED;POTEC;NTNG1;ANKRD30A;LRBA;DNAH14;VPS13B;TC2N;PRLR;DCDC1;ARHGAP32;MYO3B;TTC6;TTC3;NEK10;ZNF236;KIAA0825;ANKRD36B*
DAAM2-AS1	27/100	0.003311681967244395	*PHLPP1;HEPACAM;LUZP2;CTNND2;NRXN1;KLHL32;GRIK4;ADCY2;FMN2;NKAIN3;CDH20;FUT9;NCAM1;WASF3;JAM2;RFTN2;NTRK2;ARNT2;DTNA;CADM2;TMOD2;LSAMP;GABRG1;ETNPPL;APC;ADGRB3;PPP2R2B*
ID2-AS1	27/100	0.003311681967244395	*GALNT13;HEPACAM;LUZP2;CTNND2;NRXN1;KLHL32;GRIK4;ADCY2;FMN2;IQCJ-SCHIP1;CDH20;FUT9;NCAM1;SH3GL2;WASF3;BBS2;RFTN2;NTRK2;DTNA;CADM2;TMOD2;PRKCA;GABRG1;ETNPPL;PTPRD;APC;PPP2R2B*
SMAD9-IT1	27/100	0.003311681967244395	*RABGAP1;ATP8A1;MAML2;ZBTB20;POTEM;AKAP11;POTEH;HECTD4;SCAPER;MPDZ;ZNF462;RIC3;MBD5;TMEM178B;ZBTB16;YLPM1;ZDHHC17;MYO9A;PYGO1;TJP1;NFIA;PEAK1;CCSER2;RAPGEF2;WDFY3;UTRN;ZMYND11*
OIP5-AS1	27/100	0.003311681967244395	*ZNF573;CHD9;IREB2;UBR1;PTAR1;PCNX1;BTAF1;HERC1;TRPM7;RNF111;BPTF;USP8;AQR;PRKCB;VPS13C;MGA;RC3H1;FAM126B;MYO9A;RC3H2;PIAS1;CCDC88A;KANSL1;RFX7;GNAQ;ZNF236;TNRC6B*
EDIL3-DT	27/100	0.003311681967244395	*NRP1;PRICKLE2;MPRIP;CHSY3;DIP2C;EDIL3;GPC6;ARSB;PRKG1;RBFOX2;PDE4D;TPM1;AFAP1;EML1;PTPRD;TTLL7;COL4A2;SLMAP;EOGT;ITGA8;ULK2;CDH13;ROR1;RAPGEF5;PPP1R12B;ASB2;VCL*
FRMD6-AS1	27/100	0.0033116819	*CHD6;GREB1L;LDLRAD3;DUX4;GLI3;MIPOL1;RP*

		67244395	*S6KA5;ZNF407;POTED;ABL2;POTEC;SUPT16H;ANKRD26;FANCM;ANKRD30A;DNAH14;MRTFB;EXOC6B;TBC1D9;MCC;PRLR;ESR1;DCDC1;FRMD6;TTC6;NEK10;ZNF236*
LINC02051	26/100	0.0065133504181173075	*DGKG;GALNT13;SLC24A3;RABGAP1;SLC35F1;GRIK2;SEL1L2;TRIM9;RASSF2;HMCN2;TMEM108;TNR;NCAM1;PLXNA4;GRIA4;SPECC1;DSCAM;TCF12;LHFPL3;EPN2;TTLL7;LRRC7;SMOC1;ITGA8;ASB2;ASTN1*
LINC01739	26/100	0.0065133504181173075	*IGSF3;ABCB5;FMN1;LYST;SDCBP;SGCD;UBL3;SCFD2;CHCHD6;ABL2;RGS20;PHACTR1;PRAME;ZNF106;HMCN1;STK32A;ARHGEF11;MYEF2;MYO10;MOK;MYO5A;CABLES1;MITF;NSG1;PARVB;IGSF11*
LINC01293	26/100	0.0065133504181173075	*IGSF3;ABCB5;FMN1;LYST;SDCBP;SGCD;UBL3;CHCHD6;ABL2;RGS20;PHACTR1;PRAME;ZNF106;HMCN1;SRGAP2;ARHGEF11;BCAS3;MYEF2;MYO10;SETDB2;MOK;MYO5A;CABLES1;MITF;NSG1;IGSF11*
LINC01640	26/100	0.0065133504181173075	*PPM1L;PRICKLE2;NRXN3;CACNA1C;LPP;MED15;FYCO1;INPP5A;KIAA1328;ABL1;PGM5;LARGE1;PRKG1;RALGPS1;MAGI1;ZFHX3;RBFOX2;FOXN3;STON1-GTF2A1L;EML1;PARD3B;ASXL3;SLMAP;KCNMA1;ZNF74;CCSER2*
LINC02151	26/100	0.0065133504181173075	*GALNT13;SHC3;MEGF11;GRIK4;GRIK2;UNC80;DPP6;SNTG1;DNER;TNR;NCAM1;LRRC4C;CACNG2;GPR158;GRIA4;CA10;DSCAM;LHFPL3;SEZ6L;EPN2;SLC8A3;ADGRB3;SMOC1;DSCAML1;UNC79;ASTN1*
LINC02030	26/100	0.0065133504181173075	*SLC24A3;BNC2;LUZP2;CTNND2;PCDH15;ADCY2;KLHL13;CACNA1C;PHF21B;RIMS2;CDH20;LRIG1;NCAM1;RFTN2;KCND2;LRRC49;SHISAL1;UNC5D;TAFA5;GHRH;ADGRB1;PRR16;PPP1R12B;PLCB1;PDZRN3;APBA2*
LINC02024	26/100	0.0065133504181173075	*RYR2;KMT2C;ZBTB20;POTEM;KIAA1328;POTEH;HECTD4;POTEG;POTEB;ZNF462;ZFHX3;EPHA6;POTEB2;POTEB3;ZBTB16;PDE4D;MGA;VPS13B;PHC3;TTC6;NFIA;FAT3;UTRN;DGKI;ANKRD36B;TNRC6B*
LINC02226	26/100	0.0065133504181173075	*NLGN1;PHLPP1;CTNND2;GRIK4;SLC35F1;AKAP6;FMN2;TRIM9;UBL3;GNG7;DNER;FYN;NCAM1;SOX6;RFTN2;ARNT2;MYEF2;ST8SIA1;MAGI2;LSAMP;CORO2B;IGSF11;PPP2R2B;MDGA2;APBA2;ASTN1*
LINC01088	26/100	0.0065133504181173075	*GALNT13;HPSE2;HEPACAM;LUZP2;CTNND2;KLHL32;GRIK4;ADCY2;FMN2;CDH20;FUT9;NCAM1;WASF3;JAM2;RFTN2;NTRK2;DTNA;LSAMP;PDE4DIP;GABRG1;ETNPPL;FRMD5;PPP2R2B;CNTN1;CPE;ASB3*
LINC02764	26/100	0.0065133504181173075	*GALNT13;HEPACAM;LUZP2;CTNND2;KLHL32;GRIK4;ADCY2;FMN2;IQCJ-SCHIP1;CDH20;FUT9;NCAM1;SH3GL2;WASF3;JAM2;RFTN2;NTRK2;PACRG;DTNA;CADM2;TMOD2;TAFA5;NRG3;PPP2R2B;SPIRE1;APBA2*
LINC02157	26/100	0.0065133504181173075	*NRP1;GALNT14;ENPEP;PRKAA2;ARHGEF28;RORA;SLC6A3;SYNE2;CNDP2;TTC28;PKHD1;ARHGAP42;SNX29;ARSB;VCAM1;INSR;PPP2R3A;MYO9A;PPFIBP1;CLCN5;EVC;GSAP;WDFY3;ALPK2;TRABD*

			2B;CREB5
LINC02427	26/100	0.0065133504181173075	*CTNND2;KLHL32;OTUD7A;GRIK4;ADCY2;FMN2;NALCN;DPP6;CDH20;DNER;NCAM1;SH3GL2;WASF3;BBS2;ARNT2;DTNA;CADM2;TMOD2;MAGI2;LSAMP;MAPK8IP1;ADGRB3;PPP2R2B;CNTN1;CPE;ASTN1*
LINC02334	26/100	0.0065133504181173075	*NFAT5;CUL5;ANKRD36;CHD9;RGPD6;ZBTB20;RGPD5;PTAR1;SLC16A7;RNF152;ANKRD36C;MBD5;MON2;STPG2;VPS13C;PDE4D;RC3H1;LNPEP;PLEKHA3;PHC3;PIAS2;MOB1B;ZNF717;DGKI;ANKRD36B;TNRC6B*
LINC02338	26/100	0.0065133504181173075	*ABCD2;MYT1L;HEPACAM;CTNND2;SLC1A2;FMN2;KIAA0513;CTNNA2;ERC2;SH3GL2;GABRA2;RBFOX1;DTNA;TMOD2;SLC4A10;SYT16;SLC39A12;GRIN2B;SYN2;CNKSR2;DLG2;APC;PPP2R2B;TAFA1;SCN2A;ASTN1*
RAMP2-AS1	26/100	0.0065133504181173075	*PTPRT;GALNT13;CLSTN2;GRIK4;LDLRAD3;LDLRAD4;GLI3;SCAF8;CDH20;LRIG1;APBB2;MPDZ;WASF3;RFTN2;ARNT2;ANKRD26;MAGI2;EBF1;GFRA1;ESR1;ZDHHC17;PBX1;ARHGAP31;TTC3;ULK2;FBXL7*
GPC6-AS2	26/100	0.0065133504181173075	*NFAT5;CUL5;ANKRD36;CHD9;ZBTB20;LPP;SLC16A7;RNF152;GPC6;ANKRD36C;MBD5;STPG2;VPS13C;PDE4D;FNDC3B;RC3H1;LNPEP;PLEKHA3;PHC3;PIAS2;MOB1B;ZNF717;WDPCP;KIAA0825;DGKI;ANKRD36B*
DOCK4-AS1	26/100	0.0065133504181173075	*NFAT5;ANKRD36;KMT2C;RGPD6;ZBTB20;RGPD5;ZDHHC21;RGPD8;SLC9C1;MYSM1;ATXN1;ZNF407;ANKRD36C;MBD5;MON2;VPS13C;DNAH14;RC3H1;VPS13B;PLEKHA3;PHC3;ZNF717;WDPCP;BIRC6;KIAA0825;ANKRD36B*
LAMP5-AS1	26/100	0.0065133504181173075	*DOCK3;TENM3;TSHZ2;CELF4;CACNA1E;CLTCL1;SRGAP3;ERC2;ADAMTS9;ZNF521;RGS7;GOLGA8S;RBFOX1;PRMT8;GOLGA8T;CADM1;GRID1;IL17RA;CNKSR2;MMP16;AMPH;FAT3;CNTN3;TCF4;ANKRD18A;PDZRN4*
GLCCI1-DT	26/100	0.0065133504181173075	*STX12;DOCK3;ATP8A1;PPM1L;RNF38;DPP6;NCAM1;FYN;SCAPER;ZSWIM5;NDFIP1;GRID1;TCF12;FBXL17;PJA2;ARHGAP31;TG;FCHSD2;PDP2;NBEA;SYNJ1;TBC1D5;ELMO1;CCSER2;RAPGEF2;ZMYND11*
NCKAP5-AS1	26/100	0.0065133504181173075	*PATJ;ANKRD36;KMT2C;GREB1L;ZBTB20;DUX4;MYSM1;ATXN1;KIAA1328;ZNF407;POTEC;ANKRD26;MBD5;ANKRD30A;DNAH14;RC3H1;VPS13B;PHC3;ZNF717;TTC6;ZNF678;BIRC6;CDK12;ZNF236;KIAA0825;ANKRD36B*
MSC-AS1	26/100	0.0065133504181173075	*NRP1;GALNT14;ENPEP;MTMR2;BNC2;MAP3K7CL;DYSF;ASAP1;GLIS1;SDCBP;ARHGAP42;SNX29;CHSY3;DIP2C;GPC6;ARSB;KIRREL1;VCAM1;MITF;LHFPL2;GNG12;SNAI2;PLIN2;ALPK2;RGL1;LRP12*
WWTR1-IT1	26/100	0.0065133504181173075	*ANKRD36;KMT2C;TSHZ2;RGPD6;ZBTB20;RGPD5;RGPD8;LPP;ATXN1;KIAA1328;ZNF407;ANKRD36C;ZNF462;ZFHX3;MBD5;MGA;RC3H1;VPS13B;PHC3;ZNF717;WDPCP;BIRC6;ZNF236;KIAA0825;ANKRD36B;TNRC6B*
SLIT2-IT1	26/100	0.0065133504181173075	*NFAT5;CUL5;PPM1L;RGPD6;ZBTB20;RGPD5;RGPD8;KIAA1328;SLC16A7;THSD7A;RNF152;UNC13C;MBD5;STPG2;PDE4D;VPS13C;VPS13B;ADAM32*

			;PLEKHA3;PHC3;PIAS2;MOB1B;INPP4B;WDPCP;DGKI;CNTNAP5
SEMA6A-AS2	26/100	0.0065133504181173075	*NLGN1;PHLPP1;FMN1;NKAIN3;UBL3;MXI1;ANKFY1;MAPK1;PHACTR1;DIP2C;SOX6;SRGAP2B;ARNT2;MYEF2;MYO10;MICAL3;MYO5A;CABLES1;MITF;IGSF11;ITPKB;NRG3;APC;SPIRE1;MDGA2;MAPRE2*
SH3TC2-DT	26/100	0.0065133504181173075	*FMN1;LYST;PPM1F;COL19A1;SDCBP;GNPTAB;SCFD2;GNG7;MGAT5;ABL2;PHACTR1;ZNF106;HMCN1;SRGAP2;ANKRD28;COL22A1;MOK;MYO5A;NFATC2;MITF;LHFPL2;IGSF11;MOSMO;MELTF;B4GALT6;CNIH3*
KCNIP1-AS1	26/100	0.0065133504181173075	*MEGF11;TMPRSS3;ILDR2;SLC35F1;FAM171A1;RIC8B;SV2C;MAN2A2;SNTG1;DNER;TSPAN3;FAM81A;PAK5;TMEM132B;NTRK3;RANBP17;TCF12;CACNA2D3;PRKCA;ARHGAP26;SEZ6L;CDC42BPA;MAPK8IP1;LRFN2;CLDN10;FAT3*
PKIA-AS1	26/100	0.0065133504181173075	*NFAT5;ACSS3;CUL5;PPM1L;RGPD6;ZBTB20;RGPD5;ZDHHC21;CDH7;HS6ST3;AP5M1;KIAA1328;SLC16A7;THSD7A;RNF152;UNC13C;MBD5;STPG2;ABCA5;PDE4D;PHC3;MOB1B;INPP4B;ZNF717;DGKI;CNTNAP5*
NAV2-AS3	26/100	0.0065133504181173075	*NFAT5;RGPD6;ZBTB20;RGPD5;ZDHHC21;SLC9C1;AP5M1;KIAA1328;TRIM2;SLC16A7;KIF21A;THSD7A;SCAPER;ANKRD36C;MBD5;MON2;ARHGEF12;VPS13C;FBXL17;PLEKHA3;KLHL3;MOB1B;ZNF678;PTPN4;KIAA0825;CPEB4*
NAV2-AS1	26/100	0.0065133504181173075	*DOCK3;DOCK9;UBL3;SNX25;BBS9;STK32A;TEAD1;RXRG;MYEF2;CADM1;MYO10;DENND2B;SAMD4A;FAM189A2;CABLES1;MITF;NAV2;FRMD3;ITPKB;TANC1;TG;TCERG1L;IFT81;SLC27A6;ST6GALNAC3;ITGA9*
OPCML-IT1	26/100	0.0065133504181173075	*CRB1;MEGF11;PCDH15;GRIK3;GRIK4;SLC35F1;GRIK2;DNER;TMEM108;TNR;FYN;NCAM1;GRIA4;OPCML;GRID2;CA10;DSCAM;CADM2;TMEM132B;TCF12;LSAMP;LHFPL3;EPN2;SMOC1;DSCAML1;APBA2*
TSPAN18-AS1	26/100	0.0065133504181173075	*ENPEP;DYSF;RORA;CACNA1C;SLC9C1;LPP;TTC28;PTAR1;ZNF449;PHACTR2;PRKG1;PDK1;RBM33;MBNL1;MBD5;KSR1;ZNF160;INSR;ATP11C;RC3H1;EML1;PHC3;MYO9A;AGO2;PPP1R12B;TNRC6B*
GRM5-AS1	26/100	0.0065133504181173075	*RTN1;SHC3;KCNC1;MYT1L;NRXN1;SLC1A2;KIAA0513;GRM5;BRINP1;DLGAP1;ERC2;SH3GL2;ANKS1B;OPCML;RBFOX1;TMOD2;SLC4A10;SYT16;GRIN2B;SYN2;SNAP91;SYNJ1;ADGRB3;SCN8A;TAFA1;SCN2A*
DLG2-AS2	26/100	0.0065133504181173075	*RYR2;KMT2C;RGPD6;ZBTB20;RGPD5;ZDHHC21;RGPD8;LPP;RGPD4;POTEM;ATXN1;POTEH;TMEM225;POTEG;ANKRD36C;ZFHX3;MBD5;PLEKHA3;PHC3;PYGO1;DLG2;ZNF717;NFIA;PRR16;UTRN;PTPN4*
PAPPA-AS1	26/100	0.0065133504181173075	*NFAT5;TSPAN33;FSTL1;CMIP;SYNE2;PKHD1;SNX29;SLC17A1;GPC6;PAQR5;PDE4D;SLC2A13;SLC12A1;CRIM1;KCNJ15;LAMB1;PPP2R3A;GNG12;ARHGAP24;MYO1E;CLCN5;EVC;LATS2;PAPPA;FAT1;FBN1*
MAPT-AS1	26/100	0.0065133504181173075	*CRB1;GALNT13;RTN1;CTNND2;NRXN1;GRIK4;ADCY2;FMN2;GRIK2;TRIM9;CDH20;DNER;NCAM1;LRRC4C;GRIA4;RFTN2;GRID2;DSCAM;CADM2;TMO*

			D2;TCF12;LSAMP;ADGRB3;SMOC1;DSCAML1;ASTN1
LDLRAD4-AS1	26/100	0.0065133504181173075	*ANKRD36;KMT2C;ZBTB20;LDLRAD4;MYSM1;ATXN1;KIAA1328;ZNF407;BPTF;ANKRD26;MBD5;MON2;ANKRD30A;MGA;DNAH14;RC3H1;VPS13B;PHC3;ZNF717;ZNF678;WDPCP;BIRC6;ZNF236;KIAA0825;ANKRD36B;TNRC6B*
FBXW7-AS1	26/100	0.0065133504181173075	*MYT1L;FRMPD4;KIAA0513;AKAP11;HERC1;SV2B;ERC2;SCAPER;RNF111;RBFOX1;SYT1;SGTB;PRKCB;SLC4A10;GRIN2B;SYN2;GABRG2;SNAP91;PJA2;CNKSR2;SYNJ1;APC;SCN8A;RAPGEF2;SCN2A;RAPGEF4*
LINC02794	25/100	0.012280094346006434	*NRP1;GALNT14;ENPEP;ANKRD33B;PRKAA2;ARHGEF28;USH1C;LRP2;SLC6A3;CNDP2;PKHD1;SNX29;ENPP3;SLC17A1;CUBN;EGLN3;VCAM1;INSR;CRIM1;PPP2R3A;MYO9A;CLCN5;ALPK2;TRABD2B;CREB5*
LINC00710	25/100	0.012280094346006434	*MACF1;NFAT5;ANKRD36;CHD9;KMT2C;ZBTB20;RORA;ZDHHC21;SLC9C1;LPP;ATXN1;MACROD2;EVI5;ANKRD36C;DNAH11;MBD5;MON2;VPS13C;RC3H1;VPS13B;PLEKHA3;PHC3;KIAA0825;ANKRD36B;TNRC6B*
LINC00349	25/100	0.012280094346006434	*SH3KBP1;SEMA3A;AKAP6;SDCBP;GNG2;SCFD2;GNG7;PRAME;HMCN1;SRGAP2;SOX5;BCAS3;KIRREL1;EYA1;MYO10;PCDH7;HMGA2;LHFPL2;CORO2B;AIMP1;IGSF11;MOSMO;B4GALT6;FREM1;CNIH3*
LINC00387	25/100	0.012280094346006434	*PTPRT;TMEM63C;GREB1L;LDLRAD4;FAM214A;HHAT;PSD3;FAM241A;POTED;ST8SIA6;APBB2;PCMTD1;VAV3;KDM4B;CERS6;NEBL;SIAH2;MRTFB;TBC1D9;GFRA1;PRLR;ESR1;PBX1;NELL2;NAT1*
LINC00456	25/100	0.012280094346006434	*IGSF3;ABCB5;FMN1;LIMD1;LYST;FCRLA;SDCBP;SGCD;UBL3;GNPTAB;CHCHD6;ABL2;PHACTR1;ZNF106;PRAME;MORC1;MINAR1;ARHGEF11;MYEF2;MOK;MYO5A;CABLES1;MITF;NSG1;CNIH3*
LINC01865	25/100	0.012280094346006434	*NECAB1;RTN1;MYT1L;FRMPD4;ATL1;SLC1A2;IQCJ-SCHIP1;KIAA0513;ERC2;ZNF521;SH3GL2;RBFOX1;SGTB;TMOD2;SLC4A10;KIAA1549L;SLC39A12;GRIN2B;SYN2;CNKSR2;SYNJ1;PPP2R2B;TAFA1;AMPH;SCN2A*
LINC02676	25/100	0.012280094346006434	*PATJ;ANKRD36;CBWD3;GXYLT2;MYSM1;FAM214A;MIPOL1;PSD3;POTED;POTEG;MBD5;MON2;ADGRV1;ANKRD30A;CFAP70;LRBA;DNAH14;PLXDC2;LRP1B;ZNF717;TTC6;NEK10;ZNF678;KIAA0825;ANKRD36B*
LINC00844	25/100	0.012280094346006434	*GALNT13;RTN1;PHLPP1;HEPACAM;LUZP2;CTNND2;KLHL32;GRIK4;ADCY2;FMN2;CDH20;FUT9;PHACTR3;NCAM1;WASF3;RFTN2;NTRK2;DTNA;TMOD2;LSAMP;KLF15;ETNPPL;TAFA5;PPP2R2B;SMOC1*
LINC00391	25/100	0.012280094346006434	*HEPACAM;CTNND2;NRXN1;GRIK4;ADCY2;FMN2;TRIM9;CDH20;DNER;NCAM1;WASF3;JAM2;GRIA4;RFTN2;DTNA;DSCAM;CADM2;NTRK3;TMOD2;LSAMP;APC;ADGRB3;PPP2R2B;APBA2;ASTN1*
LINC01998	25/100	0.012280094346006434	*GRIA1;HEPACAM;LUZP2;CTNND2;KLHL32;GRIK4;ADCY2;FMN2;IQCJ-SCHIP1;NKAIN3;CDH20;FUT9;NCAM1;SH3GL2;JAM2;WASF3;RFTN2;NTRK2;DTNA;CADM2;TMOD2;LSAMP;APC;PPP2R2B;APBA2*

LINC00504	25/100	0.012280094346006434	*PTPRT;IGSF3;LDLRAD3;FMN1;AFF3;FAM214A;TMEM241;GLI3;FCRLA;HHAT;FAM241A;ABL2;ST8SIA6;VAV3;CERS6;MRTFB;TBC1D9;MITF;GFRA1;PRLR;ESR1;INPP4B;KIF16B;BMPR1B;FSIP1*
LINC02239	25/100	0.012280094346006434	*GALNT13;HEPACAM;LUZP2;CTNND2;KLHL32;PCDH15;GRIK4;ADCY2;FMN2;CDH20;FUT9;NCAM1;KIF21B;JAM2;WASF3;GRIA4;RFTN2;NTRK2;DTNA;TCF12;FRMD5;APC;PPP2R2B;SMOC1;APBA2*
LINC00928	25/100	0.012280094346006434	*CRB1;GALNT13;PHLPP1;LUZP2;CTNND2;PCDH15;GRIK4;TRIM9;CDH20;NCAM1;KIF21B;GRIA4;RFTN2;GRID2;DSCAM;KCND2;CADM2;TMOD2;TCF12;LSAMP;LHFPL3;FCHSD2;SMOC1;DSCAML1;APBA2*
LINC01751	25/100	0.012280094346006434	*NRP1;GALNT14;ENPEP;ANKRD33B;PRKAA2;ARHGEF28;LRP2;SLC6A3;CNDP2;PKHD1;ARHGAP42;TTC21B;ST8SIA4;ENPP3;PDK1;CUBN;EGLN3;VCAM1;KSR1;INSR;ANXA4;COL23A1;MYO9A;PLIN2;ALPK2*
LINC02035	25/100	0.012280094346006434	*MAPKBP1;MACF1;MTMR3;CHD9;KMT2C;PCNX1;NIPBL;RNF111;RBM33;SPEN;MBNL1;DST;EXOC6B;RC3H1;DNAJC13;LNPEP;PHC3;RC3H2;HIPK3;PIAS1;KANSL1;RFX7;SLC49A4;ZNF236;TNRC6B*
LINC02339	25/100	0.012280094346006434	*GALNT13;PID1;SHC3;MEGF11;NRXN1;SLC35F1;GRIK2;RASSF2;SNTG1;DNER;TNR;LRRC4C;CACNG2;GRIA4;GRID2;CA10;DSCAM;CADM2;TMEM132B;LHFPL3;SEZ6L;EPN2;ADGRB3;DSCAML1;UNC79*
LINC01338	25/100	0.012280094346006434	*HEPACAM;LUZP2;CTNND2;KLHL32;GRIK4;ADCY2;FMN2;IQCJ-SCHIP1;CDH20;FUT9;NCAM1;WASF3;JAM2;RFTN2;NTRK2;DTNA;TMOD2;GABRG1;ETNPPL;TAFA5;APC;PPP2R2B;CPE;ASB3;APBA2*
RBMS3-AS2	25/100	0.012280094346006434	*NFAT5;ANKRD36;CHD9;KMT2C;RGPD6;ZBTB20;ZDHHC21;SLC9C1;LPP;ATXN1;ZNF407;ANKRD36C;DNAH11;MBD5;MON2;VPS13C;RC3H1;VPS13B;PLEKHA3;PHC3;ZNF717;WDPCP;BIRC6;KIAA0825;ANKRD36B*
PKP4-AS1	25/100	0.012280094346006434	*NFAT5;PATJ;STXBP4;KMT2C;RGPD6;RGPD5;RGPD8;SYNE2;RGPD4;SCAF8;ZNF407;SAMD12;RNF111;BPTF;MBD5;ARHGEF12;LRBA;VPS13B;PHC3;MYO9A;WDPCP;WDFY3;BIRC6;ZNF236;DOCK1*
TMEM72-AS1	25/100	0.012280094346006434	*CYFIP2;GALNT13;RTN1;CTNND2;NRXN1;KLHL32;GRIK4;ADCY2;FAM171A1;CDH20;NCAM1;SH3GL2;WASF3;GRIA4;BBS2;RFTN2;ARNT2;CADM2;NTRK3;TMOD2;DNM3;ADGRB3;PPP2R2B;DSCAML1;APBA2*
MYT1L-AS1	25/100	0.012280094346006434	*RTN1;KCNC1;MYT1L;FRMPD4;ATL1;RPH3A;UNC80;KIAA0513;ERC2;CACNG2;RGS7;RBFOX1;SYT1;SLC4A10;SYT16;GRIN2B;SYN2;GABRG2;SNAP91;CNKSR2;DLG2;SYNJ1;ADGRB3;SCN8A;SCN2A*
CADM3-AS1	25/100	0.012280094346006434	*GRIA1;RTN1;KCNC1;MYT1L;CTNND2;NRXN1;ATL1;SLC1A2;FMN2;KIAA0513;NCAM1;ERC2;SH3GL2;TMOD2;LSAMP;FAM219A;SYN2;SNAP91;CNKSR2;DNM3;ADGRB3;PPP2R2B;SCN2A;APBA2;ASTN1*
PACRG-AS3	25/100	0.012280094346006434	*SH3GL3;PDE1C;PPM1L;HEPACAM;KLHL32;OTUD7A;ZBTB20;NKAIN2;GRM3;DPYSL5;MVB12B;SLC16A7;THSD7A;RNF152;ANKS1B;RFTN2;UNC13C;STPG2;MOB1B;DNM3;TTLL7;PPP2R2B;ZNF536;DGKI;CNTNAP5*

NRG3-AS1	25/100	0.012280094346006434	*ACSS3;GALNT17;EPS8;ADAMTSL1;KIF21A;THSD7A;RNF152;PACRG;ANGPT1;TANGO2;KLHL3;PARVB;MYRIP;MOB1B;TBC1D1;BACE2;NRG3;TMEM116;RCAN2;SPIRE1;ERP27;CFTR;DGKI;LHX9;CNTNAP5*
SHANK2-AS1	25/100	0.012280094346006434	*PATJ;ANKRD36;KMT2C;RGPD6;ZBTB20;SLC9C1;MYSM1;ATXN1;KIAA1328;ZNF407;ANKRD26;MBD5;ANKRD30B;ANKRD30A;DNAH14;RC3H1;VPS13B;PHC3;ZNF717;ZNF678;BIRC6;ZNF236;KIAA0825;ANKRD36B;TNRC6B*
CNTN4-AS2	25/100	0.012280094346006434	*TSHZ2;RGPD6;ZBTB20;RGPD5;RGPD8;AKAP13;SCAF8;KIAA1328;ZNF407;MPDZ;ADAMTS9;ZNF462;MBD5;ANKRD30A;VPS13B;LRP1B;ZNF717;MMP16;NFIA;TTC3;FAT3;CNTN3;CNTN4;UTRN;ZNF236*
RAPGEF4-AS1	25/100	0.012280094346006434	*RTN1;KCNC1;MYT1L;FRMPD4;SLC1A2;GRM3;KIAA0513;GRM5;SV2B;ERC2;SH3GL2;RBFOX1;SLC4A10;SYT16;ATP2B2;GRIN2B;SYN2;SNAP91;CNKSR2;DLG2;SYNJ1;SCN8A;TAFA1;SCN2A;RAPGEF4*
TPRG1-AS2	25/100	0.012280094346006434	*PTPRT;MAST4;CCDC126;GREB1L;LDLRAD3;FAM214A;TMEM241;FAM241A;POTED;ST8SIA6;VAV3;KDM4B;SIAH2;COG2;CYBRD1;TBC1D9;GFRA1;PRLR;ESR1;DCDC1;NAT1;KIF16B;NEK10;BMPR1B;FSIP1*
GNG12-AS1	25/100	0.012280094346006434	*BNC2;ABCB5;LRMDA;SDCBP;SGCD;UBL3;GNG7;RGS20;ZNF106;HMCN1;DIP2C;EVI5;SOX5;MYEF2;PCDH7;SAMD4A;CABLES1;MITF;GNG12;STON1-GTF2A1L;IGSF11;ITPKB;NFIA;PDZRN3;DDR2*
NCKAP5-IT1	25/100	0.012280094346006434	*PATJ;ANKRD36;KMT2C;SLC9C1;MYSM1;ATXN1;ZNF407;PSD3;POTED;ANKRD26;MBD5;MON2;ANKRD30A;DNAH14;RC3H1;VPS13B;PLXDC2;LRP1B;TTC6;NEK10;ZNF678;WDPCP;BIRC6;KIAA0825;ANKRD36B*
DPYD-AS1	25/100	0.012280094346006434	*MACF1;MCTP1;MARCHF1;CHD9;SNX30;BACH1;RPS6KA3;AKAP13;PTAR1;PCNX1;FCHO2;IL6R;EVI5;LYN;ANKRD36C;MBNL1;CCDC18;MON2;VPS13C;PTPN13;HIPK3;PTPRE;MDFIC;DPYD;KIAA0825*
CCDC144NL-AS1	25/100	0.012280094346006434	*TSHZ2;CACNA1C;RPTOR;AKAP10;SCAF8;HERC2;MPRIP;ZNF407;SACS;MPDZ;ADAMTS9;ZNF287;SPECC1;ZNF462;RBFOX2;EYA1;COL25A1;TOP3A;TIAM2;NCOR1;MMP16;ULK2;FAT3;CNTN3;ADGRL2*
DIRC3-AS1	25/100	0.012280094346006434	*DNAH5;PRICKLE2;COL19A1;EHBP1;SRGAP2C;GNG2;SACS;FAM180A;SRGAP2;SRGAP2B;SLC15A5;VAT1L;SOX5;MAP4K4;NTNG1;TRIM43B;KIAA1549L;FRMD4A;CORO2B;ACER2;MOSMO;OR4F15;MMP16;ZNF536;CNIH3*
CPB2-AS1	25/100	0.012280094346006434	*CRB1;GALNT13;PID1;PHLPP1;NRXN1;KLHL32;GRIK4;GRIK2;TRIM9;RASSF2;CDH20;DNER;TNR;NCAM1;GRIA4;RFTN2;ARNT2;DSCAM;CADM2;LSAMP;LHFPL3;IGSF11;ADGRB3;PPP2R2B;ASTN1*
VLDLR-AS1	25/100	0.012280094346006434	*FHOD3;PRKN;TBC1D19;ACSS3;ARHGEF28;BCL2L13;SLC16A7;KIF21A;THSD7A;DIP2C;TEAD1;PACRG;TANGO2;MITF;KLHL3;ITFG1;MOB1B;AFG3L2;TBC1D1;RCAN2;SPIRE1;ALPK3;OSBPL1A;CNTNAP5;CPEB4*
MFF-DT	25/100	0.012280094346006434	*NLGN1;PPM1L;ZBTB20;MSANTD4;UBL3;SLC16A7;THSD7A;SOX6;BBS2;RFTN2;ARNT2;MBD5;NDFI*

			P1;SLC2A13;MAGI2;FBXL17;ZDHHC17;PJA2;MOB1B;PDP2;TBC1D5;VPS41;SPIRE1;WDFY3;CPEB4
CREB3L2-AS1	25/100	0.012280094346006434	NFAT5;ANKRD36;KMT2C;ZBTB20;ZDHHC21;SLC9C1;LPP;MYSM1;ATXN1;ANKRD36C;MBD5;MON2;VPS13C;DNAH14;RC3H1;VPS13B;PLEKHA3;PHC3;ZNF717;ZNF678;WDPCP;BIRC6;KIAA0825;ANKRD36B;TNRC6B
NRXN2-AS1	25/100	0.012280094346006434	GALNT13;CTNND2;NRXN1;GRIK4;FMN2;GRIK2;TRIM9;RASSF2;CDH20;DNER;NCAM1;FYN;LRRC4C;GRIA4;RFTN2;GRID2;DSCAM;CADM2;NTRK3;TMOD2;LSAMP;LHFPL3;ADGRB3;APBA2;ASTN1
FGF14-IT1	25/100	0.012280094346006434	DOCK4;TSHZ2;ZBTB20;OR9Q1;TTC28;KIAA1328;TMEM225;SNTG1;ZNF407;SACS;MPDZ;ADAMTS9;ZNF462;DNAH11;MBD5;MYO9A;PIAS2;MMP16;NFIB;WDPCP;WDFY3;FAT3;TCF4;CNTN3;KIAA0825
NNT-AS1	25/100	0.012280094346006434	GALNT13;PHLPP1;GRIK4;FMN2;TRIM9;DNER;NCAM1;SOX6;GRIA4;BBS2;RFTN2;ARNT2;CADM2;TTC33;TMOD2;MAGI2;LSAMP;FBXL17;PRKCA;APC;ADGRB3;PPP2R2B;SPIRE1;MAPRE2;ASTN1
C1QTNF7-AS1	25/100	0.012280094346006434	ABCB5;FMN1;BCL2L13;SDCBP;SGCD;UBL3;MXI1;LONP2;ABL2;RGS20;RTTN;ZNF106;DIP2C;MYEF2;MYO10;SETDB2;MYO5A;CABLES1;MITF;PARVB;NSG1;GNG12;INPP4B;SPIRE1;OSBPL1A
ANO3-AS1	25/100	0.012280094346006434	ACSS3;CUL5;PPM1L;GLYAT;HS6ST3;CDH7;AP5M1;SLC16A7;THSD7A;SLC17A1;RNF152;UNC13C;CUBN;STPG2;ABCA5;PDE4D;SLC2A13;WDR72;MOB1B;INPP4B;MSRA;PLCXD3;DGKI;CNTNAP5;CPEB4
NAV2-AS5	25/100	0.012280094346006434	MACF1;NFAT5;ANKRD36;CHD9;KMT2C;ZBTB20;RORA;ZDHHC21;SLC9C1;LPP;RPS6KA3;ATXN1;EVI5;ANKRD36C;DNAH11;MBD5;MON2;VPS13C;RC3H1;PLEKHA3;PHC3;FER;KIAA0825;ANKRD36B;TNRC6B
OPCML-IT2	25/100	0.012280094346006434	PSIP1;SLC35F1;ZDHHC21;RIMS1;IGSF21;SNAPC3;DNER;ST8SIA5;KIF21A;FYN;NCAM1;OPCML;GRID2;CA10;DSCAM;CADM2;TMEM132B;NTRK3;LSAMP;DNM3;MMP16;SPIRE1;DSCAML1;CNTNAP5;SNTB1
TSPAN9-IT1	25/100	0.012280094346006434	NFAT5;CUL5;ANKRD36;KMT2C;RGPD6;ZBTB20;KIAA1328;ZNF407;SLC16A7;RNF152;MBD5;MON2;STPG2;PDE4D;RC3H1;VPS13B;LNPEP;PHC3;MYO9A;MOB1B;ZNF717;KIAA0825;DGKI;ANKRD36B;TNRC6B
TMEM132D-AS2	25/100	0.012280094346006434	GABRB3;SLC24A2;MYT1L;STXBP1;CELF4;NALCN;RPH3A;UNC80;DPP6;SPOCK3;TBX20;PAK3;TMEM132D;PTPRN2;SYT1;PRKCE;GABRG2;SNAP91;CNKSR2;SGSM1;SYNJ1;SCN8A;CDH18;UNC79;HCN1
JAKMIP2-AS1	25/100	0.012280094346006434	GALNT13;CTNND2;NRXN1;GRIK4;FMN2;TRIM9;DPP6;CDH20;DNER;NCAM1;LRRC4C;GRIA4;RFTN2;GRID2;DTNA;DSCAM;CADM2;TMOD2;LSAMP;FAM219A;APC;ADGRB3;PPP2R2B;APBA2;ASTN1
LINC01600	24/100	0.02289331770290072	CUBN;NRP1;GALNT14;ENPEP;NLGN1;EGLN3;VCAM1;ANKRD33B;PRKAA2;MAML2;KSR1;COL23A1;MYO9A;SLC6A3;TTC28;MXI1;GRB10;ENPP3;ALPK2;OSBPL1A;RGL1;STK32B;ZNF521;ANKRD6
LINC02052	24/100	0.0228933177	DGKG;SPECC1;GALNT13;SLC24A3;DSCAM;MAP3K

		0290072	*7CL;SLC35F1;LHFPL3;GRIK2;SEL1L2;EPN2;TTLL7;TRIM9;RASSF2;LRRC7;HMCN2;TMEM108;ITGA8;TNR;NCAM1;ASB2;PLXNA4;ASTN1;GRIA4*
LINC00320	24/100	0.0228933177 0290072	*RFTN2;SH3GL3;RTN1;DTNA;HEPACAM;CADM2;CTNND2;NRXN1;TMOD2;KLHL32;OTUD7A;ADARB2;NKAIN2;GRM3;DNM3;RASSF2;ADGRB3;CDH20;PPP2R2B;MVB12B;NCAM1;SH3GL2;WASF3;ANKS1B*
LINC02774	24/100	0.0228933177 0290072	*RFTN2;GALNT13;SHC3;RTN1;DSCAM;MEGF11;TMEM132B;NRXN1;TMOD2;GRIK4;SLC35F1;LHFPL3;GRIK2;EPN2;TRIM9;RASSF2;ADGRB3;SNTG1;TNR;NCAM1;DSCAML1;APBA2;GRIA4;OPCML*
LINC01074	24/100	0.0228933177 0290072	*RFTN2;CRB1;NTRK2;GALNT13;DTNA;DSCAM;HEPACAM;CTNND2;KLHL32;LSAMP;GRIK4;ADCY2;FMN2;GABRG1;CDH20;PPP2R2B;SMOC1;DNER;PHACTR3;TNR;NCAM1;CTNNA2;JAM2;ASTN1*
LINC02607	24/100	0.0228933177 0290072	*RBFOX1;RTN1;SYT1;MYT1L;FRMPD4;ATL1;SLC4A10;SYT16;GRIN2B;SYN2;SNAP91;CNKSR2;KIAA0513;DLG2;SYNJ1;SV2B;SCN8A;NOS1AP;TAFA1;AMPH;SCN2A;ERC2;SH3GL2;RAPGEF4*
LINC00484	24/100	0.0228933177 0290072	*ZNF573;NFAT5;ZFHX3;MBD5;ANKRD36;KMT2C;MGA;RGPD6;ZBTB20;RC3H1;LNPEP;RGPD8;PHC3;PCNX1;KIAA1328;HERC1;ZNF717;ZNF407;WDPCP;BIRC6;UTRN;ATP9B;ANKRD36B;TNRC6B*
LINC01238	24/100	0.0228933177 0290072	*PTPRT;USP14;KDM4B;AUTS2;ANKRD30A;MRTFB;COG2;GREB1L;TBC1D9;GFRA1;DUX4;ESR1;FAM214A;DCDC1;NAT1;TTC6;PSD3;FAM241A;POTED;NEK10;ST8SIA6;ZNF385D;BMPR1B;POTEC*
LINC01323	24/100	0.0228933177 0290072	*RFTN2;CRB1;GRID2;GALNT13;DSCAM;CADM2;LUZP2;PCDH15;LSAMP;GRIK4;GRIK2;PHF21B;TRIM9;FRMD5;BRINP2;DLG2;SMOC1;PHACTR3;NCAM1;FYN;CTNNA3;FGF12;DSCAML1;GRIA4*
LINC00461	24/100	0.0228933177 0290072	*RFTN2;CRB1;GRID2;GALNT13;DSCAM;HEPACAM;CADM2;CTNND2;NRXN1;TMOD2;LSAMP;GRIK4;FMN2;TRIM9;RASSF2;APC;ADGRB3;CDH20;PPP2R2B;DNER;NCAM1;APBA2;ASTN1;GRIA4*
LINC02431	24/100	0.0228933177 0290072	*LRRC69;UNC13C;NFAT5;ACSS3;STPG2;CUL5;ABCA5;PPM1L;PDE4D;ZBTB20;ADAM32;CDH7;HS6ST3;MOB1B;INPP4B;AP5M1;KIAA1328;ZNF208;SLC16A7;ERP27;THSD7A;RNF152;DGKI;CNTNAP5*
LINC02224	24/100	0.0228933177 0290072	*PTPRT;USP14;CERS6;CLSTN2;CYBRD1;TBC1D9;GFRA1;LRP2;AFF3;ESR1;TMEM241;PBX1;RERG;MED13L;DACH1;FGF14;NAT1;KIF16B;FAM241A;MALRD1;BMPR1B;FSIP1;PRKACB;FGF10*
LINC01060	24/100	0.0228933177 0290072	*CUBN;NRP1;GALNT14;ENPEP;MGAM;EGLN3;VCAM1;PRKAA2;KSR1;INSR;COL23A1;LDB2;LRP2;MYO9A;SLC6A3;CNDP2;PDGFD;GIPC2;GRB10;PLIN2;ENPP3;ALPK2;STK32B;ARSB*
LINC02123	24/100	0.0228933177 0290072	*RFTN2;NTRK2;DTNA;HEPACAM;CADM2;LUZP2;CTNND2;TMOD2;KLHL32;SLC1A2;LSAMP;GRIK4;SLC6A11;ADCY2;FMN2;APC;CDH20;PPP2R2B;PHACTR3;CTNNA2;APBA2;JAM2;WASF3;ASTN1*
LINC02389	24/100	0.0228933177 0290072	*RBFOX1;RTN1;SYT1;KCNC1;MYT1L;FRMPD4;ATL1;SLC4A10;SYT16;GRIN2B;SYN2;GABRG2;SNAP91;CNKSR2;RIMS1;KIAA0513;DLG2;SV2B;SCN8A;TBC1D30;TAFA1;SCN2A;ERC2;SH3GL2*
LINC02281	24/100	0.0228933177 0290072	*RTN1;ARPP21;KCNC1;FRMPD4;MYT1L;NRXN1;TMOD2;SLC1A2;SLC4A10;OTUD7A;SYT16;HTR2C;SYN2;SNAP91;TMEM225;ADGRB3;PPP2R2B;TAFA1;YPEL1;SCN2A;ERC2;APBA2;TBATA;OPCML*

LINC02306	24/100	0.02289331770290072	*VAV3;KDM4B;CERS6;CLSTN2;LRBA;ITPR2;TBC1D9;LDLRAD3;LRP2;AFF3;PRLR;ESR1;DCDC1;RERG;MED13L;DACH1;FGF14;FAM241A;NEK10;MALRD1;BMPR1B;FSIP1;PRKACB;FGF10*
LINC00622	24/100	0.02289331770290072	*MINAR1;ARHGEF11;KIRREL1;MYEF2;IGSF3;MYO10;MOK;MYO5A;CABLES1;MITF;FMN1;PARVB;NSG1;SDCBP;SGCD;CHCHD6;ABL2;RGS20;PHACTR1;PRAME;ZNF106;HMCN1;SRGAP2;SNX8*
LINC02516	24/100	0.02289331770290072	*ANKRD36C;PATJ;ANKRD26;MBD5;MON2;ANKRD36;ANKRD30A;LRBA;VPS13C;DNAH14;GREB1L;RC3H1;VPS13B;ZDHHC21;SLC9C1;PHC3;MYSM1;ZNF717;TTC6;ZNF407;POTED;ZNF678;KIAA0825;ANKRD36B*
LINC02564	24/100	0.02289331770290072	*RFTN2;USP14;PHLPP1;LAMA1;LUZP2;CTNND2;ABCB5;PCDH15;ADCY2;FMN2;NLK;IGSF11;FRMD5;CDH20;ZNF280B;SMOC1;ADGRB1;MVB12B;NCAM1;ERC1;SOX6;CSMD2;MDGA2;APBA2*
LINC02078	24/100	0.02289331770290072	*SVIL;SLC24A3;BNC2;DENND2B;TPM1;OR9Q1;UNC5D;CACNA1C;KLHL13;SEL1L2;RIMS2;BMPER;TMEM225;CDC42EP3;KCNMA1;HMCN2;ITGA8;SPOP;TSPAN2;PGM5;PPP1R12B;ASB2;PDZRN3;FBXL7*
LINC01801	24/100	0.02289331770290072	*ZNF573;USP24;MACF1;CREBBP;ITGA4;BICRAL;LRBA;IREB2;DENND4C;CHD6;TBC1D9;VPS13B;AFF3;MYO9A;MED13L;SCAF8;ZNF718;ELF2;HERC1;PPIP5K2;TTC21B;ST8SIA6;RNF111;BPTF*
KIRREL3-AS3	24/100	0.02289331770290072	*KIRREL3;RBFOX1;SYT1;KCNC1;MYT1L;FRMPD4;CACNA2D3;SLC4A10;CELF4;GRIN2B;SYN2;GABRG2;SNAP91;CNKSR2;LRFN2;KIAA0513;DLG2;SV2B;SCN8A;TAFA1;SCN2A;ERC2;RGS7;HCN1*
LAMC1-AS1	24/100	0.02289331770290072	*NFAT5;ZFHX3;MBD5;ZNF160;TUT4;DNAH14;FNDC3B;RGPD6;RC3H1;RGPD5;RGPD8;RASAL2;PHC3;MYSM1;BTAF1;MSANTD2;AGO2;ZNF449;KHDC4;PKN2;HIVEP2;ZNF236;TEAD1;RBM33*
ATP2B2-IT2	24/100	0.02289331770290072	*UNC13C;NFAT5;ACSS3;STPG2;CUL5;ABCA5;PPM1L;PDE4D;ZBTB20;CDH7;HS6ST3;MOB1B;INPP4B;LRFN5;AP5M1;KIAA1328;ZNF208;SLC16A7;ERP27;THSD7A;RNF152;DGKI;ANKRD7;CNTNAP5*
PTPRD-AS1	24/100	0.02289331770290072	*RFTN2;GALNT13;DTNA;HEPACAM;CADM2;CTNND2;NRXN1;NTRK3;TMOD2;KLHL32;GRIK4;FAM219A;PTPRD;IQCJ-SCHIP1;TRIM9;RASSF2;ADGRB3;CDH20;PPP2R2B;DNER;NCAM1;WASF3;ASTN1;GRIA4*
TAB2-AS1	24/100	0.02289331770290072	*RFTN2;NTRK2;DTNA;HEPACAM;LUZP2;CTNND2;TMOD2;KLHL32;GRIK4;ADCY2;FMN2;GABRG1;ETNPPL;IQCJ-SCHIP1;NKAIN3;APC;CDH20;FUT9;PPP2R2B;MVB12B;NCAM1;SH3GL2;WASF3;JAM2*
PTPRD-AS2	24/100	0.02289331770290072	*RBFOX1;RTN1;SYT1;KCNC1;MYT1L;FRMPD4;ATL1;SLC1A2;SLC4A10;SYT16;GRIN2B;SYN2;GABRG2;SNAP91;CNKSR2;KIAA0513;GRM5;SV2B;SCN8A;TAFA1;SCN2A;ERC2;SH3GL2;RAPGEF4*
NAALADL2-AS2	24/100	0.02289331770290072	*ANKRD36C;ANKRD36;CHD9;VPS13C;DNAH14;ZBTB20;LNPEP;ZDHHC21;PLEKHA3;ANK3;PTPN13;SLC9C1;LPP;PHC3;TANC2;NAALADL2;RPS6KA3;PTAR1;FER;KCNQ3;ZNF678;MCTP2;KIAA0825;ANKRD36B*
THRB-AS1	24/100	0.02289331770290072	*PACRG;ACSS3;THRB;STPG2;PPM1L;TANGO2;GALNT17;EBF2;PRKAG2;KLHL3;MYRIP;MOB1B;ADAMTSL1;TBC1D1;AUH;TMEM116;RCAN2;SLC16A7;K*

			IF21A;ERP27;THSD7A;RNF152;CNTNAP5;SNTB1
NAALADL2-AS3	24/100	0.02289331770290072	*ANKRD36C;NFAT5;MBD5;ANKRD36;KMT2C;VPS13C;DNAH14;ZBTB20;RC3H1;ADAM32;ZDHHC21;PLEKHA3;SLC9C1;LPP;PHC3;MYSM1;ATXN1;ZNF717;ZNF678;CCSER1;BIRC6;KIAA0825;ANKRD36B;KDM7A*
TCF7L1-IT1	24/100	0.02289331770290072	*ANKRD36C;ANKRD26;MBD5;ANKRD36;KMT2C;DNAH14;RGPD6;ZBTB20;RC3H1;VPS13B;RGPD5;ADAM32;RGPD8;SLC9C1;MYSM1;PIAS2;KIAA1328;ZNF717;ZNF407;AGO2;WDPCP;ZNF678;BIRC6;ANKRD36B*
RPS6KA2-IT1	24/100	0.02289331770290072	*MACF1;MBD5;TRIO;WSB1;KMT2C;PDE4D;VPS13C;RGPD6;NEDD9;RGPD5;ZDHHC21;RGPD8;BTBD9;SNX30;PTPRE;AKAP13;DPY19L1;ATXN1;NUP210L;RPS6KA2;KCNQ3;ZNF678;EYS;XPR1*
UFL1-AS1	24/100	0.02289331770290072	*RFTN2;GRID2;GALNT13;DTNA;KCND2;HEPACAM;CADM2;LUZP2;CTNND2;NRXN1;KLHL32;GRIK4;ADCY2;FMN2;NPAS3;ADGRB3;CDH20;FUT9;PPP2R2B;DNER;NCAM1;APBA2;WASF3;GRIA4*
PCDH9-AS4	24/100	0.02289331770290072	*ANKRD36C;DOCK4;MBD5;PCDH9;ANKRD36;ANKRD30A;KMT2C;RGPD6;ZBTB20;RC3H1;VPS13B;RGPD5;ZDHHC21;RGPD8;PLEKHA3;LRP1B;DNM3;ATXN1;AGO2;WDPCP;ZNF678;BIRC6;DOCK1;ANKRD36B*
SLC16A12-AS1	24/100	0.02289331770290072	*CYFIP2;PRKN;CUBN;GALNT14;ENPEP;MGAM;PRKAA2;PAQR5;ARHGEF28;PLCL1;CRIM1;KCNJ15;GLYAT;LRP2;MYO9A;ARHGAP24;CNDP2;PKHD1;MSRA;SNX29;CLCN5;GIPC2;SLC16A9;SLC17A1*
KCNMA1-AS1	24/100	0.02289331770290072	*SVIL;RBFOX2;TPM1;PRICKLE2;NRXN3;ABCC9;CACNA1C;FOXN3;STON1-GTF2A1L;EML1;LPP;SLC8A1;FOXP2;FYCO1;INPP5A;SLMAP;KCNMA1;TSPAN2;PGM5;PPP1R12B;ASB2;PDZRN3;VCL;PRKG1*
CADM2-AS1	24/100	0.02289331770290072	*RFTN2;CRB1;GRID2;GALNT13;DSCAM;KCND2;CADM2;LUZP2;CTNND2;TCF12;PCDH15;LSAMP;GRIK4;MAPK8IP1;PHF21B;DNM3;MMP16;SNTG1;CDH20;DNER;FAT3;NCAM1;APBA2;GRIA4*
ARHGAP31-AS1	24/100	0.02289331770290072	*BBS2;RFTN2;ZNF462;ARNT2;NLGN1;HFM1;MAML2;TMEM178B;PPM1L;CTTNBP2;TCF12;RNF38;ZBTB20;PYGO1;PTPRD;IQCJ-SCHIP1;CCDC88A;ARHGAP31;TBC1D5;SPIRE1;SOX6;ASB3;WASF3;CREB5*
ARHGEF26-AS1	24/100	0.02289331770290072	*RFTN2;NTRK2;GALNT13;DTNA;HEPACAM;CADM2;LUZP2;CTNND2;NRXN1;TMOD2;KLHL32;LSAMP;GRIK4;ADCY2;FMN2;ADGRB3;CDH20;FUT9;PPP2R2B;CPE;NCAM1;WASF3;JAM2;ASTN1*
SBF2-AS1	24/100	0.02289331770290072	*PACRG;ACSS3;CUL5;PDE1C;ARHGEF28;FBXL17;PRKAG2;KLHL3;ELOVL7;SCAMP1;EPS8;MOB1B;TBC1D1;AP5M1;TMEM116;ARSJ;RCAN2;SLC16A7;SPIRE1;KIF21A;ERP27;THSD7A;RNF152;CNTNAP5*
PPM1K-DT	24/100	0.02289331770290072	*RFTN2;NTRK2;RTN1;DTNA;HEPACAM;LUZP2;CTNND2;TMOD2;KLHL32;LSAMP;GRIK4;ADCY2;FMN2;GABRG1;ETNPPL;CDH20;FUT9;PPP2R2B;LRIG1;CPE;NCAM1;SH3GL2;WASF3;JAM2*
CPEB2-DT	24/100	0.02289331770290072	*ZNF573;TBC1D19;ANKRD26;MBD5;MON2;ABCA5;ANKRD36;KMT2C;DNAH14;ZBTB20;VPS13B;LNPEP;PHC3;MYO9A;PIAS2;KIAA1328;ZNF717;ZNF407;ZNF449;WDFY3;ZNF236;KIAA0825;METTL15*

			;ANKRD36B
SEMA6A-AS1	24/100	0.02289331770290072	*OCA2;NLGN1;PHLPP1;MYEF2;MYO10;MICAL3;MYO5A;CABLES1;MITF;IGSF11;ITPKB;ZNF608;NRG3;ZNF280B;MXI1;ANKFY1;PHACTR1;SOX6;DIP2C;SRGAP2;JARID2;DISC1;MDGA2;SRGAP2B*
MAST4-IT1	24/100	0.02289331770290072	*DNAH11;MBD5;MON2;ANKRD36;ANKRD30A;KMT2C;VPS13C;DNAH14;MUC19;ZBTB20;RC3H1;VPS13B;LNPEP;ZDHHC21;SLC9C1;PHC3;FAM214A;LRP1B;ATXN1;ZNF717;TTC6;ZNF678;KIAA0825;ANKRD36B*
EGFLAM-AS3	24/100	0.02289331770290072	*ANKRD36C;NFAT5;ANKRD26;MBD5;ANKRD36;KMT2C;DNAH14;RGPD6;RC3H1;VPS13B;RGPD5;PLEKHA3;SLC9C1;LPP;ATXN1;ZNF717;ZNF407;APLF;WDPCP;CCSER1;BIRC6;KIAA0825;ANKRD36B;TNRC6B*
TMEM108-AS1	24/100	0.02289331770290072	*ANKRD36C;MBD5;ANKRD36;KMT2C;VPS13C;RGPD6;RC3H1;VPS13B;ZDHHC21;PLEKHA3;SLC9C1;LPP;PHC3;ATXN1;NUP210L;ZNF407;AGO2;KCNQ3;WDPCP;ZNF678;BIRC6;EVI5;KIAA0825;ANKRD36B*
SLC25A48-AS1	24/100	0.02289331770290072	*MED1;PATJ;ANKRD26;MBD5;MON2;ANKRD36;KMT2C;DNAH14;MRTFB;ZBTB20;RC3H1;VPS13B;LNPEP;ZDHHC21;SLC9C1;PHC3;ATXN1;ZNF717;ZNF407;ZNF678;BIRC6;CDK12;KIAA0825;ANKRD36B*
MAGI2-AS1	24/100	0.02289331770290072	*ANKRD36C;NFAT5;MBD5;MON2;ANKRD36;CHD9;KMT2C;VPS13C;RGPD6;ZBTB20;RC3H1;VPS13B;LNPEP;ZDHHC21;PLEKHA3;SLC9C1;LPP;PHC3;PTAR1;FER;ATXN1;ZNF717;KIAA0825;ANKRD36B*
RERG-IT1	24/100	0.02289331770290072	*VAV3;ARFGEF1;LYPLA1;CERS6;STAU2;ANKRD30A;LRBA;MRTFB;TBC1D9;GFRA1;AFF3;PRLR;ESR1;FAM214A;PBX1;RERG;INPP4B;TTC6;FAM241A;POTED;NEK10;APBB2;FSIP1;PCMTD1*
HIF1A-AS3	24/100	0.02289331770290072	*ANKRD36C;NFAT5;MBD5;MON2;ANKRD36;CHD9;KMT2C;VPS13C;FNDC3B;RC3H1;VPS13B;LNPEP;FAM126B;SLC9C1;PHC3;MYSM1;MYO9A;PTAR1;ATXN1;WDPCP;BIRC6;KIAA0825;ANKRD36B;PDK1*
LINC02693	23/100	0.041582051761736336	*ZNF287;SGTB;TMOD2;TOP3A;LSAMP;FAM219A;FMN2;NALCN;SNAP91;DNM3;CCDC88A;DPP6;MPRIP;MMP16;ADGRB3;SNTG1;SACS;ULK2;FAT3;NCAM1;APBA2;ASTN1;MAP4K4*
LINC02656	23/100	0.041582051761736336	*ANKRD36C;NFAT5;MBD5;MON2;ANKRD36;CHD9;ZNF160;KMT2C;VPS13C;ZBTB20;RC3H1;LNPEP;FAM126B;SLC9C1;PHC3;MYSM1;MYO9A;PTAR1;ATXN1;ZNF449;KIAA0825;ANKRD36B;RBM33*
LINC00571	23/100	0.041582051761736336	*TBC1D19;PACRG;ACSS3;STPG2;ARHGEF12;CUL5;FBXL17;KLHL3;ELOVL7;SCAMP1;EPS8;MOB1B;TBC1D1;AP5M1;AUH;TRIM2;SLC16A7;KIF21A;ERP27;THSD7A;RNF152;DGKI;CNTNAP5*
LINC01266	23/100	0.041582051761736336	*NFAT5;TBC1D19;ACSS3;STPG2;ARHGEF12;CUL5;PDE1A;FBXL17;ZBTB20;PTPRM;KLHL3;MYO9A;MOB1B;TBC1D1;FER;KIAA1328;SLC16A7;BBS9;THSD7A;CNTN4;UTRN;RNF152;DGKI*
LINC00457	23/100	0.041582051761736336	*RYR2;ZFHX3;CCDC186;MAGI3;KMT2C;LRBA;PBX3;RGPD6;ZBTB20;ARHGAP28;PHC3;FRMD3;POTEM;POTEH;NBEA;TTC6;NFIA;SNX25;PRR16;POTEG;UTRN;DGKI;XKR6*
LINC01671	23/100	0.0415820517	*CUBN;NRP1;GALNT14;ENPEP;EGLN3;VCAM1;PRKAA2;KSR1;ARHGEF28;INSR;ANXA4;COL23A1;CR*

		61736336	*IM1;ARHGAP24;SLC6A3;CNDP2;PKHD1;SNX29;CLCN5;PLIN2;ENPP3;ALPK2;TRABD2B*
LINC01320	23/100	0.041582051761736336	*CUBN;NRP1;GALNT14;ENPEP;EGLN3;VCAM1;ANKRD33B;PRKAA2;ARHGEF28;INSR;ANXA4;COL23A1;LRP2;MYO9A;SLC6A3;CNDP2;PKHD1;SNX29;CLCN5;PLIN2;ENPP3;ALPK2;TRABD2B*
LINC01690	23/100	0.041582051761736336	*RFTN2;CRB1;GRID2;GALNT13;PHLPP1;DSCAM;CADM2;LUZP2;CTNND2;TMOD2;TCF12;PCDH15;LSAMP;GRIK4;TRIM9;FCHSD2;ADGRB3;CDH20;NCAM1;FYN;DSCAML1;WASF3;GRIA4*
LINC00362	23/100	0.041582051761736336	*PATJ;LRBA;VPS13B;LDLRAD3;LNPEP;GFRA1;LDLRAD4;ESR1;FAM214A;PBX1;MED13L;TTC6;BMP2K;ZNF678;GAST;APBB2;BMPR1B;FSIP1;PRKACB;CPA6;FGF12;FBXL7;SGCG*
LINC00327	23/100	0.041582051761736336	*MYEF2;EYA1;MOK;MYO5A;MITF;FMN1;AKAP6;CORO2B;COL19A1;IGSF11;SDCBP;SGCD;GNG2;IFT81;SCFD2;GNG7;SACS;PRAME;HMCN1;SRGAP2;DISC1;CNIH3;SOX5*
LINC02047	23/100	0.041582051761736336	*RFTN2;NTRK2;GALNT13;PHLPP1;HEPACAM;CADM2;LUZP2;CTNND2;NRXN1;TMOD2;KLHL32;GRIK4;ADCY2;FMN2;NKAIN3;ADGRB3;CDH20;FUT9;PPP2R2B;NCAM1;KIF21B;WASF3;GRIA4*
LINC00698	23/100	0.041582051761736336	*PHLPP1;MYEF2;ST8SIA1;CADPS;PCDH15;KIAA1549L;LSAMP;MITF;FMN1;ADCY2;AKAP6;DOCK10;IGSF11;ITPKB;FRMD5;UBL3;NRG3;CHCHD6;RNF182;SPIRE1;PHACTR1;PRAME;CNIH3*
LINC02060	23/100	0.041582051761736336	*BBS2;RFTN2;DTNA;DSCAM;HEPACAM;CADM2;LUZP2;CTNND2;TMOD2;PCDH15;LSAMP;GRIK4;ADCY2;FMN2;IQCJ-SCHIP1;TRIM9;APC;CDH20;PPP2R2B;NCAM1;APBA2;JAM2;WASF3*
LINC02735	23/100	0.041582051761736336	*MINAR1;MYEF2;IGSF3;MYO10;ABCB5;MICAL3;MYO5A;MITF;FMN1;PARVB;NSG1;MED15;IGSF11;UBL3;SGCD;ABL2;PHACTR1;PRAME;HMCN1;DIP2C;SNX8;RXRG;SOX5*
LINC02330	23/100	0.041582051761736336	*MINAR1;MYEF2;STXBP1;MOK;MYO5A;MITF;FMN1;AKAP6;PARVB;LHFPL2;CORO2B;IGSF11;SDCBP;UBL3;SGCD;NRG3;SCFD2;CHCHD6;PRAME;HMCN1;SRGAP2;CNIH3;SOX5*
LINC02296	23/100	0.041582051761736336	*LINGO2;SLC24A3;PDE1C;BNC2;GALNT17;XYLT1;MTUS2;CACNA1C;SEL1L2;ANO4;PCP4;GRIN2A;TAFA4;MPRIP;HMCN2;SACS;SH3BP5;PGM5;SLIT2;ASB2;PDZRN3;FBXL7;JAZF1*
LINC01581	23/100	0.041582051761736336	*SVIL;BNC2;NEGR1;PCDH7;PRICKLE2;CACNA1C;STON1-GTF2A1L;EML1;LPP;FOXP2;SLMAP;KCNMA1;ABL1;ROR1;TSPAN2;PGM5;CTNNA3;PPP1R12B;ASB2;PDZRN3;VCL;PDZRN4;DDR2*
LINC02188	23/100	0.041582051761736336	*CUBN;NRP1;GALNT14;ENPEP;EGLN3;VCAM1;ANKRD33B;KSR1;ARHGEF28;INSR;ANXA4;COL23A1;CRIM1;PPP2R3A;SLC6A3;CNDP2;NFIB;GRB10;PLIN2;ENPP3;ALPK2;RSU1;TRABD2B*
LINC01785	23/100	0.041582051761736336	*RBFOX1;RTN1;SYT1;FRMPD4;MYT1L;SLC1A2;SLC4A10;DEUP1;SYN2;NOL4;CDH8;SNAP91;GRM3;CNKSR2;SYNPR;CTIF;AGBL4;DLG2;AMPH;GPC5;SCN2A;SLC12A8;RGS7*
COL18A1-AS1	23/100	0.041582051761736336	*CUBN;PATJ;TBC1D19;MBD5;PRKAA2;ANKRD36;PAQR5;STXBP4;ZBTB20;AXDND1;MYO9A;ARHGAP24;PKHD1;MSRA;SNX29;CLCN5;ZNF407;WDFY3;B*

			IRC6;SLC17A1;KIAA0825;RNF152;ANKRD36B
BASP1-AS1	23/100	0.041582051761736336	*RBFOX1;RTN1;KCNC1;SGTB;MYO10;MYT1L;STXBP1;NRXN1;TMOD2;SLC4A10;OTUD7A;SYT16;MYO5A;GRIN2B;SYN2;GABRG2;SNAP91;SYNJ1;ADGRB3;PHACTR1;SCN2A;CACNG2;SH3GL2*
HTR5A-AS1	23/100	0.041582051761736336	*RBFOX1;RTN1;SYT1;KCNC1;MYT1L;FRMPD4;SLC1A2;SLC4A10;SYT16;ATP2B2;GRIN2B;SYN2;GABRG2;SNAP91;GRM3;CNKSR2;KIAA0513;SV2B;SCN8A;TAFA1;SCN2A;ERC2;SH3GL2*
EGFR-AS1	23/100	0.041582051761736336	*ZNF462;DOCK4;NLGN1;EGLN3;KSR1;NEK6;TSHZ2;NETO2;EGFR;CCDC88A;MMP16;SNTG1;SACS;FAT1;DPF3;MAPK1;FAT3;TCF4;PLIN2;ENPP3;ADAMTS9;ADGRL2;CREB5*
WARS2-IT1	23/100	0.041582051761736336	*RFTN2;CRB1;ARNT2;NTRK2;DTNA;KCND2;HEPACAM;CTNND2;ZBTB20;ADCY2;FMN2;SIPA1L2;DCLK1;IQCJ-SCHIP1;CCDC88A;TRIM9;FRMD5;APC;NFIA;FEZ2;FYN;ASB3;JAM2*
TGFA-IT1	23/100	0.041582051761736336	*PRELID2;DNAH11;MBD5;MON2;ANKRD36;CHD9;VPS13C;RC3H1;RORA;LNPEP;FAM126B;MYO9A;SYNE2;SLC6A3;PKHD1;PTAR1;ATXN1;WDFY3;BIRC6;ALPK2;KIAA0825;ANKRD36B;PDK1*
TEX26-AS1	23/100	0.041582051761736336	*SH3GLB1;CEP112;TSHZ3;PTPRQ;EBF1;AFAP1;PRG4;GXYLT2;FBXL13;PLXDC2;ANTXR1;FSTL1;FER;ATXN1;FLRT2;MDFIC;FEZ2;ZFPM2;RGL1;EVI5;TEAD1;FBN1;DDR2*
KCNMA1-AS2	23/100	0.041582051761736336	*MBD5;MON2;ANKRD36;ANKRD30A;KMT2C;ZBTB20;RC3H1;VPS13B;PLEKHA3;LPP;PHC3;LRP1B;ATXN3;ATXN1;KIAA1328;ZNF717;ZNF407;POTEG;RHPN2;ZNF236;KIAA0825;ANKRD36B;TNRC6B*
ADGRF5-AS1	23/100	0.041582051761736336	*ANKRD36C;NFAT5;DNAH11;MON2;MBD5;ANKRD36;CHD9;VPS13C;ERBIN;ZBTB20;RC3H1;VPS13B;ZDHHC21;FAM126B;CDC42BPA;SLC9C1;PHC3;FAM126A;RPS6KA3;PTAR1;ATXN1;KIAA0825;ANKRD36B*
LMCD1-AS1	23/100	0.041582051761736336	*RFTN2;ARNT2;NTRK2;DTNA;PHLPP1;PCDH9;HEPACAM;CTNND2;NRXN1;MAGI2;TMOD2;KLHL32;LSAMP;GRIK4;ADCY2;FMN2;IQCJ-SCHIP1;APC;CDH20;PPP2R2B;NCAM1;ASB3;WASF3*
ASIC4-AS1	23/100	0.041582051761736336	*RFTN2;GALNT13;PID1;DSCAM;CADM2;MEGF11;TCF12;GRIK4;LHFPL3;GRIK2;SEZ6L;TRIM9;ADGRB3;CDH20;SNTG1;SMOC1;DNER;TNR;NCAM1;CACNG2;DSCAML1;UNC79;GRIA4*
SOX21-AS1	23/100	0.041582051761736336	*RFTN2;CRB1;NTRK2;GALNT13;PHLPP1;DSCAM;HEPACAM;CADM2;LUZP2;CTNND2;TCF12;LSAMP;GRIK4;FMN2;TRIM9;CDH20;FUT9;PPP2R2B;DNER;NCAM1;APBA2;ASTN1;GRIA4*
NLGN1-AS1	23/100	0.041582051761736336	*ZNF462;DNAH11;DOCK4;MBD5;MON2;ANKRD36;STXBP4;CHD9;TSHZ2;ZBTB20;VPS13B;FBXL13;MYO9A;PIAS2;PDE10A;ZNF407;WDPCP;WDFY3;FAT3;ALPK2;KIAA0825;ADAMTS9;ANKRD36B*
ASTN2-AS1	23/100	0.041582051761736336	*NFAT5;MBD5;MON2;STPG2;ANKRD36;KMT2C;PDE4D;ZBTB20;VPS13B;LNPEP;LPP;PHC3;MOB1B;POTEM;KIAA1328;POTEH;ZNF717;SLC16A7;POTEG;RNF152;DGKI;ANKRD36B;TNRC6B*
ATXN1-AS1	23/100	0.041582051761736336	*MAGI1;ACSS3;NDFIP1;ARHGEF12;TTC33;SDC2;BCKDHB;FBXL17;FNDC3A;MYRIP;LRRC2;PJA2;ITFG1;CDC14B;PARD3B;ATXN1;ZNRF3;ZMYND11;*

			PPARA;SCAPER;TEAD1;SNTB1;CPEB4
FGF12-AS1	23/100	0.0415820517 61736336	*UNC13C;NFAT5;ZNF462;MBD5;KMT2C;TSHZ2;RGPD6;ZBTB20;VPS13B;RGPD5;RGPD8;MOB1B;KIAA1328;ZNF717;ZNF407;SLC16A7;FAT3;CCSER1;BIRC6;FGF12;RNF152;DGKI;TNRC6B*
TRAF3IP2-AS1	23/100	0.0415820517 61736336	*RFTN2;PHLPP1;DSCAM;CADM2;CTNND2;TMOD2;TCF12;LSAMP;GRIK4;ZDHHC17;CCDC88A;TRIM9;RASSF2;FCHSD2;APC;ADGRB3;CDH20;FYN;NCAM1;APBA2;ASB3;ASTN1;GRIA4*
GRM3-AS1	23/100	0.0415820517 61736336	*SH3GL3;RBFOX1;RTN1;MYT1L;TMOD2;KLHL32;SLC1A2;SLC4A10;OTUD7A;SYT16;GRIN2B;SYN2;SNAP91;NKAIN2;GRM3;KIAA0513;DLG2;PPP2R2B;SCN2A;ERC2;SH3GL2;ANKS1B;RAPGEF4*
GPR158-AS1	23/100	0.0415820517 61736336	*GALNT13;CADM2;TMOD2;LSAMP;GRIK4;GRIK2;FMN2;NALCN;SNAP91;UNC80;SLC8A3;TRIM9;DPP6;ADGRB3;DNER;TNR;NCAM1;LRRC4C;CACNG2;GPR158;UNC79;ASTN1;GRIA4*
DSCAM-IT1	23/100	0.0415820517 61736336	*RFTN2;CRB1;GRID2;GALNT13;DSCAM;CADM2;LUZP2;TCF12;PCDH15;LSAMP;GRIK4;LHFPL3;GRIK2;CDH20;SMOC1;DNER;NCAM1;FYN;SOX6;CSMD2;DSCAML1;APBA2;GRIA4*
RBMS3-AS1	23/100	0.0415820517 61736336	*ANKRD36C;NFAT5;DNAH11;MBD5;MON2;ANKRD36;CHD9;KMT2C;VPS13C;ZBTB20;RC3H1;VPS13B;ZDHHC21;PLEKHA3;SLC9C1;PHC3;PTAR1;FER;ATXN1;ZNF407;BIRC6;KIAA0825;ANKRD36B*
BARX1-DT	23/100	0.0415820517 61736336	*SVIL;ZFHX3;NRXN3;PRICKLE2;ABCC9;CACNA1C;STON1-GTF2A1L;EML1;LPP;FOXP2;SMOC2;INPP5A;SLMAP;KCNMA1;PDE3A;TSPAN2;PGM5;CTNNA3;TACC2;PPP1R12B;ASB2;VCL;PDZRN4*
STARD13-AS	23/100	0.0415820517 61736336	*NFAT5;MBD5;MON2;ANKRD36;KMT2C;VPS13C;DNAH14;RGPD6;ZBTB20;RC3H1;VPS13B;PLEKHA3;SLC9C1;PHC3;MYSM1;ATXN1;ZNF717;ZNF407;ZNF678;BIRC6;KIAA0825;ANKRD36B;TNRC6B*
MKLN1-AS	23/100	0.0415820517 61736336	*BBS2;NLGN1;MYEF2;SLC2A13;ZBTB20;PRKCA;SLC4A4;ARHGAP24;CDC14B;PARD3B;IGSF11;HADHB;BCL2L13;IMMP2L;RANBP3L;CLCN5;UBL3;TBC1D5;VPS41;EXOC4;SPIRE1;WDFY3;SOX6*
EDRF1-AS1	23/100	0.0415820517 61736336	*USP24;SETD2;KMT2C;TUT4;MGA;RC3H1;VPS13B;RGPD8;PRDM10;MYSM1;ARID1B;RGPD4;ELF2;BTAF1;KANSL1;RFX7;HECTD4;BIRC6;ZNF236;ZNF169;ANKRD36B;BPTF;TNRC6B*
KLF7-IT1	23/100	0.0415820517 61736336	*ANKRD36C;NFAT5;ANKRD26;MBD5;ANKRD36;CHD9;KMT2C;VPS13C;ZBTB20;RC3H1;VPS13B;LNPEP;SLC9C1;PHC3;MYSM1;ATXN1;ZNF717;ZNF407;WDPCP;BIRC6;KIAA0825;ANKRD36B;TNRC6B*
SNCA-AS1	23/100	0.0415820517 61736336	*ARHGEF11;MYEF2;MYO10;SGTB;STXBP1;KIAA1549L;MOK;MYO5A;MITF;FMN1;NSG1;LYST;IGSF11;SDCBP;UBL3;CHCHD6;ZNF280B;AKT3;ABL2;PHACTR1;PRAME;ZNF106;MDGA2*
FAM13A-AS1	23/100	0.0415820517 61736336	*NRP1;GALNT14;ENPEP;EGLN3;ARHGEF12;PRKAA2;KSR1;ARHGEF28;INSR;COL23A1;CRIM1;LDB2;MYO9A;ARHGAP24;SLC6A3;CNDP2;EPS8;ARHGAP42;FCHO2;RAPGEF2;PLIN2;ENPP3;ALPK2*
NREP-AS1	23/100	0.0415820517 61736336	*UNC13C;NFAT5;STPG2;CUL5;PPM1L;ANKRD30A;PDE4D;ZBTB20;EBF2;GXYLT2;LRP1B;CDH7;MOB1B;INPP4B;KIAA1328;ZNF717;SLC16A7;THSD7A;KIAA0825;RNF152;DGKI;SGCG;CNTNAP5*
DLGAP2-AS1	23/100	0.0415820517	*TMEM132C;SHC3;DMRT1;PRKCB;CHD9;DCC;KIAA*

		61736336	*1549L;F13A1;EBF2;FOXN3;CDH8;HCRTR1;SRGAP2C;MOSMO;GRM7;SV2B;AKT3;KCNQ3;GAS2;ZNF385D;SRGAP2;B4GALT6;DLGAP2*
CD44-AS1	23/100	0.0415820517 61736336	*MAPKBP1;OCA2;IGSF3;MYO10;ABCB5;MYO5A;CABLES1;MITF;NSG1;LHFPL2;MCC;SDCBP;SGCD;MTHFD1L;KIF13A;ABL2;SNAI2;RGS20;RTTN;ZNF106;SNX8;CD44;KRT6A*
CCND2-AS1	23/100	0.0415820517 61736336	*BBS2;RFTN2;RIC3;GALNT13;DSCAM;CADM2;GRID1;CTNND2;NTRK3;GRIK4;FMN2;TRIM9;DPP6;ADGRB3;CDH20;DNER;IL1RAPL1;NCAM1;FYN;LRRC4C;DSCAML1;ASTN1;GRIA4*
CACNA1C-AS4	23/100	0.0415820517 61736336	*ANKRD36C;NFAT5;MBD5;ANKRD36;KMT2C;RGPD6;ZBTB20;RC3H1;VPS13B;RGPD5;PLEKHA3;SLC9C1;LPP;PHC3;MYSM1;ATXN1;ZNF717;ZNF407;WDPCP;BIRC6;KIAA0825;ANKRD36B;TNRC6B*
SEC23A-AS1	23/100	0.0415820517 61736336	*SVIL;TANGO6;RBFOX2;TSHZ3;AFAP1;ABCC9;CACNA1C;FOXN3;GNG12;EML1;STON1-GTF2A1L;FYCO1;SLMAP;NEDD4;EOGT;KCNMA1;PPP1R12B;ASB2;TEAD1;VCL;PRKG1;MBTPS2;DDR2*
KCNIP4-IT1	23/100	0.0415820517 61736336	*ANKRD26;MBD5;MON2;ANKRD36;ANKRD30A;KMT2C;MUC19;DNAH14;DNAH6;GREB1L;ZBTB20;RFX3;RC3H1;EFCAB6;SLC9C1;PHC3;DCDC1;TMEM232;ERBB4;NEK10;WDPCP;ZNF678;KIAA0825*
PTENP1-AS	23/100	0.0415820517 61736336	*PTPRN2;CADM2;ATL1;ELAVL4;LSAMP;NALCN;TXNDC16;MAPK8IP1;PHF21B;UNC80;SLC8A3;IGSF21;DPP6;ADGRB3;DNER;CNTN1;CPE;NCAM1;ASTN2;GPR158;UNC79;ASTN1;GARNL3*
NPTN-IT1	23/100	0.0415820517 61736336	*NFAT5;MBD5;ANKRD36;CHD9;KMT2C;VPS13C;MGA;RC3H1;VPS13B;FAM126B;PHC3;MYSM1;ARID1B;PIAS1;ATXN3;KANSL1;RFX7;CCDC192;BIRC6;ZNF236;ANKRD36B;BPTF;TNRC6B*

Table S6. The intersections among rDNA-contacting genes associated with different lincRNAs in K562 before (K562) and after induced differentiation (K562-diff.) Related to the Venn diagram presented in Figure 2A.

Names	total	elements
K562-K562-diff.	902	*FSTL1 TBC1D19 HPSE2 SLMAP FAM219A SAMD4A ERBB4 KCNMA1 PDE6A APBB2 LPP KLHL13 ANKRD36B RNF38 FYB2 CUBN MACF1 SLC17A1 FAM214A PAK1 PCMTD2 CTNND2 DNAH14 FRMD3 SLC6A11 SCAF8 COL22A1 ATP9B TRIO MITF ZNF385D TC2N RTN1 SETD2 KIRREL1 SLC15A5 AMBRA1 CHD9 SYNE2 EPN2 NETO2 PDE1C DOP1B ZNF208 TTC37 NUP210L TSPAN3 IGSF3 ALPK3 MYO3B EVC MED13L NRP1 TEAD1 NFIA FRY CDH13 ZNF718 MDGA2 RC3H1 RPS6KA5 POTED LHFPL2 EPS8 TANGO2 TAOK3 HERC2 ZSCAN30 LRFN5 ZNF66 OR4C46 ABL2 DIP2C PRKCB SLC23A2 ARHGEF7 UTRN PHACTR3 GPC5 ZNF704 MAML2 ARHGEF12 SAMD12 SLC8A1 LYPLA1 MAN2A2 DACH1 EML1 CMIP LNPEP PRR16 CDK12 SLC2A13 ANKRD6 RERG HTR2C ZNF397 KCNC1 DUX4 GALNT13 DYSF STAU2 NEK4 CACNA1E SIAH3 TMEM63C FYCO1 SNTG1 LPGAT1 CNTN4 CTIF TBC1D5 USP14 EXOC6B ST8SIA4 AFG3L2 CNKSR3 FRMD5 LDLRAD4 SEMA3A IQCM TRIM2 PLIN2 SLC8A3 DNAH6 RFX3 MGAT5 RBFOX1 STARD13 PCDH7 MMP16 SLC40A1 PHC3 LRRC4C CADM2 ALCAM MALRD1 USP24 CCSER1 ERLIN2 SLCO3A1 ATP11C PDZRN3 LIMD1 ADAM32 PIK3C3 MYEF2 DCLK1 DNAH11 SLC9C1 ADAMTS9 CDC42BPA ANKRD28 MAGI3 NIPBL CEP120 POTEG CDC42EP3 CHD6 HMCN1 FGD4 FAM135B FSIP1 GRIK2 SNX25 IGSF11 RGL1 CCDC186*

		GSAP MBD5 MYO5C KIF16B NKAIN3 SDC2 NCAM1 SLC25A21 CLSTN2 TCF12 MARCHF6 SIPA1L2 CCNG2 RCAN2 KDM4B ADGRL2 GNG12 RGPD5 ODR4 TANC1 CORO2B PAPPA EVI5 NEBL ITGA8 SYCP1 VPS41 GRIK4 ZBTB20 MYO10 ZNF407 PJA2 ASB3 TCF4 TAFA4 NAALADL2 GABRG1 TSHZ2 TOM1L2 IREB2 PBX1 KIAA1549L FAM126B FAM171A1 ATP10A HECTD4 ELOVL7 PHACTR1 BICRAL TTC21B NEK6 ABCD2 SLC39A12 MOB1B COG2 ARHGAP28 DISC1 CLTCL1 SV2B HECW2 RIC3 FMN1 RALGPS1 ARHGAP42 PEAK1 EYA1 PHF21B KSR1 RORB PIEZO2 SLC35F1 KIAA1217 GPR158 ZNF236 GABRB1 SVIL ANKS1B TMEM241 NTM APBA2 TTC3 ASAP1 SGSM1 TTLL7 LYST FRMPD4 SLC16A9 COL23A1 LRRC2 EDAR PLEKHA3 ST8SIA1 ATP2B2 ANKRD26 FRMD6 RGS20 UNC79 CNDP2 FYN SH3KBP1 AGO2 NALCN CREBBP SLC1A2 ZBTB16 PRMT8 CADPS CSMD3 DLGAP1 FAM193A KIF21A LRBA B4GALT6 SLIT3 RGS7 FHIT SGTB GRIN2B ANTXR1 SHISAL1 SRGAP2 STK32A LRP1B ARSB GRIA1 OTUD7A TRABD2B TAFA5 NEGR1 SPRED1 AP5M1 MYO5A SPEN PLD5 NAV2 RABGAP1 FGF12 DGKB CACNG2 TSPAN33 PPP2R2C GARNL3 KANSL1 BTBD9 SPATA48 NPIPA1 TPM1 COL19A1 DPH6 EBF1 CRIM1 LRFN2 NOTCH2 FLNB MADD TOX CLDN10 PCDH15 ESR1 GABRG3 KIAA0232 FKBP5 NFAT5 SRGAP2C AQR ABCB5 TMOD2 PLCXD3 SLC44A5 FAM107B LUZP2 ZSWIM6 MEGF11 PDE4D TJP1 ARHGAP26 LRIG1 ERC2 PTPRT PRKACB GNG2 TRHDE PTPN4 RIMS1 GXYLT2 HIPK3 TRIM9 WSB1 TMEM225 FCHSD2 SGMS1 PARVB LAMA1 JCAD GLI3 GOLGA8S DOCK4 MAST4 GAST ITPR2 BRINP1 WDR26 NRXN1 FRMD4A SUPT16H NTRK3 BCL2L13 JAZF1 SGCG FBN1 WWOX NELL2 WASF3 LONP2 ARID1B MTUS1 HYDIN TRAPPC10 TSPAN2 CPQ WDPCP MAGI1 PRRC1 AUH MOSMO RAPGEF4 EHBP1 CNKSR2 NEK10 ZNF74 ANKRD31 PIAS1 SNX29 CDH20 ST6GALNAC3 ZNF521 PBX3 PTAR1 PRICKLE2 PCNX1 GALNT10 DPP6 LSAMP PRELID2 HMGA2 HHAT CTTNBP2 KLHL32 FHOD3 GRID1 GREB1L PARP8 ZNF287 CREB5 TNRC6B RERE CACNA2D3 SEL1L ZNF608 SLC4A10 PHLPP1 SYN2 ATL1 MYO1E SLC7A2 ZDHHC17 PKN2 ITGA4 ABCC9 CPA6 HFM1 GSG1L ACSS3 ROCK1 HERC1 CFAP70 DCDC1 DOCK1 CNTN3 CACNA1C MGAM GLIS1 ARHGAP31 UBR1 RXRG ETNPPL PRKAA2 ARHGEF11 AMPH CCSER2 RGPD4 TMEM178B SORCS1 COL4A2 DNM3 PACRG SYT1 BBS2 GRB10 ARHGAP32 SLC27A6 ASXL3 PRKN DPF3 MUC19 FOXP2 TRPM7 SCAPER EDIL3 SIAH2 LATS2 EXOC4 DOCK9 DLG2 NOL4 PPP1R12B SACS SCFD2 RORA PTPRD CDC14B GIPC2 CDYL2 LRRTM4 TTR BBS9 PTPRN2 RANBP3L TLN2 TG HDAC4 ADGRB3 ADAM29 INPP4B DGKG ZNF717 MYO9A MYO3A FOXN3 NTRK2 KIAA0513 PLCB1 HIVEP2 MAPK1 LAMB1 SDCBP ATP9A BPTF TASOR2 WDFY3 GRIK1 CUL5 SOX5 PRKG1 DSCAM BIRC6 DGKI PLXDC2 UBL3 NFIB DIP2B TUT4 NLK THSD7A SPOP NBEA EGFR PPFIA2 POTEC STXBP6 PTPRQ MGA MXI1 AFAP1 NCAM2 CLCN5 BCR TTC28 MAGI2 SLC49A4 NELL1 AKAP6 LARGE1 ANKRD30B ANKRD36C TRAPPC8 MBNL1 RTTN STX12 CSMD2 FANCM ARHGEF28 PLCL1 MELTF FUT9 GALNTL6 MYT1L TMPRSS3 SRGAP2B MOK PXDNL ANKFY1 TBC1D1 SLC37A2 IQCJ-SCHIP1 SORCS3 PDP2 SLC12A1 LRRC49 SCN2A ERP27 RNF152 POTEB3 CNTN1 ZNRF3 POU6F2 GNAQ ZNF449 AGL DOCK10 TENM3 OPCML ITPKB GRM5 MED1 MTMR7 GNPTAB ENPEP EPHA6 GLIS3 FNDC3A CA10 SPIRE1 PHACTR2 ANKRD36 RPRD1A ARAP2 AIMP1 LDB2 NECAB1 COL14A1 WDR72 SNX30 NRG3 CPEB4 ATP8A1 PID1 NLGN1 ABL1 PTPRG NCOR1 JARID2 PIGN PRDM10 PAQR5 OR9Q1 EBF2 JAM2 MICAL3 GRID2 ELMO1 FAM241A LRRC7 LRP2 RANBP17 ERBIN PRSS23 SEMA6D PCMTD1 PPM1L CABIN1 ZNF573 MBTPS2 KHDC4 SMOC1 FER PAK5 PDZRN4 SETDB2 MON2 TMEM116 RYR2 SCAMP1 ELF2 IL17RA LDLRAD3 FBXL17 MCC GLYAT SUSD4 GALNT14 PRLR ZNF160 ZNF280B HADHB NFATC2 DST FAT3 GRIK3 CACNB2 STPG2 STXBP4 PDXDC1 NTNG1 MED15 MTPN EFCAB6 VSTM2A GNG7 MTMR2 KCNH5 FBXL7 LRP12 MAPRE2 LRRC8B FGF10 ZNF169 KIF13A FAM189A2 NOS1AP SOX6 CADM1 POTEM SGCD RC3H2 TCERG1L PDE4DIP MSRA SYNPR RIC8B PCDH9 MPDZ YLPM1 TRPM3 ATF6 EYS SP3 ITFG1 IPO11 STK32B VCL ZNF106 SEZ6L ZDHHC21 XKR6 POTEB2 CNTNAP5 VCAM1 POTEH RAPGEF2 RGS12 CDH7 IFT81 SHANK2 ZMYND11 RAPGEF5 INSR SRGAP3 PDK1 AKAP13 UNC80 WDR41 ILDR2 IMMP2L MIPOL1 PPIP5K2 SYNJ1 CHCHD6 KLF15 TTC6 PPP2R2B DENND4C UNC5D ATXN1 KDM1B GABRG2 ARFGEF1 FAT1 PATJ ITGA9 ENPP1 ULK2 KIAA1328 UNC13C TANGO6 ZNF462 NRXN3 RIMS2 RHPN2

		ANKRD30A PPM1F KCNQ3 ANKRD33B SCN8A ZFHX3 DENND2B SNX8 HS6ST3 EGLN3 CHSY3 PGM5 FNDC3B ASTN1 DLGAP2 GFRA1 ATRNL1 SPHKAP GPR156 BMPR1B NSG1 RGPD8 MAPKBP1 CPE KCND2 FCRLA SEMA5A CABLES1 ATP8A2 GOLGA8T SLC24A3 KITLG PYGO1 SV2C LHFPL3 MPRIP ARHGAP24 FREM1 GRIA4 GSE1 MAP4K4 EFR3A CERS6 CNIH3 DOCK3 KIF21B CTNNA2 PRAME ANKRD18A APC APLF GPC6 PARD3B DNER ABCA5 MARCHF1 RFX7 ADGRV1 RELN MACROD2 AKAP11 VPS13C HS3ST4 BNC2 ANXA4 TTC33 EOGT KCNJ15 CEP112 NAT1 POTEB CYBRD1 AUTS2 TBC1D9 ROR1 PPP2R3A FAM81A MTMR3 STON1-GTF2A1L BMP2K SNAI2 CYFIP2 SLC4A4 KAZN ADAMTSL1 CCDC126 SYT16 NDFIP1 TNR MSANTD4 PTPN13 DTNA SNAP91 OCA2 CELF4 AKT3 ST8SIA6 CRB1 VAV3 IL1R1 BCAS3 MSANTD2 FCHO2 RFTN2 NPAS3 PRKCA HEPACAM KMT2C INPP5A USP8 USH1C MRTFB DNAH5 ZNF678 CNTN6 KCNS3 ENPP3 FMN2 ERC1 RGPD6 APP CHST3 PDLIM5 RPS6KA3 XYLT1 KIAA0825 BTAF1 SMAD5 SLC6A3 DNAJC13 NYAP2 PSD3 VPS13B ALPK2 ABCA13 ASB2 EYA2 MAPK8IP1 ADGRB1 CCDC88A GHRH ARNT2 RAP1GDS1 AFF3 SPOCK1
K562	374	*MAP4 PKNOX2 ZHX3 TSPAN11 HIVEP1 ATRX OR1L6 NBN PRTG ADAMTS18 SEC24D MPPED2 KMT2E EPB41L4A PELI2 MYO9B PWWP3A ITIH5 PACSIN2 SEC23B ZEB1 FAT4 PARN SLC15A2 TRIM23 WSCD1 FBXW8 ZFYVE1 DIP2A SFPQ MAB21L3 HOOK3 GPR137B ROCK2 ACACA C1QL3 POU1F1 TSPAN13 CCDC141 YTHDF3 SPATA17 SYT10 ZBTB38 PAFAH1B1 HECTD2 GON4L GREB1 ZNF648 USP49 ARFGEF3 FAM167B BCL2 KL MYO5B PPP1R9A RAP1GAP SPOPL SLC13A4 ZZEF1 FRA10AC1 ZFAND4 APMAP SHOC1 INO80D KDM5A NRIP1 ZNF382 GOLGA6D NDUFAF6 ITGA1 ITSN2 GDAP1L1 HIRA UFL1 EPHA4 TTLL11 DIDO1 COL4A3 ADAMTS2 PRKAA1 RASGEF1C FRYL PBLD ABCC4 MYT1 ASH1L SORBS2 PIK3R3 MEGF10 OPA3 ZC3HAV1 TMEM25 RAB38 ANKRD20A1 ZNF891 VMP1 SLC25A18 RALGAPA2 VSTM4 ADAMTS19 RAB27B CTSB MORC3 NDRG2 NSD1 NEDD4L PLS1 HDGFL3 ANK2 ABCC12 DSTYK LPCAT2 XRCC4 BZW1 FBXL20 MAP7 POTEJ ZNF518A PHC2 DCAF1 NSG2 CD2AP RGS8 KALRN ZNF124 KIAA0319L BMP7 KIAA0753 ANKRD17 LINGO1 TNKS SMPDL3A OR2T2 IMPA2 SCAI ZFP90 TULP4 S100B LTN1 ARHGAP12 KLF12 KCNN3 TINAG DIRAS2 FLI1 ANAPC1 KCNAB1 KLHL4 MTMR10 CDHR3 ITGB8 PRUNE2 POR CDH11 SETBP1 AIF1L CDS2 ZNF780B RBPMS2 LRRC9 ATP6V1E1 SLC37A1 PNPLA8 DTWD2 LMX1A RSPH3 KHDRBS2 VPS13D ZNF292 SPIN1 SNTB2 KPNA1 FUT8 ADCYAP1R1 LAMC1 ZNF611 SRP9 EPHB1 MYOCD TRIM58 GOLGA8J ZMYM1 UBE2QL1 UBAP1L PLPPR5 AVL9 FER1L6 ZNF33B PTCD2 HLCS STK10 GAPVD1 INTS7 SOGA1 UHRF1BP1L FLT1 ADSS2 RB1CC1 IKBIP NPHP4 FOXJ3 PDE2A CBLIF IL17RD ATP6V1B2 KCNJ1 SLC5A12 NCS1 TRPC5 ENTHD1 AMFR USP31 PLCB4 LOXL2 LCLAT1 FTO SLC6A1 CDH9 EPC2 GOLGA6B ADAM28 CYP4Z1 FGF9 ACSM2A TRAPPC11 NIN SLC1A1 HPCAL1 RAB3GAP2 LIMCH1 CMPK1 BAZ2B EML6 CRACD BAZ2A MEOX2 DDX6 CTSE TRPM1 CACNG3 PLEKHB2 IPCEF1 COPS8 AKAP9 PRKD1 PLCZ1 RBM47 SHROOM3 FBXO41 MSI2 ETS1 SYNE1 RALGPS2 SNRK HRH1 RABEP1 ST6GAL2 TOGARAM1 PTCHD4 SLX4IP PKHD1L1 ALKAL2 PLPPR1 MAP2 CEP192 TGFA ZFYVE26 GOLGA8F DHX29 PTPRA NUMB TNRC6C CELF2 CD70 TM9SF2 DEFB108B WSCD2 ZNF606 ESYT2 ZNF804B CAPN5 MLLT10 CSNK1G1 GOLGA6C KLHL29 ABCA4 SMTN PLEKHA2 SCG3 ZMYM4 DIPK1A DEPTOR VCAN ATAT1 RBMS3 TM9SF3 DDHD1 ICA1 SRFBP1 MATN2 RGS6 CRACR2A ZNF615 ATP6V0D2 PHF20L1 ZNF431 IARS2 S100PBP CELSR2 CFAP97 PTPRJ TET1 CATSPER2 RALGAPA1 LMX1B RRAGD SHLD2 ZMAT4 MARCHF8 CLIP1 SHC4 ELP2 ZNF613 UST TLL1 CALD1 PRDM15 UBE3A TYW1 HSF5 AGAP1 ADAM22 ZNF112 HTR2A TPH2 NCOA7 GALNT1 CHN1 GATAD2B TRPS1 INO80 SLC14A1 MFSD9 RANBP2 STRN CRISPLD2 EIPR1 CHCHD2 CLDN18 ADCY9 SLC25A48 GEMIN5 ANO6 KTN1 ERCC6L2 RUFY2 OR4K2 SUSD1 HIPK1 KCNE4 CLIC6 ACSM2B HSPA12A TTC7B PEPD BRWD1 AGO3 CNOT6L MEF2C TRAPPC6B ZNHIT6 ACTR3C MYB SLC39A6*
K562-diff.	158	*ST8SIA5 CD44 RPTOR TACC2 DMRT1 MDFIC HCN1 LRMDA TOP3A PTPRE SLC12A8 ARPP21 AGBL4 TRIM43B DSCAML1 CECR2 SLC16A7 NEDD4 RBM33 VAT1L SH3GLB1 THRB DPYD BACE2 LRRC69 IL6R PKHD1 CCDC18 CDH8 METTL15 RSU1 MAP3K7CL RNF182 ZFPM2 TMEM132C CDH18 AKAP10 DEUP1*

		SPOCK3 GALNT17 PDGFD HCRTR1 SH3GL3 ASTN2 BRINP3 TANC2 DPYSL5 PPFIBP1 ATXN3 PAK3 PSIP1 AXDND1 SEL1L2 GABRA2 SPECC1 YPEL1 PDE3A ZNF536 MORC1 SLC24A2 COL25A1 GRIN2A PCNX2 PRKAG2 MCTP2 SHC3 CCDC192 IL1RAPL1 TXNDC16 CTNNA3 KRT6A BMPER PDE1A FGF14 ANGPT1 RPH3A RPS6KA2 MVB12B PLXNA4 IGSF21 TBATA SMOC2 XPR1 PRKCE MTUS2 GRM3 FAM126A OR4F15 PPARA F13A1 ANO4 PIAS2 TAFA1 PRG4 KDM7A LYN NUBPL ELAVL4 MYSM1 TBC1D30 SH3BP5 TIAM2 TSHZ3 LINGO2 RASSF2 OSBPL1A ZNF367 CBWD3 SLC24A4 MTHFD1L NTF3 RNF111 CFTR TBX20 FLRT2 GAS2 PDE10A RBFOX2 BRINP2 ACER2 TMEM108 ZSWIM5 GABRB3 GRM7 NKAIN2 BACH1 RASAL2 PTPRM NEDD9 KIRREL3 MYRIP PCP4 STXBP1 ADCY2 ANK3 TMEM232 TMEM132B SNTB1 SH3GL2 DDR2 DCC TMEM132D HMCN2 FEZ2 SLIT2 ANKRD7 SNAPC3 MINAR1 FBXO32 KLHL3 FAM180A LHX9 FBXL13 ADARB2 ARSJ DPY19L1 BCKDHB MCTP1

Table S7. GO associations with biological processes associated with 902 overlapping rDNA-contacting genes that are associated with lincRNAs in both K562 and K562-diff. cells. The search was performed using g:Profiler (https://biit.cs.ut.ee/gprofiler/gost). Related to Figure 2B.

GO.ID	Description	padj	Genes
GO:0048731	system development	2.8586278276344312e-36	*ERBB4,PDE6A,RNF38,MACF1,PAK1,CTNND2,TRIO,MITF,RTN1,SETD2,AMBRA1,SYNE2,EPN2,IGSF3,ALPK3,EVC,NRP1,NFIA,FRY,CDH13,MDGA2,RC3H1,RPS6KA5,TAOK3,LRFN5,ABL2,PRKCB,SLC23A2,ARHGEF7,SLC8A1,EML1,KCNC1,STAU2,CNTN4,ST8SIA4,AFG3L2,SEMA3A,SLC8A3,RFX3,RBFOX1,STARD13,MMP16,SLC40A1,LRRC4C,CADM2,ALCAM,ADAM32,MYEF2,DCLK1,DNAH11,ADAMTS9,NIPBL,CEP120,MBD5,SDC2,NCAM1,CLSTN2,TCF12,KDM4B,ADGRL2,NEBL,ITGA8,TCF4,PBX1,PHACTR1,TTC21B,ABCD2,SLC39A12,DISC1,HECW2,FMN1,EYA1,RORB,KIAA1217,GABRB1,NTM,APBA2,ASAP1,TTLL7,EDAR,ATP2B2,FYN,AGO2,SLC1A2,ZBTB16,CSMD3,B4GALT6,SLIT3,GRIN2B,ANTXR1,SRGAP2,ARSB,GRIA1,TAFA5,NEGR1,SPRED1,SPEN,NAV2,FGF12,TPM1,COL19A1,CRIM1,NOTCH2,TOX,PCDH15,ESR1,SRGAP2C,ABCB5,TMOD2,ZSWIM6,MEGF11,TJP1,ARHGAP26,LRIG1,PRKACB,RIMS1,LAMA1,JCAD,GLI3,BRINP1,NRXN1,NTRK3,SGCG,FBN1,WWOX,WASF3,ARID1B,HYDIN,TSPAN2,WDPCP,MOSMO,ZNF521,PBX3,LSAMP,HMGA2,FHOD3,GREB1L,RERE,SLC4A10,PHLPP1,ATL1,MYO1E,ZDHHC17,ITGA4,ROCK1,HERC1,CACNA1C,COL4A2,DNM3,SYT1,BBS2,ARHGAP32,PRKN,DPF3,FOXP2,SCAPER,RORA,PTPRD,RANBP3L,TG,HDAC4,ADGRB3,ADAM29,DGKG,MYO9A,FOXN3,NTRK2,PLCB1,MAPK1,LAMB1,SDCBP,ATP9A,BPTF,GRIK1,SOX5,PRKG1,DSCAM,NFIB,DIP2B,THSD7A,EGFR,PPFIA2,PTPRQ,NCAM2,BCR,MAGI2,NELL1,AKAP6,LARGE1,MBNL1,ARHGEF28,FUT9,MYT1L,SRGAP2B,SCN2A,CNTN1,POU6F2,DOCK10,TENM3,OPCML,GRM5,MED1,ENPEP,EPHA6,FNDC3A,CA10,AIMP1,LDB2,NRG3,NLGN1,ABL1,PTPRG,JARID2,JAM2,GRID2,LRP2,SEMA6D,MBTPS2,*

			SMOC1,FER,SETDB2,RYR2,FBXL17,PRLR,NFATC2,FAT3,NTNG1,MTPN,MTMR2,LRP12,FGF10,SOX6,CADM1,SGCD,RC3H2,PCDH9,ATF6,SP3,VCL,SEZ6L,VCAM1,RAPGEF2,SHANK2,RAPGEF5,INSR,AKAP13,IMMP2L,SYNJ1,KLF15,UNC5D,ATXN1,GABRG2,FAT1,ULK2,NRXN3,RIMS2,KCNQ3,SCN8A,ZFHX3,ASTN1,GFRA1,BMPR1B,CPE,SEMA5A,CABLES1,ATP8A2,KITLG,PYGO1,ARHGAP24,FREM1,MAP4K4,CTNNA2,APC,APLF,DNER,ADGRV1,RELN,MACROD2,BNC2,AUTS2,ROR1,PPP2R3A,SNAI2,CYFIP2,ADAMTSL1,NDFIP1,TNR,PTPN13,CELF4,AKT3,CRB1,VAV3,BCAS3,PRKCA,USH1C,DNAH5,CNTN6,APP,PDLIM5,RPS6KA3,XYLT1,SMAD5,SLC6A3,NYAP2,VPS13B,ALPK2,ASB2,ADGRB1,CCDC88A,GHRH,ARNT2,SPOCK1
GO:0007275	multicellular organism development	1.9452001975456464e-33	ERBB4,PDE6A,RNF38,MACF1,PAK1,CTNND2,TRIO,MITF,RTN1,SETD2,AMBRA1,SYNE2,EPN2,DOP1B,IGSF3,ALPK3,MYO3B,EVC,NRP1,TEAD1,NFIA,FRY,CDH13,MDGA2,RC3H1,RPS6KA5,LHFPL2,TAOK3,LRFN5,ABL2,PRKCB,SLC23A2,ARHGEF7,SLC8A1,DACH1,EML1,CMIP,KCNC1,STAU2,SIAH3,CNTN4,ST8SIA4,AFG3L2,SEMA3A,SLC8A3,RFX3,RBFOX1,STARD13,MMP16,SLC40A1,LRRC4C,CADM2,ALCAM,ATP11C,ADAM32,MYEF2,DCLK1,DNAH11,ADAMTS9,NIPBL,CEP120,MBD5,KIF16B,SDC2,NCAM1,CLSTN2,TCF12,KDM4B,ADGRL2,NEBL,ITGA8,TCF4,IREB2,PBX1,PHACTR1,TTC21B,ABCD2,SLC39A12,DISC1,HECW2,FMN1,EYA1,RORB,KIAA1217,GABRB1,NTM,APBA2,ASAP1,TTLL7,EDAR,ATP2B2,FYN,AGO2,CREBBP,SLC1A2,ZBTB16,CSMD3,B4GALT6,SLIT3,GRIN2B,ANTXR1,SRGAP2,ARSB,GRIA1,TAFA5,NEGR1,SPRED1,SPEN,NAV2,FGF12,TPM1,COL19A1,CRIM1,NOTCH2,TOX,PCDH15,ESR1,SRGAP2C,ABCB5,TMOD2,ZSWIM6,MEGF11,TJP1,ARHGAP26,LRIG1,PRKACB,RIMS1,LAMA1,JCAD,GLI3,BRINP1,NRXN1,NTRK3,SGCG,FBN1,WWOX,WASF3,ARID1B,HYDIN,TSPAN2,WDPCP,MOSMO,ZNF521,PBX3,LSAMP,HMGA2,FHOD3,GREB1L,RERE,SLC4A10,PHLPP1,ATL1,MYO1E,ZDHHC17,ITGA4,ROCK1,HERC1,CACNA1C,COL4A2,DNM3,SYT1,BBS2,ARHGAP32,PRKN,DPF3,FOXP2,SCAPER,SIAH2,LATS2,EXOC4,RORA,PTPRD,RANBP3L,TG,HDAC4,ADGRB3,ADAM29,DGKG,MYO9A,MYO3A,FOXN3,NTRK2,PLCB1,MAPK1,LAMB1,SDCBP,ATP9A,BPTF,GRIK1,SOX5,PRKG1,DSCAM,BIRC6,NFIB,DIP2B,THSD7A,EGFR,PPFIA2,PTPRQ,NCAM2,BCR,MAGI2,NELL1,AKAP6,LARGE1,MBNL1,RTTN,ARHGEF28,FUT9,MYT1L,SRGAP2B,SCN2A,CNTN1,ZNRF3,POU6F2,DOCK10,TENM3,OPCML,ITPKB,GRM5,MED1,ENPEP,EPHA6,FNDC3A,CA10,AIMP1,LDB2,NECAB1,NRG3,NLGN1,ABL1,PTPRG,JARID2,JAM2,GRID2,LRP2,SEMA6D,MBTPS2,SMOC1,FER,SETDB2,RYR2,FBXL17,PRLR,NFATC2,FAT3,NTNG1,MTPN,MTMR2,LRP12,FGF10,SOX6,CADM1,SGCD,RC3H2,PCDH9,ATF6,SP3,VCL,SEZ6L,ZDHHC21,VCAM1,

			RAPGEF2,SHANK2,RAPGEF5,INSR,AKAP13,IMMP2L,SYNJ1,KLF15,UNC5D,ATXN1,GABRG2,FAT1,ENPP1,ULK2,NRXN3,RIMS2,KCNQ3,SCN8A,ZFHX3,ASTN1,GFRA1,BMPR1B,CPE,SEMA5A,CABLES1,ATP8A2,KITLG,PYGO1,ARHGAP24,FREM1,MAP4K4,CTNNA2,APC,APLF,DNER,ADGRV1,RELN,MACROD2,BNC2,AUTS2,ROR1,PPP2R3A,BMP2K,SNAI2,CYFIP2,ADAMTSL1,NDFIP1,TNR,PTPN13,CELF4,AKT3,ST8SIA6,CRB1,VAV3,BCAS3,PRKCA,USH1C,DNAH5,CNTN6,APP,PDLIM5,RPS6KA3,XYLT1,SMAD5,SLC6A3,NYAP2,VPS13B,ALPK2,ASB2,EYA2,ADGRB1,CCDC88A,GHRH,ARNT2,AFF3,SPOCK1
GO:0007399	nervous system development	2.0385723718164833e-33	*ERBB4,MACF1,PAK1,CTNND2,TRIO,RTN1,SETD2,AMBRA1,SYNE2,NRP1,NFIA,FRY,MDGA2,RPS6KA5,TAOK3,LRFN5,ABL2,SLC23A2,ARHGEF7,EML1,KCNC1,STAU2,CNTN4,ST8SIA4,AFG3L2,SEMA3A,SLC8A3,RBFOX1,LRRC4C,CADM2,ALCAM,MYEF2,DCLK1,DNAH11,NIPBL,CEP120,MBD5,SDC2,NCAM1,CLSTN2,TCF12,KDM4B,ADGRL2,ITGA8,TCF4,PBX1,PHACTR1,TTC21B,ABCD2,SLC39A12,DISC1,HECW2,EYA1,RORB,GABRB1,NTM,APBA2,ASAP1,TTLL7,ATP2B2,FYN,SLC1A2,ZBTB16,CSMD3,B4GALT6,SLIT3,GRIN2B,SRGAP2,ARSB,GRIA1,NEGR1,SPEN,NAV2,FGF12,CRIM1,NOTCH2,TOX,PCDH15,SRGAP2C,TMOD2,ZSWIM6,ARHGAP26,LRIG1,PRKACB,RIMS1,LAMA1,GLI3,BRINP1,NRXN1,NTRK3,WASF3,ARID1B,HYDIN,TSPAN2,WDPCP,MOSMO,ZNF521,PBX3,LSAMP,RERE,SLC4A10,ATL1,ZDHHC17,ITGA4,ROCK1,HERC1,DNM3,SYT1,BBS2,ARHGAP32,PRKN,DPF3,FOXP2,RORA,PTPRD,TG,HDAC4,ADGRB3,DGKG,MYO9A,NTRK2,PLCB1,MAPK1,LAMB1,SDCBP,ATP9A,BPTF,GRIK1,SOX5,PRKG1,DSCAM,NFIB,DIP2B,EGFR,PPFIA2,PTPRQ,NCAM2,BCR,MAGI2,NELL1,LARGE1,MBNL1,ARHGEF28,FUT9,MYT1L,SRGAP2B,SCN2A,CNTN1,POU6F2,DOCK10,TENM3,OPCML,GRM5,MED1,EPHA6,CA10,LDB2,NRG3,NLGN1,ABL1,PTPRG,JARID2,JAM2,GRID2,LRP2,SEMA6D,FBXL17,FAT3,NTNG1,MTPN,MTMR2,LRP12,FGF10,SOX6,PCDH9,VCL,SEZ6L,VCAM1,RAPGEF2,SHANK2,RAPGEF5,IMMP2L,SYNJ1,KLF15,UNC5D,ATXN1,GABRG2,ULK2,NRXN3,RIMS2,KCNQ3,SCN8A,ZFHX3,ASTN1,GFRA1,BMPR1B,SEMA5A,CABLES1,ATP8A2,MAP4K4,CTNNA2,APC,DNER,ADGRV1,RELN,MACROD2,AUTS2,ROR1,PPP2R3A,CYFIP2,ADAMTSL1,TNR,PTPN13,AKT3,CRB1,USH1C,DNAH5,CNTN6,APP,PDLIM5,RPS6KA3,SLC6A3,NYAP2,VPS13B,ADGRB1,CCDC88A,GHRH,ARNT2,SPOCK1*
GO:0048856	anatomical structure development	2.1079684661783256e-29	*FSTL1,ERBB4,PDE6A,RNF38,MACF1,PAK1,CTNND2,TRIO,MITF,RTN1,SETD2,AMBRA1,SYNE2,EPN2,DOP1B,IGSF3,ALPK3,MYO3B,EVC,NRP1,TEAD1,NFIA,FRY,CDH13,MDGA2,RC3H1,RPS6KA5,LHFPL2,EPS8,TAOK3,LRFN5,ABL2,PRKCB,SLC23A2,ARHGEF7,UTRN,SLC8A1,DACH1,EML1,CMIP,ANKRD6,KCNC1,STAU2,SIAH3,CNTN4,ST8SIA4,AFG3L2,LDLRAD4,SEMA3A,S*

			LC8A3,RFX3,RBFOX1,STARD13,MMP16,SLC40A1,LRRC4C,CADM2,ALCAM,ATP11C,LIMD1,ADAM32,MYEF2,DCLK1,DNAH11,ADAMTS9,NIPBL,CEP120,CDC42EP3,FGD4,MBD5,KIF16B,SDC2,NCAM1,CLSTN2,TCF12,KDM4B,ADGRL2,TANC1,NEBL,ITGA8,SYCP1,MYO10,TCF4,IREB2,PBX1,FAM171A1,ATP10A,PHACTR1,TTC21B,ABCD2,SLC39A12,DISC1,CLTCL1,HECW2,FMN1,EYA1,RORB,KIAA1217,GABRB1,SVIL,NTM,APBA2,ASAP1,TTLL7,EDAR,ATP2B2,FRMD6,FYN,SH3KBP1,AGO2,CREBBP,SLC1A2,ZBTB16,CSMD3,B4GALT6,SLIT3,GRIN2B,ANTXR1,SRGAP2,ARSB,GRIA1,TAFA5,NEGR1,SPRED1,SPEN,NAV2,FGF12,TPM1,COL19A1,CRIM1,NOTCH2,FLNB,TOX,PCDH15,ESR1,SRGAP2C,ABCB5,TMOD2,ZSWIM6,MEGF11,PDE4D,TJP1,ARHGAP26,LRIG1,PRKACB,RIMS1,PARVB,LAMA1,JCAD,GLI3,BRINP1,NRXN1,NTRK3,SGCG,FBN1,WWOX,WASF3,ARID1B,HYDIN,TSPAN2,CPQ,WDPCP,MOSMO,PIAS1,CDH20,ZNF521,PBX3,PRICKLE2,LSAMP,HMGA2,FHOD3,GREB1L,RERE,SLC4A10,PHLPP1,ATL1,MYO1E,ZDHHC17,ITGA4,ROCK1,HERC1,DOCK1,CACNA1C,RXRG,COL4A2,DNM3,PACRG,SYT1,BBS2,ARHGAP32,ASXL3,PRKN,DPF3,FOXP2,SCAPER,SIAH2,LATS2,EXOC4,RORA,PTPRD,RANBP3L,TG,HDAC4,ADGRB3,ADAM29,DGKG,MYO9A,MYO3A,FOXN3,NTRK2,PLCB1,MAPK1,LAMB1,SDCBP,ATP9A,BPTF,GRIK1,SOX5,PRKG1,DSCAM,BIRC6,NFIB,DIP2B,TUT4,THSD7A,EGFR,PPFIA2,PTPRQ,NCAM2,BCR,MAGI2,NELL1,AKAP6,LARGE1,MBNL1,RTTN,ARHGEF28,FUT9,MYT1L,SRGAP2B,SCN2A,CNTN1,ZNRF3,POU6F2,DOCK10,TENM3,OPCML,ITPKB,GRM5,MED1,ENPEP,EPHA6,FNDC3A,CA10,SPIRE1,AIMP1,LDB2,NECAB1,WDR72,NRG3,PID1,NLGN1,ABL1,PTPRG,JARID2,PAQR5,EBF2,JAM2,GRID2,LRP2,SEMA6D,MBTPS2,SMOC1,FER,SETDB2,RYR2,FBXL17,PRLR,ZNF160,NFATC2,FAT3,NTNG1,MTPN,MTMR2,LRP12,FGF10,SOX6,CADM1,SGCD,RC3H2,PCDH9,ATF6,EYS,SP3,VCL,SEZ6L,ZDHHC21,XKR6,VCAM1,RAPGEF2,CDH7,IFT81,SHANK2,RAPGEF5,INSR,AKAP13,ILDR2,IMMP2L,SYNJ1,KLF15,UNC5D,ATXN1,GABRG2,FAT1,ENPP1,ULK2,NRXN3,RIMS2,KCNQ3,SCN8A,ZFHX3,PGM5,ASTN1,GFRA1,BMPR1B,CPE,SEMA5A,CABLES1,ATP8A2,SLC24A3,KITLG,PYGO1,ARHGAP24,FREM1,MAP4K4,CTNNA2,APC,APLF,GPC6,DNER,ADGRV1,RELN,MACROD2,BNC2,ANXA4,AUTS2,ROR1,PPP2R3A,BMP2K,SNAI2,CYFIP2,KAZN,ADAMTSL1,NDFIP1,TNR,PTPN13,OCA2,CELF4,AKT3,ST8SIA6,CRB1,VAV3,BCAS3,PRKCA,USH1C,MRTFB,DNAH5,CNTN6,FMN2,APP,PDLIM5,RPS6KA3,XYLT1,SMAD5,SLC6A3,NYAP2,VPS13B,ALPK2,ASB2,EYA2,ADGRB1,CCDC88A,GHRH,ARNT2,AFF3,SPOCK1
GO:0032502	developmental process	4.9622408542145834e-27	*FSTL1,ERBB4,PDE6A,RNF38,MACF1,PAK1,CTNND2,TRIO,MITF,RTN1,SETD2,AMBRA1,SYNE2,EPN2,DOP1B,IGSF3,ALPK3,MYO3B,EVC,NR*

			P1,TEAD1,NFIA,FRY,CDH13,MDGA2,RC3H1,RPS6KA5,LHFPL2,EPS8,TAOK3,HERC2,LRFN5,ABL2,PRKCB,SLC23A2,ARHGEF7,UTRN,SLC8A1,DACH1,EML1,CMIP,CDK12,ANKRD6,HTR2C,KCNC1,STAU2,SIAH3,CNTN4,ST8SIA4,AFG3L2,LDLRAD4,SEMA3A,SLC8A3,RFX3,RBFOX1,STARD13,MMP16,SLC40A1,LRRC4C,CADM2,ALCAM,ATP11C,LIMD1,ADAM32,MYEF2,DCLK1,DNAH11,SLC9C1,ADAMTS9,NIPBL,CEP120,CDC42EP3,FGD4,MBD5,KIF16B,SDC2,NCAM1,CLSTN2,TCF12,KDM4B,ADGRL2,TANC1,NEBL,ITGA8,SYCP1,MYO10,TCF4,IREB2,PBX1,FAM171A1,ATP10A,PHACTR1,BICRAL,TTC21B,ABCD2,SLC39A12,DISC1,CLTCL1,HECW2,FMN1,EYA1,RORB,KIAA1217,GABRB1,SVIL,NTM,APBA2,ASAP1,TTLL7,EDAR,ATP2B2,ANKRD26,FRMD6,FYN,SH3KBP1,AGO2,CREBBP,SLC1A2,ZBTB16,CSMD3,B4GALT6,SLIT3,GRIN2B,ANTXR1,SRGAP2,ARSB,GRIA1,TAFA5,NEGR1,SPRED1,SPEN,NAV2,FGF12,SPATA48,TPM1,COL19A1,CRIM1,NOTCH2,FLNB,TOX,PCDH15,ESR1,SRGAP2C,ABCB5,TMOD2,ZSWIM6,MEGF11,PDE4D,TJP1,ARHGAP26,LRIG1,PRKACB,RIMS1,PARVB,LAMA1,JCAD,GLI3,BRINP1,NRXN1,NTRK3,SGCG,FBN1,WWOX,WASF3,ARID1B,HYDIN,TSPAN2,CPQ,WDPCP,MOSMO,PIAS1,CDH20,ZNF521,PBX3,PRICKLE2,LSAMP,HMGA2,FHOD3,GREB1L,RERE,SLC4A10,PHLPP1,ATL1,MYO1E,ZDHHC17,ITGA4,ROCK1,HERC1,DOCK1,CACNA1C,GLIS1,RXRG,COL4A2,DNM3,PACRG,SYT1,BBS2,ARHGAP32,ASXL3,PRKN,DPF3,FOXP2,SCAPER,SIAH2,LATS2,EXOC4,RORA,PTPRD,BBS9,RANBP3L,TG,HDAC4,ADGRB3,ADAM29,DGKG,MYO9A,MYO3A,FOXN3,NTRK2,PLCB1,MAPK1,LAMB1,SDCBP,ATP9A,BPTF,GRIK1,SOX5,PRKG1,DSCAM,BIRC6,NFIB,DIP2B,TUT4,THSD7A,EGFR,PPFIA2,PTPRQ,MGA,NCAM2,BCR,MAGI2,NELL1,AKAP6,LARGE1,MBNL1,RTTN,ARHGEF28,FUT9,MYT1L,SRGAP2B,SCN2A,CNTN1,ZNRF3,POU6F2,ZNF449,DOCK10,TENM3,OPCML,ITPKB,GRM5,MED1,ENPEP,EPHA6,FNDC3A,CA10,SPIRE1,AIMP1,LDB2,NECAB1,WDR72,NRG3,PID1,NLGN1,ABL1,PTPRG,JARID2,PAQR5,EBF2,JAM2,GRID2,LRP2,SEMA6D,MBTPS2,SMOC1,FER,SETDB2,RYR2,FBXL17,PRLR,ZNF160,NFATC2,FAT3,NTNG1,MED15,MTPN,VSTM2A,MTMR2,LRP12,FGF10,SOX6,CADM1,SGCD,RC3H2,PCDH9,ATF6,EYS,SP3,VCL,SEZ6L,ZDHHC21,XKR6,VCAM1,RAPGEF2,CDH7,IFT81,SHANK2,RAPGEF5,INSR,AKAP13,ILDR2,IMMP2L,SYNJ1,KLF15,UNC5D,ATXN1,GABRG2,FAT1,ENPP1,ULK2,UNC13C,NRXN3,RIMS2,KCNQ3,SCN8A,ZFHX3,PGM5,ASTN1,GFRA1,BMPR1B,CPE,FCRLA,SEMA5A,CABLES1,ATP8A2,SLC24A3,KITLG,PYGO1,ARHGAP24,FREM1,MAP4K4,CTNNA2,PRAME,APC,APLF,GPC6,DNER,ABCA5,ADGRV1,RELN,MACROD2,BNC2,ANXA4,AUTS2,ROR1,PPP2R3A,BMP2K,SNAI2,CYFIP2,KAZN,ADAMTSL1,NDFIP1,TNR,PTPN13,OCA2,CELF4,AKT

			3,ST8SIA6,CRB1,VAV3,BCAS3,PRKCA,USH1C,MRTFB,DNAH5,CNTN6,FMN2,APP,PDLIM5,RPS6KA3,XYLT1,SMAD5,SLC6A3,DNAJC13,NYAP2,VPS13B,ALPK2,ASB2,EYA2,ADGRB1,CCDC88A,GHRH,ARNT2,AFF3,SPOCK1
GO:0032501	multicellular organismal process	4.3165146366581324e-24	FSTL1,SLMAP,ERBB4,KCNMA1,PDE6A,APBB2,RNF38,CUBN,MACF1,PAK1,CTNND2,TRIO,MITF,RTN1,SETD2,KIRREL1,AMBRA1,SYNE2,EPN2,NETO2,DOP1B,IGSF3,ALPK3,MYO3B,EVC,NRP1,TEAD1,NFIA,FRY,CDH13,MDGA2,RC3H1,RPS6KA5,LHFPL2,EPS8,TAOK3,HERC2,LRFN5,OR4C46,ABL2,PRKCB,SLC23A2,ARHGEF7,UTRN,SLC8A1,DACH1,EML1,CMIP,LNPEP,SLC2A13,HTR2C,KCNC1,DYSF,STAU2,SIAH3,TMEM63C,CNTN4,ST8SIA4,AFG3L2,LDLRAD4,SEMA3A,SLC8A3,RFX3,RBFOX1,STARD13,MMP16,SLC40A1,LRRC4C,CADM2,ALCAM,SLCO3A1,ATP11C,LIMD1,ADAM32,MYEF2,DCLK1,DNAH11,SLC9C1,ADAMTS9,NIPBL,CEP120,HMCN1,GRIK2,IGSF11,MBD5,KIF16B,SDC2,NCAM1,CLSTN2,TCF12,KDM4B,ADGRL2,TANC1,CORO2B,PAPPA,NEBL,ITGA8,SYCP1,ZBTB20,PJA2,ASB3,TCF4,TAFA4,GABRG1,IREB2,PBX1,PHACTR1,BICRAL,TTC21B,ABCD2,SLC39A12,DISC1,HECW2,FMN1,ARHGAP42,EYA1,RORB,PIEZO2,KIAA1217,GABRB1,NTM,APBA2,ASAP1,TTLL7,EDAR,ATP2B2,FYN,SH3KBP1,AGO2,CREBBP,SLC1A2,ZBTB16,CSMD3,DLGAP1,B4GALT6,SLIT3,GRIN2B,ANTXR1,SRGAP2,ARSB,GRIA1,TAFA5,NEGR1,SPRED1,SPEN,NAV2,FGF12,DGKB,CACNG2,BTBD9,SPATA48,TPM1,COL19A1,CRIM1,NOTCH2,TOX,PCDH15,ESR1,GABRG3,SRGAP2C,ABCB5,TMOD2,ZSWIM6,MEGF11,PDE4D,TJP1,ARHGAP26,LRIG1,PRKACB,RIMS1,LAMA1,JCAD,GLI3,DOCK4,BRINP1,NRXN1,NTRK3,SGCG,FBN1,WWOX,WASF3,ARID1B,HYDIN,TSPAN2,WDPCP,MOSMO,NEK10,PIAS1,ZNF521,PBX3,LSAMP,HMGA2,FHOD3,GRID1,GREB1L,ZNF287,RERE,SLC4A10,PHLPP1,ATL1,MYO1E,SLC7A2,ZDHHC17,PKN2,ITGA4,ABCC9,ROCK1,HERC1,DOCK1,CACNA1C,GLIS1,PRKAA2,ARHGEF11,COL4A2,DNM3,PACRG,SYT1,BBS2,GRB10,ARHGAP32,PRKN,DPF3,FOXP2,SCAPER,SIAH2,LATS2,EXOC4,PPP1R12B,RORA,PTPRD,BBS9,RANBP3L,TG,HDAC4,ADGRB3,ADAM29,DGKG,MYO9A,MYO3A,FOXN3,NTRK2,PLCB1,MAPK1,LAMB1,SDCBP,ATP9A,BPTF,GRIK1,SOX5,PRKG1,DSCAM,BIRC6,DGKI,NFIB,DIP2B,TUT4,THSD7A,NBEA,EGFR,PPFIA2,PTPRQ,NCAM2,CLCN5,BCR,MAGI2,NELL1,AKAP6,LARGE1,MBNL1,RTTN,ARHGEF28,PLCL1,FUT9,MYT1L,TMPRSS3,SRGAP2B,SORCS3,SCN2A,CNTN1,ZNRF3,POU6F2,GNAQ,ZNF449,DOCK10,TENM3,OPCML,ITPKB,GRM5,MED1,ENPEP,EPHA6,FNDC3A,CA10,SPIRE1,AIMP1,LDB2,NECAB1,NRG3,ATP8A1,NLGN1,ABL1,PTPRG,NCOR1,JARID2,PAQR5,OR9Q1,EBF2,JAM2,GRID2,LRP2,ERBIN,SEMA6D,MBTPS2,SMOC1,FER,PAK5,SETDB2,RYR2,IL17RA,FBXL17,MCC,PRLR,NFATC2,FAT3,C

			ACNB2,NTNG1,MED15,MTPN,GNG7,MTMR2,LRP12,MAPRE2,FGF10,NOS1AP,SOX6,CADM1,SGCD,RC3H2,PCDH9,ATF6,EYS,SP3,VCL,SEZ6L,ZDHHC21,VCAM1,RAPGEF2,IFT81,SHANK2,RAPGEF5,INSR,AKAP13,ILDR2,IMMP2L,PPIP5K2,SYNJ1,KLF15,UNC5D,ATXN1,GABRG2,FAT1,ENPP1,ULK2,NRXN3,RIMS2,RHPN2,PPM1F,KCNQ3,SCN8A,ZFHX3,ASTN1,DLGAP2,GFRA1,BMPR1B,MAPKBP1,CPE,KCND2,SEMA5A,CABLES1,ATP8A2,SLC24A3,KITLG,PYGO1,LHFPL3,ARHGAP24,FREM1,MAP4K4,CTNNA2,APC,APLF,DNER,ABCA5,ADGRV1,RELN,MACROD2,AKAP11,BNC2,ANXA4,CYBRD1,AUTS2,ROR1,PPP2R3A,BMP2K,SNAI2,CYFIP2,SLC4A4,KAZN,ADAMTSL1,NDFIP1,TNR,PTPN13,DTNA,OCA2,CELF4,AKT3,ST8SIA6,CRB1,VAV3,IL1R1,BCAS3,PRKCA,USH1C,MRTFB,DNAH5,CNTN6,ENPP3,FMN2,APP,CHST3,PDLIM5,RPS6KA3,XYLT1,SMAD5,SLC6A3,DNAJC13,NYAP2,VPS13B,ALPK2,ASB2,EYA2,MAPK8IP1,ADGRB1,CCDC88A,GHRH,ARNT2,RAP1GDS1,AFF3,SPOCK1
GO:0048699	generation of neurons	1.619665827232924e-23	ERBB4,MACF1,PAK1,CTNND2,TRIO,RTN1,NRP1,NFIA,FRY,MDGA2,RPS6KA5,TAOK3,ABL2,SLC23A2,EML1,STAU2,CNTN4,AFG3L2,SEMA3A,LRRC4C,ALCAM,MYEF2,DCLK1,NIPBL,SDC2,NCAM1,TCF12,TCF4,PBX1,PHACTR1,TTC21B,SLC39A12,DISC1,HECW2,EYA1,RORB,GABRB1,NTM,ASAP1,ATP2B2,FYN,CSMD3,B4GALT6,SLIT3,SRGAP2,ARSB,NEGR1,NOTCH2,TOX,PCDH15,SRGAP2C,ZSWIM6,RIMS1,LAMA1,GLI3,BRINP1,NRXN1,NTRK3,TSPAN2,WDPCP,MOSMO,ZNF521,PBX3,RERE,SLC4A10,ATL1,ZDHHC17,ITGA4,ROCK1,HERC1,DNM3,SYT1,ARHGAP32,PRKN,RORA,PTPRD,ADGRB3,DGKG,MYO9A,NTRK2,LAMB1,ATP9A,SOX5,PRKG1,DSCAM,NFIB,DIP2B,PPFIA2,PTPRQ,NCAM2,MAGI2,LARGE1,ARHGEF28,FUT9,MYT1L,CNTN1,DOCK10,TENM3,OPCML,MED1,EPHA6,NRG3,NLGN1,ABL1,PTPRG,GRID2,LRP2,SEMA6D,FAT3,NTNG1,MTPN,MTMR2,LRP12,VCL,VCAM1,RAPGEF2,UNC5D,ULK2,NRXN3,RIMS2,KCNQ3,ZFHX3,ASTN1,GFRA1,BMPR1B,SEMA5A,ATP8A2,MAP4K4,CTNNA2,DNER,ADGRV1,RELN,AUTS2,ROR1,PPP2R3A,CYFIP2,ADAMTSL1,TNR,CRB1,USH1C,CNTN6,APP,PDLIM5,NYAP2,VPS13B,ADGRB1,CCDC88A,SPOCK1
GO:0022008	neurogenesis	8.336900134274317e-23	ERBB4,MACF1,PAK1,CTNND2,TRIO,RTN1,SYNE2,NRP1,NFIA,FRY,MDGA2,RPS6KA5,TAOK3,ABL2,SLC23A2,EML1,STAU2,CNTN4,AFG3L2,SEMA3A,SLC8A3,LRRC4C,ALCAM,MYEF2,DCLK1,NIPBL,CEP120,SDC2,NCAM1,TCF12,TCF4,PBX1,PHACTR1,TTC21B,SLC39A12,DISC1,HECW2,EYA1,RORB,GABRB1,NTM,ASAP1,ATP2B2,FYN,CSMD3,B4GALT6,SLIT3,SRGAP2,ARSB,NEGR1,SPEN,NAV2,NOTCH2,TOX,PCDH15,SRGAP2C,ZSWIM6,RIMS1,LAMA1,GLI3,BRINP1,NRXN1,NTRK3,WASF3,TSPAN2,WDPCP,MOSMO,ZNF521,PBX3,RERE,SLC4A10,ATL1,ZDHHC17,ITGA4,ROCK1,HERC1,DNM3,SYT1,ARHGAP32,

			PRKN,RORA,PTPRD,ADGRB3,DGKG,MYO9A,NTRK2,MAPK1,LAMB1,ATP9A,SOX5,PRKG1,DSCAM,NFIB,DIP2B,EGFR,PPFIA2,PTPRQ,NCAM2,MAGI2,LARGE1,ARHGEF28,FUT9,MYT1L,CNTN1,DOCK10,TENM3,OPCML,GRM5,MED1,EPHA6,NRG3,NLGN1,ABL1,PTPRG,GRID2,LRP2,SEMA6D,FAT3,NTNG1,MTPN,MTMR2,LRP12,FGF10,SOX6,VCL,VCAM1,RAPGEF2,KLF15,UNC5D,ULK2,NRXN3,RIMS2,KCNQ3,ZFHX3,ASTN1,GFRA1,BMPR1B,SEMA5A,ATP8A2,MAP4K4,CTNNA2,DNER,ADGRV1,RELN,AUTS2,ROR1,PPP2R3A,CYFIP2,ADAMTSL1,TNR,CRB1,USH1C,CNTN6,APP,PDLIM5,NYAP2,VPS13B,ADGRB1,CCDC88A,SPOCK1
GO:0030182	neuron differentiation	1.5608997182808606e-22	*ERBB4,MACF1,PAK1,CTNND2,TRIO,RTN1,NRP1,NFIA,FRY,MDGA2,RPS6KA5,TAOK3,ABL2,SLC23A2,STAU2,CNTN4,AFG3L2,SEMA3A,LRRC4C,ALCAM,MYEF2,DCLK1,SDC2,NCAM1,TCF12,TCF4,PBX1,PHACTR1,TTC21B,SLC39A12,DISC1,HECW2,EYA1,RORB,GABRB1,NTM,ASAP1,ATP2B2,FYN,CSMD3,B4GALT6,SLIT3,SRGAP2,ARSB,NEGR1,NOTCH2,TOX,PCDH15,SRGAP2C,ZSWIM6,RIMS1,LAMA1,GLI3,BRINP1,NRXN1,NTRK3,TSPAN2,WDPCP,MOSMO,ZNF521,PBX3,RERE,SLC4A10,ATL1,ZDHHC17,ITGA4,ROCK1,HERC1,DNM3,SYT1,ARHGAP32,PRKN,RORA,PTPRD,ADGRB3,DGKG,MYO9A,NTRK2,LAMB1,ATP9A,PRKG1,DSCAM,NFIB,DIP2B,PPFIA2,PTPRQ,NCAM2,MAGI2,ARHGEF28,FUT9,MYT1L,CNTN1,DOCK10,TENM3,OPCML,MED1,EPHA6,NLGN1,ABL1,PTPRG,GRID2,LRP2,SEMA6D,FAT3,NTNG1,MTPN,MTMR2,LRP12,VCL,VCAM1,RAPGEF2,UNC5D,ULK2,NRXN3,RIMS2,KCNQ3,ZFHX3,GFRA1,BMPR1B,SEMA5A,ATP8A2,MAP4K4,CTNNA2,ADGRV1,RELN,AUTS2,ROR1,PPP2R3A,CYFIP2,ADAMTSL1,TNR,CRB1,USH1C,CNTN6,APP,PDLIM5,NYAP2,VPS13B,ADGRB1,CCDC88A,SPOCK1*
GO:0009653	anatomical structure morphogenesis	9.094820197334549e-22	*ERBB4,MACF1,PAK1,CTNND2,TRIO,SETD2,EPN2,MYO3B,NRP1,NFIA,FRY,CDH13,RC3H1,RPS6KA5,EPS8,TAOK3,PRKCB,SLC23A2,ARHGEF7,ANKRD6,STAU2,CNTN4,AFG3L2,SEMA3A,STARD13,MMP16,SLC40A1,LRRC4C,ALCAM,LIMD1,DCLK1,DNAH11,ADAMTS9,NIPBL,CDC42EP3,FGD4,KIF16B,SDC2,NCAM1,TANC1,NEBL,ITGA8,MYO10,PBX1,FAM171A1,ATP10A,PHACTR1,TTC21B,SLC39A12,DISC1,CLTCL1,HECW2,FMN1,EYA1,RORB,EDAR,FRMD6,FYN,SH3KBP1,AGO2,CREBBP,ZBTB16,B4GALT6,SLIT3,SRGAP2,TAFA5,SPRED1,TPM1,NOTCH2,FLNB,PCDH15,ESR1,TMOD2,MEGF11,TJP1,LRIG1,PRKACB,RIMS1,PARVB,LAMA1,JCAD,GLI3,NRXN1,FBN1,WWOX,WASF3,WDPCP,CDH20,PBX3,PRICKLE2,HMGA2,FHOD3,GREB1L,RERE,SLC4A10,ATL1,MYO1E,ZDHHC17,ITGA4,ROCK1,HERC1,DOCK1,CACNA1C,COL4A2,DNM3,SYT1,BBS2,ARHGAP32,ASXL3,PRKN,LATS2,EXOC4,RORA,PTPRD,ADGRB3,MYO9A,MYO3A,FOXN3,NTRK2,MAPK1,LAMB1,ATP9A,SOX5,PRKG1,DSCAM,NFI*

			B,DIP2B,THSD7A,EGFR,PPFIA2,PTPRQ,BCR,MAGI2,LARGE1,MBNL1,ARHGEF28,CNTN1,ZNRF3,DOCK10,TENM3,MED1,ENPEP,EPHA6,SPIRE1,AIMP1,WDR72,NRG3,PID1,NLGN1,ABL1,JAM2,GRID2,LRP2,SEMA6D,SETDB2,RYR2,NFATC2,FAT3,NTNG1,MTPN,MTMR2,FGF10,SOX6,SGCD,SP3,VCL,RAPGEF2,CDH7,INSR,AKAP13,UNC5D,FAT1,ULK2,NRXN3,RIMS2,PGM5,BMPR1B,CPE,SEMA5A,ATP8A2,ARHGAP24,FREM1,MAP4K4,CTNNA2,APC,GPC6,RELN,AUTS2,ROR1,PPP2R3A,SNAI2,CYFIP2,ADAMTSL1,TNR,AKT3,CRB1,VAV3,BCAS3,PRKCA,USH1C,CNTN6,APP,PDLIM5,SMAD5,SLC6A3,NYAP2,VPS13B,ALPK2,ASB2,EYA2,ADGRB1,AFF3
GO:0007154	cell communication	1.0951193880004e-21	FSTL1,ERBB4,PDE6A,APBB2,FYB2,MACF1,PAK1,CTNND2,TRIO,MITF,AMBRA1,EPN2,NETO2,PDE1C,EVC,NRP1,TEAD1,NFIA,CDH13,RC3H1,RPS6KA5,EPS8,TAOK3,OR4C46,ABL2,PRKCB,ARHGEF7,UTRN,GPC5,MAML2,ARHGEF12,SAMD12,SLC8A1,LNPEP,ANKRD6,RERG,HTR2C,DYSF,STAU2,CACNA1E,SNTG1,CNTN4,USP14,CNKSR3,LDLRAD4,SEMA3A,PLIN2,SLC8A3,RFX3,MGAT5,STARD13,LRRC4C,ALCAM,ERLIN2,LIMD1,PIK3C3,DCLK1,MAGI3,CDC42EP3,FGD4,GRIK2,SNX25,IGSF11,RGL1,CCDC186,MBD5,KIF16B,NCAM1,CLSTN2,SIPA1L2,RCAN2,ADGRL2,GNG12,PAPPA,ITGA8,VPS41,GRIK4,MYO10,PJA2,ASB3,TAFA4,GABRG1,TOM1L2,TTC21B,NEK6,SLC39A12,MOB1B,ARHGAP28,DISC1,SV2B,RIC3,RALGPS1,ARHGAP42,EYA1,KSR1,RORB,GPR158,GABRB1,ANKS1B,APBA2,EDAR,FRMD6,RGS20,FYN,SH3KBP1,NALCN,CREBBP,SLC1A2,CADPS,DLGAP1,SLIT3,RGS7,FHIT,GRIN2B,SRGAP2,STK32A,GRIA1,TRABD2B,TAFA5,SPRED1,MYO5A,SPEN,FGF12,DGKB,CACNG2,GARNL3,BTBD9,CRIM1,NOTCH2,FLNB,MADD,ESR1,GABRG3,NFAT5,TMOD2,PLCXD3,PDE4D,TJP1,ARHGAP26,ERC2,PTPRT,PRKACB,GNG2,TRHDE,RIMS1,HIPK3,WSB1,FCHSD2,SGMS1,LAMA1,JCAD,GLI3,DOCK4,MAST4,GAST,ITPR2,NRXN1,NTRK3,FBN1,WWOX,WDPCP,MAGI1,MOSMO,RAPGEF4,CNKSR2,NEK10,PIAS1,PRICKLE2,HMGA2,HHAT,GRID1,PARP8,SEL1L,SLC4A10,PHLPP1,SYN2,MYO1E,ZDHHC17,PKN2,ITGA4,GSG1L,ROCK1,DCDC1,DOCK1,CACNA1C,ARHGAP31,UBR1,RXRG,PRKAA2,ARHGEF11,AMPH,SORCS1,COL4A2,SYT1,BBS2,GRB10,ARHGAP32,PRKN,SIAH2,LATS2,EXOC4,DOCK9,DLG2,PPP1R12B,RORA,PTPRD,CDC14B,TTR,PTPRN2,TG,HDAC4,ADGRB3,INPP4B,DGKG,MYO9A,FOXN3,NTRK2,PLCB1,MAPK1,LAMB1,SDCBP,GRIK1,CUL5,PRKG1,DSCAM,BIRC6,DGKI,NLK,EGFR,PPFIA2,BCR,MAGI2,AKAP6,LARGE1,ARHGEF28,PLCL1,MOK,TBC1D1,IQCJ-SCHIP1,SORCS3,SCN2A,RNF152,CNTN1,ZNRF3,GNAQ,DOCK10,TENM3,ITPKB,GRM5,MED1,ENPEP,EPHA6,ARAP2,AIMP1,NRG3,CPEB4,PID1,NLGN1,ABL1,PTPRG,NCOR1,OR9Q1,GRID2,ELMO1,LRP2,ERBIN,SEMA6D,PPM1L,CABIN1,

			MBTPS2,FER,PAK5,RYR2,IL17RA,FBXL17,MCC,PRLR,NFATC2,DST,GRIK3,CACNB2,STXBP4,NTNG1,GNG7,MTMR2,LRP12,MAPRE2,FGF10,NOS1AP,CADM1,SGCD,RC3H2,RIC8B,ATF6,STK32B,ZNF106,SEZ6L,ZDHHC21,VCAM1,RAPGEF2,RGS12,IFT81,SHANK2,ZMYND11,RAPGEF5,INSR,SRGAP3,PDK1,AKAP13,ILDR2,SYNJ1,KLF15,DENND4C,UNC5D,GABRG2,ARFGEF1,FAT1,PATJ,ITGA9,ENPP1,ULK2,UNC13C,NRXN3,RIMS2,RHPN2,PPM1F,KCNQ3,SCN8A,DENND2B,DLGAP2,GFRA1,ATRNL1,GPR156,BMPR1B,NSG1,MAPKBP1,CPE,KCND2,FCRLA,SEMA5A,KITLG,PYGO1,SV2C,ARHGAP24,FREM1,GRIA4,MAP4K4,CNIH3,DOCK3,PRAME,APC,GPC6,DNER,ADGRV1,RELN,AKAP11,ANXA4,AUTS2,ROR1,PPP2R3A,MTMR3,BMP2K,SNAI2,CYFIP2,NDFIP1,TNR,DTNA,CELF4,AKT3,CRB1,VAV3,IL1R1,BCAS3,PRKCA,INPP5A,USP8,CNTN6,FMN2,ERC1,APP,RPS6KA3,SMAD5,SLC6A3,PSD3,ALPK2,ASB2,EYA2,MAPK8IP1,ADGRB1,CCDC88A,GHRH,RAP1GDS1
GO:0034330	cell junction organization	1.4195800179495067e-21	*ERBB4,APBB2,MACF1,CTNND2,KIRREL1,NRP1,NFIA,CDH13,RPS6KA5,LRFN5,ARHGEF7,STAU2,AFG3L2,SLC8A3,LRRC4C,PDZRN3,CLSTN2,ADGRL2,TANC1,CORO2B,DISC1,FMN1,PEAK1,ASAP1,FRMPD4,FYN,GRIN2B,SRGAP2,GRIA1,NEGR1,DGKB,CACNG2,CLDN10,SRGAP2C,TJP1,ERC2,PTPRT,NRXN1,WASF3,WDPCP,CNKSR2,CDH20,CTTNBP2,GRID1,PKN2,ROCK1,DNM3,PTPRD,TLN2,ADGRB3,MYO9A,NTRK2,SDCBP,DSCAM,NBEA,PPFIA2,BCR,LARGE1,DOCK10,GRM5,NRG3,NLGN1,ABL1,GRID2,FER,DST,CACNB2,NTNG1,MTMR2,MAPRE2,NOS1AP,MPDZ,VCL,SEZ6L,RAPGEF2,CDH7,SHANK2,INSR,GABRG2,PATJ,UNC13C,NRXN3,PPM1F,MAP4K4,CTNNA2,APC,DNER,RELN,SNAI2,TNR,PTPN13,PRKCA,ERC1,APP,PDLIM5,ADGRB1*
GO:0048666	neuron development	2.5272805882926964e-21	*MACF1,PAK1,CTNND2,TRIO,NRP1,FRY,RPS6KA5,TAOK3,ABL2,SLC23A2,STAU2,CNTN4,AFG3L2,SEMA3A,LRRC4C,ALCAM,DCLK1,SDC2,NCAM1,PBX1,PHACTR1,SLC39A12,DISC1,HECW2,RORB,GABRB1,NTM,ASAP1,FYN,CSMD3,B4GALT6,SLIT3,SRGAP2,ARSB,NEGR1,NOTCH2,TOX,PCDH15,SRGAP2C,RIMS1,LAMA1,GLI3,BRINP1,NRXN1,NTRK3,TSPAN2,WDPCP,PBX3,RERE,SLC4A10,ATL1,ZDHHC17,ITGA4,ROCK1,HERC1,DNM3,SYT1,ARHGAP32,PRKN,PTPRD,ADGRB3,DGKG,MYO9A,NTRK2,LAMB1,ATP9A,PRKG1,DSCAM,NFIB,DIP2B,PPFIA2,PTPRQ,NCAM2,MAGI2,ARHGEF28,FUT9,MYT1L,CNTN1,DOCK10,TENM3,OPCML,EPHA6,NLGN1,ABL1,PTPRG,GRID2,LRP2,SEMA6D,FAT3,NTNG1,MTMR2,LRP12,VCL,RAPGEF2,UNC5D,ULK2,NRXN3,RIMS2,KCNQ3,GFRA1,BMPR1B,SEMA5A,ATP8A2,MAP4K4,CTNNA2,ADGRV1,RELN,AUTS2,ROR1,CYFIP2,ADAMTSL1,TNR,CRB1,USH1C,CNTN6,APP,PDLIM5,NYAP2,VPS13B,ADGRB1,CCDC88A,SPOCK1*
GO:002305	signaling	8.34794072	*FSTL1,ERBB4,PDE6A,APBB2,FYB2,MACF1,PA*

2		1568393e-21	*K1,CTNND2,TRIO,MITF,EPN2,NETO2,PDE1C,EVC,NRP1,TEAD1,NFIA,CDH13,RC3H1,RPS6KA5,EPS8,TAOK3,OR4C46,ABL2,PRKCB,ARHGEF7,UTRN,GPC5,MAML2,ARHGEF12,SAMD12,SLC8A1,LNPEP,ANKRD6,RERG,HTR2C,DYSF,STAU2,CACNA1E,CNTN4,USP14,CNKSR3,LDLRAD4,SEMA3A,SLC8A3,RFX3,MGAT5,STARD13,LRRC4C,ALCAM,ERLIN2,LIMD1,PIK3C3,DCLK1,MAGI3,CDC42EP3,FGD4,GRIK2,SNX25,IGSF11,RGL1,CCDC186,MBD5,KIF16B,NCAM1,CLSTN2,SIPA1L2,RCAN2,ADGRL2,GNG12,PAPPA,ITGA8,GRIK4,MYO10,PJA2,ASB3,TAFA4,GABRG1,TOM1L2,TTC21B,NEK6,SLC39A12,MOB1B,ARHGAP28,DISC1,SV2B,RIC3,RALGPS1,ARHGAP42,EYA1,KSR1,RORB,GPR158,GABRB1,ANKS1B,APBA2,EDAR,FRMD6,RGS20,FYN,SH3KBP1,NALCN,CREBBP,SLC1A2,CADPS,DLGAP1,SLIT3,RGS7,FHIT,GRIN2B,SRGAP2,STK32A,GRIA1,TRABD2B,TAFA5,SPRED1,MYO5A,SPEN,FGF12,DGKB,CACNG2,GARNL3,BTBD9,CRIM1,NOTCH2,FLNB,MADD,ESR1,GABRG3,NFAT5,TMOD2,PLCXD3,PDE4D,TJP1,ARHGAP26,ERC2,PTPRT,PRKACB,GNG2,TRHDE,RIMS1,HIPK3,WSB1,FCHSD2,SGMS1,LAMA1,JCAD,GLI3,DOCK4,MAST4,GAST,ITPR2,NRXN1,NTRK3,FBN1,WWOX,WDPCP,MAGI1,MOSMO,RAPGEF4,CNKSR2,NEK10,PIAS1,PRICKLE2,HMGA2,HHAT,GRID1,PARP8,SEL1L,SLC4A10,PHLPP1,SYN2,MYO1E,ZDHHC17,PKN2,ITGA4,GSG1L,ROCK1,DCDC1,DOCK1,CACNA1C,ARHGAP31,UBR1,RXRG,PRKAA2,ARHGEF11,AMPH,SORCS1,COL4A2,SYT1,BBS2,GRB10,ARHGAP32,PRKN,SIAH2,LATS2,EXOC4,DOCK9,DLG2,PPP1R12B,RORA,PTPRD,CDC14B,TTR,PTPRN2,TG,HDAC4,ADGRB3,INPP4B,DGKG,MYO9A,FOXN3,NTRK2,PLCB1,MAPK1,LAMB1,SDCBP,GRIK1,CUL5,PRKG1,DSCAM,BIRC6,DGKI,NLK,EGFR,PPFIA2,BCR,MAGI2,AKAP6,LARGE1,ARHGEF28,PLCL1,MOK,TBC1D1,IQCJ-SCHIP1,SORCS3,SCN2A,RNF152,CNTN1,ZNRF3,GNAQ,DOCK10,TENM3,ITPKB,GRM5,MED1,ENPEP,EPHA6,ARAP2,AIMP1,NRG3,CPEB4,PID1,NLGN1,ABL1,PTPRG,NCOR1,OR9Q1,GRID2,ELMO1,LRP2,ERBIN,SEMA6D,PPM1L,CABIN1,MBTPS2,FER,PAK5,RYR2,IL17RA,FBXL17,MCC,PRLR,NFATC2,DST,GRIK3,CACNB2,STXBP4,NTNG1,GNG7,MTMR2,LRP12,MAPRE2,FGF10,NOS1AP,CADM1,SGCD,RC3H2,RIC8B,ATF6,STK32B,ZNF106,SEZ6L,ZDHHC21,VCAM1,RAPGEF2,RGS12,IFT81,SHANK2,ZMYND11,RAPGEF5,INSR,SRGAP3,PDK1,AKAP13,ILDR2,SYNJ1,KLF15,DENND4C,UNC5D,GABRG2,ARFGEF1,FAT1,PATJ,ITGA9,ENPP1,ULK2,UNC13C,NRXN3,RIMS2,RHPN2,PPM1F,KCNQ3,DENND2B,DLGAP2,GFRA1,ATRNL1,GPR156,BMPR1B,NSG1,MAPKBP1,CPE,KCND2,FCRLA,SEMA5A,KITLG,PYGO1,SV2C,ARHGAP24,GRIA4,MAP4K4,CNIH3,DOCK3,PRAME,APC,GPC6,DNER,ADGRV1,RELN,AKAP11,ANXA4,AUTS2,ROR1,PPP2R3A,BMP2*

			K,SNAI2,CYFIP2,NDFIP1,TNR,DTNA,CELF4,AKT3,CRB1,VAV3,IL1R1,BCAS3,PRKCA,INPP5A,USP8,CNTN6,FMN2,ERC1,APP,RPS6KA3,SMAD5,SLC6A3,PSD3,ALPK2,ASB2,EYA2,MAPK8IP1,ADGRB1,CCDC88A,GHRH,RAP1GDS1
GO:0031175	neuron projection development	3.3267792803339874e-19	*MACF1,PAK1,CTNND2,TRIO,NRP1,FRY,RPS6KA5,TAOK3,ABL2,SLC23A2,STAU2,CNTN4,AFG3L2,SEMA3A,LRRC4C,ALCAM,DCLK1,SDC2,NCAM1,PHACTR1,SLC39A12,DISC1,HECW2,ASAP1,FYN,CSMD3,B4GALT6,SLIT3,SRGAP2,ARSB,NEGR1,NOTCH2,TOX,PCDH15,SRGAP2C,RIMS1,LAMA1,GLI3,NRXN1,NTRK3,TSPAN2,RERE,ATL1,ZDHHC17,ITGA4,ROCK1,HERC1,DNM3,SYT1,ARHGAP32,PRKN,PTPRD,ADGRB3,DGKG,MYO9A,NTRK2,LAMB1,ATP9A,PRKG1,DSCAM,NFIB,DIP2B,PPFIA2,NCAM2,MAGI2,ARHGEF28,FUT9,CNTN1,DOCK10,TENM3,EPHA6,NLGN1,ABL1,PTPRG,GRID2,LRP2,SEMA6D,FAT3,NTNG1,LRP12,VCL,RAPGEF2,UNC5D,ULK2,NRXN3,RIMS2,GFRA1,BMPR1B,SEMA5A,ATP8A2,MAP4K4,CTNNA2,ADGRV1,RELN,AUTS2,ROR1,CYFIP2,ADAMTSL1,TNR,USH1C,CNTN6,APP,PDLIM5,NYAP2,VPS13B,ADGRB1,CCDC88A,SPOCK1*
GO:0120036	plasma membrane bounded cell projection organization	4.508007743591528e-19	*MACF1,PAK1,CTNND2,TRIO,SYNE2,MYO3B,NRP1,FRY,CDH13,RPS6KA5,EPS8,TAOK3,ABL2,SLC23A2,ARHGEF7,STAU2,CNTN4,AFG3L2,SEMA3A,RFX3,LRRC4C,ALCAM,DCLK1,CEP120,CDC42EP3,FGD4,SDC2,NCAM1,TANC1,MYO10,PHACTR1,TTC21B,ABCD2,SLC39A12,DISC1,HECW2,ASAP1,FYN,CSMD3,B4GALT6,SLIT3,GRIN2B,SRGAP2,ARSB,NEGR1,TPM1,NOTCH2,TOX,PCDH15,SRGAP2C,RIMS1,PARVB,LAMA1,GLI3,NRXN1,NTRK3,WASF3,HYDIN,TSPAN2,WDPCP,RERE,ATL1,ZDHHC17,ITGA4,ROCK1,HERC1,CFAP70,DNM3,SYT1,BBS2,ARHGAP32,PRKN,PTPRD,CDC14B,BBS9,ADGRB3,DGKG,MYO9A,MYO3A,NTRK2,LAMB1,SDCBP,ATP9A,PRKG1,DSCAM,NFIB,DIP2B,PPFIA2,NCAM2,MAGI2,RTTN,ARHGEF28,FUT9,CNTN1,DOCK10,TENM3,EPHA6,NLGN1,ABL1,PTPRG,GRID2,LRP2,SEMA6D,FER,FAT3,NTNG1,MTMR2,LRP12,VCL,RAPGEF2,IFT81,INSR,UNC5D,ULK2,NRXN3,RIMS2,GFRA1,BMPR1B,SEMA5A,ATP8A2,ARHGAP24,MAP4K4,CTNNA2,APC,ADGRV1,RELN,AUTS2,ROR1,CYFIP2,ADAMTSL1,TNR,VAV3,USH1C,DNAH5,CNTN6,APP,PDLIM5,NYAP2,VPS13B,ADGRB1,CCDC88A,SPOCK1*
GO:0007268	chemical synaptic transmission	5.573113748814375e-19	*TRIO,NETO2,PRKCB,HTR2C,DYSF,STAU2,CACNA1E,CNTN4,USP14,SLC8A3,LRRC4C,GRIK2,IGSF11,CLSTN2,GRIK4,GABRG1,DISC1,SV2B,RIC3,GABRB1,APBA2,FYN,NALCN,SLC1A2,CADPS,DLGAP1,GRIN2B,GRIA1,FGF12,DGKB,CACNG2,BTBD9,GABRG3,TMOD2,ERC2,RIMS1,FCHSD2,NRXN1,GRID1,SLC4A10,SYN2,AMPH,SYT1,PRKN,EXOC4,DLG2,PTPRD,PTPRN2,NTRK2,PLCB1,MAPK1,SDCBP,GRIK1,DGKI,PPFIA2,BCR,LARGE1,PLCL1,SORCS3,GRM5,NRG3,NLGN1,ABL1,GRID2,GRIK3,CACNB2,NTNG1,MTMR2,RAPGEF2,SHANK2,SYNJ1,GABRG2,UNC13C*

			,NRXN3,RIMS2,KCNQ3,DLGAP2,NSG1,KCND2,SV2C,GRIA4,RELN,TNR,DTNA,CELF4,USP8,ERC1,APP,RPS6KA3,SLC6A3,ADGRB1
GO:0098916	anterograde trans-synaptic signaling	5.573113748814375e-19	TRIO,NETO2,PRKCB,HTR2C,DYSF,STAU2,CACNA1E,CNTN4,USP14,SLC8A3,LRRC4C,GRIK2,IGSF11,CLSTN2,GRIK4,GABRG1,DISC1,SV2B,RIC3,GABRB1,APBA2,FYN,NALCN,SLC1A2,CADPS,DLGAP1,GRIN2B,GRIA1,FGF12,DGKB,CACNG2,BTBD9,GABRG3,TMOD2,ERC2,RIMS1,FCHSD2,NRXN1,GRID1,SLC4A10,SYN2,AMPH,SYT1,PRKN,EXOC4,DLG2,PTPRD,PTPRN2,NTRK2,PLCB1,MAPK1,SDCBP,GRIK1,DGKI,PPFIA2,BCR,LARGE1,PLCL1,SORCS3,GRM5,NRG3,NLGN1,ABL1,GRID2,GRIK3,CACNB2,NTNG1,MTMR2,RAPGEF2,SHANK2,SYNJ1,GABRG2,UNC13C,NRXN3,RIMS2,KCNQ3,DLGAP2,NSG1,KCND2,SV2C,GRIA4,RELN,TNR,DTNA,CELF4,USP8,ERC1,APP,RPS6KA3,SLC6A3,ADGRB1
GO:0030030	cell projection organization	6.525686749666561e-19	MACF1,PAK1,CTNND2,TRIO,SYNE2,MYO3B,NRP1,FRY,CDH13,RPS6KA5,EPS8,TAOK3,ABL2,SLC23A2,ARHGEF7,STAU2,CNTN4,AFG3L2,SEMA3A,RFX3,LRRC4C,ALCAM,DCLK1,CEP120,CDC42EP3,FGD4,SDC2,NCAM1,TANC1,ITGA8,MYO10,PHACTR1,TTC21B,ABCD2,SLC39A12,DISC1,HECW2,ASAP1,FYN,CSMD3,B4GALT6,SLIT3,GRIN2B,SRGAP2,ARSB,NEGR1,TPM1,NOTCH2,TOX,PCDH15,SRGAP2C,RIMS1,PARVB,LAMA1,GLI3,NRXN1,NTRK3,WASF3,HYDIN,TSPAN2,WDPCP,RERE,ATL1,ZDHHC17,PKN2,ITGA4,ROCK1,HERC1,CFAP70,DNM3,SYT1,BBS2,ARHGAP32,PRKN,PTPRD,CDC14B,BBS9,ADGRB3,DGKG,MYO9A,MYO3A,NTRK2,LAMB1,SDCBP,ATP9A,PRKG1,DSCAM,NFIB,DIP2B,PPFIA2,NCAM2,MAGI2,RTTN,ARHGEF28,FUT9,CNTN1,DOCK10,TENM3,EPHA6,NLGN1,ABL1,PTPRG,GRID2,LRP2,SEMA6D,FER,FAT3,NTNG1,MTMR2,LRP12,VCL,RAPGEF2,IFT81,INSR,UNC5D,ULK2,NRXN3,RIMS2,GFRA1,BMPR1B,SEMA5A,ATP8A2,ARHGAP24,MAP4K4,CTNNA2,APC,ADGRV1,RELN,AUTS2,ROR1,CYFIP2,ADAMTSL1,TNR,VAV3,USH1C,DNAH5,CNTN6,APP,PDLIM5,NYAP2,VPS13B,ADGRB1,CCDC88A,SPOCK1
GO:0099536	synaptic signaling	7.101684453992585e-19	TRIO,NETO2,PRKCB,UTRN,HTR2C,DYSF,STAU2,CACNA1E,CNTN4,USP14,SLC8A3,LRRC4C,GRIK2,IGSF11,CLSTN2,GRIK4,GABRG1,DISC1,SV2B,RIC3,GABRB1,APBA2,FYN,NALCN,SLC1A2,CADPS,DLGAP1,GRIN2B,GRIA1,FGF12,DGKB,CACNG2,BTBD9,GABRG3,TMOD2,ERC2,RIMS1,FCHSD2,NRXN1,GRID1,SLC4A10,SYN2,AMPH,SYT1,PRKN,EXOC4,DLG2,PTPRD,PTPRN2,NTRK2,PLCB1,MAPK1,SDCBP,GRIK1,DGKI,PPFIA2,BCR,LARGE1,PLCL1,SORCS3,GNAQ,GRM5,NRG3,NLGN1,ABL1,GRID2,GRIK3,CACNB2,NTNG1,MTMR2,RAPGEF2,SHANK2,SYNJ1,GABRG2,UNC13C,NRXN3,RIMS2,KCNQ3,DLGAP2,NSG1,KCND2,SV2C,GRIA4,RELN,TNR,DTNA,CELF4,USP8,ERC1,APP,RPS6KA3,SLC6A3,ADGRB1
GO:000090	cell	8.95092204	MACF1,PAK1,CTNND2,TRIO,NRP1,NFIA,FRY,

2	morphogenesis	9903835e-19	*CDH13,RPS6KA5,EPS8,TAOK3,SLC23A2,ARHGEF7,STAU2,CNTN4,AFG3L2,SEMA3A,LRRC4C,ALCAM,LIMD1,DCLK1,CDC42EP3,FGD4,SDC2,NCAM1,MYO10,FAM171A1,ATP10A,PHACTR1,SLC39A12,DISC1,HECW2,FRMD6,FYN,SH3KBP1,B4GALT6,SLIT3,SRGAP2,TPM1,NOTCH2,FLNB,PCDH15,RIMS1,PARVB,GLI3,NRXN1,WASF3,WDPCP,CDH20,RERE,ATL1,ZDHHC17,ITGA4,ROCK1,DNM3,SYT1,ARHGAP32,PRKN,LATS2,PTPRD,ADGRB3,MYO9A,NTRK2,ATP9A,PRKG1,DSCAM,NFIB,DIP2B,EGFR,PPFIA2,PTPRQ,ARHGEF28,CNTN1,DOCK10,MED1,EPHA6,NLGN1,ABL1,LRP2,SEMA6D,NTNG1,VCL,RAPGEF2,CDH7,UNC5D,FAT1,ULK2,NRXN3,RIMS2,BMPR1B,SEMA5A,ATP8A2,MAP4K4,CTNNA2,RELN,AUTS2,CYFIP2,ADAMTSL1,TNR,CRB1,CNTN6,APP,PDLIM5,NYAP2,ADGRB1*
GO:0099537	trans-synaptic signaling	9.74766580184756e-19	*TRIO,NETO2,PRKCB,HTR2C,DYSF,STAU2,CACNA1E,CNTN4,USP14,SLC8A3,LRRC4C,GRIK2,IGSF11,CLSTN2,GRIK4,GABRG1,DISC1,SV2B,RIC3,GABRB1,APBA2,FYN,NALCN,SLC1A2,CADPS,DLGAP1,GRIN2B,GRIA1,FGF12,DGKB,CACNG2,BTBD9,GABRG3,TMOD2,ERC2,RIMS1,FCHSD2,NRXN1,GRID1,SLC4A10,SYN2,AMPH,SYT1,PRKN,EXOC4,DLG2,PTPRD,PTPRN2,NTRK2,PLCB1,MAPK1,SDCBP,GRIK1,DGKI,PPFIA2,BCR,LARGE1,PLCL1,SORCS3,GRM5,NRG3,NLGN1,ABL1,GRID2,GRIK3,CACNB2,NTNG1,MTMR2,RAPGEF2,SHANK2,SYNJ1,GABRG2,UNC13C,NRXN3,RIMS2,KCNQ3,DLGAP2,NSG1,KCND2,SV2C,GRIA4,RELN,TNR,DTNA,CELF4,USP8,ERC1,APP,RPS6KA3,SLC6A3,ADGRB1*
GO:0065007	biological regulation	1.3859603372111618e-17	*FSTL1,HPSE2,SLMAP,SAMD4A,ERBB4,KCNMA1,PDE6A,APBB2,KLHL13,FYB2,MACF1,PAK1,CTNND2,SLC6A11,SCAF8,ATP9B,TRIO,MITF,RTN1,SETD2,KIRREL1,AMBRA1,SYNE2,EPN2,NETO2,PDE1C,ZNF208,MYO3B,EVC,MED13L,NRP1,TEAD1,NFIA,FRY,CDH13,ZNF718,RC3H1,RPS6KA5,LHFPL2,EPS8,TAOK3,ZSCAN30,LRFN5,ZNF66,OR4C46,ABL2,PRKCB,SLC23A2,ARHGEF7,UTRN,GPC5,ZNF704,MAML2,ARHGEF12,SAMD12,SLC8A1,LYPLA1,DACH1,LNPEP,PRR16,CDK12,SLC2A13,ANKRD6,RERG,HTR2C,ZNF397,KCNC1,DYSF,STAU2,NEK4,CACNA1E,SIAH3,FYCO1,LPGAT1,CNTN4,CTIF,TBC1D5,USP14,AFG3L2,CNKSR3,FRMD5,LDLRAD4,SEMA3A,TRIM2,PLIN2,SLC8A3,RFX3,MGAT5,RBFOX1,STARD13,SLC40A1,LRRC4C,ALCAM,MALRD1,ERLIN2,SLCO3A1,ATP11C,LIMD1,PIK3C3,DCLK1,DNAH11,SLC9C1,ADAMTS9,MAGI3,NIPBL,CEP120,CDC42EP3,CHD6,FGD4,GRIK2,SNX25,IGSF11,RGL1,CCDC186,GSAP,MBD5,KIF16B,NKAIN3,SDC2,NCAM1,CLSTN2,TCF12,SIPA1L2,CCNG2,RCAN2,ADGRL2,GNG12,TANC1,CORO2B,PAPPA,EVI5,ITGA8,SYCP1,VPS41,GRIK4,ZBTB20,MYO10,ZNF407,PJA2,ASB3,TCF4,TAFA4,GABRG1,TSHZ2,TOM1L2,IREB2,PBX1,FAM171A1,ATP10A,PHACTR1,BICRAL,TTC21B,NEK6,ABCD2,SLC39A12,MOB1B,ARHGAP28*

			,DISC1,CLTCL1,SV2B,HECW2,RIC3,FMN1,RALGPS1,ARHGAP42,PEAK1,EYA1,KSR1,RORB,PIEZO2,GPR158,ZNF236,GABRB1,SVIL,ANKS1B,APBA2,ASAP1,SGSM1,FRMPD4,EDAR,ST8SIA1,ATP2B2,ANKRD26,FRMD6,RGS20,FYN,SH3KBP1,AGO2,NALCN,CREBBP,SLC1A2,ZBTB16,PRMT8,CADPS,CSMD3,DLGAP1,SLIT3,RGS7,FHIT,GRIN2B,ANTXR1,SRGAP2,STK32A,ARSB,GRIA1,TRABD2B,TAFA5,NEGR1,SPRED1,MYO5A,SPEN,NAV2,FGF12,DGKB,CACNG2,GARNL3,KANSL1,BTBD9,TPM1,COL19A1,EBF1,CRIM1,NOTCH2,FLNB,MADD,TOX,CLDN10,ESR1,GABRG3,NFAT5,SRGAP2C,ABCB5,TMOD2,PLCXD3,PDE4D,TJP1,ARHGAP26,ERC2,PTPRT,PRKACB,GNG2,TRHDE,RIMS1,HIPK3,WSB1,TMEM225,FCHSD2,SGMS1,PARVB,LAMA1,JCAD,GLI3,DOCK4,MAST4,GAST,ITPR2,BRINP1,NRXN1,FRMD4A,SUPT16H,NTRK3,BCL2L13,JAZF1,FBN1,WWOX,WASF3,LONP2,ARID1B,MTUS1,CPQ,WDPCP,MAGI1,PRRC1,MOSMO,RAPGEF4,CNKSR2,NEK10,ZNF74,ANKRD31,PIAS1,ZNF521,PBX3,PRICKLE2,DPP6,HMGA2,HHAT,CTTNBP2,FHOD3,GRID1,PARP8,ZNF287,CREB5,TNRC6B,RERE,CACNA2D3,SEL1L,ZNF608,SLC4A10,PHLPP1,SYN2,MYO1E,ZDHHC17,PKN2,ITGA4,ABCC9,GSG1L,ROCK1,HERC1,DCDC1,DOCK1,CACNA1C,GLIS1,ARHGAP31,UBR1,RXRG,PRKAA2,ARHGEF11,AMPH,SORCS1,COL4A2,DNM3,SYT1,BBS2,GRB10,ARHGAP32,ASXL3,PRKN,DPF3,FOXP2,EDIL3,SIAH2,LATS2,EXOC4,DOCK9,DLG2,PPP1R12B,SACS,RORA,PTPRD,CDC14B,CDYL2,TTR,PTPRN2,RANBP3L,TG,HDAC4,ADGRB3,INPP4B,DGKG,ZNF717,MYO9A,MYO3A,FOXN3,NTRK2,PLCB1,HIVEP2,MAPK1,LAMB1,SDCBP,ATP9A,BPTF,TASOR2,GRIK1,CUL5,SOX5,PRKG1,DSCAM,BIRC6,DGKI,NFIB,DIP2B,TUT4,NLK,SPOP,NBEA,EGFR,PPFIA2,STXBP6,MGA,MXI1,BCR,TTC28,MAGI2,NELL1,AKAP6,LARGE1,MBNL1,STX12,FANCM,ARHGEF28,PLCL1,MELTF,FUT9,MYT1L,MOK,ANKFY1,TBC1D1,IQCJ-SCHIP1,SORCS3,PDP2,SLC12A1,SCN2A,RNF152,CNTN1,ZNRF3,POU6F2,GNAQ,ZNF449,DOCK10,TENM3,ITPKB,GRM5,MED1,ENPEP,EPHA6,GLIS3,SPIRE1,ARAP2,AIMP1,LDB2,NECAB1,SNX30,NRG3,CPEB4,ATP8A1,PID1,NLGN1,ABL1,PTPRG,NCOR1,JARID2,PRDM10,OR9Q1,EBF2,JAM2,GRID2,ELMO1,LRP2,ERBIN,SEMA6D,PPM1L,CABIN1,ZNF573,MBTPS2,SMOC1,FER,PAK5,SETDB2,RYR2,ELF2,IL17RA,LDLRAD3,FBXL17,MCC,SUSD4,PRLR,ZNF160,ZNF280B,NFATC2,DST,FAT3,GRIK3,CACNB2,STXBP4,NTNG1,MED15,MTPN,VSTM2A,GNG7,MTMR2,KCNH5,LRP12,MAPRE2,FGF10,ZNF169,KIF13A,NOS1AP,SOX6,CADM1,SGCD,RC3H2,PDE4DIP,RIC8B,MPDZ,YLPM1,ATF6,SP3,STK32B,VCL,ZNF106,SEZ6L,ZDHHC21,XKR6,VCAM1,RAPGEF2,RGS12,IFT81,SHANK2,ZMYND11,RAPGEF5,INSR,SRGAP3,PDK1,AKAP13,WDR41,ILDR2,SYNJ1,KLF15,DENND4C,UNC5D,ATXN1,KDM1

			B,GABRG2,ARFGEF1,FAT1,PATJ,ITGA9,ENPP1,ULK2,UNC13C,ZNF462,NRXN3,RIMS2,RHPN2,PPM1F,KCNQ3,SCN8A,ZFHX3,DENND2B,EGLN3,DLGAP2,GFRA1,ATRNL1,GPR156,BMPR1B,NSG1,MAPKBP1,CPE,KCND2,FCRLA,SEMA5A,CABLES1,ATP8A2,SLC24A3,KITLG,PYGO1,SV2C,ARHGAP24,GRIA4,MAP4K4,CNIH3,DOCK3,CTNNA2,PRAME,APC,APLF,GPC6,DNER,ABCA5,RFX7,ADGRV1,RELN,AKAP11,VPS13C,BNC2,ANXA4,KCNJ15,AUTS2,TBC1D9,ROR1,PPP2R3A,MTMR3,BMP2K,SNAI2,CYFIP2,SLC4A4,NDFIP1,TNR,PTPN13,DTNA,SNAP91,OCA2,CELF4,AKT3,CRB1,VAV3,IL1R1,BCAS3,NPAS3,PRKCA,HEPACAM,KMT2C,INPP5A,USP8,USH1C,MRTFB,ZNF678,CNTN6,KCNS3,ENPP3,FMN2,ERC1,APP,PDLIM5,RPS6KA3,BTAF1,SMAD5,SLC6A3,DNAJC13,PSD3,ALPK2,ABCA13,ASB2,EYA2,MAPK8IP1,ADGRB1,CCDC88A,GHRH,ARNT2,RAP1GDS1,AFF3,SPOCK1
GO:0050789	regulation of biological process	3.2617644812432246e-17	FSTL1,HPSE2,SLMAP,SAMD4A,ERBB4,KCNMA1,PDE6A,APBB2,KLHL13,FYB2,MACF1,PAK1,CTNND2,SCAF8,TRIO,MITF,RTN1,SETD2,KIRREL1,AMBRA1,SYNE2,EPN2,NETO2,PDE1C,ZNF208,MYO3B,EVC,MED13L,NRP1,TEAD1,NFIA,FRY,CDH13,ZNF718,RC3H1,RPS6KA5,LHFPL2,EPS8,TAOK3,ZSCAN30,LRFN5,ZNF66,OR4C46,ABL2,PRKCB,SLC23A2,ARHGEF7,UTRN,GPC5,ZNF704,MAML2,ARHGEF12,SAMD12,SLC8A1,LYPLA1,DACH1,LNPEP,PRR16,CDK12,SLC2A13,ANKRD6,RERG,HTR2C,ZNF397,KCNC1,DYSF,STAU2,NEK4,CACNA1E,SIAH3,FYCO1,LPGAT1,CNTN4,CTIF,TBC1D5,USP14,AFG3L2,CNKSR3,FRMD5,LDLRAD4,SEMA3A,TRIM2,PLIN2,SLC8A3,RFX3,MGAT5,RBFOX1,STARD13,SLC40A1,LRRC4C,ALCAM,MALRD1,ERLIN2,SLCO3A1,ATP11C,LIMD1,PIK3C3,DCLK1,DNAH11,ADAMTS9,MAGI3,NIPBL,CEP120,CDC42EP3,CHD6,FGD4,GRIK2,SNX25,IGSF11,RGL1,CCDC186,GSAP,MBD5,KIF16B,NKAIN3,SDC2,NCAM1,CLSTN2,TCF12,SIPA1L2,CCNG2,RCAN2,ADGRL2,GNG12,TANC1,CORO2B,PAPPA,ITGA8,SYCP1,VPS41,GRIK4,ZBTB20,MYO10,ZNF407,PJA2,ASB3,TCF4,TAFA4,GABRG1,TSHZ2,TOM1L2,IREB2,PBX1,FAM171A1,ATP10A,PHACTR1,BICRAL,TTC21B,NEK6,ABCD2,SLC39A12,MOB1B,ARHGAP28,DISC1,CLTCL1,SV2B,HECW2,RIC3,FMN1,RALGPS1,ARHGAP42,PEAK1,EYA1,KSR1,RORB,GPR158,ZNF236,GABRB1,SVIL,ANKS1B,APBA2,ASAP1,FRMPD4,EDAR,ST8SIA1,ATP2B2,ANKRD26,FRMD6,RGS20,FYN,SH3KBP1,AGO2,NALCN,CREBBP,SLC1A2,ZBTB16,CADPS,CSMD3,DLGAP1,SLIT3,RGS7,FHIT,GRIN2B,ANTXR1,SRGAP2,STK32A,ARSB,GRIA1,TRABD2B,TAFA5,NEGR1,SPRED1,MYO5A,SPEN,FGF12,DGKB,CACNG2,GARNL3,KANSL1,BTBD9,TPM1,COL19A1,EBF1,CRIM1,NOTCH2,FLNB,MADD,TOX,CLDN10,ESR1,GABRG3,NFAT5,SRGAP2C,TMOD2,PLCXD3,PDE4D,TJP1,ARHGAP26,ERC2,PTPRT,PRKACB,GNG2,TRHDE,RIMS1,HI

			PK3,WSB1,TMEM225,FCHSD2,SGMS1,PARVB,LAMA1,JCAD,GLI3,DOCK4,MAST4,GAST,ITPR2,BRINP1,NRXN1,FRMD4A,SUPT16H,NTRK3,BCL2L13,JAZF1,FBN1,WWOX,WASF3,LONP2,ARID1B,MTUS1,WDPCP,MAGI1,PRRC1,MOSMO,RAPGEF4,CNKSR2,NEK10,ZNF74,ANKRD31,PIAS1,ZNF521,PBX3,PRICKLE2,DPP6,HMGA2,HHAT,CTTNBP2,FHOD3,GRID1,PARP8,ZNF287,CREB5,TNRC6B,RERE,CACNA2D3,SEL1L,ZNF608,SLC4A10,PHLPP1,SYN2,MYO1E,ZDHHC17,PKN2,ITGA4,ABCC9,GSG1L,ROCK1,HERC1,DCDC1,DOCK1,CACNA1C,GLIS1,ARHGAP31,UBR1,RXRG,PRKAA2,ARHGEF11,AMPH,SORCS1,COL4A2,DNM3,SYT1,BBS2,GRB10,ARHGAP32,ASXL3,PRKN,DPF3,FOXP2,EDIL3,SIAH2,LATS2,EXOC4,DOCK9,DLG2,PPP1R12B,SACS,RORA,PTPRD,CDC14B,CDYL2,TTR,PTPRN2,RANBP3L,TG,HDAC4,ADGRB3,INPP4B,DGKG,ZNF717,MYO9A,MYO3A,FOXN3,NTRK2,PLCB1,HIVEP2,MAPK1,LAMB1,SDCBP,ATP9A,BPTF,TASOR2,GRIK1,CUL5,SOX5,PRKG1,DSCAM,BIRC6,DGKI,NFIB,DIP2B,TUT4,NLK,SPOP,NBEA,EGFR,PPFIA2,STXBP6,MGA,MXI1,BCR,TTC28,MAGI2,NELL1,AKAP6,LARGE1,MBNL1,FANCM,ARHGEF28,PLCL1,MELTF,FUT9,MYT1L,MOK,ANKFY1,TBC1D1,IQCJ-SCHIP1,SORCS3,SCN2A,RNF152,CNTN1,ZNRF3,POU6F2,GNAQ,ZNF449,DOCK10,TENM3,ITPKB,GRM5,MED1,ENPEP,EPHA6,GLIS3,SPIRE1,ARAP2,AIMP1,LDB2,NECAB1,SNX30,NRG3,CPEB4,ATP8A1,PID1,NLGN1,ABL1,PTPRG,NCOR1,JARID2,PRDM10,OR9Q1,EBF2,JAM2,GRID2,ELMO1,LRP2,ERBIN,SEMA6D,PPM1L,CABIN1,ZNF573,MBTPS2,SMOC1,FER,PAK5,SETDB2,RYR2,ELF2,IL17RA,LDLRAD3,FBXL17,MCC,SUSD4,PRLR,ZNF160,ZNF280B,NFATC2,DST,FAT3,GRIK3,CACNB2,STXBP4,NTNG1,MED15,MTPN,VSTM2A,GNG7,MTMR2,KCNH5,LRP12,MAPRE2,FGF10,ZNF169,KIF13A,NOS1AP,SOX6,CADM1,SGCD,RC3H2,PDE4DIP,RIC8B,MPDZ,YLPM1,ATF6,SP3,STK32B,VCL,ZNF106,SEZ6L,ZDHHC21,VCAM1,RAPGEF2,RGS12,IFT81,SHANK2,ZMYND11,RAPGEF5,INSR,SRGAP3,PDK1,AKAP13,WDR41,ILDR2,SYNJ1,KLF15,DENND4C,UNC5D,ATXN1,KDM1B,GABRG2,ARFGEF1,FAT1,PATJ,ITGA9,ENPP1,ULK2,UNC13C,ZNF462,NRXN3,RIMS2,RHPN2,PPM1F,KCNQ3,SCN8A,ZFHX3,DENND2B,EGLN3,DLGAP2,GFRA1,ATRNL1,GPR156,BMPR1B,NSG1,MAPKBP1,CPE,KCND2,FCRLA,SEMA5A,CABLES1,ATP8A2,SLC24A3,KITLG,PYGO1,SV2C,ARHGAP24,GRIA4,MAP4K4,CNIH3,DOCK3,CTNNA2,PRAME,APC,APLF,GPC6,DNER,ABCA5,RFX7,ADGRV1,RELN,AKAP11,VPS13C,BNC2,ANXA4,KCNJ15,AUTS2,ROR1,PPP2R3A,MTMR3,BMP2K,SNAI2,CYFIP2,SLC4A4,NDFIP1,TNR,PTPN13,DTNA,SNAP91,CELF4,AKT3,CRB1,VAV3,IL1R1,BCAS3,NPAS3,PRKCA,HEPACAM,KMT2C,INPP5A,USP8,USH1C,MRTFB,ZNF678,CNTN6,KCNS3,ENPP3,FM

			N2,ERC1,APP,PDLIM5,RPS6KA3,BTAF1,SMAD5,SLC6A3,DNAJC13,PSD3,ALPK2,ABCA13,ASB2,EYA2,MAPK8IP1,ADGRB1,CCDC88A,GHRH,ARNT2,RAP1GDS1,AFF3,SPOCK1
GO:0050808	synapse organization	7.310219132682761e-17	*ERBB4,APBB2,CTNND2,NRP1,NFIA,RPS6KA5,LRFN5,STAU2,AFG3L2,SLC8A3,LRRC4C,PDZRN3,CLSTN2,ADGRL2,TANC1,DISC1,ASAP1,FRMPD4,FYN,GRIN2B,SRGAP2,GRIA1,NEGR1,DGKB,CACNG2,SRGAP2C,ERC2,PTPRT,NRXN1,WASF3,CNKSR2,CTTNBP2,GRID1,ROCK1,DNM3,PTPRD,ADGRB3,NTRK2,SDCBP,DSCAM,NBEA,PPFIA2,LARGE1,DOCK10,GRM5,NRG3,NLGN1,ABL1,GRID2,CACNB2,NTNG1,MTMR2,NOS1AP,SEZ6L,SHANK2,INSR,GABRG2,UNC13C,NRXN3,CTNNA2,DNER,RELN,TNR,PTPN13,ERC1,APP,PDLIM5,ADGRB1*
GO:0007267	cell-cell signaling	9.824374753861468e-17	*MACF1,CTNND2,TRIO,MITF,NETO2,NRP1,PRKCB,ARHGEF7,UTRN,GPC5,LNPEP,ANKRD6,HTR2C,DYSF,STAU2,CACNA1E,CNTN4,USP14,SLC8A3,RFX3,LRRC4C,LIMD1,GRIK2,IGSF11,CCDC186,CLSTN2,GRIK4,GABRG1,TTC21B,DISC1,SV2B,RIC3,GABRB1,APBA2,FYN,SH3KBP1,NALCN,SLC1A2,CADPS,DLGAP1,GRIN2B,GRIA1,TRABD2B,FGF12,DGKB,CACNG2,BTBD9,GABRG3,TMOD2,ERC2,TRHDE,RIMS1,FCHSD2,GLI3,NRXN1,WWOX,RAPGEF4,PRICKLE2,HMGA2,GRID1,SLC4A10,SYN2,CACNA1C,PRKAA2,AMPH,SYT1,GRB10,PRKN,SIAH2,LATS2,EXOC4,DLG2,PTPRD,PTPRN2,NTRK2,PLCB1,MAPK1,SDCBP,GRIK1,DGKI,NLK,EGFR,PPFIA2,BCR,MAGI2,LARGE1,PLCL1,TBC1D1,SORCS3,ZNRF3,GNAQ,GRM5,ENPEP,AIMP1,NRG3,NLGN1,ABL1,GRID2,RYR2,MCC,GRIK3,CACNB2,STXBP4,NTNG1,MTMR2,FGF10,RAPGEF2,SHANK2,ILDR2,SYNJ1,KLF15,GABRG2,FAT1,UNC13C,NRXN3,RIMS2,KCNQ3,DLGAP2,GPR156,NSG1,CPE,KCND2,SEMA5A,PYGO1,SV2C,GRIA4,MAP4K4,APC,GPC6,RELN,ROR1,PPP2R3A,SNAI2,TNR,DTNA,CELF4,CRB1,BCAS3,USP8,ERC1,APP,RPS6KA3,SLC6A3,ALPK2,ADGRB1,GHRH*
GO:0050794	regulation of cellular process	1.7454680421804708e-16	*FSTL1,HPSE2,SLMAP,SAMD4A,ERBB4,KCNMA1,PDE6A,APBB2,KLHL13,FYB2,MACF1,PAK1,CTNND2,SCAF8,TRIO,MITF,SETD2,KIRREL1,AMBRA1,SYNE2,EPN2,NETO2,PDE1C,ZNF208,MYO3B,EVC,MED13L,NRP1,TEAD1,NFIA,CDH13,ZNF718,RC3H1,RPS6KA5,EPS8,TAOK3,ZSCAN30,LRFN5,ZNF66,OR4C46,ABL2,PRKCB,SLC23A2,ARHGEF7,UTRN,GPC5,ZNF704,MAML2,ARHGEF12,SAMD12,SLC8A1,LYPLA1,DACH1,LNPEP,PRR16,CDK12,ANKRD6,RERG,HTR2C,ZNF397,KCNC1,DYSF,STAU2,NEK4,CACNA1E,SIAH3,FYCO1,LPGAT1,CNTN4,CTIF,TBC1D5,USP14,CNKSR3,FRMD5,LDLRAD4,SEMA3A,TRIM2,SLC8A3,RFX3,MGAT5,RBFOX1,STARD13,SLC40A1,LRRC4C,ALCAM,MALRD1,ERLIN2,SLCO3A1,ATP11C,LIMD1,PIK3C3,DCLK1,DNAH11,ADAMTS9,MAGI3,NIPBL,CEP120,CDC42EP3,CHD6,FGD4,GRIK2,SNX25,IGSF11,RGL1,MBD5,KIF16B,SDC2,NCAM1,CLSTN2,TCF12,SIPA1L2*

			,CCNG2,RCAN2,ADGRL2,GNG12,TANC1,CORO2B,PAPPA,ITGA8,SYCP1,VPS41,GRIK4,ZBTB20,MYO10,ZNF407,PJA2,ASB3,TCF4,TAFA4,GABRG1,TSHZ2,TOM1L2,IREB2,PBX1,ATP10A,PHACTR1,BICRAL,TTC21B,NEK6,ABCD2,SLC39A12,MOB1B,ARHGAP28,DISC1,CLTCL1,SV2B,HECW2,RIC3,FMN1,RALGPS1,ARHGAP42,PEAK1,EYA1,KSR1,RORB,GPR158,ZNF236,GABRB1,SVIL,ANKS1B,APBA2,ASAP1,FRMPD4,EDAR,ST8SIA1,ANKRD26,FRMD6,RGS20,FYN,SH3KBP1,AGO2,NALCN,CREBBP,SLC1A2,ZBTB16,CADPS,CSMD3,DLGAP1,SLIT3,RGS7,FHIT,GRIN2B,ANTXR1,SRGAP2,STK32A,ARSB,GRIA1,TRABD2B,TAFA5,NEGR1,SPRED1,MYO5A,SPEN,FGF12,DGKB,CACNG2,GARNL3,KANSL1,BTBD9,TPM1,COL19A1,EBF1,CRIM1,NOTCH2,FLNB,MADD,TOX,ESR1,GABRG3,NFAT5,SRGAP2C,TMOD2,PLCXD3,PDE4D,TJP1,ARHGAP26,ERC2,PTPRT,PRKACB,GNG2,TRHDE,RIMS1,HIPK3,WSB1,TMEM225,FCHSD2,SGMS1,LAMA1,JCAD,GLI3,DOCK4,MAST4,GAST,ITPR2,BRINP1,NRXN1,FRMD4A,SUPT16H,NTRK3,BCL2L13,JAZF1,FBN1,WWOX,WASF3,LONP2,ARID1B,MTUS1,WDPCP,MAGI1,PRRC1,MOSMO,RAPGEF4,CNKSR2,NEK10,ZNF74,ANKRD31,PIAS1,ZNF521,PBX3,PRICKLE2,DPP6,HMGA2,HHAT,CTTNBP2,FHOD3,GRID1,PARP8,ZNF287,CREB5,TNRC6B,RERE,CACNA2D3,SEL1L,ZNF608,SLC4A10,PHLPP1,MYO1E,ZDHHC17,PKN2,ITGA4,ABCC9,GSG1L,ROCK1,HERC1,DCDC1,DOCK1,CACNA1C,GLIS1,ARHGAP31,UBR1,RXRG,PRKAA2,ARHGEF11,SORCS1,COL4A2,DNM3,SYT1,BBS2,GRB10,ARHGAP32,ASXL3,PRKN,DPF3,FOXP2,EDIL3,SIAH2,LATS2,EXOC4,DOCK9,PPP1R12B,SACS,RORA,PTPRD,CDC14B,CDYL2,TTR,RANBP3L,TG,HDAC4,ADGRB3,INPP4B,DGKG,ZNF717,MYO9A,MYO3A,FOXN3,NTRK2,PLCB1,HIVEP2,MAPK1,LAMB1,SDCBP,ATP9A,BPTF,TASOR2,GRIK1,CUL5,SOX5,PRKG1,DSCAM,BIRC6,DGKI,NFIB,DIP2B,TUT4,NLK,NBEA,EGFR,PPFIA2,STXBP6,MGA,MXI1,BCR,TTC28,MAGI2,NELL1,AKAP6,LARGE1,MBNL1,ARHGEF28,PLCL1,MELTF,FUT9,MYT1L,MOK,ANKFY1,TBC1D1,IQCJ-SCHIP1,SORCS3,SCN2A,RNF152,CNTN1,ZNRF3,POU6F2,GNAQ,ZNF449,DOCK10,TENM3,ITPKB,GRM5,MED1,EPHA6,GLIS3,SPIRE1,ARAP2,AIMP1,LDB2,NECAB1,SNX30,NRG3,CPEB4,ATP8A1,PID1,NLGN1,ABL1,PTPRG,NCOR1,JARID2,PRDM10,OR9Q1,EBF2,JAM2,GRID2,ELMO1,LRP2,ERBIN,SEMA6D,PPM1L,CABIN1,ZNF573,MBTPS2,SMOC1,FER,PAK5,SETDB2,RYR2,ELF2,IL17RA,LDLRAD3,FBXL17,MCC,PRLR,ZNF160,ZNF280B,NFATC2,DST,FAT3,GRIK3,CACNB2,STXBP4,NTNG1,MED15,MTPN,VSTM2A,GNG7,MTMR2,KCNH5,LRP12,MAPRE2,FGF10,ZNF169,KIF13A,NOS1AP,SOX6,CADM1,SGCD,RC3H2,PDE4DIP,RIC8B,MPDZ,YLPM1,ATF6,SP3,STK32B,VCL,ZNF106,SEZ6L,ZDHHC21,VCAM1,RAPGEF2,RGS12,IFT81,SHANK2,ZMYND11

			,RAPGEF5,INSR,SRGAP3,PDK1,AKAP13,WDR41,ILDR2,SYNJ1,KLF15,DENND4C,UNC5D,ATXN1,KDM1B,GABRG2,ARFGEF1,PATJ,ITGA9,ENPP1,ULK2,UNC13C,ZNF462,NRXN3,RIMS2,RHPN2,PPM1F,KCNQ3,SCN8A,ZFHX3,DENND2B,EGLN3,DLGAP2,GFRA1,ATRNL1,GPR156,BMPR1B,NSG1,MAPKBP1,CPE,KCND2,FCRLA,SEMA5A,CABLES1,ATP8A2,SLC24A3,KITLG,PYGO1,SV2C,ARHGAP24,GRIA4,MAP4K4,CNIH3,DOCK3,CTNNA2,PRAME,APC,APLF,GPC6,DNER,ABCA5,RFX7,ADGRV1,RELN,AKAP11,VPS13C,BNC2,ANXA4,KCNJ15,AUTS2,ROR1,PPP2R3A,MTMR3,BMP2K,SNAI2,CYFIP2,SLC4A4,NDFIP1,TNR,PTPN13,DTNA,SNAP91,CELF4,AKT3,VAV3,IL1R1,BCAS3,NPAS3,PRKCA,HEPACAM,KMT2C,INPP5A,USP8,USH1C,MRTFB,ZNF678,CNTN6,KCNS3,ENPP3,FMN2,ERC1,APP,PDLIM5,RPS6KA3,BTAF1,SMAD5,SLC6A3,DNAJC13,PSD3,ALPK2,ABCA13,ASB2,EYA2,MAPK8IP1,ADGRB1,CCDC88A,GHRH,ARNT2,RAP1GDS1,AFF3,SPOCK1
GO:0010646	regulation of cell communication	2.2104092173504074e-16	FSTL1,ERBB4,MACF1,PAK1,CTNND2,TRIO,EPN2,NETO2,EVC,NRP1,CDH13,RC3H1,EPS8,TAOK3,ABL2,PRKCB,ARHGEF7,GPC5,ARHGEF12,SLC8A1,ANKRD6,HTR2C,DYSF,STAU2,CNTN4,CNKSR3,LDLRAD4,SEMA3A,SLC8A3,RFX3,MGAT5,STARD13,LRRC4C,LIMD1,FGD4,GRIK2,SNX25,IGSF11,MBD5,NCAM1,CLSTN2,SIPA1L2,ITGA8,GRIK4,PJA2,TAFA4,TTC21B,NEK6,ARHGAP28,DISC1,SV2B,RALGPS1,ARHGAP42,EYA1,KSR1,APBA2,EDAR,FRMD6,RGS20,FYN,NALCN,CREBBP,DLGAP1,SLIT3,RGS7,GRIN2B,SRGAP2,GRIA1,TRABD2B,SPRED1,FGF12,DGKB,CACNG2,GARNL3,BTBD9,CRIM1,NOTCH2,MADD,ESR1,NFAT5,TMOD2,PDE4D,ARHGAP26,ERC2,PTPRT,PRKACB,RIMS1,HIPK3,SGMS1,LAMA1,JCAD,GLI3,NRXN1,NTRK3,FBN1,WWOX,MOSMO,RAPGEF4,CNKSR2,NEK10,GRID1,SLC4A10,PHLPP1,ZDHHC17,GSG1L,ROCK1,ARHGAP31,UBR1,PRKAA2,ARHGEF11,SYT1,GRB10,ARHGAP32,PRKN,SIAH2,LATS2,RORA,PTPRD,DGKG,MYO9A,NTRK2,PLCB1,MAPK1,LAMB1,SDCBP,GRIK1,BIRC6,DGKI,NLK,EGFR,PPFIA2,BCR,MAGI2,AKAP6,LARGE1,ARHGEF28,PLCL1,TBC1D1,IQCJ-SCHIP1,SORCS3,RNF152,ZNRF3,GNAQ,ITPKB,GRM5,MED1,AIMP1,NRG3,PID1,NLGN1,ABL1,NCOR1,GRID2,LRP2,ERBIN,FER,PAK5,RYR2,FBXL17,MCC,PRLR,GRIK3,STXBP4,NTNG1,GNG7,MTMR2,MAPRE2,FGF10,NOS1AP,CADM1,RC3H2,RIC8B,ATF6,SEZ6L,RAPGEF2,RGS12,IFT81,SHANK2,ZMYND11,INSR,SRGAP3,AKAP13,KLF15,DENND4C,ARFGEF1,ENPP1,UNC13C,NRXN3,RIMS2,KCNQ3,DENND2B,DLGAP2,BMPR1B,NSG1,MAPKBP1,SEMA5A,KITLG,SV2C,ARHGAP24,GRIA4,MAP4K4,CNIH3,DOCK3,PRAME,APC,GPC6,ADGRV1,RELN,AUTS2,ROR1,PPP2R3A,BMP2K,SNAI2,CYFIP2,NDFIP1,TNR,CELF4,AKT3,VAV3,IL1R1,PRKCA,USP8,CNTN6,ER

			C1,APP,SMAD5,PSD3,ALPK2,EYA2,MAPK8IP1,ADGRB1,CCDC88A,GHRH,RAP1GDS1
GO:0048812	neuron projection morphogenesis	5.107570181310812e-16	*MACF1,PAK1,CTNND2,TRIO,NRP1,RPS6KA5,TAOK3,SLC23A2,STAU2,CNTN4,AFG3L2,SEMA3A,LRRC4C,ALCAM,DCLK1,SDC2,NCAM1,PHACTR1,SLC39A12,DISC1,HECW2,FYN,B4GALT6,SLIT3,SRGAP2,NOTCH2,RIMS1,GLI3,NRXN1,RERE,ATL1,ZDHHC17,ITGA4,ROCK1,DNM3,SYT1,ARHGAP32,PRKN,PTPRD,ADGRB3,MYO9A,NTRK2,ATP9A,PRKG1,DSCAM,NFIB,DIP2B,PPFIA2,ARHGEF28,CNTN1,DOCK10,EPHA6,NLGN1,ABL1,LRP2,SEMA6D,NTNG1,VCL,RAPGEF2,UNC5D,ULK2,NRXN3,RIMS2,BMPR1B,SEMA5A,ATP8A2,MAP4K4,CTNNA2,RELN,AUTS2,CYFIP2,ADAMTSL1,TNR,CNTN6,APP,PDLIM5,NYAP2,ADGRB1*
GO:0032989	cellular component morphogenesis	6.090797366495487e-16	*MACF1,PAK1,CTNND2,TRIO,NRP1,RPS6KA5,TAOK3,SLC23A2,ARHGEF7,STAU2,CNTN4,AFG3L2,SEMA3A,LRRC4C,ALCAM,DCLK1,SDC2,NCAM1,NEBL,PHACTR1,SLC39A12,DISC1,HECW2,FYN,B4GALT6,SLIT3,SRGAP2,TPM1,NOTCH2,TMOD2,RIMS1,GLI3,NRXN1,FHOD3,RERE,ATL1,ZDHHC17,ITGA4,ROCK1,DNM3,SYT1,ARHGAP32,PRKN,PTPRD,ADGRB3,MYO9A,NTRK2,ATP9A,PRKG1,DSCAM,NFIB,DIP2B,PPFIA2,ARHGEF28,CNTN1,DOCK10,EPHA6,PID1,NLGN1,ABL1,LRP2,SEMA6D,NTNG1,MTMR2,VCL,RAPGEF2,AKAP13,UNC5D,ULK2,NRXN3,RIMS2,PGM5,BMPR1B,SEMA5A,ATP8A2,MAP4K4,CTNNA2,RELN,AUTS2,CYFIP2,ADAMTSL1,TNR,CNTN6,APP,PDLIM5,NYAP2,VPS13B,ADGRB1*
GO:0120039	plasma membrane bounded cell projection morphogenesis	6.126306302678361e-16	*MACF1,PAK1,CTNND2,TRIO,NRP1,RPS6KA5,TAOK3,SLC23A2,ARHGEF7,STAU2,CNTN4,AFG3L2,SEMA3A,LRRC4C,ALCAM,DCLK1,SDC2,NCAM1,PHACTR1,SLC39A12,DISC1,HECW2,FYN,B4GALT6,SLIT3,SRGAP2,NOTCH2,RIMS1,GLI3,NRXN1,RERE,ATL1,ZDHHC17,ITGA4,ROCK1,DNM3,SYT1,ARHGAP32,PRKN,PTPRD,ADGRB3,MYO9A,NTRK2,ATP9A,PRKG1,DSCAM,NFIB,DIP2B,PPFIA2,ARHGEF28,CNTN1,DOCK10,EPHA6,NLGN1,ABL1,LRP2,SEMA6D,NTNG1,VCL,RAPGEF2,UNC5D,ULK2,NRXN3,RIMS2,BMPR1B,SEMA5A,ATP8A2,MAP4K4,CTNNA2,RELN,AUTS2,CYFIP2,ADAMTSL1,TNR,CNTN6,APP,PDLIM5,NYAP2,ADGRB1*
GO:0023051	regulation of signaling	7.279738747473604e-16	*FSTL1,ERBB4,MACF1,PAK1,CTNND2,TRIO,EPN2,NETO2,EVC,NRP1,CDH13,RC3H1,EPS8,TAOK3,ABL2,PRKCB,ARHGEF7,GPC5,ARHGEF12,ANKRD6,HTR2C,DYSF,STAU2,CNTN4,CNKSR3,LDLRAD4,SEMA3A,SLC8A3,RFX3,MGAT5,STARD13,LRRC4C,LIMD1,FGD4,GRIK2,SNX25,IGSF11,MBD5,KIF16B,NCAM1,CLSTN2,SIPA1L2,ITGA8,GRIK4,PJA2,TAFA4,TTC21B,NEK6,ARHGAP28,DISC1,SV2B,RALGPS1,ARHGAP42,EYA1,KSR1,APBA2,EDAR,FRMD6,RGS20,FYN,NALCN,CREBBP,DLGAP1,SLIT3,RGS7,GRIN2B,SRGAP2,GRIA1,TRABD2B,SPRED1,DGKB,CACNG2,GARNL3,BTBD9,CRIM1,NOTCH2,MADD,ESR1,NFAT5,TMOD2,PDE4D,ARHGAP26,ERC2,PTPR*

			T,PRKACB,RIMS1,HIPK3,SGMS1,LAMA1,JCAD,GLI3,NRXN1,NTRK3,FBN1,WWOX,MOSMO,RAPGEF4,CNKSR2,NEK10,GRID1,SLC4A10,PHLPP1,ZDHHC17,GSG1L,ROCK1,ARHGAP31,UBR1,PRKAA2,ARHGEF11,SYT1,GRB10,ARHGAP32,PRKN,SIAH2,LATS2,RORA,PTPRD,DGKG,MYO9A,NTRK2,PLCB1,MAPK1,LAMB1,SDCBP,GRIK1,BIRC6,DGKI,NLK,EGFR,PPFIA2,BCR,MAGI2,AKAP6,LARGE1,ARHGEF28,PLCL1,TBC1D1,IQCJ-SCHIP1,SORCS3,RNF152,ZNRF3,GNAQ,ITPKB,GRM5,MED1,AIMP1,NRG3,PID1,NLGN1,ABL1,NCOR1,GRID2,LRP2,ERBIN,FER,PAK5,FBXL17,MCC,PRLR,GRIK3,STXBP4,NTNG1,GNG7,MTMR2,MAPRE2,FGF10,NOS1AP,CADM1,RC3H2,RIC8B,ATF6,SEZ6L,RAPGEF2,RGS12,IFT81,SHANK2,ZMYND11,INSR,SRGAP3,AKAP13,KLF15,DENND4C,ARFGEF1,ENPP1,UNC13C,NRXN3,RIMS2,KCNQ3,DENND2B,DLGAP2,BMPR1B,NSG1,MAPKBP1,SEMA5A,KITLG,SV2C,ARHGAP24,GRIA4,MAP4K4,CNIH3,DOCK3,PRAME,APC,GPC6,ADGRV1,RELN,AUTS2,ROR1,PPP2R3A,BMP2K,SNAI2,CYFIP2,NDFIP1,TNR,CELF4,AKT3,VAV3,IL1R1,PRKCA,USP8,CNTN6,ERC1,APP,SMAD5,PSD3,ALPK2,EYA2,MAPK8IP1,ADGRB1,CCDC88A,GHRH,RAP1GDS1
GO:0048858	cell projection morphogenesis	9.699398738086857e-16	MACF1,PAK1,CTNND2,TRIO,NRP1,RPS6KA5,TAOK3,SLC23A2,ARHGEF7,STAU2,CNTN4,AFG3L2,SEMA3A,LRRC4C,ALCAM,DCLK1,SDC2,NCAM1,PHACTR1,SLC39A12,DISC1,HECW2,FYN,B4GALT6,SLIT3,SRGAP2,NOTCH2,RIMS1,GLI3,NRXN1,RERE,ATL1,ZDHHC17,ITGA4,ROCK1,DNM3,SYT1,ARHGAP32,PRKN,PTPRD,ADGRB3,MYO9A,NTRK2,ATP9A,PRKG1,DSCAM,NFIB,DIP2B,PPFIA2,ARHGEF28,CNTN1,DOCK10,EPHA6,NLGN1,ABL1,LRP2,SEMA6D,NTNG1,VCL,RAPGEF2,UNC5D,ULK2,NRXN3,RIMS2,BMPR1B,SEMA5A,ATP8A2,MAP4K4,CTNNA2,RELN,AUTS2,CYFIP2,ADAMTSL1,TNR,CNTN6,APP,PDLIM5,NYAP2,ADGRB1
GO:0030154	cell differentiation	1.1025808930739493e-15	FSTL1,ERBB4,MACF1,PAK1,CTNND2,TRIO,MITF,RTN1,SETD2,AMBRA1,SYNE2,ALPK3,NRP1,NFIA,FRY,MDGA2,RC3H1,RPS6KA5,TAOK3,ABL2,SLC23A2,ARHGEF7,SLC8A1,EML1,CDK12,HTR2C,STAU2,CNTN4,AFG3L2,LDLRAD4,SEMA3A,SLC8A3,RFX3,RBFOX1,LRRC4C,ALCAM,ATP11C,LIMD1,MYEF2,DCLK1,SLC9C1,ADAMTS9,NIPBL,CEP120,SDC2,NCAM1,TCF12,TANC1,NEBL,ITGA8,SYCP1,TCF4,IREB2,PBX1,PHACTR1,BICRAL,TTC21B,SLC39A12,DISC1,HECW2,EYA1,RORB,GABRB1,NTM,ASAP1,TTLL7,EDAR,ATP2B2,ANKRD26,FRMD6,FYN,ZBTB16,CSMD3,B4GALT6,SLIT3,SRGAP2,ARSB,NEGR1,SPRED1,SPEN,NAV2,SPATA48,TPM1,COL19A1,CRIM1,NOTCH2,FLNB,TOX,PCDH15,ESR1,SRGAP2C,ABCB5,TMOD2,ZSWIM6,PDE4D,TJP1,RIMS1,LAMA1,GLI3,BRINP1,NRXN1,NTRK3,FBN1,WWOX,WASF3,ARID1B,HYDIN,TSPAN2,WDPCP,MOSMO,PIAS1,ZNF521,PBX3,HMGA2,FHOD

			3,RERE,SLC4A10,ATL1,MYO1E,ZDHHC17,ITGA4,ROCK1,HERC1,DOCK1,GLIS1,RXRG,COL4A2,DNM3,PACRG,SYT1,BBS2,ARHGAP32,PRKN,DPF3,LATS2,RORA,PTPRD,BBS9,RANBP3L,HDAC4,ADGRB3,DGKG,MYO9A,NTRK2,PLCB1,MAPK1,LAMB1,SDCBP,ATP9A,SOX5,PRKG1,DSCAM,NFIB,DIP2B,TUT4,THSD7A,EGFR,PPFIA2,PTPRQ,MGA,NCAM2,BCR,MAGI2,NELL1,AKAP6,LARGE1,MBNL1,ARHGEF28,FUT9,MYT1L,CNTN1,POU6F2,DOCK10,TENM3,OPCML,ITPKB,GRM5,MED1,EPHA6,FNDC3A,NRG3,NLGN1,ABL1,PTPRG,JARID2,PAQR5,EBF2,JAM2,GRID2,LRP2,SEMA6D,SMOC1,FER,FBXL17,PRLR,ZNF160,NFATC2,FAT3,NTNG1,MTPN,VSTM2A,MTMR2,LRP12,FGF10,SOX6,CADM1,SGCD,RC3H2,SP3,VCL,ZDHHC21,VCAM1,RAPGEF2,IFT81,AKAP13,ILDR2,KLF15,UNC5D,FAT1,ENPP1,ULK2,NRXN3,RIMS2,KCNQ3,ZFHX3,PGM5,ASTN1,GFRA1,BMPR1B,FCRLA,SEMA5A,ATP8A2,KITLG,PYGO1,ARHGAP24,MAP4K4,CTNNA2,PRAME,APC,APLF,DNER,ABCA5,ADGRV1,RELN,ANXA4,AUTS2,ROR1,PPP2R3A,SNAI2,CYFIP2,KAZN,ADAMTSL1,NDFIP1,TNR,OCA2,CELF4,CRB1,PRKCA,USH1C,MRTFB,CNTN6,FMN2,APP,PDLIM5,RPS6KA3,SMAD5,DNAJC13,NYAP2,VPS13B,ALPK2,ASB2,EYA2,ADGRB1,CCDC88A,SPOCK1
GO:0048869	cellular developmental process	1.139609374567663e-15	*FSTL1,ERBB4,MACF1,PAK1,CTNND2,TRIO,MITF,RTN1,SETD2,AMBRA1,SYNE2,ALPK3,NRP1,NFIA,FRY,MDGA2,RC3H1,RPS6KA5,TAOK3,ABL2,SLC23A2,ARHGEF7,SLC8A1,EML1,CDK12,HTR2C,STAU2,CNTN4,AFG3L2,LDLRAD4,SEMA3A,SLC8A3,RFX3,RBFOX1,LRRC4C,ALCAM,ATP11C,LIMD1,MYEF2,DCLK1,SLC9C1,ADAMTS9,NIPBL,CEP120,SDC2,NCAM1,TCF12,TANC1,NEBL,ITGA8,SYCP1,TCF4,IREB2,PBX1,PHACTR1,BICRAL,TTC21B,SLC39A12,DISC1,HECW2,EYA1,RORB,GABRB1,NTM,ASAP1,TTLL7,EDAR,ATP2B2,ANKRD26,FRMD6,FYN,ZBTB16,CSMD3,B4GALT6,SLIT3,SRGAP2,ARSB,NEGR1,SPRED1,SPEN,NAV2,SPATA48,TPM1,COL19A1,CRIM1,NOTCH2,FLNB,TOX,PCDH15,ESR1,SRGAP2C,ABCB5,TMOD2,ZSWIM6,PDE4D,TJP1,RIMS1,LAMA1,GLI3,BRINP1,NRXN1,NTRK3,FBN1,WWOX,WASF3,ARID1B,HYDIN,TSPAN2,WDPCP,MOSMO,PIAS1,ZNF521,PBX3,HMGA2,FHOD3,RERE,SLC4A10,ATL1,MYO1E,ZDHHC17,ITGA4,ROCK1,HERC1,DOCK1,GLIS1,RXRG,COL4A2,DNM3,PACRG,SYT1,BBS2,ARHGAP32,PRKN,DPF3,LATS2,RORA,PTPRD,BBS9,RANBP3L,HDAC4,ADGRB3,DGKG,MYO9A,NTRK2,PLCB1,MAPK1,LAMB1,SDCBP,ATP9A,SOX5,PRKG1,DSCAM,NFIB,DIP2B,TUT4,THSD7A,EGFR,PPFIA2,PTPRQ,MGA,NCAM2,BCR,MAGI2,NELL1,AKAP6,LARGE1,MBNL1,ARHGEF28,FUT9,MYT1L,CNTN1,POU6F2,DOCK10,TENM3,OPCML,ITPKB,GRM5,MED1,EPHA6,FNDC3A,NRG3,NLGN1,ABL1,PTPRG,JARID2,PAQR5,EBF2,JAM2,GRID2,LRP2,SEMA6D,SMOC1,FER,FBXL17,PRLR,ZNF160,NFATC2,FAT3,NTNG1,MTPN,VSTM2A,MTMR2,*

			LRP12,FGF10,SOX6,CADM1,SGCD,RC3H2,SP3,VCL,ZDHHC21,VCAM1,RAPGEF2,IFT81,AKAP13,ILDR2,KLF15,UNC5D,FAT1,ENPP1,ULK2,NRXN3,RIMS2,KCNQ3,ZFHX3,PGM5,ASTN1,GFRA1,BMPR1B,FCRLA,SEMA5A,ATP8A2,KITLG,PYGO1,ARHGAP24,MAP4K4,CTNNA2,PRAME,APC,APLF,DNER,ABCA5,ADGRV1,RELN,ANXA4,AUTS2,ROR1,PPP2R3A,SNAI2,CYFIP2,KAZN,ADAMTSL1,NDFIP1,TNR,OCA2,CELF4,CRB1,PRKCA,USH1C,MRTFB,CNTN6,FMN2,APP,PDLIM5,RPS6KA3,SMAD5,DNAJC13,NYAP2,VPS13B,ALPK2,ASB2,EYA2,ADGRB1,CCDC88A,SPOCK1
GO:0007155	cell adhesion	1.2269679294032576e-15	*LPP,FYB2,MACF1,CTNND2,KIRREL1,AMBRA1,NRP1,CDH13,RC3H1,LRFN5,ABL2,SLC23A2,ARHGEF7,UTRN,CNTN4,FRMD5,PCDH7,LRRC4C,CADM2,ALCAM,ADAM32,ADAMTS9,HMCN1,IGSF11,NCAM1,CLSTN2,CORO2B,ITGA8,MYO10,DISC1,FMN1,PEAK1,NTM,FYN,ZBTB16,ANTXR1,SRGAP2,NEGR1,TPM1,COL19A1,CLDN10,PCDH15,NFAT5,MEGF11,TJP1,PTPRT,PARVB,LAMA1,JCAD,GLI3,NRXN1,FBN1,ARID1B,WDPCP,MAGI1,CDH20,LSAMP,PKN2,ITGA4,ROCK1,DOCK1,CNTN3,TRPM7,EDIL3,PTPRD,TLN2,LAMB1,PRKG1,DSCAM,EGFR,PPFIA2,STXBP6,NCAM2,BCR,MELTF,FUT9,CNTN1,TENM3,OPCML,ITPKB,FNDC3A,COL14A1,NLGN1,ABL1,JAM2,GRID2,ERBIN,FER,PRLR,DST,FAT3,NTNG1,CADM1,RC3H2,PCDH9,VCL,ZDHHC21,CNTNAP5,VCAM1,CDH7,ILDR2,UNC5D,FAT1,ITGA9,NRXN3,PPM1F,ZFHX3,PGM5,ASTN1,SEMA5A,KITLG,FREM1,MAP4K4,CTNNA2,APC,PARD3B,ADGRV1,RELN,SNAI2,CYFIP2,ADAMTSL1,NDFIP1,TNR,CRB1,VAV3,PRKCA,HEPACAM,CNTN6,APP,PDLIM5,ADGRB1,SPOCK1*
GO:0048513	animal organ development	1.367420501894655e-15	*ERBB4,PDE6A,RNF38,MITF,RTN1,SETD2,SYNE2,IGSF3,ALPK3,MYO3B,EVC,NRP1,TEAD1,NFIA,MDGA2,RC3H1,PRKCB,UTRN,SLC8A1,EML1,ANKRD6,KCNC1,STAU2,CNTN4,LDLRAD4,SEMA3A,RFX3,RBFOX1,MMP16,SLC40A1,CADM2,ADAM32,DCLK1,DNAH11,ADAMTS9,NIPBL,CEP120,TCF12,KDM4B,ADGRL2,NEBL,ITGA8,PBX1,PHACTR1,TTC21B,DISC1,FMN1,EYA1,RORB,SVIL,EDAR,ATP2B2,FYN,SLC1A2,ZBTB16,SLIT3,GRIN2B,SRGAP2,GRIA1,SPRED1,FGF12,TPM1,COL19A1,NOTCH2,FLNB,TOX,PCDH15,ESR1,SRGAP2C,ABCB5,ZSWIM6,MEGF11,LRIG1,LAMA1,GLI3,NRXN1,NTRK3,SGCG,FBN1,WWOX,HYDIN,WDPCP,PBX3,PRICKLE2,HMGA2,FHOD3,GREB1L,RERE,SLC4A10,MYO1E,ROCK1,HERC1,CACNA1C,BBS2,ARHGAP32,ASXL3,DPF3,FOXP2,SCAPER,LATS2,EXOC4,RORA,RANBP3L,TG,HDAC4,ADAM29,MYO3A,FOXN3,NTRK2,PLCB1,MAPK1,LAMB1,SDCBP,BPTF,SOX5,PRKG1,DSCAM,BIRC6,NFIB,EGFR,PTPRQ,BCR,MAGI2,NELL1,AKAP6,LARGE1,SCN2A,CNTN1,ZNRF3,TENM3,MED1,ENPEP,FNDC3A,CA10,LDB2,WDR72,NRG3,ABL1,JARID2,EBF2,GRID2,LRP2,SEMA6D,MBTPS2,SMOC1,FER,SETDB2,RYR2,FBXL17,PRLR,NFATC2,FAT3,MTPN,FGF10,S*

			OX6,CADM1,SGCD,RC3H2,PCDH9,ATF6,SP3,ZDHHC21,VCAM1,RAPGEF2,INSR,AKAP13,ILDR2,IMMP2L,SYNJ1,KLF15,ATXN1,FAT1,ENPP1,ZFHX3,GFRA1,BMPR1B,CPE,SEMA5A,ATP8A2,SLC24A3,KITLG,PYGO1,FREM1,CTNNA2,APC,APLF,GPC6,DNER,ADGRV1,RELN,MACROD2,BNC2,ROR1,PPP2R3A,BMP2K,SNAI2,KAZN,TNR,CELF4,AKT3,CRB1,USH1C,MRTFB,DNAH5,APP,PDLIM5,XYLT1,SMAD5,SLC6A3,VPS13B,ALPK2,ASB2,ADGRB1,GHRH,ARNT2
GO:0032990	cell part morphogenesis	1.6298790011699335e-15	*MACF1,PAK1,CTNND2,TRIO,NRP1,RPS6KA5,TAOK3,SLC23A2,ARHGEF7,STAU2,CNTN4,AFG3L2,SEMA3A,LRRC4C,ALCAM,DCLK1,SDC2,NCAM1,PHACTR1,SLC39A12,DISC1,HECW2,FYN,B4GALT6,SLIT3,SRGAP2,NOTCH2,RIMS1,GLI3,NRXN1,RERE,ATL1,ZDHHC17,ITGA4,ROCK1,DNM3,SYT1,ARHGAP32,PRKN,PTPRD,ADGRB3,MYO9A,NTRK2,ATP9A,PRKG1,DSCAM,NFIB,DIP2B,PPFIA2,ARHGEF28,CNTN1,DOCK10,EPHA6,PID1,NLGN1,ABL1,LRP2,SEMA6D,NTNG1,VCL,RAPGEF2,UNC5D,ULK2,NRXN3,RIMS2,BMPR1B,SEMA5A,ATP8A2,MAP4K4,CTNNA2,RELN,AUTS2,CYFIP2,ADAMTSL1,TNR,CNTN6,APP,PDLIM5,NYAP2,ADGRB1*
GO:0065008	regulation of biological quality	2.01574520346671e-15	*SLMAP,SAMD4A,ERBB4,KCNMA1,MACF1,SLC6A11,ATP9B,KIRREL1,NETO2,MYO3B,NRP1,RC3H1,RPS6KA5,EPS8,LRFN5,ABL2,PRKCB,ARHGEF7,SLC8A1,LNPEP,PRR16,HTR2C,KCNC1,DYSF,STAU2,SIAH3,CNTN4,SEMA3A,SLC8A3,RFX3,ATP11C,LIMD1,SLC9C1,CDC42EP3,FGD4,GRIK2,IGSF11,CCDC186,CLSTN2,ADGRL2,TANC1,CORO2B,GRIK4,MYO10,TAFA4,GABRG1,IREB2,FAM171A1,ATP10A,ARHGAP28,DISC1,SV2B,RIC3,FMN1,ARHGAP42,PIEZO2,GABRB1,SVIL,ASAP1,FRMPD4,FYN,SH3KBP1,AGO2,NALCN,CREBBP,SLC1A2,CADPS,GRIN2B,GRIA1,NEGR1,NAV2,FGF12,DGKB,CACNG2,TPM1,ESR1,GABRG3,ABCB5,TMOD2,PDE4D,TJP1,ERC2,PTPRT,PRKACB,RIMS1,FCHSD2,PARVB,DOCK4,NRXN1,WASF3,CPQ,WDPCP,MOSMO,RAPGEF4,CTTNBP2,FHOD3,GRID1,TNRC6B,SEL1L,SLC4A10,SYN2,ABCC9,GSG1L,ROCK1,CACNA1C,DNM3,SYT1,BBS2,ARHGAP32,PRKN,PTPRD,PTPRN2,TG,ADGRB3,DGKG,MYO3A,NTRK2,PLCB1,MAPK1,ATP9A,GRIK1,PRKG1,DSCAM,DGKI,DIP2B,TUT4,NLK,NBEA,PPFIA2,BCR,AKAP6,LARGE1,STX12,TBC1D1,SORCS3,SLC12A1,GNAQ,GRM5,MED1,ENPEP,AIMP1,ATP8A1,PID1,NLGN1,ABL1,GRID2,ERBIN,SEMA6D,FER,RYR2,PRLR,GRIK3,CACNB2,STXBP4,MTPN,MTMR2,KCNH5,FGF10,NOS1AP,RC3H2,VCL,ZDHHC21,XKR6,RAPGEF2,SHANK2,ILDR2,SYNJ1,GABRG2,ARFGEF1,ULK2,UNC13C,NRXN3,RIMS2,KCNQ3,SCN8A,NSG1,CPE,KCND2,SEMA5A,ATP8A2,SV2C,GRIA4,MAP4K4,CTNNA2,GPC6,ADGRV1,RELN,AKAP11,CYFIP2,SLC4A4,TNR,PTPN13,OCA2,CELF4,AKT3,VAV3,PRKCA,USP8,USH1C,ERC1,APP,PDLIM5,SLC6A3,EYA2,ADGRB1,GHRH,RAP1GDS1*

GO:0050804	modulation of chemical synaptic transmission	3.9240554465294744e-15	TRIO,NETO2,PRKCB,DYSF,STAU2,CNTN4,SLC8A3,LRRC4C,GRIK2,IGSF11,CLSTN2,GRIK4,DISC1,SV2B,APBA2,FYN,NALCN,DLGAP1,GRIN2B,GRIA1,DGKB,CACNG2,BTBD9,ERC2,RIMS1,NRXN1,GRID1,SLC4A10,SYT1,PRKN,PTPRD,NTRK2,PLCB1,MAPK1,GRIK1,DGKI,PPFIA2,BCR,LARGE1,PLCL1,SORCS3,GRM5,NRG3,NLGN1,ABL1,GRID2,GRIK3,NTNG1,MTMR2,RAPGEF2,SHANK2,UNC13C,NRXN3,RIMS2,KCNQ3,DLGAP2,NSG1,SV2C,GRIA4,RELN,TNR,CELF4,USP8,ERC1,APP,ADGRB1
GO:0099177	regulation of trans-synaptic signaling	4.3646625017282386e-15	TRIO,NETO2,PRKCB,DYSF,STAU2,CNTN4,SLC8A3,LRRC4C,GRIK2,IGSF11,CLSTN2,GRIK4,DISC1,SV2B,APBA2,FYN,NALCN,DLGAP1,GRIN2B,GRIA1,DGKB,CACNG2,BTBD9,ERC2,RIMS1,NRXN1,GRID1,SLC4A10,SYT1,PRKN,PTPRD,NTRK2,PLCB1,MAPK1,GRIK1,DGKI,PPFIA2,BCR,LARGE1,PLCL1,SORCS3,GRM5,NRG3,NLGN1,ABL1,GRID2,GRIK3,NTNG1,MTMR2,RAPGEF2,SHANK2,UNC13C,NRXN3,RIMS2,KCNQ3,DLGAP2,NSG1,SV2C,GRIA4,RELN,TNR,CELF4,USP8,ERC1,APP,ADGRB1
GO:0007165	signal transduction	2.4369065185324927e-14	FSTL1,ERBB4,PDE6A,APBB2,FYB2,MACF1,PAK1,CTNND2,TRIO,MITF,EPN2,NETO2,PDE1C,EVC,NRP1,TEAD1,NFIA,CDH13,RC3H1,RPS6KA5,EPS8,TAOK3,OR4C46,ABL2,PRKCB,ARHGEF7,GPC5,MAML2,ARHGEF12,SAMD12,SLC8A1,LNPEP,ANKRD6,RERG,HTR2C,CNKSR3,LDLRAD4,SEMA3A,SLC8A3,MGAT5,STARD13,ALCAM,ERLIN2,LIMD1,PIK3C3,DCLK1,MAGI3,CDC42EP3,FGD4,GRIK2,SNX25,IGSF11,RGL1,MBD5,KIF16B,NCAM1,SIPA1L2,RCAN2,ADGRL2,GNG12,PAPPA,ITGA8,GRIK4,MYO10,PJA2,ASB3,TAFA4,GABRG1,TOM1L2,TTC21B,NEK6,SLC39A12,MOB1B,ARHGAP28,DISC1,RALGPS1,ARHGAP42,EYA1,KSR1,RORB,GPR158,GABRB1,ANKS1B,EDAR,FRMD6,RGS20,FYN,CREBBP,DLGAP1,SLIT3,RGS7,FHIT,GRIN2B,SRGAP2,STK32A,GRIA1,TRABD2B,TAFA5,SPRED1,MYO5A,SPEN,FGF12,DGKB,CACNG2,GARNL3,CRIM1,NOTCH2,FLNB,MADD,ESR1,GABRG3,NFAT5,TMOD2,PLCXD3,PDE4D,TJP1,ARHGAP26,PTPRT,PRKACB,GNG2,TRHDE,RIMS1,HIPK3,WSB1,SGMS1,LAMA1,JCAD,GLI3,DOCK4,MAST4,GAST,ITPR2,NRXN1,NTRK3,FBN1,WWOX,WDPCP,MAGI1,MOSMO,RAPGEF4,CNKSR2,NEK10,PIAS1,PRICKLE2,HMGA2,HHAT,GRID1,PARP8,SEL1L,PHLPP1,MYO1E,ZDHHC17,PKN2,ITGA4,GSG1L,ROCK1,DCDC1,DOCK1,CACNA1C,ARHGAP31,UBR1,RXRG,PRKAA2,ARHGEF11,SORCS1,COL4A2,BBS2,GRB10,ARHGAP32,PRKN,SIAH2,LATS2,DOCK9,PPP1R12B,RORA,PTPRD,CDC14B,TTR,TG,HDAC4,ADGRB3,INPP4B,DGKG,MYO9A,FOXN3,NTRK2,PLCB1,MAPK1,LAMB1,SDCBP,GRIK1,CUL5,PRKG1,DSCAM,BIRC6,DGKI,NLK,EGFR,BCR,MAGI2,AKAP6,LARGE1,ARHGEF28,PLCL1,MOK,IQCJ-SCHIP1,SORCS3,SCN2A,RNF152,CNTN1,ZNRF3,GNAQ,DOCK10,TENM3,ITPKB,GRM5,MED1,E

			PHA6,ARAP2,AIMP1,NRG3,CPEB4,PID1,NLGN1,ABL1,PTPRG,NCOR1,OR9Q1,GRID2,ELMO1,LRP2,ERBIN,SEMA6D,PPM1L,CABIN1,MBTPS2,FER,PAK5,RYR2,IL17RA,FBXL17,MCC,PRLR,NFATC2,DST,GRIK3,STXBP4,GNG7,MTMR2,LRP12,MAPRE2,FGF10,NOS1AP,CADM1,SGCD,RC3H2,RIC8B,ATF6,STK32B,ZNF106,SEZ6L,ZDHHC21,VCAM1,RAPGEF2,RGS12,IFT81,SHANK2,ZMYND11,RAPGEF5,INSR,SRGAP3,PDK1,AKAP13,KLF15,DENND4C,UNC5D,GABRG2,ARFGEF1,PATJ,ITGA9,ENPP1,ULK2,NRXN3,RIMS2,RHPN2,PPM1F,KCNQ3,DENND2B,DLGAP2,GFRA1,ATRNL1,GPR156,BMPR1B,NSG1,MAPKBP1,CPE,FCRLA,SEMA5A,KITLG,PYGO1,ARHGAP24,GRIA4,MAP4K4,CNIH3,DOCK3,PRAME,APC,GPC6,DNER,ADGRV1,RELN,AKAP11,ANXA4,AUTS2,ROR1,PPP2R3A,BMP2K,SNAI2,CYFIP2,NDFIP1,DTNA,CELF4,AKT3,VAV3,IL1R1,PRKCA,INPP5A,USP8,CNTN6,FMN2,ERC1,APP,RPS6KA3,SMAD5,PSD3,ALPK2,ASB2,EYA2,MAPK8IP1,ADGRB1,CCDC88A,GHRH,RAP1GDS1
GO:0051179	localization	9.868224973489982e-14	*SLMAP,ERBB4,KCNMA1,FYB2,CUBN,MACF1,SLC17A1,PAK1,SLC6A11,ATP9B,SETD2,SLC15A5,SYNE2,EPN2,NETO2,DOP1B,NRP1,CDH13,TANGO2,HERC2,ABL2,PRKCB,SLC23A2,ARHGEF7,UTRN,GPC5,SLC8A1,LYPLA1,SLC2A13,HTR2C,KCNC1,DYSF,STAU2,CACNA1E,SIAH3,TMEM63C,FYCO1,TBC1D5,EXOC6B,AFG3L2,CNKSR3,PLIN2,SLC8A3,RFX3,RBFOX1,SLC40A1,SLCO3A1,ATP11C,PIK3C3,DCLK1,DNAH11,SLC9C1,ADAMTS9,NIPBL,CEP120,GRIK2,SNX25,IGSF11,CCDC186,MYO5C,KIF16B,NKAIN3,SLC25A21,ODR4,CORO2B,EVI5,ITGA8,SYCP1,VPS41,GRIK4,MYO10,ASB3,TAFA4,GABRG1,TOM1L2,IREB2,ATP10A,TTC21B,ABCD2,SLC39A12,COG2,DISC1,CLTCL1,SV2B,HECW2,RIC3,PIEZO2,SLC35F1,GPR158,GABRB1,TMEM241,APBA2,LYST,SLC16A9,PLEKHA3,ATP2B2,FRMD6,FYN,SH3KBP1,NALCN,CREBBP,SLC1A2,ZBTB16,CADPS,LRBA,RGS7,SGTB,GRIN2B,ANTXR1,LRP1B,ARSB,GRIA1,AP5M1,MYO5A,FGF12,CACNG2,TSPAN33,BTBD9,NPIPA1,CLDN10,ESR1,GABRG3,ABCB5,SLC44A5,MEGF11,PDE4D,TJP1,ERC2,RIMS1,FCHSD2,GLI3,ITPR2,NRXN1,FRMD4A,FBN1,LONP2,TRAPPC10,WDPCP,RAPGEF4,EHBP1,NEK10,DPP6,PRELID2,GRID1,CACNA2D3,SEL1L,SLC4A10,SYN2,MYO1E,SLC7A2,ZDHHC17,ITGA4,ABCC9,GSG1L,ROCK1,DOCK1,CACNA1C,PRKAA2,AMPH,RGPD4,SORCS1,DNM3,PACRG,SYT1,BBS2,GRB10,SLC27A6,PRKN,TRPM7,LATS2,EXOC4,DLG2,SCFD2,BBS9,PTPRN2,RANBP3L,TG,NTRK2,PLCB1,MAPK1,SDCBP,ATP9A,GRIK1,CUL5,PRKG1,DGKI,SPOP,NBEA,EGFR,PPFIA2,STXBP6,CLCN5,BCR,MAGI2,AKAP6,LARGE1,TRAPPC8,STX12,MELTF,TMPRSS3,ANKFY1,TBC1D1,SLC37A2,SLC12A1,SCN2A,CNTN1,GRM5,MED1,GNPTAB,SPIRE1,AIMP1,WDR72,SNX30,ATP8A1,PID1,NLGN1,ABL1,JARID2,MICAL3,GRID2,ELMO1,LRP2,*

			RANBP17,ERBIN,FER,MON2,RYR2,SCAMP1,LDLRAD3,MCC,PRLR,DST,GRIK3,CACNB2,STXBP4,VSTM2A,MTMR2,KCNH5,LRP12,MAPRE2,LRRC8B,FGF10,KIF13A,NOS1AP,SGCD,TRPM3,IPO11,VCL,ZDHHC21,XKR6,RAPGEF2,IFT81,INSR,UNC80,WDR41,ILDR2,IMMP2L,SYNJ1,KLF15,DENND4C,ATXN1,GABRG2,ARFGEF1,ENPP1,UNC13C,TANGO6,NRXN3,RIMS2,PPM1F,KCNQ3,SCN8A,SNX8,NSG1,CPE,KCND2,ATP8A2,SLC24A3,PYGO1,SV2C,GRIA4,MAP4K4,EFR3A,CNIH3,APC,APLF,GPC6,PARD3B,DNER,ABCA5,ADGRV1,RELN,AKAP11,VPS13C,KCNJ15,CEP112,CYBRD1,STON1-GTF2A1L,BMP2K,SLC4A4,NDFIP1,SNAP91,OCA2,CRB1,VAV3,FCHO2,RFTN2,HEPACAM,USP8,USH1C,DNAH5,KCNS3,FMN2,ERC1,APP,SLC6A3,DNAJC13,VPS13B,ABCA13,EYA2,MAPK8IP1,ADGRB1,CCDC88A,GHRH,RAP1GDS1
GO:0048468	cell development	1.0942844847376146e-13	*ERBB4,MACF1,PAK1,CTNND2,TRIO,MITF,SETD2,AMBRA1,ALPK3,NRP1,FRY,RC3H1,RPS6KA5,TAOK3,ABL2,SLC23A2,ARHGEF7,SLC8A1,EML1,STAU2,CNTN4,AFG3L2,SEMA3A,SLC8A3,RFX3,LRRC4C,ALCAM,ATP11C,LIMD1,DCLK1,SDC2,NCAM1,NEBL,SYCP1,IREB2,PBX1,PHACTR1,SLC39A12,DISC1,HECW2,RORB,GABRB1,NTM,ASAP1,FRMD6,FYN,ZBTB16,CSMD3,B4GALT6,SLIT3,SRGAP2,ARSB,NEGR1,SPEN,TPM1,NOTCH2,FLNB,TOX,PCDH15,ESR1,SRGAP2C,TMOD2,PDE4D,TJP1,RIMS1,LAMA1,GLI3,BRINP1,NRXN1,NTRK3,FBN1,WASF3,ARID1B,HYDIN,TSPAN2,WDPCP,PBX3,FHOD3,RERE,SLC4A10,ATL1,MYO1E,ZDHHC17,ITGA4,ROCK1,HERC1,DOCK1,DNM3,PACRG,SYT1,BBS2,ARHGAP32,PRKN,RORA,PTPRD,HDAC4,ADGRB3,DGKG,MYO9A,NTRK2,PLCB1,MAPK1,LAMB1,ATP9A,PRKG1,DSCAM,NFIB,DIP2B,TUT4,PPFIA2,PTPRQ,NCAM2,BCR,MAGI2,AKAP6,LARGE1,ARHGEF28,FUT9,MYT1L,CNTN1,DOCK10,TENM3,OPCML,ITPKB,GRM5,MED1,EPHA6,FNDC3A,NLGN1,ABL1,PTPRG,PAQR5,JAM2,GRID2,LRP2,SEMA6D,FER,ZNF160,NFATC2,FAT3,NTNG1,MTMR2,LRP12,SGCD,RC3H2,SP3,VCL,ZDHHC21,VCAM1,RAPGEF2,IFT81,AKAP13,UNC5D,FAT1,ULK2,NRXN3,RIMS2,KCNQ3,PGM5,GFRA1,BMPR1B,SEMA5A,ATP8A2,KITLG,PYGO1,MAP4K4,CTNNA2,DNER,ADGRV1,RELN,AUTS2,ROR1,SNAI2,CYFIP2,ADAMTSL1,NDFIP1,TNR,OCA2,CELF4,CRB1,PRKCA,USH1C,CNTN6,FMN2,APP,PDLIM5,SMAD5,NYAP2,VPS13B,ALPK2,ASB2,ADGRB1,CCDC88A,SPOCK1*
GO:0007417	central nervous system development	6.198223730240454e-13	*ERBB4,RTN1,SETD2,SYNE2,NRP1,MDGA2,EML1,KCNC1,CNTN4,SEMA3A,SLC8A3,CADM2,DCLK1,NIPBL,CEP120,KDM4B,ADGRL2,PBX1,PHACTR1,TTC21B,DISC1,GABRB1,FYN,SLC1A2,ZBTB16,B4GALT6,GRIN2B,SRGAP2,GRIA1,NAV2,TOX,SRGAP2C,ZSWIM6,GLI3,BRINP1,NRXN1,WASF3,HYDIN,TSPAN2,PBX3,RERE,SLC4A10,HERC1,BBS2,ARHGAP32,PRKN,FOXP2,RORA,NTRK2,PLCB1,MAPK1,LAMB1,BPTF,GRIK1,P*

			RKG1,NFIB,EGFR,BCR,LARGE1,ARHGEF28,SCN2A,CNTN1,POU6F2,MED1,CA10,NRG3,ABL1,JARID2,GRID2,LRP2,SEMA6D,MTPN,FGF10,SOX6,PCDH9,RAPGEF2,IMMP2L,SYNJ1,ATXN1,ZFHX3,BMPR1B,SEMA5A,CTNNA2,DNER,RELN,MACROD2,ROR1,TNR,AKT3,DNAH5,CNTN6,APP,RPS6KA3,SLC6A3,VPS13B,GHRH,ARNT2,SPOCK1
GO:0034329	cell junction assembly	1.9469570653433832e-12	ERBB4,MACF1,CTNND2,NRP1,CDH13,LRFN5,ARHGEF7,STAU2,CLSTN2,ADGRL2,CORO2B,FMN1,PEAK1,SRGAP2,GRIA1,NEGR1,CLDN10,SRGAP2C,TJP1,NRXN1,WDPCP,CDH20,PKN2,ROCK1,DNM3,PTPRD,TLN2,ADGRB3,MYO9A,NTRK2,SDCBP,DSCAM,BCR,LARGE1,NRG3,NLGN1,ABL1,GRID2,FER,DST,MPDZ,VCL,RAPGEF2,CDH7,SHANK2,GABRG2,PATJ,NRXN3,PPM1F,MAP4K4,APC,DNER,SNAI2,PTPN13,PRKCA,APP,PDLIM5,ADGRB1
GO:0051716	cellular response to stimulus	2.2796917193778696e-12	FSTL1,ERBB4,PDE6A,APBB2,FYB2,MACF1,PAK1,CTNND2,TRIO,MITF,SETD2,AMBRA1,EPN2,NETO2,PDE1C,EVC,NRP1,TEAD1,NFIA,CDH13,RC3H1,RPS6KA5,EPS8,TAOK3,HERC2,OR4C46,ABL2,PRKCB,SLC23A2,ARHGEF7,GPC5,MAML2,ARHGEF12,SAMD12,SLC8A1,LNPEP,ANKRD6,RERG,HTR2C,KCNC1,STAU2,NEK4,USP14,CNKSR3,LDLRAD4,SEMA3A,PLIN2,SLC8A3,MGAT5,STARD13,SLC40A1,ALCAM,ERLIN2,LIMD1,PIK3C3,DCLK1,MAGI3,NIPBL,CDC42EP3,FGD4,GRIK2,SNX25,IGSF11,RGL1,CCDC186,MBD5,KIF16B,NCAM1,MARCHF6,SIPA1L2,RCAN2,ADGRL2,GNG12,CORO2B,PAPPA,ITGA8,SYCP1,VPS41,GRIK4,ZBTB20,MYO10,PJA2,ASB3,TAFA4,GABRG1,TOM1L2,TTC21B,NEK6,SLC39A12,MOB1B,ARHGAP28,DISC1,RALGPS1,ARHGAP42,EYA1,KSR1,RORB,PIEZO2,GPR158,ZNF236,GABRB1,ANKS1B,LYST,EDAR,ST8SIA1,FRMD6,RGS20,FYN,CREBBP,SLC1A2,DLGAP1,SLIT3,RGS7,FHIT,GRIN2B,SRGAP2,STK32A,GRIA1,TRABD2B,TAFA5,SPRED1,MYO5A,SPEN,FGF12,DGKB,CACNG2,GARNL3,TPM1,CRIM1,NOTCH2,FLNB,MADD,ESR1,GABRG3,NFAT5,TMOD2,PLCXD3,PDE4D,TJP1,ARHGAP26,PTPRT,PRKACB,GNG2,TRHDE,RIMS1,HIPK3,WSB1,SGMS1,LAMA1,JCAD,GLI3,DOCK4,MAST4,GAST,ITPR2,BRINP1,NRXN1,SUPT16H,NTRK3,FBN1,WWOX,ARID1B,MTUS1,WDPCP,MAGI1,MOSMO,RAPGEF4,CNKSR2,NEK10,PIAS1,PRICKLE2,HMGA2,HHAT,GRID1,PARP8,SEL1L,PHLPP1,MYO1E,ZDHHC17,PKN2,ITGA4,GSG1L,ROCK1,DCDC1,DOCK1,CACNA1C,ARHGAP31,UBR1,RXRG,PRKAA2,ARHGEF11,SORCS1,COL4A2,PACRG,SYT1,BBS2,GRB10,ARHGAP32,PRKN,DPF3,SIAH2,LATS2,DOCK9,DLG2,PPP1R12B,RORA,PTPRD,CDC14B,TTR,PTPRN2,TG,HDAC4,ADGRB3,INPP4B,DGKG,MYO9A,FOXN3,NTRK2,PLCB1,MAPK1,LAMB1,SDCBP,GRIK1,CUL5,PRKG1,DSCAM,BIRC6,DGKI,NLK,EGFR,BCR,MAGI2,AKAP6,LARGE1,FANCM,ARHGEF28,PLCL1,MOK,PXDNL,TBC1D1,IQCJ-

			SCHIP1,SORCS3,SCN2A,ERP27,RNF152,CNTN1,ZNRF3,GNAQ,DOCK10,TENM3,ITPKB,GRM5,MED1,EPHA6,SPIRE1,ARAP2,AIMP1,NRG3,CPEB4,PID1,NLGN1,ABL1,PTPRG,NCOR1,JARID2,OR9Q1,GRID2,ELMO1,LRP2,ERBIN,SEMA6D,PPM1L,CABIN1,MBTPS2,FER,PAK5,RYR2,IL17RA,FBXL17,MCC,GLYAT,PRLR,HADHB,NFATC2,DST,GRIK3,STXBP4,MTPN,GNG7,MTMR2,LRP12,MAPRE2,FGF10,NOS1AP,CADM1,SGCD,RC3H2,MSRA,RIC8B,ATF6,STK32B,ZNF106,SEZ6L,ZDHHC21,VCAM1,RAPGEF2,RGS12,IFT81,SHANK2,ZMYND11,RAPGEF5,INSR,SRGAP3,PDK1,AKAP13,IMMP2L,CHCHD6,KLF15,DENND4C,UNC5D,GABRG2,ARFGEF1,PATJ,ITGA9,ENPP1,ULK2,NRXN3,RIMS2,RHPN2,PPM1F,KCNQ3,DENND2B,EGLN3,DLGAP2,GFRA1,ATRNL1,GPR156,BMPR1B,NSG1,MAPKBP1,CPE,KCND2,FCRLA,SEMA5A,KITLG,PYGO1,ARHGAP24,GRIA4,MAP4K4,CNIH3,DOCK3,PRAME,APC,APLF,GPC6,DNER,ADGRV1,RELN,MACROD2,AKAP11,VPS13C,ANXA4,NAT1,AUTS2,ROR1,PPP2R3A,MTMR3,BMP2K,SNAI2,CYFIP2,NDFIP1,TNR,PTPN13,DTNA,CELF4,AKT3,CRB1,VAV3,IL1R1,BCAS3,PRKCA,INPP5A,USP8,CNTN6,FMN2,ERC1,APP,RPS6KA3,SMAD5,PSD3,ALPK2,ASB2,EYA2,MAPK8IP1,ADGRB1,CCDC88A,GHRH,RAP1GDS1
GO:0048667	cell morphogenesis involved in neuron differentiation	2.7661937955961964e-12	*MACF1,PAK1,CTNND2,TRIO,NRP1,RPS6KA5,STAU2,CNTN4,AFG3L2,SEMA3A,LRRC4C,ALCAM,DCLK1,SDC2,NCAM1,PHACTR1,DISC1,HECW2,FYN,B4GALT6,SLIT3,NOTCH2,PCDH15,GLI3,NRXN1,WDPCP,RERE,ATL1,ZDHHC17,ITGA4,DNM3,ARHGAP32,PTPRD,ADGRB3,NTRK2,PRKG1,DSCAM,NFIB,DIP2B,PPFIA2,PTPRQ,ARHGEF28,CNTN1,DOCK10,EPHA6,NLGN1,ABL1,SEMA6D,NTNG1,VCL,RAPGEF2,UNC5D,ULK2,NRXN3,BMPR1B,SEMA5A,ATP8A2,CTNNA2,RELN,AUTS2,CYFIP2,ADAMTSL1,TNR,CNTN6,APP,PDLIM5,ADGRB1*
GO:0050793	regulation of developmental process	4.868436140178604e-12	*ERBB4,MACF1,PAK1,TRIO,MITF,AMBRA1,EPN2,NRP1,RC3H1,EPS8,PRKCB,SLC23A2,ARHGEF7,SLC8A1,CDK12,ANKRD6,HTR2C,STAU2,CNTN4,AFG3L2,LDLRAD4,SEMA3A,RFX3,RBFOX1,LRRC4C,ATP11C,LIMD1,ADAMTS9,NIPBL,CDC42EP3,FGD4,MBD5,SDC2,CLSTN2,TCF12,ADGRL2,MYO10,TCF4,PBX1,FAM171A1,ATP10A,BICRAL,SLC39A12,DISC1,HECW2,EYA1,RORB,ASAP1,ANKRD26,FYN,SH3KBP1,AGO2,ZBTB16,CSMD3,TAFA5,SPRED1,SPEN,TPM1,CRIM1,NOTCH2,TOX,ESR1,SRGAP2C,TJP1,RIMS1,PARVB,LAMA1,JCAD,GLI3,BRINP1,NRXN1,FBN1,WASF3,ARID1B,WDPCP,MOSMO,PIAS1,PRICKLE2,HMGA2,ROCK1,GLIS1,COL4A2,DNM3,SYT1,BBS2,ARHGAP32,PRKN,DPF3,LATS2,RORA,PTPRD,RANBP3L,TG,HDAC4,ADGRB3,DGKG,MYO9A,NTRK2,PLCB1,MAPK1,LAMB1,SDCBP,SOX5,DSCAM,NFIB,DIP2B,EGFR,PPFIA2,BCR,MAGI2,NELL1,AKAP6,ZNRF3,ITPKB,GRM5,MED1,SPIRE1,PID1,NLGN1,ABL1,JARID2,JAM2,G*

			RID2,LRP2,SEMA6D,SMOC1,PRLR,NFATC2,FAT3,NTNG1,MTPN,VSTM2A,MTMR2,FGF10,SOX6,RC3H2,VCL,ZDHHC21,RAPGEF2,INSR,ENPP1,ULK2,RIMS2,ZFHX3,BMPR1B,SEMA5A,ATP8A2,KITLG,MAP4K4,PRAME,APC,APLF,GPC6,ABCA5,ADGRV1,RELN,ROR1,BMP2K,SNAI2,NDFIP1,TNR,PTPN13,CELF4,AKT3,PRKCA,MRTFB,APP,PDLIM5,RPS6KA3,SMAD5,SLC6A3,ALPK2,ADGRB1,GHRH
GO:0016477	cell migration	7.557627387575634e-12	*FSTL1,ERBB4,MACF1,PAK1,MITF,SETD2,SYNE2,NRP1,CDH13,EPS8,ABL2,ARHGEF7,GPC5,DACH1,FRMD5,LDLRAD4,SEMA3A,MGAT5,STARD13,LIMD1,DCLK1,ADAMTS9,CDC42BPA,NIPBL,SDC2,TAFA4,PHACTR1,DISC1,PEAK1,LYST,FYN,SH3KBP1,AGO2,SRGAP2,ARSB,TAFA5,SPRED1,TPM1,SRGAP2C,TJP1,PTPRT,LAMA1,JCAD,GLI3,DOCK4,NTRK3,MTUS1,WDPCP,RERE,PKN2,ITGA4,ROCK1,DOCK1,GRB10,ARHGAP32,NTRK2,PLCB1,MAPK1,LAMB1,SDCBP,PRKG1,EGFR,BCR,MAGI2,LARGE1,FUT9,DOCK10,ENPEP,AIMP1,LDB2,NRG3,ATP8A1,ABL1,PTPRG,JAM2,SEMA6D,FER,PAK5,IL17RA,MCC,NFATC2,FAT3,NTNG1,LRP12,MAPRE2,FGF10,VCL,VCAM1,RAPGEF2,INSR,SRGAP3,UNC5D,FAT1,ITGA9,PPM1F,ASTN1,GFRA1,SEMA5A,KITLG,ARHGAP24,MAP4K4,CTNNA2,APC,GPC6,DNER,RELN,AUTS2,PPP2R3A,SNAI2,TNR,AKT3,VAV3,IL1R1,BCAS3,PRKCA,FMN2,APP,ASB2,ADGRB1,CCDC88A,SPOCK1*
GO:0035556	intracellular signal transduction	1.2308730856421943e-11	*ERBB4,APBB2,PAK1,TRIO,NRP1,TEAD1,CDH13,RC3H1,RPS6KA5,EPS8,TAOK3,ABL2,PRKCB,ARHGEF7,ARHGEF12,SLC8A1,LNPEP,ANKRD6,RERG,HTR2C,CNKSR3,SEMA3A,STARD13,LIMD1,PIK3C3,DCLK1,MAGI3,FGD4,GRIK2,RGL1,SIPA1L2,RCAN2,PJA2,ASB3,NEK6,MOB1B,ARHGAP28,DISC1,RALGPS1,ARHGAP42,KSR1,EDAR,FRMD6,FYN,CREBBP,RGS7,FHIT,GRIN2B,SRGAP2,STK32A,SPRED1,MYO5A,FGF12,DGKB,GARNL3,NOTCH2,MADD,ESR1,NFAT5,PDE4D,ARHGAP26,PRKACB,HIPK3,WSB1,SGMS1,JCAD,DOCK4,MAST4,NRXN1,NTRK3,WWOX,RAPGEF4,CNKSR2,NEK10,HMGA2,PHLPP1,ZDHHC17,PKN2,ROCK1,DCDC1,DOCK1,CACNA1C,ARHGAP31,UBR1,PRKAA2,ARHGEF11,GRB10,ARHGAP32,PRKN,SIAH2,LATS2,DOCK9,RORA,CDC14B,HDAC4,DGKG,MYO9A,FOXN3,NTRK2,PLCB1,MAPK1,SDCBP,CUL5,PRKG1,DGKI,NLK,EGFR,BCR,MAGI2,AKAP6,ARHGEF28,PLCL1,MOK,IQCJ-SCHIP1,SCN2A,RNF152,DOCK10,ITPKB,GRM5,NRG3,NLGN1,ABL1,NCOR1,ELMO1,LRP2,PPM1L,FER,PAK5,RYR2,NFATC2,MAPRE2,FGF10,NOS1AP,SGCD,RC3H2,STK32B,SEZ6L,VCAM1,RAPGEF2,SHANK2,ZMYND11,RAPGEF5,INSR,SRGAP3,PDK1,AKAP13,DENND4C,ARFGEF1,PATJ,PPM1F,DENND2B,MAPKBP1,SEMA5A,KITLG,ARHGAP24,MAP4K4,DOCK3,APC,ADGRV1,RELN,AKAP11,AUTS2,ROR1,SNAI2,NDFIP1,AKT3,VAV3,PRKCA,INPP5A,USP8,FMN2,ERC1,APP,RPS6KA3,SMAD5,PSD3,ASB2,MAPK8IP1,CCDC*

			88A,RAP1GDS1
GO:0051128	regulation of cellular component organization	1.697598478860012e-11	*MACF1,PAK1,SCAF8,KIRREL1,AMBRA1,SYNE2,MYO3B,NRP1,TEAD1,CDH13,RPS6KA5,EPS8,LRFN5,ABL2,SLC23A2,ARHGEF7,RERG,DYSF,STAU2,FYCO1,TBC1D5,SEMA3A,LRRC4C,CEP120,CDC42EP3,SDC2,CLSTN2,ADGRL2,TANC1,CORO2B,VPS41,MYO10,TOM1L2,ATP10A,NEK6,SLC39A12,ARHGAP28,DISC1,HECW2,FMN1,PEAK1,SVIL,ASAP1,FRMPD4,FYN,CSMD3,SLIT3,GRIN2B,ANTXR1,ARSB,TRABD2B,NEGR1,DGKB,KANSL1,BTBD9,TPM1,TOX,SRGAP2C,TMOD2,TJP1,ERC2,PTPRT,RIMS1,FCHSD2,LAMA1,NRXN1,NTRK3,WASF3,ARID1B,WDPCP,CTTNBP2,FHOD3,GRID1,GSG1L,ROCK1,PRKAA2,ARHGEF11,DNM3,SYT1,ARHGAP32,PRKN,DPF3,SACS,PTPRD,ADGRB3,DGKG,MYO9A,MYO3A,NTRK2,PLCB1,MAPK1,LAMB1,SDCBP,DSCAM,DIP2B,EGFR,PPFIA2,STXBP6,BCR,MAGI2,AKAP6,MELTF,FUT9,ANKFY1,IQCJ-SCHIP1,CNTN1,TENM3,SPIRE1,SNX30,NRG3,ATP8A1,PID1,NLGN1,ABL1,PTPRG,GRID2,SEMA6D,FER,PAK5,SETDB2,NFATC2,FAT3,NTNG1,MTPN,MTMR2,MAPRE2,PDE4DIP,MPDZ,YLPM1,VCL,RAPGEF2,SHANK2,INSR,AKAP13,SYNJ1,ARFGEF1,PATJ,ENPP1,ULK2,RIMS2,RHPN2,PPM1F,SEMA5A,ATP8A2,ARHGAP24,MAP4K4,CTNNA2,APC,RELN,VPS13C,AUTS2,ROR1,MTMR3,BMP2K,SNAI2,CYFIP2,TNR,PTPN13,SNAP91,BCAS3,USP8,USH1C,APP,PDLIM5,RPS6KA3,ABCA13,ADGRB1,CCDC88A,RAP1GDS1,SPOCK1*
GO:0007420	brain development	3.6708179356983265e-11	*ERBB4,RTN1,SETD2,SYNE2,NRP1,EML1,KCNC1,CNTN4,SEMA3A,CADM2,DCLK1,NIPBL,CEP120,KDM4B,ADGRL2,PBX1,PHACTR1,TTC21B,DISC1,FYN,SLC1A2,GRIN2B,SRGAP2,GRIA1,TOX,SRGAP2C,ZSWIM6,GLI3,NRXN1,HYDIN,PBX3,RERE,SLC4A10,HERC1,BBS2,ARHGAP32,FOXP2,RORA,NTRK2,PLCB1,LAMB1,BPTF,PRKG1,NFIB,EGFR,BCR,LARGE1,SCN2A,CNTN1,MED1,CA10,NRG3,ABL1,GRID2,LRP2,SEMA6D,MTPN,FGF10,SOX6,PCDH9,RAPGEF2,IMMP2L,SYNJ1,ATXN1,ZFHX3,SEMA5A,CTNNA2,RELN,MACROD2,TNR,AKT3,DNAH5,APP,SLC6A3,GHRH,ARNT2*
GO:0030029	actin filament-based process	5.3439470999962476e-11	*PAK1,FRMD3,KIRREL1,SYNE2,MYO3B,NRP1,EPS8,PHACTR3,STAU2,FRMD5,STARD13,CDC42BPA,CDC42EP3,HMCN1,FGD4,MYO5C,CORO2B,NEBL,FAM171A1,PHACTR1,ARHGAP28,FMN1,SVIL,FRMPD4,FRMD6,SH3KBP1,SRGAP2,MYO5A,FGF12,TPM1,FLNB,PCDH15,TMOD2,PDE4D,TJP1,ARHGAP26,FCHSD2,WASF3,FHOD3,MYO1E,ABCC9,ROCK1,CACNA1C,ARHGEF11,PRKN,TRPM7,MYO3A,SDCBP,PRKG1,BCR,SPIRE1,PHACTR2,ABL1,MICAL3,ELMO1,FER,RYR2,CACNB2,MTPN,NOS1AP,SGCD,AKAP13,ARFGEF1,FAT1,RHPN2,PPM1F,PGM5,SEMA5A,CTNNA2,AKAP11,CYFIP2,BCAS3,USH1C,FMN2,PDLIM5,CCDC88A,RAP1GDS1*
GO:000681	transport	5.63345824	*SLMAP,ERBB4,KCNMA1,CUBN,MACF1,SLC17A1*

0		450553e-11	,*PAK1,SLC6A11,ATP9B,SETD2,SLC15A5,SYNE2,EPN2,DOP1B,NRP1,CDH13,TANGO2,HERC2,ABL2,PRKCB,SLC23A2,ARHGEF7,UTRN,SLC8A1,LYPLA1,SLC2A13,HTR2C,KCNC1,DYSF,STAU2,CACNA1E,SIAH3,TMEM63C,FYCO1,TBC1D5,EXOC6B,AFG3L2,CNKSR3,PLIN2,SLC8A3,RFX3,RBFOX1,SLC40A1,SLCO3A1,ATP11C,PIK3C3,DCLK1,DNAH11,SLC9C1,ADAMTS9,CEP120,GRIK2,SNX25,CCDC186,MYO5C,KIF16B,NKAIN3,SLC25A21,EVI5,VPS41,GRIK4,MYO10,TAFA4,GABRG1,TOM1L2,IREB2,ATP10A,TTC21B,ABCD2,SLC39A12,COG2,CLTCL1,SV2B,HECW2,PIEZO2,SLC35F1,GABRB1,TMEM241,APBA2,LYST,SLC16A9,PLEKHA3,ATP2B2,FYN,SH3KBP1,NALCN,SLC1A2,CADPS,RGS7,SGTB,GRIN2B,ANTXR1,LRP1B,ARSB,GRIA1,AP5M1,MYO5A,FGF12,CACNG2,BTBD9,NPIPA1,CLDN10,GABRG3,ABCB5,SLC44A5,MEGF11,PDE4D,ERC2,RIMS1,FCHSD2,GLI3,ITPR2,NRXN1,FRMD4A,LONP2,TRAPPC10,WDPCP,RAPGEF4,EHBP1,NEK10,DPP6,PRELID2,GRID1,CACNA2D3,SEL1L,SLC4A10,SYN2,MYO1E,SLC7A2,ZDHHC17,ITGA4,ABCC9,GSG1L,ROCK1,DOCK1,CACNA1C,AMPH,RGPD4,SORCS1,DNM3,SYT1,BBS2,GRB10,SLC27A6,PRKN,TRPM7,EXOC4,DLG2,SCFD2,BBS9,PTPRN2,RANBP3L,TG,NTRK2,PLCB1,MAPK1,SDCBP,ATP9A,GRIK1,CUL5,PRKG1,DGKI,EGFR,PPFIA2,STXBP6,CLCN5,BCR,MAGI2,AKAP6,LARGE1,TRAPPC8,STX12,MELTF,TMPRSS3,ANKFY1,TBC1D1,SLC37A2,SLC12A1,SCN2A,CNTN1,GRM5,MED1,GNPTAB,SPIRE1,AIMP1,SNX30,ATP8A1,PID1,NLGN1,ABL1,MICAL3,GRID2,ELMO1,LRP2,RANBP17,ERBIN,FER,MON2,RYR2,SCAMP1,LDLRAD3,PRLR,DST,GRIK3,CACNB2,STXBP4,MTMR2,KCNH5,LRP12,LRRC8B,FGF10,KIF13A,NOS1AP,TRPM3,IPO11,ZDHHC21,XKR6,IFT81,INSR,UNC80,WDR41,ILDR2,IMMP2L,SYNJ1,KLF15,DENND4C,ATXN1,GABRG2,ARFGEF1,ENPP1,UNC13C,TANGO6,NRXN3,RIMS2,PPM1F,KCNQ3,SCN8A,SNX8,NSG1,CPE,KCND2,ATP8A2,SLC24A3,SV2C,GRIA4,MAP4K4,CNIH3,DNER,ABCA5,RELN,VPS13C,KCNJ15,CYBRD1,STON1-GTF2A1L,BMP2K,SLC4A4,NDFIP1,SNAP91,OCA2,VAV3,FCHO2,RFTN2,DNAH5,KCNS3,FMN2,ERC1,APP,SLC6A3,DNAJC13,VPS13B,ABCA13,EYA2,MAPK8IP1,ADGRB1,CCDC88A,GHRH,RAP1GDS1*
GO:0048870	cell motility	9.966940485489741e-11	*FSTL1,ERBB4,MACF1,PAK1,MITF,SETD2,SYNE2,NRP1,CDH13,EPS8,ABL2,ARHGEF7,GPC5,DACH1,FRMD5,LDLRAD4,SEMA3A,RFX3,MGAT5,STARD13,LIMD1,DCLK1,DNAH11,SLC9C1,ADAMTS9,CDC42BPA,NIPBL,SDC2,TAFA4,PHACTR1,DISC1,PEAK1,LYST,FYN,SH3KBP1,AGO2,SRGAP2,ARSB,TAFA5,SPRED1,TPM1,SRGAP2C,TJP1,PTPRT,LAMA1,JCAD,GLI3,DOCK4,NTRK3,MTUS1,WDPCP,RERE,PKN2,ITGA4,ROCK1,DOCK1,BBS2,GRB10,ARHGAP32,NTRK2,PLCB1,MAPK1,LAMB1,SDCBP,PRKG1,EGFR,BCR,MAG*

			I2,LARGE1,FUT9,DOCK10,ENPEP,AIMP1,LDB2,NRG3,ATP8A1,ABL1,PTPRG,JAM2,ELMO1,SEMA6D,FER,PAK5,IL17RA,MCC,NFATC2,DST,FAT3,NTNG1,LRP12,MAPRE2,FGF10,VCL,VCAM1,RAPGEF2,IFT81,INSR,SRGAP3,UNC5D,FAT1,ITGA9,PPM1F,ASTN1,GFRA1,SEMA5A,KITLG,ARHGAP24,MAP4K4,CTNNA2,APC,GPC6,DNER,RELN,AUTS2,PPP2R3A,SNAI2,TNR,AKT3,VAV3,IL1R1,BCAS3,PRKCA,DNAH5,FMN2,APP,ASB2,ADGRB1,CCDC88A,SPOCK1
GO:0051234	establishment of localization	1.3311737153378688e-10	SLMAP,ERBB4,KCNMA1,CUBN,MACF1,SLC17A1,PAK1,SLC6A11,ATP9B,SETD2,SLC15A5,SYNE2,EPN2,DOP1B,NRP1,CDH13,TANGO2,HERC2,ABL2,PRKCB,SLC23A2,ARHGEF7,UTRN,SLC8A1,LYPLA1,SLC2A13,HTR2C,KCNC1,DYSF,STAU2,CACNA1E,SIAH3,TMEM63C,FYCO1,TBC1D5,EXOC6B,AFG3L2,CNKSR3,PLIN2,SLC8A3,RFX3,RBFOX1,SLC40A1,SLCO3A1,ATP11C,PIK3C3,DCLK1,DNAH11,SLC9C1,ADAMTS9,NIPBL,CEP120,GRIK2,SNX25,CCDC186,MYO5C,KIF16B,NKAIN3,SLC25A21,CORO2B,EVI5,ITGA8,VPS41,GRIK4,MYO10,TAFA4,GABRG1,TOM1L2,IREB2,ATP10A,TTC21B,ABCD2,SLC39A12,COG2,CLTCL1,SV2B,HECW2,PIEZO2,SLC35F1,GABRB1,TMEM241,APBA2,LYST,SLC16A9,PLEKHA3,ATP2B2,FYN,SH3KBP1,NALCN,SLC1A2,CADPS,RGS7,SGTB,GRIN2B,ANTXR1,LRP1B,ARSB,GRIA1,AP5M1,MYO5A,FGF12,CACNG2,BTBD9,NPIPA1,CLDN10,GABRG3,ABCB5,SLC44A5,MEGF11,PDE4D,ERC2,RIMS1,FCHSD2,GLI3,ITPR2,NRXN1,FRMD4A,LONP2,TRAPPC10,WDPCP,RAPGEF4,EHBP1,NEK10,DPP6,PRELID2,GRID1,CACNA2D3,SEL1L,SLC4A10,SYN2,MYO1E,SLC7A2,ZDHHC17,ITGA4,ABCC9,GSG1L,ROCK1,DOCK1,CACNA1C,AMPH,RGPD4,SORCS1,DNM3,SYT1,BBS2,GRB10,SLC27A6,PRKN,TRPM7,EXOC4,DLG2,SCFD2,BBS9,PTPRN2,RANBP3L,TG,NTRK2,PLCB1,MAPK1,SDCBP,ATP9A,GRIK1,CUL5,PRKG1,DGKI,EGFR,PPFIA2,STXBP6,CLCN5,BCR,MAGI2,AKAP6,LARGE1,TRAPPC8,STX12,MELTF,TMPRSS3,ANKFY1,TBC1D1,SLC37A2,SLC12A1,SCN2A,CNTN1,GRM5,MED1,GNPTAB,SPIRE1,AIMP1,SNX30,ATP8A1,PID1,NLGN1,ABL1,MICAL3,GRID2,ELMO1,LRP2,RANBP17,ERBIN,FER,MON2,RYR2,SCAMP1,LDLRAD3,MCC,PRLR,DST,GRIK3,CACNB2,STXBP4,MTMR2,KCNH5,LRP12,LRRC8B,FGF10,KIF13A,NOS1AP,TRPM3,IPO11,ZDHHC21,XKR6,IFT81,INSR,UNC80,WDR41,ILDR2,IMMP2L,SYNJ1,KLF15,DENND4C,ATXN1,GABRG2,ARFGEF1,ENPP1,UNC13C,TANGO6,NRXN3,RIMS2,PPM1F,KCNQ3,SCN8A,SNX8,NSG1,CPE,KCND2,ATP8A2,SLC24A3,SV2C,GRIA4,MAP4K4,CNIH3,APC,PARD3B,DNER,ABCA5,ADGRV1,RELN,VPS13C,KCNJ15,CYBRD1,STON1-GTF2A1L,BMP2K,SLC4A4,NDFIP1,SNAP91,OCA2,VAV3,FCHO2,RFTN2,DNAH5,KCNS3,FMN2,ERC1,APP,SLC6A3,DNAJC13,VPS13B,ABCA13,EYA2,MAPK8IP1,ADGRB1,CCDC88A,GHRH,RA

			P1GDS1
GO:0060322	head development	3.5232255703319106e-10	*ERBB4,RTN1,SETD2,SYNE2,NRP1,EML1,KCNC1,CNTN4,SEMA3A,CADM2,DCLK1,NIPBL,CEP120,KDM4B,ADGRL2,PBX1,PHACTR1,TTC21B,DISC1,FYN,SLC1A2,GRIN2B,SRGAP2,GRIA1,TOX,SRGAP2C,ZSWIM6,GLI3,NRXN1,HYDIN,PBX3,RERE,SLC4A10,HERC1,BBS2,ARHGAP32,FOXP2,RORA,NTRK2,PLCB1,MAPK1,LAMB1,BPTF,PRKG1,NFIB,EGFR,BCR,LARGE1,SCN2A,CNTN1,MED1,CA10,NRG3,ABL1,GRID2,LRP2,SEMA6D,MTPN,FGF10,SOX6,PCDH9,RAPGEF2,IMMP2L,SYNJ1,ATXN1,ZFHX3,SEMA5A,CTNNA2,RELN,MACROD2,TNR,AKT3,DNAH5,APP,SLC6A3,GHRH,ARNT2*
GO:0007610	behavior	1.1048862104561557e-9	*EPS8,ABL2,DACH1,HTR2C,SLC8A3,DNAH11,GRIK2,MBD5,CLSTN2,TANC1,ITGA8,PJA2,TTC21B,APBA2,FYN,SLC1A2,GRIN2B,GRIA1,NEGR1,NAV2,FGF12,BTBD9,PCDH15,TMOD2,GLI3,BRINP1,NRXN1,PIAS1,PBX3,GRID1,SLC4A10,BBS2,PRKN,ADGRB3,NTRK2,PLCB1,MAPK1,DSCAM,DGKI,EGFR,LARGE1,SORCS3,SCN2A,CNTN1,GRM5,ATP8A1,NLGN1,ABL1,NCOR1,PAK5,GNG7,SHANK2,INSR,SYNJ1,ATXN1,GABRG2,NRXN3,KCNQ3,ZFHX3,ASTN1,KCND2,ATP8A2,RELN,TNR,APP,SLC6A3,GHRH*
GO:0008283	cell population proliferation	1.1547572171847763e-9	*HPSE2,ERBB4,MACF1,PAK1,CTNND2,TRIO,MITF,AMBRA1,SYNE2,MYO3B,NRP1,NFIA,FRY,CDH13,RC3H1,RPS6KA5,EPS8,TAOK3,ABL2,SLC23A2,ARHGEF7,DACH1,EML1,RERG,STAU2,CNTN4,AFG3L2,SEMA3A,RFX3,MMP16,LRRC4C,ALCAM,DCLK1,CEP120,CDC42EP3,FGD4,SDC2,NCAM1,TANC1,ITGA8,MYO10,PBX1,PHACTR1,BICRAL,TTC21B,ABCD2,SLC39A12,DISC1,HECW2,EYA1,KSR1,ASAP1,ST8SIA1,FYN,ZBTB16,CSMD3,B4GALT6,SLIT3,GRIN2B,SRGAP2,ARSB,TAFA5,NEGR1,TPM1,NOTCH2,TOX,PCDH15,ESR1,SRGAP2C,TJP1,GNG2,RIMS1,PARVB,LAMA1,JCAD,GLI3,NRXN1,NTRK3,WASF3,HYDIN,TSPAN2,WDPCP,PIAS1,HMGA2,RERE,ATL1,ZDHHC17,PKN2,ITGA4,ROCK1,HERC1,CFAP70,DNM3,SYT1,BBS2,ARHGAP32,PRKN,RORA,PTPRD,CDC14B,BBS9,HDAC4,ADGRB3,DGKG,MYO9A,MYO3A,NTRK2,MAPK1,LAMB1,SDCBP,ATP9A,SOX5,PRKG1,DSCAM,BIRC6,NFIB,DIP2B,EGFR,PPFIA2,NCAM2,MAGI2,NELL1,RTTN,ARHGEF28,FUT9,CNTN1,ZNRF3,DOCK10,TENM3,ITPKB,MED1,ENPEP,EPHA6,AIMP1,PID1,NLGN1,ABL1,PTPRG,JARID2,GRID2,LRP2,SEMA6D,FER,PAK5,MCC,PRLR,NFATC2,FAT3,STXBP4,NTNG1,VSTM2A,MTMR2,LRP12,FGF10,RC3H2,VCL,VCAM1,RAPGEF2,IFT81,SHANK2,INSR,PDK1,UNC5D,ULK2,NRXN3,RIMS2,EGLN3,GFRA1,BMPR1B,SEMA5A,ATP8A2,KITLG,ARHGAP24,MAP4K4,CTNNA2,PRAME,APC,ADGRV1,RELN,AUTS2,ROR1,SNAI2,CYFIP2,ADAMTSL1,NDFIP1,TNR,OCA2,AKT3,VAV3,PRKCA,USH1C,DNAH5,CNTN6,ENPP3,APP,PDLIM5,NYAP2,VPS13B,MAPK8IP1,ADGRB1,CCDC88A,GHRH,SPOCK1*

GO:0031344	regulation of cell projection organization	1.3681577323394312e-9	*MACF1,PAK1,SYNE2,MYO3B,NRP1,EPS8,ABL2,ARHGEF7,STAU2,SEMA3A,LRRC4C,CEP120,CDC42EP3,SDC2,MYO10,SLC39A12,DISC1,HECW2,FYN,CSMD3,GRIN2B,ARSB,NEGR1,TOX,SRGAP2C,NRXN1,NTRK3,WDPCP,DNM3,ARHGAP32,PTPRD,ADGRB3,DGKG,MYO9A,MYO3A,NTRK2,DSCAM,DIP2B,PPFIA2,MAGI2,FUT9,CNTN1,TENM3,NLGN1,ABL1,PTPRG,GRID2,SEMA6D,FER,FAT3,NTNG1,RAPGEF2,ULK2,SEMA5A,ATP8A2,ARHGAP24,MAP4K4,CTNNA2,APC,RELN,AUTS2,ROR1,TNR,USH1C,PDLIM5,CCDC88A,SPOCK1*
GO:0009966	regulation of signal transduction	1.3869486899101463e-9	*FSTL1,ERBB4,MACF1,PAK1,CTNND2,TRIO,EPN2,NETO2,EVC,NRP1,CDH13,RC3H1,EPS8,TAOK3,ABL2,PRKCB,GPC5,ARHGEF12,ANKRD6,HTR2C,CNKSR3,LDLRAD4,SEMA3A,SLC8A3,MGAT5,STARD13,LIMD1,FGD4,GRIK2,SNX25,IGSF11,MBD5,NCAM1,SIPA1L2,ITGA8,PJA2,TAFA4,TTC21B,NEK6,ARHGAP28,DISC1,RALGPS1,ARHGAP42,EYA1,KSR1,EDAR,FRMD6,RGS20,FYN,CREBBP,DLGAP1,SLIT3,RGS7,GRIN2B,SRGAP2,TRABD2B,SPRED1,CACNG2,GARNL3,CRIM1,NOTCH2,MADD,ESR1,NFAT5,TMOD2,PDE4D,ARHGAP26,PTPRT,PRKACB,RIMS1,HIPK3,SGMS1,LAMA1,JCAD,GLI3,NRXN1,NTRK3,FBN1,WWOX,MOSMO,CNKSR2,NEK10,PHLPP1,ZDHHC17,GSG1L,ROCK1,ARHGAP31,UBR1,PRKAA2,ARHGEF11,GRB10,ARHGAP32,PRKN,SIAH2,LATS2,RORA,PTPRD,DGKG,MYO9A,NTRK2,PLCB1,MAPK1,LAMB1,SDCBP,BIRC6,DGKI,NLK,EGFR,BCR,MAGI2,AKAP6,ARHGEF28,IQCJ-SCHIP1,RNF152,ZNRF3,GNAQ,ITPKB,GRM5,MED1,PID1,NLGN1,ABL1,NCOR1,LRP2,ERBIN,FER,PAK5,FBXL17,MCC,PRLR,GNG7,MTMR2,MAPRE2,FGF10,NOS1AP,CADM1,RC3H2,RIC8B,ATF6,SEZ6L,RAPGEF2,RGS12,IFT81,SHANK2,ZMYND11,INSR,SRGAP3,AKAP13,KLF15,DENND4C,ARFGEF1,ENPP1,RIMS2,DENND2B,DLGAP2,BMPR1B,MAPKBP1,SEMA5A,KITLG,ARHGAP24,MAP4K4,CNIH3,DOCK3,PRAME,APC,GPC6,ADGRV1,RELN,AUTS2,ROR1,PPP2R3A,BMP2K,SNAI2,CYFIP2,NDFIP1,CELF4,AKT3,VAV3,IL1R1,PRKCA,USP8,CNTN6,APP,SMAD5,PSD3,ALPK2,EYA2,MAPK8IP1,CCDC88A,GHRH,RAP1GDS1*
GO:0120035	regulation of plasma membrane bounded cell projection organization	1.4163310898375232e-9	*MACF1,PAK1,SYNE2,MYO3B,NRP1,EPS8,ABL2,ARHGEF7,STAU2,SEMA3A,LRRC4C,CEP120,CDC42EP3,SDC2,MYO10,SLC39A12,DISC1,HECW2,FYN,CSMD3,GRIN2B,ARSB,NEGR1,TOX,SRGAP2C,NRXN1,NTRK3,WDPCP,DNM3,ARHGAP32,PTPRD,ADGRB3,DGKG,MYO3A,NTRK2,DSCAM,DIP2B,PPFIA2,MAGI2,FUT9,CNTN1,TENM3,NLGN1,ABL1,PTPRG,GRID2,SEMA6D,FER,FAT3,NTNG1,RAPGEF2,ULK2,SEMA5A,ATP8A2,ARHGAP24,MAP4K4,CTNNA2,APC,RELN,AUTS2,ROR1,TNR,USH1C,PDLIM5,CCDC88A,SPOCK1*
GO:0007264	small GTPase	1.9549121953819947e-	*TRIO,NRP1,CDH13,EPS8,ABL2,ARHGEF12,RERG,STARD13,FGD4,RGL1,SIPA1L2,ARHGAP28,RALGPS1,ARHGAP42,KSR1,SRGAP2,GARNL3,*

	mediated signal transduction	9	*NOTCH2,MADD,ARHGAP26,DOCK4,RAPGEF4,ROCK1,DOCK1,ARHGAP31,ARHGEF11,ARHGAP32,SIAH2,DOCK9,MYO9A,SDCBP,DGKI,BCR,ARHGEF28,DOCK10,ITPKB,ABL1,ELMO1,MAPRE2,FGF10,RAPGEF2,RAPGEF5,SRGAP3,AKAP13,DENND4C,ARFGEF1,KITLG,ARHGAP24,MAP4K4,DOCK3,RELN,AUTS2,VAV3,USP8,PSD3,CCDC88A*
GO:0030036	actin cytoskeleton organization	6.256648777188123e-9	*PAK1,FRMD3,KIRREL1,MYO3B,NRP1,EPS8,PHACTR3,STAU2,FRMD5,STARD13,CDC42BPA,CDC42EP3,HMCN1,FGD4,MYO5C,CORO2B,NEBL,FAM171A1,PHACTR1,ARHGAP28,FMN1,SVIL,FRMPD4,SH3KBP1,MYO5A,TPM1,FLNB,PCDH15,TMOD2,TJP1,ARHGAP26,FCHSD2,WASF3,FHOD3,MYO1E,ROCK1,ARHGEF11,PRKN,TRPM7,MYO3A,SDCBP,PRKG1,BCR,SPIRE1,PHACTR2,ABL1,MICAL3,ELMO1,FER,MTPN,NOS1AP,AKAP13,ARFGEF1,FAT1,RHPN2,PPM1F,PGM5,SEMA5A,CTNNA2,AKAP11,CYFIP2,BCAS3,USH1C,FMN2,PDLIM5,CCDC88A,RAP1GDS1*
GO:0003008	system process	6.607549872963021e-9	*SLMAP,KCNMA1,PDE6A,APBB2,KIRREL1,NETO2,DOP1B,MYO3B,OR4C46,UTRN,SLC8A1,LNPEP,SLC2A13,HTR2C,TMEM63C,SLC8A3,RBFOX1,SLCO3A1,DNAH11,NIPBL,HMCN1,GRIK2,IGSF11,CLSTN2,TANC1,CORO2B,ITGA8,PJA2,ASB3,TAFA4,GABRG1,IREB2,ARHGAP42,EYA1,RORB,PIEZO2,GABRB1,ATP2B2,FYN,SLC1A2,DLGAP1,GRIN2B,GRIA1,NAV2,FGF12,CACNG2,BTBD9,TPM1,PCDH15,GABRG3,TMOD2,PDE4D,TJP1,LRIG1,PRKACB,RIMS1,DOCK4,BRINP1,NRXN1,SGCG,WASF3,NEK10,PIAS1,PBX3,SLC4A10,MYO1E,SLC7A2,ABCC9,ROCK1,HERC1,CACNA1C,ARHGEF11,BBS2,PRKN,PPP1R12B,BBS9,HDAC4,ADGRB3,MYO9A,MYO3A,NTRK2,PLCB1,MAPK1,PRKG1,DGKI,EGFR,PTPRQ,CLCN5,BCR,AKAP6,LARGE1,TMPRSS3,SORCS3,SCN2A,POU6F2,GRM5,ENPEP,ATP8A1,NLGN1,ABL1,JARID2,OR9Q1,JAM2,GRID2,LRP2,PAK5,RYR2,CACNB2,MTPN,MTMR2,FGF10,NOS1AP,SGCD,ATF6,EYS,ZDHHC21,SHANK2,INSR,AKAP13,IMMP2L,PPIP5K2,SYNJ1,KLF15,ATXN1,GABRG2,NRXN3,RIMS2,RHPN2,KCNQ3,SCN8A,DLGAP2,KCND2,ATP8A2,SLC24A3,LHFPL3,CTNNA2,ADGRV1,RELN,AKAP11,ROR1,SNAI2,SLC4A4,TNR,DTNA,CELF4,CRB1,PRKCA,USH1C,APP,PDLIM5,SMAD5,SLC6A3,ASB2,RAP1GDS1*
GO:0050896	response to stimulus	7.662408963409832e-9	*FSTL1,ERBB4,KCNMA1,PDE6A,APBB2,FYB2,CUBN,MACF1,PAK1,CTNND2,SLC6A11,TRIO,MITF,SETD2,AMBRA1,EPN2,NETO2,PDE1C,MYO3B,EVC,NRP1,TEAD1,NFIA,CDH13,RC3H1,RPS6KA5,EPS8,TAOK3,HERC2,LRFN5,OR4C46,ABL2,PRKCB,SLC23A2,ARHGEF7,GPC5,MAML2,ARHGEF12,SAMD12,SLC8A1,DACH1,LNPEP,ANKRD6,RERG,HTR2C,KCNC1,DYSF,STAU2,NEK4,TBC1D5,USP14,AFG3L2,CNKSR3,LDLRAD4,SEMA3A,PLIN2,SLC8A3,MGAT5,STARD13,SLC40A1,ALCAM,ERLIN2,LIMD1,PIK3C3,DCLK1,ADAMTS9,MAGI3,NIPBL,CDC42EP3,HMCN1,FGD4,GRIK2,SNX25,IGSF11,RGL1,CCDC186,MBD5*

			,KIF16B,NCAM1,TCF12,MARCHF6,SIPA1L2,RCAN2,ADGRL2,GNG12,TANC1,CORO2B,PAPPA,ITGA8,SYCP1,VPS41,GRIK4,ZBTB20,MYO10,PJA2,ASB3,TAFA4,NAALADL2,GABRG1,TOM1L2,TTC21B,NEK6,ABCD2,SLC39A12,MOB1B,ARHGAP28,DISC1,RALGPS1,ARHGAP42,EYA1,KSR1,RORB,PIEZO2,GPR158,ZNF236,GABRB1,ANKS1B,LYST,EDAR,ST8SIA1,FRMD6,RGS20,FYN,CREBBP,SLC1A2,DLGAP1,SLIT3,RGS7,FHIT,GRIN2B,SRGAP2,STK32A,ARSB,GRIA1,TRABD2B,TAFA5,SPRED1,MYO5A,SPEN,FGF12,DGKB,CACNG2,GARNL3,TPM1,CRIM1,NOTCH2,FLNB,MADD,PCDH15,ESR1,GABRG3,FKBP5,NFAT5,TMOD2,PLCXD3,PDE4D,TJP1,ARHGAP26,PTPRT,PRKACB,GNG2,TRHDE,RIMS1,HIPK3,WSB1,SGMS1,LAMA1,JCAD,GLI3,DOCK4,MAST4,GAST,ITPR2,BRINP1,NRXN1,SUPT16H,NTRK3,FBN1,WWOX,LONP2,ARID1B,MTUS1,TSPAN2,WDPCP,MAGI1,MOSMO,RAPGEF4,CNKSR2,NEK10,PIAS1,PRICKLE2,HMGA2,HHAT,GRID1,PARP8,SEL1L,SLC4A10,PHLPP1,MYO1E,ZDHHC17,PKN2,ITGA4,ABCC9,GSG1L,ROCK1,DCDC1,DOCK1,CACNA1C,ARHGAP31,UBR1,RXRG,PRKAA2,ARHGEF11,SORCS1,COL4A2,PACRG,SYT1,BBS2,GRB10,ARHGAP32,PRKN,DPF3,SIAH2,LATS2,DOCK9,DLG2,PPP1R12B,RORA,PTPRD,CDC14B,TTR,BBS9,PTPRN2,TG,HDAC4,ADGRB3,INPP4B,DGKG,MYO9A,MYO3A,FOXN3,NTRK2,PLCB1,MAPK1,LAMB1,SDCBP,BPTF,GRIK1,CUL5,SOX5,PRKG1,DSCAM,BIRC6,DGKI,NFIB,NLK,EGFR,PTPRQ,BCR,MAGI2,AKAP6,LARGE1,FANCM,ARHGEF28,PLCL1,MOK,PXDNL,TBC1D1,IQCJ-SCHIP1,SORCS3,SCN2A,ERP27,RNF152,CNTN1,ZNRF3,GNAQ,AGL,DOCK10,TENM3,ITPKB,GRM5,MED1,EPHA6,SPIRE1,ARAP2,AIMP1,NRG3,CPEB4,PID1,NLGN1,ABL1,PTPRG,NCOR1,JARID2,OR9Q1,GRID2,ELMO1,LRP2,ERBIN,SEMA6D,PPM1L,CABIN1,MBTPS2,FER,PAK5,RYR2,IL17RA,FBXL17,MCC,GLYAT,SUSD4,PRLR,HADHB,NFATC2,DST,GRIK3,STXBP4,MTPN,VSTM2A,GNG7,MTMR2,LRP12,MAPRE2,FGF10,NOS1AP,SOX6,CADM1,SGCD,RC3H2,MSRA,RIC8B,ATF6,EYS,STK32B,VCL,ZNF106,SEZ6L,ZDHHC21,VCAM1,RAPGEF2,RGS12,IFT81,SHANK2,ZMYND11,RAPGEF5,INSR,SRGAP3,PDK1,AKAP13,WDR41,ILDR2,IMMP2L,CHCHD6,KLF15,DENND4C,UNC5D,GABRG2,ARFGEF1,PATJ,ITGA9,ENPP1,ULK2,NRXN3,RIMS2,RHPN2,PPM1F,KCNQ3,ZFHX3,DENND2B,EGLN3,DLGAP2,GFRA1,ATRNL1,GPR156,BMPR1B,NSG1,MAPKBP1,CPE,KCND2,FCRLA,SEMA5A,ATP8A2,KITLG,PYGO1,ARHGAP24,GRIA4,MAP4K4,CERS6,CNIH3,DOCK3,CTNNA2,PRAME,APC,APLF,GPC6,DNER,MARCHF1,ADGRV1,RELN,MACROD2,AKAP11,VPS13C,ANXA4,NAT1,CYBRD1,AUTS2,ROR1,PPP2R3A,MTMR3,BMP2K,SNAI2,CYFIP2,NDFIP1,TNR,PTPN13,DTNA,CELF4,AKT3,CRB1,VAV3,IL1R1,BCAS3,RFTN2,PRKCA,KMT2C,INPP5

			A,USP8,CNTN6,ENPP3,FMN2,ERC1,APP,RPS6KA3,SMAD5,SLC6A3,PSD3,ALPK2,ASB2,EYA2,MAPK8IP1,ADGRB1,CCDC88A,GHRH,ARNT2,RAP1GDS1,AFF3
GO:0042391	regulation of membrane potential	1.5816173162350133e-8	*SLMAP,KCNMA1,SLC8A1,KCNC1,SLC8A3,GRIK2,IGSF11,GRIK4,TAFA4,GABRG1,PIEZO2,GABRB1,NALCN,GRIN2B,GRIA1,FGF12,CACNG2,GABRG3,ABCB5,RIMS1,NRXN1,GRID1,CACNA1C,PRKN,NTRK2,GRIK1,DGKI,AKAP6,GNAQ,GRM5,PID1,NLGN1,ABL1,GRID2,RYR2,GRIK3,CACNB2,MTMR2,KCNH5,NOS1AP,GABRG2,RIMS2,KCNQ3,SCN8A,KCND2,GRIA4,RELN,SLC4A4,CELF4,APP*
GO:0007166	cell surface receptor signaling pathway	1.6531586029751337e-8	*FSTL1,ERBB4,FYB2,MACF1,PAK1,CTNND2,TRIO,MITF,EPN2,EVC,NRP1,NFIA,CDH13,RC3H1,RPS6KA5,PRKCB,ARHGEF7,GPC5,MAML2,SAMD12,LNPEP,ANKRD6,LDLRAD4,SEMA3A,SLC8A3,MGAT5,LIMD1,GRIK2,SNX25,IGSF11,MBD5,KIF16B,NCAM1,ADGRL2,PAPPA,ITGA8,GRIK4,PJA2,TTC21B,DISC1,EYA1,ANKS1B,FYN,CREBBP,SLIT3,GRIN2B,GRIA1,TRABD2B,SPRED1,SPEN,CRIM1,NOTCH2,MADD,PDE4D,TJP1,PTPRT,PRKACB,RIMS1,LAMA1,JCAD,GLI3,NRXN1,NTRK3,FBN1,WWOX,WDPCP,MAGI1,MOSMO,PIAS1,PRICKLE2,HHAT,GRID1,SEL1L,MYO1E,ZDHHC17,ITGA4,DOCK1,PRKAA2,COL4A2,BBS2,GRB10,PRKN,SIAH2,LATS2,RORA,PTPRD,HDAC4,ADGRB3,NTRK2,PLCB1,MAPK1,LAMB1,SDCBP,GRIK1,CUL5,DSCAM,BIRC6,DGKI,NLK,EGFR,MAGI2,ARHGEF28,CNTN1,ZNRF3,GNAQ,ITPKB,GRM5,MED1,EPHA6,NRG3,CPEB4,PID1,NLGN1,ABL1,PTPRG,GRID2,LRP2,ERBIN,SEMA6D,PPM1L,CABIN1,FER,PAK5,IL17RA,FBXL17,MCC,PRLR,NFATC2,DST,GRIK3,STXBP4,GNG7,MTMR2,FGF10,CADM1,RC3H2,ZNF106,RAPGEF2,IFT81,ZMYND11,INSR,KLF15,UNC5D,ITGA9,ENPP1,RIMS2,GFRA1,BMPR1B,CPE,FCRLA,SEMA5A,KITLG,PYGO1,GRIA4,APC,GPC6,DNER,ADGRV1,RELN,ANXA4,ROR1,PPP2R3A,BMP2K,SNAI2,CYFIP2,CELF4,VAV3,IL1R1,PRKCA,USP8,CNTN6,APP,SMAD5,ALPK2,EYA2,ADGRB1,CCDC88A,GHRH*
GO:0007409	axonogenesis	1.9641093661867267e-8	*MACF1,PAK1,TRIO,NRP1,RPS6KA5,CNTN4,AFG3L2,SEMA3A,LRRC4C,ALCAM,DCLK1,NCAM1,DISC1,FYN,B4GALT6,SLIT3,NOTCH2,GLI3,NRXN1,ATL1,ZDHHC17,ITGA4,ARHGAP32,PTPRD,NTRK2,PRKG1,DSCAM,NFIB,DIP2B,ARHGEF28,CNTN1,EPHA6,ABL1,SEMA6D,NTNG1,VCL,UNC5D,ULK2,NRXN3,BMPR1B,SEMA5A,ATP8A2,CTNNA2,RELN,AUTS2,CYFIP2,ADAMTSL1,TNR,CNTN6,APP,ADGRB1*
GO:0030334	regulation of cell migration	5.3793404845278074e-8	*ERBB4,MACF1,PAK1,MITF,SYNE2,NRP1,CDH13,ABL2,ARHGEF7,DACH1,FRMD5,LDLRAD4,SEMA3A,MGAT5,STARD13,ADAMTS9,NIPBL,PHACTR1,AGO2,SRGAP2,ARSB,TAFA5,SPRED1,TPM1,SRGAP2C,TJP1,PTPRT,LAMA1,JCAD,DOCK4,NTRK3,MTUS1,WDPCP,ITGA4,ROCK1,DOCK1,ARHGAP32,PLCB1,MAPK1,LAMB1,SDCBP,PRKG1,EGFR,BCR,MAGI2,FUT9,DOCK10,LDB2,NRG*

			3,ATP8A1,ABL1,PTPRG,JAM2,SEMA6D,FER,MCC,NTNG1,MAPRE2,FGF10,VCL,RAPGEF2,INSR,SRGAP3,UNC5D,PPM1F,SEMA5A,KITLG,MAP4K4,CTNNA2,APC,RELN,PPP2R3A,SNAI2,TNR,AKT3,IL1R1,BCAS3,PRKCA,APP,ADGRB1
GO:0010975	regulation of neuron projection development	6.515865395317228e-8	*MACF1,PAK1,NRP1,ABL2,STAU2,SEMA3A,LRRC4C,SDC2,SLC39A12,DISC1,HECW2,FYN,CSMD3,ARSB,NEGR1,TOX,NRXN1,NTRK3,DNM3,ARHGAP32,PTPRD,ADGRB3,DGKG,NTRK2,DSCAM,DIP2B,PPFIA2,MAGI2,FUT9,CNTN1,TENM3,NLGN1,ABL1,PTPRG,GRID2,SEMA6D,FAT3,NTNG1,RAPGEF2,ULK2,SEMA5A,ATP8A2,MAP4K4,CTNNA2,RELN,ROR1,TNR,PDLIM5,CCDC88A,SPOCK1*
GO:0007010	cytoskeleton organization	8.598314441337469e-8	*MACF1,PAK1,FRMD3,SETD2,KIRREL1,SYNE2,MYO3B,NRP1,EPS8,ARHGEF7,PHACTR3,EML1,STAU2,FRMD5,STARD13,LIMD1,DCLK1,CDC42BPA,CEP120,CDC42EP3,HMCN1,FGD4,MYO5C,CORO2B,NEBL,FAM171A1,PHACTR1,NEK6,SLC39A12,ARHGAP28,DISC1,FMN1,SVIL,TTLL7,FRMPD4,SH3KBP1,SRGAP2,MYO5A,TPM1,FLNB,PCDH15,SRGAP2C,TMOD2,TJP1,ARHGAP26,FCHSD2,GOLGA8S,MAST4,WASF3,HYDIN,WDPCP,FHOD3,MYO1E,ROCK1,PRKAA2,ARHGEF11,CCSER2,BBS2,PRKN,TRPM7,CDC14B,TLN2,MYO3A,MAPK1,SDCBP,PRKG1,BCR,LARGE1,RTTN,ARHGEF28,IQCJ-SCHIP1,SPIRE1,PHACTR2,NLGN1,ABL1,NCOR1,MICAL3,ELMO1,ERBIN,FER,PAK5,DST,MTPN,MAPRE2,FGF10,NOS1AP,PDE4DIP,MPDZ,AKAP13,ARFGEF1,FAT1,PATJ,RHPN2,PPM1F,PGM5,SEMA5A,ATP8A2,CTNNA2,APC,PARD3B,AKAP11,CYFIP2,BCAS3,USH1C,DNAH5,FMN2,PDLIM5,CCDC88A,RAP1GDS1*
GO:0098609	cell-cell adhesion	9.61471842082118e-8	*LPP,CTNND2,KIRREL1,AMBRA1,CDH13,RC3H1,LRFN5,ABL2,CNTN4,PCDH7,LRRC4C,CADM2,ALCAM,HMCN1,IGSF11,CLSTN2,ITGA8,MYO10,FYN,ZBTB16,NEGR1,COL19A1,CLDN10,PCDH15,NFAT5,MEGF11,TJP1,PTPRT,GLI3,NRXN1,ARID1B,MAGI1,CDH20,ITGA4,ROCK1,PTPRD,TLN2,LAMB1,PRKG1,DSCAM,EGFR,STXBP6,NCAM2,FUT9,CNTN1,TENM3,ITPKB,FNDC3A,COL14A1,NLGN1,ABL1,JAM2,GRID2,FER,FAT3,NTNG1,CADM1,RC3H2,PCDH9,VCL,ZDHHC21,VCAM1,CDH7,ILDR2,UNC5D,FAT1,ITGA9,NRXN3,PPM1F,ASTN1,KITLG,CTNNA2,ADGRV1,CYFIP2,NDFIP1,TNR,CRB1,PRKCA,CNTN6,PDLIM5*
GO:0051056	regulation of small GTPase mediated signal transduction	9.701466465812935e-8	*TRIO,NRP1,EPS8,ABL2,ARHGEF12,STARD13,FGD4,SIPA1L2,ARHGAP28,RALGPS1,ARHGAP42,SRGAP2,GARNL3,NOTCH2,MADD,ARHGAP26,ARHGAP31,ARHGEF11,ARHGAP32,MYO9A,DGKI,BCR,ARHGEF28,ITPKB,ABL1,MAPRE2,FGF10,SRGAP3,AKAP13,DENND4C,ARFGEF1,KITLG,ARHGAP24,MAP4K4,DOCK3,RELN,AUTS2,VAV3,PSD3*
GO:0048589	developmental growth	9.732208595592196e-8	*ERBB4,MACF1,EVC,NRP1,SLC23A2,AFG3L2,SEMA3A,ALCAM,DCLK1,NIPBL,MBD5,SLC39A12,DISC1,FMN1,APBA2,SLC1A2,SLIT3,NOTCH2*

			,PCDH15,ESR1,RIMS1,GLI3,CPQ,SLC4A10,ITGA4,SYT1,BBS2,ARHGAP32,PRKN,SCAPER,LATS2,PLCB1,PRKG1,DSCAM,DIP2B,MAGI2,AKAP6,LARGE1,MED1,ABL1,JARID2,SEMA6D,PRLR,MTPN,FGF10,RC3H2,EYS,VCL,INSR,ULK2,RIMS2,BMPR1B,SEMA5A,ATP8A2,BNC2,AUTS2,PPP2R3A,CYFIP2,TNR,APP,PDLIM5,SLC6A3,GHRH
GO:0060078	regulation of postsynaptic membrane potential	1.2091612236826347e-7	SLC8A3,GRIK2,IGSF11,GRIK4,GABRG1,GABRB1,GRIN2B,GRIA1,GABRG3,RIMS1,NRXN1,GRID1,GRIK1,DGKI,GRM5,NLGN1,GRID2,GRIK3,MTMR2,GABRG2,RIMS2,KCND2,GRIA4,RELN,CELF4,APP
GO:0022603	regulation of anatomical structure morphogenesis	1.920264706689713e-7	MACF1,PAK1,EPN2,NRP1,RC3H1,EPS8,PRKCB,SLC23A2,ARHGEF7,ANKRD6,STAU2,SEMA3A,LRRC4C,LIMD1,ADAMTS9,CDC42EP3,FGD4,SDC2,MYO10,FAM171A1,ATP10A,SLC39A12,DISC1,HECW2,FYN,SH3KBP1,AGO2,TAFA5,SPRED1,TPM1,ESR1,TJP1,RIMS1,PARVB,JCAD,WASF3,WDPCP,PRICKLE2,HMGA2,ROCK1,COL4A2,DNM3,SYT1,ARHGAP32,PRKN,PTPRD,ADGRB3,MYO9A,NTRK2,DSCAM,DIP2B,PPFIA2,BCR,MAGI2,ZNRF3,SPIRE1,PID1,NLGN1,ABL1,SEMA6D,NTNG1,FGF10,RAPGEF2,ULK2,RIMS2,SEMA5A,GPC6,RELN,ROR1,SNAI2,TNR,AKT3,PRKCA,PDLIM5,ADGRB1
GO:2000145	regulation of cell motility	1.976854853079083e-7	ERBB4,MACF1,PAK1,MITF,SYNE2,NRP1,CDH13,ABL2,ARHGEF7,DACH1,FRMD5,LDLRAD4,SEMA3A,MGAT5,STARD13,ADAMTS9,NIPBL,PHACTR1,AGO2,SRGAP2,ARSB,TAFA5,SPRED1,TPM1,SRGAP2C,TJP1,PTPRT,LAMA1,JCAD,DOCK4,NTRK3,MTUS1,WDPCP,PKN2,ITGA4,ROCK1,DOCK1,BBS2,ARHGAP32,PLCB1,MAPK1,LAMB1,SDCBP,PRKG1,EGFR,BCR,MAGI2,FUT9,DOCK10,LDB2,NRG3,ATP8A1,ABL1,PTPRG,JAM2,SEMA6D,FER,MCC,NTNG1,MAPRE2,FGF10,VCL,RAPGEF2,INSR,SRGAP3,UNC5D,PPM1F,SEMA5A,KITLG,MAP4K4,CTNNA2,APC,RELN,PPP2R3A,SNAI2,TNR,AKT3,IL1R1,BCAS3,PRKCA,APP,ADGRB1
GO:0061564	axon development	2.0578640067015382e-7	MACF1,PAK1,TRIO,NRP1,RPS6KA5,CNTN4,AFG3L2,SEMA3A,LRRC4C,ALCAM,DCLK1,NCAM1,DISC1,FYN,B4GALT6,SLIT3,NOTCH2,GLI3,NRXN1,TSPAN2,ATL1,ZDHHC17,ITGA4,ARHGAP32,PTPRD,NTRK2,PRKG1,DSCAM,NFIB,DIP2B,NCAM2,ARHGEF28,CNTN1,EPHA6,ABL1,SEMA6D,NTNG1,VCL,UNC5D,ULK2,NRXN3,BMPR1B,SEMA5A,ATP8A2,CTNNA2,RELN,AUTS2,CYFIP2,ADAMTSL1,TNR,CNTN6,APP,ADGRB1
GO:0040012	regulation of locomotion	2.6141071228351844e-7	ERBB4,MACF1,PAK1,MITF,SYNE2,NRP1,CDH13,ABL2,ARHGEF7,DACH1,USP14,FRMD5,LDLRAD4,SEMA3A,MGAT5,STARD13,ADAMTS9,NIPBL,PHACTR1,AGO2,SRGAP2,ARSB,TAFA5,SPRED1,TPM1,SRGAP2C,TJP1,PTPRT,LAMA1,JCAD,DOCK4,NTRK3,MTUS1,WDPCP,PKN2,ITGA4,ROCK1,DOCK1,BBS2,ARHGAP32,PLCB1,MAPK1,LAMB1,SDCBP,PRKG1,DSCAM,EGFR,BCR,MAGI2,FUT9,DOCK10,LDB2,NRG3,ATP8A1,ABL1,P

			TPRG,JAM2,SEMA6D,FER,MCC,NTNG1,MAPRE2,FGF10,VCL,RAPGEF2,INSR,SRGAP3,UNC5D,PPM1F,SEMA5A,KITLG,MAP4K4,CTNNA2,APC,RELN,PPP2R3A,SNAI2,TNR,AKT3,IL1R1,BCAS3,PRKCA,APP,ADGRB1
GO:0048583	regulation of response to stimulus	4.070846573113534e-7	*FSTL1,ERBB4,FYB2,MACF1,PAK1,CTNND2,TRIO,SETD2,EPN2,NETO2,EVC,NRP1,CDH13,RC3H1,EPS8,TAOK3,LRFN5,ABL2,PRKCB,GPC5,ARHGEF12,ANKRD6,HTR2C,USP14,CNKSR3,LDLRAD4,SEMA3A,SLC8A3,MGAT5,STARD13,LIMD1,FGD4,GRIK2,SNX25,IGSF11,MBD5,NCAM1,SIPA1L2,CORO2B,ITGA8,PJA2,TAFA4,TTC21B,NEK6,ARHGAP28,DISC1,RALGPS1,ARHGAP42,EYA1,KSR1,EDAR,FRMD6,RGS20,FYN,CREBBP,DLGAP1,SLIT3,RGS7,GRIN2B,SRGAP2,TRABD2B,TAFA5,SPRED1,CACNG2,GARNL3,CRIM1,NOTCH2,MADD,ESR1,NFAT5,TMOD2,PDE4D,ARHGAP26,PTPRT,PRKACB,RIMS1,HIPK3,SGMS1,LAMA1,JCAD,GLI3,NRXN1,NTRK3,FBN1,WWOX,ARID1B,MTUS1,MOSMO,CNKSR2,NEK10,HMGA2,PHLPP1,ZDHHC17,GSG1L,ROCK1,ARHGAP31,UBR1,PRKAA2,ARHGEF11,BBS2,GRB10,ARHGAP32,PRKN,DPF3,SIAH2,LATS2,RORA,PTPRD,HDAC4,DGKG,MYO9A,NTRK2,PLCB1,MAPK1,LAMB1,SDCBP,PRKG1,DSCAM,BIRC6,DGKI,NLK,EGFR,BCR,MAGI2,AKAP6,ARHGEF28,TBC1D1,IQCJ-SCHIP1,RNF152,ZNRF3,GNAQ,ITPKB,GRM5,MED1,SPIRE1,PID1,NLGN1,ABL1,NCOR1,JARID2,GRID2,LRP2,ERBIN,SEMA6D,MBTPS2,FER,PAK5,IL17RA,FBXL17,MCC,SUSD4,PRLR,NFATC2,MTPN,GNG7,MTMR2,MAPRE2,FGF10,NOS1AP,CADM1,RC3H2,RIC8B,ATF6,SEZ6L,RAPGEF2,RGS12,IFT81,SHANK2,ZMYND11,INSR,SRGAP3,AKAP13,WDR41,KLF15,DENND4C,ARFGEF1,ENPP1,RIMS2,PPM1F,DENND2B,DLGAP2,BMPR1B,MAPKBP1,SEMA5A,KITLG,ARHGAP24,MAP4K4,CNIH3,DOCK3,CTNNA2,PRAME,APC,APLF,GPC6,ADGRV1,RELN,VPS13C,AUTS2,ROR1,PPP2R3A,BMP2K,SNAI2,CYFIP2,NDFIP1,TNR,CELF4,AKT3,VAV3,IL1R1,PRKCA,USP8,CNTN6,ENPP3,FMN2,APP,RPS6KA3,SMAD5,SLC6A3,PSD3,ALPK2,EYA2,MAPK8IP1,CCDC88A,GHRH,RAP1GDS1*
GO:0007611	learning or memory	4.945433364538413e-7	*SLC8A3,DNAH11,CLSTN2,TANC1,ITGA8,PJA2,FYN,GRIN2B,GRIA1,BTBD9,TMOD2,BRINP1,NRXN1,PIAS1,PRKN,ADGRB3,NTRK2,PLCB1,MAPK1,DGKI,EGFR,LARGE1,SORCS3,SCN2A,GRM5,ATP8A1,ABL1,PAK5,SHANK2,INSR,SYNJ1,ATXN1,NRXN3,RELN,TNR,APP*
GO:0050807	regulation of synapse organization	9.65163951809437e-7	*RPS6KA5,LRFN5,STAU2,CLSTN2,ADGRL2,TANC1,DISC1,ASAP1,FRMPD4,FYN,GRIN2B,NEGR1,DGKB,PTPRT,NRXN1,CTTNBP2,GRID1,ROCK1,DNM3,PTPRD,ADGRB3,NTRK2,PPFIA2,NLGN1,ABL1,GRID2,SHANK2,CTNNA2,RELN,PTPN13,APP,PDLIM5,ADGRB1*
GO:0007215	glutamate receptor	9.788821148680427e-7	*GRIK2,GRIK4,FYN,GRIN2B,GRIA1,GRID1,PLCB1,GRIK1,GNAQ,GRM5,CPEB4,GRID2,GRIK3,GRIA4,APP*

	signaling pathway		
GO:0035235	ionotropic glutamate receptor signaling pathway	0.000001118016987144 1538	*GRIK2,GRIK4,GRIN2B,GRIA1,GRID1,GRIK1,CPEB4,GRID2,GRIK3,GRIA4,APP*
GO:0016192	vesicle-mediated transport	0.000001172565614597 0602	*CUBN,MACF1,PAK1,ATP9B,EPN2,DOP1B,NRP1,CDH13,ABL2,PRKCB,LYPLA1,DYSF,TBC1D5,EXOC6B,PIK3C3,DCLK1,ADAMTS9,KIF16B,EVI5,VPS41,TAFA4,COG2,CLTCL1,SV2B,LYST,PLEKHA3,FYN,SH3KBP1,CADPS,LRP1B,GRIA1,AP5M1,MYO5A,CACNG2,BTBD9,MEGF11,ERC2,RIMS1,FCHSD2,NRXN1,TRAPPC10,RAPGEF4,EHBP1,SYN2,MYO1E,ITGA4,GSG1L,ROCK1,DOCK1,AMPH,SORCS1,DNM3,SYT1,BBS2,PRKN,EXOC4,SCFD2,MAPK1,SDCBP,ATP9A,EGFR,PPFIA2,STXBP6,CLCN5,BCR,MAGI2,TRAPPC8,STX12,TMPRSS3,ANKFY1,SPIRE1,SNX30,NLGN1,ABL1,MICAL3,ELMO1,LRP2,FER,MON2,SCAMP1,LDLRAD3,MTMR2,LRP12,KIF13A,XKR6,INSR,WDR41,SYNJ1,ARFGEF1,ENPP1,UNC13C,RIMS2,SNX8,NSG1,SV2C,CNIH3,DNER,VPS13C,STON1-GTF2A1L,BMP2K,SNAP91,VAV3,FCHO2,FMN2,ERC1,APP,DNAJC13,VPS13B,ABCA13,MAPK8IP1,ADGRB1*
GO:0060560	developmental growth involved in morphogenesis	0.000001652525614193 0695	*MACF1,NRP1,SLC23A2,SEMA3A,ALCAM,DCLK1,SLC39A12,DISC1,FMN1,SLIT3,ESR1,RIMS1,ITGA4,SYT1,ARHGAP32,PRKN,PRKG1,DSCAM,DIP2B,MAGI2,MED1,ABL1,SEMA6D,FGF10,VCL,ULK2,RIMS2,SEMA5A,AUTS2,PPP2R3A,CYFIP2,TNR,APP*
GO:0048522	positive regulation of cellular process	0.000001853589637765 895	*HPSE2,SAMD4A,ERBB4,KCNMA1,APBB2,MACF1,PAK1,SCAF8,MITF,SETD2,KIRREL1,AMBRA1,SYNE2,EPN2,ZNF208,MYO3B,EVC,NRP1,TEAD1,NFIA,CDH13,RC3H1,RPS6KA5,EPS8,TAOK3,ABL2,PRKCB,SLC23A2,ARHGEF7,UTRN,GPC5,MAML2,PRR16,CDK12,ANKRD6,HTR2C,KCNC1,STAU2,NEK4,FYCO1,LPGAT1,CTIF,TBC1D5,CNKSR3,FRMD5,SEMA3A,SLC8A3,RFX3,MGAT5,SLC40A1,SLCO3A1,ATP11C,ADAMTS9,NIPBL,CEP120,CDC42EP3,CHD6,GRIK2,IGSF11,MBD5,CLSTN2,TCF12,ADGRL2,CORO2B,ITGA8,ZBTB20,MYO10,PJA2,TCF4,IREB2,PBX1,ATP10A,BICRAL,TTC21B,NEK6,ABCD2,MOB1B,DISC1,CLTCL1,RIC3,FMN1,EYA1,KSR1,RORB,SVIL,ASAP1,FRMPD4,EDAR,ST8SIA1,FRMD6,FYN,SH3KBP1,AGO2,NALCN,CREBBP,SLC1A2,ZBTB16,CADPS,RGS7,GRIN2B,ARSB,GRIA1,TRABD2B,NEGR1,SPRED1,SPEN,CACNG2,KANSL1,TPM1,NOTCH2,MADD,TOX,ESR1,NFAT5,SRGAP2C,TMOD2,PDE4D,TJP1,ERC2,RIMS1,FCHSD2,LAMA1,JCAD,GLI3,DOCK4,BRINP1,NRXN1,FRMD4A,SUPT16H,NTRK3,WWOX,WASF3,ARID1B,MAGI1,PRRC1,RAPGEF4,NEK10,ANKRD31,PIAS1,ZNF521,PBX3,HMGA2,ZNF287,CREB5,T*

			NRC6B,RERE,ZDHHC17,PKN2,ITGA4,ROCK1,DOCK1,GLIS1,RXRG,PRKAA2,ARHGEF11,DNM3,SYT1,GRB10,ARHGAP32,ASXL3,PRKN,DPF3,EDIL3,LATS2,RORA,PTPRD,CDC14B,RANBP3L,HDAC4,ADGRB3,ZNF717,MYO3A,NTRK2,PLCB1,MAPK1,LAMB1,SDCBP,BPTF,SOX5,PRKG1,DSCAM,BIRC6,DGKI,NFIB,TUT4,EGFR,MGA,BCR,MAGI2,NELL1,AKAP6,LARGE1,MELTF,FUT9,ANKFY1,IQCJ-SCHIP1,RNF152,CNTN1,TENM3,ITPKB,GRM5,MED1,GLIS3,SPIRE1,AIMP1,LDB2,SNX30,ATP8A1,PID1,NLGN1,ABL1,PRDM10,EBF2,JAM2,GRID2,LRP2,SEMA6D,MBTPS2,FER,SETDB2,RYR2,ELF2,IL17RA,PRLR,NFATC2,CACNB2,STXBP4,MED15,MTPN,VSTM2A,MTMR2,MAPRE2,FGF10,NOS1AP,SOX6,CADM1,RC3H2,PDE4DIP,ATF6,SP3,VCAM1,RAPGEF2,SHANK2,INSR,AKAP13,SYNJ1,KLF15,KDM1B,ULK2,ZNF462,RIMS2,PPM1F,ZFHX3,DENND2B,GFRA1,BMPR1B,NSG1,MAPKBP1,SEMA5A,ATP8A2,SLC24A3,KITLG,PYGO1,MAP4K4,DOCK3,PRAME,APC,APLF,ADGRV1,RELN,AUTS2,ROR1,PPP2R3A,BMP2K,SNAI2,CYFIP2,SLC4A4,NDFIP1,TNR,CELF4,AKT3,VAV3,IL1R1,BCAS3,NPAS3,PRKCA,KMT2C,USP8,MRTFB,CNTN6,FMN2,APP,RPS6KA3,SMAD5,ABCA13,EYA2,MAPK8IP1,ADGRB1,CCDC88A,GHRH,ARNT2
GO:0055085	transmembrane transport	0.000001889852922793917	*SLMAP,KCNMA1,SLC17A1,SLC6A11,SLC15A5,PRKCB,SLC23A2,UTRN,SLC8A1,SLC2A13,HTR2C,KCNC1,CACNA1E,TMEM63C,AFG3L2,CNKSR3,SLC8A3,SLC40A1,SLCO3A1,ATP11C,SLC9C1,GRIK2,SLC25A21,GRIK4,GABRG1,ATP10A,ABCD2,SLC39A12,CLTCL1,SV2B,HECW2,PIEZO2,SLC35F1,GABRB1,TMEM241,SLC16A9,ATP2B2,FYN,NALCN,SLC1A2,RGS7,GRIN2B,GRIA1,FGF12,CACNG2,GABRG3,ABCB5,SLC44A5,PDE4D,ITPR2,NRXN1,LONP2,DPP6,GRID1,CACNA2D3,SLC4A10,SLC7A2,ABCC9,GSG1L,CACNA1C,GRB10,TRPM7,PLCB1,GRIK1,CUL5,PRKG1,CLCN5,BCR,AKAP6,LARGE1,MELTF,SLC37A2,SLC12A1,SCN2A,GRM5,ATP8A1,PID1,NLGN1,ABL1,GRID2,LRP2,RYR2,GRIK3,CACNB2,STXBP4,KCNH5,LRRC8B,NOS1AP,TRPM3,INSR,UNC80,KLF15,GABRG2,ENPP1,KCNQ3,SCN8A,KCND2,SLC24A3,SV2C,GRIA4,CNIH3,ABCA5,RELN,KCNJ15,CYBRD1,SLC4A4,OCA2,KCNS3,APP,SLC6A3,ABCA13*
GO:0050890	cognition	0.0000020398307031739632	*DOP1B,SLC8A3,DNAH11,NIPBL,CLSTN2,TANC1,ITGA8,PJA2,FYN,GRIN2B,GRIA1,BTBD9,TMOD2,BRINP1,NRXN1,PIAS1,PRKN,ADGRB3,NTRK2,PLCB1,MAPK1,DGKI,EGFR,LARGE1,SORCS3,SCN2A,GRM5,ATP8A1,ABL1,PAK5,SHANK2,INSR,SYNJ1,ATXN1,NRXN3,RELN,TNR,APP*
GO:0050803	regulation of synapse structure or activity	0.0000020401472969832026	*RPS6KA5,LRFN5,STAU2,CLSTN2,ADGRL2,TANC1,DISC1,ASAP1,FRMPD4,FYN,GRIN2B,NEGR1,DGKB,PTPRT,NRXN1,CTTNBP2,GRID1,ROCK1,DNM3,PTPRD,ADGRB3,NTRK2,PPFIA2,NLGN1,ABL1,GRID2,SHANK2,CTNNA2,RELN,PTPN13,APP,PDLIM5,ADGRB1*

GO:0040007	growth	0.000002062416960742616	*ERBB4,MACF1,EVC,NRP1,TEAD1,PRKCB,SLC23A2,RERG,AFG3L2,SEMA3A,ALCAM,DCLK1,NIPBL,MBD5,SLC39A12,DISC1,FMN1,APBA2,SLC1A2,SLIT3,NOTCH2,PCDH15,ESR1,RIMS1,GLI3,CPQ,SLC4A10,ITGA4,ARHGEF11,SYT1,BBS2,ARHGAP32,PRKN,SCAPER,LATS2,PLCB1,SDCBP,PRKG1,DSCAM,DIP2B,EGFR,MAGI2,AKAP6,LARGE1,MED1,NRG3,ABL1,JARID2,SEMA6D,PAK5,PRLR,MTPN,LRP12,FGF10,RC3H2,EYS,VCL,INSR,ENPP1,ULK2,RIMS2,PPM1F,BMPR1B,SEMA5A,ATP8A2,BNC2,AUTS2,PPP2R3A,CYFIP2,TNR,APP,PDLIM5,RPS6KA3,SLC6A3,GHRH,SPOCK1*
GO:0051641	cellular localization	0.0000024139181687035393	*SLMAP,ERBB4,FYB2,CUBN,MACF1,PAK1,SLC6A11,SETD2,SLC15A5,SYNE2,NETO2,DOP1B,NRP1,CDH13,TANGO2,HERC2,PRKCB,GPC5,SLC8A1,LYPLA1,HTR2C,DYSF,STAU2,SIAH3,FYCO1,TBC1D5,EXOC6B,RFX3,SLC40A1,PIK3C3,DCLK1,DNAH11,ADAMTS9,NIPBL,CEP120,GRIK2,SNX25,IGSF11,CCDC186,MYO5C,KIF16B,ODR4,CORO2B,EVI5,ITGA8,SYCP1,VPS41,MYO10,ASB3,TOM1L2,IREB2,TTC21B,ABCD2,COG2,DISC1,CLTCL1,SV2B,RIC3,GPR158,APBA2,LYST,PLEKHA3,FRMD6,FYN,CREBBP,SLC1A2,ZBTB16,CADPS,LRBA,SGTB,LRP1B,ARSB,AP5M1,MYO5A,CACNG2,TSPAN33,BTBD9,NPIPA1,ESR1,PDE4D,TJP1,ERC2,RIMS1,FCHSD2,GLI3,ITPR2,NRXN1,FRMD4A,FBN1,LONP2,TRAPPC10,WDPCP,RAPGEF4,EHBP1,DPP6,SEL1L,SYN2,MYO1E,ZDHHC17,ITGA4,GSG1L,ROCK1,CACNA1C,PRKAA2,AMPH,RGPD4,DNM3,PACRG,SYT1,BBS2,PRKN,LATS2,EXOC4,DLG2,SCFD2,BBS9,PTPRN2,RANBP3L,PLCB1,MAPK1,SDCBP,ATP9A,DGKI,NBEA,EGFR,PPFIA2,BCR,MAGI2,AKAP6,LARGE1,TRAPPC8,STX12,ANKFY1,TBC1D1,MED1,GNPTAB,SPIRE1,WDR72,SNX30,PID1,NLGN1,ABL1,JARID2,GRID2,LRP2,RANBP17,ERBIN,FER,MON2,RYR2,SCAMP1,MCC,DST,CACNB2,STXBP4,MTMR2,MAPRE2,FGF10,KIF13A,IPO11,VCL,ZDHHC21,RAPGEF2,IFT81,ILDR2,IMMP2L,SYNJ1,DENND4C,ATXN1,ARFGEF1,UNC13C,TANGO6,NRXN3,RIMS2,PPM1F,KCNQ3,SNX8,NSG1,CPE,PYGO1,SV2C,MAP4K4,EFR3A,APC,APLF,GPC6,PARD3B,ADGRV1,RELN,AKAP11,VPS13C,CYBRD1,NDFIP1,SNAP91,CRB1,FCHO2,HEPACAM,USP8,USH1C,DNAH5,FMN2,ERC1,APP,SLC6A3,DNAJC13,VPS13B,ABCA13,EYA2,CCDC88A,RAP1GDS1*
GO:0098657	import into cell	0.0000031738533199455505	*CUBN,PAK1,SLC6A11,ATP9B,EPN2,CDH13,ABL2,SLC8A1,DYSF,CACNA1E,TBC1D5,SLC8A3,PIK3C3,SLC9C1,TAFA4,SLC39A12,CLTCL1,LYST,FYN,SH3KBP1,SLC1A2,LRP1B,GRIA1,CACNG2,BTBD9,MEGF11,FCHSD2,EHBP1,MYO1E,SLC7A2,ITGA4,ABCC9,GSG1L,ROCK1,DOCK1,CACNA1C,AMPH,DNM3,SYT1,PRKN,MAPK1,SDCBP,ATP9A,EGFR,CLCN5,BCR,MAGI2,TMPRSS3,ANKFY1,SLC12A1,SCN2A,NLGN1,ABL1,ELMO1,LRP2,SCAMP1,LDLRAD3,MTMR2,LRP12,XKR6,INSR,SYNJ1,ENPP1,SCN8A,DNER,KCNJ15,*

			STON1-GTF2A1L,BMP2K,SNAP91,VAV3,FCHO2,APP,SLC6A3,DNAJC13,ABCA13,ADGRB1
GO:0048518	positive regulation of biological process	0.000004537059043646632	*HPSE2,SAMD4A,ERBB4,KCNMA1,APBB2,FYB2,MACF1,PAK1,SCAF8,MITF,SETD2,KIRREL1,AMBRA1,SYNE2,EPN2,ZNF208,MYO3B,EVC,NRP1,TEAD1,NFIA,CDH13,RC3H1,RPS6KA5,LHFPL2,EPS8,TAOK3,ABL2,PRKCB,SLC23A2,ARHGEF7,UTRN,GPC5,MAML2,SLC8A1,PRR16,CDK12,SLC2A13,ANKRD6,HTR2C,KCNC1,STAU2,NEK4,FYCO1,LPGAT1,CTIF,TBC1D5,CNKSR3,FRMD5,SEMA3A,PLIN2,SLC8A3,RFX3,MGAT5,SLC40A1,SLCO3A1,ATP11C,LIMD1,ADAMTS9,NIPBL,CEP120,CDC42EP3,CHD6,GRIK2,IGSF11,GSAP,MBD5,CLSTN2,TCF12,ADGRL2,CORO2B,ITGA8,ZBTB20,MYO10,PJA2,TCF4,IREB2,PBX1,ATP10A,BICRAL,TTC21B,NEK6,ABCD2,SLC39A12,MOB1B,DISC1,CLTCL1,RIC3,FMN1,EYA1,KSR1,RORB,SVIL,ASAP1,FRMPD4,EDAR,ST8SIA1,FRMD6,FYN,SH3KBP1,AGO2,NALCN,CREBBP,SLC1A2,ZBTB16,CADPS,RGS7,GRIN2B,ANTXR1,ARSB,GRIA1,TRABD2B,NEGR1,SPRED1,SPEN,FGF12,CACNG2,KANSL1,TPM1,NOTCH2,MADD,TOX,ESR1,NFAT5,SRGAP2C,TMOD2,PDE4D,TJP1,ERC2,RIMS1,FCHSD2,LAMA1,JCAD,GLI3,DOCK4,BRINP1,NRXN1,FRMD4A,SUPT16H,NTRK3,BCL2L13,WWOX,WASF3,ARID1B,MAGI1,PRRC1,RAPGEF4,NEK10,ANKRD31,PIAS1,ZNF521,PBX3,HMGA2,ZNF287,CREB5,TNRC6B,RERE,ZDHHC17,PKN2,ITGA4,ROCK1,DOCK1,GLIS1,RXRG,PRKAA2,ARHGEF11,DNM3,SYT1,BBS2,GRB10,ARHGAP32,ASXL3,PRKN,DPF3,EDIL3,LATS2,RORA,PTPRD,CDC14B,RANBP3L,HDAC4,ADGRB3,ZNF717,MYO3A,NTRK2,PLCB1,MAPK1,LAMB1,SDCBP,BPTF,SOX5,PRKG1,DSCAM,BIRC6,DGKI,NFIB,DIP2B,TUT4,EGFR,MGA,BCR,MAGI2,NELL1,AKAP6,LARGE1,FANCM,MELTF,FUT9,ANKFY1,IQCJ-SCHIP1,RNF152,CNTN1,TENM3,ITPKB,GRM5,MED1,GLIS3,SPIRE1,AIMP1,LDB2,SNX30,ATP8A1,PID1,NLGN1,ABL1,PRDM10,EBF2,JAM2,GRID2,LRP2,ERBIN,SEMA6D,MBTPS2,FER,SETDB2,RYR2,ELF2,IL17RA,SUSD4,PRLR,NFATC2,CACNB2,STXBP4,MED15,MTPN,VSTM2A,MTMR2,MAPRE2,FGF10,NOS1AP,SOX6,CADM1,RC3H2,PDE4DIP,ATF6,SP3,VCAM1,RAPGEF2,SHANK2,INSR,AKAP13,SYNJ1,KLF15,KDM1B,ARFGEF1,ULK2,ZNF462,RIMS2,PPM1F,ZFHX3,DENND2B,EGLN3,GFRA1,BMPR1B,NSG1,MAPKBP1,SEMA5A,ATP8A2,SLC24A3,KITLG,PYGO1,MAP4K4,DOCK3,PRAME,APC,APLF,ABCA5,ADGRV1,RELN,AUTS2,ROR1,PPP2R3A,BMP2K,SNAI2,CYFIP2,SLC4A4,NDFIP1,TNR,CELF4,AKT3,VAV3,IL1R1,BCAS3,NPAS3,PRKCA,KMT2C,USP8,MRTFB,CNTN6,ENPP3,FMN2,APP,RPS6KA3,SMAD5,SLC6A3,ABCA13,EYA2,MAPK8IP1,ADGRB1,CCDC88A,GHRH,ARNT2*
GO:1990806	ligand-gated ion channel	0.000004575341013824	*GRIK2,GRIK4,GRIN2B,GRIA1,GRID1,PLCB1,GRIK1,CPEB4,GRID2,GRIK3,GRIA4,APP*

	signaling pathway	816	
GO:0040011	locomotion	0.000005356189474489572	*ERBB4,MACF1,PAK1,MITF,SYNE2,NRP1,CDH13,ABL2,ARHGEF7,DACH1,USP14,FRMD5,LDLRAD4,SEMA3A,MGAT5,STARD13,ADAMTS9,NIPBL,TAFA4,PHACTR1,LYST,AGO2,SLIT3,SRGAP2,ARSB,TAFA5,SPRED1,TPM1,SRGAP2C,TJP1,PTPRT,LAMA1,JCAD,DOCK4,NTRK3,MTUS1,WDPCP,PKN2,ITGA4,ROCK1,DOCK1,BBS2,ARHGAP32,PLCB1,MAPK1,LAMB1,SDCBP,PRKG1,DSCAM,EGFR,BCR,MAGI2,FUT9,DOCK10,LDB2,NRG3,ATP8A1,ABL1,PTPRG,JAM2,SEMA6D,FER,IL17RA,MCC,NTNG1,MAPRE2,FGF10,VCL,VCAM1,RAPGEF2,INSR,SRGAP3,UNC5D,ITGA9,PPM1F,SEMA5A,KITLG,MAP4K4,CTNNA2,APC,RELN,PPP2R3A,SNAI2,TNR,AKT3,VAV3,IL1R1,BCAS3,PRKCA,APP,ADGRB1*
GO:0016358	dendrite development	0.00000822317311599826	*CTNND2,NRP1,STAU2,SEMA3A,DCLK1,SDC2,PHACTR1,DISC1,HECW2,ASAP1,FYN,CSMD3,SRGAP2,SRGAP2C,RERE,DNM3,PTPRD,ADGRB3,DGKG,PRKG1,DSCAM,PPFIA2,DOCK10,NLGN1,ABL1,FAT3,RAPGEF2,CTNNA2,RELN,APP,PDLIM5*
GO:0048167	regulation of synaptic plasticity	0.000011512783591507674	*STAU2,CNTN4,SLC8A3,GRIK2,IGSF11,GRIN2B,GRIA1,ERC2,RIMS1,SLC4A10,NTRK2,MAPK1,DGKI,LARGE1,SORCS3,GRM5,ABL1,GRID2,RAPGEF2,SHANK2,UNC13C,RIMS2,KCNQ3,NSG1,RELN,TNR,ERC1,APP,ADGRB1*
GO:0007416	synapse assembly	0.000012641586574001866	*ERBB4,LRFN5,STAU2,CLSTN2,ADGRL2,SRGAP2,GRIA1,NEGR1,SRGAP2C,NRXN1,DNM3,PTPRD,ADGRB3,NTRK2,SDCBP,DSCAM,LARGE1,NRG3,NLGN1,GRID2,SHANK2,GABRG2,NRXN3,DNER,PTPN13,APP,PDLIM5,ADGRB1*
GO:0007612	learning	0.000014662482629540292	*SLC8A3,CLSTN2,TANC1,FYN,NRXN1,PIAS1,PRKN,ADGRB3,NTRK2,PLCB1,DGKI,SORCS3,GRM5,ATP8A1,ABL1,PAK5,SHANK2,INSR,SYNJ1,ATXN1,NRXN3,RELN,TNR,APP*
GO:0030900	forebrain development	0.000014792979918592 2	*ERBB4,SETD2,SYNE2,NRP1,KCNC1,SEMA3A,DCLK1,CEP120,PHACTR1,TTC21B,DISC1,FYN,SLC1A2,SRGAP2,GRIA1,TOX,SRGAP2C,ZSWIM6,GLI3,SLC4A10,HERC1,BBS2,FOXP2,NTRK2,PLCB1,LAMB1,PRKG1,NFIB,EGFR,LARGE1,SCN2A,NRG3,LRP2,FGF10,PCDH9,RAPGEF2,SEMA5A,RELN,TNR,DNAH5,APP,SLC6A3,GHRH*
GO:0051239	regulation of multicellular organismal process	0.000016317643939451274	*ERBB4,MACF1,MITF,SETD2,AMBRA1,EPN2,NETO2,NRP1,RC3H1,RPS6KA5,LRFN5,ABL2,PRKCB,SLC8A1,LNPEP,HTR2C,STAU2,AFG3L2,LDLRAD4,SEMA3A,SLC8A3,RFX3,STARD13,ATP11C,ADAMTS9,NIPBL,GRIK2,IGSF11,MBD5,CLSTN2,ADGRL2,CORO2B,ZBTB20,PJA2,ASB3,TAFA4,PBX1,BICRAL,TTC21B,ABCD2,SLC39A12,DISC1,ARHGAP42,ATP2B2,FYN,SH3KBP1,AGO2,ZBTB16,DLGAP1,GRIN2B,ARSB,TAFA5,SPRED1,SPEN,FGF12,TPM1,NOTCH2,TOX,ESR1,PDE4D,TJP1,RIMS1,LAMA1,JCAD,GLI3,DOCK4,BRINP1,NRXN1,FBN1,WASF3,ARID1B,WDPCP,PBX3,HMGA2,ZNF287,ABCC9,ROCK1,DOCK1,CACNA1C,COL4A2,BBS2,GRB10,ARHGAP32,*

			LATS2,PPP1R12B,RORA,PTPRD,TG,HDAC4,ADGRB3,NTRK2,PLCB1,MAPK1,SDCBP,SOX5,PRKG1,DSCAM,NFIB,DIP2B,EGFR,BCR,NELL1,AKAP6,PLCL1,GNAQ,ITPKB,GRM5,MED1,NLGN1,ABL1,PTPRG,JARID2,EBF2,JAM2,GRID2,LRP2,ERBIN,SEMA6D,FER,RYR2,IL17RA,MCC,PRLR,NFATC2,CACNB2,MTPN,MTMR2,MAPRE2,FGF10,NOS1AP,SOX6,CADM1,RC3H2,VCL,ZDHHC21,VCAM1,RAPGEF2,INSR,ILDR2,ENPP1,ULK2,RIMS2,PPM1F,ZFHX3,DLGAP2,BMPR1B,MAPKBP1,SEMA5A,ATP8A2,KITLG,MAP4K4,APC,APLF,ADGRV1,RELN,ANXA4,BMP2K,SNAI2,NDFIP1,TNR,PTPN13,CELF4,AKT3,VAV3,IL1R1,BCAS3,PRKCA,MRTFB,ENPP3,APP,SLC6A3,ALPK2,MAPK8IP1,ADGRB1,GHRH
GO:0009888	tissue development	0.000017199445503005738	FSTL1,ERBB4,PAK1,SETD2,AMBRA1,ALPK3,EVC,NRP1,NFIA,SLC8A1,ANKRD6,LDLRAD4,SEMA3A,RFX3,RBFOX1,STARD13,SLC40A1,ADAMTS9,KIF16B,NEBL,ITGA8,PBX1,SLC39A12,FMN1,EYA1,SVIL,EDAR,FRMD6,ZBTB16,SPRED1,TPM1,COL19A1,NOTCH2,FLNB,TOX,PCDH15,ESR1,PDE4D,TJP1,PRKACB,LAMA1,GLI3,SGCG,HYDIN,WDPCP,PRICKLE2,HMGA2,FHOD3,GREB1L,MYO1E,ITGA4,ROCK1,COL4A2,BBS2,LATS2,EXOC4,RANBP3L,HDAC4,MYO9A,PLCB1,MAPK1,LAMB1,SDCBP,BPTF,SOX5,PRKG1,BIRC6,NFIB,EGFR,PTPRQ,BCR,MAGI2,NELL1,AKAP6,LARGE1,ZNRF3,MED1,FNDC3A,LDB2,WDR72,ABL1,JARID2,EBF2,LRP2,SEMA6D,FER,SETDB2,RYR2,FBXL17,PRLR,NFATC2,MTPN,FGF10,SOX6,SGCD,VCL,ZDHHC21,RAPGEF2,INSR,AKAP13,KLF15,FAT1,ENPP1,PGM5,BMPR1B,SEMA5A,SLC24A3,KITLG,ARHGAP24,APC,APLF,GPC6,DNER,ADGRV1,BNC2,ANXA4,ROR1,PPP2R3A,BMP2K,SNAI2,KAZN,USH1C,MRTFB,PDLIM5,SMAD5,VPS13B,ALPK2,ASB2,EYA2
GO:0035249	synaptic transmission, glutamatergic	0.000017248535780097515	GRIK2,GRIK4,DISC1,GRIN2B,GRIA1,CACNG2,NRXN1,GRID1,SYT1,PRKN,GRIK1,DGKI,GRM5,NLGN1,GRID2,GRIK3,UNC13C,GRIA4,RELN,TNR
GO:1901888	regulation of cell junction assembly	0.00003373823174834973	MACF1,NRP1,LRFN5,STAU2,CLSTN2,ADGRL2,FMN1,PEAK1,NEGR1,TJP1,NRXN1,WDPCP,ROCK1,PTPRD,ADGRB3,NTRK2,NLGN1,ABL1,GRID2,VCL,RAPGEF2,PPM1F,MAP4K4,SNAI2,PTPN13,APP,PDLIM5,ADGRB1
GO:0006897	endocytosis	0.000035896040457260226	CUBN,PAK1,ATP9B,EPN2,CDH13,ABL2,DYSF,TBC1D5,PIK3C3,TAFA4,CLTCL1,LYST,FYN,SH3KBP1,LRP1B,GRIA1,CACNG2,BTBD9,MEGF11,FCHSD2,EHBP1,MYO1E,ITGA4,GSG1L,ROCK1,DOCK1,AMPH,DNM3,SYT1,PRKN,MAPK1,SDCBP,ATP9A,EGFR,CLCN5,BCR,MAGI2,TMPRSS3,ANKFY1,NLGN1,ABL1,ELMO1,LRP2,SCAMP1,LDLRAD3,MTMR2,LRP12,XKR6,INSR,SYNJ1,ENPP1,DNER,STON1-GTF2A1L,BMP2K,SNAP91,VAV3,FCHO2,APP,DNAJC13,ABCA13,ADGRB1
GO:009917	postsynapse	0.00003761	CTNND2,NRP1,RPS6KA5,STAU2,TANC1,DISC1

3	organization	878764119946	,*ASAP1,FRMPD4,FYN,GRIN2B,DGKB,NRXN1,CNKSR2,GRID1,DNM3,PTPRD,PPFIA2,DOCK10,NLGN1,GRID2,MTMR2,NOS1AP,SHANK2,INSR,NRXN3,RELN,PDLIM5*
GO:0048880	sensory system development	0.00003828559147451798	*PDE6A,MITF,NRP1,NFIA,STAU2,SEMA3A,DCLK1,NIPBL,PBX1,RORB,ATP2B2,SPRED1,NOTCH2,ABCB5,MEGF11,LAMA1,GLI3,FBN1,WDPCP,PBX3,CACNA1C,SCAPER,NTRK2,DSCAM,NFIB,EGFR,LARGE1,TENM3,MED1,SMOC1,FAT3,FGF10,ATF6,SP3,FAT1,BMPR1B,ATP8A2,PPP2R3A,CELF4,CRB1,USH1C,SLC6A3*
GO:0009887	animal organ morphogenesis	0.00003872739742831593	*ERBB4,SETD2,MYO3B,NRP1,PRKCB,ANKRD6,STAU2,MMP16,SLC40A1,DNAH11,NIPBL,ITGA8,PBX1,FMN1,EYA1,RORB,EDAR,SLIT3,TPM1,NOTCH2,PCDH15,ESR1,MEGF11,LRIG1,LAMA1,GLI3,FBN1,WWOX,WDPCP,PBX3,PRICKLE2,GREB1L,SLC4A10,BBS2,ASXL3,EXOC4,MYO3A,FOXN3,NTRK2,MAPK1,LAMB1,SOX5,DSCAM,NFIB,EGFR,PTPRQ,BCR,MAGI2,LARGE1,ZNRF3,TENM3,MED1,WDR72,NRG3,ABL1,LRP2,SETDB2,RYR2,FAT3,FGF10,SOX6,SP3,INSR,FAT1,BMPR1B,CPE,ATP8A2,FREM1,CTNNA2,APC,GPC6,ROR1,PPP2R3A,SNAI2,AKT3,CRB1,USH1C,ALPK2,ASB2*
GO:0001654	eye development	0.000056131651542705	*PDE6A,MITF,NRP1,NFIA,STAU2,DCLK1,NIPBL,PBX1,RORB,ATP2B2,SPRED1,NOTCH2,ABCB5,MEGF11,LAMA1,GLI3,FBN1,WDPCP,PBX3,CACNA1C,SCAPER,NTRK2,DSCAM,NFIB,EGFR,LARGE1,TENM3,MED1,SMOC1,FAT3,FGF10,ATF6,SP3,FAT1,BMPR1B,ATP8A2,PPP2R3A,CELF4,CRB1,USH1C,SLC6A3*
GO:0001764	neuron migration	0.0000703040505781477	*NRP1,SEMA3A,DCLK1,NIPBL,PHACTR1,DISC1,FYN,SRGAP2,SRGAP2C,ARHGAP32,NTRK2,PRKG1,LARGE1,NRG3,FAT3,NTNG1,LRP12,RAPGEF2,UNC5D,ASTN1,CTNNA2,DNER,RELN,AUTS2,SPOCK1*
GO:0150063	visual system development	0.00007410543723620689	*PDE6A,MITF,NRP1,NFIA,STAU2,DCLK1,NIPBL,PBX1,RORB,ATP2B2,SPRED1,NOTCH2,ABCB5,MEGF11,LAMA1,GLI3,FBN1,WDPCP,PBX3,CACNA1C,SCAPER,NTRK2,DSCAM,NFIB,EGFR,LARGE1,TENM3,MED1,SMOC1,FAT3,FGF10,ATF6,SP3,FAT1,BMPR1B,ATP8A2,PPP2R3A,CELF4,CRB1,USH1C,SLC6A3*
GO:0048588	developmental cell growth	0.00008048646331385824	*MACF1,NRP1,SLC23A2,SEMA3A,ALCAM,DCLK1,SLC39A12,DISC1,SLIT3,RIMS1,ITGA4,SYT1,ARHGAP32,PRKN,PRKG1,DSCAM,DIP2B,AKAP6,ABL1,SEMA6D,VCL,ULK2,RIMS2,SEMA5A,AUTS2,CYFIP2,TNR,APP,PDLIM5*
GO:0050877	nervous system process	0.00009169618096081745	*PDE6A,NETO2,DOP1B,MYO3B,OR4C46,HTR2C,SLC8A3,RBFOX1,DNAH11,NIPBL,HMCN1,GRIK2,IGSF11,CLSTN2,TANC1,ITGA8,PJA2,TAFA4,GABRG1,EYA1,RORB,PIEZO2,GABRB1,ATP2B2,FYN,DLGAP1,GRIN2B,GRIA1,NAV2,FGF12,CACNG2,BTBD9,PCDH15,GABRG3,TMOD2,TJP1,LRIG1,RIMS1,BRINP1,NRXN1,WASF3,PIAS1,PBX3,SLC4A10,HERC1,BBS2,PRKN,BBS9,ADGRB3,MYO9A,MYO3A,NTRK2,PLCB1,MAPK1,DGKI,EGFR,PTPRQ,BCR,LARGE1,TMPRSS3,SORCS3,SCN2A,POU6F2,GRM5,ATP8A1,NLGN1,AB*

			L1,OR9Q1,JAM2,GRID2,LRP2,PAK5,CACNB2,MTMR2,ATF6,EYS,SHANK2,INSR,PPIP5K2,SYNJ1,ATXN1,GABRG2,NRXN3,RIMS2,KCNQ3,SCN8A,DLGAP2,KCND2,ATP8A2,LHFPL3,CTNNA2,ADGRV1,RELN,ROR1,SNAI2,TNR,CELF4,CRB1,USH1C,APP,SLC6A3
GO:0007423	sensory organ development	0.0000926119156662275 3	*PDE6A,MITF,MYO3B,NRP1,NFIA,STAU2,DCLK1,NIPBL,ITGA8,PBX1,EYA1,RORB,ATP2B2,SPRED1,NOTCH2,PCDH15,ABCB5,MEGF11,LRIG1,LAMA1,GLI3,FBN1,WDPCP,PBX3,CACNA1C,SCAPER,MYO3A,NTRK2,MAPK1,DSCAM,NFIB,EGFR,PTPRQ,BCR,LARGE1,TENM3,MED1,SMOC1,FAT3,FGF10,ATF6,SP3,FAT1,BMPR1B,ATP8A2,ADGRV1,BNC2,ROR1,PPP2R3A,CELF4,CRB1,USH1C,SLC6A3*
GO:1990138	neuron projection extension	0.0000985485003272216 6	*MACF1,NRP1,SLC23A2,SEMA3A,ALCAM,DCLK1,SLC39A12,DISC1,SLIT3,RIMS1,ITGA4,SYT1,ARHGAP32,PRKN,DSCAM,DIP2B,ABL1,SEMA6D,VCL,ULK2,RIMS2,SEMA5A,AUTS2,CYFIP2,TNR*
GO:0072359	circulatory system development	0.0001354277493306567 4	*ERBB4,SETD2,EPN2,ALPK3,NRP1,CDH13,PRKCB,SLC8A1,STARD13,DNAH11,ADAMTS9,NIPBL,NEBL,SLC39A12,EYA1,AGO2,SLIT3,ANTXR1,TAFA5,SPRED1,FGF12,TPM1,NOTCH2,TJP1,LAMA1,JCAD,GLI3,NRXN1,NTRK3,SGCG,FBN1,WDPCP,HMGA2,FHOD3,GREB1L,MYO1E,ROCK1,CACNA1C,COL4A2,RORA,ADGRB3,NTRK2,MAPK1,PRKG1,THSD7A,EGFR,AKAP6,LARGE1,MED1,ENPEP,AIMP1,ABL1,JARID2,LRP2,SETDB2,RYR2,NFATC2,FGF10,SOX6,SGCD,VCAM1,RAPGEF2,INSR,AKAP13,IMMP2L,NRXN3,CPE,SEMA5A,ARHGAP24,APC,SNAI2,AKT3,VAV3,BCAS3,PRKCA,DNAH5,PDLIM5,SMAD5,ALPK2,ASB2,ADGRB1*
GO:0031644	regulation of nervous system process	0.0001709971627474019 7	*NETO2,HTR2C,SLC8A3,GRIK2,IGSF11,TAFA4,DLGAP1,GRIN2B,FGF12,RIMS1,NRXN1,WASF3,NLGN1,JAM2,MTMR2,RIMS2,DLGAP2,RELN,TNR,CELF4,APP*
GO:0044087	regulation of cellular component biogenesis	0.0001975937469225298 8	*MACF1,PAK1,KIRREL1,AMBRA1,SYNE2,MYO3B,NRP1,EPS8,LRFN5,ARHGEF7,STAU2,CEP120,CDC42EP3,CLSTN2,ADGRL2,CORO2B,VPS41,MYO10,SLC39A12,ARHGAP28,FMN1,PEAK1,SVIL,ANTXR1,TRABD2B,NEGR1,TPM1,SRGAP2C,TMOD2,TJP1,FCHSD2,NRXN1,WDPCP,FHOD3,ROCK1,PRKAA2,DNM3,PRKN,SACS,PTPRD,ADGRB3,MYO3A,NTRK2,SDCBP,STXBP6,LDB2,SNX30,NLGN1,ABL1,GRID2,FER,SETDB2,MTPN,PDE4DIP,VCL,RAPGEF2,ARFGEF1,RHPN2,PPM1F,ARHGAP24,MAP4K4,APC,AUTS2,MTMR3,SNAI2,CYFIP2,PTPN13,PRKCA,APP,PDLIM5,ADGRB1,CCDC88A*
GO:0034220	monoatomic ion transmembrane transport	0.000202757390508728	*SLMAP,KCNMA1,SLC17A1,SLC6A11,UTRN,SLC8A1,HTR2C,KCNC1,CACNA1E,TMEM63C,AFG3L2,CNKSR3,SLC8A3,SLC40A1,ATP11C,SLC9C1,GRIK2,GRIK4,GABRG1,ATP10A,SLC39A12,HECW2,PIEZO2,GABRB1,ATP2B2,FYN,NALCN,SLC1A2,RGS7,GRIN2B,GRIA1,FGF12,CACNG2,GABRG3,PDE4D,ITPR2,DPP6,GRID1,CACNA2D*

			3,SLC4A10,ABCC9,CACNA1C,TRPM7,PLCB1,GRIK1,CUL5,PRKG1,CLCN5,AKAP6,LARGE1,MELTF,SLC12A1,SCN2A,GRM5,ATP8A1,ABL1,GRID2,RYR2,GRIK3,CACNB2,KCNH5,LRRC8B,NOS1AP,TRPM3,UNC80,GABRG2,KCNQ3,SCN8A,KCND2,SLC24A3,GRIA4,RELN,KCNJ15,SLC4A4,OCA2,KCNS3,SLC6A3
GO:0008104	protein localization	0.00021100026993359615	*SLMAP,ERBB4,FYB2,CUBN,MACF1,PAK1,SETD2,SLC15A5,NETO2,DOP1B,NRP1,TANGO2,HERC2,PRKCB,GPC5,LYPLA1,STAU2,SIAH3,TBC1D5,EXOC6B,RFX3,PIK3C3,DCLK1,DNAH11,ADAMTS9,NIPBL,CEP120,GRIK2,SNX25,IGSF11,CCDC186,ODR4,CORO2B,ITGA8,SYCP1,VPS41,ASB3,TOM1L2,TTC21B,COG2,DISC1,CLTCL1,RIC3,GPR158,APBA2,LYST,FRMD6,FYN,CREBBP,ZBTB16,CADPS,LRBA,SGTB,LRP1B,AP5M1,MYO5A,CACNG2,TSPAN33,NPIPA1,ESR1,TJP1,RIMS1,FCHSD2,GLI3,NRXN1,FRMD4A,FBN1,LONP2,WDPCP,RAPGEF4,EHBP1,DPP6,SEL1L,ZDHHC17,ITGA4,ROCK1,PRKAA2,RGPD4,PACRG,BBS2,PRKN,LATS2,EXOC4,DLG2,SCFD2,BBS9,PTPRN2,RANBP3L,PLCB1,SDCBP,NBEA,EGFR,BCR,MAGI2,AKAP6,LARGE1,STX12,TBC1D1,MED1,GNPTAB,SPIRE1,WDR72,SNX30,PID1,NLGN1,ABL1,JARID2,GRID2,LRP2,RANBP17,ERBIN,MON2,RYR2,SCAMP1,MCC,CACNB2,STXBP4,MAPRE2,FGF10,KIF13A,IPO11,VCL,ZDHHC21,RAPGEF2,ILDR2,IMMP2L,DENND4C,ARFGEF1,TANGO6,NRXN3,RIMS2,PPM1F,KCNQ3,SNX8,NSG1,CPE,PYGO1,MAP4K4,EFR3A,APC,APLF,GPC6,PARD3B,ADGRV1,RELN,AKAP11,VPS13C,NDFIP1,SNAP91,CRB1,FCHO2,HEPACAM,USP8,USH1C,FMN2,ERC1,DNAJC13,CCDC88A,RAP1GDS1*
GO:0036211	protein modification process	0.00022171680000658342	*ERBB4,KLHL13,RNF38,PAK1,PCMTD2,TRIO,SETD2,KIRREL1,AMBRA1,ALPK3,MYO3B,NRP1,FRY,RC3H1,RPS6KA5,TAOK3,HERC2,ABL2,PRKCB,SLC8A1,LYPLA1,MAN2A2,LNPEP,CDK12,GALNT13,NEK4,USP14,ST8SIA4,CNKSR3,TRIM2,SLC8A3,MGAT5,USP24,SLCO3A1,PDZRN3,PIK3C3,DCLK1,CDC42BPA,NIPBL,MARCHF6,CCNG2,PJA2,ASB3,NEK6,MOB1B,HECW2,PEAK1,EYA1,KSR1,TTC3,TTLL7,ST8SIA1,FYN,CREBBP,ZBTB16,PRMT8,B4GALT6,STK32A,OTUD7A,TRABD2B,SPRED1,KANSL1,DPH6,NOTCH2,PDE4D,PTPRT,PRKACB,PTPN4,GXYLT2,HIPK3,TRIM9,WSB1,LAMA1,MAST4,NRXN1,NTRK3,PRRC1,NEK10,PIAS1,ST6GALNAC3,PTAR1,GALNT10,HMGA2,HHAT,PARP8,ZDHHC17,PKN2,ROCK1,HERC1,UBR1,PRKAA2,PRKN,TRPM7,SIAH2,LATS2,PTPRD,CDC14B,PTPRN2,HDAC4,MYO3A,NTRK2,MAPK1,CUL5,PRKG1,BIRC6,DIP2B,NLK,SPOP,EGFR,PTPRQ,BCR,MAGI2,LARGE1,FANCM,FUT9,GALNTL6,MOK,PDP2,RNF152,ZNRF3,GNAQ,ITPKB,GRM5,MED1,MTMR7,EPHA6,PID1,ABL1,PTPRG,PIGN,PCMTD1,PPM1L,FER,PAK5,SETDB2,FBXL17,GALNT14,PRLR,MTMR2,FBXL7,FGF10,NOS1AP,CADM1,RC3H2,MSRA,STK32B,ZDHHC21,RAPGEF2,INSR,PDK1,KLF*

			15,ARFGEF1,ENPP1,ULK2,PPM1F,EGLN3,GFRA1,BMPR1B,CPE,KITLG,MAP4K4,DOCK3,PRAME,APC,MARCHF1,RELN,MACROD2,EOGT,AUTS2,ROR1,PPP2R3A,MTMR3,BMP2K,NDFIP1,PTPN13,AKT3,ST8SIA6,PRKCA,KMT2C,USP8,ERC1,APP,RPS6KA3,SMAD5,ALPK2,ASB2,EYA2,MAPK8IP1,ADGRB1,CCDC88A
GO:0014706	striated muscle tissue development	0.000244607003728048	*ERBB4,ALPK3,SLC8A1,ADAMTS9,NEBL,EYA1,TPM1,NOTCH2,SGCG,FHOD3,PRKG1,AKAP6,LARGE1,MED1,ABL1,JARID2,LRP2,RYR2,MTPN,SOX6,SGCD,AKAP13,PGM5,MRTFB,PDLIM5,SMAD5,ALPK2,ASB2,EYA2*
GO:0099175	regulation of postsynapse organization	0.000247029971554687 7	*RPS6KA5,STAU2,TANC1,DISC1,ASAP1,FYN,GRIN2B,DGKB,NRXN1,GRID1,DNM3,PTPRD,PPFIA2,NLGN1,GRID2,SHANK2,RELN,PDLIM5*
GO:0070727	cellular macromolecule localization	0.000267521668848957 4	*SLMAP,ERBB4,FYB2,CUBN,MACF1,PAK1,SETD2,SLC15A5,NETO2,DOP1B,NRP1,TANGO2,HERC2,PRKCB,GPC5,LYPLA1,STAU2,SIAH3,TBC1D5,EXOC6B,RFX3,PIK3C3,DCLK1,DNAH11,ADAMTS9,NIPBL,CEP120,GRIK2,SNX25,IGSF11,CCDC186,ODR4,CORO2B,ITGA8,SYCP1,VPS41,ASB3,TOM1L2,TTC21B,COG2,DISC1,CLTCL1,RIC3,GPR158,APBA2,LYST,FRMD6,FYN,CREBBP,ZBTB16,CADPS,LRBA,SGTB,LRP1B,AP5M1,MYO5A,CACNG2,TSPAN33,NPIPA1,ESR1,TJP1,RIMS1,FCHSD2,GLI3,NRXN1,FRMD4A,FBN1,LONP2,WDPCP,RAPGEF4,EHBP1,DPP6,SEL1L,ZDHHC17,ITGA4,ROCK1,PRKAA2,RGPD4,PACRG,BBS2,PRKN,LATS2,EXOC4,DLG2,SCFD2,BBS9,PTPRN2,RANBP3L,PLCB1,SDCBP,NBEA,EGFR,BCR,MAGI2,AKAP6,LARGE1,STX12,TBC1D1,MED1,GNPTAB,SPIRE1,WDR72,SNX30,PID1,NLGN1,ABL1,JARID2,GRID2,LRP2,RANBP17,ERBIN,MON2,RYR2,SCAMP1,MCC,CACNB2,STXBP4,MAPRE2,FGF10,KIF13A,IPO11,VCL,ZDHHC21,RAPGEF2,ILDR2,IMMP2L,DENND4C,ARFGEF1,TANGO6,NRXN3,RIMS2,PPM1F,KCNQ3,SNX8,NSG1,CPE,PYGO1,MAP4K4,EFR3A,APC,APLF,GPC6,PARD3B,ADGRV1,RELN,AKAP11,VPS13C,NDFIP1,SNAP91,CRB1,FCHO2,HEPACAM,USP8,USH1C,FMN2,ERC1,DNAJC13,CCDC88A,RAP1GDS1*
GO:0031346	positive regulation of cell projection organization	0.00030640890611444697	*MACF1,MYO3B,NRP1,EPS8,ABL2,ARHGEF7,STAU2,CEP120,CDC42EP3,DISC1,FYN,ARSB,NEGR1,TOX,NRXN1,NTRK3,DNM3,ARHGAP32,PTPRD,MYO3A,NTRK2,DSCAM,MAGI2,FUT9,CNTN1,TENM3,NLGN1,ABL1,RAPGEF2,SEMA5A,ATP8A2,APC,RELN,AUTS2,ROR1,CCDC88A*
GO:0048813	dendrite morphogenesis	0.000322700295066569 7	*CTNND2,NRP1,STAU2,SEMA3A,DCLK1,SDC2,PHACTR1,HECW2,FYN,RERE,DNM3,PTPRD,ADGRB3,DSCAM,PPFIA2,DOCK10,NLGN1,RAPGEF2,CTNNA2,RELN,PDLIM5*
GO:0007265	Ras protein signal transduction	0.000361540934906881 9	*NRP1,CDH13,EPS8,ABL2,ARHGEF12,STARD13,RGL1,RALGPS1,ARHGAP42,KSR1,NOTCH2,MADD,RAPGEF4,ROCK1,ARHGEF11,SDCBP,DGKI,BCR,ARHGEF28,ITPKB,ABL1,ELMO1,MAPRE2,*

			FGF10,RAPGEF2,RAPGEF5,AKAP13,DENND4C,ARFGEF1,KITLG,ARHGAP24,MAP4K4,AUTS2,USP8,PSD3
GO:0051130	positive regulation of cellular component organization	0.000416986384345274 8	*MACF1,PAK1,KIRREL1,AMBRA1,MYO3B,NRP1,EPS8,ABL2,ARHGEF7,STAU2,FYCO1,TBC1D5,CEP120,CDC42EP3,CLSTN2,ADGRL2,ATP10A,DISC1,FMN1,ASAP1,FRMPD4,FYN,ARSB,TRABD2B,NEGR1,TPM1,TOX,ERC2,FCHSD2,NRXN1,NTRK3,WASF3,ROCK1,DNM3,SYT1,ARHGAP32,PRKN,PTPRD,ADGRB3,MYO3A,NTRK2,PLCB1,MAPK1,SDCBP,DSCAM,BCR,MAGI2,MELTF,FUT9,ANKFY1,CNTN1,TENM3,SPIRE1,SNX30,ATP8A1,NLGN1,ABL1,GRID2,FER,SETDB2,NFATC2,MAPRE2,PDE4DIP,RAPGEF2,INSR,SYNJ1,PPM1F,SEMA5A,ATP8A2,MAP4K4,APC,RELN,AUTS2,ROR1,USP8,APP,ABCA13,ADGRB1,CCDC88A*
GO:0033036	macromolecule localization	0.000508975018577836 8	*SLMAP,ERBB4,FYB2,CUBN,MACF1,PAK1,ATP9B,SETD2,SLC15A5,NETO2,DOP1B,NRP1,TANGO2,HERC2,PRKCB,GPC5,LYPLA1,STAU2,SIAH3,TBC1D5,EXOC6B,PLIN2,RFX3,RBFOX1,SLCO3A1,ATP11C,PIK3C3,DCLK1,DNAH11,ADAMTS9,NIPBL,CEP120,GRIK2,SNX25,IGSF11,CCDC186,SLC25A21,ODR4,CORO2B,ITGA8,SYCP1,VPS41,ASB3,TOM1L2,ATP10A,TTC21B,ABCD2,COG2,DISC1,CLTCL1,RIC3,GPR158,APBA2,LYST,FRMD6,FYN,CREBBP,ZBTB16,CADPS,LRBA,SGTB,LRP1B,AP5M1,MYO5A,CACNG2,TSPAN33,NPIPA1,ESR1,TJP1,RIMS1,FCHSD2,GLI3,NRXN1,FRMD4A,FBN1,LONP2,WDPCP,RAPGEF4,EHBP1,DPP6,PRELID2,SEL1L,ZDHHC17,ITGA4,ROCK1,PRKAA2,RGPD4,PACRG,BBS2,SLC27A6,PRKN,LATS2,EXOC4,DLG2,SCFD2,BBS9,PTPRN2,RANBP3L,PLCB1,SDCBP,ATP9A,NBEA,EGFR,BCR,MAGI2,AKAP6,LARGE1,STX12,TBC1D1,MED1,GNPTAB,SPIRE1,WDR72,SNX30,ATP8A1,PID1,NLGN1,ABL1,JARID2,GRID2,LRP2,RANBP17,ERBIN,MON2,RYR2,SCAMP1,MCC,CACNB2,STXBP4,VSTM2A,MAPRE2,FGF10,KIF13A,IPO11,VCL,ZDHHC21,XKR6,RAPGEF2,ILDR2,IMMP2L,DENND4C,ARFGEF1,ENPP1,TANGO6,NRXN3,RIMS2,PPM1F,KCNQ3,SNX8,NSG1,CPE,ATP8A2,PYGO1,MAP4K4,EFR3A,APC,APLF,GPC6,PARD3B,ABCA5,ADGRV1,RELN,AKAP11,VPS13C,NDFIP1,SNAP91,CRB1,FCHO2,RFTN2,HEPACAM,USP8,USH1C,FMN2,ERC1,DNAJC13,VPS13B,ABCA13,CCDC88A,RAP1GDS1*
GO:1902531	regulation of intracellular signal transduction	0.000518908572083241 6	*ERBB4,PAK1,TRIO,NRP1,CDH13,RC3H1,EPS8,TAOK3,ABL2,PRKCB,ARHGEF12,ANKRD6,HTR2C,CNKSR3,SEMA3A,STARD13,LIMD1,FGD4,GRIK2,SIPA1L2,PJA2,NEK6,ARHGAP28,RALGPS1,ARHGAP42,KSR1,EDAR,FRMD6,FYN,SRGAP2,SPRED1,GARNL3,NOTCH2,MADD,ESR1,NFAT5,PDE4D,ARHGAP26,HIPK3,SGMS1,JCAD,NRXN1,NTRK3,NEK10,PHLPP1,ZDHHC17,ROCK1,ARHGAP31,UBR1,PRKAA2,ARHGEF11,ARHGAP32,PRKN,RORA,DGKG,MYO9A,NTRK2,PLCB1,MAPK1,SDCBP,DGKI,EGFR,BCR,MAGI2,AKAP6,AR*

			HGEF28,IQCJ-SCHIP1,RNF152,ITPKB,GRM5,NLGN1,ABL1,NCOR1,LRP2,PAK5,MAPRE2,FGF10,NOS1AP,RC3H2,SEZ6L,RAPGEF2,SHANK2,ZMYND11,INSR,SRGAP3,AKAP13,DENND4C,ARFGEF1,DENND2B,MAPKBP1,SEMA5A,KITLG,ARHGAP24,MAP4K4,DOCK3,ADGRV1,RELN,AUTS2,ROR1,SNAI2,NDFIP1,AKT3,VAV3,PRKCA,APP,PSD3,MAPK8IP1,RAP1GDS1
GO:0007626	locomotory behavior	0.000544609362154569 2	EPS8,HTR2C,APBA2,NEGR1,NAV2,FGF12,BTBD9,PCDH15,PBX3,SLC4A10,PRKN,DSCAM,LARGE1,CNTN1,GRM5,NCOR1,PAK5,GNG7,SHANK2,ZFHX3,ASTN1,KCND2,RELN,TNR,APP,SLC6A3
GO:0007411	axon guidance	0.000597065481933205 1	TRIO,NRP1,RPS6KA5,CNTN4,SEMA3A,ALCAM,NCAM1,FYN,SLIT3,NOTCH2,GLI3,NRXN1,PTPRD,DSCAM,NFIB,CNTN1,EPHA6,SEMA6D,UNC5D,NRXN3,BMPR1B,SEMA5A,RELN,CYFIP2,ADAMTSL1,TNR,CNTN6,APP
GO:0097485	neuron projection guidance	0.000597065481933205 1	TRIO,NRP1,RPS6KA5,CNTN4,SEMA3A,ALCAM,NCAM1,FYN,SLIT3,NOTCH2,GLI3,NRXN1,PTPRD,DSCAM,NFIB,CNTN1,EPHA6,SEMA6D,UNC5D,NRXN3,BMPR1B,SEMA5A,RELN,CYFIP2,ADAMTSL1,TNR,CNTN6,APP
GO:2001222	regulation of neuron migration	0.000669163082052948 1	SEMA3A,NIPBL,PHACTR1,SRGAP2,SRGAP2C,ARHGAP32,NRG3,NTNG1,RAPGEF2,UNC5D,CTNNA2,RELN
GO:0098815	modulation of excitatory postsynaptic potential	0.000669163082052948 1	SLC8A3,GRIK2,IGSF11,GRIN2B,RIMS1,NRXN1,NLGN1,MTMR2,RIMS2,RELN,CELF4,APP
GO:0006811	monoatomic ion transport	0.000863756424005231 6	SLMAP,KCNMA1,SLC17A1,SLC6A11,PRKCB,SLC23A2,UTRN,SLC8A1,HTR2C,KCNC1,CACNA1E,TMEM63C,AFG3L2,CNKSR3,SLC8A3,SLC40A1,SLCO3A1,ATP11C,SLC9C1,GRIK2,NKAIN3,GRIK4,GABRG1,ATP10A,SLC39A12,HECW2,PIEZO2,GABRB1,ATP2B2,FYN,NALCN,SLC1A2,RGS7,GRIN2B,GRIA1,FGF12,CACNG2,CLDN10,GABRG3,PDE4D,ITPR2,DPP6,GRID1,CACNA2D3,SLC4A10,ABCC9,CACNA1C,TRPM7,TG,PLCB1,GRIK1,CUL5,PRKG1,CLCN5,AKAP6,LARGE1,MELTF,SLC12A1,SCN2A,CNTN1,GRM5,ATP8A1,ABL1,GRID2,LRP2,RYR2,GRIK3,CACNB2,KCNH5,LRRC8B,NOS1AP,TRPM3,UNC80,GABRG2,KCNQ3,SCN8A,KCND2,SLC24A3,GRIA4,RELN,KCNJ15,CYBRD1,SLC4A4,NDFIP1,OCA2,KCNS3,SLC6A3
GO:0016310	phosphorylation	0.001019501923719903 4	ERBB4,PAK1,TRIO,KIRREL1,AMBRA1,ALPK3,MYO3B,NRP1,RPS6KA5,TAOK3,ABL2,PRKCB,SLC8A1,CDK12,NEK4,CNKSR3,SLC8A3,SLCO3A1,LIMD1,PIK3C3,DCLK1,CDC42BPA,CCNG2,NEK6,MOB1B,PEAK1,KSR1,FYN,SH3KBP1,STK32A,SPRED1,DGKB,NOTCH2,PDE4D,PTPRT,PRKACB,PTPN4,HIPK3,SGMS1,LAMA1,MAST4,NRXN1,NTRK3,PRRC1,NEK10,HMGA2,PKN2,ROCK1,PRKAA2,GRB10,PRKN,TRPM7,LATS2,DGKG,M

			YO3A,NTRK2,MAPK1,SDCBP,PRKG1,DSCAM,BIRC6,DGKI,NLK,EGFR,BCR,MAGI2,LARGE1,MOK,GNAQ,ITPKB,GRM5,GNPTAB,EPHA6,LDB2,PID1,ABL1,FER,PAK5,PRLR,FGF10,CADM1,STK32B,RAPGEF2,INSR,PDK1,PPIP5K2,ENPP1,ULK2,PPM1F,GFRA1,BMPR1B,KITLG,MAP4K4,DOCK3,APC,RELN,ROR1,BMP2K,PTPN13,AKT3,VAV3,PRKCA,ERC1,APP,RPS6KA3,SMAD5,ALPK2,MAPK8IP1,CCDC88A
GO:0007015	actin filament organization	0.001131830446769061	*PAK1,KIRREL1,NRP1,EPS8,CDC42EP3,MYO5C,CORO2B,NEBL,FAM171A1,PHACTR1,ARHGAP28,FMN1,SVIL,SH3KBP1,MYO5A,TPM1,PCDH15,TMOD2,TJP1,FCHSD2,WASF3,FHOD3,MYO1E,ROCK1,PRKN,SPIRE1,ABL1,MICAL3,ELMO1,FER,MTPN,ARFGEF1,FAT1,RHPN2,PPM1F,SEMA5A,CTNNA2,CYFIP2,USH1C,FMN2,CCDC88A*
GO:0032970	regulation of actin filament-based process	0.001196639770144519	*PAK1,KIRREL1,MYO3B,NRP1,EPS8,STAU2,CDC42EP3,CORO2B,ARHGAP28,FMN1,SVIL,FRMD6,TPM1,TMOD2,PDE4D,TJP1,FCHSD2,WASF3,FHOD3,ROCK1,CACNA1C,PRKN,MYO3A,ABL1,FER,RYR2,MTPN,AKAP13,ARFGEF1,RHPN2,PPM1F,SEMA5A,CTNNA2,CYFIP2,BCAS3,CCDC88A*
GO:0032879	regulation of localization	0.001432866037353898	*SLMAP,ERBB4,KCNMA1,SETD2,NETO2,NRP1,CDH13,ABL2,PRKCB,ARHGEF7,UTRN,GPC5,SLC8A1,LYPLA1,HTR2C,KCNC1,DYSF,CACNA1E,SIAH3,TBC1D5,CNKSR3,PLIN2,RFX3,DCLK1,CEP120,NKAIN3,CORO2B,SYCP1,TTC21B,CLTCL1,SV2B,HECW2,RIC3,FYN,NALCN,CREBBP,SLC1A2,CADPS,RGS7,GRIN2B,FGF12,CACNG2,BTBD9,CLDN10,PDE4D,ERC2,RIMS1,GLI3,ITPR2,NRXN1,FRMD4A,WDPCP,RAPGEF4,DPP6,CACNA2D3,ABCC9,GSG1L,ROCK1,CACNA1C,PRKAA2,DNM3,SYT1,GRB10,PRKN,LATS2,PTPRN2,PLCB1,MAPK1,SDCBP,ATP9A,PRKG1,NBEA,EGFR,PPFIA2,STXBP6,BCR,MAGI2,AKAP6,ANKFY1,TBC1D1,SCN2A,CNTN1,GRM5,AIMP1,ATP8A1,PID1,NLGN1,ABL1,FER,RYR2,CACNB2,STXBP4,VSTM2A,MTMR2,KCNH5,FGF10,NOS1AP,VCL,INSR,WDR41,KLF15,ENPP1,RIMS2,PPM1F,KCNQ3,SCN8A,KCND2,ATP8A2,SV2C,MAP4K4,CNIH3,APC,GPC6,ABCA5,RELN,KCNJ15,BMP2K,NDFIP1,SNAP91,USP8,KCNS3,APP,DNAJC13,ABCA13,CCDC88A,GHRH,RAP1GDS1*
GO:0098742	cell-cell adhesion via plasma-membrane adhesion molecules	0.001434660978009308	*CDH13,LRFN5,CNTN4,PCDH7,LRRC4C,CADM2,ALCAM,HMCN1,IGSF11,CLSTN2,CLDN10,PCDH15,PTPRT,NRXN1,CDH20,PTPRD,DSCAM,TENM3,NLGN1,GRID2,FAT3,NTNG1,CADM1,PCDH9,VCAM1,CDH7,UNC5D,FAT1,CRB1,CNTN6*
GO:0010976	positive regulation of neuron projection development	0.001464897018879556 3	*NRP1,ABL2,STAU2,DISC1,FYN,ARSB,NEGR1,TOX,NRXN1,NTRK3,NTRK2,MAGI2,FUT9,CNTN1,TENM3,NLGN1,ABL1,RAPGEF2,ATP8A2,RELN,ROR1*
GO:001604	cellular	0.00174011	*HPSE2,ERBB4,KCNMA1,APBB2,MACF1,PAK1,C*

3	component organization	41275164103	*TNND2,FRMD3,SCAF8,COL22A1,ATP9B,TRIO,MITF,RTN1,SETD2,KIRREL1,AMBRA1,CHD9,SYNE2,DOP1B,MYO3B,NRP1,TEAD1,NFIA,FRY,CDH13,RC3H1,RPS6KA5,EPS8,TANGO2,TAOK3,LRFN5,ABL2,PRKCB,SLC23A2,ARHGEF7,PHACTR3,EML1,PRR16,RERG,KCNC1,DYSF,STAU2,SIAH3,FYCO1,CNTN4,TBC1D5,EXOC6B,AFG3L2,FRMD5,SEMA3A,PLIN2,SLC8A3,RFX3,STARD13,MMP16,LRRC4C,ALCAM,ATP11C,PDZRN3,LIMD1,PIK3C3,DCLK1,ADAMTS9,CDC42BPA,NIPBL,CEP120,CDC42EP3,CHD6,HMCN1,FGD4,MYO5C,SDC2,NCAM1,CLSTN2,KDM4B,ADGRL2,TANC1,CORO2B,NEBL,ITGA8,SYCP1,VPS41,MYO10,TCF4,TOM1L2,FAM171A1,ATP10A,PHACTR1,BICRAL,TTC21B,NEK6,ABCD2,SLC39A12,COG2,ARHGAP28,DISC1,HECW2,RIC3,FMN1,PEAK1,EYA1,SVIL,ASAP1,TTLL7,LYST,FRMPD4,COL23A1,PLEKHA3,FYN,SH3KBP1,AGO2,CREBBP,SLC1A2,PRMT8,CSMD3,LRBA,B4GALT6,SLIT3,SGTB,GRIN2B,ANTXR1,SRGAP2,ARSB,GRIA1,TRABD2B,NEGR1,MYO5A,NAV2,DGKB,CACNG2,TSPAN33,KANSL1,BTBD9,TPM1,COL19A1,NOTCH2,FLNB,TOX,CLDN10,PCDH15,ESR1,NFAT5,SRGAP2C,TMOD2,TJP1,ARHGAP26,ERC2,PTPRT,PRKACB,RIMS1,WSB1,FCHSD2,PARVB,LAMA1,GLI3,GOLGA8S,MAST4,NRXN1,SUPT16H,NTRK3,WASF3,LONP2,ARID1B,HYDIN,TRAPPC10,TSPAN2,WDPCP,MAGI1,CNKSR2,ANKRD31,PIAS1,CDH20,PRELID2,HMGA2,CTTNBP2,FHOD3,GRID1,RERE,ATL1,MYO1E,ZDHHC17,PKN2,ITGA4,HFM1,GSG1L,ROCK1,HERC1,CFAP70,DOCK1,PRKAA2,ARHGEF11,CCSER2,COL4A2,DNM3,SYT1,BBS2,ARHGAP32,PRKN,DPF3,TRPM7,SIAH2,EXOC4,SACS,PTPRD,CDC14B,BBS9,TLN2,HDAC4,ADGRB3,DGKG,MYO9A,MYO3A,NTRK2,PLCB1,MAPK1,LAMB1,SDCBP,ATP9A,BPTF,TASOR2,PRKG1,DSCAM,NFIB,DIP2B,NBEA,EGFR,PPFIA2,STXBP6,NCAM2,BCR,MAGI2,AKAP6,LARGE1,TRAPPC8,RTTN,STX12,FANCM,ARHGEF28,MELTF,FUT9,ANKFY1,IQCJ-SCHIP1,SLC12A1,CNTN1,DOCK10,TENM3,GRM5,MED1,GNPTAB,EPHA6,SPIRE1,PHACTR2,COL14A1,WDR72,SNX30,NRG3,ATP8A1,PID1,NLGN1,ABL1,PTPRG,NCOR1,JARID2,MICAL3,GRID2,ELMO1,LRP2,ERBIN,SEMA6D,CABIN1,KHDC4,SMOC1,FER,PAK5,SETDB2,PRLR,NFATC2,DST,FAT3,CACNB2,NTNG1,MED15,MTPN,MTMR2,LRP12,MAPRE2,FGF10,KIF13A,NOS1AP,PDE4DIP,MPDZ,YLPM1,TRPM3,VCL,SEZ6L,XKR6,RAPGEF2,CDH7,IFT81,SHANK2,ZMYND11,INSR,AKAP13,IMMP2L,SYNJ1,CHCHD6,UNC5D,KDM1B,GABRG2,ARFGEF1,FAT1,PATJ,ENPP1,ULK2,UNC13C,ZNF462,NRXN3,RIMS2,RHPN2,PPM1F,KCNQ3,PGM5,GFRA1,BMPR1B,NSG1,KCND2,SEMA5A,ATP8A2,GOLGA8T,PYGO1,ARHGAP24,MAP4K4,CTNNA2,APC,APLF,PARD3B,DNER,ABCA5,ADGRV1,RELN,AKAP11,VPS13C,AUTS2,ROR1,MTMR3,BMP2K,SNAI2,CYFIP2,ADAM*

			TSL1,TNR,PTPN13,SNAP91,OCA2,CELF4,AKT3,CRB1,VAV3,BCAS3,FCHO2,PRKCA,KMT2C,USP8,USH1C,DNAH5,CNTN6,KCNS3,FMN2,ERC1,APP,PDLIM5,RPS6KA3,BTAF1,DNAJC13,NYAP2,VPS13B,ABCA13,ASB2,EYA2,ADGRB1,CCDC88A,RAP1GDS1,SPOCK1
GO:0048646	anatomical structure formation involved in morphogenesis	0.001806857874634180 7	*SETD2,EPN2,NRP1,CDH13,RC3H1,PRKCB,STARD13,SLC40A1,ADAMTS9,KIF16B,TANC1,NEBL,ITGA8,TTC21B,SLC39A12,FMN1,EYA1,EDAR,AGO2,TAFA5,SPRED1,TPM1,NOTCH2,TMOD2,MEGF11,TJP1,PRKACB,JCAD,GLI3,NRXN1,HMGA2,FHOD3,ITGA4,ROCK1,HERC1,DOCK1,COL4A2,LATS2,EXOC4,RORA,ADGRB3,MAPK1,LAMB1,DSCAM,NFIB,THSD7A,LARGE1,CNTN1,MED1,ENPEP,AIMP1,WDR72,NRG3,ABL1,JAM2,GRID2,LRP2,NFATC2,FAT3,MTPN,MTMR2,FGF10,SP3,AKAP13,NRXN3,PGM5,SEMA5A,ATP8A2,ARHGAP24,RELN,PPP2R3A,SNAI2,AKT3,CRB1,VAV3,BCAS3,PRKCA,VPS13B,EYA2,ADGRB1*
GO:0021953	central nervous system neuron differentiation	0.002082372472691734 6	*NRP1,MDGA2,DCLK1,TTC21B,DISC1,GABRB1,B4GALT6,TOX,ZSWIM6,GLI3,BRINP1,NRXN1,SLC4A10,HERC1,RORA,NTRK2,NFIB,ARHGEF28,GRID2,MTPN,RAPGEF2,BMPR1B,SPOCK1*
GO:0050806	positive regulation of synaptic transmission	0.002190532304949294 4	*SLC8A3,GRIK2,IGSF11,CLSTN2,NALCN,GRIN2B,GRIA1,CACNG2,RIMS1,NRXN1,SYT1,NTRK2,MAPK1,LARGE1,NLGN1,ABL1,SHANK2,RIMS2,NSG1,RELN,TNR,APP*
GO:0042127	regulation of cell population proliferation	0.002265555167215828 2	*HPSE2,ERBB4,MACF1,PAK1,MITF,AMBRA1,SYNE2,MYO3B,NRP1,CDH13,RC3H1,EPS8,ABL2,ARHGEF7,DACH1,RERG,STAU2,SEMA3A,LRRC4C,CEP120,CDC42EP3,SDC2,MYO10,PBX1,BICRAL,SLC39A12,DISC1,HECW2,EYA1,KSR1,ST8SIA1,FYN,ZBTB16,CSMD3,SLIT3,GRIN2B,ARSB,TAFA5,NEGR1,TPM1,NOTCH2,TOX,ESR1,SRGAP2C,TJP1,JCAD,GLI3,NRXN1,NTRK3,WDPCP,PIAS1,HMGA2,ITGA4,DNM3,ARHGAP32,PTPRD,HDAC4,ADGRB3,DGKG,MYO9A,MYO3A,NTRK2,MAPK1,LAMB1,SDCBP,PRKG1,DSCAM,BIRC6,NFIB,DIP2B,EGFR,PPFIA2,MAGI2,NELL1,FUT9,CNTN1,TENM3,MED1,AIMP1,PID1,NLGN1,ABL1,PTPRG,JARID2,GRID2,LRP2,SEMA6D,FER,MCC,PRLR,NFATC2,FAT3,STXBP4,NTNG1,VSTM2A,FGF10,VCAM1,RAPGEF2,SHANK2,INSR,ULK2,EGLN3,BMPR1B,SEMA5A,ATP8A2,KITLG,ARHGAP24,MAP4K4,CTNNA2,PRAME,APC,RELN,AUTS2,ROR1,SNAI2,NDFIP1,TNR,AKT3,VAV3,PRKCA,USH1C,ENPP3,APP,PDLIM5,MAPK8IP1,ADGRB1,CCDC88A,GHRH,SPOCK1*
GO:0046578	regulation of Ras protein signal transduction	0.002292027793407568	*NRP1,EPS8,ABL2,STARD13,RALGPS1,ARHGAP42,NOTCH2,MADD,DGKI,BCR,ARHGEF28,ITPKB,ABL1,MAPRE2,FGF10,AKAP13,DENND4C,ARFGEF1,KITLG,ARHGAP24,MAP4K4,AUTS2,PSD3*

GO:0007158	neuron cell-cell adhesion	0.002690169276110357 7	*CNTN4,NRXN1,NCAM2,NLGN1,NRXN3,ASTN1,TNR*
GO:0044057	regulation of system process	0.002732263584165191	*NETO2,SLC8A1,HTR2C,SLC8A3,GRIK2,IGSF11,CORO2B,ASB3,TAFA4,ARHGAP42,ATP2B2,DLGAP1,GRIN2B,FGF12,TPM1,PDE4D,RIMS1,DOCK4,NRXN1,WASF3,PBX3,ABCC9,ROCK1,CACNA1C,PPP1R12B,HDAC4,PRKG1,AKAP6,NLGN1,ABL1,JARID2,JAM2,RYR2,CACNB2,MTPN,MTMR2,FGF10,NOS1AP,ZDHHC21,RIMS2,DLGAP2,RELN,TNR,CELF4,PRKCA,APP*
GO:0051493	regulation of cytoskeleton organization	0.003128619582307573 7	*PAK1,KIRREL1,MYO3B,NRP1,EPS8,ARHGEF7,STAU2,CEP120,CDC42EP3,CORO2B,SLC39A12,ARHGAP28,FMN1,SVIL,TPM1,TMOD2,TJP1,FCHSD2,WASF3,FHOD3,ROCK1,PRKAA2,PRKN,MYO3A,MAPK1,IQCJ-SCHIP1,ABL1,FER,MTPN,MAPRE2,PDE4DIP,MPDZ,AKAP13,ARFGEF1,PATJ,RHPN2,PPM1F,SEMA5A,CTNNA2,APC,CYFIP2,BCAS3,CCDC88A*
GO:0034762	regulation of transmembrane transport	0.003490061954303749	*SLMAP,KCNMA1,PRKCB,UTRN,SLC8A1,KCNC1,CACNA1E,CNKSR3,CLTCL1,HECW2,FYN,NALCN,SLC1A2,RGS7,GRIN2B,FGF12,CACNG2,PDE4D,NRXN1,DPP6,CACNA2D3,ABCC9,GSG1L,CACNA1C,GRB10,PRKG1,AKAP6,SCN2A,GRM5,PID1,NLGN1,ABL1,RYR2,CACNB2,STXBP4,KCNH5,NOS1AP,INSR,KLF15,ENPP1,KCNQ3,SCN8A,KCND2,CNIH3,RELN,KCNJ15,KCNS3,APP*
GO:0007167	enzyme-linked receptor protein signaling pathway	0.003600579897205072	*FSTL1,ERBB4,PAK1,TRIO,EPN2,NRP1,NFIA,CDH13,PRKCB,ARHGEF7,SAMD12,LNPEP,LDLRAD4,SNX25,MBD5,KIF16B,ITGA8,ANKS1B,FYN,CREBBP,SPRED1,CRIM1,NOTCH2,PTPRT,JCAD,NRXN1,NTRK3,FBN1,MYO1E,ZDHHC17,COL4A2,GRB10,LATS2,PTPRD,NTRK2,PLCB1,MAPK1,SDCBP,CUL5,NLK,EGFR,MAGI2,ARHGEF28,EPHA6,NRG3,PID1,ABL1,PTPRG,LRP2,ERBIN,PPM1L,FER,PRLR,STXBP4,GNG7,FGF10,CADM1,ZNF106,RAPGEF2,INSR,ENPP1,GFRA1,BMPR1B,ROR1,CYFIP2,SMAD5,CCDC88A,GHRH*
GO:0003012	muscle system process	0.003938853496486844	*SLMAP,KCNMA1,APBB2,UTRN,SLC8A1,SLC8A3,ASB3,ARHGAP42,FGF12,TPM1,TMOD2,PDE4D,DOCK4,ABCC9,ROCK1,CACNA1C,ARHGEF11,BBS2,PPP1R12B,HDAC4,PRKG1,AKAP6,LARGE1,JARID2,RYR2,CACNB2,MTPN,NOS1AP,SGCD,ZDHHC21,KLF15,ATP8A2,DTNA,PRKCA,PDLIM5,SMAD5,ASB2,RAP1GDS1*
GO:0106027	neuron projection organization	0.004018541959754992	*CTNND2,STAU2,TANC1,ABCD2,FYN,GRIN2B,DNM3,PPFIA2,DOCK10,NLGN1,MTMR2,INSR,RELN,APP,PDLIM5*
GO:0023056	positive regulation of signaling	0.004211820237250751	*ERBB4,MACF1,PAK1,EPN2,EVC,NRP1,CDH13,RC3H1,TAOK3,PRKCB,ARHGEF7,GPC5,ANKRD6,HTR2C,SEMA3A,SLC8A3,MGAT5,GRIK2,IGSF11,MBD5,CLSTN2,ITGA8,PJA2,TTC21B,NEK6,DISC1,KSR1,EDAR,FRMD6,FYN,NALCN,CREBBP,GRIN2B,GRIA1,SPRED1,CACNG2,NOTCH2,MADD,NFAT5,TMOD2,RIMS1,LAMA1,JCAD,NRXN1,NTRK3,WWOX,RAPGEF4,NEK10,ZDHHC17,ROCK1,SYT1,GRB10,PRKN,NTRK2,PLCB1,MAPK1,LAMB1,SDCBP,DGKI,EGFR,AKAP6,LARGE1,*

			IQCJ-SCHIP1,ITPKB,GRM5,MED1,AIMP1,NLGN1,ABL1,LRP2,PRLR,MAPRE2,FGF10,NOS1AP,RC3H2,ATF6,RAPGEF2,SHANK2,INSR,AKAP13,RIMS2,DENND2B,BMPR1B,NSG1,MAPKBP1,SEMA5A,KITLG,MAP4K4,ADGRV1,RELN,AUTS2,ROR1,PPP2R3A,BMP2K,CYFIP2,NDFIP1,TNR,AKT3,IL1R1,PRKCA,USP8,CNTN6,APP,MAPK8IP1,CCDC88A,GHRH
GO:0010647	positive regulation of cell communication	0.004211820237250751	*ERBB4,MACF1,PAK1,EPN2,EVC,NRP1,CDH13,RC3H1,TAOK3,PRKCB,ARHGEF7,GPC5,ANKRD6,HTR2C,SEMA3A,SLC8A3,MGAT5,GRIK2,IGSF11,MBD5,CLSTN2,ITGA8,PJA2,TTC21B,NEK6,DISC1,KSR1,EDAR,FRMD6,FYN,NALCN,CREBBP,GRIN2B,GRIA1,SPRED1,CACNG2,NOTCH2,MADD,NFAT5,TMOD2,RIMS1,LAMA1,JCAD,NRXN1,NTRK3,WWOX,RAPGEF4,NEK10,ZDHHC17,ROCK1,SYT1,GRB10,PRKN,NTRK2,PLCB1,MAPK1,LAMB1,SDCBP,DGKI,EGFR,AKAP6,LARGE1,IQCJ-SCHIP1,ITPKB,GRM5,MED1,AIMP1,NLGN1,ABL1,LRP2,PRLR,MAPRE2,FGF10,NOS1AP,RC3H2,ATF6,RAPGEF2,SHANK2,INSR,AKAP13,RIMS2,DENND2B,BMPR1B,NSG1,MAPKBP1,SEMA5A,KITLG,MAP4K4,ADGRV1,RELN,AUTS2,ROR1,PPP2R3A,BMP2K,CYFIP2,NDFIP1,TNR,AKT3,IL1R1,PRKCA,USP8,CNTN6,APP,MAPK8IP1,CCDC88A,GHRH*
GO:0061061	muscle structure development	0.00471003796554207	*ALPK3,EVC,UTRN,SLC8A1,AFG3L2,RBFOX1,MYEF2,TCF12,TANC1,NEBL,ITGA8,SVIL,TPM1,COL19A1,FLNB,TMOD2,LAMA1,SGCG,ARID1B,PIAS1,FHOD3,DOCK1,DPF3,RORA,RANBP3L,HDAC4,ADGRB3,PLCB1,LAMB1,PRKG1,AKAP6,LARGE1,MBNL1,MED1,JAM2,LRP2,RYR2,NFATC2,MTPN,FGF10,SOX6,SGCD,AKAP13,ZFHX3,PGM5,DNER,PPP2R3A,MRTFB,PDLIM5,ALPK2,ASB2,ADGRB1*
GO:0032956	regulation of actin cytoskeleton organization	0.004823035599300828	*PAK1,KIRREL1,MYO3B,NRP1,EPS8,STAU2,CDC42EP3,CORO2B,ARHGAP28,FMN1,SVIL,TPM1,TMOD2,TJP1,FCHSD2,WASF3,FHOD3,ROCK1,PRKN,MYO3A,ABL1,FER,MTPN,AKAP13,ARFGEF1,RHPN2,PPM1F,SEMA5A,CTNNA2,CYFIP2,BCAS3,CCDC88A*
GO:0043087	regulation of GTPase activity	0.005145843881090785	*ARHGEF7,TBC1D5,FGD4,RGL1,SIPA1L2,EVI5,RALGPS1,ARHGAP42,ASAP1,SGSM1,RGS7,SRGAP2,GARNL3,NTRK3,RAPGEF4,DOCK9,MYO9A,NTRK2,PRKG1,DGKI,BCR,TBC1D1,DOCK10,ARAP2,MAPRE2,RAPGEF2,RAPGEF5,WDR41,ARFGEF1,ARHGAP24,MAP4K4,TBC1D9,VAV3,RAP1GDS1*
GO:0099003	vesicle-mediated transport in synapse	0.005415382690177777	*PRKCB,SV2B,CADPS,BTBD9,ERC2,RIMS1,NRXN1,RAPGEF4,SYN2,GSG1L,ROCK1,AMPH,DNM3,SYT1,PRKN,PPFIA2,NLGN1,SYNJ1,UNC13C,RIMS2,NSG1,SV2C,SNAP91,FCHO2,ABCA13*
GO:0099643	signal release from synapse	0.00596213173737856	*PRKCB,DYSF,SV2B,CADPS,ERC2,RIMS1,NRXN1,SYN2,SYT1,PRKN,PTPRN2,DGKI,PPFIA2,NLGN1,SYNJ1,UNC13C,NRXN3,RIMS2,SV2C*

GO:0007269	neurotransmitter secretion	0.00596213173737856	*PRKCB,DYSF,SV2B,CADPS,ERC2,RIMS1,NRXN1,SYN2,SYT1,PRKN,PTPRN2,DGKI,PPFIA2,NLGN1,SYNJ1,UNC13C,NRXN3,RIMS2,SV2C*
GO:0140058	neuron projection arborization	0.005980690596810933	*MACF1,NRP1,SEMA3A,PHACTR1,ROCK1,MYO9A,NLGN1,LRP2,NTNG1*
GO:0006468	protein phosphorylation	0.006362247941254857	*ERBB4,PAK1,TRIO,KIRREL1,ALPK3,MYO3B,NRP1,RPS6KA5,TAOK3,ABL2,PRKCB,SLC8A1,CDK12,NEK4,CNKSR3,SLC8A3,SLCO3A1,PIK3C3,DCLK1,CDC42BPA,CCNG2,NEK6,MOB1B,PEAK1,KSR1,FYN,STK32A,SPRED1,NOTCH2,PDE4D,PTPRT,PRKACB,PTPN4,HIPK3,LAMA1,MAST4,NRXN1,NTRK3,PRRC1,NEK10,HMGA2,PKN2,ROCK1,PRKAA2,PRKN,TRPM7,LATS2,MYO3A,NTRK2,MAPK1,PRKG1,BIRC6,NLK,EGFR,BCR,LARGE1,MOK,GNAQ,ITPKB,GRM5,EPHA6,PID1,ABL1,FER,PAK5,PRLR,FGF10,CADM1,STK32B,RAPGEF2,INSR,PDK1,ENPP1,ULK2,PPM1F,GFRA1,BMPR1B,KITLG,MAP4K4,DOCK3,APC,RELN,ROR1,BMP2K,PTPN13,AKT3,PRKCA,ERC1,APP,RPS6KA3,SMAD5,ALPK2,MAPK8IP1,CCDC88A*
GO:0031589	cell-substrate adhesion	0.006646769138503404	*MACF1,NRP1,CDH13,ARHGEF7,UTRN,ADAMTS9,CORO2B,ITGA8,DISC1,FMN1,PEAK1,ANTXR1,SRGAP2,PARVB,WDPCP,ITGA4,ROCK1,DOCK1,TRPM7,EDIL3,LAMB1,PPFIA2,BCR,MELTF,ABL1,FER,VCL,VCAM1,ITGA9,PPM1F,FREM1,MAP4K4,SPOCK1*
GO:0048638	regulation of developmental growth	0.007059341708998513	*ERBB4,MACF1,NRP1,SLC23A2,AFG3L2,SEMA3A,NIPBL,MBD5,DISC1,RIMS1,SYT1,BBS2,ARHGAP32,PRKN,LATS2,PLCB1,DSCAM,DIP2B,AKAP6,ABL1,JARID2,SEMA6D,INSR,ULK2,RIMS2,SEMA5A,ATP8A2,TNR,APP,SLC6A3,GHRH*
GO:0090596	sensory organ morphogenesis	0.007215962859614934	*MYO3B,STAU2,NIPBL,ITGA8,EYA1,RORB,NOTCH2,PCDH15,MEGF11,LRIG1,GLI3,FBN1,WDPCP,MYO3A,NTRK2,MAPK1,DSCAM,PTPRQ,BCR,LARGE1,TENM3,FAT3,FGF10,SP3,FAT1,ATP8A2,PPP2R3A,CRB1,USH1C*
GO:0006996	organelle organization	0.007257681503902507	*ERBB4,MACF1,PAK1,FRMD3,RTN1,SETD2,KIRREL1,AMBRA1,SYNE2,DOP1B,MYO3B,NRP1,NFIA,RC3H1,EPS8,TANGO2,PRKCB,ARHGEF7,PHACTR3,EML1,STAU2,SIAH3,FYCO1,AFG3L2,FRMD5,PLIN2,RFX3,STARD13,LIMD1,PIK3C3,DCLK1,CDC42BPA,NIPBL,CEP120,CDC42EP3,HMCN1,FGD4,MYO5C,CORO2B,NEBL,SYCP1,VPS41,TOM1L2,FAM171A1,PHACTR1,TTC21B,NEK6,ABCD2,SLC39A12,COG2,ARHGAP28,DISC1,HECW2,FMN1,SVIL,ASAP1,TTLL7,LYST,FRMPD4,PLEKHA3,SH3KBP1,LRBA,SRGAP2,ARSB,MYO5A,NAV2,TPM1,FLNB,PCDH15,SRGAP2C,TMOD2,TJP1,ARHGAP26,ERC2,FCHSD2,GOLGA8S,MAST4,NRXN1,WASF3,LONP2,ARID1B,HYDIN,TRAPPC10,WDPCP,ANKRD31,HMGA2,FHOD3,ATL1,MYO1E,HFM1,ROCK1,CFAP70,PRKAA2,ARHGEF11,CCSER2,SYT1,BBS2,PRKN,DPF3,TRPM7,PTPRD,CDC14B,BBS9,TLN2,MYO3A,PLCB1,MAPK1,SDCBP,PRKG1,NFIB,STXBP6,BCR,LARGE1,TRAPPC8,RTTN,STX12,FANCM,ARHGEF*

			28,ANKFY1,IQCJ-SCHIP1,GNPTAB,SPIRE1,PHACTR2,SNX30,PID1,NLGN1,ABL1,NCOR1,MICAL3,GRID2,ELMO1,ERBIN,FER,PAK5,SETDB2,DST,MTPN,MAPRE2,FGF10,KIF13A,NOS1AP,PDE4DIP,MPDZ,YLPM1,IFT81,SHANK2,INSR,AKAP13,IMMP2L,SYNJ1,CHCHD6,ARFGEF1,FAT1,PATJ,ULK2,UNC13C,NRXN3,RHPN2,PPM1F,PGM5,SEMA5A,ATP8A2,GOLGA8T,PYGO1,CTNNA2,APC,PARD3B,RELN,AKAP11,VPS13C,MTMR3,CYFIP2,SNAP91,OCA2,AKT3,VAV3,BCAS3,PRKCA,USP8,USH1C,DNAH5,FMN2,PDLIM5,DNAJC13,VPS13B,ASB2,EYA2,CCDC88A,RAP1GDS1
GO:0043547	positive regulation of GTPase activity	0.007427156021617341 5	ARHGEF7,TBC1D5,RGL1,SIPA1L2,EVI5,RALGPS1,ARHGAP42,ASAP1,SGSM1,RGS7,SRGAP2,GARNL3,NTRK3,RAPGEF4,DOCK9,MYO9A,BCR,TBC1D1,DOCK10,ARAP2,MAPRE2,RAPGEF2,RAPGEF5,WDR41,ARHGAP24,MAP4K4,TBC1D9,RAP1GDS1
GO:0043010	camera-type eye development	0.007521626248392931	PDE6A,MITF,NRP1,NFIA,DCLK1,RORB,ATP2B2,SPRED1,NOTCH2,MEGF11,LAMA1,GLI3,FBN1,WDPCP,CACNA1C,SCAPER,NTRK2,DSCAM,NFIB,EGFR,LARGE1,TENM3,MED1,FAT3,FGF10,SP3,FAT1,BMPR1B,ATP8A2,CELF4,CRB1,USH1C,SLC6A3
GO:1901890	positive regulation of cell junction assembly	0.008240909143781712	NRP1,STAU2,CLSTN2,ADGRL2,FMN1,NRXN1,ROCK1,PTPRD,ADGRB3,NTRK2,NLGN1,ABL1,GRID2,PPM1F,MAP4K4,ADGRB1
GO:0001505	regulation of neurotransmitter levels	0.008264095241244811	SLC6A11,PRKCB,DYSF,SV2B,SLC1A2,CADPS,ERC2,RIMS1,NRXN1,SYN2,SYT1,PRKN,PTPRN2,DGKI,PPFIA2,NLGN1,SYNJ1,UNC13C,NRXN3,RIMS2,SV2C,SLC6A3
GO:0050954	sensory perception of mechanical stimulus	0.008264095241244811	MYO3B,NIPBL,EYA1,PIEZO2,ATP2B2,FYN,NAV2,PCDH15,TJP1,LRIG1,MYO3A,PTPRQ,LARGE1,TMPRSS3,LRP2,PPIP5K2,KCNQ3,LHFPL3,ADGRV1,ROR1,SNAI2,USH1C
GO:0099504	synaptic vesicle cycle	0.008861769359363137	PRKCB,SV2B,CADPS,BTBD9,ERC2,RIMS1,NRXN1,RAPGEF4,SYN2,ROCK1,AMPH,DNM3,SYT1,PRKN,PPFIA2,NLGN1,SYNJ1,UNC13C,RIMS2,SV2C,SNAP91,FCHO2,ABCA13
GO:0007507	heart development	0.009127653395611767	ERBB4,SETD2,ALPK3,NRP1,SLC8A1,DNAH11,ADAMTS9,NIPBL,NEBL,EYA1,SLIT3,SPRED1,FGF12,TPM1,NOTCH2,GLI3,NTRK3,SGCG,FBN1,FHOD3,GREB1L,ROCK1,CACNA1C,MAPK1,PRKG1,EGFR,AKAP6,LARGE1,MED1,ABL1,JARID2,LRP2,SETDB2,RYR2,SOX6,SGCD,VCAM1,INSR,AKAP13,CPE,APC,SNAI2,DNAH5,PDLIM5,SMAD5,ALPK2,ASB2
GO:0043412	macromolecule modification	0.010527459062602848	ERBB4,KLHL13,RNF38,PAK1,PCMTD2,TRIO,SETD2,KIRREL1,AMBRA1,ALPK3,MYO3B,NRP1,FRY,RC3H1,RPS6KA5,TAOK3,HERC2,ABL2,PRKCB,SLC8A1,LYPLA1,MAN2A2,LNPEP,CDK12,GALNT13,NEK4,USP14,ST8SIA4,CNKSR3,TRI

			M2,SLC8A3,MGAT5,USP24,SLCO3A1,PDZRN3,PIK3C3,DCLK1,CDC42BPA,NIPBL,MARCHF6,CCNG2,PJA2,ASB3,NEK6,MOB1B,HECW2,PEAK1,EYA1,KSR1,TTC3,TTLL7,ST8SIA1,FYN,CREBBP,ZBTB16,PRMT8,B4GALT6,STK32A,OTUD7A,TRABD2B,SPRED1,KANSL1,DPH6,NOTCH2,TOX,PDE4D,PTPRT,PRKACB,PTPN4,GXYLT2,HIPK3,TRIM9,WSB1,LAMA1,MAST4,NRXN1,NTRK3,PRRC1,NEK10,PIAS1,ST6GALNAC3,PTAR1,GALNT10,HMGA2,HHAT,PARP8,ZDHHC17,PKN2,ROCK1,HERC1,UBR1,PRKAA2,PRKN,TRPM7,SIAH2,LATS2,PTPRD,CDC14B,PTPRN2,HDAC4,MYO3A,NTRK2,MAPK1,CUL5,PRKG1,BIRC6,DIP2B,NLK,SPOP,EGFR,PTPRQ,BCR,MAGI2,LARGE1,FANCM,FUT9,GALNTL6,MOK,PDP2,RNF152,ZNRF3,GNAQ,ITPKB,GRM5,MED1,MTMR7,EPHA6,PID1,ABL1,PTPRG,PIGN,PCMTD1,PPM1L,FER,PAK5,SETDB2,FBXL17,GALNT14,PRLR,MTMR2,FBXL7,FGF10,NOS1AP,CADM1,RC3H2,MSRA,STK32B,ZDHHC21,RAPGEF2,INSR,PDK1,KLF15,ARFGEF1,ENPP1,ULK2,PPM1F,EGLN3,GFRA1,BMPR1B,CPE,KITLG,MAP4K4,DOCK3,PRAME,APC,MARCHF1,RELN,MACROD2,EOGT,AUTS2,ROR1,PPP2R3A,MTMR3,BMP2K,NDFIP1,PTPN13,AKT3,ST8SIA6,PRKCA,KMT2C,USP8,ERC1,APP,RPS6KA3,SMAD5,ALPK2,ASB2,EYA2,MAPK8IP1,ADGRB1,CCDC88A
GO:1902903	regulation of supramolecular fiber organization	0.0112084275334896	*PAK1,KIRREL1,NRP1,EPS8,ARHGEF7,CDC42EP3,CORO2B,SLC39A12,ARHGAP28,FMN1,SVIL,TPM1,TMOD2,TJP1,FCHSD2,WASF3,FHOD3,ROCK1,PRKN,ABL1,FER,MTPN,PDE4DIP,AKAP13,ARFGEF1,RHPN2,PPM1F,SEMA5A,CTNNA2,APC,CYFIP2,USP8,APP,CCDC88A*
GO:0048639	positive regulation of developmental growth	0.011687547262689387	*ERBB4,MACF1,NRP1,SLC23A2,NIPBL,DISC1,RIMS1,SYT1,BBS2,ARHGAP32,PRKN,PLCB1,DSCAM,AKAP6,INSR,RIMS2,SEMA5A,ATP8A2,SLC6A3,GHRH*
GO:0043954	cellular component maintenance	0.01220990858632214	*KIRREL1,TANC1,FYN,GRIN2B,TJP1,ERC2,NRXN1,ADGRB3,NLGN1,MTMR2,SHANK2,INSR,ERC1*
GO:0007605	sensory perception of sound	0.012854127967874673	*MYO3B,NIPBL,EYA1,ATP2B2,NAV2,PCDH15,TJP1,LRIG1,MYO3A,PTPRQ,LARGE1,TMPRSS3,LRP2,PPIP5K2,KCNQ3,LHFPL3,ADGRV1,ROR1,SNAI2,USH1C*
GO:0051093	negative regulation of developmental process	0.014762642765110969	*TRIO,EPN2,NRP1,RC3H1,CDK12,CNTN4,LDLRAD4,SEMA3A,LIMD1,ADAMTS9,PBX1,BICRAL,RORB,ASAP1,ANKRD26,ZBTB16,TAFA5,SPRED1,CRIM1,SRGAP2C,GLI3,BRINP1,FBN1,ROCK1,GLIS1,COL4A2,DNM3,BBS2,PRKN,RORA,RANBP3L,HDAC4,ADGRB3,MAPK1,NFIB,DIP2B,EGFR,BCR,ITPKB,MED1,NLGN1,JARID2,SEMA6D,NFATC2,FAT3,FGF10,SOX6,RC3H2,RAPGEF2,ENPP1,ULK2,ZFHX3,SEMA5A,MAP4K4,PRAME,ABCA5,ADGRV1,SNAI2,NDFIP1,TNR,PTPN13,APP,ALPK2,ADGRB1*

GO:0030335	positive regulation of cell migration	0.015229401398676097	*PAK1,SYNE2,NRP1,CDH13,ABL2,ARHGEF7,SEMA3A,MGAT5,NIPBL,AGO2,SRGAP2C,TJP1,JCAD,DOCK4,NTRK3,ITGA4,DOCK1,ARHGAP32,MAPK1,LAMB1,SDCBP,EGFR,ATP8A1,ABL1,JAM2,SEMA6D,FER,MAPRE2,FGF10,RAPGEF2,INSR,PPM1F,SEMA5A,KITLG,MAP4K4,APC,RELN,SNAI2,AKT3,IL1R1,BCAS3,PRKCA,APP*
GO:0060041	retina development in camera-type eye	0.015508343617645928	*PDE6A,NRP1,NFIA,DCLK1,RORB,ATP2B2,MEGF11,LAMA1,SCAPER,NTRK2,DSCAM,NFIB,LARGE1,MED1,FAT3,BMPR1B,ATP8A2,CELF4,CRB1,USH1C*
GO:0060537	muscle tissue development	0.016198531115462052	*ERBB4,ALPK3,SLC8A1,RBFOX1,ADAMTS9,NEBL,ITGA8,EYA1,SVIL,TPM1,COL19A1,NOTCH2,FLNB,SGCG,FHOD3,HDAC4,PRKG1,AKAP6,LARGE1,MED1,ABL1,JARID2,LRP2,RYR2,MTPN,SOX6,SGCD,AKAP13,PGM5,DNER,MRTFB,PDLIM5,SMAD5,ALPK2,ASB2,EYA2*
GO:0048675	axon extension	0.017570140188901066	*MACF1,NRP1,SEMA3A,ALCAM,DCLK1,DISC1,SLIT3,ARHGAP32,DSCAM,DIP2B,ABL1,SEMA6D,VCL,ULK2,SEMA5A,AUTS2,TNR*
GO:0110053	regulation of actin filament organization	0.017751898405774718	*PAK1,KIRREL1,NRP1,EPS8,CDC42EP3,CORO2B,ARHGAP28,FMN1,SVIL,TPM1,TMOD2,TJP1,FCHSD2,WASF3,FHOD3,ROCK1,PRKN,ABL1,FER,MTPN,ARFGEF1,RHPN2,PPM1F,SEMA5A,CTNNA2,CYFIP2,CCDC88A*
GO:0045927	positive regulation of growth	0.018335285903596222	*ERBB4,MACF1,NRP1,TEAD1,SLC23A2,NIPBL,DISC1,RIMS1,SYT1,BBS2,ARHGAP32,PRKN,PLCB1,SDCBP,DSCAM,EGFR,AKAP6,MTPN,INSR,RIMS2,PPM1F,SEMA5A,ATP8A2,RPS6KA3,SLC6A3,GHRH*
GO:0051049	regulation of transport	0.018454979452577968	*SLMAP,KCNMA1,SETD2,NRP1,CDH13,ABL2,PRKCB,ARHGEF7,UTRN,SLC8A1,LYPLA1,HTR2C,KCNC1,DYSF,CACNA1E,SIAH3,TBC1D5,CNKSR3,RFX3,NKAIN3,TTC21B,CLTCL1,SV2B,HECW2,FYN,NALCN,SLC1A2,CADPS,RGS7,GRIN2B,FGF12,CACNG2,BTBD9,CLDN10,PDE4D,ERC2,RIMS1,GLI3,NRXN1,FRMD4A,RAPGEF4,DPP6,CACNA2D3,ABCC9,GSG1L,ROCK1,CACNA1C,DNM3,SYT1,GRB10,PRKN,PTPRN2,PLCB1,MAPK1,SDCBP,ATP9A,PRKG1,PPFIA2,STXBP6,BCR,MAGI2,AKAP6,ANKFY1,TBC1D1,SCN2A,CNTN1,GRM5,AIMP1,ATP8A1,PID1,NLGN1,ABL1,FER,RYR2,CACNB2,STXBP4,MTMR2,KCNH5,FGF10,NOS1AP,INSR,WDR41,KLF15,ENPP1,RIMS2,PPM1F,KCNQ3,SCN8A,KCND2,ATP8A2,SV2C,MAP4K4,CNIH3,ABCA5,RELN,KCNJ15,BMP2K,NDFIP1,SNAP91,KCNS3,APP,DNAJC13,ABCA13,GHRH,RAP1GDS1*
GO:0051094	positive regulation of developmental process	0.020289281469967474	*ERBB4,MACF1,AMBRA1,NRP1,PRKCB,SLC23A2,ARHGEF7,SLC8A1,HTR2C,STAU2,RFX3,ATP11C,ADAMTS9,NIPBL,CLSTN2,TCF12,ADGRL2,TCF4,BICRAL,SLC39A12,DISC1,AGO2,ZBTB16,SPEN,NOTCH2,TOX,TJP1,RIMS1,LAMA1,JCAD,GLI3,BRINP1,NRXN1,ARID1B,PIAS1,HMGA2,SYT1,BBS2,ARHGAP32,PRKN,DPF3,PTPRD,RANBP3L,ADGRB3,NTRK2,PLCB1,LAMB1,SDCBP,SOX5,DSCAM,NELL1,AKAP6,ITPKB,GRM5,MED1,SPIRE1,NLGN1,ABL1,GRID2,LRP2,NFA*

			TC2,VSTM2A,FGF10,SOX6,RAPGEF2,INSR,RIMS2,ZFHX3,BMPR1B,SEMA5A,ATP8A2,KITLG,ADGRV1,RELN,SNAI2,AKT3,PRKCA,MRTFB,RPS6KA3,SMAD5,SLC6A3,ADGRB1,GHRH
GO:0046328	regulation of JNK cascade	0.021254190288257792	TAOK3,ANKRD6,SEMA3A,GRIK2,PJA2,EDAR,HIPK3,PHLPP1,PRKN,PLCB1,SDCBP,EGFR,NCOR1,ZMYND11,MAPKBP1,MAP4K4,APP,MAPK8IP1
GO:0007169	transmembrane receptor protein tyrosine kinase signaling pathway	0.02191360970493484	ERBB4,PAK1,EPN2,NRP1,CDH13,PRKCB,ARHGEF7,SAMD12,MBD5,KIF16B,ANKS1B,FYN,CRIM1,PTPRT,JCAD,NRXN1,NTRK3,MYO1E,ZDHHC17,COL4A2,GRB10,NTRK2,PLCB1,MAPK1,CUL5,EGFR,ARHGEF28,EPHA6,NRG3,PID1,ABL1,PTPRG,ERBIN,FER,PRLR,STXBP4,FGF10,CADM1,ZNF106,RAPGEF2,INSR,ENPP1,GFRA1,ROR1,CYFIP2,CCDC88A,GHRH
GO:0071840	cellular component organization or biogenesis	0.022218669055758488	HPSE2,ERBB4,KCNMA1,APBB2,MACF1,PAK1,CTNND2,FRMD3,SCAF8,COL22A1,ATP9B,TRIO,MITF,RTN1,SETD2,KIRREL1,AMBRA1,CHD9,SYNE2,DOP1B,MYO3B,NRP1,TEAD1,NFIA,FRY,CDH13,RC3H1,RPS6KA5,EPS8,TANGO2,TAOK3,LRFN5,ABL2,PRKCB,SLC23A2,ARHGEF7,PHACTR3,EML1,PRR16,RERG,KCNC1,DYSF,STAU2,SIAH3,FYCO1,CNTN4,TBC1D5,EXOC6B,AFG3L2,FRMD5,SEMA3A,PLIN2,SLC8A3,RFX3,STARD13,MMP16,LRRC4C,ALCAM,ATP11C,PDZRN3,LIMD1,PIK3C3,DCLK1,ADAMTS9,CDC42BPA,NIPBL,CEP120,CDC42EP3,CHD6,HMCN1,FGD4,MYO5C,SDC2,NCAM1,CLSTN2,KDM4B,ADGRL2,TANC1,CORO2B,NEBL,ITGA8,SYCP1,VPS41,MYO10,TCF4,TOM1L2,FAM171A1,ATP10A,PHACTR1,BICRAL,TTC21B,NEK6,ABCD2,SLC39A12,COG2,ARHGAP28,DISC1,HECW2,RIC3,FMN1,PEAK1,EYA1,SVIL,ASAP1,TTLL7,LYST,FRMPD4,COL23A1,PLEKHA3,FYN,SH3KBP1,AGO2,CREBBP,SLC1A2,PRMT8,CSMD3,LRBA,B4GALT6,SLIT3,SGTB,GRIN2B,ANTXR1,SRGAP2,ARSB,GRIA1,TRABD2B,NEGR1,MYO5A,NAV2,DGKB,CACNG2,TSPAN33,KANSL1,BTBD9,TPM1,COL19A1,NOTCH2,FLNB,TOX,CLDN10,PCDH15,ESR1,NFAT5,SRGAP2C,TMOD2,TJP1,ARHGAP26,ERC2,PTPRT,PRKACB,RIMS1,WSB1,FCHSD2,PARVB,LAMA1,GLI3,GOLGA8S,MAST4,NRXN1,SUPT16H,NTRK3,WASF3,LONP2,ARID1B,HYDIN,TRAPPC10,TSPAN2,WDPCP,MAGI1,CNKSR2,ANKRD31,PIAS1,CDH20,PRELID2,HMGA2,CTTNBP2,FHOD3,GRID1,RERE,ATL1,MYO1E,ZDHHC17,PKN2,ITGA4,HFM1,GSG1L,ROCK1,HERC1,CFAP70,DOCK1,PRKAA2,ARHGEF11,CCSER2,COL4A2,DNM3,SYT1,BBS2,ARHGAP32,PRKN,DPF3,TRPM7,SIAH2,EXOC4,SACS,PTPRD,CDC14B,BBS9,TLN2,HDAC4,ADGRB3,DGKG,MYO9A,MYO3A,NTRK2,PLCB1,MAPK1,LAMB1,SDCBP,ATP9A,BPTF,TASOR2,PRKG1,DSCAM,NFIB,DIP2B,NBEA,EGFR,PPFIA2,STXBP6,NCAM2,BCR,MAGI2,AKAP6,LARGE1,TRAPPC8,RTTN,STX12,FANCM,ARHGEF28,MELTF,FUT9,ANKFY1,IQCJ-SCHIP1,SLC12A1,CNTN1,DOCK10,TENM3,GRM

			5,MED1,GNPTAB,EPHA6,SPIRE1,PHACTR2,LDB2,COL14A1,WDR72,SNX30,NRG3,ATP8A1,PID1,NLGN1,ABL1,PTPRG,NCOR1,JARID2,MICAL3,GRID2,ELMO1,LRP2,ERBIN,SEMA6D,CABIN1,KHDC4,SMOC1,FER,PAK5,SETDB2,PRLR,NFATC2,DST,FAT3,CACNB2,NTNG1,MED15,MTPN,MTMR2,LRP12,MAPRE2,FGF10,KIF13A,NOS1AP,PDE4DIP,MPDZ,YLPM1,TRPM3,VCL,SEZ6L,XKR6,RAPGEF2,CDH7,IFT81,SHANK2,ZMYND11,INSR,AKAP13,IMMP2L,SYNJ1,CHCHD6,UNC5D,KDM1B,GABRG2,ARFGEF1,FAT1,PATJ,ENPP1,ULK2,UNC13C,ZNF462,NRXN3,RIMS2,RHPN2,PPM1F,KCNQ3,PGM5,GFRA1,BMPR1B,NSG1,KCND2,SEMA5A,ATP8A2,GOLGA8T,PYGO1,ARHGAP24,MAP4K4,CTNNA2,APC,APLF,PARD3B,DNER,ABCA5,ADGRV1,RELN,AKAP11,VPS13C,AUTS2,ROR1,MTMR3,BMP2K,SNAI2,CYFIP2,ADAMTSL1,TNR,PTPN13,SNAP91,OCA2,CELF4,AKT3,CRB1,VAV3,BCAS3,FCHO2,PRKCA,KMT2C,USP8,USH1C,DNAH5,CNTN6,KCNS3,FMN2,ERC1,APP,PDLIM5,RPS6KA3,BTAF1,DNAJC13,NYAP2,VPS13B,ABCA13,ASB2,EYA2,ADGRB1,CCDC88A,RAP1GDS1,SPOCK1
GO:0065009	regulation of molecular function	0.022765501809730165	SLMAP,ERBB4,PAK1,AMBRA1,NETO2,NRP1,RPS6KA5,TAOK3,ABL2,PRKCB,ARHGEF7,UTRN,SLC8A1,CDK12,KCNC1,TBC1D5,USP14,CNKSR3,SLC8A3,MGAT5,SLCO3A1,FGD4,RGL1,SIPA1L2,CCNG2,EVI5,TAFA4,PBX1,ARHGAP28,DISC1,HECW2,RALGPS1,ARHGAP42,KSR1,ASAP1,SGSM1,FYN,PRMT8,DLGAP1,RGS7,GRIN2B,ANTXR1,SRGAP2,SPRED1,FGF12,CACNG2,GARNL3,TPM1,NOTCH2,ESR1,PDE4D,PTPRT,HIPK3,TMEM225,GLI3,NRXN1,NTRK3,BCL2L13,PRRC1,RAPGEF4,NEK10,HMGA2,ITGA4,ABCC9,GSG1L,ROCK1,CACNA1C,PRKN,SIAH2,LATS2,DOCK9,CDC14B,HDAC4,MYO9A,NTRK2,MAPK1,PRKG1,BIRC6,DGKI,EGFR,BCR,MAGI2,AKAP6,LARGE1,PLCL1,TBC1D1,PDP2,GNAQ,DOCK10,GRM5,ARAP2,LDB2,NLGN1,ABL1,EBF2,ERBIN,MBTPS2,FER,RYR2,PRLR,CACNB2,MTPN,GNG7,MAPRE2,FGF10,NOS1AP,ZDHHC21,RAPGEF2,RAPGEF5,INSR,WDR41,ARFGEF1,ENPP1,ZNF462,PPM1F,EGLN3,DLGAP2,KITLG,ARHGAP24,MAP4K4,CNIH3,DOCK3,APC,ADGRV1,RELN,ANXA4,TBC1D9,ROR1,CYFIP2,NDFIP1,VAV3,BCAS3,ERC1,APP,RPS6KA3,BTAF1,MAPK8IP1,CCDC88A,RAP1GDS1,SPOCK1
GO:0048738	cardiac muscle tissue development	0.023580419252188973	ERBB4,ALPK3,SLC8A1,ADAMTS9,NEBL,TPM1,NOTCH2,SGCG,FHOD3,PRKG1,AKAP6,LARGE1,MED1,ABL1,JARID2,LRP2,RYR2,SOX6,SGCD,AKAP13,PDLIM5,SMAD5,ALPK2,ASB2
GO:0090066	regulation of anatomical structure size	0.02359707085899 02	KCNMA1,MACF1,KIRREL1,MYO3B,NRP1,EPS8,SLC8A1,PRR16,SEMA3A,CDC42EP3,ARHGAP28,DISC1,FMN1,ARHGAP42,SVIL,TMOD2,FCHSD2,DOCK4,FHOD3,ROCK1,BBS2,ARHGAP32,MYO3A,PRKG1,DSCAM,DIP2B,SLC12A1,ABL1,SEMA6D,FER,MTPN,ZDHHC21,ARFGEF1,ULK2,SEM

			A5A,CYFIP2,TNR,AKT3,VAV3,USH1C,RAP1GDS1
GO:0032535	regulation of cellular component size	0.024334948947814336	*KCNMA1,MACF1,KIRREL1,MYO3B,NRP1,EPS8,PRR16,SEMA3A,CDC42EP3,ARHGAP28,DISC1,FMN1,SVIL,TMOD2,FCHSD2,FHOD3,ARHGAP32,MYO3A,DSCAM,DIP2B,SLC12A1,ABL1,SEMA6D,FER,MTPN,ARFGEF1,ULK2,SEMA5A,CYFIP2,TNR,AKT3,VAV3,USH1C*
GO:0070848	response to growth factor	0.024547731939276916	*FSTL1,ERBB4,EPN2,NRP1,NFIA,PRKCB,KCNC1,LDLRAD4,SNX25,KIF16B,ITGA8,FYN,CREBBP,GRIA1,SPRED1,CRIM1,NOTCH2,JCAD,NRXN1,NTRK3,FBN1,WWOX,ZDHHC17,ROCK1,COL4A2,GRB10,LATS2,NTRK2,MAPK1,SDCBP,BPTF,SOX5,NLK,EGFR,MAGI2,MED1,ABL1,LRP2,FER,VSTM2A,FGF10,SOX6,VCAM1,RAPGEF2,INSR,ZFHX3,GFRA1,BMPR1B,SNAI2,CYFIP2,USP8,SMAD5*
GO:0051966	regulation of synaptic transmission, glutamatergic	0.025727829936113365	*GRIK2,DISC1,GRIN2B,CACNG2,NRXN1,SYT1,GRIK1,DGKI,GRM5,NLGN1,GRIK3,RELN,TNR*
GO:0097061	dendritic spine organization	0.025727829936113365	*CTNND2,STAU2,TANC1,FYN,GRIN2B,DNM3,PPFIA2,DOCK10,NLGN1,MTMR2,INSR,RELN,PDLIM5*
GO:0150115	cell-substrate junction organization	0.025891495247336777	*MACF1,NRP1,ARHGEF7,CORO2B,FMN1,PEAK1,WDPCP,ROCK1,BCR,ABL1,DST,MAPRE2,VCL,PPM1F,MAP4K4*
GO:0006936	muscle contraction	0.025949605813370358	*SLMAP,KCNMA1,APBB2,UTRN,SLC8A1,SLC8A3,ASB3,ARHGAP42,FGF12,TPM1,TMOD2,PDE4D,DOCK4,ABCC9,ROCK1,CACNA1C,ARHGEF11,BBS2,PPP1R12B,HDAC4,PRKG1,LARGE1,RYR2,CACNB2,NOS1AP,SGCD,ZDHHC21,ATP8A2,DTNA,SMAD5,RAP1GDS1*
GO:0042692	muscle cell differentiation	0.026031009345831597	*ALPK3,SLC8A1,AFG3L2,MYEF2,TANC1,NEBL,ITGA8,TPM1,TMOD2,LAMA1,PIAS1,FHOD3,DOCK1,RORA,HDAC4,ADGRB3,LAMB1,PRKG1,AKAP6,LARGE1,JAM2,NFATC2,MTPN,FGF10,SOX6,SGCD,AKAP13,PGM5,DNER,MRTFB,PDLIM5,ALPK2,ASB2,ADGRB1*
GO:0022604	regulation of cell morphogenesis	0.02880532538557497	*MACF1,EPS8,SLC23A2,ARHGEF7,STAU2,LIMD1,CDC42EP3,FGD4,MYO10,FAM171A1,ATP10A,FYN,SH3KBP1,TPM1,RIMS1,PARVB,WASF3,WDPCP,SYT1,PRKN,PTPRD,MYO9A,NTNG1,RIMS2,RELN*
GO:0099505	regulation of presynaptic membrane potential	0.03375165609955328	*KCNC1,GRIK2,GRIK4,GABRB1,GRIN2B,GRIA1,GRIK3,GRIA4*
GO:0030031	cell projection	0.03408053299448471	*SYNE2,MYO3B,NRP1,CDH13,EPS8,ARHGEF7,STAU2,RFX3,DCLK1,CEP120,CDC42EP3,FGD4,MYO10,TTC21B,DISC1,ASAP1,SRGAP2,PCDH1*

	assembly		*5,SRGAP2C,PARVB,NRXN1,WASF3,HYDIN,WDPCP,ROCK1,CFAP70,DNM3,BBS2,CDC14B,BBS9,MYO3A,SDCBP,NLGN1,ABL1,FER,VCL,RAPGEF2,IFT81,ARHGAP24,APC,AUTS2,CYFIP2,VAV3,DNAH5,CCDC88A*
GO:0031032	actomyosin structure organization	0.03430513233978309	*PAK1,FRMD3,NRP1,FRMD5,CDC42BPA,CORO2B,NEBL,FAM171A1,PHACTR1,ARHGAP28,TPM1,TMOD2,TJP1,FHOD3,ROCK1,TRPM7,ABL1,AKAP13,RHPN2,PPM1F,PGM5,CCDC88A*
GO:0021537	telencephalon development	0.03687524943580345	*ERBB4,SYNE2,KCNC1,SEMA3A,CEP120,PHACTR1,DISC1,SLC1A2,SRGAP2,GRIA1,SRGAP2C,ZSWIM6,GLI3,HERC1,BBS2,FOXP2,NTRK2,PLCB1,LAMB1,NFIB,EGFR,LARGE1,SCN2A,NRG3,RELN,TNR,DNAH5*
GO:0006836	neurotransmitter transport	0.03708859196951116	*SLC6A11,PRKCB,DYSF,SV2B,SLC1A2,CADPS,ERC2,RIMS1,NRXN1,SYN2,SYT1,PRKN,PTPRN2,DGKI,PPFIA2,NLGN1,SYNJ1,UNC13C,NRXN3,RIMS2,SV2C,SLC6A3*
GO:0007160	cell-matrix adhesion	0.03928512400305017	*MACF1,NRP1,CDH13,ARHGEF7,UTRN,ADAMTS9,CORO2B,ITGA8,DISC1,FMN1,PEAK1,WDPCP,ITGA4,ROCK1,TRPM7,PPFIA2,BCR,ABL1,VCL,VCAM1,ITGA9,PPM1F,FREM1,MAP4K4*
GO:0071495	cellular response to endogenous stimulus	0.03975183505880648	*FSTL1,ERBB4,PAK1,EPN2,NRP1,NFIA,PRKCB,SLC8A1,HTR2C,LDLRAD4,SNX25,MBD5,KIF16B,ITGA8,GABRB1,FYN,CREBBP,SLC1A2,SLIT3,GRIA1,SPRED1,MYO5A,CRIM1,NOTCH2,ESR1,PDE4D,GNG2,JCAD,ITPR2,NRXN1,NTRK3,FBN1,WWOX,ARID1B,ZDHHC17,ITGA4,ROCK1,UBR1,RXRG,PRKAA2,COL4A2,BBS2,GRB10,PRKN,LATS2,NTRK2,PLCB1,MAPK1,SDCBP,BPTF,SOX5,NLK,EGFR,MAGI2,AKAP6,LARGE1,GNAQ,GRM5,MED1,CPEB4,PID1,ABL1,NCOR1,LRP2,FER,RYR2,PRLR,STXBP4,VSTM2A,FGF10,SOX6,ZNF106,VCAM1,RAPGEF2,INSR,KLF15,DENND4C,GABRG2,ENPP1,GFRA1,BMPR1B,NSG1,SNAI2,CYFIP2,BCAS3,USP8,APP,SMAD5,RAP1GDS1*
GO:0016049	cell growth	0.04064594541517787	*MACF1,NRP1,TEAD1,SLC23A2,RERG,SEMA3A,ALCAM,DCLK1,SLC39A12,DISC1,SLIT3,RIMS1,ITGA4,ARHGEF11,SYT1,ARHGAP32,PRKN,SDCBP,PRKG1,DSCAM,DIP2B,EGFR,AKAP6,NRG3,ABL1,SEMA6D,PAK5,MTPN,VCL,ENPP1,ULK2,RIMS2,SEMA5A,AUTS2,CYFIP2,TNR,APP,PDLIM5,RPS6KA3,SPOCK1*
GO:0040017	positive regulation of locomotion	0.04249313524049236	*PAK1,SYNE2,NRP1,CDH13,ABL2,ARHGEF7,SEMA3A,MGAT5,NIPBL,AGO2,SRGAP2C,TJP1,JCAD,DOCK4,NTRK3,ITGA4,DOCK1,ARHGAP32,MAPK1,LAMB1,SDCBP,DSCAM,EGFR,ATP8A1,ABL1,JAM2,SEMA6D,FER,MAPRE2,FGF10,RAPGEF2,INSR,PPM1F,SEMA5A,KITLG,MAP4K4,APC,RELN,SNAI2,AKT3,IL1R1,BCAS3,PRKCA,APP*
GO:0120031	plasma membrane bounded cell projection assembly	0.044330275348464274	*SYNE2,MYO3B,NRP1,CDH13,EPS8,ARHGEF7,STAU2,RFX3,DCLK1,CEP120,CDC42EP3,FGD4,MYO10,TTC21B,DISC1,ASAP1,SRGAP2,PCDH15,SRGAP2C,PARVB,NRXN1,WASF3,HYDIN,WDPCP,ROCK1,CFAP70,DNM3,BBS2,CDC14B,BBS9,MYO3A,SDCBP,NLGN1,ABL1,FER,VCL,RAPGEF2,IFT81,ARHGAP24,APC,AUTS2,VAV3,DNAH*

			5,CCDC88A
GO:2000147	positive regulation of cell motility	0.04865537477210208	PAK1,SYNE2,NRP1,CDH13,ABL2,ARHGEF7,SEMA3A,MGAT5,NIPBL,AGO2,SRGAP2C,TJP1,JCAD,DOCK4,NTRK3,ITGA4,DOCK1,ARHGAP32,MAPK1,LAMB1,SDCBP,EGFR,ATP8A1,ABL1,JAM2,SEMA6D,FER,MAPRE2,FGF10,RAPGEF2,INSR,PPM1F,SEMA5A,KITLG,MAP4K4,APC,RELN,SNAI2,AKT3,IL1R1,BCAS3,PRKCA,APP
GO:0007044	cell-substrate junction assembly	0.04975114634460492	MACF1,NRP1,ARHGEF7,CORO2B,FMN1,PEAK1,WDPCP,ROCK1,BCR,ABL1,DST,VCL,PPM1F,MAP4K4

Table S8. GO associations with biological processes associated with 158 rDNA-contacting genes that are associated with lincRNAs in K562-diff. cells. The search was performed using g:Profiler (https://biit.cs.ut.ee/gprofiler/gost). Related to Figure 2C.

GO.ID	Description	padj	Genes
GO:0048699	generation of neurons	6.880667464910143e-11	HCN1,AGBL4,DSCAML1,CECR2,NEDD4,THRB,SH3GL3,ASTN2,BRINP3,TANC2,DPYSL5,PAK3,ZNF536,IL1RAPL1,PLXNA4,TAFA1,LYN,ELAVL4,TIAM2,NTF3,TBX20,FLRT2,RBFOX2,BRINP2,TMEM108,GRM7,PTPRM,KIRREL3,PCP4,STXBP1,ANK3,SH3GL2,DDR2,DCC,HMCN2,FEZ2,SLIT2,MINAR1,LHX9
GO:0007399	nervous system development	7.166248343204405e-11	TACC2,HCN1,AGBL4,DSCAML1,CECR2,NEDD4,THRB,SH3GL3,ASTN2,BRINP3,TANC2,DPYSL5,ATXN3,PAK3,GABRA2,ZNF536,GRIN2A,SHC3,IL1RAPL1,FGF14,PLXNA4,IGSF21,TAFA1,KDM7A,LYN,ELAVL4,TIAM2,LINGO2,SLC24A4,MTHFD1L,NTF3,TBX20,FLRT2,RBFOX2,BRINP2,TMEM108,GABRB3,GRM7,PTPRM,KIRREL3,PCP4,STXBP1,ANK3,SH3GL2,DDR2,DCC,HMCN2,FEZ2,SLIT2,MINAR1,LHX9
GO:0048731	system development	8.794561953895423e-11	CD44,TACC2,DMRT1,HCN1,AGBL4,DSCAML1,CECR2,NEDD4,THRB,IL6R,PKHD1,ZFPM2,PDGFD,SH3GL3,ASTN2,BRINP3,TANC2,DPYSL5,ATXN3,PAK3,GABRA2,ZNF536,GRIN2A,SHC3,IL1RAPL1,BMPER,FGF14,ANGPT1,RPS6KA2,PLXNA4,IGSF21,SMOC2,PPARA,TAFA1,KDM7A,LYN,ELAVL4,TIAM2,LINGO2,RASSF2,SLC24A4,MTHFD1L,NTF3,TBX20,FLRT2,GAS2,RBFOX2,BRINP2,TMEM108,GABRB3,GRM7,PTPRM,KIRREL3,PCP4,STXBP1,ANK3,SH3GL2,DDR2,DCC,HMCN2,FEZ2,SLIT2,MINAR1,KLHL3,LHX9
GO:0007275	multicellular organism development	2.6113594131417265e-10	CD44,TACC2,DMRT1,HCN1,AGBL4,DSCAML1,CECR2,NEDD4,THRB,IL6R,PKHD1,ZFPM2,PDGFD,SH3GL3,ASTN2,BRINP3,TANC2,DPYSL5,ATXN3,PAK3,GABRA2,SPECC1,ZNF536,GRIN2A,SHC3,IL1RAPL1,BMPER,FGF14,ANGPT1,RPS6KA2,PLXNA4,IGSF21,SMOC2,PPARA,TAFA1,KDM7A,LYN,ELAVL4,MYSM1,TIAM2,LINGO2,RASSF2,

			SLC24A4,MTHFD1L,NTF3,RNF111,CFTR,TBX20,FLRT2,GAS2,RBFOX2,BRINP2,TMEM108,GABRB3,GRM7,PTPRM,NEDD9,KIRREL3,PCP4,STXBP1,ANK3,SH3GL2,DDR2,DCC,HMCN2,FEZ2,SLIT2,MINAR1,KLHL3,LHX9
GO:0030182	neuron differentiation	3.3358106204730936e-10	*HCN1,AGBL4,DSCAML1,CECR2,NEDD4,THRB,SH3GL3,BRINP3,TANC2,DPYSL5,PAK3,ZNF536,IL1RAPL1,PLXNA4,LYN,ELAVL4,TIAM2,NTF3,TBX20,FLRT2,RBFOX2,BRINP2,TMEM108,GRM7,PTPRM,KIRREL3,PCP4,STXBP1,ANK3,SH3GL2,DDR2,DCC,HMCN2,FEZ2,SLIT2,MINAR1,LHX9*
GO:0022008	neurogenesis	1.2176342862607e-9	*HCN1,AGBL4,DSCAML1,CECR2,NEDD4,THRB,SH3GL3,ASTN2,BRINP3,TANC2,DPYSL5,PAK3,ZNF536,GRIN2A,IL1RAPL1,PLXNA4,TAFA1,LYN,ELAVL4,TIAM2,NTF3,TBX20,FLRT2,RBFOX2,BRINP2,TMEM108,GRM7,PTPRM,KIRREL3,PCP4,STXBP1,ANK3,SH3GL2,DDR2,DCC,HMCN2,FEZ2,SLIT2,MINAR1,LHX9*
GO:0032990	cell part morphogenesis	4.483568904568718e-9	*CD44,DSCAML1,NEDD4,TANC2,DPYSL5,PAK3,IL1RAPL1,PLXNA4,NUBPL,ELAVL4,TIAM2,NTF3,FLRT2,RBFOX2,TMEM108,PTPRM,KIRREL3,STXBP1,ANK3,SH3GL2,DCC,HMCN2,FEZ2,SLIT2,LHX9*
GO:0000902	cell morphogenesis	8.017875646384687e-9	*CD44,DMRT1,DSCAML1,NEDD4,PKHD1,CDH8,CDH18,TANC2,DPYSL5,PAK3,IL1RAPL1,PLXNA4,ELAVL4,TIAM2,NTF3,FLRT2,GAS2,RBFOX2,TMEM108,PTPRM,KIRREL3,STXBP1,ANK3,SH3GL2,DCC,HMCN2,FEZ2,SLIT2,LHX9*
GO:0120039	plasma membrane bounded cell projection morphogenesis	1.3889521241804676e-8	*CD44,DSCAML1,NEDD4,TANC2,DPYSL5,PAK3,IL1RAPL1,PLXNA4,ELAVL4,TIAM2,NTF3,FLRT2,RBFOX2,TMEM108,PTPRM,KIRREL3,STXBP1,ANK3,SH3GL2,DCC,HMCN2,FEZ2,SLIT2,LHX9*
GO:0048856	anatomical structure development	1.4006849545927393e-8	*CD44,TACC2,DMRT1,HCN1,AGBL4,DSCAML1,CECR2,NEDD4,THRB,IL6R,PKHD1,CDH8,ZFPM2,CDH18,DEUP1,PDGFD,SH3GL3,ASTN2,BRINP3,TANC2,DPYSL5,ATXN3,PAK3,GABRA2,SPECC1,PDE3A,ZNF536,GRIN2A,SHC3,IL1RAPL1,KRT6A,BMPER,FGF14,ANGPT1,RPS6KA2,PLXNA4,IGSF21,SMOC2,PPARA,TAFA1,KDM7A,LYN,NUBPL,ELAVL4,MYSM1,TIAM2,LINGO2,RASSF2,SLC24A4,MTHFD1L,NTF3,RNF111,CFTR,TBX20,FLRT2,GAS2,RBFOX2,BRINP2,TMEM108,GABRB3,GRM7,RASAL2,PTPRM,NEDD9,KIRREL3,PCP4,STXBP1,ANK3,SH3GL2,DDR2,DCC,HMCN2,FEZ2,SLIT2,MINAR1,KLHL3,LHX9*
GO:0048858	cell projection morphogenesis	1.628928640202945e-8	*CD44,DSCAML1,NEDD4,TANC2,DPYSL5,PAK3,IL1RAPL1,PLXNA4,ELAVL4,TIAM2,NTF3,FLRT2,RBFOX2,TMEM108,PTPRM,KIRREL3,STXBP1,ANK3,SH3GL2,DCC,HMCN2,FEZ2,SLIT2,LHX9*

GO:0048666	neuron development	2.1761878538727378e-8	*HCN1,AGBL4,DSCAML1,CECR2,NEDD4,THRB,TANC2,DPYSL5,PAK3,IL1RAPL1,PLXNA4,LYN,ELAVL4,TIAM2,NTF3,FLRT2,RBFOX2,TMEM108,GRM7,PTPRM,KIRREL3,STXBP1,ANK3,SH3GL2,DDR2,DCC,HMCN2,FEZ2,SLIT2,MINAR1,LHX9*
GO:0048812	neuron projection morphogenesis	5.684327755749413e-8	*DSCAML1,NEDD4,TANC2,DPYSL5,PAK3,IL1RAPL1,PLXNA4,ELAVL4,TIAM2,NTF3,FLRT2,RBFOX2,TMEM108,PTPRM,KIRREL3,STXBP1,ANK3,SH3GL2,DCC,HMCN2,FEZ2,SLIT2,LHX9*
GO:0032502	developmental process	5.777700108079656e-8	*CD44,TACC2,DMRT1,HCN1,LRMDA,AGBL4,DSCAML1,CECR2,NEDD4,THRB,IL6R,PKHD1,CDH8,ZFPM2,CDH18,DEUP1,PDGFD,SH3GL3,ASTN2,BRINP3,TANC2,DPYSL5,ATXN3,PAK3,GABRA2,SPECC1,PDE3A,ZNF536,MORC1,GRIN2A,SHC3,IL1RAPL1,KRT6A,BMPER,FGF14,ANGPT1,RPS6KA2,PLXNA4,IGSF21,TBATA,SMOC2,PPARA,TAFA1,KDM7A,LYN,NUBPL,ELAVL4,MYSM1,TIAM2,LINGO2,RASSF2,SLC24A4,MTHFD1L,NTF3,RNF111,CFTR,TBX20,FLRT2,GAS2,RBFOX2,BRINP2,TMEM108,GABRB3,GRM7,RASAL2,PTPRM,NEDD9,KIRREL3,PCP4,STXBP1,ANK3,SH3GL2,DDR2,DCC,HMCN2,FEZ2,SLIT2,MINAR1,KLHL3,LHX9*
GO:0032989	cellular component morphogenesis	9.911093769042061e-8	*CD44,DSCAML1,NEDD4,TANC2,DPYSL5,PAK3,IL1RAPL1,PLXNA4,NUBPL,ELAVL4,TIAM2,NTF3,FLRT2,RBFOX2,TMEM108,PTPRM,KIRREL3,STXBP1,ANK3,SH3GL2,DCC,HMCN2,FEZ2,SLIT2,LHX9*
GO:0031175	neuron projection development	9.971556895982489e-8	*DSCAML1,CECR2,NEDD4,TANC2,DPYSL5,PAK3,IL1RAPL1,PLXNA4,LYN,ELAVL4,TIAM2,NTF3,FLRT2,RBFOX2,TMEM108,GRM7,PTPRM,KIRREL3,STXBP1,ANK3,SH3GL2,DDR2,DCC,HMCN2,FEZ2,SLIT2,MINAR1,LHX9*
GO:0007154	cell communication	3.5069269057350927e-7	*CD44,RPTOR,DMRT1,MDFIC,HCN1,PTPRE,NEDD4,SH3GLB1,THRB,IL6R,PKHD1,CDH8,RSU1,AKAP10,PDGFD,HCRTR1,SH3GL3,DPYSL5,ATXN3,PAK3,GABRA2,PDE3A,ZNF536,SLC24A2,GRIN2A,PRKAG2,MCTP2,SHC3,IL1RAPL1,CTNNA3,BMPER,PDE1A,FGF14,ANGPT1,RPH3A,RPS6KA2,MVB12B,PLXNA4,SMOC2,XPR1,PRKCE,GRM3,OR4F15,PPARA,PIAS2,TAFA1,LYN,SH3BP5,TIAM2,TSHZ3,RASSF2,SLC24A4,NTF3,RNF111,CFTR,TBX20,FLRT2,GAS2,PDE10A,RBFOX2,ACER2,TMEM108,GABRB3,GRM7,RASAL2,PTPRM,NEDD9,MYRIP,PCP4,STXBP1,ADCY2,ANK3,SH3GL2,DDR2,DCC,FEZ2,SLIT2,MINAR1,MCTP1*
GO:0009653	anatomical structure morphogenesis	7.169724380881554e-7	*CD44,DMRT1,HCN1,DSCAML1,CECR2,NEDD4,THRB,PKHD1,CDH8,ZFPM2,CDH18,ASTN2,TANC2,DPYSL5,PAK3,IL1RAPL1,KRT6A,BMPER,ANGPT1,PLXNA4,SMOC2,PPARA,NUBPL,ELAVL4,TIAM2,SLC24A4,MTHFD1L,NTF3,CFTR,TBX20,FLRT2,GAS2,RBFOX2,TMEM108,PTPRM,KIRREL3,STXBP*

			1,ANK3,SH3GL2,DCC,HMCN2,FEZ2,SLIT2,MINAR1,KLHL3,LHX9
GO:0032501	multicellular organismal process	0.0000014789783542769327	*CD44,RPTOR,TACC2,DMRT1,HCN1,AGBL4,DSCAML1,CECR2,SLC16A7,NEDD4,THRB,IL6R,PKHD1,ZFPM2,PDGFD,HCRTR1,SH3GL3,ASTN2,BRINP3,TANC2,DPYSL5,ATXN3,PAK3,GABRA2,SPECC1,PDE3A,ZNF536,MORC1,SLC24A2,GRIN2A,SHC3,IL1RAPL1,CTNNA3,KRT6A,BMPER,FGF14,ANGPT1,RPS6KA2,PLXNA4,IGSF21,TBATA,SMOC2,PRKCE,OR4F15,PPARA,F13A1,TAFA1,KDM7A,LYN,ELAVL4,MYSM1,TIAM2,TSHZ3,LINGO2,RASSF2,SLC24A4,MTHFD1L,NTF3,RNF111,CFTR,TBX20,FLRT2,GAS2,RBFOX2,BRINP2,TMEM108,GABRB3,GRM7,RASAL2,PTPRM,NEDD9,KIRREL3,PCP4,STXBP1,ANK3,SNTB1,SH3GL2,DDR2,DCC,HMCN2,FEZ2,SLIT2,MINAR1,FBXO32,KLHL3,LHX9*
GO:0023052	signaling	0.0000039134625074112 25	*CD44,RPTOR,DMRT1,MDFIC,HCN1,PTPRE,NEDD4,THRB,IL6R,PKHD1,CDH8,RSU1,AKAP10,PDGFD,HCRTR1,SH3GL3,DPYSL5,ATXN3,PAK3,GABRA2,PDE3A,ZNF536,SLC24A2,GRIN2A,PRKAG2,MCTP2,SHC3,IL1RAPL1,BMPER,PDE1A,FGF14,ANGPT1,RPH3A,RPS6KA2,MVB12B,PLXNA4,SMOC2,PRKCE,GRM3,OR4F15,PPARA,PIAS2,TAFA1,LYN,SH3BP5,TIAM2,TSHZ3,RASSF2,SLC24A4,NTF3,RNF111,CFTR,TBX20,FLRT2,GAS2,PDE10A,RBFOX2,ACER2,TMEM108,GABRB3,GRM7,RASAL2,PTPRM,NEDD9,MYRIP,PCP4,STXBP1,ADCY2,ANK3,SH3GL2,DDR2,DCC,FEZ2,SLIT2,MINAR1,MCTP1*
GO:0048667	cell morphogenesis involved in neuron differentiation	0.0000149872544989502 16	*DSCAML1,NEDD4,TANC2,DPYSL5,PAK3,IL1RAPL1,PLXNA4,ELAVL4,TIAM2,FLRT2,RBFOX2,PTPRM,STXBP1,ANK3,DCC,HMCN2,FEZ2,SLIT2,LHX9*
GO:0030030	cell projection organization	0.0000153735741716544 05	*CD44,DSCAML1,CECR2,NEDD4,PKHD1,DEUP1,TANC2,DPYSL5,PAK3,IL1RAPL1,PLXNA4,LYN,ELAVL4,TIAM2,NTF3,FLRT2,RBFOX2,TMEM108,GRM7,PTPRM,NEDD9,KIRREL3,STXBP1,ANK3,SH3GL2,DDR2,DCC,HMCN2,FEZ2,SLIT2,MINAR1,LHX9*
GO:0120036	plasma membrane bounded cell projection organization	0.00003194571511988 5	*CD44,DSCAML1,CECR2,NEDD4,PKHD1,TANC2,DPYSL5,PAK3,IL1RAPL1,PLXNA4,LYN,ELAVL4,TIAM2,NTF3,FLRT2,RBFOX2,TMEM108,GRM7,PTPRM,NEDD9,KIRREL3,STXBP1,ANK3,SH3GL2,DDR2,DCC,HMCN2,FEZ2,SLIT2,MINAR1,LHX9*
GO:0050808	synapse organization	0.0000390231200371906 46	*NEDD4,CDH8,TANC2,PPFIBP1,PAK3,GABRA2,IL1RAPL1,PLXNA4,IGSF21,LINGO2,FLRT2,TMEM108,GABRB3,NEDD9,KIRREL3,ANK3,HMCN2*
GO:005	cellular	0.00003944616500834	*CD44,RPTOR,DMRT1,MDFIC,HCN1,TOP3A,PTPRE,ARPP21,NEDD4,SH3GLB1,THRB,*

1716	response to stimulus	126	*IL6R,PKHD1,RSU1,AKAP10,PDGFD,HCRTR1,SH3GL3,BRINP3,DPYSL5,ATXN3,PAK3,SEL1L2,GABRA2,PDE3A,ZNF536,SLC24A2,GRIN2A,PRKAG2,MCTP2,SHC3,IL1RAPL1,BMPER,PDE1A,FGF14,ANGPT1,RPS6KA2,MVB12B,PLXNA4,SMOC2,XPR1,PRKCE,GRM3,OR4F15,PPARA,PIAS2,TAFA1,LYN,ELAVL4,SH3BP5,TIAM2,RASSF2,SLC24A4,NTF3,RNF111,CFTR,TBX20,FLRT2,GAS2,PDE10A,RBFOX2,BRINP2,ACER2,TMEM108,GABRB3,GRM7,BACH1,RASAL2,PTPRM,NEDD9,PCP4,STXBP1,ADCY2,ANK3,SH3GL2,DDR2,FEZ2,SLIT2,MINAR1,FBXO32,MCTP1*
GO:0030154	cell differentiation	0.000048687234603870954	*DMRT1,HCN1,LRMDA,AGBL4,DSCAML1,CECR2,NEDD4,THRB,IL6R,PKHD1,ZFPM2,DEUP1,SH3GL3,ASTN2,BRINP3,TANC2,DPYSL5,PAK3,PDE3A,ZNF536,MORC1,GRIN2A,IL1RAPL1,KRT6A,ANGPT1,RPS6KA2,PLXNA4,TBATA,PPARA,TAFA1,LYN,ELAVL4,MYSM1,TIAM2,RASSF2,NTF3,CFTR,TBX20,FLRT2,RBFOX2,BRINP2,TMEM108,GRM7,PTPRM,NEDD9,KIRREL3,PCP4,STXBP1,ANK3,SH3GL2,DDR2,DCC,HMCN2,FEZ2,SLIT2,MINAR1,LHX9*
GO:0048869	cellular developmental process	0.00004910517599828307	*DMRT1,HCN1,LRMDA,AGBL4,DSCAML1,CECR2,NEDD4,THRB,IL6R,PKHD1,ZFPM2,DEUP1,SH3GL3,ASTN2,BRINP3,TANC2,DPYSL5,PAK3,PDE3A,ZNF536,MORC1,GRIN2A,IL1RAPL1,KRT6A,ANGPT1,RPS6KA2,PLXNA4,TBATA,PPARA,TAFA1,LYN,ELAVL4,MYSM1,TIAM2,RASSF2,NTF3,CFTR,TBX20,FLRT2,RBFOX2,BRINP2,TMEM108,GRM7,PTPRM,NEDD9,KIRREL3,PCP4,STXBP1,ANK3,SH3GL2,DDR2,DCC,HMCN2,FEZ2,SLIT2,MINAR1,LHX9*
GO:0048468	cell development	0.00006166861586459082	*DMRT1,HCN1,AGBL4,DSCAML1,CECR2,NEDD4,THRB,IL6R,PKHD1,TANC2,DPYSL5,PAK3,PDE3A,IL1RAPL1,ANGPT1,RPS6KA2,PLXNA4,PPARA,LYN,ELAVL4,MYSM1,TIAM2,RASSF2,NTF3,CFTR,TBX20,FLRT2,RBFOX2,TMEM108,GRM7,PTPRM,NEDD9,KIRREL3,STXBP1,ANK3,SH3GL2,DDR2,DCC,HMCN2,FEZ2,SLIT2,MINAR1,LHX9*
GO:0050896	response to stimulus	0.00007523312737468003	*CD44,RPTOR,DMRT1,MDFIC,HCN1,TOP3A,PTPRE,ARPP21,AGBL4,TRIM43B,NEDD4,SH3GLB1,THRB,IL6R,PKHD1,CDH8,RSU1,AKAP10,PDGFD,HCRTR1,SH3GL3,BRINP3,DPYSL5,ATXN3,PAK3,PSIP1,SEL1L2,GABRA2,PDE3A,ZNF536,MORC1,SLC24A2,GRIN2A,PRKAG2,MCTP2,SHC3,IL1RAPL1,KRT6A,BMPER,PDE1A,FGF14,ANGPT1,RPS6KA2,MVB12B,PLXNA4,SMOC2,XPR1,PRKCE,GRM3,OR4F15,PPARA,F13A1,PIAS2,TAFA1,PRG4,LYN,ELAVL4,SH3BP5,TIAM2,RASSF2,SLC24A4,NTF3,RNF111,CFTR,TBX20,FLRT2,GAS2,PDE10A,RBFOX2,BRINP2,ACER2,TMEM108,GABRB3,GRM7,BACH1,RASAL2,PTPRM,NEDD9,PCP4,*

			STXBP1,ADCY2,ANK3,SH3GL2,DDR2,HMCN2,FEZ2,SLIT2,MINAR1,FBXO32,BCKDHB,MCTP1
GO:0008283	cell population proliferation	0.00007962494527429469	*CD44,RPTOR,TACC2,DMRT1,AGBL4,DSCAML1,CECR2,NEDD4,IL6R,PKHD1,ZFPM2,DEUP1,PDGFD,TANC2,DPYSL5,PAK3,IL1RAPL1,KRT6A,BMPER,PDE1A,ANGPT1,RPS6KA2,PLXNA4,TAFA1,LYN,ELAVL4,TIAM2,NTF3,TBX20,FLRT2,RBFOX2,ACER2,TMEM108,GRM7,PTPRM,NEDD9,KIRREL3,STXBP1,ANK3,SH3GL2,DDR2,DCC,HMCN2,FEZ2,SLIT2,MINAR1,LHX9*
GO:0065008	regulation of biological quality	0.00010317928471441255	*RPTOR,HCN1,SLC12A8,NEDD4,SH3GLB1,THRB,BACE2,CDH8,TANC2,PAK3,GABRA2,PDE3A,SLC24A2,GRIN2A,MCTP2,IL1RAPL1,CTNNA3,ANGPT1,RPH3A,RPS6KA2,PLXNA4,PRKCE,PPARA,F13A1,ANO4,LYN,ELAVL4,TSHZ3,LINGO2,RASSF2,SLC24A4,CFTR,FLRT2,GAS2,TMEM108,GABRB3,NEDD9,MYRIP,STXBP1,ANK3,SH3GL2,DCC,SLIT2,MCTP1*
GO:0010646	regulation of cell communication	0.00016298194761245295	*CD44,RPTOR,DMRT1,MDFIC,HCN1,PTPRE,NEDD4,THRB,IL6R,PKHD1,PDGFD,HCRTR1,PAK3,PDE3A,ZNF536,SLC24A2,GRIN2A,MCTP2,BMPER,ANGPT1,MVB12B,SMOC2,PRKCE,GRM3,PPARA,PIAS2,TAFA1,LYN,TIAM2,TSHZ3,RASSF2,SLC24A4,NTF3,RNF111,CFTR,TBX20,GAS2,PDE10A,TMEM108,GRM7,MYRIP,STXBP1,ANK3,DDR2,DCC,SLIT2,MINAR1,MCTP1*
GO:0099537	trans-synaptic signaling	0.00021108678487973543	*HCN1,CDH8,HCRTR1,ATXN3,GABRA2,SLC24A2,GRIN2A,MCTP2,IL1RAPL1,RPH3A,RPS6KA2,PRKCE,GRM3,TSHZ3,TMEM108,GABRB3,GRM7,STXBP1,DCC,MCTP1*
GO:0050793	regulation of developmental process	0.00022253528318741118	*CD44,DMRT1,AGBL4,NEDD4,IL6R,PKHD1,ZFPM2,SH3GL3,BRINP3,TANC2,DPYSL5,PAK3,PDE3A,ZNF536,IL1RAPL1,BMPER,PLXNA4,SMOC2,PPARA,LYN,ELAVL4,MYSM1,TIAM2,LINGO2,RASSF2,NTF3,CFTR,TBX20,FLRT2,GAS2,RBFOX2,BRINP2,PTPRM,NEDD9,PCP4,DDR2,DCC,SLIT2,MINAR1*
GO:0099536	synaptic signaling	0.000356127908999918	*HCN1,CDH8,HCRTR1,ATXN3,GABRA2,SLC24A2,GRIN2A,MCTP2,IL1RAPL1,RPH3A,RPS6KA2,PRKCE,GRM3,TSHZ3,TMEM108,GABRB3,GRM7,STXBP1,DCC,MCTP1*
GO:0023051	regulation of signaling	0.00041652983468079097	*CD44,RPTOR,DMRT1,MDFIC,HCN1,PTPRE,NEDD4,THRB,IL6R,PKHD1,PDGFD,HCRTR1,PAK3,PDE3A,ZNF536,SLC24A2,GRIN2A,MCTP2,BMPER,ANGPT1,MVB12B,SMOC2,PRKCE,GRM3,PPARA,PIAS2,TAFA1,LYN,TIAM2,TSHZ3,RASSF2,SLC24A4,NTF3,RNF111,CFTR,TBX20,GAS2,PDE10A,TMEM108,GRM7,MYRIP,STXBP1,DDR2,DCC,SLIT2,MINAR1,MCTP1*
GO:0071495	cellular response to endogenous	0.0004675510837850311	*CD44,RPTOR,HCN1,PTPRE,NEDD4,THRB,PDGFD,HCRTR1,PDE3A,BMPER,SMOC2,PRKCE,PPARA,PIAS2,LYN,ELAVL4,NTF3,RNF111,CFTR,TBX20,FLRT2,RBFOX2,TME*

	stimulus		M108,GABRB3,SH3GL2,DDR2,SLIT2,FBXO32
GO:0007165	signal transduction	0.0007410529326035551	CD44,RPTOR,DMRT1,MDFIC,PTPRE,NEDD4,THRB,IL6R,PKHD1,RSU1,AKAP10,PDGFD,HCRTR1,SH3GL3,DPYSL5,PAK3,GABRA2,PDE3A,ZNF536,SLC24A2,GRIN2A,PRKAG2,MCTP2,SHC3,IL1RAPL1,BMPER,PDE1A,FGF14,ANGPT1,RPS6KA2,MVB12B,PLXNA4,SMOC2,PRKCE,GRM3,OR4F15,PPARA,PIAS2,TAFA1,LYN,SH3BP5,TIAM2,RASSF2,SLC24A4,NTF3,RNF111,TBX20,FLRT2,GAS2,PDE10A,RBFOX2,ACER2,TMEM108,GABRB3,GRM7,RASAL2,PTPRM,NEDD9,PCP4,ADCY2,ANK3,SH3GL2,DDR2,FEZ2,SLIT2,MINAR1,MCTP1
GO:0098916	anterograde trans-synaptic signaling	0.000868686068107308	HCN1,CDH8,HCRTR1,ATXN3,GABRA2,SLC24A2,GRIN2A,MCTP2,RPH3A,RPS6KA2,PRKCE,GRM3,TSHZ3,TMEM108,GABRB3,GRM7,STXBP1,DCC,MCTP1
GO:0007268	chemical synaptic transmission	0.000868686068107308	HCN1,CDH8,HCRTR1,ATXN3,GABRA2,SLC24A2,GRIN2A,MCTP2,RPH3A,RPS6KA2,PRKCE,GRM3,TSHZ3,TMEM108,GABRB3,GRM7,STXBP1,DCC,MCTP1
GO:0009719	response to endogenous stimulus	0.0009351759623752188	CD44,RPTOR,HCN1,PTPRE,NEDD4,THRB,PDGFD,HCRTR1,PDE3A,BMPER,SMOC2,PRKCE,PPARA,PIAS2,LYN,ELAVL4,SLC24A4,NTF3,RNF111,CFTR,TBX20,FLRT2,RBFOX2,TMEM108,GABRB3,SH3GL2,DDR2,SLIT2,FBXO32,BCKDHB
GO:0034330	cell junction organization	0.0010198021552909609	NEDD4,PKHD1,CDH8,CDH18,TANC2,PPFIBP1,PAK3,GABRA2,IL1RAPL1,PLXNA4,IGSF21,LINGO2,FLRT2,TMEM108,GABRB3,NEDD9,KIRREL3,ANK3,HMCN2
GO:0061564	axon development	0.0025356201718702343	DSCAML1,DPYSL5,PAK3,PLXNA4,TIAM2,FLRT2,GRM7,PTPRM,STXBP1,ANK3,DCC,HMCN2,FEZ2,SLIT2,LHX9
GO:0007155	cell adhesion	0.0029302147413265334	CD44,DSCAML1,PKHD1,CDH8,RSU1,CDH18,ASTN2,PPFIBP1,ATXN3,IL1RAPL1,CTNNA3,ANGPT1,PLXNA4,IGSF21,PRKCE,PPARA,LYN,FLRT2,ACER2,PTPRM,NEDD9,KIRREL3,STXBP1,ANK3,DDR2,DCC,HMCN2
GO:0007409	axonogenesis	0.0030081314063490344	DSCAML1,DPYSL5,PAK3,PLXNA4,TIAM2,FLRT2,PTPRM,STXBP1,ANK3,DCC,HMCN2,FEZ2,SLIT2,LHX9
GO:0007411	axon guidance	0.00794972624238169	DSCAML1,DPYSL5,PLXNA4,FLRT2,PTPRM,DCC,HMCN2,FEZ2,SLIT2,LHX9
GO:0097485	neuron projection guidance	0.00794972624238169	DSCAML1,DPYSL5,PLXNA4,FLRT2,PTPRM,DCC,HMCN2,FEZ2,SLIT2,LHX9
GO:0043116	negative regulation of vascular permeability	0.0113498757824683344	PDE3A,ANGPT1,SH3GL2,SLIT2
GO:000	behavior	0.0116725945905933 1	RPTOR,HCN1,THRB,HCRTR1,ATXN3,SPECC1,MORC1,SLC24A2,GRIN2A,PRKCE,PPA

7610		2	*RA,ELAVL4,SLC24A4,NTF3,NEDD9,KIRREL3*
GO:0035249	synaptic transmission, glutamatergic	0.013962552721893772	*HCN1,CDH8,GRIN2A,GRM3,TSHZ3,GRM7,STXBP1*
GO:0010975	regulation of neuron projection development	0.015936400707484706	*NEDD4,TANC2,DPYSL5,PAK3,IL1RAPL1,PLXNA4,LYN,ELAVL4,TIAM2,DDR2,DCC,SLIT2,MINAR1*
GO:0071363	cellular response to growth factor stimulus	0.019347785759461767	*CD44,NEDD4,PDGFD,PDE3A,BMPER,SMOC2,PPARA,ELAVL4,NTF3,RNF111,TBX20,FLRT2,TMEM108,SH3GL2,DDR2,SLIT2*
GO:0051966	regulation of synaptic transmission, glutamatergic	0.024256774300206545	*HCN1,GRIN2A,GRM3,TSHZ3,GRM7,STXBP1*
GO:0007167	enzyme-linked receptor protein signaling pathway	0.03278898761584116	*DMRT1,PTPRE,NEDD4,PDGFD,PAK3,SHC3,BMPER,ANGPT1,MVB12B,SMOC2,PPARA,LYN,NTF3,RNF111,TBX20,FLRT2,TMEM108,NEDD9,DDR2*
GO:0070848	response to growth factor	0.03290789318317691	*CD44,NEDD4,PDGFD,PDE3A,BMPER,SMOC2,PPARA,ELAVL4,NTF3,RNF111,TBX20,FLRT2,TMEM108,SH3GL2,DDR2,SLIT2*
GO:0120035	regulation of plasma membrane bounded cell projection organization	0.03940890045022272	*CD44,NEDD4,TANC2,DPYSL5,PAK3,IL1RAPL1,PLXNA4,LYN,ELAVL4,TIAM2,NEDD9,DDR2,DCC,SLIT2,MINAR1*
GO:0018108	peptidyl-tyrosine phosphorylation	0.043728441098152285	*CD44,IL6R,PDGFD,ANGPT1,PRKCE,LYN,SH3BP5,NTF3,NEDD9,DDR2*
GO:0018212	peptidyl-tyrosine modification	0.04634724530064585	*CD44,IL6R,PDGFD,ANGPT1,PRKCE,LYN,SH3BP5,NTF3,NEDD9,DDR2*
		CC	
GO:0045202	synapse	4.362099333251276e-10	*HCN1,DSCAML1,SLC16A7,NEDD4,CDH8,HCRTR1,SH3GL3,TANC2,PPFIBP1,ATXN3,PAK3,GABRA2,GRIN2A,MCTP2,IL1RAPL1,RPH3A,RPS6KA2,PLXNA4,IGSF21,PRKCE,GRM3,LYN,ELAVL4,TIAM2,NTF3,FLRT2,TMEM108,GABRB3,GRM7,KIRREL3,MYRIP,STXBP1,ANK3,SNTB1,SH3GL2,DCC,MCTP1*
GO:0030054	cell junction	2.289624393977403e-9	*CD44,HCN1,DSCAML1,SLC16A7,NEDD4,CDH8,RSU1,CDH18,HCRTR1,SH3GL3,TANC2,PPFIBP1,ATXN3,PAK3,GABRA2,GRIN2*

			A,MCTP2,IL1RAPL1,CTNNA3,RPH3A,RPS6KA2,PLXNA4,IGSF21,PRKCE,GRM3,LYN,ELAVL4,TIAM2,NTF3,FLRT2,TMEM108,GABRB3,GRM7,PTPRM,NEDD9,KIRREL3,MYRIP,STXBP1,ANK3,SNTB1,SH3GL2,DDR2,DCC,HMCN2,MCTP1
GO:0043005	neuron projection	9.305621446365178e-7	RPTOR,HCN1,AGBL4,DSCAML1,NEDD4,CDH8,BRINP3,TANC2,DPYSL5,GABRA2,GRIN2A,IL1RAPL1,RPH3A,GRM3,ELAVL4,TIAM2,TSHZ3,SLC24A4,FLRT2,BRINP2,TMEM108,GRM7,KIRREL3,MYRIP,STXBP1,ADCY2,ANK3,DCC,HMCN2,FEZ2
GO:0036477	somatodendritic compartment	0.000004890971925586654	RPTOR,HCN1,NEDD4,ASTN2,BRINP3,TANC2,DPYSL5,GABRA2,GRIN2A,IL1RAPL1,PDE1A,RPH3A,GRM3,ELAVL4,TIAM2,PDE10A,BRINP2,TMEM108,GRM7,KIRREL3,ADCY2,ANK3,HMCN2
GO:0098794	postsynapse	0.000005106055927063768	HCN1,SLC16A7,NEDD4,SH3GL3,TANC2,PAK3,GABRA2,GRIN2A,IL1RAPL1,RPH3A,IGSF21,GRM3,LYN,ELAVL4,TMEM108,GABRB3,GRM7,STXBP1,ANK3,DCC
GO:0120025	plasma membrane bounded cell projection	0.000017848094023113083	CD44,RPTOR,HCN1,AGBL4,DSCAML1,NEDD4,PKHD1,CDH8,BRINP3,TANC2,DPYSL5,GABRA2,GRIN2A,IL1RAPL1,CTNNA3,ANGPT1,RPH3A,TBATA,GRM3,ELAVL4,TIAM2,TSHZ3,SLC24A4,FLRT2,BRINP2,TMEM108,GRM7,PTPRM,NEDD9,KIRREL3,MYRIP,STXBP1,ADCY2,ANK3,DCC,HMCN2,FEZ2,FBXL13
GO:0042995	cell projection	0.000021057895866006035	CD44,RPTOR,HCN1,AGBL4,DSCAML1,NEDD4,PKHD1,CDH8,BRINP3,TANC2,DPYSL5,GABRA2,GRIN2A,IL1RAPL1,CTNNA3,ANGPT1,RPH3A,TBATA,GRM3,ELAVL4,TIAM2,TSHZ3,SLC24A4,FLRT2,BRINP2,TMEM108,GRM7,PTPRM,NEDD9,KIRREL3,MYRIP,STXBP1,ADCY2,ANK3,SH3GL2,DCC,HMCN2,FEZ2,FBXL13
GO:0030424	axon	0.00002522913756797758	HCN1,AGBL4,DSCAML1,CDH8,TANC2,GABRA2,IL1RAPL1,GRM3,ELAVL4,TIAM2,TSHZ3,TMEM108,GRM7,KIRREL3,STXBP1,ANK3,DCC,HMCN2,FEZ2
GO:0030425	dendrite	0.00007985591179037648	RPTOR,HCN1,NEDD4,BRINP3,TANC2,DPYSL5,GABRA2,GRIN2A,IL1RAPL1,RPH3A,GRM3,ELAVL4,BRINP2,TMEM108,GRM7,KIRREL3,ADCY2,ANK3
GO:0097447	dendritic tree	0.0000837189709823328	RPTOR,HCN1,NEDD4,BRINP3,TANC2,DPYSL5,GABRA2,GRIN2A,IL1RAPL1,RPH3A,GRM3,ELAVL4,BRINP2,TMEM108,GRM7,KIRREL3,ADCY2,ANK3
GO:0045211	postsynaptic membrane	0.000135769237820735	HCN1,SLC16A7,GABRA2,GRIN2A,IL1RAPL1,RPH3A,IGSF21,GRM3,GABRB3,GRM7,ANK3,DCC
GO:0097060	synaptic membrane	0.00016752457297704618	HCN1,SLC16A7,CDH8,GABRA2,GRIN2A,IL1RAPL1,RPH3A,IGSF21,GRM3,GABRB3,GRM7,STXBP1,ANK3,DCC
GO:0098793	presynapse	0.0004872919399178952	HCN1,CDH8,SH3GL3,PPFIBP1,GABRA2,GRIN2A,MCTP2,RPH3A,IGSF21,GRM3,NTF3,GRM7,KIRREL3,STXBP1,SH3GL2,MCTP1

GO:0098978	glutamatergic synapse	0.0024541127354646445	*HCN1,SLC16A7,CDH8,SH3GL3,PAK3,GRIN2A,IL1RAPL1,PLXNA4,GRM3,LYN,ELAVL4,STXBP1,SH3GL2*
GO:0098590	plasma membrane region	0.007968532401752437	*CD44,HCN1,SLC16A7,NEDD4,IL6R,PKHD1,CDH8,GABRA2,GRIN2A,IL1RAPL1,RPH3A,IGSF21,GRM3,SLC24A4,CFTR,GABRB3,GRM7,NEDD9,STXBP1,ANK3,DDR2,DCC,HMCN2*
GO:0071944	cell periphery	0.025051814649502156	*CD44,TACC2,MDFIC,HCN1,PTPRE,DSCAML1,SLC16A7,NEDD4,BACE2,IL6R,PKHD1,CDH8,CDH18,AKAP10,SPOCK3,HCRTR1,ASTN2,PPFIBP1,ATXN3,PAK3,GABRA2,SLC24A2,COL25A1,GRIN2A,SHC3,IL1RAPL1,BMPER,ANGPT1,RPH3A,MVB12B,PLXNA4,IGSF21,SMOC2,XPR1,PRKCE,GRM3,OR4F15,F13A1,ANO4,PRG4,LYN,NUBPL,TSHZ3,LINGO2,RASSF2,SLC24A4,CFTR,FLRT2,GABRB3,GRM7,NKAIN2,PTPRM,NEDD9,KIRREL3,MYRIP,STXBP1,ADCY2,ANK3,SNTB1,SH3GL2,DDR2,DCC,HMCN2,MINAR1*
GO:0016020	membrane	0.026907548066322937	*ST8SIA5,CD44,RPTOR,TACC2,MDFIC,HCN1,PTPRE,SLC12A8,DSCAML1,SLC16A7,NEDD4,SH3GLB1,BACE2,IL6R,PKHD1,CDH8,METTL15,RNF182,TMEM132C,CDH18,AKAP10,DEUP1,GALNT17,PDGFD,HCRTR1,SH3GL3,ASTN2,PPFIBP1,ATXN3,PAK3,SEL1L2,GABRA2,SPECC1,PDE3A,SLC24A2,COL25A1,GRIN2A,PCNX2,PRKAG2,MCTP2,SHC3,IL1RAPL1,KRT6A,ANGPT1,RPH3A,MVB12B,PLXNA4,IGSF21,XPR1,PRKCE,GRM3,OR4F15,ANO4,LYN,NUBPL,SH3BP5,TIAM2,TSHZ3,LINGO2,RASSF2,OSBPL1A,SLC24A4,MTHFD1L,CFTR,FLRT2,GAS2,ACER2,TMEM108,GABRB3,GRM7,NKAIN2,PTPRM,NEDD9,KIRREL3,STXBP1,ADCY2,ANK3,TMEM232,TMEM132B,SNTB1,SH3GL2,DDR2,DCC,TMEM132D,HMCN2,SLIT2,MINAR1,ARSJ,DPY19L1,MCTP1*
GO:0005737	cytoplasm	0.04318589025221875	*ST8SIA5,CD44,RPTOR,TACC2,DMRT1,MDFIC,TOP3A,PTPRE,ARPP21,AGBL4,TRIM43B,SLC16A7,NEDD4,SH3GLB1,DPYD,BACE2,PKHD1,CCDC18,METTL15,RSU1,MAP3K7CL,RNF182,ZFPM2,AKAP10,DEUP1,GALNT17,PDGFD,SH3GL3,ASTN2,BRINP3,DPYSL5,PPFIBP1,ATXN3,PAK3,PSIP1,SEL1L2,GABRA2,SPECC1,PDE3A,COL25A1,GRIN2A,PRKAG2,MCTP2,SHC3,IL1RAPL1,TXNDC16,CTNNA3,KRT6A,PDE1A,FGF14,RPH3A,RPS6KA2,MVB12B,TBATA,XPR1,PRKCE,MTUS2,F13A1,TAFA1,LYN,NUBPL,ELAVL4,MYSM1,SH3BP5,TIAM2,RASSF2,OSBPL1A,SLC24A4,MTHFD1L,NTF3,RNF111,CFTR,TBX20,FLRT2,GAS2,PDE10A,RBFOX2,BRINP2,ACER2,TMEM108,GABRB3,GRM7,BACH1,RASAL2,PTPRM,NEDD9,KIRREL3,MYRIP,PCP4,STXBP1,ADCY2,ANK3,SNTB1,SH3GL2,DCC,HMCN2,FEZ2,SLIT2,FBXO32,KLHL3,FBXL13,ADARB2,A*

			RSJ,BCKDHB,MCTP1

Table S9. GO associations with biological processes associated with 374 rDNA-contacting genes that are associated with lincRNAs in initial K562 cells. The search was performed using g:Profiler (https://biit.cs.ut.ee/gprofiler/gost). Related to Figure 2D.

GO.ID	Description	padj	Genes
GO:0043167	ion binding	2.447976807298887e-7	ZHX3,HIVEP1,ATRX,ADAMTS18,SEC24D,MPPED2,KMT2E,MYO9B,PACSIN2,SEC23B,ZEB1,FAT4,PARN,TRIM23,ZFYVE1,ROCK2,ACACA,SYT10,ZBTB38,ZNF648,USP49,MYO5B,ZZEF1,ZFAND4,KDM5A,ZNF382,ITGA1,ITSN2,EPHA4,TTLL11,DIDO1,ADAMTS2,PRKAA1,ABCC4,MYT1,ASH1L,ZC3HAV1,RAB38,ZNF891,ADAMTS19,RAB27B,MORC3,NSD1,PLS1,ABCC12,DSTYK,LPCAT2,ZNF518A,PHC2,DCAF1,KALRN,ZNF124,KIAA0753,TNKS,SMPDL3A,IMPA2,ZFP90,S100B,LTN1,KLF12,DIRAS2,KCNAB1,CDHR3,ITGB8,PRUNE2,POR,CDH11,AIF1L,ZNF780B,PNPLA8,LMX1A,ZNF292,ZNF611,EPHB1,TRIM58,ZMYM1,UBE2QL1,FER1L6,ZNF33B,HLCS,STK10,FLT1,ADSS2,PDE2A,ATP6V1B2,KCNJ1,NCS1,TRPC5,AMFR,PLCB4,LOXL2,FTO,SLC6A1,CDH9,ADAM28,CYP4Z1,ACSM2A,NIN,SLC1A1,HPCAL1,LIMCH1,CMPK1,BAZ2A,DDX6,PLEKHB2,PRKD1,PLCZ1,SNRK,ZFYVE26,DHX29,ZNF606,ESYT2,ZNF804B,MLLT10,CSNK1G1,ABCA4,PLEKHA2,ZMYM4,DEPTOR,VCAN,DDHD1,MATN2,CRACR2A,ZNF615,PHF20L1,ZNF431,IARS2,CELSR2,TET1,LMX1B,RRAGD,ZMAT4,MARCHF8,CLIP1,ZNF613,TLL1,PRDM15,UBE3A,TYW1,AGAP1,ZNF112,HTR2A,TPH2,GALNT1,CHN1,GATAD2B,TRPS1,INO80,RANBP2,ADCY9,ANO6,ERCC6L2,RUFY2,SUSD1,HIPK1,ACSM2B,HSPA12A,PEPD,AGO3,CNOT6L,ZNHIT6,ACTR3C
GO:0036094	small molecule binding	5.008752864240382e-7	ZHX3,HIVEP1,ATRX,ADAMTS18,SEC24D,MPPED2,KMT2E,MYO9B,PACSIN2,SEC23B,ZEB1,FAT4,PARN,TRIM23,ZFYVE1,ROCK2,ACACA,SYT10,ZBTB38,ZNF648,USP49,KL,MYO5B,ZZEF1,ZFAND4,KDM5A,ZNF382,ITGA1,ITSN2,EPHA4,TTLL11,DIDO1,ADAMTS2,PRKAA1,ABCC4,MYT1,ASH1L,ZC3HAV1,RAB38,ZNF891,ADAMTS19,RAB27B,MORC3,NSD1,PLS1,ABCC12,DSTYK,LPCAT2,ZNF518A,PHC2,DCAF1,KALRN,ZNF124,KIAA0753,TNKS,SMPDL3A,IMPA2,ZFP90,S100B,LTN1,KLF12,TINAG,DIRAS2,KCNAB1,CDHR3,ITGB8,PRUNE2,POR,CDH11,AIF1L,ZNF780B,PNPLA8,LMX1A,ZNF292,ZNF611,EPHB1,TRIM58,ZMYM1,UBE2QL1,FER1L6,ZNF33B,HLCS,STK10,FLT1,ADSS2,PDE2A,CBLIF,ATP6V1B2,KCNJ1,NCS1,TRPC5,AMFR,PLCB4,LOXL2,FTO,SLC6A1,CDH9,ADAM28,CYP4Z1,ACSM2A,NIN,SLC1A1,HPCAL1,LIMCH1,CMPK1,BAZ2A,DDX6,PLEKH

			B2,PRKD1,PLCZ1,SNRK,ZFYVE26,DHX29,ZNF606,ESYT2,ZNF804B,MLLT10,CSNK1G1,ABCA4,PLEKHA2,ZMYM4,DEPTOR,VCAN,DDHD1,MATN2,CRACR2A,ZNF615,PHF20L1,ZNF431,IARS2,CELSR2,TET1,LMX1B,RRAGD,ZMAT4,MARCHF8,CLIP1,ZNF613,TLL1,PRDM15,UBE3A,TYW1,AGAP1,ZNF112,HTR2A,TPH2,GALNT1,CHN1,GATAD2B,TRPS1,INO80,RANBP2,ADCY9,ANO6,ERCC6L2,RUFY2,SUSD1,HIPK1,ACSM2B,HSPA12A,PEPD,AGO3,CNOT6L,ZNHIT6,ACTR3C
GO:0046872	metal ion binding	5.676660044394386e-7	*ZHX3,HIVEP1,ATRX,ADAMTS18,SEC24D,MPPED2,KMT2E,MYO9B,SEC23B,ZEB1,FAT4,PARN,TRIM23,ZFYVE1,ROCK2,ACACA,SYT10,ZBTB38,ZNF648,USP49,ZZEF1,ZFAND4,KDM5A,ZNF382,ITGA1,ITSN2,TTLL11,DIDO1,ADAMTS2,PRKAA1,MYT1,ASH1L,ZC3HAV1,ZNF891,ADAMTS19,MORC3,NSD1,PLS1,LPCAT2,ZNF518A,PHC2,KALRN,ZNF124,KIAA0753,TNKS,SMPDL3A,IMPA2,ZFP90,S100B,LTN1,KLF12,CDHR3,ITGB8,PRUNE2,CDH11,AIF1L,ZNF780B,LMX1A,ZNF292,ZNF611,TRIM58,ZMYM1,FER1L6,ZNF33B,ADSS2,PDE2A,NCS1,AMFR,PLCB4,LOXL2,FTO,SLC6A1,CDH9,ADAM28,CYP4Z1,ACSM2A,NIN,SLC1A1,HPCAL1,LIMCH1,BAZ2A,PRKD1,PLCZ1,SNRK,ZFYVE26,ZNF606,ESYT2,ZNF804B,MLLT10,ZMYM4,VCAN,DDHD1,MATN2,CRACR2A,ZNF615,PHF20L1,ZNF431,CELSR2,TET1,LMX1B,ZMAT4,MARCHF8,CLIP1,ZNF613,TLL1,PRDM15,UBE3A,TYW1,AGAP1,ZNF112,TPH2,GALNT1,CHN1,GATAD2B,TRPS1,RANBP2,ADCY9,ANO6,RUFY2,SUSD1,ACSM2B,PEPD,AGO3,CNOT6L,ZNHIT6*
GO:0043169	cation binding	0.000001047943246191549	*ZHX3,HIVEP1,ATRX,ADAMTS18,SEC24D,MPPED2,KMT2E,MYO9B,SEC23B,ZEB1,FAT4,PARN,TRIM23,ZFYVE1,ROCK2,ACACA,SYT10,ZBTB38,ZNF648,USP49,ZZEF1,ZFAND4,KDM5A,ZNF382,ITGA1,ITSN2,TTLL11,DIDO1,ADAMTS2,PRKAA1,MYT1,ASH1L,ZC3HAV1,ZNF891,ADAMTS19,MORC3,NSD1,PLS1,LPCAT2,ZNF518A,PHC2,KALRN,ZNF124,KIAA0753,TNKS,SMPDL3A,IMPA2,ZFP90,S100B,LTN1,KLF12,CDHR3,ITGB8,PRUNE2,CDH11,AIF1L,ZNF780B,LMX1A,ZNF292,ZNF611,TRIM58,ZMYM1,FER1L6,ZNF33B,ADSS2,PDE2A,NCS1,AMFR,PLCB4,LOXL2,FTO,SLC6A1,CDH9,ADAM28,CYP4Z1,ACSM2A,NIN,SLC1A1,HPCAL1,LIMCH1,BAZ2A,PRKD1,PLCZ1,SNRK,ZFYVE26,ZNF606,ESYT2,ZNF804B,MLLT10,ZMYM4,VCAN,DDHD1,MATN2,CRACR2A,ZNF615,PHF20L1,ZNF431,CELSR2,TET1,LMX1B,ZMAT4,MARCHF8,CLIP1,ZNF613,TLL1,PRDM15,UBE3A,TYW1,AGAP1,ZNF112,HTR2A,TPH2,GALNT1,CHN1,GATAD2B,TRPS1,RANBP2,ADCY9,ANO6,RUFY2,SUSD1,ACSM2B,PEPD,AGO3,CNOT6L,ZNHIT6*

GO:0043167	ion binding	2.447976807298887e-7	*ZHX3,HIVEP1,ATRX,ADAMTS18,SEC24D,MPPED2,KMT2E,MYO9B,PACSIN2,SEC23B,ZEB1,FAT4,PARN,TRIM23,ZFYVE1,ROCK2,ACACA,SYT10,ZBTB38,ZNF648,USP49,MYO5B,ZZEF1,ZFAND4,KDM5A,ZNF382,ITGA1,ITSN2,EPHA4,TTLL11,DIDO1,ADAMTS2,PRKAA1,ABCC4,MYT1,ASH1L,ZC3HAV1,RAB38,ZNF891,ADAMTS19,RAB27B,MORC3,NSD1,PLS1,ABCC12,DSTYK,LPCAT2,ZNF518A,PHC2,DCAF1,KALRN,ZNF124,KIAA0753,TNKS,SMPDL3A,IMPA2,ZFP90,S100B,LTN1,KLF12,DIRAS2,KCNAB1,CDHR3,ITGB8,PRUNE2,POR,CDH11,AIF1L,ZNF780B,PNPLA8,LMX1A,ZNF292,ZNF611,EPHB1,TRIM58,ZMYM1,UBE2QL1,FER1L6,ZNF33B,HLCS,STK10,FLT1,ADSS2,PDE2A,ATP6V1B2,KCNJ1,NCS1,TRPC5,AMFR,PLCB4,LOXL2,FTO,SLC6A1,CDH9,ADAM28,CYP4Z1,ACSM2A,NIN,SLC1A1,HPCAL1,LIMCH1,CMPK1,BAZ2A,DDX6,PLEKHB2,PRKD1,PLCZ1,SNRK,ZFYVE26,DHX29,ZNF606,ESYT2,ZNF804B,MLLT10,CSNK1G1,ABCA4,PLEKHA2,ZMYM4,DEPTOR,VCAN,DDHD1,MATN2,CRACR2A,ZNF615,PHF20L1,ZNF431,IARS2,CELSR2,TET1,LMX1B,RRAGD,ZMAT4,MARCHF8,CLIP1,ZNF613,TLL1,PRDM15,UBE3A,TYW1,AGAP1,ZNF112,HTR2A,TPH2,GALNT1,CHN1,GATAD2B,TRPS1,INO80,RANBP2,ADCY9,ANO6,ERCC6L2,RUFY2,SUSD1,HIPK1,ACSM2B,HSPA12A,PEPD,AGO3,CNOT6L,ZNHIT6,ACTR3C*
GO:0036094	small molecule binding	5.008752864240382e-7	*ZHX3,HIVEP1,ATRX,ADAMTS18,SEC24D,MPPED2,KMT2E,MYO9B,PACSIN2,SEC23B,ZEB1,FAT4,PARN,TRIM23,ZFYVE1,ROCK2,ACACA,SYT10,ZBTB38,ZNF648,USP49,KL,MYO5B,ZZEF1,ZFAND4,KDM5A,ZNF382,ITGA1,ITSN2,EPHA4,TTLL11,DIDO1,ADAMTS2,PRKAA1,ABCC4,MYT1,ASH1L,ZC3HAV1,RAB38,ZNF891,ADAMTS19,RAB27B,MORC3,NSD1,PLS1,ABCC12,DSTYK,LPCAT2,ZNF518A,PHC2,DCAF1,KALRN,ZNF124,KIAA0753,TNKS,SMPDL3A,IMPA2,ZFP90,S100B,LTN1,KLF12,TINAG,DIRAS2,KCNAB1,CDHR3,ITGB8,PRUNE2,POR,CDH11,AIF1L,ZNF780B,PNPLA8,LMX1A,ZNF292,ZNF611,EPHB1,TRIM58,ZMYM1,UBE2QL1,FER1L6,ZNF33B,HLCS,STK10,FLT1,ADSS2,PDE2A,CBLIF,ATP6V1B2,KCNJ1,NCS1,TRPC5,AMFR,PLCB4,LOXL2,FTO,SLC6A1,CDH9,ADAM28,CYP4Z1,ACSM2A,NIN,SLC1A1,HPCAL1,LIMCH1,CMPK1,BAZ2A,DDX6,PLEKHB2,PRKD1,PLCZ1,SNRK,ZFYVE26,DHX29,ZNF606,ESYT2,ZNF804B,MLLT10,CSNK1G1,ABCA4,PLEKHA2,ZMYM4,DEPTOR,VCAN,DDHD1,MATN2,CRACR2A,ZNF615,PHF20L1,ZNF431,IARS2,CELSR2,TET1,LMX1B,RRAGD,ZMAT4,MARCHF8,CLIP1,ZNF613,TLL1,PRDM15,UBE3A,TYW1,AGAP1,ZNF112,HTR2A,TPH2,GALNT1,CHN1,GATAD2B,TRPS*

			1,INO80,RANBP2,ADCY9,ANO6,ERCC6L2,RUFY2,SUSD1,HIPK1,ACSM2B,HSPA12A,PEPD,AGO3,CNOT6L,ZNHIT6,ACTR3C
GO:0046872	metal ion binding	5.676660044394386e-7	*ZHX3,HIVEP1,ATRX,ADAMTS18,SEC24D,MPPED2,KMT2E,MYO9B,SEC23B,ZEB1,FAT4,PARN,TRIM23,ZFYVE1,ROCK2,ACACA,SYT10,ZBTB38,ZNF648,USP49,ZZEF1,ZFAND4,KDM5A,ZNF382,ITGA1,ITSN2,TTLL11,DIDO1,ADAMTS2,PRKAA1,MYT1,ASH1L,ZC3HAV1,ZNF891,ADAMTS19,MORC3,NSD1,PLS1,LPCAT2,ZNF518A,PHC2,KALRN,ZNF124,KIAA0753,TNKS,SMPDL3A,IMPA2,ZFP90,S100B,LTN1,KLF12,CDHR3,ITGB8,PRUNE2,CDH11,AIF1L,ZNF780B,LMX1A,ZNF292,ZNF611,TRIM58,ZMYM1,FER1L6,ZNF33B,ADSS2,PDE2A,NCS1,AMFR,PLCB4,LOXL2,FTO,SLC6A1,CDH9,ADAM28,CYP4Z1,ACSM2A,NIN,SLC1A1,HPCAL1,LIMCH1,BAZ2A,PRKD1,PLCZ1,SNRK,ZFYVE26,ZNF606,ESYT2,ZNF804B,MLLT10,ZMYM4,VCAN,DDHD1,MATN2,CRACR2A,ZNF615,PHF20L1,ZNF431,CELSR2,TET1,LMX1B,ZMAT4,MARCHF8,CLIP1,ZNF613,TLL1,PRDM15,UBE3A,TYW1,AGAP1,ZNF112,TPH2,GALNT1,CHN1,GATAD2B,TRPS1,RANBP2,ADCY9,ANO6,RUFY2,SUSD1,ACSM2B,PEPD,AGO3,CNOT6L,ZNHIT6*
GO:0043169	cation binding	0.000001047943246191549	*ZHX3,HIVEP1,ATRX,ADAMTS18,SEC24D,MPPED2,KMT2E,MYO9B,SEC23B,ZEB1,FAT4,PARN,TRIM23,ZFYVE1,ROCK2,ACACA,SYT10,ZBTB38,ZNF648,USP49,ZZEF1,ZFAND4,KDM5A,ZNF382,ITGA1,ITSN2,TTLL11,DIDO1,ADAMTS2,PRKAA1,MYT1,ASH1L,ZC3HAV1,ZNF891,ADAMTS19,MORC3,NSD1,PLS1,LPCAT2,ZNF518A,PHC2,KALRN,ZNF124,KIAA0753,TNKS,SMPDL3A,IMPA2,ZFP90,S100B,LTN1,KLF12,CDHR3,ITGB8,PRUNE2,CDH11,AIF1L,ZNF780B,LMX1A,ZNF292,ZNF611,TRIM58,ZMYM1,FER1L6,ZNF33B,ADSS2,PDE2A,NCS1,AMFR,PLCB4,LOXL2,FTO,SLC6A1,CDH9,ADAM28,CYP4Z1,ACSM2A,NIN,SLC1A1,HPCAL1,LIMCH1,BAZ2A,PRKD1,PLCZ1,SNRK,ZFYVE26,ZNF606,ESYT2,ZNF804B,MLLT10,ZMYM4,VCAN,DDHD1,MATN2,CRACR2A,ZNF615,PHF20L1,ZNF431,CELSR2,TET1,LMX1B,ZMAT4,MARCHF8,CLIP1,ZNF613,TLL1,PRDM15,UBE3A,TYW1,AGAP1,ZNF112,HTR2A,TPH2,GALNT1,CHN1,GATAD2B,TRPS1,RANBP2,ADCY9,ANO6,RUFY2,SUSD1,ACSM2B,PEPD,AGO3,CNOT6L,ZNHIT6*
BP			
GO:0065007	biological regulation	0.00195200699746543	*MAP4,PKNOX2,ZHX3,HIVEP1,ATRX,OR1L6,NBN,PRTG,ADAMTS18,KMT2E,PELI2,MYO9B,PACSIN2,ZEB1,FAT4,PARN,SLC15A2,TRIM23,FBXW8,DIP2A,SFPQ,HOOK3,GPR137B,ROCK2,C1QL3,POU1F1,TSPAN13,YTHDF3,SYT10,ZBTB38,PAFAH1B1,GON4L,ZNF648,ARFGEF3,BCL2,KL,MYO5B,PPP1R9A*

			,RAP1GAP,SPOPL,INO80D,KDM5A,NRIP1,ZNF382,ITGA1,ITSN2,HIRA,UFL1,EPHA4,DIDO1,COL4A3,PRKAA1,RASGEF1C,PBLD,ABCC4,MYT1,ASH1L,SORBS2,PIK3R3,MEGF10,OPA3,ZC3HAV1,TMEM25,RAB38,ZNF891,VMP1,RALGAPA2,VSTM4,RAB27B,CTSB,MORC3,NDRG2,NSD1,NEDD4L,PLS1,HDGFL3,ANK2,DSTYK,XRCC4,BZW1,FBXL20,ZNF518A,PHC2,DCAF1,NSG2,CD2AP,RGS8,KALRN,ZNF124,BMP7,ANKRD17,TNKS,OR2T2,IMPA2,SCAI,ZFP90,S100B,ARHGAP12,KLF12,DIRAS2,FLI1,ANAPC1,KCNAB1,CDHR3,ITGB8,POR,CDH11,SETBP1,ZNF780B,RBPMS2,ATP6V1E1,PNPLA8,LMX1A,KHDRBS2,VPS13D,ZNF292,KPNA1,FUT8,ADCYAP1R1,LAMC1,ZNF611,SRP9,EPHB1,MYOCD,TRIM58,PLPPR5,FER1L6,ZNF33B,PTCD2,STK10,GAPVD1,INTS7,SOGA1,FLT1,RB1CC1,NPHP4,FOXJ3,PDE2A,IL17RD,ATP6V1B2,KCNJ1,NCS1,TRPC5,AMFR,PLCB4,LOXL2,FTO,SLC6A1,EPC2,FGF9,TRAPPC11,NIN,SLC1A1,RAB3GAP2,LIMCH1,CRACD,BAZ2A,MEOX2,DDX6,TRPM1,CACNG3,PLEKHB2,COPS8,AKAP9,PRKD1,PLCZ1,RBM47,SHROOM3,MSI2,ETS1,SYNE1,RALGPS2,SNRK,HRH1,RABEP1,TOGARAM1,ALKAL2,PLPPR1,MAP2,TGFA,ZFYVE26,DHX29,PTPRA,NUMB,TNRC6C,CELF2,CD70,TM9SF2,ZNF606,CAPN5,MLLT10,CSNK1G1,ABCA4,ZMYM4,DEPTOR,VCAN,ATAT1,RBMS3,DDHD1,ICA1,RGS6,CRACR2A,ZNF615,ATP6V0D2,PHF20L1,ZNF431,IARS2,CELSR2,PTPRJ,TET1,RALGAPA1,LMX1B,RRAGD,SHLD2,CLIP1,SHC4,ELP2,ZNF613,UST,PRDM15,UBE3A,HSF5,ADAM22,ZNF112,HTR2A,NCOA7,CHN1,GATAD2B,TRPS1,INO80,RANBP2,STRN,EIPR1,CHCHD2,CLDN18,ADCY9,GEMIN5,ANO6,RUFY2,OR4K2,HIPK1,KCNE4,PEPD,BRWD1,AGO3,CNOT6L,MEF2C,TRAPPC6B,MYB,SLC39A6
GO:0050789	regulation of biological process	0.003483739098752321	MAP4,PKNOX2,ZHX3,HIVEP1,ATRX,OR1L6,NBN,PRTG,ADAMTS18,KMT2E,PELI2,MYO9B,PACSIN2,ZEB1,FAT4,PARN,SLC15A2,TRIM23,FBXW8,DIP2A,SFPQ,HOOK3,GPR137B,ROCK2,C1QL3,POU1F1,TSPAN13,YTHDF3,SYT10,ZBTB38,PAFAH1B1,GON4L,ZNF648,ARFGEF3,BCL2,KL,PPP1R9A,RAP1GAP,SPOPL,INO80D,KDM5A,NRIP1,ZNF382,ITGA1,ITSN2,HIRA,UFL1,EPHA4,DIDO1,COL4A3,PRKAA1,RASGEF1C,PBLD,ABCC4,MYT1,ASH1L,SORBS2,PIK3R3,MEGF10,OPA3,ZC3HAV1,TMEM25,RAB38,ZNF891,VMP1,RALGAPA2,RAB27B,CTSB,MORC3,NDRG2,NSD1,NEDD4L,PLS1,HDGFL3,ANK2,DSTYK,BZW1,FBXL20,ZNF518A,PHC2,DCAF1,NSG2,CD2AP,RGS8,KALRN,ZNF124,BMP7,ANKRD17,TNKS,OR2T2,IMPA2,SCAI,ZFP90,S100B,ARHGAP12,KLF12,DIRAS2,FLI1,ANAPC1,KCNAB1,CDHR3,ITGB8,CDH11,S

			ETBP1,ZNF780B,RBPMS2,ATP6V1E1,PNPLA8,LMX1A,KHDRBS2,VPS13D,ZNF292,KPNA1,FUT8,ADCYAP1R1,LAMC1,ZNF611,SRP9,EPHB1,MYOCD,TRIM58,PLPPR5,FER1L6,ZNF33B,PTCD2,STK10,GAPVD1,INTS7,SOGA1,FLT1,RB1CC1,NPHP4,FOXJ3,PDE2A,IL17RD,ATP6V1B2,KCNJ1,NCS1,TRPC5,AMFR,PLCB4,LOXL2,FTO,SLC6A1,EPC2,FGF9,NIN,SLC1A1,RAB3GAP2,LIMCH1,CRACD,BAZ2A,MEOX2,DDX6,TRPM1,CACNG3,PLEKHB2,COPS8,AKAP9,PRKD1,PLCZ1,RBM47,SHROOM3,MSI2,ETS1,SYNE1,RALGPS2,SNRK,HRH1,RABEP1,TOGARAM1,ALKAL2,PLPPR1,MAP2,TGFA,ZFYVE26,DHX29,PTPRA,NUMB,TNRC6C,CELF2,CD70,TM9SF2,ZNF606,CAPN5,MLLT10,CSNK1G1,ABCA4,ZMYM4,DEPTOR,VCAN,ATAT1,RBMS3,DDHD1,ICA1,RGS6,CRACR2A,ZNF615,ATP6V0D2,PHF20L1,ZNF431,CELSR2,PTPRJ,TET1,RALGAPA1,LMX1B,RRAGD,SHLD2,CLIP1,SHC4,ELP2,ZNF613,UST,PRDM15,UBE3A,HSF5,ADAM22,ZNF112,HTR2A,NCOA7,CHN1,GATAD2B,TRPS1,INO80,RANBP2,STRN,EIPR1,CHCHD2,CLDN18,ADCY9,GEMIN5,ANO6,RUFY2,OR4K2,HIPK1,KCNE4,PEPD,BRWD1,AGO3,CNOT6L,MEF2C,MYB,SLC39A6
GO:0031175	neuron projection development	0.004079893129277976	*MAP4,PRTG,FAT4,FBXW8,DIP2A,PAFAH1B1,BCL2,PPP1R9A,ITGA1,ITSN2,EPHA4,FRYL,NEDD4L,PLS1,HDGFL3,POTEJ,KALRN,BMP7,S100B,CDH11,LMX1A,EPHB1,PLPPR5,NCS1,TRPC5,NIN,PRKD1,SYNE1,ALKAL2,MAP2,NUMB,MATN2,CELSR2,UST,UBE3A,CHN1,STRN,MEF2C*
GO:0050794	regulation of cellular process	0.004632931329085173	*MAP4,PKNOX2,ZHX3,HIVEP1,ATRX,OR1L6,NBN,PRTG,ADAMTS18,KMT2E,PELI2,MYO9B,PACSIN2,ZEB1,FAT4,PARN,SLC15A2,TRIM23,FBXW8,DIP2A,SFPQ,HOOK3,GPR137B,ROCK2,C1QL3,POU1F1,TSPAN13,YTHDF3,SYT10,ZBTB38,PAFAH1B1,GON4L,ZNF648,ARFGEF3,BCL2,KL,PPP1R9A,RAP1GAP,INO80D,KDM5A,NRIP1,ZNF382,ITGA1,ITSN2,HIRA,UFL1,EPHA4,DIDO1,COL4A3,PRKAA1,RASGEF1C,PBLD,MYT1,ASH1L,SORBS2,PIK3R3,MEGF10,ZC3HAV1,TMEM25,RAB38,ZNF891,VMP1,RALGAPA2,RAB27B,CTSB,MORC3,NDRG2,NSD1,NEDD4L,PLS1,HDGFL3,ANK2,DSTYK,BZW1,FBXL20,ZNF518A,PHC2,DCAF1,NSG2,CD2AP,RGS8,KALRN,ZNF124,BMP7,ANKRD17,TNKS,OR2T2,IMPA2,SCAI,ZFP90,S100B,ARHGAP12,KLF12,DIRAS2,FLI1,ANAPC1,KCNAB1,ITGB8,CDH11,SETBP1,ZNF780B,RBPMS2,ATP6V1E1,PNPLA8,LMX1A,KHDRBS2,VPS13D,ZNF292,KPNA1,FUT8,ADCYAP1R1,LAMC1,ZNF611,SRP9,EPHB1,MYOCD,TRIM58,PLPPR5,FER1L6,ZNF33B,PTCD2,STK10,GAPVD1,INTS7,SOGA1,FLT1,RB1CC1,NPHP4,FOXJ3,PDE2A,IL17RD,ATP6V1B2,KCNJ1,NCS1,TRPC5,AMFR,PLCB4,LOXL2,FTO,SL*

			C6A1,EPC2,FGF9,NIN,SLC1A1,RAB3GAP2,LIMCH1,CRACD,BAZ2A,MEOX2,DDX6,TRPM1,CACNG3,PLEKHB2,COPS8,AKAP9,PRKD1,PLCZ1,RBM47,MSI2,ETS1,SYNE1,RALGPS2,SNRK,HRH1,RABEP1,TOGARAM1,ALKAL2,PLPPR1,MAP2,TGFA,ZFYVE26,DHX29,PTPRA,NUMB,TNRC6C,CELF2,CD70,TM9SF2,ZNF606,CAPN5,MLLT10,CSNK1G1,ABCA4,DEPTOR,VCAN,ATAT1,RBMS3,DDHD1,RGS6,CRACR2A,ZNF615,ATP6V0D2,PHF20L1,ZNF431,CELSR2,PTPRJ,TET1,RALGAPA1,LMX1B,RRAGD,SHLD2,CLIP1,SHC4,ELP2,ZNF613,UST,PRDM15,UBE3A,HSF5,ADAM22,ZNF112,HTR2A,NCOA7,CHN1,GATAD2B,TRPS1,INO80,STRN,EIPR1,CHCHD2,CLDN18,ADCY9,GEMIN5,ANO6,RUFY2,OR4K2,HIPK1,PEPD,BRWD1,AGO3,CNOT6L,MEF2C,MYB,SLC39A6
GO:0016358	dendrite development	0.00479205667545515	FBXW8,DIP2A,PAFAH1B1,EPHA4,NEDD4L,KALRN,BMP7,EPHB1,TRPC5,SYNE1,MAP2,MATN2,CELSR2,UBE3A,STRN,MEF2C
GO:0032502	developmental process	0.005141066767646673	MAP4,ZHX3,ATRX,NBN,PRTG,ADAMTS18,SEC24D,KMT2E,MYO9B,PACSIN2,ZEB1,FAT4,FBXW8,DIP2A,HOOK3,GPR137B,ROCK2,C1QL3,POU1F1,CCDC141,YTHDF3,PAFAH1B1,BCL2,KL,PPP1R9A,RAP1GAP,INO80D,NRIP1,ITGA1,ITSN2,HIRA,UFL1,EPHA4,COL4A3,ADAMTS2,PRKAA1,FRYL,MYT1,ASH1L,SORBS2,PIK3R3,MEGF10,RAB38,VMP1,VSTM4,CTSB,MORC3,NDRG2,NEDD4L,PLS1,HDGFL3,ANK2,XRCC4,POTEJ,PHC2,DCAF1,KALRN,KIAA0319L,BMP7,ANKRD17,S100B,ARHGAP12,FLI1,CDHR3,ITGB8,CDH11,RBPMS2,ATP6V1E1,LMX1A,KPNA1,FUT8,ADCYAP1R1,LAMC1,EPHB1,MYOCD,TRIM58,PLPPR5,PTCD2,FLT1,RB1CC1,NPHP4,FOXJ3,PDE2A,IL17RD,ATP6V1B2,NCS1,TRPC5,LOXL2,FTO,CDH9,ADAM28,FGF9,NIN,SLC1A1,MEOX2,DDX6,PLEKHB2,PRKD1,RBM47,SHROOM3,MSI2,ETS1,SYNE1,SNRK,ALKAL2,PLPPR1,MAP2,TGFA,NUMB,SMTN,ZMYM4,VCAN,ATAT1,DDHD1,MATN2,CRACR2A,ZNF431,CELSR2,CFAP97,PTPRJ,TET1,CATSPER2,LMX1B,SHLD2,SHC4,UST,TLL1,CALD1,UBE3A,ADAM22,HTR2A,CHN1,GATAD2B,TRPS1,INO80,STRN,CRISPLD2,CLDN18,ADCY9,ANO6,HIPK1,BRWD1,MEF2C,TRAPPC6B,MYB,SLC39A6
GO:0048813	dendrite morphogenesis	0.008936902254655548	FBXW8,DIP2A,PAFAH1B1,EPHA4,NEDD4L,KALRN,EPHB1,TRPC5,SYNE1,MAP2,CELSR2,UBE3A
GO:0000902	cell morphogenesis	0.013897165395899901	PRTG,MYO9B,PACSIN2,FBXW8,DIP2A,PAFAH1B1,BCL2,ITGA1,ITSN2,EPHA4,FRYL,NEDD4L,PLS1,POTEJ,KALRN,BMP7,S100B,CDHR3,CDH11,LMX1A,EPHB1,TRPC5,CDH9,NIN,SHROOM3,SYNE1,MAP2,NUMB,ZMYM4,MATN2,CELSR2,UST,UBE3A,CHN1,BRWD1,MEF2C

GO:0048667	cell morphogenesis involved in neuron differentiation	0.014636586704668964	*PRTG,FBXW8,DIP2A,PAFAH1B1,BCL2,EPHA4,NEDD4L,PLS1,POTEJ,KALRN,BMP7,S100B,CDH11,LMX1A,EPHB1,TRPC5,NIN,SYNE1,MAP2,NUMB,MATN2,CELSR2,UST,UBE3A,CHN1,MEF2C*
GO:0048858	cell projection morphogenesis	0.016439276546125627	*PRTG,MYO9B,PACSIN2,FBXW8,DIP2A,PAFAH1B1,BCL2,ITGA1,ITSN2,EPHA4,NEDD4L,POTEJ,KALRN,BMP7,S100B,CDH11,LMX1A,EPHB1,TRPC5,NIN,SYNE1,MAP2,NUMB,MATN2,CELSR2,UST,UBE3A,CHN1*
GO:0048699	generation of neurons	0.017789961757975727	*MAP4,NBN,PRTG,ZEB1,FAT4,FBXW8,DIP2A,PAFAH1B1,BCL2,PPP1R9A,RAP1GAP,ITGA1,ITSN2,EPHA4,FRYL,NEDD4L,PLS1,HDGFL3,POTEJ,KALRN,KIAA0319L,BMP7,S100B,CDH11,LMX1A,EPHB1,PLPPR5,NPHP4,NCS1,TRPC5,NIN,DDX6,PRKD1,SYNE1,ALKAL2,MAP2,NUMB,VCAN,ATAT1,MATN2,CELSR2,LMX1B,UST,UBE3A,CHN1,STRN,HIPK1,MEF2C*
GO:0022008	neurogenesis	0.018340249407298108	*MAP4,NBN,PRTG,ZEB1,FAT4,FBXW8,DIP2A,HOOK3,PAFAH1B1,BCL2,PPP1R9A,RAP1GAP,ITGA1,ITSN2,UFL1,EPHA4,FRYL,NEDD4L,PLS1,HDGFL3,POTEJ,KALRN,KIAA0319L,BMP7,S100B,CDH11,LMX1A,EPHB1,PLPPR5,NPHP4,NCS1,TRPC5,NIN,SLC1A1,DDX6,PRKD1,SYNE1,ALKAL2,MAP2,NUMB,VCAN,ATAT1,MATN2,CELSR2,LMX1B,UST,UBE3A,ADAM22,CHN1,STRN,HIPK1,MEF2C,MYB*
GO:0048666	neuron development	0.018574943327500554	*MAP4,PRTG,FAT4,FBXW8,DIP2A,PAFAH1B1,BCL2,PPP1R9A,ITGA1,ITSN2,EPHA4,FRYL,NEDD4L,PLS1,HDGFL3,POTEJ,KALRN,BMP7,S100B,CDH11,LMX1A,EPHB1,PLPPR5,NPHP4,NCS1,TRPC5,NIN,PRKD1,SYNE1,ALKAL2,MAP2,NUMB,ATAT1,MATN2,CELSR2,UST,UBE3A,CHN1,STRN,MEF2C*
GO:0031331	positive regulation of cellular catabolic process	0.023026859485269134	*PARN,TRIM23,ROCK2,YTHDF3,UFL1,PRKAA1,ZC3HAV1,VPS13D,TRIM58,RB1CC1,FTO,RAB3GAP2,PRKD1,TNRC6C,DEPTOR,HTR2A,AGO3,CNOT6L*
GO:0098662	inorganic cation transmembrane transport	0.0243561941888359	*SLC15A2,TSPAN13,BCL2,SLC13A4,VMP1,SLC25A18,NEDD4L,ANK2,KCNN3,KCNAB1,ATP6V1E1,ADCYAP1R1,CBLIF,ATP6V1B2,KCNJ1,NCS1,TRPC5,PLCB4,SLC6A1,SLC1A1,TRPM1,CACNG3,AKAP9,PRKD1,CRACR2A,ATP6V0D2,CATSPER2,HTR2A,ANO6,KCNE4,SLC39A6*
GO:0048856	anatomical structure development	0.02630830178799854	*MAP4,ATRX,NBN,PRTG,ADAMTS18,SEC24D,KMT2E,MYO9B,PACSIN2,ZEB1,FAT4,FBXW8,DIP2A,HOOK3,GPR137B,ROCK2,C1QL3,POU1F1,CCDC141,YTHDF3,PAFAH1B1,BCL2,KL,PPP1R9A,RAP1GAP,INO80D,NRIP1,ITGA1,ITSN2,HIRA,UFL1,EPHA4,COL4A3,ADAMTS2,PRKAA1,FRYL,MYT1,ASH1L,SORBS2,PIK3R3,MEGF10,VMP1,VSTM4,CTS*

			B,MORC3,NDRG2,NEDD4L,PLS1,HDGFL3,ANK2,XRCC4,POTEJ,DCAF1,KALRN,KIAA0319L,BMP7,ANKRD17,S100B,ARHGAP12,FLI1,CDHR3,ITGB8,CDH11,RBPMS2,LMX1A,KPNA1,FUT8,ADCYAP1R1,LAMC1,EPHB1,MYOCD,TRIM58,PLPPR5,PTCD2,FLT1,RB1CC1,NPHP4,PDE2A,IL17RD,NCS1,TRPC5,LOXL2,FTO,CDH9,FGF9,NIN,SLC1A1,MEOX2,DDX6,PRKD1,RBM47,SHROOM3,MSI2,ETS1,SYNE1,SNRK,ALKAL2,PLPPR1,MAP2,TGFA,NUMB,SMTN,ZMYM4,VCAN,ATAT1,DDHD1,MATN2,CRACR2A,CELSR2,PTPRJ,TET1,CATSPER2,LMX1B,SHLD2,UST,TLL1,CALD1,UBE3A,ADAM22,CHN1,TRPS1,INO80,STRN,CRISPLD2,CLDN18,ADCY9,ANO6,HIPK1,BRWD1,MEF2C,TRAPPC6B,MYB,SLC39A6
GO:0007275	multicellular organism development	0.027628340132392425	MAP4,ATRX,NBN,PRTG,ADAMTS18,SEC24D,ZEB1,FAT4,FBXW8,DIP2A,HOOK3,GPR137B,ROCK2,C1QL3,POU1F1,CCDC141,YTHDF3,PAFAH1B1,BCL2,KL,PPP1R9A,RAP1GAP,INO80D,NRIP1,ITGA1,ITSN2,HIRA,UFL1,EPHA4,COL4A3,ADAMTS2,FRYL,MYT1,ASH1L,SORBS2,PIK3R3,VMP1,VSTM4,MORC3,NDRG2,NEDD4L,PLS1,HDGFL3,ANK2,XRCC4,POTEJ,DCAF1,KALRN,KIAA0319L,BMP7,ANKRD17,S100B,ITGB8,CDH11,RBPMS2,LMX1A,FUT8,ADCYAP1R1,EPHB1,MYOCD,PLPPR5,PTCD2,FLT1,RB1CC1,NPHP4,PDE2A,NCS1,TRPC5,LOXL2,CDH9,FGF9,NIN,SLC1A1,MEOX2,DDX6,PRKD1,SHROOM3,MSI2,ETS1,SYNE1,ALKAL2,PLPPR1,MAP2,TGFA,NUMB,VCAN,ATAT1,MATN2,CELSR2,PTPRJ,TET1,LMX1B,SHLD2,UST,TLL1,CALD1,UBE3A,ADAM22,CHN1,TRPS1,INO80,STRN,CRISPLD2,CLDN18,ADCY9,ANO6,HIPK1,MEF2C,TRAPPC6B,MYB
GO:0098660	inorganic ion transmembrane transport	0.03176022204331626	SLC15A2,TSPAN13,BCL2,SLC13A4,VMP1,SLC25A18,NEDD4L,ANK2,KCNN3,KCNAB1,ATP6V1E1,SLC37A1,ADCYAP1R1,CBLIF,ATP6V1B2,KCNJ1,NCS1,TRPC5,PLCB4,SLC6A1,SLC1A1,TRPM1,CACNG3,AKAP9,PRKD1,CRACR2A,ATP6V0D2,CATSPER2,HTR2A,ANO6,KCNE4,CLIC6,SLC39A6
GO:0032989	cellular anatomical entity morphogenesis	0.036579289930047935	PRTG,MYO9B,PACSIN2,FBXW8,DIP2A,PAFAH1B1,BCL2,ITGA1,ITSN2,EPHA4,NEDD4L,ANK2,POTEJ,KALRN,BMP7,S100B,CDH11,LMX1A,EPHB1,TRPC5,NIN,PRKD1,SYNE1,MAP2,NUMB,MATN2,CELSR2,UST,UBE3A,CHN1
GO:0120039	plasma membrane bounded cell projection morphogenesis	0.040184439041797256	PRTG,MYO9B,FBXW8,DIP2A,PAFAH1B1,BCL2,ITGA1,ITSN2,EPHA4,NEDD4L,POTEJ,KALRN,BMP7,S100B,CDH11,LMX1A,EPHB1,TRPC5,NIN,SYNE1,MAP2,NUMB,MATN2,CELSR2,UST,UBE3A,CHN1
GO:0098655	monoatomic	0.041062670075	SLC15A2,TSPAN13,BCL2,SLC13A4,VMP1,SLC25A18,NEDD4L,ANK2,KCNN3,KCNAB1,

	cation transmembrane transport	40742	*ATP6V1E1,ADCYAP1R1,CBLIF,ATP6V1B2,KCNJ1,NCS1,TRPC5,PLCB4,SLC6A1,SLC1A1,TRPM1,CACNG3,AKAP9,PRKD1,CRACR2A,ATP6V0D2,CATSPER2,HTR2A,ANO6,KCNE4,SLC39A6*
GO:0030182	neuron differentiation	0.04320531359330936	*MAP4,PRTG,ZEB1,FAT4,FBXW8,DIP2A,PAFAH1B1,BCL2,PPP1R9A,RAP1GAP,ITGA1,ITSN2,EPHA4,FRYL,NEDD4L,PLS1,HDGFL3,POTEJ,KALRN,BMP7,S100B,CDH11,LMX1A,EPHB1,PLPPR5,NPHP4,NCS1,TRPC5,NIN,DDX6,PRKD1,SYNE1,ALKAL2,MAP2,NUMB,ATAT1,MATN2,CELSR2,LMX1B,UST,UBE3A,CHN1,STRN,HIPK1,MEF2C*

Table S10. The intersections among lincRNAs associated with rDNA-conataninig genes in HEK293T cells, in K562 before (K562) and after induced differentiation (K562-diff.) Related to the Venn diagram presented in Figure 3B.

Names	total	elements
linc-HEK293T linc-K562-diff. linc-K562	31	*CXXC4-AS1 PRICKLE2-AS1 LINC02552 SLC8A1-AS1 LINC01741 LINC01602 EDIL3-DT MKLN1-AS LINC02720 NKAIN3-IT1 ARHGEF7-AS1 SEMA6A-AS2 ZNF32-AS3 ADAMTS9-AS2 ARHGEF7-IT1 FRMPD3-AS1 LINC01640 ANKRD62P1-PARP4P3 LINC00689 LINC01651 SLC6A1-AS1 PRICKLE2-AS3 GSN-AS1 NMBR-AS1 LINC02598 LINC02520 PPP1R12A-AS1 PGR-AS1 NNT-AS1 CHL1-AS1 LRRC7-AS1*
HEK293T linc-K562-diff.	36	*LINC02051 TRAF3IP2-AS1 LINC01074 LINC02339 LINC02226 LMCD1-AS1 MAPT-AS1 CPB2-AS1 LINC01415 ASIC4-AS1 ARHGEF26-AS1 GNG12-AS1 LINC00928 LINC00945 OPCML-IT1 LINC01546 LINC02060 LINC02058 KCNMA1-AS1 LINC00461 LINC02774 NCAM1-AS1 JAKMIP2-AS1 DSCAM-IT1 LINC02008 LINC00391 LINC01690 SEC23A-AS1 TMEM72-AS1 SOX21-AS1 LINC01572 LRP4-AS1 NRXN2-AS1 CCND2-AS1 LINC01351 GPR158-AS1*
linc-K562-diff. linc-linc-K562	146	*MYCBP2-AS1 SHANK2-AS3 TGFA-IT1 NCKAP5-AS1 PKP4-AS1 AGBL4-IT1 C1QTNF7-AS1 WWTR1-IT1 FGF10-AS1 KCNMA1-AS3 LINC00517 ID2-AS1 LINC00839 SFTPD-AS1 MSC-AS1 SYNE1-AS1 LINC02017 LINC02115 LINC01945 TSPAN18-AS1 MYO16-AS2 GLCCI1-DT EGFLAM-AS3 RORB-AS1 LINC02676 LINC01761 LINC00362 LINC02794 MAPK10-AS1 ANO3-AS1 LINC02157 PCDH9-AS4 DOCK4-AS1 LINC00710 CNTN4-AS2 DLG2-AS2 TSC22D1-AS1 CREB3L2-AS1 RORA-AS2 PTPRG-AS1 LINC01906 FAM13A-AS1 LINC02024 LINC00836 PEX5L-AS2 NAV2-AS2 TPM1-AS LINC00446 UST-AS1 LINC01069 FAM198B-AS1 LINC01087 SH3TC2-DT SLC25A48-AS1 OIP5-AS1 GPC6-AS2 LINC00499 LINC01924 LAMP5-AS1 SHANK2-AS2 LINC00484 LINC02827 OBI1-AS1 LINC01751 PRICKLE2-AS2 RAMP2-AS1 LAMC1-AS1 LINC02556 EGFR-AS1 LINC01739 WDFY3-AS1 MFF-DT LRRC8C-DT SRGAP3-AS1 LDLRAD4-AS1 EDRF1-AS1 FRMD6-AS1 SMAD9-IT1 PKIA-AS1 NAV2-AS1 LINC01538 CNTN4-AS1 TMEM26-AS1 NAV2-AS5 MAGI2-AS1 RBMS3-AS1 PABPC5-AS1 LINC02224 LINC01567 RERG-AS1 NAV2-AS3 MAPRE3-AS1 SLIT2-IT1 NR2F2-AS1 INHBA-AS1 C3orf67-AS1 SEMA6A-AS1 STARD13-AS LINC00387 PAPPA-AS1 TSPAN9-IT1 CLSTN2-AS1 MRPS30-DT LINC02334 TUB-AS1 SHANK2-AS1 KCNIP1-AS1 PACRG-AS2 LINC02035 LINC02030 LINC02151 LINC00457 CYP1B1-AS1 NCKAP5-IT1 LINC01293 LINC00564 LINC01677 RBMS3-AS2 LINC01255 ARHGAP31-AS1 LINC00498 WDFY3-AS2 FBXW7-AS1 LINC01238 LINC00472 VWC2L-IT1 LINC01344 LINC00305 LRP1-AS LINC02006 MAST4-IT1 LINC01490 LINC01801 LINC00862 TPRG1-AS2 ETV5-AS1 LINC02571*

		RERG-IT1 LINC02516 VLDLR-AS1 LINC02199 LINC01088 SLC16A12-AS1 PPP3CB-AS1 LINC00504 SLC14A2-AS1
linc-HEK293T	57	*SEPTIN7-AS1 LINC01516 LINC01114 LINC00652 LINC01963 MIR9-3HG CTNNA2-AS1 LINC00609 LINC02798 NKX2-2-AS1 LINC01141 LINC02282 RIC3-DT BDNF-AS LINC00298 LINC00437 LINC02318 LINC01727 ADGRL3-AS1 LINC01066 LINGO1-AS1 LINC02134 GRID1-AS1 LINC02549 LINC02440 LINC00906 NFIA-AS2 LINC02110 LINC01340 LINC02488 LINC00396 LINC01563 LINC00511 LINC02283 LINC01102 LINC02588 INKA2-AS1 EDNRB-AS1 LINC01397 FOXG1-AS1 MRVI1-AS1 LINC01103 MYLK-AS1 LINC00994 LINC02144 LINC02234 GDNF-AS1 NR2F1-AS1 LINC01349 LINC00237 LINC01152 SOX2-OT LINC02731 LINC01561 LINC00606 LINC02293 NDP-AS1*
linc-K562	21	*LINC01016 LINC02085 LINC02679 EIPR1-IT1 GPC5-AS2 IQCJ-SCHIP1-AS1 ZFAT-AS1 LINC00863 LINC01209 LINC00630 DENND6A-AS1 ENTPD1-AS1 LINC00654 TTC3-AS1 BRWD1-AS1 ZMYM4-AS1 GCC2-AS1 GPC5-IT1 ZBTB20-AS4 LINC01674 LINC01208*
linc-K562-diff.	99	*LINC02281 LINC02764 LINC02607 PACRG-AS3 LINC00349 LINC02427 KCND3-AS1 LINC01060 DAAM2-AS1 TCF7L1-IT1 PTPRD-AS1 BASP1-AS1 KCNIP4-IT1 FGF12-AS1 NAALADL2-AS2 LINC02306 LINC01266 RPS6KA2-IT1 LINC02510 LINC01865 LINC00320 CACNA1C-AS4 KLF7-IT1 LINC00698 LINC00327 OPCML-IT2 LINC02338 LINC00456 PTENP1-AS HIF1A-AS3 LINC01338 KAZN-AS1 LINC02389 DIRC3-AS1 LINC02239 LINC01998 NAALADL2-AS3 HTR5A-AS1 SBF2-AS1 FGF14-IT1 LINC01671 CCDC144NL-AS1 NLGN1-AS1 LINC02188 LINC02123 LINC02296 DLGAP2-AS1 LINC00571 WDR11-AS1 NPTN-IT1 LINC00641 ASTN2-AS1 LINC01581 CADM2-AS1 ATP2B2-IT2 LINC02330 LINC00622 GRM5-AS1 TMEM108-AS1 UFL1-AS1 TMEM132D-AS2 NREP-AS1 LINC01323 DPYD-AS1 THRB-AS1 RAPGEF4-AS1 LINC01600 CPEB2-DT ADGRF5-AS1 LINC00500 LINC01785 CADM3-AS1 NRG3-AS1 LINC02052 LINC02564 COL18A1-AS1 ATXN1-AS1 THSD4-AS1 KCNMA1-AS2 MYT1L-AS1 KIRREL3-AS3 LINC02693 PTPRD-AS2 LINC00943 LINC01320 GRM3-AS1 LINC02656 LINC02078 CD44-AS1 LINC02735 LINC02431 SNCA-AS1 PPM1K-DT TAB2-AS1 TEX26-AS1 WARS2-IT1 BARX1-DT LINC02047 LINC00844*

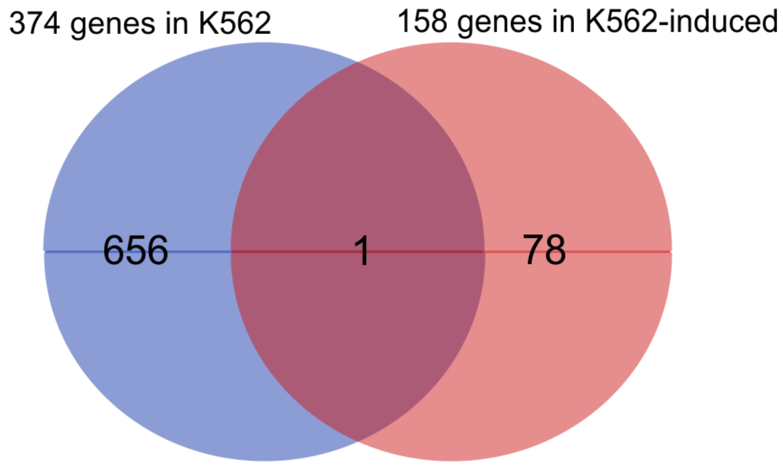

Figure S1. Venn diagram shows the detected intersection among lincRNAs associated with 374 mostly actively expressed genes in initial cells and the 158 genes enriched by repressed genes in K562-induced cells. Related to Figure 2A and 2E. There is only one common lincRNA – LINC02185.
